# Novel Lipophilic Hydroxamates Based on Spirocarbocyclic Hydantoin Scaffolds with Potent Antiviral and Trypanocidal Activity

**DOI:** 10.3390/ph16071046

**Published:** 2023-07-24

**Authors:** Vasiliki Pardali, Erofili Giannakopoulou, George Mpekoulis, Vassilina Tsopela, Georgios Panos, Martin C. Taylor, John M. Kelly, Niki Vassilaki, Grigoris Zoidis

**Affiliations:** 1 School of Health Sciences, Department of Pharmacy, Division of Pharmaceutical Chemistry, National and Kapodistrian University of Athens, Panepistimiopolis Zografou, 15771 Athens, Greece; vasilikipard@pharm.uoa.gr (V.P.); evgian@pharm.uoa.gr (E.G.); 2 Molecular Virology Laboratory, Hellenic Pasteur Institute, Vas. Sofias Avenue, 11521 Athens, Greece; g.mpekoulis@pasteur.gr (G.M.); vas.tsopela@gmail.com (V.T.); panosgeorgiosbio@gmail.com (G.P.); nikiv@pasteur.gr (N.V.); 3 Department of Infection Biology, London School of Hygiene and Tropical Medicine, Keppel Street, London WC1E 7HT, UK; martin.taylor@lshtm.ac.uk (M.C.T.); john.kelly@lshtm.ac.uk (J.M.K.)

**Keywords:** acetohydroxamic acid, antitrypanosomal agent, *T. brucei*, *T. cruzi*, antivirus agent, hydantoin, HCV, DENV, YFV, drug design and synthesis, SAR, NMR

## Abstract

*Flaviviridae* infections, such as those caused by hepatitis C (HCV) and dengue viruses (DENVs), represent global health risks. Infected people are in danger of developing chronic liver failure or hemorrhagic fever, both of which can be fatal if not treated. The tropical parasites *Trypanosoma brucei* and *Trypanosoma cruzi* cause enormous socioeconomic burdens in Sub-Saharan Africa and Latin America. Anti-HCV chemotherapy has severe adverse effects and is expensive, whereas dengue has no clinically authorized treatment. Antiparasitic medicines are often toxic and difficult to administer, and treatment failures are widely reported. There is an urgent need for new chemotherapies. Based on our previous research, we have undertaken structural modification of lead compound **V** with the goal of producing derivatives with both antiviral and trypanocidal activity. The novel spirocarbocyclic-substituted hydantoin analogs were designed, synthesized, and tested for antiviral activity against three HCV genotypes (1b, 3a, 4a), DENV, yellow fever virus (YFV), and two trypanosome species (*T. brucei*, *T. cruzi*). The optimization was successful and led to compounds with significant antiviral and trypanocidal activity and exceptional selectivity. Several modifications were made to further investigate the structure–activity relationships (SARs) and confirm the critical role of lipophilicity and conformational degrees of freedom.

## 1. Introduction

Human African trypanosomiasis (HAT), or sleeping sickness, and Chagas disease are major neglected tropical diseases (NTDs) with a devastating impact on public health, affecting populations mostly in low- or middle-income countries in Africa and Latin America, respectively [1,2]. HAT is caused by two distinct but morphologically indistinguishable subspecies of the hemoflagellate parasite *Trypanosoma brucei*, *T. b. rhodesiense,* and *T. b. gambiense,* which are transmitted to humans by the bite of infected tsetse flies. *T. b. rhodesiense* causes a fast-progressing disease that accounts for 2% of the total burden. *T. b. gambiense* is responsible for the majority of reported cases (98%) and causes a slow-progressing chronic infection [3].

**Figure 1:**
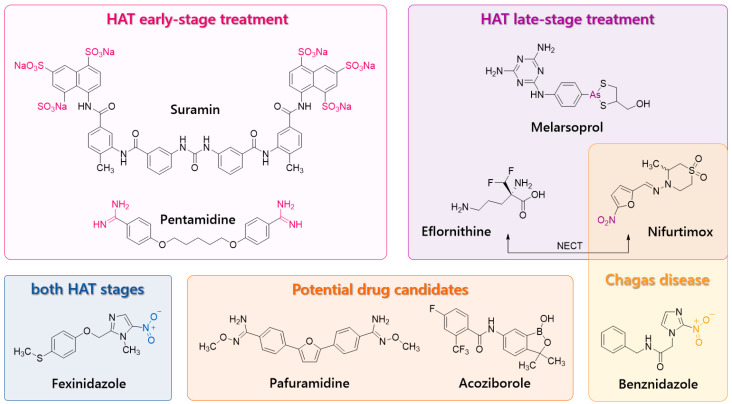
Current medications for the treatment of human African trypanosomiasis (HAT) and Chagas disease. The highlighted regions are reported to play a pivotal role in the mechanism of action.

Recently, as a result of public health measures, there has been a major reduction in the number of reported cases of HAT. However, the potential for epidemic outbreaks remains high, and trypanosome infection of domestic animals continues to impose a large economic burden. Problems with the currently used drugs include a high level of treatment failures, the requirement for parenteral administration, toxicity issues, and the necessity of drugs against stage 2 infections to penetrate the blood–brain barrier [4]. Recent drug discovery efforts for HAT have yielded pafuramidine, fexinidazole, and acoziborole (Figure 1) [5]. Of these, only the orally bioavailable fexinidazole has been approved for use against both stages of *T. b. gambiense* HAT [6]. Acoziborole is still in clinical trials, and the development of pafuramidine has been discontinued due to renal toxicity [7,8].

Infections by *Trypanosoma cruzi,* the causative agent of Chagas disease, are life-long and can be fatal owing to severe cardiac disorders and/or digestive tract pathology. Approximately 6 to 7 million individuals are infected, with at least 90 to 100 million people at risk [9,10]. Current chemotherapy comprises two nitroheterocyclic derivatives, benznidazole and nifurtimox. However, these require long periods of administration and exhibit toxicity, and treatment failure is a common outcome [11,12]. 

Infections with *Flaviviridae* viruses, such as hepatitis C (HCV), dengue (DENV), and yellow fever (YFV) viruses, are severe public health problems worldwide. HCV (Hepacivirus genus) is a main cause of chronic liver disease, with ~58 million individuals at risk of developing cirrhosis and hepatocellular carcinoma (HCC) [13]. The mosquito-borne DENV (Flavivirus genus) infects an estimated 400 million humans each year in over 100 countries, and YFV remains endemic in many parts of the world, despite the existence of an effective vaccine [14]. DENV and YFV lead to visceral and central nervous system diseases [15,16]. They result in a broad spectrum of clinical symptoms, ranging from asymptomatic infections and mild fevers to severe hemorrhagic fevers that can be life-threatening when left untreated [17,18]. In the case of DENV, there are four distinct serotypes, and secondary heterotypic infection is associated with an increased chance of developing severe disease [19]. *Flaviviridae* viruses have a positive-sense single-stranded RNA genome encoding a single polyprotein. This is processed into structural proteins, which are involved in receptor binding, virus fusion, and virion assembly, and non-structural (NS) proteins, which are responsible for the replication of the viral genome and critical for the evasion of host cell immune responses [20,21,22,23,24].

**Figure 2:**
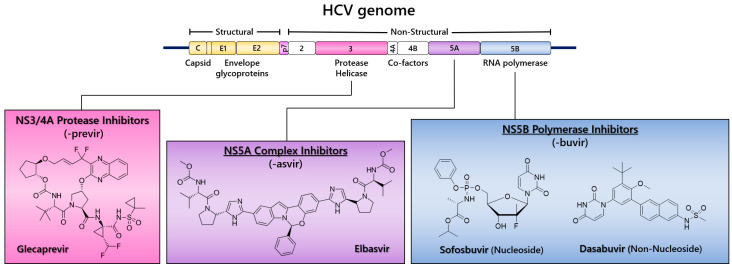
Chemical structures of representative clinically approved DAAs and their respective EC_50_s against HCV genotype 1b.

Until very recently, a standard of care for HCV was pegylated-interferon alpha (PEG-IFN-α) and ribavirin, but this had modest success rates and severe side effects, particularly for those patients with advanced liver disease [25]. The development of a cell culture system supporting the HCV full replication cycle and production of infectious virions, which is based on the Japanese genotype 2 strain (JFH-1) and modified hepatocyte cell lines [26], accelerated drug discovery, leading to the identification of effective direct-acting antiviral agents (DAAs). Since 2011, three main classes of DAAs have been introduced, targeting the HCV proteins NS3/4A serine-type protease, which is involved in the processing of the HCV polyprotein, NS5B, which is the viral RNA polymerase, and NS5A, which is essential for viral RNA replication and assembly [27]. In the last three years, several new DAA combinations have been approved for the treatment of the hepatitis C virus (HCV) infection, including elbasvir/grazoprevir, glecaprevir/pibrentasvir, ledipasvir/sofosbuvir, sofosbuvir/velpatasvir, and sofosbuvir/velpatasvir/voxilaprevir [28] (Figure 2). DAAs are able to attain sustained virologic responses in more than 90% of patients [29]. However, there is a high risk of the development of drug resistance, depending on the DAA regimen and the virus genotype [30]. The high genetic variation in HCV [31] influences the genetic barrier to drug resistance to DAAs [32]. This poses a major challenge for developing pangenotypic DAAs. Moreover, available DAAs are costly (several thousand euros per treatment), limiting their widespread clinical use; thus, therapy remains low on a global scale. Based on the above, there remains a need for novel effective drugs with a high resistance barrier and low cost. In the case of DENV and YFV, there is no approved antiviral therapy [33,34]. In the last 10 years, many antiviral compounds have been discovered, but only a few have been further evaluated in pre-clinical or clinical trials.

Here, in an extension of previous work, we report on a structure–activity relationship study that has led to the discovery of novel lipophilic hydroxamates that have enhanced antiviral and trypanocidal properties.

## 2. Results and Discussion

### 2.1. Design

**Figure 3:**
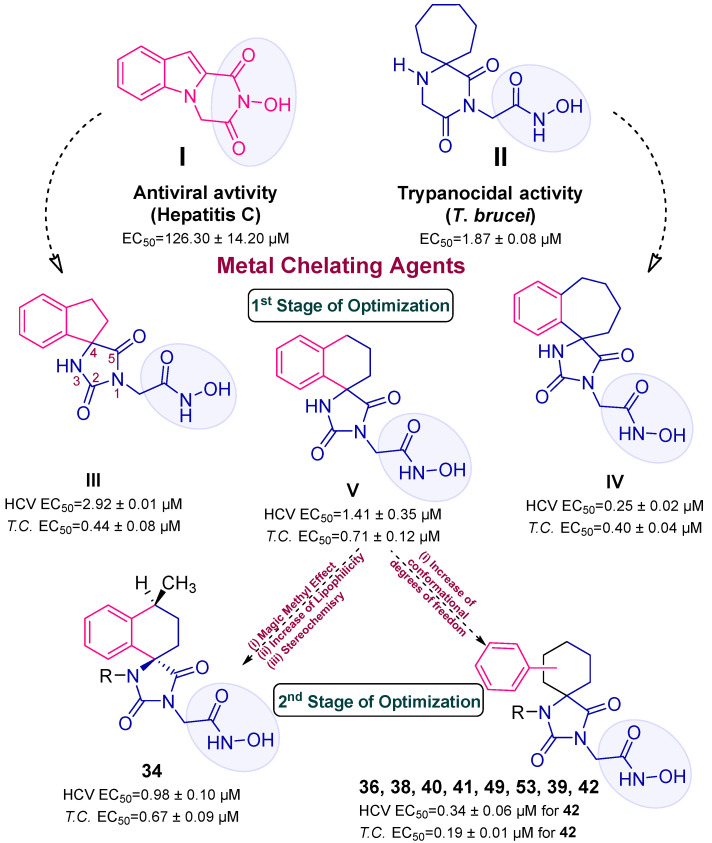
Indole-flutimide analog (**I**) [35] with anti-HCV activity, cycloheptane spiro-substituted 2,6-DKP analog (**II**) [36] with antitrypanosomal activity, and newly synthesized derivatives described in this study after two stages of optimization. Metal-binding groups are represented by elliptical plum shapes.

We previously reported that compounds containing metal-chelating moieties showed highly promising inhibitory efficacy against influenza A and HCV [35], as well as parasites of the *Trypanosoma* genus [36,37]. Although structurally diverse, the aforementioned series of analogs was based on the rational incorporation of metal-chelating moieties on scaffolds with pronounced drug-likeness, such as flutimide with the indole ring (**I**) or incorporation of the acetohydroxamic acid group on a 2,6-diketopiperazine (2,6-DKP) scaffold (**II**) (Figure 3). We designed analogs in pursuit of a conceptual approach to developing bioactive compounds with potential metal-chelating efficacy and inhibitory activity against both HCV viruses and *Trypanosoma* parasites. This was based on a combination of the exocyclic acetohydroxamic acid pharmacophore, which has been shown to have antitrypanosomal activity, and a lipophilic moiety based on a bicyclic system-substituted hydantoin that could effectively mimic the antiviral flutimide scaffold while having increased steric bulk [38], as shown comprehensively in Figure 3 (analogs **III**–**V**). 

To counteract potential water solubility concerns caused by antiviral lead **I**’s tricyclic system, we chose to improve the sp3 character of the new scaffold by introducing a spiro link between the acetohydroxamic acid group-carrying hydantoin and the indane system. In this scenario, mimicking spiro trypanocidal lead **II** would help to prevent potential Lipinski’s rule-of-five violations. As a result, the indole nitrogen atom was replaced by carbon, yielding a hydrophobic indane (compound **III**). Additionally, to evaluate the impact of the critical parameter of lipophilicity on biological activity, extensions of the indane ring to tetralin or benzocycloheptane (compounds **V** and **IV**) were considered, as was the addition of alkyl substituents such as methyl or benzyl groups on N3 of hydantoin. 

Based on the antiviral and antiparasitic results [38], we chose one of the most promising lipophilic scaffolds (tetralin **V**) for further optimization. On a second round of optimization, we added a methyl at the 4′ position of tetralin, seeking firstly for the ‘magic methyl effect’. It has been frequently observed that methylation reduces the free energy of desolvation required to undress a ligand of solvated molecules of water when it transfers from an aqueous environment to the lipophilic cavity of a protein. In this way, it energetically favors binding and lowers the EC_50_ value. Secondly, we rationalized that methylation of compound **V** would help us evaluate the contribution of the critical parameter of lipophilicity, which, from our experience on the SARs of this class of compound, favors both antiviral and antiparasitic activity [39]. To confirm the importance of the methyl’s stereochemistry, we separated the two pairs of enantiomers and tested them separately. 

To identify the structural features of the hydantoin-based acetohydroxamates required for potent antiviral and antitrypanosomal activity, we also modified the spirocarbocyclic hydantoin core structure by replacing the annulated aromatic component with an aryl substituent. We reasoned that the increased flexibility of the novel compounds might enhance the attainment of favorable aromatic interactions, leading to better activity and selectivity compared to the more constrained annulated counterparts with the planar shape and the less conformational degrees of freedom. 

**Scheme 1:**
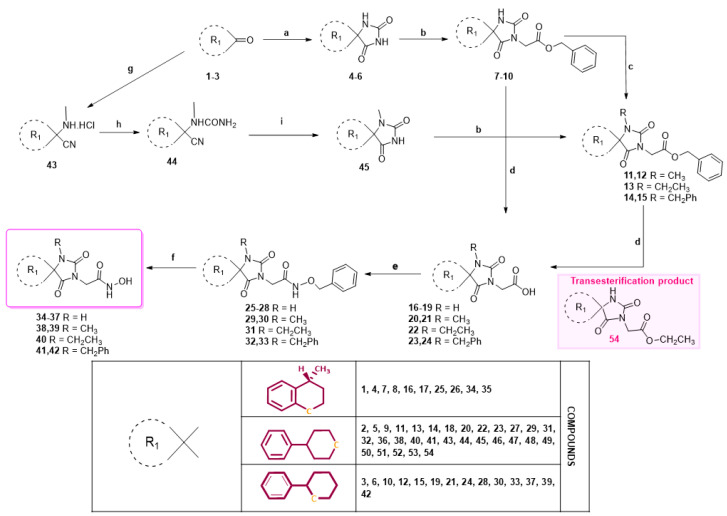
Synthesis of the target acetohydroxamic acid analogs **34**–**42**. Reagents and conditions: (a) **method A**: NaCN (2.27 eq.), (NH_4_)_2_CO_3_ (6 eq.), EtOH/H_2_O (1.1:1), reflux, 8 h–11 days, (30%); | **method B**: KCN (1.3 eq.), (NH_4_)_2_CO_3_ (5 eq.), EtOH/H_2_O (1:3), Microwave (MW) (100 W), 120 °C, 25 min–1 h, (84–90%); (b) (i) [(CH_3_)_3_Si]_2_NK (1.02 eq.), dry THF (0–5 °C), and then r.t., 20 min–1 h, argon; (ii) BrCH_2_COOCH_2_Ph (1.05 eq.), dry DMF, 35–38 °C, 48 h, argon, (71–91%); (c) (i) NaH (1.2 eq.), dry DMF, (0–5 °C), and then r.t., 15 min, argon; (ii) CH_3_I, or CH_3_CH_2_ (ethyl ester obtained because of unexpected transesterification reaction (pink frames)) or PhCH_2_Br (1.2 eq.), 60 °C, 7 days, argon (33–69%); (d) H_2_, Pd/C 10%, EtOH/AcOEt 3:1, 50–55 psi, 40–45 °C, 3 h, (almost quantitative yield); (e) **method A**: EDCI·HCl (1.2 eq.), HOBt (1.2 eq.), PhCH_2_ONH_2_·HCl (1.2 eq.), TEA (5.8 eq.), dry CH_2_Cl_2_/dry DMF, 30–35 °C, 40 h, argon, (30–60%); | **method B**: (i) CDI (1.2 eq.), dry THF, 28–30 °C, 1 h, argon; (ii) PhCH_2_ONH_2_·HCl (1.2 eq.), TEA (1.82 eq.), 28–30 °C, 24 h, argon, (44–74%); (f) H_2_, Pd/C 10%, EtOH/AcOEt 3:1, 50–55 psi, 40–45 °C, 3 h, (almost quantitative yield); (g) (i) NaCN, CH_2_NH_2_.HCl, DMSO/H_2_O, DMSO, 46 h, r.t.; (ii) HCl/EtOH (0 °C) (72%); (h) KOCN, AcOH/H_2_O 1–5 h, 35 °C (93%); (i) NaH (0 °C), dry DMF, 72 h, 50 °C, and then HCl (10%), 24 h, 50 °C, (52%).

In this paper, we describe the design and synthesis of a new series of acetohydroxamic acid derivatives (Scheme 1, **34**–**42**, **49,** and **53**) as *Flaviviridae* virus and *Trypanosoma* species inhibitors based on conformationally constrained 2,5-dioxo-imidazolidine scaffolds. The novel synthesized analogs were evaluated as trypanocidal and antiviral agents and exhibited significant inhibitory activity with respect to their parent compound **V**. Specifically, they were evaluated for their effect on RNA replication and cell viability of different HCV genotypes (1b, 3a, 4a), YFV and DENV. Their trypanocidal activity was evaluated based on their ability to inhibit the proliferation of cultured bloodstream from *T. brucei* and *T. cruzi* epimastigotes in vitro.

### 2.2. Chemistry

The synthesis of compounds **4**–**6** was achieved according to procedures similar to those reported in our previous publications [38,40], which are outlined in Scheme 1. The Bucherer–Bergs reaction was chosen as a starting point to afford the highly functionalized nitrogen-containing 5-membered hydantoin heterocycle. The commercially available ketones were readily converted to the desired hydantoins upon microwave-assisted reaction with ammonium carbonate and potassium cyanide in a one-pot step. Deprotonation of the imidic nitrogen of hydantoins **4**–**6** and **45** by potassium bis(trimethylsilyl)amide, followed by treatment of the resulting potassium imidate salts with benzyl bromoacetate or benzyl 2-bromopropanoate, afforded the corresponding benzyl esters **7**–**11** in moderate to good yields. The protective benzyl group was removed by catalytic hydrogenation in the presence of 10% Pd/C under mild conditions to yield the respective carboxylic acids **16**–**19**. One-pot amide coupling of the latter with O-benzylhydroxylamine hydrochloride in the presence of N-(3-dimethylaminopropyl)-N′-ethylcarbodiimide hydrochloride (EDCI·HCl), 1-hydroxybenzotriazole (HOBt), or 1,1′-carbonyldiimidazole (CDI) and a tertiary amine successfully led to the O-benzyl hydroxamates **25**–**28** and **48**. Hydrogenation of **25**–**28** and **48** over Pd/C gave rise to the desired acetohydroxamic acids almost quantitatively. In the ^1^H-NMR spectra, two sets of singlets were attributed to NH and OH groups for the two conformations of hydroxamic acids (Supplementary Materials). Our previous publication assigned E as the favorable conformation in DMSO-*d_6_* in a ratio of 3:1 [41]. In the case of N-hydroxy propanamides, the ^1^H NMR spectra showed a significant difference in E:Z ratio, calculated as 16:1 (Supplementary Materials).

**Scheme 2:**
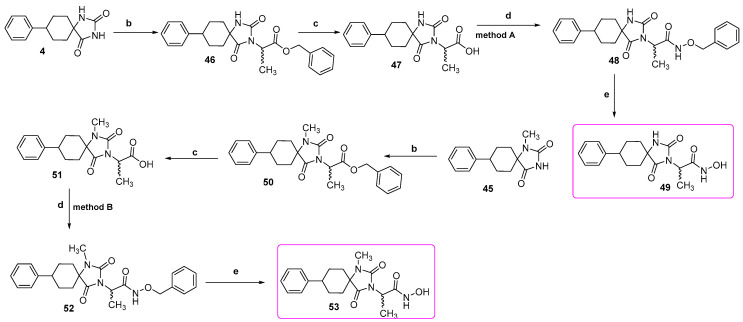
Synthesis of the target acetohydroxamic acid analogs **49** and **53**. Reagents and conditions: (b) (i) [(CH_3_)_3_Si]_2_NK (1.02 eq.), dry THF (0–5 °C), and then r.t., 20 min–1 h, argon; (ii) BrCH_2_COOCH_2_Ph (1.05 eq.), dry DMF, 35–38 °C, 48 h, argon (79%); (c) H_2_, Pd/C 10%, EtOH/AcOEt 3:1, 50–55 psi, 40–45 °C, 3 h, (almost quantitative yield); (d) **method A**: EDCI·HCl (1.2 eq.), HOBt (1.2 eq.), PhCH_2_ONH_2_·HCl (1.2 eq.), TEA (5.8 eq.), dry CH_2_Cl_2_/dry DMF, 30–35 °C, 40 h, argon, (30%); | **method B**: (i) CDI (1.2 eq.), dry THF, 28–30 °C, 1 h, argon; (ii) PhCH_2_ONH_2_·HCl (1.2 eq.), TEA (1.82 eq.), 28–30 °C, 24 h, argon (71%); (e) H_2_, Pd/C 10%, EtOH/AcOEt 3:1, 50–55 psi, 40–45 °C, 3 h, (almost quantitative yield).

1-Methyl hydantoin **45** was synthesized by a three-step synthetic procedure (Scheme 2). Starting from 4-phenylcyclohexan-1-one **2**, the respective aminonitrile **43**, as hydrochloride salt, was generated upon condensation with sodium cyanide and methylamine hydrochloride and subsequent treatment with a saturated ethanolic solution of gaseous HCl by employment of a three-component sequential Strecker reaction. Then, nucleophilic addition of potassium cyanate (KOCN) onto the aminonitrile **43** via a typical SN2 reaction led to the corresponding ureido derivative **44**. Cyclization of the latter with sodium hydride under mild heating and in situ acidic hydrolysis provided the target methyl derivative **45**. Using the previously described methodology, substitution with benzyl bromoacetate or benzyl 4-bromopropanoate, deprotection of the obtained benzyl esters **11** and **50**, amidation of the respective carboxylic acid (**20**), and catalytic hydrogenolysis yielded the methylated analog **38**.

It was possible to directly introduce a methyl group into the amidic nitrogen of the hydantoin core by applying a N-substitution reaction to form a new C-N bond (Scheme 1). The conducted N1-alkylation was achieved upon treatment of the precursor benzyl esters with sodium hydride and methyl iodide to yield the N1-methylated congeners. Following the same synthetic route, the N-ethylated or N-benzylated derivatives were also prepared using ethyl iodide or benzyl bromide as alkylating agents, respectively. Employment of the same reaction sequence yielded the desired N-alkylated hydroxamates **39**–**42** and **53**. It should be noted that in case of N-ethylation of the 4-phenylcyclohexanone hydantoin derivative, a mixture of products was afforded, which included products of a transesterification reaction. N-alkylation and transesterification products were isolated separately after chromatographic purification with the desired product **13** being the major component of the product mixture. The mechanism of this intriguing reaction has been previously described by our group [40]. No transesterification products were obtained with the more sterically hindered 2-phenylcyclohexanone.

### 2.3. NMR

In the ^1^H and ^13^C NMR spectra of 4-phenyl cyclohexane-substituted compounds, some characteristic peaks are observed, which can be attributed to the two different isomers. Each group can be found in an axial or an equatorial position. Hydantoin **5** is afforded as a mixture of two isomers, and the ratio was determined to be 9:1 (NMR spectra and analysis are in the Supplementary Materials File). The two possible conformations are one with the phenyl and the carbonyl groups in equatorial positions (and the NH group axial, α-isomer) and the other with the phenyl group equatorial and the carbonyl group axial (β-isomer). Structure elucidation was performed using routine 1D ^1^H, ^13^C and 2D gCOSY, gHSQC, gHMBC, and NOESY NMR techniques. In the major conformer, hydantoin has its phenyl and NH group in a *cis* relationship. In accordance with compound **5**, the respective benzylester **9** is formed preferentially with the same configuration in a 12:1 ratio. Two distinct ^1^H NMR resonances appeared for the protons of the two methylene groups and the amidic proton. In contrast to benzylester **9**, the *N*-alkylated products **20** and **22** exist predominantly in the form in which the phenyl group is in an equatorial position, and the *N*-alkyl substituent also adopts the equatorial orientation (*trans* configuration). The reason for this is that the axial isomer is not favorable due to steric 1,3-diaxial interactions assigned to the bulky substituents. 

### 2.4. Biology

#### 2.4.1. Trypanocidal Assays

##### Activity of the Compounds against *T. brucei*

All acetohydroxamic acid derivatives exhibited significant activity against *T. brucei*, with EC_50_s ranging from 0.009 to 4.41 μM. Compounds **41** and **40** were the most potent against African trypanosomes, with low nanomolar EC_50_ values (9 nM and 29 nM, respectively, Table 1), while hydroxamates **41**, **42,** and **40** were also significantly active against *T. cruzi* epimastigotes, with EC_50_ values in the sub and low micromolar range.

Changing the 4-phenyl cyclohexane ring in structure **36** to a 2-phenyl cyclohexane ring (compound **37**) decreased the activity, with the EC_50_ value of **37** being 5-fold higher (EC_50_ = 4.41 μM). In contrast, addition of a methyl group at position 4 of the tetralin ring led to compounds **34** and **35** (2 pairs of enantiomers), which were almost equipotent to lead compound **V** (EC_50_ **34** = 0.67 μM; EC_50_ **35** = 0.56 μM; EC_50_ **V** = 0.71 μM) [38]. Therefore, it seems that the stereochemistry of the tetralin ring does not play a critical role in trypanocidal activity. Both 2- and 4-phenyl cyclohexane derivatives appeared to confer a beneficial effect on trypanocidal potency, with respect to the structurally related tetralin analog **V** (EC_50_ = 0.71 μM), indicating that the more rigid fused bicyclic systems that were used as substituents at position 4 of the hydantoin core were less structurally favorable compared with the phenyl-substituted cyclohexane ring. 

Methyl substitution on the amide nitrogen atom of the respective 2,4-diketoimidazolidine residue of the parent compound **36** led to a 9-fold increase in potency for the 4-phenyl cyclohexane-substituted analog **38**. Moreover, incorporation of a methyl substituent in **49** and **37** enhanced the activity observed for the N-methylated analogs **53** and **39**, with EC_50_ values 0.154 μM and 1.46 μM, respectively, but to a lesser extent. Of note, the ethyl substitution on the amide nitrogen of the hydantoin ring significantly increased the activity of the parent compound **36** (30 times), as opposed to the methyl substitution of the acetohydroxamic carbon, which has no effect on the activity of the parent molecule **36** ((EC_50_ **36** = 0.88 μM; EC_50_ **49** = 0.88 μM (Table 1)). Thus, the ‘magic methyl effect’ occurred only when the installation of the methyl was on the hydantoin ring. 

Conversely, addition of the bulky lipophilic benzyl substituent to the amide nitrogen of the hydantoin ring yielded analogs that were extremely potent against *T. brucei*, as exemplified by compounds **41** and **42** (Table 1). Their activities were, respectively, 100 and 23 times higher than the parent compounds **36** and **37**. The N-benzylated analogs retained high potency, with EC_50_ values ranging between 9 and 191 nM (Table 1). This enhanced potency of analogs **41** and **42** reflects strongly favorable stereoelectronic and hydrophobic effects exerted by the benzyl substituent in the binding site. 

The cytotoxicity of all target compounds against mammalian cells was determined using the rat skeletal myoblast L6 cell line. Notably, the non-substituted analogs **34**, **35**, **37**, and **49** displayed very low cytotoxicity against mammalian cells, having CC_50_s over 200 μM, thus resulting in remarkable selectivity indices, which varied from 55 (**37**) to 400 (**35**). In general, it was observed that the incorporation of an alkyl substituent onto the amide nitrogen atom progressively increased the cytotoxicity of the compounds, except for compound **39** (CC_50_ = 217 μM, SI 150). Finally, the acetohydroxamic acid derivative **40**, which had the second highest activity against *T. brucei* (EC_50_ = 0.029 μM) displayed an excellent selectivity (SI 1870, one of the highest reported), despite the ethyl substitution.

##### Activity of the Compounds against *T. cruzi*

Compounds were also tested against cultured *T. cruzi* epimastigotes, with EC_50_ values at submicromolar to micromolar levels (0.34–8.50 μΜ) (Table 1). Acetohydroxamic acid derivatives with a substituent on the amide nitrogen atom were able to inhibit parasite growth, an effect that was not observed with the non-substituted derivates. More specifically, compounds **34**–**37** and **49** exhibited no activity at the highest concentration tested (30 μM), whereas the N-methylated analogs showed medium inhibitory activity (EC_50_ **38** = 6.25 μM; EC_50_ **53** = 8.5 μM), with the only exception being analog **39**. Crucially, incorporation of an ethyl or benzyl group resulted in highly potent analogs. The EC_50_s ranged from 1.12 μM for the ethyl-substituted analog **40** to 0.34 μM for the benzyl-substituted analog **41**, indicating a positive effect of increasing the bulkiness and lipophilicity of the compounds, a pattern also observed in *T. brucei* sp. Interestingly, the ethyl-substituted analog **40** also exhibited the best selectivity also against *T. cruzi* (SI = 48, Table 1).

#### 2.4.2. Biological Evaluation of the Compounds against *Flaviviridae* Viruses

##### Screening of Compounds in the HCV Genotype 1b Replicon System

Previously, we investigated the activity of several fused bicyclic hydantoin analogs (lead compound **V**, Figure 3), rationally designed to target HCV RdRp. Based on these results, in the present study, our aim was to examine the anti-*Flaviviridae* activity of a new series of compounds that contain different substitutions on the core scaffold. 

**Figure 4:**
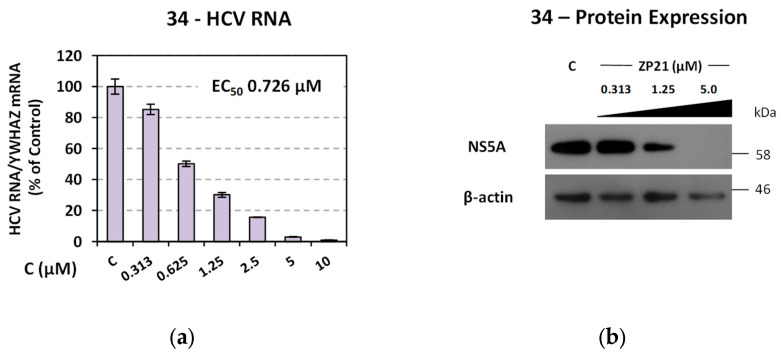
Inhibition profile of **34** on HCV RNA levels and protein expression in a subgenomic 1b (Con1) replicon assay. Huh5-2 cells were treated with serial dilutions of **34** or the solvent DMSO (control-C). (**a**) Quantification of (+) strand HCV RNA by RT-qPCR. Values from compound-treated cells are expressed as percentage of those derived from cells treated with the solvent (control). The housekeeping gene 14-3-3-zeta polypeptide (YWHAZ) mRNA was used for normalization. Bars represent mean values obtained from three separate experiments in triplicate. Error bars represent standard deviation (SD). (**b**) Western blot analysis for NS5A. β-actin was used as loading control. A representative of three independent experiments is shown.

First, we evaluated the efficacy of the compounds against HCV replication and, in parallel, their impact on cell viability in the stable cell line Huh5-2, harboring an HCV genotype 1b (strain Con1) subgenomic replicon. This viral construct expresses the reporter firefly luciferase (F-Luc) protein under the translational control of the HCV IRES, which functions as a marker of viral RNA replication. ATP cellular content was used as an indicator of viable and metabolically active cells. The effects of serial dilutions of the compounds on viral replication-driven luciferase activity and intracellular ATP levels were quantified and used to determine their median or half-maximal effective concentrations (EC_50_) and their median cytotoxic concentrations (CC_50_), respectively (Table 2). Selectivity indices (SI) = CC_50_/EC_50_ were also calculated. Dose–response curve analysis is presented for the potent analog showing the highest selectivity index (34) (Figure 4).

##### Comparing the Anti-HCV Intergenotypic Activity of the Compounds

The activity of the compounds was further characterized in other HCV genotypes. Specifically, we evaluated their effect on viral replication-derived luciferase activity in Huh7.5-3a and Huh7.5-4a stable cell lines containing subgenomic replicons of HCV GT 3a (strain S52) and GT 4a (strain ED43), respectively (Table 2). 

**Figure 5:**
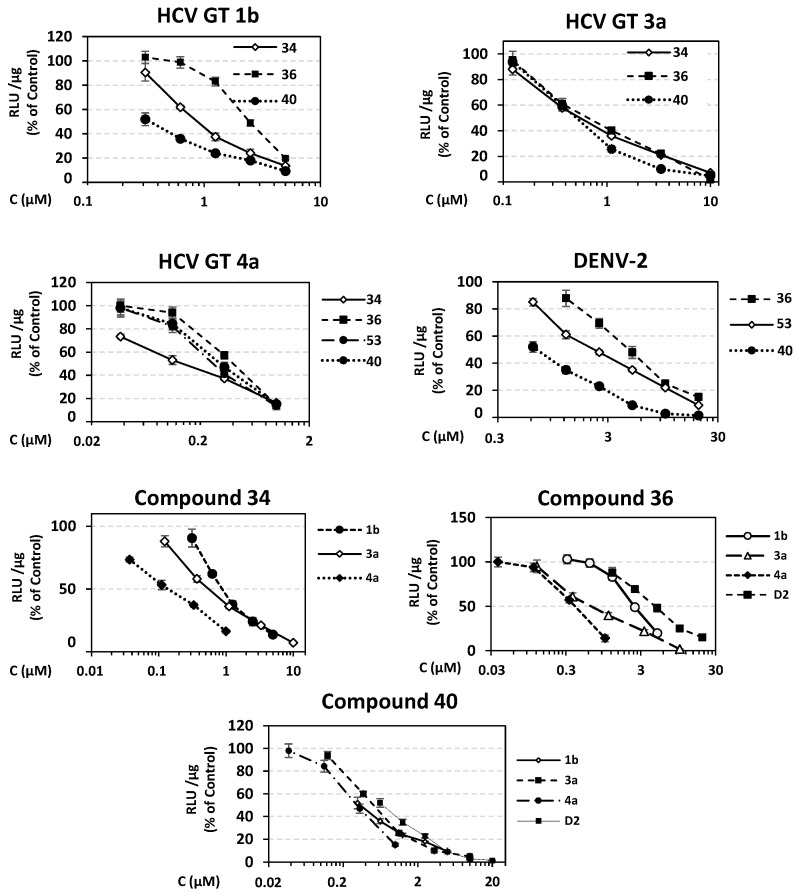
Dose–response curve analysis of the most promising compounds. Replicon cells of HCV GT 1a, GT 3a, GT 4a, or DENV-2 were treated with serial dilutions of compounds for 72 h. F-Luc activity was quantified and normalized to total protein. Values from the compound-treated cells are expressed as a percentage of those obtained with DMSO-only-treated cells (control).

As shown in Table 2, acetohydroxamic acid analogs were potent against HCV-1b replication, with EC_50_ values ranging from 0.34 to 8.66 μM. Compounds **34**, **40,** and 2-phenyl cyclohexane analog **42** exhibited the highest potency against HCV GT 1b, with EC_50_s in the submicromolar range (EC_50_ 0.98 μM, 0.34 μΜ, and 0.74 μM, respectively). Compounds **34** and **40** were also very effective against both GT 3a (EC_50_ 0.63 μΜ and 0.52 μΜ, respectively) and GT 4a (EC_50_ 0.14 μΜ and 0.31 μΜ, respectively), while **42** was also active against GT 4a (EC_50_ 0.45 μΜ). Interestingly, compounds **35** and **53**, which showed marginal activity against GT 1b (7.31 μΜ and 8.66 μΜ, respectively), were very potent against GT 4a (EC_50_ 0.41 μΜ and 0.28 μΜ, respectively), while **35** was also active against GT 3a (EC_50_ 0.77 μΜ). Moreover, **36** and **38** which showed moderate activity against GT 1b (2.53 μΜ and 2.75 μΜ, respectively), were very potent against both GT 3a (EC_50_ 0.75 μΜ and 0.63 μΜ, respectively) and GT 4a (EC_50_ 0.39 μΜ and 0.22 μΜ, respectively). Compounds **34**, **38,** and **40** were the most active of the compounds against GT 3a, with a high selectivity index in the case of **34** (SI = 318). Finally, the 4-methyl tetralin analog **34** was also the most active against GT 4a with a very high selectivity index (SI = 1429), being 2.5-fold more active than the structurally related lead molecule **V**. In the case of anti-HCV activity, the ‘magic methyl effect’ was present regardless of the position of the methylation. For the compounds that showed the highest activity based on their EC_50_ values with concomitant high selectivity indices, we performed a dose–response curve analysis (Figure 5). The activity of the compounds against 3a is of particular importance, as patients with genotype 3 infection are the most difficult to treat, especially in patients with cirrhosis [42].

##### Screening of Compounds against DENV and YFV Replicon Systems

Taking into account that the fused bicyclic hydantoin analogs (lead compound **V**, Figure 3) were rationally designed to target *Flaviviridae* RdRp, which is well conserved among HCV and Flaviviruses [43,44], next, we aimed to study their activity against DENV and YFV RNA replication. Specifically, we measured viral replication-derived luciferase activity in Huh7-D2 and Huh7-YF stable cell lines harboring the subgenomic replicon of DENV serotype 2 (strain 16681) or YFV (strain 17D).

In the case of DENV, 4-phenyl cyclohexane analog **40** was the most active compound, followed by **42** and **53**. Moderate potency was displayed by **36** and **39**. For YFV replication, only analogs **42**, **40,** and **49** showed marginal activity and selectivity.

##### Validation of Compound Activity with Additional Assays

The inhibition profile of **34** against HCV GT1b in Huh5-2 cells, estimated above by the luciferase assay, was further evaluated at the level of viral RNA and protein expression by performing reverse transcription-quantitative polymerase chain reaction (RT-qPCR) or Western blot analysis, respectively. We observed that **34** reduced HCV RNA amounts (Figure 4a), with an EC_50_ similar to that estimated on the basis of virus-derived luciferase activity (Table 2). A consistent negative effect was shown on the levels of the viral NS5A protein (Figure 4b).

##### Combinatory Effect of Compound 34 with Daclatasvir (DCV)

**Figure 6:**
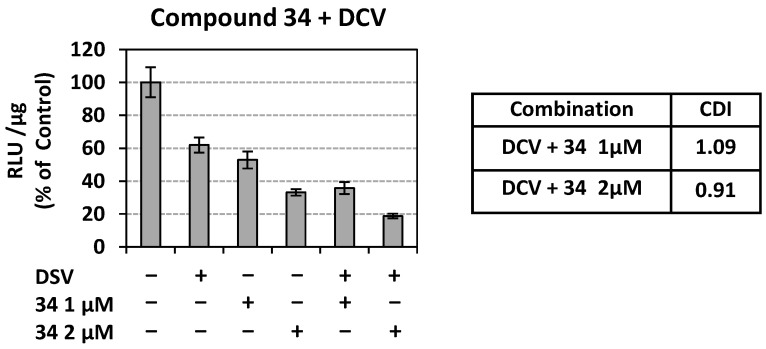
Combinatory activity of **34** with DCV against HCV GT 1b (Con1). Huh5-2 cells were treated or not with the analog in the presence or absence of 20 pM DCV. F-Luc activity was measured and calculated as relative light units (RLU) per μg of total protein. Values were expressed as a percentage of those derived from cells treated with the solvent DMSO (control-C). CDI: coefficient of drug interaction. Bars represent mean values obtained from three separate experiments in triplicate. Error bars represent standard deviation (SD).

The Huh5-2 replicon cell line was treated with compound **34** in the presence or absence of daclatasvir (DCV) to test its effectiveness in combination with an authorized NS5A inhibitor (DCV) (Figure 6). The two drugs had an additive impact, as determined by the coefficient of drug interaction (CDI ≈ 1).

## 3. Materials and Methods

### 3.1. Experimental Methods

Melting points were determined using a Büchi capillary apparatus and were uncorrected. The melting point of the molecules is reported with the solvent system (e.g., EtOH/*n*-pentane) used for their recrystallization. NMR experiments were performed to elucidate the structure and determine the purity of the newly synthesized compounds. Microwave synthesis was carried out on a Start E, Milestone microwave reactor. Catalytic hydrogenations were conducted using a Paar Shaker Hydrogenation Apparatus. ^1^H NMR and 2D NMR spectra (COSY, HSQC, HSQC-DEPT, and HMBC) were recorded on a Bruker DRX400 spectrometer (400.13 MHz, ^1^H NMR) and a Bruker Ultrashield™ Plus Avance III 600 spectrometer (600.11 MHz, ^1^H NMR). ^13^C NMR and DEPT NMR spectra were recorded on a Bruker Avance 200 spectrometer (50.32 MHz, ^13^C NMR), a Bruker DRX400 spectrometer (100.61 MHz, ^13^C NMR), and a Bruker Ultrashield™ Plus Avance III 600 spectrometer (150.9 MHz, ^13^C NMR). Chemical shifts *δ (delta)* are reported in parts per million (ppm) downfield from the NMR solvent, with the tetramethylsilane or deuterated solvent as the internal standard. The solvents used to obtain the spectra were: deuterated chloroform, CDCl_3_ (s, 7.26 ppm, ^1^H NMR; t, 77.16 ppm, ^13^C NMR), and deuterated dimethyl sulfoxide, DMSO-*d_6_* (quin, 2.50 ppm, ^1^H NMR; septet, 39.52 ppm, ^13^C NMR). The spectra were recorded at 293 K (20 °C) unless otherwise specified. Data processing, including Fourier transformation, baseline correction, phasing, peak peaking, and integrations, were performed using MestReNova software v.12.0.0. Splitting patterns are designated as follows: s, singlet; br s, broad singlet; d, doublet; br d, broad doublet; t, triplet; q, quartet; dd, doublet of doublets; ddd, doublet of doublets of doublets; dddd, doublet of doublets of doublets of doublets; ddddd, doublet of doublets of doublets of doublets of doublets; dt, doublet of triplets; dq, doublet of quartets; ddt, doublet of doublets of triplets; ddq, doublet of doublets of quartets; dtt, doublet of triplets of triplets; dqt, doublet of quartets of triplets; ddtd, doublet of doublets of triplets of doublets; td, triplet of doublets; tdd, triplet of doublets of doublets; qt, quartet of triplets; m, multiplet; complex m, complex multiplet. Coupling constants (*J*) are expressed in units of Hertz (Hz). Analytical thin-layer chromatography (TLC) was used to monitor the progress of the reactions, as well as to authenticate the compounds. TLCs were conducted on precoated with normal-phase silica gel, aluminum sheets (Silica gel 60 F_254_, Merck) (layer thickness 0.2 mm). Developed plates were examined under a UV light source at wavelengths of 254 nm and 365 nm or after being stained by iodine vapors. The retention factor (*R*_f_) of the newly synthesized compounds, which equals the distance migrated over the total distance covered by the solvent, was also measured on the chromatoplates. Column chromatography was used to isolate the desired compounds from by-products of reactions, and it was carried out using elution solvents of increasing polarity in a stationary phase of SiO_2_ (Silica gel 60, 70–230 mesh ASTM). Column packing was performed using the slurry method, while the mixtures to be analyzed were loaded using either the technique of dry deposition or as a solution in the first eluent where this was possible. Elemental analyses (C, H, N) were performed by the Service Central de Microanalyse at CNRS (France) and were within ±0.4% of the theoretical values. Elemental analysis results for the tested compounds correspond to ˃95% purity. The commercial reagents were purchased from Alfa Aesar, Sigma-Aldrich, and Merck and were used without further purification, except for the benzyl bromoacetate. This reagent was purified by fractional distillation in vacuo prior to use. Benzyl 2-bromopropanoate was synthesized according to the reported method. Organic solvents used were of the highest purity and, when necessary, were dried by standard methods. Solvent and reagent abbreviations: AcOEt, ethyl acetate; CDI, 1,1′-carbonyldiimidazole; DMF, *N*,*N*-dimethylformamide; DMSO, dimethyl sulfoxide; EDCI·HCl, *N*-(3-dimethylaminopropyl)-*N*′-ethylcarbodiimide hydrochloride; Et_2_O, diethyl ether; EtOH, ethanol; HOBt, 1-hydroxybenzotriazole; MeOH, methanol; TEA, triethylamine; THF, tetrahydrofuran.

### 3.2. General Experimental Procedures

General experimental procedure for the synthesis of hydantoins. Method A: A mixture of the ketone (5.0 mmol, 1.0 eq) in EtOH (5 mL), sodium cyanide (11.35 mmol, 2.27 eq), and ammonium carbonate (30.0 mmol, 6.0 eq) in H_2_O (4 mL) was refluxed for 6 h, at 100–120 °C. The reaction mixture was allowed to cool at rt, and ethanol was evaporated under reduced pressure. The residue was diluted with 15 mL H_2_O and acidified with conc. HCl to pH~2–3 under ice cooling. The solid formed was filtered off, washed with portions of H_2_O and Et_2_O, and dried over P_2_O_5_. Method B: A microwave reaction vessel containing a stirrer bar was charged with the ketone (5 mmol, 1.0 eq), ammonium carbonate (25 mmol, 5.0 eq), and potassium cyanide (6.5 mmol, 1.3 eq) in EtOH/H_2_O 1:3 (14 mL). The vessel was sealed, placed in a Milestone Microwave Apparatus, and allowed to react under irradiation for 25 min to 1 h at 120 °C at 100 W. Then, the vessel was cooled to rt and washed with EtOH/H_2_O (2:1). Ethanol was concentrated in vacuo, and the residual was acidified with a 10% HCl solution until pH~2–3 at 0 °C. The precipitate formed was filtered off, washed with H_2_O to remove the inorganic salts and Et_2_O, and dried over P_2_O_5_ to yield the desired hydantoin.

4′-methyl-3′,4′-dihydro-2*H*,2′*H*,5*H*-spiro[imidazolidine-4,1′-naphthalene]-2,5-dione (4). To a solution of 4-methyl-3,4-dihydro-2*H*-naphthalen-1-one 1 (3.0 g, 18.72 mmol) in 17.5 mL EtOH, a mixture of (NH_4_)_2_CO_3_ (10.79 g, 112.32 mmol) and NaCN (2.08 g, 42.49 mmol) in H_2_O (16 mL) was added. The mixture was refluxed at 95–100 °C for 6 h. The reaction mixture was then worked up following the general procedure described for the synthesis of hydantoins (Method A). The residual was extracted with AcOEt (3 × 180 mL), the combined organic extracts were washed with H_2_O (2 × 120 mL) and brine (2 × 120 mL) and dried over anh. Na_2_SO_4,_ and the solvent was evaporated under vacuum. The obtained viscous, off-yellow oil was purified by column chromatography on silica gel with CH_2_Cl_2_/AcOEt 10:1, 5:1, and AcOEt as eluents to afford **4** as a white crystalline solid. (1.3 g, 30%); *R*_f_ = 0.21 (CH_2_Cl_2_/AcOEt 5:1); mp 196–198 °C (EtOH/dry Et_2_O-*n*-pentane); ^1^H NMR (400.13 MHz, [D_6_]DMSO): *δ* (ppm) = 1.26, 1.27 (d + d, *J* = 7.0 Hz, *J* = 6.9 Hz, 3H, C*H_3_*), 1.52–1.62 (m, 0.55H, H_3′_), 1.77–1.90 (m, 1H, H_2′_, H_3′_), 1.91–2.06 (complex m, 1.5H, H_2′_, H_3′_), 2.13–2.27 (complex m, 1H, H_2′_, H_3′_), 2.85–2.96 (m, 1H, H_4′_), 7.05 (d, *J* = 7.7 Hz, 1H, H_8′_), 7.20 (td, *J_1_* = 7.4 Hz, *J_2_* = 1.8 Hz, 1H, H_7′_), 7.27 (td, *J_1_* = 7.8 Hz, *J_2_* = 1.2 Hz, 1H, H_6′_), 7.30 (d, *J* = 7.4 Hz, 1H, H_5′_), 8.51, 8.53 (s + s, 1H, H_3_), 10.83 (s, 1H, H_1_); ^13^C NMR (50.32 MHz, [D_6_]DMSO): *δ* (ppm) = 22.2 (*C*H_3_), 25.8, 26.3 (C_3′_), 30.3, 30.6 (C_2′_), 31.3, 31.6 (C_4′_), 63.3 (C_4_), 126.4 (C_7′_, C_8′_), 128.1, 128.2, 128.4 (C_5′_, C_6′_), 133.9 (C_8′a_), 142.6, 142.8 (C_4′a_), 156.5 (C_2_=O), 177.8 (C_5_=O); elemental analysis calcd (%) for C_13_H_14_N_2_O_2_: C 67.81, H 6.13, N 12.17, found: C, 67.88, H 6.23, N 12.21. 

8-Phenyl-1,3-diazaspiro [4.5]decane-2,4-dione (5) [45]. 4-Phenyl cyclohexanone **2** (900 mg, 5.17 mmol) was dissolved in 4 mL of EtOH and mixed with a solution of (NH_4_)_2_CO_3_ (2.48 g, 25.85 mmol) and KCN (438 mg, 6.72 mmol) in 12 mL H_2_O. The total reaction mixture was irradiated in a closed vessel Milestone Microwave Apparatus (100 W), for 25 min at 120 °C. A wash solution of EtOH/H_2_O (2:1) was used to clean the reaction vessel and then ethanol was evaporated under reduced pressure. The crude residue was acidified with 10% HCl (pH~2–3) under ice cooling and cooled at 5 °C for 1 h. The solid formed was filtered off in vacuo and washed with H_2_O and Et_2_O sequentially and dried over P_2_O_5_ to afford the hydantoin **5** as a white crystalline solid (1.13 g, 90%). Mp > 250 °C (AcOEt/*n*-pentane), R*_f_* = 0.18 (CH_2_Cl_2_/AcOEt 5:1). ^1^H NMR (400.13 MHz, DMSO-*d_6_*) *δ* (ppm) 1.63 (~d, 2H, *J* = 9.4 Hz, H_6a_, H_10a_, *cis*), 1.67–1.84 (m, 6H, H_6e_, H_7_, H_9_, H_10e_, *cis*), 1.90 (d, 0.45H, *J* = 12.7 Hz, H_6a_, H_10a_, *trans*), 2.11 (q, 0.4H, *J* = 12 Hz, H_7_, H_9_, *trans*), 2.45–2.61 (m, 1H, H_8_), 7.14 (td, 1H, *J_1_* = 7.4 Hz, *J_2_* = 1.5 Hz, H_4′_), 7.22–7.36 (m, 4H, H_2′_, H_3′_, H_5′_, H_6′_), 7.92 (s, 0.17H, H_1_, *trans*), 8.69 (s, 0.85H, H_1_, *cis*), 10.58 (s, 1H, H_3_); ^13^C NMR (50.32 MHz, DMSO-*d_6_*) *δ* (ppm) 28.5 (C_7_, C_9_), 33.4 (C_6_, C_10_, *cis*), 34.0 (C_6_, C_10_, *trans*), 41.3 (C_8_*, trans*), 42.3 (C_8_, *cis*), 59.6 (C_5_, *trans*), 61.8 (C_5_, *cis*), 126.0 (C_4′_), 126.7 (C_2′_, C_6′_, *trans*), 127.0 (C_2′_, C_6′_, *cis*), 128.2 (C_3′_, C_5′_), 146.2 (C_1′_, *trans*), 146.7 (C_1′_, *cis*), 156.0 (*C_2_*=O, *trans*), 156.4 (C_2_=O, *cis*), 178.4 (C_4_=O, *trans*), 178.6 (C_4_=O, *cis*). Anal. Calcd for C_14_H_16_N_2_O_2_: C, 68.83; H, 6.60; N, 11.47. Found: C, 68.75; H, 6.65; N, 11.50.

6-Phenyl-1,3-diazaspiro [4.5]decane-2,4-dione (6). To a solution of 2-phenylcyclohexanone 3 (1000 mg, 5.74 mmol) in 4 mL EtOH was added a solution of (NH_4_)_2_CO_3_ (2.76 g, 28.7 mmol) and KCN (486 mg, 7.46 mmol) in 12 mL H_2_O, and the resultant stock slurry was stirred at room temperature. The mixture was processed in a Milestone Microwave Apparatus and irradiated in a closed vessel (100 W) for 1 h at 120 °C. A wash solution of EtOH/H_2_O (2:1) was used to clean the reaction vessel, and ethanol was evaporated under reduced pressure. The residue was acidified with 10% HCl (pH~2–3) under ice cooling. The resulting mixture was cooled at 5 °C for 1 h and filtered off in vacuo to yield a white precipitate, which was washed with H_2_O and Et_2_O sequentially and dried over P_2_O_5_ to afford the title compound **6** as a white crystalline solid (1.180 mg, 84%). Mp 244–246 °C (EtOH/*n*-pentane), R*_f_* = 0.26 (CH_2_Cl_2_/AcOEt 5:1). ^1^H NMR (600.11 MHz, DMSO-*d_6_*) *δ* (ppm): 1.43 (qt, 1H, *J_1_* = 13.1 Hz, *J_2_* = 3.5 Hz, H_8_), 1.53 (tt, 1H, *J_1_* = 12.6 Hz, *J_2_* = 3.1 Hz, H_9_), 1.59 (dd, 1H, *J_1_* = 13.1 Hz, *J_2_* = 3.4 Hz, H_7_), 1.63–1.71 (m, 2H, H_9_, H_10_), 1.74–1.84 (m, 2H, H_8_, H_10_), 1.97 (qd, 1H, *J_1_* = 13.2 Hz, *J_2_* = 3.6 Hz, H_7_), 2.90 (dd, 1H, *J_1_* = 13.4 Hz, *J_2_* = 3.5 Hz, H_6_), 7.13–7.17 (m, 2H, H_2′_, H_6′_), 7.19 (~tt, 1H, *J_1_* = 7.2 Hz, *J_2_*≈1.7 Hz, H_4′_), 7.21–7.27 (m, 2H, H_3′_, H_5′_), 8.54 (s, 1H, H_1_), 10.09 (s, 1H, H_3_); ^13^C NMR (150.9 MHz, DMSO-*d_6_*) *δ* (ppm): 20.7 (C_9_), 25.3 (C_8_), 27.2 (C_7_), 34.5 (C_10_), 47.5 (C_6_), 66.3 (C_5_), 126.8 (C_4′_), 127.7 (C_3′_, C_5′_), 128.6 (C_2′_, C_6′_), 140.0 (C_1′_), 156.4 (C_2_=O), 177.2 (C_4_=O). Anal. Calcd for C_14_H_16_N_2_O_2_: C, 68.83; H, 6.60; N, 11.47. Found: C, 68.90; H, 6.62; N, 11.55.

### 3.3. Experimental Procedure for the Synthesis of Methyl Hydantoin (45)

1-(Methylamino)-4-phenylcyclohexane-1-carbonitrile hydrochloride (43). To a stirred suspension of sodium cyanide (843 mg, 17.2 mmol) and methylamine hydrochloride (1.16 g, 17.2 mmol) in 12 mL of DMSO/H_2_O 9:1 (*v*/*v*), a solution of 4-phenylcyclohexanone X (3.0 g, 17.2 mmol) in DMSO (24 mL) was added in one portion. The reaction mixture was stirred for 46 h at rt, poured into 130 mL of an ice-water mixture, and extracted with AcOEt (3 × 60 mL). The combined organic phases were washed with brine (2 × 60 mL) and dried with anh. Na_2_SO_4,_ and the solvent were evaporated under reduced pressure. The residue was dissolved in Et_2_O (80 mL) and treated dropwise with an ethanolic solution saturated with gaseous hydrochloric acid to pH~2 under an ice bath. The precipitate formed was filtered off in vacuo, washed with small portions of dry Et_2_O, and dried over P_2_O_5_ to afford the title compound **43** as a white crystalline solid (3.2 g, 72%). This intermediate was used for the next reaction without further purification.

1-(1-Cyano-4-phenylcyclohexyl)-1-methylurea (44). To a stirred solution of the carbonitrile **43** (3.1 g, 12.4 mmol) in 20 mL acetic acid, a solution of potassium cyanate (2.01 g, 24.8 mmol) in 3 mL H_2_O was added. After stirring for 1 h at 35 °C, the reaction mixture was poured into 70 mL H_2_O and extracted with CHCl_3_ (3 × 50 mL). The combined organic layers were washed with H_2_O (3 × 50 mL) and brine (2 × 50 mL) and dried with anh. Na_2_SO_4,_ and the solvents were evaporated to dryness under reduced pressure to afford the title compound **44** as a white solid (2.97 g, 93%). This intermediate was used for the next reaction without further purification.

1-Methyl-8-phenyl-1,3-diazaspiro [4.5]decane-2,4-dione (45). A stirred solution of 44 (2.9 g, 11.3 mmol) in 40 mL dry DMF was cooled in an ice bath, and sodium hydride (353 mg, 14.7 mmol, 60% dispersion in mineral oil) was added portionwise. After 4 d of stirring at 50 °C under argon, the mixture was treated with a solution of HCl 10% (96 mL), and stirring was continued for 24 h at 50 °C. After this time, the reaction mixture was poured into 400 mL of an ice-water mixture and extracted with CHCl_3_ (4 × 200 mL). The combined organic extracts were washed with H_2_O (3 × 250 mL) and brine (2 × 250 mL) and dried with anh. Na_2_SO_4,_ and the solvent were evaporated in vacuo. The remaining solid was treated with Et_2_O and *n*-pentane to yield the desired compound **45** as a pale-yellow crystalline product. (2.98 g, 67%); *R*_f_ = 0.34 (CH_2_Cl_2_/AcOEt 5:1); mp 211–214 °C (AcOEt/dry Et_2_O-*n*-pentane); ^1^H NMR (400.13 MHz, CDCl_3_): *δ* (ppm) = 1.80–1.94 (complex m, 6H, H_6_, H_7_, H_9_, H_10_), 2.31–2.45 (complex m, 2H, H_7_, H_9_), 2.52 (tt, *J_1_* = 11.7 Hz, *J_2_* = 2.9 Hz, 1H, H_8_), 2.87 (s, 3H, C*H_3_*), 7.22 (td, *J_1_* = 6.5 Hz, *J_2_* = 1.9 Hz, 1H, H_4′_), 7.30 (d, *J* = 7.1 Hz, 2H, H_2′_, H_6′_), 7.32 (t, *J* = 7.9 Hz, 2H, H_3′_, H_5′_), 9.27 (s, 1H, H_3_); ^13^C NMR (150.9 MHz, CDCl_3_): *δ* (ppm) = 23.6 (*C*H_3_), 28.3 28.7 (C_7_, C_9_), 31.1, 31.9 (C_6_, C_10_), 40.5, 43.0 (C_8_), 62.8, 63.7 (C_5_), 126.4 (C_4′_), 126.8, 127.0 (C_2′_, C_6′_), 128.5, 128.7 (C_3′_, C_5′_), 145.2, 146.0 (C_1′_), 155.7, 156.1 (C_2_=O), 177.0, 177.5 (C_4_=O); elemental analysis calcd (%) for C_15_H_18_N_2_O_2_: C 69.74, H 7.02, N 10.84, found: C 69.78, H 7.12, N 10.77.

General experimental procedure for the synthesis of benzyl esters (7–15 and 46). Potassium bis(trimethylsilyl)amide (3.06 mmol, 1.02 eq, 95% purity) was added portionwise under ice cooling to a solution of the hydantoin (3 mmol, 1.0 eq) in dry THF (30 mL) under argon atmosphere. The reaction mixture was stirred for 20 min to 1 h at rt, and the resulting emulsion was concentrated to dryness under reduced pressure. The remaining potassium imidate salt was dissolved in dry DMF (30 mL), and benzyl bromoacetate or benzyl 2-bromopropanoate (3.15 mmol, 1.05 eq) was added dropwise. After stirring for 48–64 h at 35–38 °C under argon, the reaction mixture was diluted in ice water (300 mL) and extracted with AcOEt (3 × 150 mL). The combined organic extracts were washed with H_2_O (3 × 200 mL) and brine (2 × 200 mL), dried over anh. Na_2_SO_4,_ and evaporated in vacuo. The crude residue was purified by silica gel chromatography to yield the title benzyl esters (7–15 and 46).

Benzyl (4′-methyl-2,5-dioxo-3′,4′-dihydro-2′*H*-spiro[imidazolidine-4,1′-naphthalene]-1-yl)acetate (7, 8). Hydantoin 4 (1.2 g, 5.21 mmol) was dissolved in 52 mL dry THF, and to this solution, an amount of potassium bis(trimethylsilyl)amide (1.12 g, 5.31 mmol, 95% purity) was added portionwise under ice cooling. After stirring for 1 h at rt under argon, the solvent was removed under reduced pressure, and the remaining potassium imidate salt was dissolved in dry DMF (52 mL). Benzyl bromoacetate (1.25 g, 5.47 mmol) was added dropwise. The stirring was continued at 35–38 °C for 64 h under argon, and the reaction mixture was worked up following the general procedure for the preparation of benzyl esters. The viscous oily residue was chromatographed on silica gel using CH_2_Cl_2_, CH_2_Cl_2_/AcOEt 20:1 και 10:1 as eluents to afford two pairs of enantiomers in a 70/30 ratio. (1.7 g, 86%).

((4*R*,4′*S*)/(4*S*,4′*R*)-(4′-Methyl-2,5-dioxo-3′,4′-dihydro-2′*H*-spiro[imidazolidine-4,1′-naphthalene]-1-yl)acetate (7). White foamy product, which was crystallized upon treatment with *n*-pentane under ice cooling. (1.19 g); *R*_f_ = 0.78 (CH_2_Cl_2_/AcOEt 8:1); mp 111–113 °C (AcOEt/dry Et_2_O-*n*-pentane); ^1^H NMR (400.13 MHz, CDCl_3_): *δ* (ppm) = 1.27, 1.28 (d + d, *J* = 7.1 Hz, *J* = 7.1 Hz, 3H, C*H_3_*), 1.45 (dddd, *J_1_* = 13.2 Hz, *J_2_* = 9.9 Hz, *J_3_* = 7.3 Hz, *J_4_* = 2.9 Hz, 0.55H, H_3′_), 1.84 (dtd, *J_1_* = 11.9 Hz, *J_2_* = 8.6 Hz, *J_3_* = 3.8 Hz, 0.5H, H_2′_), 1.92 (dddd, *J_1_* = 10.8 Hz, *J_2_* = 9.0 Hz, *J_3_* = 5.9 Hz, *J_4_* = 2.7 Hz, 0.9H, H_3′_), 2.00 (ddd, *J_1_* = 13.5 Hz, *J_2_* = 8.6 Hz, *J_3_* = 2.9 Hz, 0.5H, H_2′_), 2.15 (ddd, *J_1_* = 13.0 Hz, *J_2_* = 9.6 Hz, *J_3_* = 3.1 Hz, 0.5H, H_2′_), 2.28 (ddt, *J_1_* = 11.7 Hz, *J_2_* = 5.5 Hz, *J_3_* = 2.4 Hz, 0.5H, H_3′_), 2.33 (ddd, *J_1_* = 13.5 Hz, *J_2_* = 6.4 Hz, *J_3_* = 2.8 Hz, 0.5H, H_2′_), 3.00 (dq, *J_1_* = 12.9 Hz, *J_2_* = 6.3 Hz, 1H, H_4′_), 4.21–4.36 (2q, AB, *J_1AB_* = *J_2AB_* = 17.4 Hz, 2H, NC*H_2_*COO), 5.07–5.17 (q, AB, *J_AΒ_* = 12.2 Hz, 2H, OC*H_2_*Ph), 5.94, 5.96 (s + s, 1H, H_3_), 7.02 (td, *J_1_* = 8.3 Hz, *J_2_* = 2.2 Hz, 1H, H_7′_), 7.12–7.22 (complex m, 3H, H_5′_, H_6′_, H_8′_), 7.23–7.34 (complex m, 5H, H_2″_, H_3″_, H_4″_, H_5″_, H_6″_); ^13^C (50.32 MHz, CDCl_3_): *δ* (ppm) = 22.4 (*C*H_3_), 26.6, 27.2 (C_3′_), 31.2, 31.7 (C_2′_), 32.0, 32.1 (C_4′_), 39.7 (N*C*H_2_COO), 63.7, 63.8 (C_4_), 67.9 (O*C*H_2_Ph), 127.1 (C_7′_), 127.3 (C_8′_), 128.6, 128.8, 129.0, 129.1 (C_5′_, C_6′_, C_2″_, C_3″_, C_4″_, C_5″_, C_6″_), 132.3 (C_8′a_), 135.0 (C_1″_), 143.0, 143.1 (C_4′a_), 155.7 (C_2_=O), 167.1 (*C*OOCH_2_Ph), 175.7 (C_5_=O); elemental analysis calcd (%) for C_22_H_22_N_2_O_4_: C 69.83, H 5.86, N 7.40, found: C 69.79, H 5.89, N 7.49.

((4*R*,4′*R*)/(4*S*,4′*S*)-4′-Methyl-2,5-dioxo-3′,4′-dihydro-2′*H*-spiro[imidazolidine-4,1′-naphthalene]-1-yl)acetate (8). White foamy product. (510 mg); *R*_f_ = 0.70 (CH_2_Cl_2_/AcOEt 8:1); ^1^HNMR (400.13 MHz, CDCl_3_): *δ* (ppm) = 1.33, 1.34 (dd, *J* = 6.9 Hz, *J* = 7.0 Hz, 3H, C*H_3_*), 1.55 (ddt, *J_1_* = 13.9 Hz, *J_2_* = 10.1 Hz, *J_3_* = 4.9 Hz, 0.6H, H_3′_), 1.88 (ddd, *J_1_* = 13.4 Hz, *J_2_* = 7.5 Hz, *J_3_* = 3.6 Hz, 0.5H, H_2′_), 1.91–1.99 (complex m, 0.9H, H_3′_), 2.05 (ddd, *J_1_* = 13.5 Hz, *J_2_* = 8.6 Hz, *J_3_* = 2.5 Hz, 0.45H, H_2′_), 2.18 (ddd, *J_1_* = 12.7 Hz, *J_2_* = 9.7 Hz, *J_3_* = 2.8 Hz, 0.5H, H_2′_), 2.32 (ddd, *J_1_* = 14.5 Hz, *J_2_* = 7.1 Hz, *J_3_* = 2.8 Hz, 0.5H, H_3′_), 2.36 (ddd, *J_1_* = 13.2 Hz, *J_2_* = 8.8 Hz, *J_3_* = 4.4 Hz, 0.5H, H_2′_), 2.97 (dq, *J_1_* = 12.0 Hz, *J_2_* = 6.3 Hz, 1H, H_4′_), 4.23–4.40 (2q, AB, *J_1AB_* = *J_2AB_* = 17.4 Hz, 2H, NC*H_2_*CO), 5.11–5.24 (q, AB, *J_AB_* = 12.2 Hz, 2H, OC*H_2_*Ph), 6.84, 6.89 (s + s, 1H, H_3_), 7.07 (t, *J* = 7.2 Hz, 1H, H_7′_), 7.14–7.28 (complex m, 3H, H_5′_, H_6′_, H_8′_), 7.30–7.46 (complex m, 5H, H_2″_, H_3″_, H_4″_, H_5″_, H_6″_); ^13^C (50.32 MHz, CDCl_3_): *δ* (ppm) = 22.1 (*C*H_3_), 26.2, 26.8 (C_3′_), 30.7, 31.2 (C_2′_), 31.7, 31.9 (C_4′_), 39.3 (N*C*H_2_CO), 63.4, 63.5 (C_4_), 67.5 (O*C*H_2_Ph), 126.7 (C_7′_), 127.0 (C_8′_), 128.3, 128.4, 128.5 (C_5′_, C_6′_, C_2″_, C_3″_, C_4″_, C_5″_, C_6″_), 132.2 (C_8′a_), 134.8 (C_1″_), 142.76, 142.84 (C_4′a_), 155.8 (C_2_=O), 167.0 (*C*OOCH_2_Ph), 175.7 (C_5_=O). HRMS/ESI^+^: *m*/*z* calcd for C_22_H_22_N_2_O_4_: 378.1580; found: 378.1586.

Benzyl 2-(8-phenyl-2,4-dioxo-1,3-diazaspiro [4.5] decan-3-yl)acetate (9). Potassium bis(trimethylsilyl)amide (527 mg, 2.51 mmol, 95% purity) was added portionwise, under ice cooling, to a solution of hydantoin 5 (600 mg, 2.46 mmol) in 30 mL dry THF. The mixture was stirred at r.t. for 20 min under argon. After removal of the solvent under reduced pressure, the residue was dissolved in 30 mL dry DMF, and benzyl bromoacetate (591 mg, 2.58 mmol) was added dropwise. The stirring was continued at 35–38 °C for 48 h under argon, and afterward, the reaction mixture was poured into 200 mL of an ice-water mixture and extracted with AcOEt (3 × 150 mL). The combined organic phases were washed with H_2_O (3 × 150 mL) and brine (2 × 150 mL), dried over anh. Na_2_SO_4,_ and evaporated under reduced pressure affording a white crude solid product, which was purified by silica gel column chromatography using CH_2_Cl_2_ to CH_2_Cl_2_/AcOEt (9:1) as eluents. The column chromatography afforded 880 mg of **9** as a white crystalline solid (91%). Mp 199–201 °C (AcOEt/*n*-pentane), R*_f_* = 0.59 (CH_2_Cl_2_/AcOEt 8:1). ^1^H NMR (400.13 MHz, CDCl_3_) *δ* (ppm) 1.75 (qd, 2H, *J_1_* = 13.6 Hz, *J_2_* = 3.2 Hz, H_7e_, H_9a_), 1.83 (d, 1.7H, *J* = 12.8 Hz, H_6a_, H_10a_, *cis*), 1.94 (dd, 1.75H, *J_1_* = 14.0 Hz, *J_2_* = 3.0 Hz, H_7a_, H_9e_*, cis*), 2.05 (td, 1.85H, *J_1_* = 13.7 Hz, *J_2_* = 3.9 Hz, H_6e_, H_10e_), 2.11 (d, 0.35H, *J* = 13.7 Hz, H_6a_, H_10a_*, trans*), 2.31 (qd, 0.35H, *J_1_* = 13.2 Hz, *J_2_* = 3.0 Hz, H_7a_, H_9e_, *trans*), 2.58 (tt, 0.18H, *J_1_* = 12.0 Hz, *J_2_* = 3.7 Hz, H_8_, *trans*), 2.63 (tt, 0.9H, *J_1_* = 12.3 Hz, *J_2_* = 3.6 Hz, H_8_, *cis*), 4.33 (s, 0.34H, NC*H_2_*COO, *trans*), 4.38 (s, 1.7H, NC*H_2_*COO, *cis*), 5.15 (s, 1.7H, COOC*H_2_*Ph, *cis*), 5.16 (s, 0.34H, COOC*H_2_*Ph, *trans*), 6.68 (s, 0.16H, H_1_, *trans*), 7.19 (tt, 0.88H, *J_1_* = 7.1 Hz, *J_2_* = 1.3 Hz, H_4′_, *cis*), 7.22 (tt, 0.18H, *J_1_* = 7.1 Hz, *J_2_* = 1.5 Hz, H_4′_, *trans*), 7.28 (d, 1.8H, *J* = 7.7 Hz, H_3′_, H_5′_), 7.30–7.39 (m, 7H, H_2′_, H_6′_, H_2″_, H_3″_, H_4″_, H_5″_, H_6″_), 8.64 (s, 0.8H, H_1_, *cis*); ^13^C NMR (150.9 MHz, CDCl_3_) *δ* (ppm) 29.2 (C_7_, C_9_), 33.5 (C_6_, C_10_, *cis*), 35.0 (C_6_, C_10_, *trans*), 39.2 (N*C*H_2_COO, *trans*), 39.5 (N*C*H_2_COO, *cis*), 42.6 (C_8_, *trans*), 42.9 (C_8_, *cis*), 60.4 (C_5_, *trans*), 62.5 (C_5_, *cis*), 67.8 (COO*C*H_2_Ph), 126.5 (C_4′_, *trans*), 126.6 (C_4′_, *cis*), 127.0 (C_2′_, C_6′_, *cis*), 127.1 (C_2′_, C_6′_, *trans*), 128.5 (C_2″_, C_6″_), 128.61 (C_3′_, C_5′_, *trans*), 128.65 (C_3′_, C_5′_, *cis*), 128.7 (C_4″_), 128.8 (C_3″_, C_5″_), 135.0 (C_1″_), 146.0 (C_1′_, *trans*), 146.1 (C_1′_, *cis*), 156.1 (C_2_=O, *trans*), 157.0 (C_2_=O, *cis*), 167.1 (*C*OOCH_2_Ph), 175.8 (C_4_=O, *trans*), 176.7 (C_4_=O, *cis*). Anal. Calcd for C_23_H_24_N_2_O_4_: C, 70.39; H, 6.16; N, 7.14. Found: C, 70.35; H, 6.22; N, 7.16.

Benzyl 2-(2,4-dioxo-6-phenyl-1,3-diazaspiro [4.5]decan-3-yl) acetate (10). Using the general experimental procedure described for the preparation of benzyl esters, a solution of hydantoin 6 (1000 mg, 4.09 mmol) in 41 mL dry THF was treated with potassium bis(trimethylsilyl)amide (877 mg, 4.17 mmol, 95% purity), which was added portionwise under ice cooling. The stirring was continued at r.t. for 20 min under argon, and afterward, the solvent was evaporated under reduced pressure. The resulting potassium imidate salt was dissolved in 41 mL dry DMF, and then benzyl bromoacetate (985 mg, 4.30 mmol) was added dropwise. After being stirred at 35–38 °C for 64 h under argon, the reaction mixture was poured into 400 mL of ice-water mixture and extracted with AcOEt (3 × 200 mL). The organic phases were combined and washed with H_2_O (3 × 270 mL) and brine (2 × 270 mL), dried over anh. Na_2_SO_4,_ and evaporated in vacuo. The residual viscous oil was chromatographed on a silica gel column, using CH_2_Cl_2_ to CH_2_Cl_2_/AcOEt (10:1), to afford the desired compound **10** as a white product (1.140 mg, 71%). Mp 182–184 °C (AcOEt/*n*-pentane), R*_f_* = 0.65 (CH_2_Cl_2_/AcOEt 8:1). ^1^H NMR (600.11 MHz, CDCl_3_) *δ* (ppm): 1.45 (qt, 1H, *J_1_* = 13.8 Hz, *J_2_* = 3.1 Hz, H_9_), 1.49–1.56 (m, 1H, H_8_), 1.79–1.93 (complex m, 5H, H_7_, H_8_, H_9_, H_10_), 2.07 (td, 1H, *J_1_* = 13.5 Hz, *J_2_* = 4.0 Hz, H_10_), 3.09–3.16 (sym m, 1H, H_6_), 3.85–3.95 (q, AB, 2H, *J_AB_* = 17.5 Hz, NC*H_2_*COO), 5.04–5.16 (q, AB, 2H, *J_AB_* = 12.3 Hz, OC*H_2_*Ph), 7.13–7.38 (complex m, 10H, H_2′_, H_3′_, H_4′_, H_5′_, H_6′_, H_2″_, H_3″_, H_4″_, H_5″_, H_6″_), 7.98 (dd, 1H, *J_1_* = 29.4 Hz, *J_2_* = 8.3 Hz, H_1_); ^13^C NMR (150.9 MHz, CDCl_3_) *δ* (ppm): 21.5 (C_9_), 25.6 (C_8_), 27.7 (C_7_), 34.4 (C_10_), 39.1 (N*C*H_2_COO), 48.0 (C_6_), 66.9 (C_5_), 67.4 (O*C*H_2_Ph), 127.5 (C_4″_), 128.2 (C_2″_, C_6″_), 128.3 (C_3″_, C_5″_), 128.6 (C_4′_), 128.7 (C_2′_, C_6′_), 128.7 (C_3′_, C_5′_), 135.2 (C_1″_), 139.1 (C_1′_), 157.0 (C_2_=O), 166.9 (NCH_2_*C*OO), 175.6 (C_4_=O). Anal. Calcd for C_23_H_24_N_2_O_4_: C, 70.39; H, 6.16; N, 7.14. Found: C, 70.30; H, 6.17; N, 7.08.

Benzyl 2-(1-methyl-2,4-dioxo-8-phenyl-1,3-diazaspiro [4.5]decan-3-yl)acetate (11). Sodium hydride (102 mg, 2.56 mmol, 95% purity) was added portionwise to a solution of hydantoin 45 (550 mg, 2.13 mmol) in 21 mL dry DMF under ice cooling. The mixture was stirred for 20 min at rt under argon, and then benzyl bromoacetate (586 mg, 2.56 mmol) was added dropwise. The stirring was continued for 48 h at 35–38 °C under argon, and the mixture was then worked up following the same procedure described for the synthesis of benzyl esters. The crude colorless oily residue was purified by column chromatography with CH_2_Cl_2_, CH_2_Cl_2_/AcOEt 30:1 and 20:1 to yield a colorless oily product, which was crystallized upon treatment with *n*-pentane and Et_2_O at 0 °C to afford the desired **11** as a white crystalline solid. (765 mg, 88%); *R*_f_ = 0.6, 0.37 (CH_2_Cl_2_/AcOEt 10:1); mp 92–94 °C (AcOEt/dry Et_2_O-*n*-pentane); ^1^H NMR (600.11 MHz, CDCl_3_): *δ* (ppm) = 1.79–1.93 (complex m, 6H, H_6_, H_7_, H_9_, H_10_), 2.37 (qd, *J_1_* = 12.2 Hz, *J_2_* = 3.2 Hz, 2H, H_7_, H_9_), 2.52 (tt, *J_1_* = 12.4 Hz, *J_2_* = 3.3 Hz, 1H, H_8_), 2.91 (s, 3H, C*H_3_*), 4.33 (s, 2H, NC*H_2_*COO), 5.18 (s, 2H, OC*H_2_*Ph), 7.22 (tt, *J_1_* = 7.0 Hz, *J_2_* = 1.7 Hz, 1H, H_4′_), 7.27–7.38 (complex m, 9H, H_2′_, H_3′_, H_5′_, H_6′_, H_2″_, H_3″_, H_4″_, H_5″_, H_6″_); ^13^C NMR (150.9 MHz, CDCl_3_): *δ* (ppm) = 24.0 (CH_3_), 29.0 (C_7_, C_9_), 31.1 (C_6_, C_10_), 39.4 (N*C*H_2_COO), 43.1 (C_8_), 62.2 (C_5_), 67.7 (O*C*H_2_Ph), 126.5 (C_4′_), 127.1 (C_2′_, C_6′_), 128.5 (C_3′_, C_5′_), 128.6 (C_2″_, C_6″_), 128.66 (C_4″_), 128.75 (C_3″_, C_5″_), 135.1 (C_1″_), 146.1 (C_1′_), 154.7 (C_2_=O), 167.3 (*C*OOCH_2_Ph), 175.5 (C_4_=O); elemental analysis calcd (%) for C_24_H_26_N_2_O_4_: C 70.92, H 6.45, N 6.89, found: C 70.98, H 6.50, N 6.84.

Benzyl 2-bromopropanoate. A two-necked round-bottom flask equipped with a magnetic stirrer and a dropping funnel was charged with *N*,*N*′-dicyclohexylcarbodiimide (6.19 g, 30.0 mmol) and Et_2_O (75 mL). To this suspension, 2-bromopropionic acid (3.82 g, 25.0 mmol) was added. The dropping funnel was charged with benzyl alcohol (3.24 g, 30 mmol), a catalytic amount of 4-dimethylaminopyridine (183 mg, 1.5 mmol), and Et_2_O (15 mL), and this solution was added dropwise to the suspension. After stirring at ambient temperature for 3 h, the reaction mixture was evaporated to half of its volume under reduced pressure and poured into 130 mL *n*-hexane. The precipitate formed was filtered off through a pad of Celite, and the filtrate was concentrated to dryness. The viscous oily residue was purified by column chromatography on silica gel with *n*-hexane/AcOEt 80:1, 40:1, and 20:1 as eluents to afford 4.83 g (79%) of pure benzyl 2-bromopropanoate as a colorless oil. ^1^H NMR spectrum in CDCl_3_ identical to that reported in the literature.

Benzyl 2-(2,4-dioxo-8-phenyl-1,3-diazaspiro [4.5]decan-3-yl)propanoate (46). To a solution of the hydantoin 5 (750 mg, 3.07 mmol) in dry THF (30 mL), potassium bis(trimethylsilyl)amide (657 mg, 3.13 mmol, 95% purity) was added portionwise at 0 °C. After stirring for 15 min at ambient temperature under argon, the solvent was removed under reduced pressure, and the remaining potassium imidate salt was dissolved in dry DMF (30 mL). Benzyl 2-bromopropanoate (783 mg, 3.22 mmol) was then added dropwise, and the stirring was continued for 62 h at 42 °C under argon. The reaction mixture was worked up following the general procedure described for the synthesis of benzyl esters. The resulting yellowish oil was chromatographed on silica gel using *n*-hexane/AcOEt 10:1, 6:1, and 4:1 as eluents to afford the title compound **46** as a white crystalline solid. (985 mg, 79%); *R*_f_ = 0.21 (*n*-hexane/AcOEt 4:1); mp 177–179 °C (AcOEt/*n*-pentane); ^1^H NMR (600.11 MHz, CDCl_3_): *δ* (ppm) = 1.66–1.84 (complex m, 4H, H_6_, H_7_, H_9_, H_10_), 1.76 (d, *J* = 7.4 Hz, 3H, NCH(C*H_3_*)COO), 1.87–1.93 (complex m, 1.9H, H_7_, H_9_), 1.99 (td, *J_1_* = 13.8 Hz, *J_1_* = 4.1 Hz, 2H, H_6_, H_10_), 2.27 (dtdd, *J_1_* = 16.3 Hz, *J_2_* = 13.1 Hz, *J_3_* = 8.1 Hz, *J_4_* = 3.3 Hz, 0.2H, H_7_, H_9_), 2.55 (tt, *J_1_* = 12.1 Hz, *J_2_* = 3.5 Hz, 0.1H, H_8_), 2.60 (tt, *J_1_* = 12.2 Hz, *J_2_* = 3.5 Hz, 0.9H, H_8_), 4.82 (q, *J* = 7.3 Hz, 0.1H, NC*H*(CH_3_)COO), 4.88 (q, *J* = 7.3 Hz, 0.8H, NC*H*(CH_3_)COO), 5.02–5.23 (q, AX, *J_AX_* = 12.3 Hz, 1.8H, OC*H_2_*Ph), 5.04–5.27 (q, AX, *J_AX_* = 12.3 Hz, 0.2H, OC*H_2_*Ph), 6.42 (s, 0.1H, H_1_), 7.22 (td, *J_1_* = 7.0 Hz, *J_2_* = 1.6 Hz, 1H, H_4′_), 7.25–7.37 (complex m, 9H, H_2′_, H_3′_, H_5′_, H_6′_, H_2″_, H_3″_, H_4″_, H_5″_, H_6″_), 8.67 (s, 0.9H, H_1_); ^13^C NMR (50.32 MHz, CDCl_3_): *δ* (ppm) = 14.9 (NCH(*C*H_3_)COO), 29.2 (C_7_, C_9_), 33.4 (C_6_, C_10_), 43.1 (C_8_), 48.2 (N*C*H(CH_3_)COO), 62.0 (C_5_), 67.7 (O*C*H_2_Ph), 126.6 (C_4′_), 127.0 (C_2′_, C_6′_), 128.4 (C_2″_, C_6″_), 128.5 (C_4″_), 128.6 (C_3′_, C_5′_), 128.7 (C_3″_, C_5″_), 135.3 (C_1″_), 146.2 (C_1′_), 157.3 (C_2_=O), 169.5 (*C*OOCH_2_Ph), 176.7 (C_4_=O); elemental analysis calcd (%) for C_24_H_26_N_2_O_4_: C 70.92, H 6.45, N 6.89, found: C 71.00, H 6.49, N 6.82.

General *N*-alkylation procedure for the preparation of analogs (12–15 and 50). To a well-stirred and ice-cooled solution of the benzyl ester (3.25 mmol, 1.0 eq) in dry DMF (15 mL), NaH (3.9 mmol, 1.2 eq, 60% dispersion in mineral oil) was slowly added in small portions. After 15 min of stirring at ambient temperature under argon, the mixture was treated dropwise with methyl iodide/ethyl iodide/benzyl bromide (3.9 mmol, 1.2 eq), and it was allowed to react for 7 days at 60–65 °C under argon. The reaction mixture was poured into ice water (150 mL) with AcOEt (3 × 120 mL). The combined organic extracts were washed with H_2_O (3 × 180 mL) and brine (2 × 180 mL) and dried over anh. Na_2_SO_4,_ and the solvent were concentrated under reduced pressure to afford a viscous oily residue. The crude product was chromatographed on silica gel to yield the respective *N*-alkylated products.

Benzyl 2-(1-methyl-2,4-dioxo-6-phenyl-1,3-diazaspiro [4.5]decan-3-yl)acetate (12). A stirred solution of **10** (500 mg, 1.27 mmol) in dry DMF (5.9 mL) was treated with sodium hydride (61 mg, 1.53 mmol, 60% dispersion in mineral oil), which was added portionwise under ice cooling. After stirring at r.t. for 15 min under argon, methyl iodide (217 mg, 1.53 mmol) was added dropwise. The reaction mixture was stirred at 60 °C for 7 days under argon, and then it was poured into an ice-water mixture (60 mL) and extracted with AcOEt (3 × 45 mL). The combined organic phases were washed with H_2_O (3 × 70 mL) and brine (2 × 70 mL), dried over anh. Na_2_SO_4,_ and evaporated under reduced pressure. The yellowish crude oily product was purified by silica gel column chromatography, using CH_2_Cl_2_/AcOEt 15:1 as eluent to afford the target compound **12** (280 mg, 54%) as a colorless highly viscous oil, which, after being left under vacuum, was converted to semisolid. Low mp, R*_f_* = 0.85 (CH_2_Cl_2_/AcOEt 8:1). ^1^H NMR (600.11 MHz, CDCl_3_) *δ* (ppm): 1.65 (qt, 1H, *J_1_* = 13.2 Hz, *J_2_* = 3.7 Hz, H_8_), 1.83 (qt, 1H, *J_1_* = 14.0 Hz, *J_2_* = 4.1 Hz, H_9_), 1.92 (dq, 2H, *J_1_* = 14.3 Hz, *J_2_* = 4.1 Hz, H_7_, H_9_), 1.97 (dp, 1H, *J_1_* = 14.5 Hz, *J_2_* = 1.7 Hz, H_10_), 2.05–2.09 (m, 1H, H_8_), 2.13 (ddd, 1H, *J_1_* = 13.8 Hz, *J_2_* = 12.8 Hz, *J_3_* = 3.6 Hz, H_7_), 2.22 (td, 1H, *J_1_* = 14.4 Hz, *J_2_* = 5.4 Hz, H_10_), 3.23 (dd, 1H, *J_1_* = 13.9 Hz, *J_2_* = 4.1 Hz, H_6_), 3.34 (s, 3H, NC*H_3_*), 3.86–3.98 (q, AB, 2H, *J_AB_* = 17.4 Hz, NC*H_2_*COO), 5.05–5.13 (q, AB, 2H, *J_AB_* = 12.3 Hz, OC*H_2_*Ph), 7.06–7.11 (m, 2H, H_2′_, H_6′_), 7.17–7.20 (m, 1H, H_4′_), 7.21–7.24 (m, 2H, H_3′_, H_5′_), 7.26–7.29 (m, 2H, H_2″_, H_6″_), 7.30–7.37 (complex m, 3H, H_3″_, H_4″_, H_5″_); ^13^C NMR (150.9 MHz, CDCl_3_) *δ* (ppm): 22.0 (C_9_), 25.4 (C_8_), 27.8 (C_7_), 30.5 (N*C*H_3_), 32.9 (C_10_), 39.7 (N*C*H_2_COO), 49.4 (C_6_), 67.5 (O*C*H_2_Ph), 67.8 (C_5_), 127.6 (C_4′_), 128.1 (C_2′_, C_6′_), 128.4 (C_2″_, C_6″_), 128.5 (C_3′_, C_5′_), 128.6 (C_4″_), 128.7 (C_3″_, C_5″_), 135.2 (C_1″_), 139.2 (C_1′_), 155.3 (C_2_=O), 166.9 (NCH_2_*C*OO), 175.5 (C_4_=O). Anal. Calcd for C_24_H_26_N_2_O_4_: C, 70.92; H, 6.45; N, 6.89. Found: C, 70.93; H, 6.39; N, 6.86.

Benzyl 2-(1-ethyl-8-phenyl-2,4-dioxo-1,3-diazaspiro [4.5]decan-3-yl)acetate (13). To a stirred solution of **9** (660 mg, 1.68 mmol) in dry DMF (12 mL), sodium hydride (81 mg, 2.02 mmol, 60% dispersion in mineral oil) was added portionwise under ice cooling. After stirring at ambient temperature for 15 min under argon, ethyl iodide (393 mg, 2.52 mmol) was added dropwise. Stirring was continued at 60 °C for 7 days under argon, and the reaction mixture was diluted with 120 mL ice-water mixture and extracted with AcOEt (3 × 80 mL). The combined organic extracts were washed with H_2_O (3 × 100 mL) and brine (2 × 100 mL), dried with anh. Na_2_SO_4,_ and evaporated in vacuo. The yellow viscous oil was purified by column chromatography on silica gel, with CH_2_Cl_2_ to CH_2_Cl_2_/AcOEt 30:1 to afford the desired compound **13** (310 mg, 44%) as a colorless oil, which solidified on cooling. A mixture of CH_2_Cl_2_/AcOEt 20:1 was then used to elute the transesterification product **54** (80 mg, 13%) as a viscous oil, which was crystallized upon treatment with n-pentane. Mp 84–86 °C (AcOEt/n-pentane), R_f_ = 0.87 (CH_2_Cl_2_/AcOEt 8:1). ^1^H NMR (600.11 MHz, CDCl_3_) δ (ppm) 1.25 (t, 3H, J = 7.1 Hz, NCH_2_CH_3_), 1.88–1.95 (m, 2H, H_6a_, H_10a_), 1.98 (ddd, 2H, J_1_ = 13.2 Hz, J_2_ = 9.5 Hz, J_3_ = 3.3 Hz, H_7a_, H_9e_), 2.02 (ddd, 2H, J_1_ = 10.5 Hz, J_2_ = 8.7 Hz, J_3_ = 3.2 Hz, H_6e_, H_10e_), 2.20 (ddd, 2H, J_1_ = 10.2 Hz, J_2_ = 8.4 Hz, J_3_ = 3.7 Hz, H_7e_, H_9e_), 2.87 (ddd, 1H, J_1_ = 13.2 Hz, J_2_ = 9.0 Hz, J_3_ = 4.2 Hz, H_8_), 3.58 (q, 2H, J = 7.1 Hz, NCH_2_CH_3_), 4.31 (s, 2H, NCH_2_COO), 5.17 (s, 2H, COOCH_2_Ph), 7.24 (tt, 1H, J_1_ = 7.3 Hz, J_2_ = 1.1 Hz, H_4′_), 7.28 (d, 2H, J = 7.3 Hz, H_2′_, H_6′_), 7.32–7.39 (m, 7H, H_3′_, H_5′_, H_2″_, H_3″_, H_4″_, H_5″_, H_6″_); ^13^C NMR (150.9 MHz, CDCl_3_) δ (ppm) 15.0 (NCH_2_CH_3_), 28.0 (C_7_, C_9_), 31.9 (C_6_, C_10_), 37.0 (NCH_2_CH_3_), 39.6 (C_8_), 39.8 (NCH_2_COO), 63.4 (C_5_), 67.7 (COOCH_2_Ph), 126.5 (C_4′_), 126.9 (C_2′_, C_6′_), 128.5 (C_2″_, C_6″_), 128.7 (C_4″_), 128.8 (C_3′_, C_5′_, C_3″_, C_5″_), 135.1 (C_1″_), 144.9 (C_1′_), 154.7 (C_2_=O), 167.2 (COOCH_2_Ph), 176.3 (C_4_=O). Anal. Calcd for C_25_H_28_N_2_O_4_: C, 71.41; H, 6.71; N, 6.66. Found: C, 71.45; H, 6.77; N, 6.60.

Ethyl 2-(1-ethyl-8-phenyl-2,4-dioxo-1,3-diazaspiro [4.5]decan-3-yl)acetate (54). Mp 91–93 °C (AcOEt/*n*-pentane), R*_f_* = 0.76 (CH_2_Cl_2_/AcOEt 8:1). ^1^H NMR (600.11 MHz, CDCl_3_) *δ* (ppm) 1.27 (t, 3H, *J* = 7.1 Hz, NCH_2_C*H_3_*), 1.28 (t, 3H, *J* = 7.1 Hz, COOCH_2_C*H_3_*), 1.97 (ddd, 2H, *J_1_* = 16.7 Hz, *J_2_* = 8.9 Hz, *J_3_* = 3.8 Hz, H_6a_, H_10a_), 2.00 (ddd, 2H, *J_1_* = 13.5 Hz, *J_2_* = 9.6 Hz, *J_3_* = 3.6 Hz, H_7a_, H_9e_), 2.04–2.11 (m, 2H, H_7e_, H_9a_), 2.89 (ddd, 1H, *J_1_* = 13.1 Hz, *J_2_* = 9.0 Hz, *J_3_* = 4.2 Hz, H_8_), 3.60 (q, 2H, *J* = 7.1 Hz, NC*H_2_*CH_3_), 4.21 (q, 2H, *J* = 7.1 Hz, COOC*H_2_*CH_3_), 4.25 (s, 2H, NC*H_2_*COO), 7.24 (tt, 1H, *J_1_* = 7.3 Hz, *J_2_* = 1.1 Hz, H_4′_), 7.28 (d, 2H, *J* = 7.3 Hz, H_2′_, H_6′_), 7.35 (tt, 2H, *J_1_* = 7.7 Hz, *J_2_* = 1.1 Hz, H_3′_, H_5′_); ^13^C NMR (150.9 MHz, CDCl_3_) *δ* (ppm) 14.2 (COOCH_2_*C*H_3_), 15.0 (NCH_2_*C*H_3_), 28.0 (C_7_, C_9_), 32.0 (C_6_, C_10_), 37.0 (N*C*H_2_CH_3_), 39.7 (N*C*H_2_COO), 39.8 (C_8_), 61.9 (COO*C*H_2_CH_3_), 63.4 (C_5_), 126.5 (C_4′_), 126.9 (C_2′_, C_6′_), 128.8 (C_3′_, C_5′_), 144.9 (C_1′_), 154.8 (C_2_=O), 167.3 (*C*OOCH_2_Ph), 176.4 (C_4_=O). Anal. Calcd for C_20_H_26_N_2_O_4_: C, 67.02; H, 7.31; N, 7.82. Found: C, 67.00; H, 7.42; N, 7.90.

Benzyl 2-(1-benzyl-2,4-dioxo-8-phenyl-1,3-diazaspiro [4.5]decan-3-yl)acetate (14). To a stirred solution of 9 (800 mg, 2.04 mmol) in 9 mL dry DMF, sodium hydride (98 mg, 2.45 mmol, 60% dispersion in mineral oil) was added portionwise under ice cooling. The mixture was stirred at ambient temperature for 30 min under argon, and then benzyl bromide (419 mg, 2.45 mmol) was added dropwise. After 7 days of stirring at 65 °C under argon, the reaction mixture was worked up according to the general *N*-benzylation procedure described. The crude yellowish oily residue was purified by column chromatography on silica gel with CH_2_Cl_2_ and then CH_2_Cl_2_/AcOEt 20:1 to yield an off-yellow oily product, which was crystallized after treatment with hexane to afford the title compound **14** as a white crystalline solid. (330 mg, 33%); *R*_f_ = 0.80 (CH_2_Cl_2_/AcOEt 8:1); mp 120–122 °C (AcOEt/dry Et_2_O-*n*-pentane); ^1^H NMR (600.11 MHz, CDCl_3_): *δ* (ppm) = 1.72 (dtd, *J_1_* = 13.6 Hz, *J_2_* = 9.9 Hz, *J_3_* = 3.9 Hz, 2H, H_7_, H_9_), 1.87 (ddd, *J_1_* = 14.8 Hz, *J_2_* = 10.3 Hz, *J_3_* = 4.9 Hz, 2H, H_6_, H_10_), 1.91 (ddd, *J_1_* = 14.5 Hz, *J_2_* = 11.3 Hz, *J_3_* = 4.4 Hz, 2H, H_6_, H_10_), 2.02 (dq, *J_1_* = 14.3 Hz, *J_2_* = 5.3, 4.9 Hz, 2H, H_7_, H_9_), 2.77 (tt, *J_1_* = 8.8 Hz, *J_2_* = 4.0 Hz, 1H, H_8_), 4.39 (s, 2H, NC*H_2_*COO), 4.75 (s, 2H, NC*H_2_*Ph), 5.21 (s, 2H, OC*H_2_*Ph), 7.13 (d, *J* = 7.6 Hz, 2H, H_2′_, H_6′_), 7.20 (d, *J* = 7.4 Hz, 2H, H_2Bz_, H_6Bz_), 7.23 (t, *J* = 7.2 Hz, 1H, H_4′_), 7.27 (d, *J* = 7.5 Hz, 2H, H_2″_, H_6″_), 7.28 (complex m, 8H, H_3′_, H_5′_, H_3″_, H_4″_, H_5″_, H_3Bz_, H_4Bz_, H_5Bz_); ^13^C NMR (150.9 MHz, CDCl_3_): *δ* (ppm) = 27.8 (C_7_, C_9_), 31.4 (C_6_, C_10_), 39.3 (C_8_), 40.0 (N*C*H_2_COO), 44.9 (N*C*H_2_Ph), 64.1 (C_5_), 67.8 (O*C*H_2_Ph), 126.4 (C_4′_), 127.0 (C_2Bz_, C_6Bz_), 127.1 (C_2′_, C_6′_), 127.7 (C_4Bz_), 128.6 (C_2″_, C_6″_), 128.67 (C_3′_, C_5′_), 128.73 (C_4″_), 128.8 (C_3″_, C_5″_), 128.9 (C_3Bz_, C_5Bz_), 135.1 (C_1″_), 137.8 (C_1Bz_), 144.8 (C_1′_), 155.9 (C_2_=O), 167.2 (*C*OOCH_2_Ph), 176.3 (C_4_=O); elemental analysis calcd (%) for C_30_H_30_N_2_O_4_: C 74.67, H 6.27, N 5.81, found: C 74.63, H 6.33, N 5.85.

Benzyl 2-(1-benzyl-2,4-dioxo-6-phenyl-1,3-diazaspiro [4.5] decan-3-yl)acetate (15). Prepared by *N*-alkylation of benzyl ester 10 (500 mg, 1.27 mmol) in dry DMF (5.9 mL) by using NaH (61 mg, 1.53 mmol, 60% dispersion in mineral oil) and benzyl bromide (262 mg, 1.53 mmol) as described in the main manuscript for the preparation of *N*-benzylated derivatives. After stirring at 65 °C for 7 days under argon, the reaction mixture was diluted with 60 mL ice water and extracted with AcOEt (3 × 45 mL). The combined organic extracts were washed with H_2_O (3 × 70 mL) and brine (2 × 70 mL), dried with anh. Na_2_SO_4,_ and evaporated in vacuo. The crude oily residue was purified by column chromatography on silica gel using CH_2_Cl_2_ to afford the title compound **15** as a colorless oil, which was further crystallized to a white crystalline solid upon treatment with *n*-pentane/Et_2_O (393 mg, 64%). Mp 98–100 °C (AcOEt/*n*-pentane, Et_2_O), R*_f_* = 0.41 (*n*-hexane/AcOEt 3:1). ^1^H NMR (400.13 MHz, CDCl_3_) *δ* (ppm): 1.29 (qt, 1H, *J_1_* = 14.0 Hz, *J_2_* = 4.2 Hz, H_9_), 1.42–1.58 (complex m, 2H, H_8_, H_9_), 1.77 (dp, 1H, *J_1_* = 14.7 Hz, *J_2_* = 1.9 Hz, H_10_), 1.81–1.96 (m, 2H, H_7_, H_8_), 2.03 (td, 1H, *J_1_* = 14.3 Hz, *J_2_* = 4.7 Hz, H_10_), 2.13 (td, 1H, *J_1_* = 13.3 Hz, *J_2_* = 3.4 Hz, H_7_), 3.15 (dd, 1H, *J_1_* = 13.9 Hz, *J_2_* = 4.2 Hz, H_6_), 3.79–4.01 (q, AB, 2H, *J_AB_* = 17.3 Hz, NC*H_2_*COO), 4.63–5.31 (q, AX, 2H, *J_AX_* = 16.7 Hz, NC*H_2_*Ph), 5.02–5.12 (q, AB, 2H, *J_AB_* = 12.2 Hz, OC*H_2_*Ph), 7.03 (~dd, 2H, *J_1_* = 7.8 Hz, *J_2_* = 1.8 Hz, H_2′_, H_6′_), 7.11–7.31 (complex m, 13H, H_3′_, H_4′_, H_5′_, H_2″_, H_3″_, H_4″_, H_5″_, H_6″_, H_2Bz_, H_3Bz_, H_4Bz_, H_5Bz_, H_6Bz_); ^13^C NMR (50.32 ΜHz, CDCl_3_) *δ* (ppm): 20.9 (C_9_), 25.2 (C_8_), 28.0 (C_7_), 32.2 (C_10_), 39.7 (N*C*H_2_COO), 47.0 (N*C*H_2_Ph), 50.2 (C_6_), 67.6 (O*C*H_2_Ph), 68.8 (C_5_), 126.5 (C_2Bz_, C_6Bz_), 127.4 (C_4Bz_), 127.8 (C_4′_), 128.2 (C_2″_, C_6″_), 128.3 (C_2′_, C_6′_), 128.5 (C_4″_, C_3Bz_, C_5Bz_), 128.7 (C_3″_, C_5″_), 128.8 (C_3′_, C_5′_), 135.1 (C_1″_), 137.4 (C_1Bz_), 138.9 (C_1′_), 155.9 (C_2_=O), 167.0 (NCH_2_*C*OO), 175.5 (C_4_=O). Anal. Calcd for C_30_H_30_N_2_O_4_: C, 74.67; H, 6.27; N, 5.81. Found: C, 74.75; H, 6.30; N, 5.89.

Benzyl 2-(1-methyl-2,4-dioxo-8-phenyl-1,3-diazaspiro [4.5]decan-3-yl)propanoate (50). Potassium bis(trimethylsilyl)amide (664 mg, 3.16 mmol, 95% purity) was added portionwise, under ice cooling, to a solution of methyl hydantoin **4*5*** (800 mg, 3.10 mmol) in 31 mL dry THF. The mixture was stirred for 15 min at rt under argon. After removal of the solvent under vacuum, the residue was dissolved in 31 mL dry DMF, and benzyl 2-bromopropanoate (793 mg, 3.26 mmol) was added dropwise. After stirring for another 70 h at 45 °C under argon, the reaction mixture was worked up following the general procedure previously described. The crude yellow oil was purified by column chromatography on silica gel eluting with *n*-hexane/AcOEt 8:1, 5:1, and 4:1 to yield the desired benzyl ester 50 as a colorless oil. Crystallization upon treatment with *n*-pentane under ice cooling yielded **50** as a white solid. (900 mg, 69%); *R*_f_ = 0.12 (*n*-hexane/AcOEt 4:1); mp 76–78 °C (AcOEt/*n*-pentane); ^1^H NMR (400.13 MHz, CDCl_3_): *δ* (ppm) = 1.60–1.90 (complex m, 6H, H_6_, H_7_, H_9_, H_10_), 1.68 (d, *J =* 7.3 Hz, 3H, NCH(C*H_3_*)COO), 2.32 (qd, *J_1_ =* 13.4 Hz, *J_2_ =* 4.0 Hz, 2H, H_7_, H_9_), 2.48 (tt, *J_1_ =* 12.3 Hz, *J_2_ =* 3.3 Hz, 1H, H_8_), 2.86 (s, 3H, NC*H_3_*), 4.82 (q, *J =* 7.3 Hz, 1H, NC*H*(CH_3_)COO), 5.03–5.30 (q, AX, *J_AX_ =* 12.2 Hz, 2H, OC*H_2_*Ph), 7.22 (tt, *J_1_ =* 7.0 Hz, *J_2_ =* 1.9 Hz, 1H, H_4_), 7.25–7.36 (complex m, 9H, H_2′_, H_3′_, H_5′_, H_6′_, H_2″_, H_3″_, H_4″_, H_5″_, H_6″_); ^13^C NMR (150.9 MHz, CDCl_3_): *δ* (ppm) = 14.9 (NCH(*C*H_3_)COO), 23.9 (N*C*H_3_), 28.86, 28.89 (C_7_, C_9_), 30.9 (C_6_, C_10_), 43.1 (C_8_), 48.1 (N*C*H(CH_3_)COO), 61.4 (C_5_), 67.6 (O*C*H_2_Ph), 126.4 (C_4′_), 127.1 (C_2′_, C_6′_), 128.5 (C_2″_, C_6″_), 128.57, 128.60 (C_3′_, C_5′_, C_3″_, C_4″_, C_5″_), 135.4 (C_1″_), 146.1 (C_1′_), 154.8 (C_2_=O), 169.7 (*C*OOCH_2_Ph), 175.3 (C_4_=O); elemental analysis calcd (%) for C_25_H_28_N_2_O_4_: C 71.41, H 6.71, N 6.66, found: C 71.44, H 6.80, N 6.60.

General experimental procedure for the preparation of carboxylic acids (16–24, 47, and 51). A solution of the benzyl ester (2.0 mmol) was hydrogenated over Pd/C (10 wt.%) catalyst for 3 h at 44–46 °C under 50–55 psi pressure in a mixture of EtOH/AcOEt 3:1 (40 mL). The solution was filtered off in vacuo to remove the catalyst, the filtration pad was washed with portions of hot EtOH (3 × 15 mL), and the combined filtrates were evaporated under reduced pressure to afford the desired carboxylic acid. 

((4*R*,4′*S*)/(4*S*,4′*R*)-4′-Methyl-2,5-dioxo-3′,4′-dihydro-2′*H*-spiro[imidazolidine-4,1′-naphthalene]-1-yl)acetic acid (16). A solution of benzyl ester 7 (1.0 g, 2.64 mmol) in 53 mL abs EtOH/AcOEt (4:1) was hydrogenated in the presence of Pd/C (120 mg) according to the general procedure described for the synthesis of carboxylic acids to yield a white foamy product. Removal of the entrapped solvents upon drying under high vacuum yielded the desired compound **16** as a glass solid, which was crystallized upon treatment with dry Et_2_O under ice cooling (760 mg, almost quantitative yield); *R*_f_ = 0.06 (CH_2_Cl_2_/AcOEt 4:1); mp 200–202 °C (AcOEt/dry Et_2_O-*n*-pentane); ^1^H NMR (400.13 MHz, [D_6_]DMSO): *δ* (ppm) = 1.27, 1.29 (d + d, *J* = 7.1 Hz, *J_2_* = 7.0 Hz, 3H, C*H_3_*), 1.56–1.67 (m, 0.45 H, H_3′_), 1.77–1.90 (m, 1H, H_2′_, H_3′_), 1.94–2.10 (m, 1.45H, H_2′_, H_3′_), 2.12–2.29 (m, 1H, H_2′_, H_3′_), 2.94 (dq, *J_1_* = 13.4 Hz, *J_2_* = 7.9 Hz, 1H, H_4′_), 4.05–4.21 (2q, AB, *J_1AB_* = *J_2AB_* = 17.6 Hz, 2H, NC*H_2_*COOH), 7.18 (t, *J* = 7.6 Hz, 1H, H_7′_), 7.22 (dd, 1H, *J_1_* = 7.8 Hz, *J_2_* = 2.0 Hz, H_8′_), 7.26–7.31 (m, 1H, H_6′_), 7.33 (d, *J* = 7.6 Hz, 1H, H_5′_), 8.96, 8.97 (s + s, 1H, H_3′_), 13.26 (s, 1H, NCH_2_COO*H*); ^13^C (50.32 MHz, [D_6_]DMSO): *δ* (ppm) = 22.0, 22.3 (*C*H_3_), 25.6, 26.4 (C_3′_), 30.1, 31.0 (C_2′_), 31.2, 31.6 (C_4′_), 39.2, 39.3 (N*C*H_2_COOH), 62.5, 62.6 (C_4_), 126.4 (C_7′_), 127.1 (C_8′_), 128.0, 128.3, 128.5 (C_5′_, C_6′_), 133.5, 133.6 (C_8′a_), 142.65, 142.74 (C_4′a_), 155.1 (C_2_=O), 168.9 (NCH_2_*C*OOH), 175.9 (C_5_=O); elemental analysis calcd (%) for C_15_H_16_N_2_O_4_: C 62.49, H 5.59, N 9.72; found: C 62.56, H 5.63, N 9.69.

((4*R*,4′*R*)/(4*S*,4′*S*)-4′-Methyl-2,5-dioxo-3′,4′-dihydro-2′*H*-spiro[imidazolidine-4,1′-naphthalene]-1-yl)acetic acid (17). A total of 10 wt% Pd (52 mg) on charcoal was added to a solution of the benzyl ester 8 (430 mg, 1.14 mmol) in a mixture of EtOH/AcOEt 3:1 (23 mL), and the mixture was hydrogenated following the procedure previously described. The white foamy solid obtained strongly binds the aforementioned solvents. Removal of the entrapped solvents upon drying under high vacuum yielded the target compound **17** as a glass solid, which was crystallized upon treatment with dry Et_2_O under ice cooling (325 mg, almost quantitative yield); *R*_f_ = 0.02 (CH_2_Cl_2_/AcOEt 4:1); mp 154–156 °C (AcOEt/dry Et_2_O-*n*-pentane); ^1^H NMR (400.13 MHz, CDCl_3_) *δ* (ppm): 1.35 (~t, *J* = 6.5 Hz, 3H, C*H_3_*), 1.54 (ddt, *J_1_* = 14.0 Hz, *J_2_* = 9.7 Hz, *J_3_* = 4.8 Hz, 0.6H, H_3′_), 1.89–2.04 (complex m, 1.2H, H_2′_, H_3′_), 2.11 (ddd, *J_1_* = 13.2 Hz, *J_2_* = 8.7 Hz, *J_3_* = 2.7 Hz, 0.6H, H_2′_), 2.24 (ddd, *J_1_* = 12.4 Hz, *J_2_* = 9.7 Hz, *J_3_* = 2.6 Hz, 0.6H, H_2′_), 2.31–2.46 (complex m, 1H, H_2′_, H_3′_), 2.94–3.06 (dt, *J_1_* = 12.9 Hz, *J_2_* = 6.3 Hz, 1H, H_4′_), 4.26–4.42 (2q, AB, *J_1AB_* = 17.6 Hz, *J_2AB_* = 18.0 Hz, 2H, NC*H_2_*COOH), 6.58, 6.60 (s + s, 1H, H_3_), 7.11 (td, *J_1_* = 8.0 Hz, *J_2_* = 2.2 Hz, 1H, H_7′_), 7.21 (d, *J* = 7.7 Hz, 1H, H_8′_), 7.23–7.33 (m, 2H, H_5′_, H_6′_); ^13^C (50.32 MHz, CDCl_3_): *δ* (ppm) = 22.4 (*C*H_3_), 26.6, 27.2 (C_3′_), 31.1, 31.6 (C_2′_), 32.0, 32.1 (C_4′_), 39.4 (N*C*H_2_COOH), 63.9, 64.0 (C_4_), 127.1 (C_7′_), 127.2 (C_8′_), 128.6, 128.9 (C_6′_), 129.2 (C_5′_), 132.0 (C_8′a_), 143.1 (C_4′a_), 156.6 (C_2_=O), 171.2 (NCH_2_*C*OOH), 175.7 (C_5_=O); elemental analysis calcd (%) for C_15_H_16_N_2_O_4_: C 62.49, H 5.59, N 9.72; found: C 62.55, H 5.62, N 9.66. 

2-(2,4-Dioxo-8-phenyl-1,3-diazaspiro [4.5]decan-3-yl)acetic acid (18). A mixture of benzyl ester 9 (400 mg, 1.02 mmol) and 10% Pd on charcoal (48 mg) in EtOH/AcOEt 3:1 (20 mL) was hydrogenated following the general procedure described for the preparation of carboxylic acids to afford the title compound **18** as a white crystalline solid. (308 mg, almost quantitative yield); *R*_f_ = 0.05 (CH_2_Cl_2_/AcOEt 5:1); mp >250 °C (MeOH/dry Et_2_O-*n*-pentane); ^1^H NMR (600.11 MHz, [D_6_]DMSO): *δ* (ppm) = 1.65 (dd, *J_1_* = 11.5 Hz, *J_2_* = 2.4 Hz, 1.7H, H_6a_, H_10a_, *cis*), 1.70–1.82 (complex m, 3.7H, H_7_, H_9_, *cis*), 1.86 (qd, *J_1_* = 14.2 Hz, *J_2_* = 3.7 Hz, 2H, H_6e_, H_10e_), 1.92 (tt, *J_1_* = 14.0 Hz, *J_2_* = 3.3 Hz, 0.3H, H_6a_, H_10a_, *trans*), 2.12 (qd, *J_1_* = 13.6 Hz, *J_2_* = 3.7 Hz, 0.3H, H_7_, H_9_, *trans*), 2.59 (tt, *J_1_* = 11.4 Hz, *J_2_* = 4.2 Hz, 1H, H_8_), 4.02 (s, 0.35H, NC*H_2_*COOH, *trans*), 4.06 (s, 1.65H, NC*H_2_*COOH, *cis*), 7.18 (tt, *J_1_* = 7.3 Hz, *J_2_* = 1.5 Hz, 1H, H_4′_), 7.26 (dd, *J_1_* = 8.2 Hz, *J_2_* = 1.6 Hz, 0.3H, H_2′_, H_6′_, *trans*), 7.29 (t, *J* = 7.6 Hz, 2H, H_3′_, H_5′_), 7.33 (dd, *J_1_* = 8.3 Hz, *J_2_* = 1.6 Hz, 1.7H, H_2′_, H_6′_, *cis*), 8.31 (s, 0.15H, H_1_, *trans*), 9.07 (s, 0.8H, H_1_, *cis*), 13.08 (v br s, 1H, NCH_2_COO*H*); ^13^C NMR (100.61 MHz, [D_6_]DMSO): *δ* (ppm) = 28.4 (C_7_, C_9_, *cis*), 28.7 (C_7_, C_9_, *trans*), 33.4 (C_6_, C_10_, *cis*), 33.9 (C_6_, C_10_, *trans*), 38.7 (N*C*H_2_COOH, *trans*), 39.0 (N*C*H_2_COOH, *cis*), 41.3 (C_8_, *trans*), 42.2 (C_8_, *cis*), 59.0 (C_5_, *trans*), 61.1 (C_5_, *cis*), 126.0 (C_4′_), 126.7 (C_2′_, C_6′_, *trans*), 126.9 (C_2′_, C_6′_, *cis*), 128.2 (C_3′_, C_5′_, *cis*),128.4 (C_3′_, C_5′_, *trans*), 146.1 (C_1′_, *trans*), 146.6 (C_1′_, *cis*), 154.8 (C_2_=O, *trans*), 155.2 (C_2_=O, *cis*), 168.8 (NCH_2_*C*OOH, *cis*), 168.9 (NCH_2_*C*OOH, *trans*), 176.1 (C_4_=O, *trans*), 176.5 (C_4_=O, *cis*); elemental analysis calcd (%) for C_16_H_18_N_2_O_4_: C 63.56, H 6.00, N 9.27, found: C 63.64, H 6.07, N 9.20.

2-(2,4-Dioxo-6-phenyl-1,3-diazaspiro [4.5]decan-3-yl)acetic acid (19). Following the general hydrogenolysis procedure for the preparation of carboxylic acids described in the main manuscript, benzyl ester 10 (400 mg, 1.02 mmol) in a mixture of 20 mL EtOH/AcOEt (3:1) provided the target compound **19** as a white crystalline solid (300 mg, almost quantitative yield). Mp > 250 °C (AcOEt/*n*-pentane), R*_f_* = 0.14 (AcOEt). ^1^H NMR (600.11 MHz, DMSO-*d_6_*) *δ* (ppm): 1.46 (qt, 1H, *J_1_* = 13.0 Hz, *J_2_* = 3.6 Hz, H_8_), 1.56 (tt, 1H, *J_1_* = 13.4 Hz, *J_2_* = 3.5 Hz, H_9_), 1.59–1.69 (m, 2H, H_7_, H_10_), 1.72 (dt, 1H, *J_1_* = 13.4 Hz, *J_2_* = 3.6 Hz, H_9_), 1.80 (dt, 1H, *J_1_* = 13.0 Hz, *J_2_* = 3.6 Hz, H_8_), 1.88 (td, 1H, *J_1_* = 13.5 Hz, *J_2_* = 4.0 Hz, H_10_), 2.01 (qd, 1H, *J_1_* = 13.2 Hz, *J_2_* = 3.6 Hz, H_7_), 2.98 (dd, 1H, *J_1_* = 13.4 Hz, *J_2_* = 3.6 Hz, H_6_), 3.50–3.66 (q, AB, 2H, *J_AB_* = 17.4 Hz, NC*H_2_*COOH), 7.08–7.12 (m, 2H, H_3′_, H_5′_), 7.17 (~tt, 1H, *J_1_* = 7.2 Hz, *J_2_*≈2.0 Hz, H_4′_), 7.19–7.24 (m, 2H, H_2′_, H_6′_), 8.98 (s, 1H, H_1_), 12.90 (brs, 1H, NCH_2_COO*H*); ^13^C NMR (150.9 MHz, DMSO-*d_6_*) *δ* (ppm): 20.5 (C_9_), 25.1 (C_8_), 27.0 (C_7_), 34.6 (C_10_), 38.4 (N*C*H_2_COOH), 47.2 (C_6_), 65.7 (C_5_), 126.9 (C_4′_), 127.7 (C_2′_, C_6′_), 128.3 (C_3′_, C_5′_), 139.5 (C_1′_), 155.1 (C_2_=O), 168.4 (NCH_2_*C*OOH), 175.2 (C_4_=O). Anal. Calcd for C_16_H_18_N_2_O_4_: C, 63.56; H, 6.00; N, 9.27. Found: C, 63.61; H, 6.03; N, 9.26.

2-(1-Methyl-2,4-dioxo-8-phenyl-1,3-diazaspiro [4.5]decan-3-yl)acetic acid (20). Following the general hydrogenolysis procedure for the synthesis of carboxylic acids previously described, benzyl ester 11 (630 mg, 1.55 mmol) in a mixture of 31 mL EtOH/AcOEt (3:1) in the presence of Pd/C (76 mg) yielded the target compound **20** as a white crystalline solid (490 mg, almost quantitative yield); *R*_f_ = 0.03 (CH_2_Cl_2_/AcOEt 4:1); mp 234–236 °C (MeOH/*n*-pentane); ^1^H NMR (400.13 MHz, [D_6_]DMSO): *δ* (ppm) = 1.71 (d, *J* = 12.9 Hz, 2H, H_6_, H_10_), 1.76 (d, *J* = 12.4 Hz, 2H, H_7_, H_9_), 2.02 (td, *J_1_* = 13.2 Hz, *J_2_* = 3.5 Hz, 2H, H_6_, H_10_), 2.16 (qd, *J_1_* = 12.6 Hz, *J_2_* = 3.1 Hz, 2H, H_7_, H_9_), 2.62 (tt, *J_1_* = 12.3 Hz, *J_2_* = 3.6 Hz, 1H, H_8_), 2.83 (s, 3H, C*H_3_*), 4.08 (s, 2H, NC*H_2_*COOH), 7.20 (t, *J* = 7.1 Hz, 1H, H_4′_), 7.25 (d, *J* = 7.3 Hz, 2H, H_2′_, H_6′_), 7.31 (t, *J* = 7.4 Hz, 2H, H_3′_, H_5′_), 13.14 (br s, 1H, NCH_2_COO*H*); ^13^C NMR (150.9 MHz, [D_6_]DMSO): *δ* (ppm) = 23.6 (*C*H_3_), 28.6 (C_7_, C_9_), 30.0 (C_6_, C_10_), 39.0 (N*C*H_2_COOH), 41.4 (C_8_), 61.3 (C_5_), 126.1 (C_4′_), 126.6 (C_2′_, C_6′_), 128.4 (C_3′_, C_5′_), 146.3 (C_1′_), 154.2 (C_2_=O), 168.8 (NCH_2_*C*OOH), 175.2 (C_4_=O); elemental analysis calcd (%) for C_17_H_20_N_2_O_4_: C 64.54, H 6.37, N 8.86, found: C 64.50, H 6.45, N 8.88.

2-(1-Methyl-2,4-dioxo-6-phenyl-1,3-diazaspiro [4.5]decan-3-yl)acetic acid (21). It was prepared by hydrogenolysis of benzyl ester 12 (250 mg, 0.62 mmol) in a mixture of EtOH/AcOEt 3:1 (12.5 mL) following the general procedure previously described. Evaporation of the solvents yielded the corresponding carboxylic acid **21** as a shiny white crystalline solid (190 mg, almost quantitative yield). Mp 184–186 °C (AcOEt/*n*-pentane, Et_2_O), R*_f_* = 0.14 (AcOEt). ^1^H NMR (600.11 MHz, DMSO-*d_6_*) *δ* (ppm): 1.52–1.61 (sym m, 1H, H_8_), 1.74 (dq, 1H, *J_1_ =* 13.7 Hz, *J_2_ =* 3.7 Hz, H_7_), 1.79–1.86 (m, 2H, H_9_), 1.89–1.95 (m, 1H, H_10_), 1.95–2.01 (m, 1H, H_8_), 2.02–2.09 (m, 1H, H_10_), 2.20 (qd, 1H, *J_1_ =* 13.6 Hz, *J_2_ =* 3.6 Hz, H_7_), 3.11 (dd, 1H, *J_1_ =* 13.9 Hz, *J_2_ =* 4.1 Hz, H_6_), 3.23 (s, 3H, NC*H_3_*), 3.59–3.77 (q, AB, 2H, *J_AB_ =* 17.3 Hz, NC*H_2_*COOH), 7.05–7.09 (m, 2H, H_2′_, H_6′_), 7.19 (~tt, 1H, *J_1_ =* 7.3 Hz, *J_2_* ≈ 1.7 Hz, H_4′_), 7.21–7.26 (m, 2H, H_3′_, H_5′_), 13.05 (vbs, 1H, NCH_2_COO*H*); ^13^C NMR (150.9 MHz, DMSO-*d_6_*) *δ* (ppm): 21.2 (C_9_), 24.9 (C_8_), 27.0 (C_7_), 29.7 (N*C*H_3_), 32.5 (C_10_), 39.8 (N*C*H_2_COOH), 48.5 (C_6_), 66.8 (C_5_), 127.1 (C_4′_), 127.9 (C_2′_, C_6′_), 128.0 (C_3′_, C_5′_), 139.3 (C_1′_), 154.7 (C_2_=O), 168.2 (NCH_2_*C*OOH), 174.9 (C_4_=O). Anal. Calcd for C_17_H_20_N_2_O_4_: C, 64.54; H, 6.37; N, 8.86. Found: C, 64.50; H, 6.31; N, 8.89.

2-(1-Ethyl-2,4-dioxo-8-phenyl-1,3-diazaspiro [4.5]decan-3-yl)acetic acid (22). A mixture of benzyl ester 13 (480 mg, 1.14 mmol) and 10% Pd on charcoal (58 mg) in EtOH/AcOEt 3:1 (22.8 mL) was subjected to catalytic hydrogenolysis following the general procedure previously described to yield the title compound **22** as a glass solid. Crystallization upon treatment with *n*-pentane (0 °C) yielded 22 as a white crystalline solid (375 mg, almost quantitative yield); *R*_f_ = 0.07 (AcOEt); mp 153–155 °C (AcOEt/dry Et_2_O-*n*-pentane); ^1^H NMR (600.11 MHz, [D_6_]DMSO): *δ* (ppm) = 1.16 (t, *J* = 7.1 Hz, 3H, NCH_2_C*H_3_*), 1.75 (dq, *J_1_* = 7.8 Hz, *J_2_* = 3.8 Hz, 0.4H, H_6_, H_10_, *trans*), 1.84–1.98 (m, 5.6H, H_6_, H_10_, *cis*, H_7_, H_9_), 2.04 (dd, *J_1_* = 8.4 Hz, *J_2_* = 6.0 Hz, 1.8H, H_7_, H_9_, *cis*), 2.18 (qd, *J_1_* = 13.3 Hz, *J_2_* = 4.0 Hz, 0.2H, H_7_, H_9_, *trans*), 2.65 (tt, *J_1_* = 12.6 Hz, *J_2_* = 3.6 Hz, 0.1H, H_8_, *trans*), 2.81 (dt, *J_1_* = 9.4 Hz, *J_2_* = 4.9 Hz, 0.9H, H_8_, *cis*), 3.54 (q, *J* = 7.0 Hz, 2H, NC*H_2_*CH_3_), 4.07 (s, 0.2H, NC*H_2_*COOH, *trans*), 4.08 (s, 1.8H, NC*H_2_*COOH, *cis*), 7.19 (tt, *J_1_* = 7.2 Hz, *J_2_* = 1.6 Hz, 0.1H, H_4′_, *trans*), 7.21 (tt, *J_1_* = 6.4 Hz, *J_2_* = 2.1 Hz, 0.9H, H_4′_, *cis*), 7.24 (dd, *J_1_* = 8.5 Hz, *J_2_* = 1.6 Hz, 0.2H, H_2′_, H_6′_, *trans*), 7.31 (td, *J_1_* = 7.5 Hz, *J_2_* = 1.9 Hz, 0.2H, H_3′_, H_5′_, *trans*), 7.21–7.38 (m, 3.8H, H_2′_, H_6′_, *cis*, H_3′_, H_5′_, *cis*), 13.15 (br s, 1H, NCH_2_COO*H*); ^13^C NMR (150.9 MHz, [D_6_]DMSO): *δ* (ppm) = 14.5 (NCH_2_*C*H_3_), 27.6 (C_7_, C_9_, *cis*), 28.6 (C_7_, C_9_, *trans*), 31.0 (C_6_, C_10_, *trans*), 31.7 (C_6_, C_10_, *cis*), 36.4 (N*C*H_2_CH_3_), 39.1 (N*C*H_2_COOH), 39.4 (C_8_), 62.3 (C_5_), 120.0 (C_4′_), 126.5 (C_2′_, C_6′_, *trans*), 126.8 (C_2′_, C_6′_, *cis*), 128.4 (C_3′_, C_5′_), 145.2 (C_1′_), 154.1 (C_2_=O), 168.6 (NCH_2_*C*OOH), 175.7 (C_4_=O); elemental analysis calcd (%) for C_18_H_22_N_2_O_4_: C 65.44, H 6.71, N 8.48, found: C 65.52, H 6.69, N 8.55.

2-(1-Benzyl-2,4-dioxo-8-phenyl-1,3-diazaspiro [4.5]decan-3-yl)acetic acid (23). It was prepared from benzyl ester 14 (280 mg, 0.58 mmol) by catalytic hydrogenolysis (H_2_/10% Pd-C, 34 mg) in a mixture of EtOH/AcOEt 3:1 (12 mL) following the general procedure previously described. The glass solid formed was crystallized upon treatment with *n*-pentane and some drops of Et_2_O under ice cooling to yield the carboxylic acid **23** as a white crystalline solid. (225 mg, almost quantitative yield); *R*_f_ = 0.10 (AcOEt); mp 129–131 °C (AcOEt/*n*-pentane); ^1^H NMR (600.11 MHz, [D_6_]DMSO): *δ* (ppm) = 1.62 (qd, *J_1_* = 10.5 Hz, *J_2_* = 5.6 Hz, 1.7H, H_7_, H_9_), 1.70 (d, *J* = 10.9 Hz, 0.8H, H_6_, H_10_), 1.75–1.91 (complex m, 4.7H, H_6_, H_7_, H_9_, H_10_), 1.94 (td, *J_1_* = 13.2 Hz, *J_2_* = 3.6 Hz, 0.4H, H_6_, H_10_), 2.14 (qd, *J_1_* = 12.4 Hz, *J_2_* = 2.6 Hz, 0.4H, H_7_, H_9_), 2.57 (t, *J* = 12.5 Hz, 0.2H, H_8_, *cis*), 2.67 (tt, *J_1_* = 10.3, *J_2_* = 3.8 Hz, 0.8H, H_8_, *trans*), 4.14 (s, 0.4H, NC*H_2_*COOH, *cis*), 4.17 (s, 1.6H, NC*H_2_*COOH, *trans*), 4.59 (s, 0.35H, NC*H_2_*Ph, *cis*), 4.81 (s, 1.6H, NC*H_2_*Ph, *trans*), 7.14–7.19 (m, 2H, H_2′_, H_6′_), 7.21 (td, *J_1_* = 7.3 Hz, *J_2_* = 1.3 Hz, 1H, H_4′_), 7.26 (tt, *J_1_* = 7.2 Hz, *J_2_* = 1.6 Hz, 1H, H_4Bz_), 7.27–7.31 (m, 4H, H_2Bz_, H_3Bz_, H_5Bz_, H_6Bz_ 7.31–7.36 (m, 2H, H_3′_, H_5′_), 13.21 (v br s, 1H, NCH_2_COO*H*); ^13^C NMR (150.9 MHz, [D_6_]DMSO): *δ* (ppm) = 27.7 (C_7_, C_9_), 31.6 (C_6_, C_10_), 39.1 (N*C*H_2_COOH, *cis*), 39.4 (C_8_), 39.8 (N*C*H_2_COOH, *trans*), 41.2 (low) (N*C*H_2_Ph, *cis*), 44.2 (N*C*H_2_Ph, *trans*), 63.0 (C_5_), 126.0 (C_4′_), 126.5 (C_2Bz_, C_6Bz_), 126.8 (C_2′_, C_6′_), 128.3 (C_3′_, C_5′_, C_4Bz_), 128.5 (C_3Bz_, C_5Bz_), 138.2 (C_1Bz_), 145.3 (C_1′_), 155.6 (C_2_=O), 168.7 (NCH_2_*C*OOH), 175.9 (C_4_=O); elemental analysis calcd (%) for C_23_H_24_N_2_O_4_: C 70.39, H 6.16, N 7.14, found: C 70.46, H 6.19, N 7.02.

2-(1-Benzyl-2,4-dioxo-6-phenyl-1,3-diazaspiro [4.5]decan-3-yl)acetic acid (24). A mixture of benzyl ester 15 (350 mg, 0.73 mmol) and 10% Pd on charcoal (42 mg) in EtOH/AcOEt 3:1 (14.5 mL) was hydrogenated following the general procedure for the preparation of carboxylic acids to yield the title compound **24** as a foamy solid. Crystallization upon treatment with *n*-pentane (0 °C) afforded a white crystalline solid (276 mg, almost quantitative yield). Mp 119–123 °C (AcOEt/*n*-pentane, Et_2_O), R*_f_* = 0.29 (AcOEt). ^1^H NMR (600.11 MHz, DMSO-*d_6_*) *δ* (ppm): 1.26 (qt, 1H, *J_1_ =* 13.9 Hz, *J_2_ =* 3.9 Hz, H_9_), 1.41 (dt, 1H, *J_1_ =* 13.9 Hz, *J_2_ =* 3.8 Hz, H_9_), 1.49 (qt, 1H, *J_1_ =* 13.1 Hz, *J_2_ =* 4.0 Hz, H_8_), 1.69–1.78 (m, 2H, H_7_, H_10_), 1.88 (dt, 1H, *J_1_ =* 13.3 Hz, *J_2_ =* 3.5 Hz, H_8_), 1.95 (td, 1H, *J_1_ =* 14.3 Hz, *J_2_ =* 4.9 Hz, H_10_), 2.36 (qd, 1H, *J_1_ =* 13.7 Hz, *J_2_ =* 3.8 Hz, H_7_), 3.11 (dd, 1H, *J_1_ =* 14.0 Hz, *J_2_ =* 4.3 Hz, H_6_), 3.61 (brs, 1H, NCH_2_COO*H*, under DMSO-water peak), 3.61–3.90 (q, AB, 2H, *J_AB_ =* 17.3 Hz, NC*H_2_*COOH), 4.91–5.10 (q, AB, 2H, *J_AB_ =* 17.2 Hz, NC*H_2_*Ph), 7.13 (~dd, 2H, *J_1_ =* 7.0 Hz, *J_2_ =* 1.6 Hz, H_2′_, H_6′_), 7.20–7.35 (complex m, 8H, H_3′_, H_4′_, H_5′_, H_2Bz_, H_3Bz_, H_4Bz_, H_5Bz_, H_6Bz_); ^13^C NMR (50.32 ΜHz, DMSO-*d_6_*) *δ* (ppm): 20.0 (C_9_), 24.6 (C_8_), 26.9 (C_7_), 31.9 (C_10_), 39.5 (N*C*H_2_COOH), 45.7 (N*C*H_2_Ph), 49.3 (C_6_), 67.8 (C_5_), 126.1 (C_2Bz_, C_6Bz_), 126.9 (C_4Bz_), 127.2 (C_4′_), 128.1 (C_2′_, C_3′_, C_5′_, C_6′_), 128.3 (C_3Bz_, C_5Bz_), 137.9 (C_1Bz_), 139.1 (C_1′_), 155.3 (C_2_=O), 168.4 (NCH_2_*C*OOH), 175.0 (C_4_=O). Anal. Calcd for C_23_H_24_N_2_O_4_: C, 70.39; H, 6.16; N, 7.14. Found: C, 70.32; H, 6.14; N, 7.09.

2-(2,4-Dioxo-8-phenyl-1,3-diazaspiro [4.5]decan-3-yl)propanoic acid (47). A mixture of benzyl ester 46 (900 mg, 2.21 mmol) and 10% Pd on charcoal (108 mg) in EtOH/AcOEt 3:1 (44 mL) was hydrogenated following the general procedure described above for the preparation of carboxylic acids to yield the desired compound **47** as a white crystalline solid. (695 mg, almost quantitative yield); *R*_f_ = 0.13 (AcOEt); mp 236–238 °C (MeOH/*n*-pentane); ^1^H NMR (600.11 MHz, [D_6_]DMSO): *δ* (ppm) = 1.46 (d, *J* = 7.3 Hz, 3H, NCH(C*H_3_*)COOH), 1.62 (d, *J* = 12.3 Hz, 1.8H, H_6_, H_10_), 1.67–1.80 (complex m, 3.8H, H_7_, H_9_), 1.84 (tq, *J_1_* = 13.4 Hz, *J_1_* = 4.4 Hz, 2H, H_6_, H_10_), 2.12 (qd, *J_1_* = 13.7 Hz, *J_2_* = 4.3 Hz, 0.2H, H_7_, H_9_), 2.58 (tt, *J_1_* = 11.2 Hz, *J_2_* = 4.2 Hz, 1H, H_8_), 4.55 (q, *J* = 7.4 Hz, 0.1H, NC*H*(CH_3_)COOH), 4.58 (q, *J* = 7.3 Hz, 0.8H, NC*H*(CH_3_)COOH), 7.18 (td, *J_1_* = 7.1 Hz, *J_2_* = 1.8 Hz, 1H, H_4′_), 7.26 (dd, *J_1_* = 7.0 Hz, *J_2_* = 1.5 Hz, 0.2H, H_2′_, H_6′_), 7.29 (t, *J* = 7.5 Hz, 2H, H_3′_, H_5′_), 7.33 (dd, *J_1_* = 7.1 Hz, *J_2_* = 1.5 Hz, 1.8H, H_2′_, H_6′_), 8.29 (s, 0.1H, H_1_), 9.06 (s, 0.9H, H_1_), 12.92 (v br s, 1H, NCH(CH_3_)COO*H*); ^13^C NMR (150.9 MHz, [D_6_]DMSO): *δ* (ppm) = 14.5 (NCH(*C*H_3_)COOH), 28.4, 28.7 (C_7_, C_9_), 33.3, 33.9 (C_6_, C_10_), 41.3, 42.2 (C_8_), 46.7, 47.0 (N*C*H(CH_3_)COOH), 58.4, 60.6 (C_5_), 126.0 (C_4′_), 126.7, 126.9 (C_2′_, C_6′_), 128.2, 128.3 (C_3′_, C_5′_), 146.2, 146.6 (C_1′_), 154.8, 155.1 (C_2_=O), 171.0 (NCH(CH_3_)*C*OOH), 175.9, 176.3 (C_4_=O); elemental analysis calcd (%) for C_17_H_20_N_2_O_4_: C 64.54, H 6.37, N 8.86, found: C 64.50, H 6.48, N 8.88.

2-(1-Methyl-2,4-dioxo-8-phenyl-1,3-diazaspiro [4.5]decan-3-yl)propanoic acid (51). Benzyl ester 50 (800 mg, 1.90 mmol) in EtOH/AcOEt 3:1 (38 mL) was subjected to catalytic hydrogenolysis in the presence of 10% Pd on charcoal (96 mg) following the general procedure previously to yield the corresponding carboxylic acid **51** as a white crystalline solid. (625 mg, almost quantitative yield); *R*_f_ = 0.24 (AcOEt); mp 217–219 °C (AcOEt/*n*-pentane); ^1^H NMR (400.13 MHz, [D_6_]DMSO): *δ* (ppm) = 1.46 (d, *J* = 7.3 Hz, 3H, NCH(C*H_3_*)COOH), 1.68 (d, *J* = 13.0 Hz, 2H, H_6_, H_10_), 1.75 (d, *J* = 12.5 Hz, 2H, H_7_, H_9_), 2.00 (tt, *J_1_* = 13.2 Hz, *J_2_* = 4.0 Hz, 2H, H_6_, H_10_), 2.17 (qd, *J_1_* = 12.9 Hz, *J_2_* = 2.8 Hz, 2H, H_7_, H_9_), 2.60 (tt, *J_1_* = 12.5 Hz, *J_2_* = 3.8 Hz, 1H, H_8_), 2.81 (s, 3H, NC*H_3_*), 4.61 (q, *J* = 7.2 Hz, 1H, NC*H*(CH_3_)COOH), 7.19 (td, *J_1_* = 7.2 Hz, *J_2_* = 1.8 Hz, 1H, H_4′_), 7.24 (dd, *J_1_* = 7.1 Hz, *J_2_* = 1.7 Hz, 2H, H_2′_, H_6′_), 7.31 (t, *J* = 7.4 Hz, 2H, H_3′_, H_5′_), 13.03 (v br s, 1H, NCH(CH_3_)COO*H*); ^13^C NMR (150.9 MHz, [D_6_]DMSO): *δ* (ppm) = 14.5 (NCH(*C*H_3_)COOH), 23.6 (N*C*H_3_), 28.5, 28.6 (C_7_, C_9_), 29.9 (C_6_, C_10_), 41.4 (C_8_), 47.1 (N*C*H(CH_3_)COOH), 60.7 (C_5_), 126.1 (C_4′_), 126.6 (C_2′_, C_6′_), 128.4 (C_3′_, C_5′_), 146.3 (C_1′_), 154.1 (C_2_=O), 171.0 (NCH(CH_3_)*C*OOH), 175.0 (C_4_=O); elemental analysis calcd (%) for C_18_H_22_N_2_O_4_: C 65.44, H 6.71, N 8.48, found: C 65.53, H 6.78, N 8.45.

General experimental procedure for the preparation of *N*-(phelylmethoxy)acetamides (25–33, 48, and 52). Method A: To a solution of the carboxylic acid (1.5 mmol, 1.0 eq) in a mixture of dry CH_2_Cl_2_/dry DMF 4:1 (15 mL), EDCI·HCl (1.8 mmol, 1.2 eq) and HOBt (1.8 mmol, 1.2 eq, monohydrate, 97%) were added, followed by *O*-benzylhydroxylamine hydrochloride (1.8 mmol, 1.2 eq) and TEA (8.7 mmol, 5.8 eq). After stirring for 40–45 h at 30–35 °C under argon, CH_2_Cl_2_ was evaporated under vacuum. The reaction mixture was quenched with water (30 mL) and extracted with AcOEt (4 × 30 mL). The combined organic phases were washed with H_2_O (3 × 50 mL) and brine (2 × 50 mL), dried over anh. Na_2_SO_4,_ and concentrated under reduced pressure. The crude product was purified by column chromatography on silica gel to yield the corresponding acetamides or propanamides. Method B: The carboxylic acid (1.5 mmol, 1.0 eq) was dissolved in 20 mL dry THF, and to this solution, CDI (1.8 mmol, 1.2 eq) was added. After stirring for 1 h at 28 °C under argon, *O*-benzylhydroxylamine hydrochloride (1.8 mmol, 1.2 eq) and TEA (2.7 mmol, 1.8 eq) were added sequentially, and the stirring was continued for 24 h at 28 °C under argon and for 1 h at 40°C. Then, THF was evaporated under reduced pressure. The reaction mixture was poured into ice water (30 mL) and extracted with AcOEt (4 × 30 mL). The combined organic layers were washed with H_2_O (3 × 50 mL) and brine (2 × 50 mL), dried with anh. Na_2_SO_4,_ and concentrated under vacuum. The crude residue was purified by column chromatography on silica gel to yield the title acetamides or propanamides.

*N*-(Phenylmethoxy)-2-((4*R*,4′*S*)/(4*S*,4′*R*)-4′-methyl-2,5-dioxo-3′,4′-dihydro-2′*H*- spiro[imidazolidine-4,1′-naphthalene]-1-yl)acetamide (25). To a stirred solution of carboxylic acid 16 (650 mg, 2.29 mmol) in 23 mL dry CH_2_Cl_2_/dry DMF (4:1), EDCI·HCl (527 mg, 2.75 mmol), HOBt (434 mg, 2.75 mmol, monohydrate, 97%), *O*-benzylhydroxylamine hydrochloride (439 mg, 2.75 mmol), and TEA (1.35 g, 13.3 mmol) were added successively, and the mixture was allowed to react for 41 h at 30 °C under argon. Following the general procedure described for the preparation of *N*-(phenylmethoxy)acetamides (Method A), the off-yellow oily residue was chromatographed on silica gel with CH_2_Cl_2_/AcOEt 50:1, 30:1, 3:1 και AcOEt as eluents. The white foamy product obtained was dried under high vacuum to yield **25** as a glass solid. (500 mg, 55%), *R*_f_ = 0.08 (CH_2_Cl_2_/AcOEt 8:1); ^1^H NMR (400.13 MHz, [D_6_]DMSO): *δ* (ppm) = 1.27, 1.30 (d +d, *J* = 7.0 Hz, *J* = 6.9 Hz, 3H, C*H_3_*), 1.62 (ddd, *J_1_* = 13.9 Hz, *J_2_* = 10.9 Hz, *J_3_* = 5.2 Hz, 0.45H, H_3′_), 1.77–1.90 (m, 1H, H_2′_, H_3′_), 1.97–2.11 (m, 1.3H, H_2′_, H_3′_), 2.12–2.29 (m, 1H, H_2′_, H_3′_), 2.94 (td, *J_1_* = 12.8 Hz, *J_2_* = 6.1 Hz, 1H, *H_4′_*), 3.91–4.08 (2q, AB, 1.6H, *J_1AΒ_* = 16.1 Hz, *J_2AΒ_* = 16.2 Hz, NC*H_2_*CO), 4.27 (s, 0.35H, NC*H_2_*CO), 4.81, 4.87 (s + s, 2H, OC*H_2_*Ph), 7.21 (t, *J* = 7.6 Hz, 1H, H_7′_), 7.25–7.31 (m, 2H, H_6′_, H_8′_), 7.33 (d, *J* = 7.2 Hz, 1H, *H_5′_*), 7.35–7.53 (complex m, 5H, H_2″_, H_3″_, H_4″_, H_5″_, H_6″_), 8.94 (s, 1H, H_3_), 11.06 (s, 0.12H, CON*H*OCH_2_Ph), 11.44 (s, 0.72H, CON*H*OCH_2_Ph); ^13^C (50.32 MHz, [D_6_]DMSO): *δ* (ppm) = 21.9, 22.4 (*C*H_3_), 25.6, 26.5 (C_3′_), 30.0, 31.1 (C_2′_), 31.3, 31.7 (C_4′_), 38.0, 38.3 (N*C*H_2_CO), 62.6, 62.7 (C_4_), 77.0, 78.6 (low) (O*C*H_2_Ph), 126.4 (C_7′_), 127.4 (C_8′_), 127.9 (C_5′_), 128.4 (C_6′_, C_3″_, C_4″_, C_5″_), 128.8 (C_2″_, C_6″_), 133.6, 133.7 (C_8′a_), 135.8 (C_1″_), 142.6, 142.7 (C_4′a_), 155.3 (C_2_=O), 163.7 (*C*ONHOCH_2_Ph), 176.1 (C_5_=O). HRMS/ESI^+^: *m*/*z* calcd for C_22_H_23_N_3_O_4_: 393.1689; found: 393.1691.

*Ν*-(Phenylmethoxy)-2-((4*R*,4′*R*)/(4*S*,4′*S*)-4′-methyl-2,5-dioxo-3′,4′-dihydro-2′*H*- spiro[imidazolidine-4,1′-naphthalene]-1-yl)acetamide (26). Using the general procedure described for the preparation of *N*-(benzyloxy)acetamides (Method A), the carboxylic acid precursor 17 (280 mg, 0.97 mmol) was treated successively with EDCI·HCl (222 mg, 1.16 mmol), HOBt (183 mg, 1.16 mmol, monohydrate, 97%), *O*-benzylhydroxylamine hydrochloride (185 mg, 1.16 mmol), and TEA (570 mg, 5.63 mmol) in dry CH_2_Cl_2_/dry DMF 4:1 (10 mL). The reaction mixture was then worked up following the general synthetic procedure previously described, and the yellowish oily residue was purified by column chromatography on silica gel eluting with CH_2_Cl_2_/AcOEt 50:1, 30:1, 10:1 and then AcOEt. The obtained foamy product was dried under high vacuum to afford **26** as a glass solid. (230 mg, 60%); *R*_f_ = 0.09 (CH_2_Cl_2_/AcOEt 8:1); ^1^H NMR (400.13 MHz, [D_6_]DMSO): *δ* (ppm) = 1.27, 1.30 (d + d, *J* = 7.0 Hz, *J* = 6.9 Hz, 3H, C*H_3_*), 1.57–1.68 (m, 0.55H, H_3′_), 1.78–1.90 (m, 0.8H, H_2′_, H_3′_), 1.95–2.11 (m, 1.5H, H_2′_, H_3′_), 2.12–2.29 (m, 1H, H_2′_, H_3′_), 2.94 (tt, *J_1_* = 7.0 Hz, *J_2_* = 6.9 Hz, 1H, H_4′_), 3.90–4.07 (2q, AB, *J_1AΒ_* = *J_2AΒ_* = 16.2 Hz, 1.5H, NC*H_2_*CO), 4.27 (s, 0.35H, NC*H_2_*CO), 4.81, 4.87 (s + s, 2H, OC*H_2_*Ph), 7.21 (t, *J* = 7.4 Hz, 1H, H_7′_), 7.25–7.35 (m, 3H, H_5′_, H_6′_, H_8′_), 7.36–7.52 (m, 5H, H_2″_, H_3″_, H_4″_, H_5″_, H_6″_), 8.94 (s, 1H, H_3_), 11.06 (s, 0.12H, CON*H*OCH_2_Ph), 11.44 (s, 0.75H, CON*H*OCH_2_Ph); ^13^C (50.32 MHz, [D_6_]DMSO): *δ* (ppm) = 21.9, 22.4 (*C*H_3_), 25.6, 26.5 (C_3′_), 30.0, 31.1 (C_2′_), 31.2, 31.7 (C_4′_), 38.0 (N*C*H_2_CO), 62.5, 62.7 (C_4_), 77.0, 78.5 (O*C*H_2_Ph), 126.4 (C_7′_), 127.4 (C_8′_), 127.9 (C_5′_), 128.4 (C_6′_, C_3″_, C_4″_, C_5″_), 128.8 (C_2″_, C_6″_), 133.6, 133.7 (C_8′a_), 135.8 (C_1″_), 142.6 (C_4′a_), 155.3 (C_2_=O), 163.7 (*C*ONHOCH_2_Ph), 176.1 (C_5_=O). HRMS/ESI^+^: *m*/*z* calcd for C_22_H_23_N_3_O_4_: 393.1689; found: 393.1687. 

*N*-(Phenylmethoxy)-2-(2,4-dioxo-8-phenyl-1,3-diazaspiro [4.5]decan-3-yl)acetamide (27). The *N*-benzyloxy precursor 27 was prepared from carboxylic acid 18 (280 mg, 0.93 mmol) in dry THF (12 mL) upon treatment with CDI (182 mg, 1.12 mmol), *O*-benzylhydroxylamine hydrochloride (177 mg, 1.12 mmol), and TEA (169 mg, 1.67 mmol) successively following the same procedure described for the preparation of *O*-benzyl hydroxamates (Method B). The crude glass solid was purified by column chromatography on silica gel eluting with CH_2_Cl_2_/AcOEt 20:1, 10:1, 4:1, and then AcOEt to yield the corresponding *O*-benzyl hydroxamate **27** as a white semisolid, which was crystallized under cooling (white crystals, 280 mg, 74%); *R*_f_ = 0.29 (CH_2_Cl_2_/AcOEt 8:1); mp 193–195 °C (AcOEt/*n*-pentane); ^1^H NMR (600.11 MHz, [D_6_]DMSO): *δ* (ppm) = 1.70 (d, *J* = 12.6 Hz, 2H, H_6_, H_10_), 1.73–1.83 (m, 3.8H, H_7_, H_9_, *cis*), 1.86 (td, *J_1_* = 12.6 Hz, *J_2_* = 4.3 Hz, 1.7H, H_6_, H_10_, *cis*), 1.96 (d, *J* = 13.5 Hz, 0.25H, H_6_, H_10_, *trans*), 2.14 (qd, *J_1_* = 12.0 Hz, *J_2_* = 3.4 Hz, 0.15H, H_7_, H_9_, *trans*), 2.60 (dt, *J_1_* = 11.2 Hz, *J_2_* = 4.1 Hz, 1H, H_8_), 3.89 (low), 3.92 (s + s, 1.55H, NC*H_2_*CO), 4.20 (s, 0.30H, NC*H_2_*CO), 4.80, 4.86 (low) (s + s, 2H, OC*H_2_*Ph), 7.19 (tt, *J_1_* = 7.2 Hz, *J_2_* = 1.6 Hz, 1H, H_4′_, *cis*), 7.27 (d, *J* = 7.5 Hz, 0.2H, H_2′_, H_6′_, *trans*), 7.31 (t, *J* = 7.5 Hz, 2H, H_3′_, H_5′_), 7.35 (d, *J_1_* = 7.3 Hz, *J_2_* = 1.6 Hz, 1.8H, H_2′_, H_6′_, *cis*), 7.35–7.50 (m, 5H, H_2″_, H_3″_, H_4″_, H_5″_, H_6″_), 8.28 (low) (s, 0.05H, H_1_, *trans*), 9.04 (s, 0.82H, H_1_, *cis*), 10.99 (low) (s, 0.1H, CON*H*OCH_2_Ph), 11.35 (s, 0.7H, CON*H*OCH_2_Ph); ^13^C NMR (150.9 MHz, [D_6_]DMSO): *δ* (ppm) = 28.4 (C_7_, C_9_, *cis*), 28.7 (low) (C_7_, C_9_, *trans*), 33.4 (C_6_, C_10_, *cis*), 34.0 (low) (C_6_, C_10_, *trans*), 37.6 (low), 37.9 (N*C*H_2_CO), 41.3 (low) (C_8_, *trans*), 42.2 (C_8_, *cis*), 61.0 (low) (C_5_, *trans*), 61.1 (C_5_, *cis*), 77.1, 78.6 (low) (O*C*H_2_Ph), 126.0 (C_4′_), 126.7 (low) (C_2′_, C_6′_, *trans*), 126.9 (C_2′_, C_6′_, *cis*), 128.2 (C_3′_, C_5′_), 128.3 (C_3″_, C_4″_, C_5″_), 128.9, 129.3 (low) (C_2″_, C_6″_), 135.8 (C_1″_), 146.2 (low) (C_1′_, *trans*), 146.6 (C_1′_, *cis*), 155.0 (low) (C_2_=O, *trans*), 155.4 (C_2_=O, *cis*), 163.6, 168.8 (low) (*C*ONHOCH_2_Ph), 176.3 (low) (C_4_=O, *trans*), 176.7 (C_4_=O, *cis*); elemental analysis calcd (%) for C_23_H_25_N_3_O_4_: C 67.80, H 6.18, N 10.31, found: C 67.88, H 6.14, N 10.33.

*N*-(Phenylmethoxy)-2-(2,4-dioxo-6-phenyl-1,3-diazaspiro [4.5]decan-3-yl)acetamide (28). The *N*-benzyloxy analog 28 was prepared from carboxylic acid 19 (250 mg, 0.83 mmol) in a mixture of dry CH_2_Cl_2_/dry DMF (8.5 mL) upon treatment with EDCI·HCl (192 mg, 1.00 mmol), HOBt (139 mg, 1.00 mmol, monohydrate, 97%), *O*-benzylhydroxylamine hydrochloride (160 mg, 1.00 mmol), and TEA (486 mg, 4.8 mmol) following the amidation procedure described in the main manuscript (Method A). After removal of the CH_2_Cl_2_ under vacuum, the reaction mixture was poured into 17 mL ice water and extracted with AcOEt (4 × 17 mL). The combined organic phases were washed with H_2_O (3 × 28 mL) and brine (2 × 28 mL), dried with anh. Na_2_SO_4,_ and evaporated under reduced pressure. The resulting almost-colorless viscous oil was chromatographed on silica gel using CH_2_Cl_2_/AcOEt 20:1, 10:1, 5:1, and finally, 100% AcOEt as eluents to afford the corresponding *O*-benzyl hydroxamate **28** as a glass solid. The product was treated with *n*-pentane under ice cooling to yield a white crystalline solid (158 mg, 47%). Mp 189–191 °C (AcOEt/*n*-pentane), R*_f_* = 0.31 (CH_2_Cl_2_/AcOEt 6:1). ^1^H NMR (600.11 MHz, DMSO-*d_6_*) *δ* (ppm): 1.47 (qt, 1H, *J_1_* = 13.2 Hz, *J_2_* = 3.3 Hz, H_8_), 1.58 (tt, 1H, *J_1_* = 14.1 Hz, *J_2_* = 3.8 Hz, H_9_), 1.63 (dq, 1H, *J_1_* = 12.6 Hz, *J_2_* = 3.5 Hz, H_7_), 1.73 (d, 2H, *J* = 11.9 Hz, H_9_, H_10_), 1.81 (~dq, 1H, *J_1_* = 12.3 Hz, *J_2_* = 3.0 Hz, H_8_), 1.89 (td, 1H, *J_1_* = 13.0 Hz, *J_2_* = 3.7 Hz, H_10_), 2.02 (qd, 1H, *J_1_* = 13.2 Hz, *J_2_* = 3.6 Hz, H_7_), 2.97 (dd, 1H, *J_1_* = 13.4 Hz, *J_2_* = 3.5 Hz, H_6_), 3.31–3.49 (q, AB, 1.7H, *J_AB_* = 16.0 Hz, NC*H_2_*CONH, *E*/*Z*-isomer), 3.62–3.80 (low) (q, AB, 0.3H, *J_AB_* = 15.5 Hz, NC*H_2_*CONH, *E*/*Z*-isomer), 4.72, 4.74 (low) (s + brs, 2H, OC*H_2_*Ph, *E*/*Z* isomers), 7.09–7.13 (m, 2H, H_3′_, H_5′_), 7.16–7.25 (complex m, 3H, H_2′_, H_4′_, H_6′_), 7.30–7.41 (sym m, 5H, H_2″_, H_3″_, H_4″_, H_5″_, H_6″_), 8.93 (s, 1H, H_1_), 10.86 (low), 11.10 (brs + s, 2H, NCH_2_CON*H*, *E*/*Z* isomers); ^13^C NMR (100.61 MHz, DMSO-*d_6_*) *δ* (ppm): 20.6 (C_9_), 25.1 (C_8_), 26.9 (C_7_), 34.3 (C_10_), 37.3 (N*C*H_2_CONH), 47.4 (C_6_), 65.7 (C_5_), 77.0 (O*C*H_2_Ph), 126.9 (C_4′_), 127.7 (C_3′_, C_5′_), 128.3 (C_2′_, C_6′_, C_3″_, C_4″_, C_5″_), 128.8 (C_2″_, C_6″_), 135.7 (C_1″_), 139.6 (C_1′_), 155.3 (C_2_=O), 163.2 (NCH_2_*C*ONH), 175.3 (C_4_=O). Anal. Calcd for C_23_H_25_N_3_O_4_: C, 67.80; H, 6.18; N, 10.31. Found: C, 67.88; H, 6.23; N, 10.33.

*N*-(Phenylmethoxy)-2-(1-methyl-2,4-dioxo-8-phenyl-1,3-diazaspiro [4.5]decan-3-yl)acetamide (29). Using the general procedure described for the preparation of *N*-(benzyloxy)acetamides (Method A), the carboxylic acid precursor 20 (420 mg, 1.33 mmol) was treated successively with EDCI·HCl (307 mg, 1.60 mmol), HOBt (253 mg, 1.60 mmol, monohydrate, 97%), *O*-benzylhydroxylamine hydrochloride (255 mg, 1.60 mmol), and TEA (780 mg, 7.71 mmol) in dry CH_2_Cl_2_/dry DMF 4:1 (13.3 mL). The reaction mixture was then worked up following the general synthetic procedure described above, and the yellowish oily residue was chromatographed on a silica gel column with CH_2_Cl_2_/AcOEt 20:1, 10:1, and then AcOEt, as eluents. The obtained glass solid was solidified upon treatment with *n*-pentane and Et_2_O to afford 260 mg of **29** as white crystals (46%); *R*_f_ = 0.27 (CH_2_Cl_2_/AcOEt 7:1); mp 153–155 °C (AcOEt/dry Et_2_O-*n*-pentane); ^1^H NMR (400.13 MHz, CDCl_3_): *δ* (ppm) = 1.77–1.99 (m, 6H, H_6_, H_7_, H_9_, H_10_), 2.27–2.44 (m, 2H, H_7_, H_9_), 2.52 (t, *J* = 12.3 Hz, 1H, H_8_), 2.89 (s, 3H, C*H_3_*), 4.09 (s, 0.8H, NC*H_2_*CO), 4.40 (s, 0.8H, NC*H_2_*CO), 4.92 (s, 2H, OC*H_2_*Ph), 7.21 (td, 1H, *J_1_* = 6.5 Hz, *J_2_* = 2.3 Hz, H_4′_), 7.26–7.33 (m, 4H, H_2′_, H_3′,_ H_5′_, H_6′_), 7.33–7.45 (m, 5H, H_2″_, H_3″_, H_4″_, H_5″_, H_6″_), 8.22 (s, 0.35H, CON*H*OCH_2_Ph), 8.94 (s, 0.4H, CON*H*OCH_2_Ph); ^13^C NMR (150.9 MHz, CDCl_3_): *δ* (ppm) = 24.0 (*C*H_3_), 28.6, 28.9 (C_7_, C_9_), 31.1, 31.8 (C_6_, C_10_), 38.9, 39.2 (N*C*H_2_CO), 43.1 (C_8_), 62.3 (C_5_), 78.4, 79.8 (O*C*H_2_Ph), 126.5 (C_4′_), 126.9, 127.1 (C_2′_, C_6′_), 128.6, 128.7 (C_3′_, C_5′_, C_3″_, C_4″_, C_5″_), 129.4 (C_2″_, C_6″_), 134.3 (C_1″_), 146.1 (C_1′_), 155.1 (C_2_=O), 164.5 (*C*ONHOCH_2_Ph), 175.6 (C_4_=O); elemental analysis calcd (%) for C_24_H_27_N_3_O_4_: C 68.39, H 6.46, N 9.97, found: C 68.44, H 6.48, N 10.09.

*N*-(Phenylmethoxy)-2-(1-methyl-2,4-dioxo-6-phenyl-1,3-diazaspiro [4.5]decan-3-yl)acetamide (30). Using the general procedure described for the preparation of *N*-(phenylmethoxy)acetamides (Method A), the carboxylic acid precursor 21 (160 mg, 0.51 mmol) was treated with EDCI·HCl (116 mg, 0.61 mmol), HOBt (85 mg, 0.61 mmol, monohydrate, 97%), *O*-benzylhydroxylamine hydrochloride (97 mg, 0.61 mmol), and TEA (297 mg, 2.93 mmol) in dry CH_2_Cl_2_/dry DMF 4:1 (5 mL). After removal of the CH_2_Cl_2_ under reduced pressure, the reaction mixture was quenched with 10 mL ice water and extracted with AcOEt (4 × 10 mL). The combined organic phases were washed with H_2_O (3 × 17 mL) and brine (2 × 17 mL), dried with anh. Na2SO4, and evaporated in vacuo. The resulting yellowish crude product was chromatographed on silica gel using CH_2_Cl_2_/AcOEt 20:1, 10:1, and AcOEt as eluents to afford the corresponding *O*-benzyl hydroxamate **30** as an off-white glass solid. The title compound was crystallized to a white crystalline solid after treatment with *n*-pentane under ice cooling (106 mg, 50%). Mp 83–85 °C (AcOEt/*n*-pentane), R*_f_* = 0.25 (CH_2_Cl_2_/AcOEt 6:1^1^H NMR (600.11 MHz, DMSO-*d_6_*) *δ* (ppm): 1.52–1.62 (sym m, 1H, H_8_), 1.75 (dq, 1H, *J_1_* = 13.7 Hz, *J_2_* = 3.7 Hz, H_7_), 1.80–1.88 (m, 2H, H_9_), 1.92–2.02 (m, 2H, H_8_, H_10_), 2.02–2.11 (m, 1H, H_10_), 2.20 (qd, 1H, *J_1_* = 13.6 Hz, *J_2_* = 3.6 Hz, H_7_), 3.09 (dd, 1H, *J_1_* = 13.9 Hz, *J_2_* = 4.1 Hz, H_6_), 3.23 (s, 3H, NC*H_3_*), 3.39–3.62 (q, AB, 2H, *J_AB_* = 16.0 Hz, NC*H_2_*CONH), 4.73, 4.77 (low) (s + brs, 2H, OC*H_2_*Ph, *E*/*Z* isomers), 7.05–7.09 (m, 2H, H_2′_, H_6′_), 7.18–7.22 (m, 1H, H_4′_), 7.22–7.26 (m, 2H, H_3′_, H_5′_), 7.31–7.42 (m, 5H, H_2″_, H_3″_, H_4″_, H_5″_, H_6″_), 10.89 (low), 11.16 (vbs + s, 1H, NCH_2_CON*H*, *E*/*Z* isomers); ^13^C NMR (100.61 MHz, DMSO-*d_6_*) *δ* (ppm): 21.2 (C_9_), 24.9 (C_8_), 26.9 (C_7_), 29.7 (N*C*H_3_), 32.1 (C_10_), 38.2 (N*C*H_2_CONH), 48.8 (C_6_), 66.8 (C_5_), 77.0 (O*C*H_2_Ph), 127.1 (C_4′_), 127.9 (C_2′_, C_6′_), 128.0 (C_3′_, C_5′_), 128.3 (C_3″_, C_4″_, C_5″_), 128.8 (C_2″_, C_6″_), 135.7 (C_1″_), 139.3 (C_1′_), 154.7 (C_2_=O), 163.0 (NCH_2_*C*ONH), 175.0 (C_4_=O). Anal. Calcd for C_24_H_27_N_3_O_4_: C, 68.39; H, 6.46; N, 9.97. Found: C, 68.38; H, 6.51; N, 9.99.

*N*-(Phenylmethoxy)-2-(1-ethyl-2,4-dioxo-8-phenyl-1,3-diazaspiro [4.5]decan-3-yl)acetamide (31). Carboxylic acid 22 (290 mg, 0.88 mmol) was treated with CDI (172 mg, 1.06 mmol), *O*-benzylhydroxylamine hydrochloride (169 mg, 1.06 mmol), and TEA (160 mg, 1.58 mmol) in 12 mL dry THF as described for the synthesis of *N*-(phenylmethoxy)acetamides (Method B). The reaction was worked up as previously described, and the resulting oily residue was purified by column chromatography on silica gel using CH_2_Cl_2_/AcOEt 20:1, 5:1, and AcOEt as eluents to afford the title compound **31** as a white foamy solid, which strongly binds the elution solvents. Removal of the entrapped solvents yielded **31** as a glass solid. (250 mg, 65%); *R*_f_ = 0.12 (CH_2_Cl_2_/AcOEt 8:1); ^1^H NMR (400.13 MHz, [D_6_]DMSO): *δ* (ppm) = 1.16 (t, *J* = 7.0 Hz, 3H, NCH_2_C*H_3_*), 1.56 (qd, *J*_1_ = 6.6 Hz, *J_2_* = 2.7 Hz, 0.2H, H_7_, H_9_), 1.77 (t, *J* = 12.5 Hz, 0.5H, H_7_, H_9_), 1.84–2.10 (complex m, 7H, H_6_, H_7_, H_9_, H_10_), 2.16 (dt, *J*_1_ = 13.2 Hz, *J_2_* = 6.6 Hz, 0.2H, H_7_, H_9_), 2.31 (td, *J*_1_ = 7.5 Hz, *J_2_* = 5.3 Hz, 0.1H, H_6_, H_10_), 2.64 (t, *J* = 11.5 Hz, 0.1H, H_8_, *trans*), 2.74–2.86 (m, 0.9H, H_8_, *cis*), 3.40 (qd, *J_1_* = 6.4 Hz, *J_2_* = 2.3 Hz, 0.2H, NC*H_2_*NCH_3_), 3.53 (q, *J* = 7.1 Hz, 1.8H, NC*H_2_*NCH_3_), 3.95 (s, 1.6H, NC*H_2_*CO), 4.22 (s, 0.4H, NC*H_2_*CO), 4.80, 4.86 (s + s, 2H, OC*H_2_*Ph), 7.21 (ddd, *J_1_* = 8.7 Hz, *J_2_* = 5.9 Hz, *J_3_* = 2.5 Hz, 1H, H_4′_), 7.28–7.50 (m, 9H, H_2′_, H_3′_, H_5′_, H_6′_, H_2″_, H_3″_, H_4″_, H_5″_, H_6″_), 11.03 (s, 0.18H, CON*H*OCH_2_Ph), 11.40 (s, 0.82H, CON*H*OCH_2_Ph); ^13^C NMR (150.9 MHz, [D_6_]DMSO): *δ* (ppm) = 14.6, 15.0 (NCH_2_*C*H_3_), 25.8, 27.7, 28.7, 28.9, 29.4 (C_7_, C_9_), 31.1, 31.7 (C_6_, C_10_), 36.4 (N*C*H_2_CH_3_), 37.9, 38.4 (N*C*H_2_CO), 39.5 (C_8_), 62.2, 62.4 (C_5_), 77.1, 78.6 (O*C*H_2_Ph), 126.1 (C_4′_), 126.6, 126.9 (C_2′_, C_6′_), 128.3 (C_3′_, C_5′_), 128.4 (C_3″_, C_4″_, C_5″_), 128.9, 129.3 (C_2″_, C_6″_), 135.8 (C_1″_), 145.2, 146.4 (C_1′_), 154.1, 154.3 (C_2_=O), 163.5, 168.6 (*C*ONHOCH_2_Ph), 175.3, 175.9 (C_4_=O).

*N*-(Phenylmethoxy)-2-(1-benzyl-2,4-dioxo-8-phenyl-1,3-diazaspiro [4.5]decan-3-yl)acetamide (32). The *N*-benzyloxy precursor 32 was prepared from carboxylic acid 23 (190 mg, 0.48 mmol) in dry CH_2_Cl_2_/dry DMF 4:1 (5 mL) upon treatment with EDCI·HCl (111 mg, 0.58 mmol), HOBt (92 mg, 0.58 mmol, monohydrate, 97%), *O*-benzylhydroxylamine hydrochloride (93 mg, 0.58 mmol), and TEA (281 mg, 2.78 mmol) sequentially, following the general procedure described for the preparation of *N*-(phenylmethoxy)acetamides (Method A). The yellowish oily residue was purified by column chromatography on silica gel with CH_2_Cl_2_/AcOEt 30:1, 20:1, and then AcOEt as eluents to afford the *N*-benzyloxy precursor **32** as a white foamy solid, which strongly binds the elution solvents. Removal of the entrapped solvents upon drying under high vacuum yielded **32** as a glass solid, which was solidified upon treatment with *n*-pentane (0 °C) to afford white crystals. (110 mg, 46%); *R*_f_ = 0.30 (CH_2_Cl_2_/AcOEt 8:1); mp 61–63 °C (AcOEt/*n*-pentane); ^1^H NMR (600.11 MHz, [D_6_]DMSO): *δ* (ppm) = 1.62 (qd, *J_1_* = 10.3 Hz, *J_2_* = 5.1 Hz, 1.8H, H_7_, H_9_, *trans*), 1.67–1.90 (complex m, 6H, H_7_, H_9_, H_6_, H_10_, *trans*), 1.93 (td, *J_1_* = 13.8 Hz, *J_2_* = 4.0 Hz, 0.6H, H_6_, H_10_, *cis*), 2.15 (qd, *J_1_* = 12.7 Hz, *J_2_* = 2.4 Hz, 0.4H, H_7_, H_9_, *cis*), 2.57 (tt, *J_1_* = 12.4 Hz, *J_2_* = 3.1 Hz, 0.1H, H_8_, *cis*), 2.67 (td, *J_1_* = 8.8 Hz, *J_2_* = 3.8 Hz, 0.9H, H_8_, *trans*), 4.00, 4.04 (s + s, 1.6H, NC*H_2_*CO), 4.31 (s, 0.35H, NC*H_2_*CO), 4.59 (s, 0.35H, NC*H_2_*Ph), 4.80 (s, 1.6H, NC*H_2_*Ph), 4.82, 4.88 (s + s, 2H, OC*H_2_*Ph), 7.15–7.50 (complex m, 15H, H_Ar_), 11.05 (s, 0.15H, CON*H*OCH_2_Ph), 11.41 (s, 0.72H, CON*H*OCH_2_Ph); ^13^C NMR (100.61 MHz, [D_6_]DMSO): *δ* (ppm) = 27.8, 28.6 (C_7_, C_9_), 31.3, 31.7 (C_6_, C_10_), 38.7, 38.9 (N*C*H_2_CO), 39.1 (C_8_), 44.2 (N*C*H_2_Ph), 62.6, 63.0 (C_5_), 77.1, 78.6 (O*C*H_2_Ph), 126.0 (C_4′_), 126.5 (C_2Bz_, C_6Bz_), 126.8 (C_2′_, C_6′_) 127.0 (C_2Bz_, C_6Bz_), 127.2 (C_4Bz_), 128.4 (C_3Bz_, C_5Bz_, C_3′_, C_5′_, C_4″_), 128.5 (C_3″_, C_5″_),128.9, 129.3 (C_2″_, C_6″_), 135.8 (C_1″_), 138.3, 138.6 (C_1Bz_), 145.4, 146.3 (C_1′_), 155.1, 155.7 (C_2_=O), 163.5, 163.6 (*C*ONHOCH_2_Ph), 175.2, 176.1 (C_4_=O); elemental analysis calcd (%) for C_30_H_31_N_3_O_4_: C 72.41, H 6.28, N 8.44, found: C 72.49, H 6.24, N 8.55.

*N*-(Phenylmethoxy)-2-(1-benzyl-2,4-dioxo-6-phenyl-1,3-diazaspiro [4.5]decan-3-yl)acetamide (33). Carboxylic acid 24 (235 mg, 0.60 mmol) was treated with CDI (117 mg, 0.72 mmol), *O*-benzylhydroxylamine hydrochloride (115 mg, 0.72 mmol), and TEA (109 mg, 1.08 mmol) in 8 mL dry THF as described for the synthesis of *O*-benzyl hydroxamates (Method B). The reaction was worked up in exactly the same way described in the main manuscript, and the resulting oily residue was purified by column chromatography on silica gel using CH_2_Cl_2_, CH_2_Cl_2_/AcOEt 20:1, 5:1, and AcOEt as eluents to yield the corresponding *O*-benzyl hydroxamate **33** as a colorless oily product. Crystallization upon treatment with *n*-pentane/Et_2_O afforded a white crystalline solid (131 mg, 44%). Mp 157–159 °C (AcOEt/*n*-pentane), R*_f_* = 0.42 (CH_2_Cl_2_/AcOEt 8:1). ^1^H NMR (600.11 MHz, DMSO-*d_6_*) *δ* (ppm): 1.27 (qt, 1H, *J_1_* = 17.6 Hz, *J_2_* = 4.0 Hz, H_9_), 1.42 (d, 1H, *J* = 14.0 Hz, H_9_), 1.49 (qt, 1H, *J_1_* = 13.7 Hz, *J_2_* = 4.0 Hz, H_8_), 1.75 (td, 2H, *J_1_* = 13.6 Hz, *J_2_* = 6.7 Hz, H_7_, H_10_), 1.89 (d, 1H, *J* = 13.3 Hz, H_8_), 1.96 (td, 1H, *J_1_* = 14.3 Hz, *J_2_* = 4.7 Hz, H_10_), 2.35 (qd, 1H, *J_1_* = 13.8 Hz, *J_2_* = 3.7 Hz, H_7_), 3.09 (dd, 1H, *J_1_* = 14.0 Hz, *J_2_* = 4.3 Hz, H_6_), 3.40–3.74 (q, AB, 2H, *J_AB_* = 16.1 Hz, NC*H_2_*CONH), 4.76, 4.79 (low) (s + brs, 2H, OC*H_2_*Ph, *E*/*Z* isomers), 4.90–5.11 (q, AB, 2H, *J_AB_* = 17.3 Hz, NC*H_2_*Ph), 7.09–7.14 (m, 2H, H_2′_, H_6′_), 7.20–7.43 (complex m, 13H, H_3′_, H_4′_, H_5′_, H_2″_, H_3″_, H_4″_, H_5″_, H_6″_, H_2Bz_, H_3Bz_, H_4Bz_, H_5Bz_, H_6Bz_), 11.22 (vbs, 1H, NCH_2_CON*H*); ^13^C NMR (50.32 ΜHz, DMSO-*d_6_*) *δ* (ppm): 20.0 (C_9_), 24.6 (C_8_), 26.9 (C_7_), 31.7 (C_10_), 38.4 (N*C*H_2_CONH), 45.7 (Ν*C*H_2_Ph), 49.5 (C_6_), 67.7 (C_5_), 77.0 (O*C*H_2_Ph), 126.1 (C_2Bz_, C_6Bz_), 126.9 (C_4Bz_), 127.2 (C_4′_), 128.0 (C_3′_, C_5′_, C_3Bz_, C_5Bz_), 128.3 (C_2′_, C_6′_, C_3″_, C_4″_, C_5″_), 128.8 (C_2″_, C_6″_), 135.7 (C_1″_), 137.9 (C_1Bz_), 139.1 (C_1′_), 155.4 (C_2_=O), 163.0 (NCH_2_*C*ONH), 175.1 (C_4_=O). Anal. Calcd for C_30_H_31_N_3_O_4_: C, 72.41; H, 6.28; N, 8.44. Found: C, 72.38; H, 6.24; N, 8.49.

*N*-(Phenylmethoxy)-2-(2,4-dioxo-8-phenyl-1,3-diazaspiro [4.5]decan-3-yl)propanamide (48). The *N*-benzyloxy precursor 48 was prepared from carboxylic acid 47 (600 mg, 1.90 mmol) in a mixture of dry CH_2_Cl_2_/dry DMF 4:1 (19 mL) upon treatment with EDCI·HCl (437 mg, 2.28 mmol), HOBt (360 mg, 2.28 mmol, monohydrate, 97%), *O*-benzylhydroxylamine hydrochloride (364 mg, 2.28 mmol), and TEA (1.12 g, 11.02 mmol) according to the procedure described for the preparation of acetamides (Method A). The crude colorless oily residue was chromatographed on silica gel with CH_2_Cl_2_/AcOEt 20:1, 7:1, and then AcOEt to afford the corresponding *O*-benzyl hydroxamate **48** as a glass solid, which was crystallized upon treatment with *n*-pentane under ice cooling. (240 mg, 30%); *R*_f_ = 0.21 (CH_2_Cl_2_/AcOEt 8:1); mp 157–159 °C (AcOEt/*n*-pentane); ^1^H NMR (600.11 MHz, [D_6_]DMSO): *δ* (ppm) = 1.45 (d, *J* = 7.2 Hz, 3H, NCH(C*H_3_*)CO), 1.59–1.90 (complex m, 8H, H_6_, H_7_, H_9_, H_10_), 2.55–2.63 (complex m, 1H, H_8_), 4.42 (q, *J* = 7.1 Hz, 0.1H, NC*H*(CH_3_)CO), 4.46 (q, *J* = 7.1 Hz, 0.8H, NC*H*(CH_3_)CO), 5.02–5.23 (q, AB, *J_AB_* = 10.8 Hz, 2H, OC*H_2_*Ph), 7.19 (tt, *J_1_* = 7.2 Hz, *J_2_* = 1.4 Hz, 1H, H_4′_), 7.25 (d, *J* = 7.4 Hz, 0.2H, H_2′_, H_6′_), 7.29 (t, *J* = 7.5 Hz, 2H, H_3′_, H_5′_), 7.33 (dd, *J_1_* = 7.7 Hz, *J_2_* = 1.2 Hz, 1.8H, H_2′_, H_6′_), 7.34–7.43 (complex m, 5H, H_2″_, H_3″_, H_4″_, H_5″_, H_6″_), 8.22 (s, 0.05H, H_1_), 8.98, 9.02 (s + s, 0.9H, H_1_), 11.27 (s, 0.85H, CON*H*OCH_2_Ph); ^13^C NMR (150.9 MHz, [D_6_]DMSO): *δ* (ppm) = 14.5 (NCH(*C*H_3_)CO), 28.3 (C_7_, C_9_), 33.2 (C_6_, C_10_), 42.2 (C_8_), 46.7 (N*C*H(CH_3_)CO), 60.4 (C_5_), 76.8 (O*C*H_2_Ph), 126.0 (C_4′_), 126.9 (C_2′_, C_6′_), 128.15 (C_3′_, C_5′_), 128.21 (C_3″_, C_4″_, C_5″_), 128.8 (C_2″_, C_6″_), 135.8 (C_1″_), 146.6 (C_1′_), 155.1 (C_2_=O), 166.0 (*C*ONHOCH_2_Ph), 176.4 (C_4_=O); elemental analysis calcd (%) for C_24_H_27_N_3_O_4_: C 68.39, H 6.46, N 9.97, found: C 68.45, H 6.57, N 10.02.

*N*-(Phenylmethoxy)-2-(1-methyl-2,4-dioxo-8-phenyl-1,3-diazaspiro [4.5]decan-3-yl)propanamide (52). Prepared from carboxylic acid 51 (500 mg, 1.51 mmol) upon treatment with CDI (293 mg, 1.81 mmol), *O*-benzylhydroxylamine hydrochloride (289 mg, 1.81 mmol), and TEA (275 mg, 2.72 mmol) sequentially in dry THF (20 mL) following the general procedure for the preparation of *O*-benzyl hydroxamates (Method B). The colorless oil obtained was chromatographed on silica gel with CH_2_Cl_2_/AcOEt 15:1, 8:1, and then AcOEt to afford the title compound **52** as a glass solid. The product was crystallized upon treatment with *n*-pentane under ice cooling. (470 mg, 71%); *R*_f_ = 0.30 (CH_2_Cl_2_/AcOEt 8:1); mp 103–106 °C (AcOEt/*n*-pentane); ^1^H NMR (600.11 MHz, [D_6_]DMSO): *δ* (ppm) = 1.46 (d, *J* = 7.3 Hz, 3H, NCH(C*H_3_*)CO), 1.69–1.82 (m, 4H, H_6_, H_7_, H_9_, H_10_), 1.96 (tt, *J_1_* = 13.2 Hz, *J_2_* = 4.8 Hz, 2H, H_6_, H_10_), 2.17 (q, *J* = 12.9 Hz, 2H, H_7_, H_9_), 2.60 (t, *J* = 12.4 Hz, 1H, H_8_), 2.80 (s, 3H, NC*H_3_*), 4.50 (q, *J* = 7.1 Hz, 1H, NC*H*(CH_3_)CO), 4.71–4.83 (q, AB, *J_AB_* = 10.8 Hz, 2H, OC*H_2_*Ph), 7.19 (t, *J* = 7.3 Hz, 1H, H_4′_), 7.25 (d, *J* = 7.6 Hz, 2H, H_2′_, H_6′_), 7.31 (t, *J* = 7.5 Hz, 2H, H_3′_, H_5′_), 7.32–7.44 (complex m, 5H, H_2″_, H_3″_, H_4″_, H_5″_, H_6″_), 9.81 (s, 0.07H, CON*H*OCH_2_Ph), 10.79 (s, 0.03H, CON*H*OCH_2_Ph), 11.29 (s, 0.9H, CON*H*OCH_2_Ph); ^13^C NMR (150.9 MHz, [D_6_]DMSO): *δ* (ppm) = 14.4 (NCH(*C*H_3_)CO), 23.5 (N*C*H_3_), 28.5, 28.6 (C_7_, C_9_), 29.78, 29.85 (C_6_, C_10_), 41.5 (C_8_), 46.8 (N*C*H(CH_3_)CO), 60.6 (C_5_), 76.8, 77.3 (O*C*H_2_Ph), 126.0 (C_4′_), 126.6 (C_2′_, C_6′_), 128.1 (C_3′_, C_5′_), 128.2 (C_3′_, C_5′_, C_4″_), 128.3 (C_3″_, C_5″_), 128.6, 128.8 (C_2″_, C_6″_), 135.8 (C_1″_), 146.4 (C_1′_), 154.1 (C_2_=O), 166.0 (*C*ONHOCH_2_Ph), 175.0 (C_4_=O); elemental analysis calcd (%) for C_25_H_29_N_3_O_4_: C 68.95, H 6.71, N 9.65, found: C 69.00, H 6.75, N 9.67.

General experimental procedure for the preparation of *N*-(hydroxy)acetamides (34–42, 49, and 53). A mixture of the *N*-benzyloxy precursor (1.0 mmol) and 10 wt.% Pd on charcoal in EtOH/AcOEt 3:1 (40 mL) was subjected to catalytic hydrogenolysis for 3 h under an atmosphere of 50–55 psi hydrogen at 44–46 °C. The catalyst was removed by filtration and washed with portions of hot MeOH (3 × 15 mL). The combined filtrates were concentrated to dryness under reduced pressure to obtain the desired acetohydroxamic acid analogs. 

*Ν*-(hydroxy)-2-((4*R*,4′*S*)/(4*S*,4′*R*)-4′-methyl-2,5-dioxo-3′,4′-dihydro-2′*H*- spiro[imidazolidine-4,1′-naphthalene]-1-yl)acetamide (34). A mixture of *O*-benzyl hydroxamate 25 (400 mg, 1.02 mmol) and 10% Pd on charcoal (48 mg) in EtOH/AcOEt 3:1 (41 mL) was subjected to catalytic hydrogenolysis following the general procedure previously described to afford the title compound 34 as a glass solid. The product was chromatographed on silica gel eluting with CH_2_Cl_2_/AcOEt 5:1, AcOEt, and AcOEt/MeOH 20:1 to yield 34 as a white foamy product, which strongly binds the elution solvents. Removal of the entrapped solvents yielded **34** as a glass solid, which was crystallized upon treatment with dry Et_2_O to afford a white semisolid. (375 mg, almost quantitative yield); *R*_f_ = 0.38 (AcOEt); mp melted gradually from 95 °C (AcOEt/dry Et_2_O); ^1^H NMR (600.11 MHz, [D_6_]DMSO): *δ* (ppm) = 1.27, 1.30 (d + d, *J* = 7.1 Hz, *J* = 6.9 Hz, 3H, C*H_3_*), 1.62 (tdd, *J_1_* = 13.2 Hz, *J_2_* = 9.0 Hz, *J_3_* = 3.0 Hz, 0.5H, H_3′_), 1.79–1.89 (m, 1H, H_2′_, H_3′_), 2.01 (ddd, *J_1_* = 12.6 Hz, *J_2_* = 7.9 Hz, *J_3_* = 3.3 Hz, 0.5H, H_3′_), 2.05 (td, *J_1_* = 10.5 Hz, *J_2_* = 3.1 Hz, 1H, H_2′_), 2.16 (qd, *J_1_* = 8.7 Hz, *J_2_* = 3.4 Hz, 0.5H, H_3′_), 2.24 (~t, *J* = 11.2 Hz, 0.5H, H_2′_), 2.94 (dq, *J_1_* = 13.4 Hz, *J_2_* = 6.6 Hz, 1H, H_4′_), 3.91–4.03 (2q, AB, *J_1A_* = *J_2AB_* = 16.0 Hz, 1.5H, NC*H_2_*CO, *E*-isomer), 4.20–4.32 (2q, AB, *J_1AB_* = *J_2AB_* = 17.2 Hz, 0.5H, NC*H_2_*CO, *Z*-isomer), 7.18 (t, *J* = 7.9 Hz, 1H, H_7′_), 7.24–7.41 (complex m, 3H, H_5′_, H_6′_, H_8′_), 8.86 (s, 1H, H_3_), 8.96 (s, 0.68H, NCH_2_CONHO*H*, *E*-isomer), 9.34 (s, 0.2H, NCH_2_CONHO*H*, *Z*-isomer), 10.28 (s, 0.2H, NCH_2_CON*H*OH, *Z*-isomer), 10.72 (s, 0.66H, NCH_2_CON*H*OH, *E*-isomer); ^13^C NMR (150.9 MHz, [D_6_]DMSO): *δ* (ppm) = 21.9, 22.3 (*C*H_3_), 25.6, 26.5 (C_3′_), 29.9, 31.1 (C_2′_), 31.2, 31.6 (C_4′_), 37.9 (N*C*H_2_CO, *E*-isomer), 38.5 (N*C*H_2_CO, *Z*-isomer), 62.4, 62.6 (C_4_), 126.3 (C_7′_), 127.4, 127.8 (C_8′_), 128.2, 128.4 (C_5′_, C_6′_), 133.6, 133.7 (C_8′a_), 142.5, 142.6 (C_4′a_), 155.3 (C_2_=O, *E*-isomer), 155.5 (C_2_=O, *Z*-isomer), 163.4 (NCH_2_*C*ONHOH, *E*-isomer), 168.8 (NCH_2_*C*ONHOH, *Z*-isomer), 176.1 (C_5_=O, *E*-isomer), 176.3 (C_5_=O, *Z*-isomer); elemental analysis calcd (%) for C_15_H_17_N_3_O_4_: C 59.40, H 5.65, N 13.85; found: C 59.45, H 5.61, N 13.93.

*Ν*-(Hydroxy)-2-((4*R*,4′*R*)/(4*S*,4′*S*)-4′-methyl-2,5-dioxo-3′,4′-dihydro-2′*H*- spiro[imidazolidine-4,1′-naphthalene]-1-yl)acetamide (35). A solution of the *N*-phenylmethoxy acetamide 26 (180 mg, 0.46 mmol) in a mixture of EtOH/AcOEt (3:1, 18 mL) was subjected to hydrogenolysis over Pd/C (22 mg) according to the procedure described for the preparation of hydroxamate analogs. Evaporation of the solvents in vacuo yielded the title compound 35 as a white foamy product, which strongly binds the solvents. Removal of the entrapped solvents under high vacuum afforded **35** as a glass solid. Crystallization upon treatment with *n*-pentane and dry Et_2_O yielded **35** as a white semisolid. (139 mg, almost quantitative yield); *R*_f_ = 0.40 (AcOEt); mp melted gradually from 83 °C (AcOEt/dry Et_2_O-*n*-pentane); ^1^H NMR (400.13 MHz, [D_6_]DMSO): *δ* (ppm) = 1.26, 1.29 (d + d, *J* = 7.0 Hz, *J* = 6.9 Hz, 3H, C*H_3_*), 1.56–1.67 (m, 0.55H, H_3′_), 1.77–1.89 (m, 0.8H, H_2′_, H_3′_), 2.03 (td, *J_1_* = 10.1 Hz, *J_2_* = 2.8 Hz, 1.6H, H_2′_, H_3′_), 2.11–2.29 (m, 1H, H_2′_, H_3′_), 2.86–3.00 (dt, *J_1_* = 13.5 Hz, *J_2_* = 7.0 Hz, 1H, H_4′_), 3.89–4.08 (2q, AB, *J_1AΒ_* = 16.1 Hz, *J_2AΒ_* = 16.5 Hz, 1.5H, NC*H_2_*CO, *E*-isomer), 4.19–4.32 (2q, AB, *J_1AΒ_* = 17.5 Hz, *J_2AΒ_* = 17.2 Hz, 0.5H, NC*H_2_*CO, *Z*-isomer), 7.18 (t, *J* = 7.7 Hz, 1H, H_7′_), 7.23–7.42 (complex m, 3H, H_5′_, H_6′_, H_8′_), 8.86, 8.91 (s + s, 1H, H_3_), 9.02 (s, 0.6H, NCH_2_CONHO*H*, *E*-isomer), 9.39 (s, 0.2H, NCH_2_CONHO*H*, *Z*-isomer), 10.34 (s, 0.2H, NCH_2_CON*H*OH, *Z*-isomer), 10.76 (s, 0.6H, NCH_2_CON*H*OH, *E*-isomer); ^13^C NMR (50.32 MHz, [D_6_]DMSO): *δ* (ppm) = 21.9, 22.4 (*C*H_3_), 25.6, 26.5 (C_3′_), 29.9 (C_2′_), 31.2, 31.7 (C_4′_), 38.3 (N*C*H_2_CO, *E*-isomer), 39.9 (N*C*H_2_CO, *Z*-isomer), 62.5, 62.7 (C_4_), 126.4 (C_7′_), 127.5, 127.8 (C_6′_, C_8′_), 128.3, 128.5 (C_5′_), 133.7, 133.8 (C_8′a_), 142.6 (C_4′a_), 155.4 (C_2_=O), 163.4 (NCH_2_*C*ONHOH, *E*-isomer), 168.9 (NCH_2_*C*ONHOH, *Z*-isomer), 176.2 (C_5_=O); elemental analysis calcd (%) for C_15_H_17_N_3_O_4_: C 59.40, H 5.65, N 13.85; found: C 59.45, H 5.60, N 13.89. 

*N*-Hydroxy-2-(2,4-dioxo-8-phenyl-1,3-diazaspiro [4.5]decan-3-yl) acetamide (36). A solution of the *N*-phenylmethoxy acetamide 27 (220 mg, 0.54 mmol) in a mixture of EtOH/AcOEt (3:1, 22 mL) was subjected to hydrogenolysis in the presence of Pd/C (26 mg) according to the procedure described for the preparation of hydroxamate analogs. Evaporation of the solvents in vacuo yielded the title compound 36 as a white crystalline solid. (170 mg, almost quantitative yield); *R*_f_ = 0.62 (AcOEt); mp 217–219 °C (MeOH/dry Et_2_O-*n*-pentane); ^1^H NMR (600.11 MHz, [D_6_]DMSO): *δ* (ppm) = 1.68 (d, *J* = 11.3 Hz, 2H, H_6_, H_10_), 1.71–1.83 (complex m, 3.9H, H_7_, H_9_, *cis*), 1.85 (td, *J_1_* = 12.3 Hz, *J_2_* = 4.0 Hz, 1.9H, H_6_, H_10_), 1.95 (dt, *J_1_* = 13.9 Hz, *J_2_* = 4.6 Hz, 0.1H, H_6_, H_10_, *trans*), 2.10 (qd, *J_1_* = 13.1 Hz, *J_2_* = 3.9 Hz, 0.1H, H_7_, H_9_, *trans*), 2.58 (tt, *J_1_* = 11.2 Hz, *J_2_* = 4.1 Hz, 1H, H_8_), 3.86 (low), 3.89 (s + s, 1.5H, NC*H_2_*CO, *E*-isomer), 4.16 (low), 4.19 (s + s, 0.45H, NC*H_2_*CO, *Z*-isomer), 7.18 (tt, *J_1_* = 7.2 Hz, *J_2_* = 1.4 Hz, 1H, H_4′_), 7.25 (dd, *J_1_* = 7.0 Hz, *J_2_* = 1.6 Hz, 0.2H, H_2′_, H_6′_, *trans*), 7.29 (td *J_1_* = 7.6 Hz, *J_2_* = 1.6 Hz, 2H, H_3′_, H_5′_), 7.33 (dd, *J_1_* = 7.2 Hz, *J_2_* = 1.6 Hz, 1.8H, H_2′_, H_6′_, *cis*), 8.22, 8.24 (s + s, 0.05H, H_1_, *trans*), 8.91 (s, 0.6H, NCH_2_CONHO*H*, *E*-isomer), 8.99, 9.00 (s + s, 0.95H, H_1_, *cis*), 9.31 (s, 0.2H, NCH_2_CONHO*H*, *Z*-isomer), 10.25 (s, 0.2H, NCH_2_CON*H*OH, *Z*-isomer), 10.67 (s, 0.6H, NCH_2_CON*H*OH, *E*-isomer); ^13^C NMR (150.9 MHz, [D_6_]DMSO): *δ* (ppm) = 28.4 (C_7_, C_9_, *cis*), 28.7 (low) (C_7_, C_9_, *trans*), 33.3 (C_6_, C_10_, *cis*), 33.9 (low) (C_6_, C_10_, *trans*), 37.9 (N*C*H_2_CO, *E*-isomer), 38.2 (N*C*H_2_CO, *Z*-isomer), 41.3 (low) (C_8_, *trans*), 42.2 (C_8_, *cis*), 58.8 (low) (C_5_, *trans*), 60.9 (C_5_, *cis*), 126.0 (C_4′_), 126.6 (low) (C_2′_, C_6′_, *trans*), 126.9 (C_2′_, C_6′_, *cis*), 128.2 (C_3′_, C_5′_, *cis*), 128.3 (low) (C_3′_, C_5′_, *trans*), 146.2 (low) (C_1′_, *trans*), 146.6 (C_1′_, *cis*), 155.4 (C_2_=O, *E*-isomer), 155.6 (low) (C_2_=O, *Z*-isomer), 163.3 (NCH_2_*C*ONHOH, *E*-isomer), 168.8 (NCH_2_*C*ONHOH, *Z*-isomer), 176.7 (C_4_=O, *E*-isomer), 177.0 (low) (C_4_=O, *Z*-isomer); elemental analysis calcd (%) for C_16_H_19_N_3_O_4_: C 60.56, H 6.04, N 13.24, found: C 60.60, H 6.09, N 13.32.

*N*-Hydroxy-2-(2,4-dioxo-6-phenyl-1,3-diazaspiro [4.5]decan-3-yl) acetamide (37). The *N-*benzyloxy precursor 28 (120 mg, 0.29 mmol) was subjected to catalytic hydrogenation in a mixture of EtOH/AcOEt 3:1 (12 mL) according to the procedure described in the main manuscript for the synthesis of hydroxamate analogs. Concentration to dryness under reduced pressure afforded the title compound **37** as a white solid (90 mg, almost quantitative yield). Mp 196–199 °C (AcOEt/*n*-pentane), R*_f_* = 0.28 (AcOEt). This compound appeared in the ^1^H and ^13^C NMR spectra as a mixture of *E*/*Z* conformers. ^1^H NMR (600.11 MHz, DMSO-*d_6_*) *δ* (ppm): 1.46 (qt, 1H, *J_1_* = 12.7 Hz, *J_2_* = 3.2 Hz, H_8_), 1.56 (tt, 1H, *J_1_* = 14.8 Hz, *J_2_* = 3.9 Hz, H_9_), 1.62 (dq, 1H, *J_1_* = 17.6 Hz, *J_2_* = 4.0 Hz, H_7_), 1.67–1.76 (m, 2H, H_9_, H_10_), 1.80 (d, 1H, *J* = 12.3 Hz, H_8_), 1.86 (td, 1H, *J_1_* = 13.6 Hz, *J_2_* = 4.2 Hz, H_10_), 2.01 (qd, 1H, *J_1_* = 13.1 Hz, *J_2_* = 3.6 Hz, H_7_), 2.96 (dd, 1H, *J_1_* = 13.4 Hz, *J_2_* = 3.6 Hz, H_6_), 3.29–3.43 (q, AB, 1.6H, *J_AB_* = 15.9 Hz, NC*H_2_*CO, *E*-isomer), 3.59–3.75 (q, AB, 0.4H, *J_AB_* = 17.2 Hz, NC*H_2_*CO, *Z*-isomer), 7.08–7.13 (m, 2H, H_2′_, H_6′_), 7.16–7.24 (complex m, 3H, H_3′_, H_4′_, H_5′_), 8.86 (s, 0.6H, CH_2_CONHO*H*, *E*-isomer), 8.89 (s, 0.3H, H_1_, *Z*-isomer), 8.92 (s, 0.7H, H_1_, *E*-isomer), 9.16 (s, 0.2H, CH_2_CONHO*H*, *Z*-isomer), 10.16 (s, 0.2H, CH_2_CON*H*OH, *Z*-isomer), 10.43 (s, 0.6H, CH_2_CON*H*OH, *E*-isomer); ^13^C NMR (150.9 MHz, DMSO-*d_6_*) *δ* (ppm): 20.6 (C_9_), 25.1 (C_8_), 26.9 (C_7_), 34.2 (C_10_, *E*-isomer), 34.4 (C_10_, *Z*-isomer), 37.3 (N*C*H_2_CO, *E*-isomer), 37.5 (N*C*H_2_CO, *Z*-isomer), 47.5 (C_6_), 65.6 (C_5_), 127.0 (C_4′_), 127.7 (C_3′_, C_5′_), 128.3 (C_2′_, C_6′_), 139.6 (C_1′_), 155.4 (C_2_=O, *E*-isomer), 155.6 (C_2_=O, *Z*-isomer), 163.0 (NCH_2_*C*O, *E*-isomer), 168.5 (NCH_2_*C*O, *Z*-isomer), 175.4 (C_4_=O, *E*-isomer), 175.6 (C_4_=O, *Z*-isomer). Anal. Calcd for C_16_H_19_N_3_O_4_: C, 60.56; H, 6.04; N, 13.24. Found: C, 60.58; H, 6.08; N, 13.25.

*N*-Hydroxy-2-(1-methyl-2,4-dioxo-8-phenyl-1,3-diazaspiro [4.5]decan-3-yl)acetamide (38). A mixture of the *O*-benzyl hydroxamate 29 (200 mg, 0.47 mmol) and 10% Pd on charcoal (24 mg) in EtOH/AcOEt 3:1 (18.8 mL) was hydrogenated following the general procedure for the preparation of acetohydroxamic acids previously described to afford the title compound **38** as a white crystalline solid. (155 mg, almost quantitative yield); *R*_f_ = 0.50 (AcOEt); mp 197–200 °C (MeOH/dry Et_2_O-*n*-pentane); ^1^H NMR (400.13 MHz, [D_6_]DMSO): *δ* (ppm) = 1.75 (d, *J* = 9.9 Hz, 4H, H_6_, H_7_, H_9_, H_10_), 2.00 (dt, *J_1_* = 12.7 Hz, *J_2_* = 6.7 Hz, 2H, H_6_, H_10_), 2.16 (qd, *J_1_* = 13.2 Hz, *J_2_* = 3.8 Hz, 2H, H_7_, H_9_), 2.61 (t, *J* = 12.1 Hz, 1H, H_8_), 2.81 (s, 3H, NC*H_3_*), 3.91, 3.94 (s + s, 1.5H, NC*H_2_*CO, *E*-isomer), 4.20 (s, 0.45H, NC*H_2_*CO, *Z*-isomer), 7.19 (t, *J* = 7.1 Hz, 1H, H_4′_), 7.24 (d, *J* = 7.5 Hz, 2H, H_2′_, H_6′_), 7.31 (t, *J* = 7.5 Hz, 2H, H_3′_, H_5′_), 8.96 (s, 0.69H, ΝCH_2_CONHO*H*, *E*-isomer), 9.35 (s, 0.19H, ΝCH_2_CONHO*H*, *Z*-isomer), 10.31 (s, 0.19H, ΝCH_2_CON*H*OH, *Z*-isomer), 10.72 (s, 0.69H, ΝCH_2_CON*H*OH, *E*-isomer); ^13^C NMR (150.9 MHz, [D_6_]DMSO): *δ* (ppm) = 23.6 (*C*H_3_), 28.6 (C_7_, C_9_), 30.0 (C_6_, C_10_), 38.1 (N*C*H_2_CO, *E*-isomer), 38.3 (N*C*H_2_CO, *Z*-isomer), 41.4 (C_8_), 61.2 (C_5_), 126.1 (C_4′_), 126.6 (C_2′_, C_6′_), 128.4 (C_3′_, C_5′_), 146.4 (C_1′_), 154.4 (C_2_=O, *E*-isomer), 154.6 (C_2_=O, *Z*-isomer), 163.3 (NCH_2_*C*ONHOH, *E*-isomer), 168.7 (NCH_2_*C*ONHOH, *Z*-isomer), 175.4 (C_4_=O, *E*-isomer), 175.7 (C_4_=O, *Z*-isomer); elemental analysis calcd (%) for C_17_H_21_N_3_O_4_: C 61.62, H 6.39, N 12.68, found: C 61.64, H 6.47, N 12.64.

*N*-Hydroxy-2-(1-methyl-2,4-dioxo-6-phenyl-1,3-diazaspiro [4.5]decan-3-yl)acetamide (39). A solution of the *O*-benzyl hydroxamate 30 (85 mg, 0.20 mmol) in EtOH/AcOEt 3:1 (8 mL) was hydrogenated as described in the main manuscript. Evaporation of the solvents in vacuo yielded the title compound **39** as a glass solid, which is crystallized with *n*-pentane/Et_2_O at 0 °C to a white solid (62 mg, almost quantitative yield). Mp 195–197 °C (AcOEt, EtOH/*n*-pentane), R*_f_* = 0.49 (AcOEt). This compound exhibited distinct peaks attributed to each of the two *E*/*Z* conformers in the ^1^H and ^13^C NMR spectra. ^1^H NMR (600.11 MHz, MeOD) *δ* (ppm): 1.66 (1H, *J_1_* = 12.9 Hz, *J_2_* = 4.3 Hz, H_8_), 1. 85 (dq, 1H, *J_1_* = 13.5 Hz, *J_2_* = 3.6 Hz, H_7_), 1.89–2.00 (complex m, 2H, H_9_), 2.07–2.15 (m, 2H, H_8_, H_10_), 2.18 (dd, 1H, *J_1_* = 13.3 Hz, *J_2_* = 6.4 Hz, H_10_), 2.24 (qd, 1H, *J_1_* = 13.6 Hz, *J_2_* = 3.6 Hz), 3.16 (dd, 1H, J = 13.9, 4.1 Hz), 3.34 (s, 3H, NC*H_3_*), 3.50–3.74 (q, AB, 1.6H, *J_AB_* = 16.0 Hz, NC*H_2_*CO, *E*/*Z*-isomer), 3.85–4.10 (q, AB, 0.4H, *J_AB_* = 17.4 Hz, NC*H_2_*CO, *E*/*Z*-isomer), 4.83 (s, 2H, CH_2_COΝ*H*O*H*, under MeOD-water peak), 7.08–7.14 (m, 2H, H_2′_, H_6′_), 7.18–7.27 (m, 3H, H_3′_, H_4′_, H_5′_); ^13^C NMR (150.9 MHz, MeOD) *δ* (ppm): 22.8 (C_9_), 26.5 (C_8_), 28.7 (C_7_), 30.8 (N*C*H_3_), 33.0 (C_10_, *E*/*Z*-isomer), 33.3 (C_10_, *E*/*Z*-isomer), 39.5 (N*C*H_2_CO, *E*/*Z*-isomer), 39.6 (N*C*H_2_CO, *E*/*Z*-isomer), 51.1 (C_6_, *E*/*Z*-isomer), 51.2 (C_6_, *E*/*Z*-isomer), 69.2 (C_5_), 128.5 (C_4′_), 129.2 (C_2′_, C_6′_), 129.3 (C_3′_, C_5′_), 140.6 (C_1′_), 157.2 (C_2_=O), 165.8 (NCH_2_*C*O, *E*/*Z*-isomer), 169.2 (NCH_2_*C*O, *E*/*Z*-isomer), 177.3 (C_4_=O). Anal. Calcd for C_17_H_21_N_3_O_4_: C, 61.62; H, 6.39; N, 12.68. Found: C, 61.67; H, 6.43; N, 12.66.

*N*-Hydroxy-2-(1-ethyl-2,4-dioxo-8-phenyl-1,3-diazaspiro [4.5]decan-3-yl)acetamide (40). A total of 10 wt% Pd (23 mg) on charcoal was added to a solution of the *O*-benzyl hydroxamate 31 (190 mg, 0.44 mmol) in a mixture of EtOH/AcOEt 3:1 (17.6 mL), and the mixture was hydrogenated following the procedure described for the synthesis of hydroxamate analogs. The white foamy solid obtained strongly binds the aforementioned solvents. Removal of the entrapped solvents upon drying under high vacuum yielded the target compound **40** as a glass solid, which was crystallized upon treatment under ice cooling (150 mg, almost quantitative yield); *R*_f_ = 0.25 (AcOEt); mp 147–150 °C (AcOEt/dry Et_2_O-*n*-pentane); ^1^H NMR (600.11 MHz, [D_6_]DMSO): *δ* (ppm) = 1.16 (t, *J* = 7.1 Hz, 3H, NCH_2_C*H_3_*), 1.75 (dq, *J_1_* = 7.8 Hz, *J_2_* = 3.8 Hz, 0.4H, H_6_, H_10_, *trans*), 1.81–1.98 (m, 5.6H, H_6_, H_10_, *cis*, H_7_, H_9_), 2.04 (dd, *J_1_* = 8.6 Hz, *J_2_* = 4.0 Hz, 1.8H, H_7_, H_9_, *cis*), 2.18 (qd, *J_1_* = 13.1 Hz, *J_2_* = 2.5 Hz, 0.2H, H_7_, H_9_, *trans*), 2.65 (tt, *J_1_* = 12.6 Hz, *J_2_* = 3.6 Hz, 0.1H, H_8_, *trans*), 2.81 (dt, *J_1_* = 9.4 Hz, *J_2_* = 4.9 Hz, 0.9H, H_8_, *cis*), 3.53 (q, *J* = 6.9 Hz, 2H, NC*H_2_*CH_3_), 3.91, 3.95 (s + s, 1.55H, NC*H_2_*CO, *E*-isomer), 4.21 (s, 0.45H, NC*H_2_*CO_,_ *Z*-isomer), 7.19 (tt, *J_1_* = 6.8 Hz, *J_2_* = 1.4 Hz, 0.1H, H_4′_, *trans*), 7.21 (tt, *J_1_* = 6.5 Hz, *J_2_* = 2.0 Hz, 0.9H, H_4′_, *cis*), 7.24 (dd, *J_1_* = 8.1 Hz, *J_2_* = 1.5 Hz, 0.2H, H_2′_, H_6′_, *trans*), 7.31 (td, *J_1_* = 7.5 Hz, *J_2_* = 1.9 Hz, 0.2H, H_3′_, H_5′_, *trans*), 7.31–7.37 (m, 3.8H, H_2′_, H_6′_, *cis*, H_3′_, H_5′_, *cis*), 8.93 (s, 0.6H, ΝCH_2_CONHO*H*, *E*-isomer), 9.31 (s, 0.2H, ΝCH_2_CONHO*H*, *Z*-isomer), 10.26 (s, 0.2H, ΝCH_2_CON*H*OH, *Z*-isomer), 10.66 (br s, 0.4H, ΝCH_2_CON*H*OH, *E*-isomer); ^13^C NMR (150.9 MHz, [D_6_]DMSO): *δ* (ppm) = 14.6 (NCH_2_*C*H_3_), 27.6 (C_7_, C_9_, *cis*), 28.6 (C_7_, C_9_, *trans*), 31.6 (C_6_, C_10_, *cis*), 31.7 (C_6_, C_10_, *trans*), 36.3 (N*C*H_2_CH_3_), 38.4 (N*C*H_2_CO, *E*-isomer), 38.7 (N*C*H_2_CO, *Z*-isomer), 39.4 (C_8_), 62.3 (C_5_), 126.0 (C_4′_), 126.6 (C_2′_, C_6′_, *trans*), 126.8 (C_2′_, C_6′_, *cis*), 128.4 (C_3′_, C_5′_), 145.2 (C_1′_), 154.3 (C_2_=O, *E*-isomer), 154.5 (C_2_=O, *Z*-isomer), 163.1 (NCH_2_*C*ONHOH, *E*-isomer), 168.6 (NCH_2_*C*ONHOH, *Z*-isomer), 175.9 (C_4_=O, *E*-isomer), 176.2 (C_4_=O, *Z*-isomer); elemental analysis calcd (%) for C_18_H_23_N_3_O_4_: C 62.59, H 6.71, N 12.17, found: C 62.54, H 6.78, N 12.23.

*N*-Hydroxy-2-(1-benzyl-2,4-dioxo-8-phenyl-1,3-diazaspiro [4.5]decan-3-yl)acetamide (41). A mixture of the *O*-benzyl hydroxamate 32 (85 mg, 0.17 mmol) and 10% Pd on charcoal (10 mg) in EtOH/AcOEt 3:1 (7 mL) was hydrogenated following the general procedure for the synthesis of acetohydroxamic acids previously described to afford the title compound **41** a white foamy solid, which strongly binds the aforementioned solvents. Removal of the entrapped solvents upon drying under high vacuum yielded the desired compound **41** as a glass solid, which was crystallized upon treatment with *n*-pentane under ice cooling. (69 mg, almost quantitative yield); *R*_f_ = 0.48 (AcOEt); mp 105–107 °C (AcOEt/*n*-pentane); ^1^H NMR (400.13 MHz, [D_6_]Acetone): *δ* (ppm) = 1.60–2.07 (complex m, 7.3H, H_6_, H_7_, H_9_, H_10_), 2.25 (qd, *J_1_* = 13.8 Hz, *J_2_* = 3.6 Hz, 0.3H, H_7_, H_9_, *cis*), 2.50 (td, *J_1_* = 11.8 Hz, *J_2_* = 6.1 Hz, 0.15H, H_8_, *cis*), 2.66 (td, *J_1_* = 10.0 Hz, *J_2_* = 4.0 Hz, 0.7H, H_8_, *trans*), 4.11, 4.17 (s + s, 1.2H, NC*H_2_*CO, *E*-isomer), 4.42 (s, 0.55H, NC*H_2_*CO, *Z*-isomer), 4.59 (s, 0.35H, NC*H_2_*Ph), 4.82 (s, 1.5H, NC*H_2_*Ph), 7.06–7.40 (complex m, 10H, H_Ar_), 8.18 (s, 0.25H, ΝCH_2_CONHO*H*, *E*-isomer), 9.68 (s, 0.12H, ΝCH_2_CONHO*H*, *Z*-isomer), 9.50 (s, 0.15H, ΝCH_2_CON*H*OH, *Z*-isomer), 10.15 (s, 0.25H, ΝCH_2_CON*H*OH, *E*-isomer); ^13^C NMR (50.32 MHz, [D_6_]Acetone): *δ* (ppm) = 29.1 (C_7_, C_9_), 32.7, 32.8 (C_6_, C_10_), 39.7 (N*C*H_2_CO, *E*-isomer), 40.0 (N*C*H_2_CO, *Z*-isomer), 41.0 (C_8_, *trans*), 42.4 (N*C*H_2_Ph), 43.2 (C_8_, *cis*), 45.3 (N*C*H_2_Ph), 63.9, 64.4 (C_5_), 126.9 (C_4′_), 127.6 (C_2′_, C_6′_), 127.8 (C_2′_, C_6′_, C_2Bz_, C_6Bz_), 128.0 (C_4Bz_), 128.1 (C_2Bz_, C_6Bz_), 129.2 (C_3′_, C_5′_), 129.4 (C_3Bz_, C_5Bz_), 139.7, 139.9 (C_1Bz_), 146.4, 147.5 (C_1′_), 157.1 (C_2_=O), 164.8 (NCH_2_*C*ONHOH, *E*-isomer), 170.7 (NCH_2_*C*ONHOH, *Z*-isomer), 177.1 (C_4_=O); elemental analysis calcd (%) for C_23_H_25_N_3_O_4_: C 67.80, H 6.18; N 10.31, found: C 67.85, H 6.15; N 10.33.

*N*-Hydroxy-2-(1-benzyl-2,4-dioxo-6-phenyl-1,3-diazaspiro [4.5] decan-3-yl)acetamide (42). A solution of the *O*-benzyl hydroxamate 33 (100 mg, 0.20 mmol) in EtOH/AcOEt 3:1 (8 mL) was hydrogenated in the presence of Pd/C (12 mg) as described for the preparation of *N*-(hydroxy)acetamides. Concentration to dryness yielded acetohydroxamic acid **42** as a colorless glass solid. Treatment with *n*-pentane/Et_2_O at 0 °C afforded a white crystalline solid (78 mg, almost quantitative yield). Mp 110–111 °C (AcOEt/*n*-pentane), R*_f_* = 0.64 (AcOEt). Ιn the ^1^H and ^13^C NMR spectra of this compound, a double set of characteristic peaks are distinguished for each of the two *E*/*Z* conformers. ^1^H NMR (600.11 MHz, DMSO-*d_6_*) *δ* (ppm): 1.26 (qt, 1H, *J_1_* = 14.1 Hz, *J_2_* = 3.2 Hz, H_9_), 1.36–1.44 (m, 1H, H_9_), 1.49 (qt, 1H, *J_1_* = 13.1 Hz, *J_2_* = 3.9 Hz, H_8_), 1.69–1.79 (m, 2H, H_7_, H_10_), 1.85–1.91 (m, 1H, H_8_), 1.94 (td, 1H, *J_1_* = 14.2 Hz, *J_2_* = 4.7 Hz, H_10_), 2.37 (qd, 1H, *J_1_* = 12.5 Hz, *J_2_* = 3.8 Hz, H_7_), 3.09 (dd, 1H, *J_1_* = 14.1 Hz, *J_2_* = 4.2 Hz, H_6_), 3.34 (brs, 2H, CH_2_COΝ*H*O*H*, under DMSO-water peak), 3.41–3.71 (q, AB, 1.6H, *J_AB_* = 15.9 Hz, NC*H_2_*CO, *E*-isomer), 3.71–4.00 (q, AB, 0.4H, *J_AB_* = 17.2 Hz, NC*H_2_*CO, *Z*-isomer), 4.92–5.11 (q, AB, 2H, *J_AB_* = 17.3 Hz, NC*H_2_*Ph), 7.08–7.18 (m, 2H, H_2′_, H_6′_), 7.19–7.43 (complex m, 8H, H_3′_, H_4′_, H_5′_, H_2Bz_, H_3Bz_, H_4Bz_, H_5Bz_, H_6Bz_); ^13^C NMR (100.61 ΜHz, DMSO-*d_6_*) *δ* (ppm): 20.1 (C_9_), 24.7 (C_8_), 26.9 (C_7_), 31.7 (C_10_, *E*-isomer), 31.9 (C_10_, *Z*-isomer), 38.1 (N*C*H_2_CO), 45.7 (N*C*H_2_Ph), 49.6 (C_6_), 67.7 (C_5_), 126.2 (C_2Bz_, C_6Bz_), 126.9 (C_4Bz_), 127.3 (C_4′_), 128.1 (C_2′_, C_6′_, C_3Bz_, C_5Bz_), 128.3 (C_3′_, C_5′_), 138.0 (C_1Bz_), 139.2 (C_1′_), 155.5 (C_2_=O, *E*-isomer), 155.7 (C_2_=O, *Z*-isomer), 162.8 (NCH_2_*C*O, *E*-isomer), 168.2 (NCH_2_*C*O, *Z*-isomer), 175.2 (C_4_=O, *E*-isomer), 175.3 (C_4_=O, *Z*-isomer). Anal. Calcd for C_23_H_25_N_3_O_4_: C, 67.80; H, 6.18; N, 10.31. Found: C, 67.89; H, 6.24; N, 10.35.

*N*-(Hydroxy)-2-(2,4-dioxo-8-phenyl-1,3-diazaspiro [4.5]decan-3-yl)propanamide (49). The *N-*benzyloxy precursor 48 (200 mg, 0.47 mmol) was subjected to catalytic hydrogenation in a mixture of EtOH/AcOEt 3:1 (19 mL) according to the procedure described for the synthesis of hydroxamate analogs to afford the title compound **49** as a white crystalline solid. (155 mg, almost quantitative yield); *R*_f_ = 0.34 (AcOEt); mp 184–186 °C (MeOH/*n*-pentane); ^1^H NMR (600.11 MHz, [D_6_]DMSO): *δ* (ppm) = 1.43 (d, *J* = 7.1 Hz, 0.1H, NCH(C*H_3_*)CO), 1.49 (d, *J* = 7.3 Hz, 2.8H, NCH(C*H_3_*)CO, 1.66–1.88 (complex m, 7.8H, H_6_, H_7_, H_9_, H_10_), 2.11 (q, *J_1_* = 12.0 Hz, 0.15H, H_7_, H_9_), 2.57 (tt, *J_1_* = 11.0 Hz, *J_2_* = 5.8 Hz, 1H, H_8_), 4.42 (q, *J* = 7.4 Hz, 0.1H, NC*H*(CH_3_)CO), 4.45 (q, *J* = 7.4 Hz, 0.8H, NC*H*(CH_3_)CO), 7.18 (td, *J_1_* = 7.1 Hz, *J_2_* = 1.4 Hz, 1H, H_4′_), 7.25 (d, *J* = 7.6 Hz, 0.2H, H_2′_, H_6′_), 7.29 (t, *J* = 7.5 Hz, 2H, H_3′_, H_5′_), 7.33 (d, *J* = 7.6 Hz, 1.8H, H_2′_, H_6′_), 8.18 (s, 0.05H, H_1_), 8.81 (s, 0.8H, NCH(CH_3_)CONHO*H*, *E*-isomer), 8.95, 9.02 (s + s, 0.85H, H_1_), 9.16 (s, 0.05H, NCH(CH_3_)CONHO*H*, *Z*-isomer), 10.00 (s, 0.05H, NCH(CH_3_)CON*H*OH, *Z*-isomer), 10.60 (s, 0.75H, NCH(CH_3_)CON*H*OH, *E*-isomer); ^13^C NMR (150.9 MHz, [D_6_]DMSO): *δ* (ppm) = 15.1 (NCH(*C*H_3_)CO), 28.9 (C_7_, C_9_), 33.7 (C_6_, C_10_), 42.7 (C_8_), 47.4 (N*C*H(CH_3_)CO), 60.8 (C_5_), 126.5 (C_4′_), 127.4 (C_2′_, C_6′_), 128.7 (C_3′_, C_5′_), 147.1 (C_1′_), 155.8 (C_2_=O), 166.4 (NCH(CH_3_)*C*ONHOH, *E*-isomer), 168.7 (NCH(CH_3_)*C*ONHOH, *Z*-isomer), 177.1 (C_4_=O); elemental analysis calcd (%) for C_17_H_21_N_3_O_4_: C 61.62, H 6.39, N 12.68, found: C 61.62, H 6.39, N 12.68.

*N*-(hydroxy)-2-(1-methyl-2,4-dioxo-8-phenyl-1,3-diazaspiro [4.5]decan-3-yl)propanamide (53). A mixture of the *O*-benzyl hydroxamate 52 (350 mg, 0.80 mmol) and 10% Pd on charcoal (42 mg) in EtOH/AcOEt 3:1 (32 mL) was hydrogenated following the general procedure for the preparation of acetohydroxamic acids previously described to afford the title compound **53** as a white foamy solid, which strongly binds the aforementioned solvents. Removal of the entrapped solvents upon drying under high vacuum yielded **53** as a glass solid. Purification of this by column chromatography on silica gel using CH_2_Cl_2_/AcOEt 4:1 and AcOEt provided the pure hydroxamic acid as a white crystalline solid. (275 mg, almost quantitative yield); *R*_f_ = 0.37 (AcOEt); mp 143–145 °C (MeOH/*n*-pentane); ^1^H NMR (600.11 MHz, [D_6_]DMSO): *δ* (ppm) = 1.45 (d, *J* = 7.2 Hz, 0.1H, NCH(C*H_3_*)CO), 1.49 (d, *J* = 7.4 Hz, 2.9H, NCH(C*H_3_*)CO), 1.70–1.82 (complex m, 4H, H_6_, H_7_, H_9_, H_10_), 1.96 (tt, *J_1_* = 12.9 Hz, *J_2_* = 3.9 Hz, 2H, H_6_, H_10_), 2.17 (qd, *J_1_* = 12.3 Hz, *J_2_* = 2.6 Hz, 2H, H_7_, H_9_), 2.59 (tt, *J_1_* = 12.3 Hz, *J_2_* = 3.5 Hz, 1H, H_8_), 2.80 (s, 3H, NC*H_3_*), 4.45 (q, *J* = 7.4 Hz, 0.05H, NC*H*(CH_3_)CO), 4.49 (q, *J* = 7.2 Hz, 0.8H, NC*H*(CH_3_)CO), 7.19 (td, *J_1_* = 7.3 Hz, *J_2_* = 1.5 Hz, 1H, H_4′_), 7.24 (dd, *J_1_* = 7.6 Hz, *J_2_* = 1.6 Hz, 2H, H_2′_, H_6′_), 7.31 (td, *J_1_* = 7.6 Hz, *J_2_* = 1.8 Hz, 2H, H_3′_, H_5′_), 8.85 (s, 0.8H, NCH(CH_3_)CONHO*H*, *E*-isomer), 9.20 (s, 0.05H, NCH(CH_3_)CONHO*H*, *Z*-isomer), 10.05 (s, 0.05H, NCH(CH_3_)CON*H*OH, *Z*-isomer), 10.64 (s, 0.8H, NCH(CH_3_)CON*H*OH, *E*-isomer); ^13^C NMR (50.32 MHz, [D_6_]DMSO): *δ* (ppm) = 14.6 (NCH(*C*H_3_)CO), 23.6 (N*C*H_3_), 28.6, 28.7 (C_7_, C_9_), 29.9 (C_6_, C_10_), 41.6 (C_8_), 47.2 (N*C*H(CH_3_)CO), 60.6 (C_5_), 126.1 (C_4′_), 126.7 (C_2′_, C_6′_), 128.5 (C_3′_, C_5′_), 146.5 (C_1′_), 154.4 (C_2_=O), 166.0 (NCH(CH_3_)*C*ONHOH, *E*-isomer), 170.4 (NCH(CH_3_)*C*ONHOH, *Z*-isomer), 175.4 (C_4_=O); elemental analysis calcd (%) for C_18_H_23_N_3_O_4_: C 62.59, H 6.71, N 12.17, found: C 62.66, H 6.74, N 12.11.

## 4. Conclusions

The novel bicyclic-substituted hydantoin analogs not only demonstrated significantly improved antiviral and antiparasitic activity when compared to lead analog **V,** but also exceptional inhibitory activity against different HCV genotypes (1b, 3a, 4a) and DENV, as well as two trypanosome species (*T. brucei*, *T. cruzi*). According to the structure–activity relationships established from the biological studies, the major conclusions can be summarized as follows: (i) the increased flexibility of the new compounds improved the attainment of particularly favorable aromatic interactions, resulting in higher activity and selectivity compared to the more constrained annulated counterparts with the planar shape and lower conformational degrees of freedom. (ii) Furthermore, enhancing the lipophilicity of the compounds, notably by introducing a methyl, ethyl, or benzyl substituent at the amide nitrogen atom of the hydantoin ring, significantly increased the activity. (iii) Unlike lipophilicity, the stereochemistry of the tetralin backbone does not seem to play a critical role in either antiviral or trypanocidal activity, except for anti-HCV (genotype 1b) activity, where compound **34** exhibited eight-times-better potency in comparison with **35**. (iv) Finally, the selectivity indices observed for most of the tested analogs are remarkable against all HCV genotypes tested and *T. brucei*, suggesting that the novel scaffold of an appropriately substituted hydantoin ring and a metal-binding moiety could be a successful strategy for producing multifunctional drugs and may be the key to producing therapeutics against a wide range of pathogens.

## Figures and Tables

**Table 1:** Activity of acetohydroxamic acid analogs **34**–**42**, **49**, **53,** and **V** (lead compound) tested against cultured bloodstream form *T*. *brucei* and *T*. *cruzi* epimastigotes. Cytotoxicity against cultured rat skeletal myoblast L6 cells and selectivity indices.

Compound	Structure	*T. brucei* ΕC_50_ (μM) ^1^	*T. brucei* ΕC_90_ (μM) ^1^	SI ^3^	*T. cruzi* ΕC_50_ (μM) ^1^	*T. cruzi* ΕC_90_ (μM) ^1^	SI ^3^	L6 Cells CC_50_ (μM) ^2^
**34**	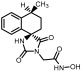	0.67 ± 0.09	0.92 ± 0.23	335	>30	-	-	224 ± 20
**35**	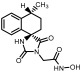	0.56 ± 0.07	0.86 ± 0.19	400	>30	-	-	224 ± 7
**36**	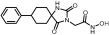	0.88 ± 0.10	1.18 ± 0.04	70	>30	-	-	60.8 ± 8.2
**38**	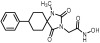	0.10 ± 0.01	0.14 ± 0.01	220	6.25 ± 0.24	13.3 ± 0.3	4	21.7 ± 3.9
**40**	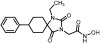	0.029 ± 0.001	0.055 ± 0.003	1870	1.12 ± 0.03	1.86 ± 0.04	48	54.3 ± 5.9
**41**	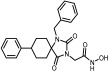	0.009 ± 0.001	0.011 ± 0.001	440	0.34 ± 0.02	0.74 ± 0.05	12	3.98 ± 0.32
**49**	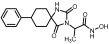	0.876 ± 0.051	1.42 ± 0.19	270	>30	-	-	233 ± 33
**53**	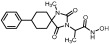	0.154 ± 0.009	0.281 ± 0.006	590	8.50 ± 0.23	11.9 ± 0.5	11	90.5 ± 7.3
**37**	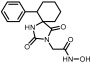	4.41 ± 0.22	5.92 ± 0.09	55	>30	-	-	246 ± 3
**39**	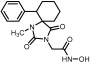	1.46 ± 0.06	1.75 ± 0.02	150	>30	-	-	217 ± 6
**42**	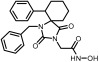	0.191 ± 0.006	0.237 ± 0.003	62	1.95 ± 0.05	3.12 ± 0.12	6	11.9 ± 0.7
**V**	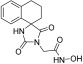	0.71 ± 0.12	1.27 ± 0.02	590	>30	>30	-	419 ± 55

^1^ Concentrations required to inhibit growth of *T. brucei* and *T. cruzi* by 50% and 90%, respectively. EC_50_ and EC_90_ data are the mean of triplicate experiments ± SEM. ^2^ Cytotoxicity was determined by establishing the concentration required to inhibit growth of cultured L6 cells by 50% (CC_50_). Data are the means of triplicate experiments ± SEM. ^3^ Selectivity indices were calculated as the ratio of the CC_50_ for L6 cells to EC_50_ for *T. brucei* or *T. cruzi*, respectively.

**Table 2:** Activity of acetohydroxamic acid analogs **34**–**42**, **49**, **53,** and **V** (lead compound) in replicon cell lines of HCV GT 1b (Con1), HCV GT 3a (S52), HCV GT 4a (ED43), DENV-2, and YFV-17D. Cytotoxicity experiments were performed on the HCV GT 1b replicon stable cell line (Huh5.2 cells).

		Huh5-2 (HCV Replicon 1b)			Huh7.5-3a(S52) (HCV Replicon 3a)		Huh7.5-4a(ED43) (HCV Replicon 4a)		Huh7-YF(YFV Replicon 17D)		Huh7-D2 (DENV-2 Replicon)	
Compound	Structure	CC_50_ (μΜ) ^3^	EC_50_ (μΜ) ^1^	SI ^2^	EC_50_ (μΜ) ^1^	SI ^2^	EC_50_ (μΜ) ^1^	SI ^2^	EC_50_ (μΜ) ^1^	SI ^2^	EC_50_ (μΜ) ^1^	SI ^2^
**34**	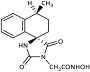	>200	0.98 ± 0.10	204	0.63 ± 0.08	318	0.14 ± 0.01	1429	>10	-	>10	-
**35**	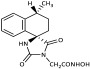	>200	7.31 ± 0.42	28	0.77 ± 0.03	260	0.41 ± 0.02	488	>10	-	>10	-
**36**	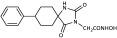	75.39 ± 8.46	2.53 ± 0.11	30	0.75 ± 0.13	101	0.39 ± 0.01	193	>10	-	4.64 ± 0.11	16
**38**	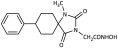	43.14 ± 2.11	2.75 ± 0.96	16	0.63 ± 0.01	69	0.22 ± 0.00	196	>10	-	10.23 ± 0.18	4
**40**	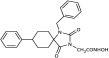	10.62 ±0.12	0.34 ± 0.06	31	0.52 ± 0.08	20	0.31 ± 0.13	34	6.67 ± 0.05	1.59	0.68 ± 0.03	15.62
**41**	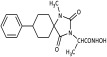	>10 *	>10	1	>10	-	>10	-	>10	-	>10	-
**49**	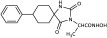	>10 *	>10	-	>10	-	>10	-	7.98 ± 0.18	1	>10	-
**53**	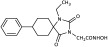	100.2 ± 3.57	8.66 ± 0.76	12	>10	-	0.28 ± 0.02	358	>10	-	2.45 ± 0.22	41
**37**	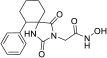	>10 *	>10		>10	-	>10	-	>10	-	>10	-
**39**	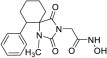	>10 *	>10		>10	-	>10	-	>10	-	4.62 ± 0.39	2
**42**	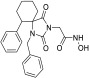	9.37 ± 0.182	0.74 ± 0.02	13	>10	-	0.45 ± 0.02	21	4.68 ± 0.23	2	1.37 ± 0.08	7
**V**	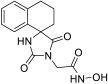	>200	1.41 ± 0.35	>142	2.37 ± 0.17	>84	0.38 ± 0.04	>526	-	-	61.49 ± 3.86	>3

^1^ EC_50_ (median effective concentration) values represent the mean ± SD of three independent experiments, each carried out in triplicate. Calculated against HCV genotype 1b (Huh5-2, HCV 1b replicon cells), genotype 3a (Huh7.5-3a, HCV 3a replicon cells), genotype 4a (Huh7.5-4a, HCV 4a replicon cells). ^2^ Selectivity index: CC_50_/EC_50_. ^3^ CC_50_ (median cytotoxic concentration against Huh5-2, HCV replicon cells) values represent the mean ± SD of three independent experiments, each carried out in triplicate. * Compounds with EC_50_ > 10 μΜ were considered non-effective, and for this, the higher concentration used in the cytotoxicity experiments was 10 μΜ.

## Data Availability

All relevant data are within the manuscript.

## Supplementary Materials

The following supporting information can be downloaded at: https://www.mdpi.com/article/10.3390/ph16071046/s1, List of Contents: I. ^1^H NMR and ^13^C NMR spectra of 4-phenyl-cyclohexane substituted analogue **9** (page 3, SM); II. Experimental procedures for the biological evaluation of the compounds (page 8, SM); IIa. Anti-HCV Activity (page 8, SM); IIb. Trypanocidal Activity (page 9, SM); III. References (page 10, SM); IV. Copies of NMR spectra (page 11, SM).

## References

[1] Hotez P.J., Pecoul B., Rijal S., Boehme C., Aksoy S., Malecela M., Tapia-Conyer R., Reeder J.C. (2016). Eliminating the Neglected Tropical Diseases: Translational Science and New Technologies. PLoS Negl. Trop. Dis..

[2] Hotez P.J., Aksoy S., Brindley P.J., Kamhawi S. (2020). What Constitutes a Neglected Tropical Disease?. PLoS Negl. Trop. Dis..

[3] Gao J.-M., Qian Z.-Y., Hide G., Lai D.-H., Lun Z.-R., Wu Z.-D. (2020). Human African Trypanosomiasis: The Current Situation in Endemic Regions and the Risks for Non-Endemic Regions from Imported Cases. Parasitology.

[4] De Koning H.P. (2020). The Drugs of Sleeping Sickness: Their Mechanisms of Action and Resistance, and a Brief History. Trop. Med. Infect. Dis..

[5] Dickie E.A., Giordani F., Gould M.K., Mäser P., Burri C., Mottram J.C., Rao S.P.S., Barrett M.P. (2020). New Drugs for Human African Trypanosomiasis: A Twenty First Century Success Story. Trop. Med. Infect. Dis..

[6] Deeks E.D. (2019). Fexinidazole: First Global Approval. Drugs.

[7] Giordani F., Paape D., Vincent I.M., Pountain A.W., Fernández-Cortés F., Rico E., Zhang N., Morrison L.J., Freund Y., Witty M.J. (2020). Veterinary Trypanocidal Benzoxaboroles Are Peptidase-Activated Prodrugs. PLoS Pathog..

[8] Moreno-Herrera A., Cortez-Maya S., Bocanegra-Garcia V., Banik B.K., Rivera G. (2021). Recent Advances in the Development of Broad-Spectrum Antiprotozoal Agents. Curr. Med. Chem..

[9] Guarner J. (2019). Chagas Disease as Example of a Reemerging Parasite. Semin. Diagn. Pathol..

[10] Gascon J., Pinazo M.-J. (2015). Chagas Disease: From Latin America to the World. Rep. Parasitol..

[11] Pavan T.B.S., da Silva J.W., Martins L.C., Costa S.C.B., de Almeida E.A. (2018). Hepatic Changes by Benznidazole in a Specific Treatment for Chagas Disease. PLoS ONE.

[12] Kratz J.M. (2019). Drug Discovery for Chagas Disease: A Viewpoint. Acta Trop..

[13] World Health Organization (2021). Hepatitis C. http://www.who.int/En/News-Room/Fact-Sheets/Detail/Hepatitis-c.

[14] Pierson T.C., Diamond M.S. (2020). The Continued Threat of Emerging Flaviviruses. Nat. Microbiol..

[15] Stanaway J.D., Shepard D.S., Undurraga E.A., Halasa Y.A., Coffeng L.E., Brady O.J., Hay S.I., Bedi N., Bensenor I.M., Castañeda-Orjuela C.A. (2016). The Global Burden of Dengue: An Analysis from the Global Burden of Disease Study 2013. Lancet Infect. Dis..

[16] Monath T.P., Vasconcelos P.F.C. (2015). Yellow Fever. J. Clin. Virol..

[17] World Health Organization (2021). Yellow Fever Virus. https://www.who.int/En/News-Room/Fact-Sheets/Detail/Yellow-Fever.

[18] World Health Organization (2021). Dengue Virus. https://www.who.int/En/News-Room/Fact-Sheets/Detail/Dengue-and-Severe-Dengue.

[19] Acosta E.G., Bartenschlager R. (2016). Paradoxical Role of Antibodies in Dengue Virus Infections: Considerations for Prophylactic Vaccine Development. Expert Rev. Vaccines.

[20] Bartenschlager R., Lohmann V., Penin F. (2013). The Molecular and Structural Basis of Advanced Antiviral Therapy for Hepatitis C Virus Infection. Nat. Rev. Microbiol..

[21] Moradpour D., Penin F., Rice C.M. (2007). Replication of Hepatitis C Virus. Nat. Rev. Microbiol..

[22] Bartenschlager R., Miller S. (2008). Molecular Aspects of Dengue Virus Replication. Future Microbiol..

[23] Diamond M.S., Pierson T.C. (2015). Molecular Insight into Dengue Virus Pathogenesis and Its Implications for Disease Control. Cell.

[24] Douam F., Ploss A. (2018). Yellow Fever Virus: Knowledge Gaps Impeding the Fight Against an Old Foe. Trends Microbiol..

[25] Soriano V., Peters M.G., Zeuzem S. (2009). New Therapies for Hepatitis C Virus Infection. Clin. Infect. Dis..

[26] Wakita T., Pietschmann T., Kato T., Date T., Miyamoto M., Zhao Z., Murthy K., Habermann A., Kräusslich H.-G., Mizokami M. (2005). Production of Infectious Hepatitis C Virus in Tissue Culture from a Cloned Viral Genome. Nat. Med..

[27] De Clercq E., Li G. (2016). Approved Antiviral Drugs over the Past 50 Years. Clin. Microbiol. Rev..

[28] Hepatitis C. https://www.hepatitisc.uw.edu/Page/Treatment/Drugs.

[29] Asselah T., Marcellin P., Schinazi R.F. (2018). Treatment of Hepatitis C Virus Infection with Direct-Acting Antiviral Agents: 100% Cure?. Liver Int..

[30] Cuypers L., Ceccherini-Silberstein F., Van Laethem K., Li G., Vandamme A.-M., Rockstroh J.K. (2016). Impact of HCV Genotype on Treatment Regimens and Drug Resistance: A Snapshot in Time: HCV Genotyping for Treatment and Drug Resistance. Rev. Med. Virol..

[31] Tsukiyama-Kohara K., Kohara M. (2017). Hepatitis C Virus: Viral Quasispecies and Genotypes. Int. J. Mol. Sci..

[32] Kliemann D.A., Tovo C.V., Gorini Da Veiga A.B., Machado A.L., West J. (2016). Genetic Barrier to Direct Acting Antivirals in HCV Sequences Deposited in the European Databank. PLoS ONE.

[33] Troost B., Smit J.M. (2020). Recent Advances in Antiviral Drug Development towards Dengue Virus. Curr. Opin. Virol..

[34] Julander J.G. (2013). Experimental Therapies for Yellow Fever. Antivir. Res..

[35] Zoidis G., Giannakopoulou E., Stevaert A., Frakolaki E., Myrianthopoulos V., Fytas G., Mavromara P., Mikros E., Bartenschlager R., Vassilaki N. (2016). Novel Indole–Flutimide Heterocycles with Activity against Influenza PA Endonuclease and Hepatitis C Virus. Med. Chem. Commun..

[36] Fytas C., Zoidis G., Tzoutzas N., Taylor M.C., Fytas G., Kelly J.M. (2011). Novel Lipophilic Acetohydroxamic Acid Derivatives Based on Conformationally Constrained Spiro Carbocyclic 2,6-Diketopiperazine Scaffolds with Potent Trypanocidal Activity. J. Med. Chem..

[37] Zoidis G., Tsotinis A., Tsatsaroni A., Taylor M.C., Kelly J.M., Efstathiou A., Smirlis D., Fytas G. (2018). Lipophilic Conformationally Constrained Spiro Carbocyclic 2,6-Diketopiperazine-1-Acetohydroxamic Acid Analogues as Trypanocidal and Leishmanicidal Agents: An Extended SAR Study. Chem. Biol. Drug Des..

[38] Giannakopoulou E., Pardali V., Frakolaki E., Siozos V., Myrianthopoulos V., Mikros E., Taylor M.C., Kelly J.M., Vassilaki N., Zoidis G. (2019). Scaffold Hybridization Strategy towards Potent Hydroxamate-Based Inhibitors of *Flaviviridae* Viruses and *Trypanosoma* Species. Med. Chem. Commun..

[39] Papanastasiou I., Tsotinis A., Zoidis G., Kolocouris N., Prathalingam S.R., Kelly J.M. (2009). Design and Synthesis of *Trypanosoma Brucei* Active 1-Alkyloxy and 1-Benzyloxyadamantano 2-Guanylhydrazones. ChemMedChem.

[40] Giannakopoulou E., Pardali V., Skrettas I., Zoidis G. (2019). Transesterification Instead of N-Alkylation: An Intriguing Reaction. ChemistrySelect.

[41] Tsatsaroni A., Zoidis G., Zoumpoulakis P., Tsotinis A., Taylor M.C., Kelly J.M., Fytas G. (2013). An E/Z Conformational Behaviour Study on the Trypanocidal Action of Lipophilic Spiro Carbocyclic 2,6-Diketopiperazine-1-Acetohydroxamic Acids. Tetrahedron Lett..

[42] Gondeau C. (2015). Hepatitis C Virus Infection: Are There Still Specific Problems with Genotype 3?. World J. Gastroenterol..

[43] Wu J., Liu W., Gong P. (2015). A Structural Overview of RNA-Dependent RNA Polymerases from the Flaviviridae Family. Int. J. Mol. Sci..

[44] Caillet-Saguy C., Lim S.P., Shi P.-Y., Lescar J., Bressanelli S. (2014). Polymerases of Hepatitis C Viruses and Flaviviruses: Structural and Mechanistic Insights and Drug Development. Antivir. Res..

[45] Czopek A., Kołaczkowski M., Bucki A., Byrtus H., Pawłowski M., Kazek G., Bojarski A.J., Piaskowska A., Kalinowska-Tłuścik J., Partyka A. (2015). Novel Spirohydantoin Derivative as a Potent Multireceptor-Active Antipsychotic and Antidepressant Agent. Bioorg. Med. Chem..

