# Supplementary material for: Novel Lipophilic Hydroxamates Based on Spirocarbocyclic Hydantoin Scaffolds with Potent Antiviral and Trypanocidal Activity
Source: Pharmaceuticals (Basel). 2023 Jul 24;16(7):1046. doi: 10.3390/ph16071046 (PMC10385743; doi:10.3390/ph16071046)

Supplementary Information

Novel lipophilic hydroxamates based on spirocarbocyclic hydantoin scaffolds with potent antiviral and trypanocidal activity

Vasiliki Pardali [1], Erofili Giannakopoulou [1], George Mpekoulis [2], Vassilina Tsopela [2], Georgios Panos [2], Martin C. Taylor [3], John M. Kelly [3], Niki Vassilaki [2] and Grigoris Zoidis [1,*]

[1] School of Health Sciences, Department of Pharmacy, Division of Pharmaceutical Chemistry, National and Kapodistrian University of Athens, Panepistimiopolis Zografou, 15771 Athens, Greece; vasilikipard@pharm.uoa.gr (V.P.); evgian@pharm.uoa.gr (E.G.)

[2] Molecular Virology Laboratory, Hellenic Pasteur Institute, Vas. Sofias Avenue, GR-11521, Athens, Greece; nikiv@pasteur.gr (N.V.); g.mpekoulis@pasteur.gr (G.M.); vas.tsopela@gmail.com (V.T.); panosgeorgiosbio@gmail.com (G.P.)

[3] Department of Infection Biology, London School of Hygiene and Tropical Medicine, Keppel Street, London WC1E 7HT, UK; Martin.Taylor@lshtm.ac.uk (M.C.T.); John.Kelly@lshtm.ac.uk (J.M.K.)

* Correspondence: zoidis@pharm.uoa.gr (G.Z.); Tel.: +30-210-7274809

List of Contents

I. ^{1}H NMR and ^{13}C NMR spectra of 4-phenyl-cyclohexane substituted analogue 9 **3**
II. Experimental procedures for the biological evaluation of the compounds **8**
IIa. Anti-HCV Activity **8**
IIb. Trypanocidal Activity **9**
III. References **10**

IV. Copies of NMR spectra **11**
NMR spectra of **4** (^{1}H, ^{13}C, COSY, HSQC, HMBC) **11**
NMR spectra of **6** (^{1}H, ^{13}C, COSY, HSQC, HMBC) **16**
NMR spectra of **7** (^{1}H, ^{13}C, COSY, HSQC, HMBC, NOESY) **21**
NMR spectra of **8** (^{1}H, ^{13}C, COSY, HSQC, HMBC) **27**
NMR spectra of **10** (^{1}H, ^{13}C, COSY, HSQC, HMBC) **32**
NMR spectra of **11** (^{1}H, ^{13}C, COSY, HSQC-DEPT, HMBC) **37**
NMR spectra of **12** (^{1}H, ^{13}C, COSY, HSQC, HMBC) **42**
NMR spectra of **14** (^{1}H, ^{13}C, COSY, HSQC, HMBC) **47**
NMR spectra of **15** (^{1}H, ^{13}C, COSY, HSQC, HMBC) **52**
NMR spectra of **16** (^{1}H, ^{13}C, COSY, HSQC, HMBC) **57**
NMR spectra of **17** (^{1}H, ^{13}C, COSY, HSQC-DEPT, HMBC) **62**
NMR spectra of **18** (^{1}H, ^{13}C, COSY, HSQC-DEPT, HMBC, NOESY) **67**
NMR spectra of **19** (^{1}H, ^{13}C, COSY, HSQC, HMBC) **73**
NMR spectra of **20** (^{1}H, ^{13}C, COSY, HSQC, HMBC) **78**
NMR spectra of **21** (^{1}H, ^{13}C, COSY, HSQC, HMBC) **83**
NMR spectra of **22** (^{1}H, ^{13}C, COSY, HSQC-DEPT, HMBC) **88**
NMR spectra of **23** (^{1}H, ^{13}C, COSY, HSQC, HMBC) **93**
NMR spectra of **24** (^{1}H, ^{13}C, COSY, HSQC, HMBC) **98**
NMR spectra of **25** (^{1}H, ^{13}C, COSY, HSQC, HMBC) **103**
NMR spectra of **26** (^{1}H, ^{13}C, COSY, HSQC, HMBC) **108**
NMR spectra of **27** (^{1}H, ^{13}C, COSY, HSQC, HMBC) **113**
NMR spectra of **28** (^{1}H, ^{13}C, COSY, HSQC-DEPT, HMBC) **118**
NMR spectra of **29** (^{1}H, ^{13}C, COSY, HSQC, HMBC) **123**
NMR spectra of **30** (^{1}H, ^{13}C, COSY, HSQC-DEPT, HMBC) **128**
NMR spectra of **31** (^{1}H, ^{13}C, COSY, HSQC-DEPT, HMBC) **133**
NMR spectra of **32** (^{1}H, ^{13}C, COSY, HSQC-DEPT, HMBC) **138**
NMR spectra of **33** (^{1}H, ^{13}C, COSY, HSQC-DEPT, HMBC) **143**
NMR spectra of **34** (^{1}H, ^{13}C, COSY, HSQC-DEPT, HMBC, NOESY) **148**
NMR spectra of **35** (^{1}H, ^{13}C, COSY, HSQC, HMBC, NOESY) **154**
NMR spectra of **36** (^{1}H, ^{13}C, COSY, HSQC-DEPT, HMBC) **160**
NMR spectra of **37** (^{1}H, ^{13}C, COSY, HSQC, HMBC) **165**
NMR spectra of **38** (^{1}H, ^{13}C, COSY, HSQC, HMBC) **170**
NMR spectra of **39** (^{1}H, ^{13}C, COSY, HSQC-DEPT, HMBC) **175**
NMR spectra of **40** (^{1}H, ^{13}C, COSY, HSQC-DEPT, HMBC) **180**
NMR spectra of **41** (^{1}H, ^{13}C, COSY, HSQC, HMBC) **185**
NMR spectra of **42** (^{1}H, ^{13}C, COSY, HSQC-DEPT, HMBC) **190**
NMR spectra of **46** (^{1}H, ^{13}C, COSY, HSQC, HMBC) **195**
NMR spectra of **47** (^{1}H, ^{13}C, COSY, HSQC, HMBC) **200**
NMR spectra of **48** (^{1}H, ^{13}C, COSY, HSQC-DEPT, HMBC) **205**
NMR spectra of **49** (^{1}H, ^{13}C, COSY, HSQC-DEPT, HMBC) **210**
NMR spectra of **50** (^{1}H, ^{13}C, COSY, HSQC, HMBC) **215**
NMR spectra of **51** (^{1}H, ^{13}C, COSY, HSQC, HMBC) **220**
NMR spectra of **52** (^{1}H, ^{13}C, COSY, HSQC-DEPT, HMBC) **225**
NMR spectra of **53** (^{1}H, ^{13}C, COSY, HSQC, HMBC) **230**

I. ^{1}H NMR and ^{13}C NMR spectra of 4-phenyl-cyclohexane substituted analogue 9

Of particular interest are the NMR spectra of 4-phenyl-cyclohexane substituted analogues. In the chair configuration of cyclohexane, substituents can take either axial or equatorial positions. Nevertheless, the two isomeric structures cannot be distinguished, due to the rapid interconversion between the two configurations, resulting in the alternation of axial and equatorial positions. In the case of 1,4-disubstituted cyclohexanes, the two conformers are not equally stable, and the equatorial-conformer is more stable than the axial one. This is due to the presence of stereochemical interactions between the axial substituents of C-1 and C-4 and the two axial hydrogens of C-3 and C-5, the so-called 1,3-diaxial interactions (**SI Fig. 1**).

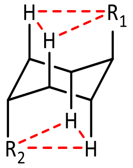

SI FIGURE 1. 1,3-Diaxial interactions in axially substituted cyclohexane 'chair-like' transition state. (chair configuration of cyclohexane)

The stereochemistry of Bucherer-Bergs and Strecker reactions for substituted cyclohexanes has been extensively studied. It was first noted that, in the case of 4-tert-butylcyclohexanone, Bucherer-Bergs reaction affords predominantly one of the stereoisomeric hydantoins, while the other stereoisomer is only obtained in traces. The main product of the Bucherer-Bergs reaction is the α-isomer, while that of the Strecker reaction is the β-isomer (**Fig. 2**).

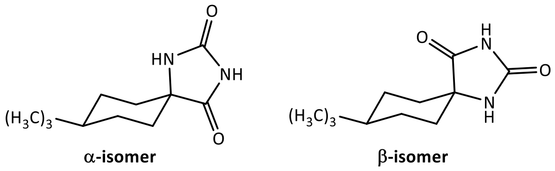

SI FIGURE 2| Major products of the Bucherer-Bergs and Strecker reactions of 4-tert-butylcyclohexanone.

The limiting step for the reaction is the cyclization of cyanocarbamic acid to 5-imino-2-oxazolidinone (**SI Scheme 1**, **SI Scheme 2**). The formation of the α-conformer as the main product is a result of steric hindrance between the atoms of the C=NH bond and the 3,5-axial hydrogens in the limiting step of the mechanism, which leads to the *cis* arrangement of the imino group and the *tert*-butyl group.

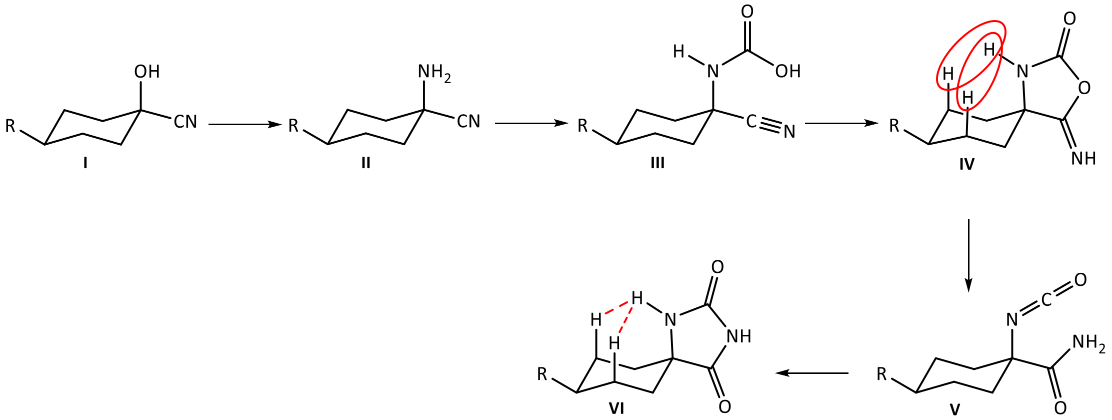

SI Scheme 1. Basic structures of the *Bucherer-Bergs* reaction mechanism. In structure **IV** and **VI** the 1,3-diaxial interactions are highlighted.

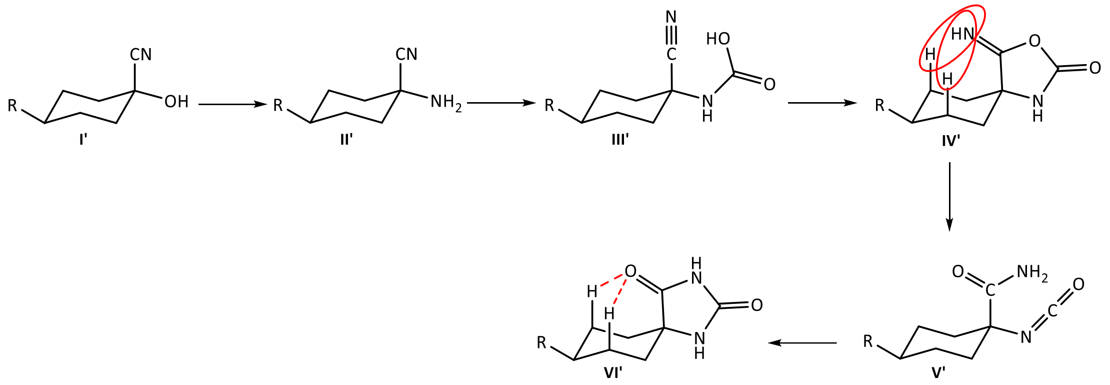

SI Scheme 2. Basic structures of the *Strecker* reaction mechanism. In structure **IV'** and **VI'** the 1,3-diaxial interactions are highlighted.

Measurements of the distances between the axial hydrogens and the hydrogen of N-1' of structure **VI**, as well as the axial hydrogens and oxygen of C-4' of structure **VI'**, showed that the steric strain is greater in the latter case, which proves that that the formation of the isomer, in which the amino group is in *cis* configuration with the R substituent, is favored. The fact that the α-former is formed preferentially over the β indicates that the first pathway involves a lower overall energy barrier during the transition of hydantoin reactants. In the first run, structure **III** is less favorable than **III'**, however the steric hindrance in structures **IV'**, **V'** and **IV'** is greater compared to **IV**, **V** and **IV** and the total energy sum is greater in the case of the second course. Also, when applying the *Bucherer-Bergs* reaction to 4-benzoyloxycyclohexanone, the two isomers were formed corresponding to the above in a ratio of 3:1 for the isomer in which the NH group is in a cis arrangement with the 4- benzoyloxy substituent to the isomer, in which the two groups are in a trans arrangement. In the spectra of the 4-phenyl-cyclohexane substituted analogues different peaks can be distinguished, which correspond to the two conformers, according to what was mentioned above. The ^{1}H NMR and ^{13}C NMR spectra of benzyl ester **9** (**SI FIGURE 4**, **SI FIGURE 5**) of the series of 4-phenyl-cyclohexane substituted analogs are listed below. The amide proton peak for the **A** isomer, where the NH group is in *cis* configuration with the phenyl, appears at lower field values than for **B**. The methylene protons of the benzyl group of the **A** isomer are coordinated at higher field values due to of their shielding compared to those of **B**, while the methylene protons of the acetoxy group of the **A** conformer are more shielded than those of **B** and appear at lower field values. The relative complements of the peaks show that the ratio of the two isomers **A** and **B** is 5:1.

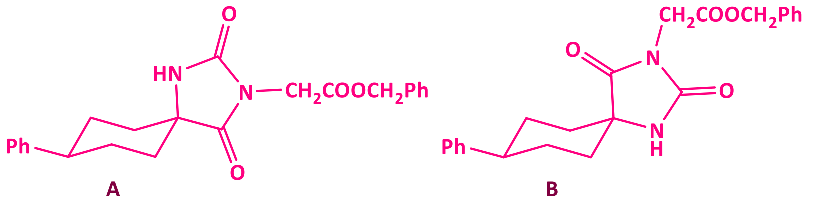

SI FIGURE 3| Benzyl ester **9** conformers.

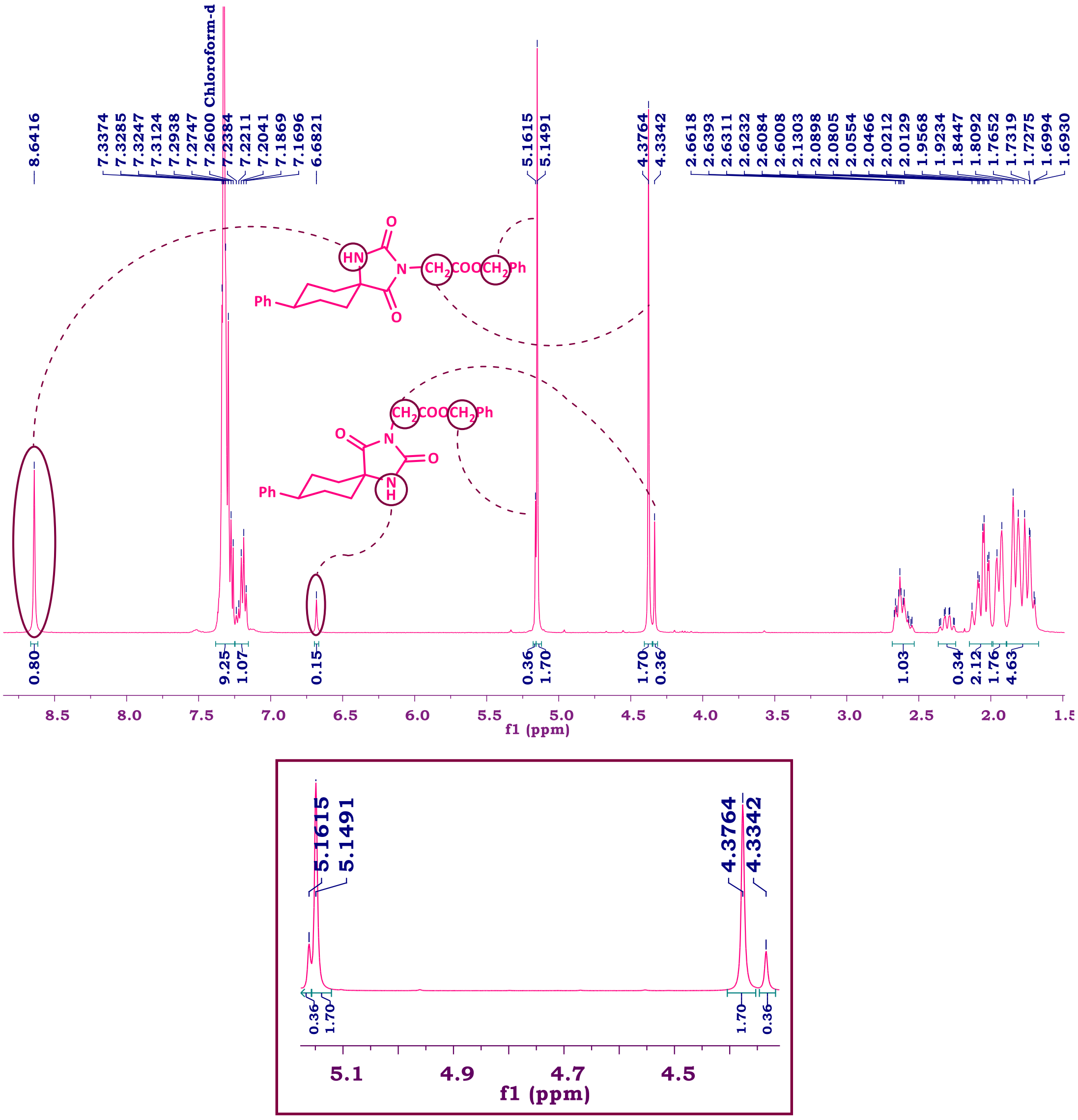

SI FIGURE 4. Top: ^{1}H NMR spectrum (1.50-9.0 ppm) of compound **9** (400 MHz, $CDCl_3$), where the characteristic peaks corresponding to the two conformers **A** and **B** are marked. Bottom: Broadening of the 4.30-5.17 ppm region of the 1H NMR spectrum of the compound 21. On the right are the two peaks for the methylene protons of the acetoxy group and on the left the methylene protons of the benzyl group.

In the ^{13}C NMR spectrum (**SI FIGURE 5**), differences are observed for the two conformers at the cyclohexane carbons C-5, C-6/10, C-8, the aromatic carbons of the 4-phenyl substituent C-1' and C-2 '/6', on the three carbonyl carbons and the methylene carbon of the acetoxy group.

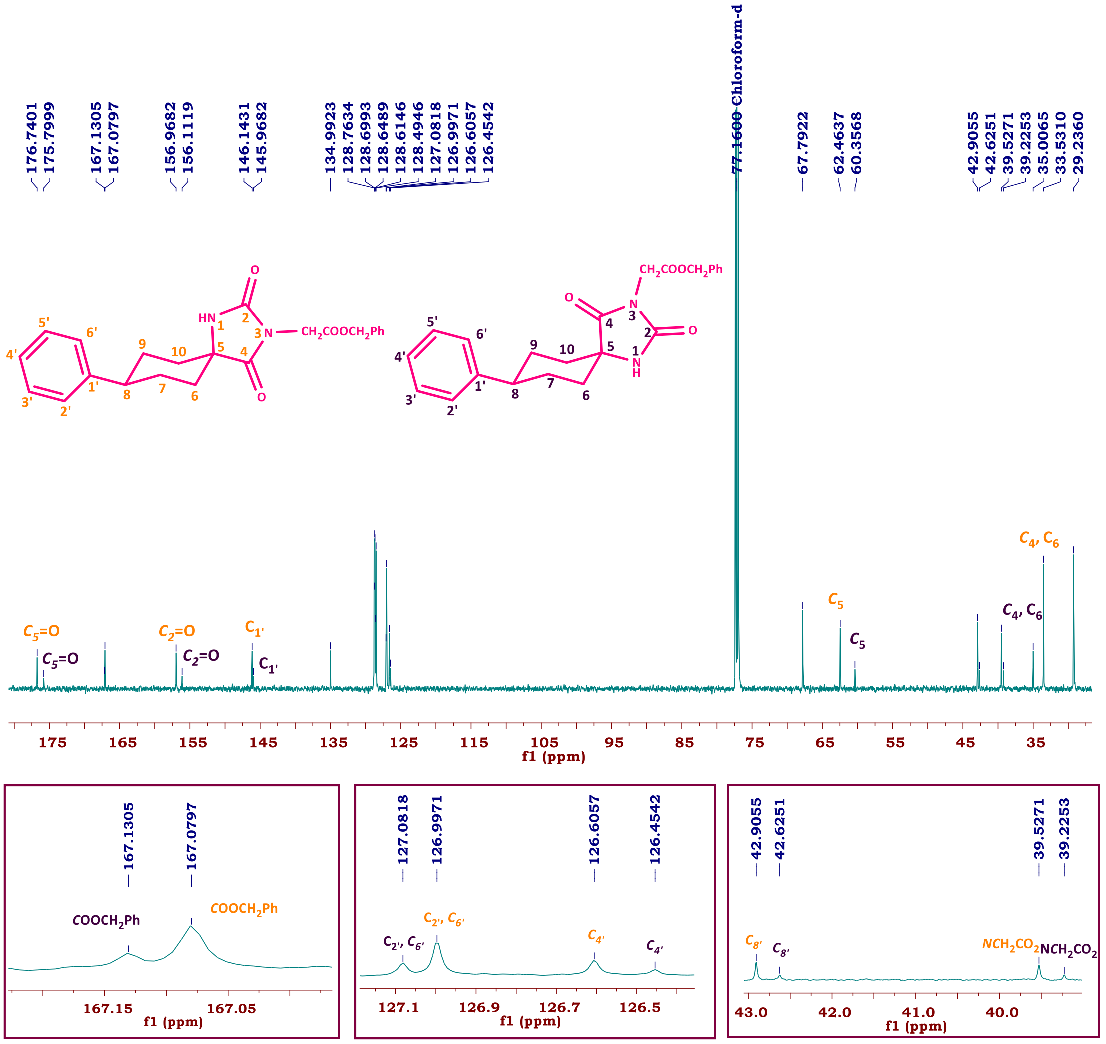

SI FIGURE 5 Top: ^{13}C NMR spectrum (22.5-180.0 ppm) of compound **9** (150 MHz, CDCl3). Carbons in the *cis* configuration are highlighted in orange and their counterparts in the *trans* configuration in purple. Bottom (from left to right): Enlargement of the 166.97-167.22 ppm, 126.37-127.15 ppm, and 39.0-43.0 ppm region of the ^{13}C NMR spectrum of compound **9**. Carbons in the *cis* configuration are highlighted in orange and the corresponding ones in the trans configuration are purple.

In the case of the methylated analogs, due to the stronger 1,3-diaxial interactions of the N-methyl group and the axial hydrogens of C-3 and C-5 of the *cis* configuration (**SI FIGURE 6**), exclusively (>99%) the conformer is formed , in which the methylated nitrogen and the 4-phenyl substituent are in a *trans* arrangement to each other.

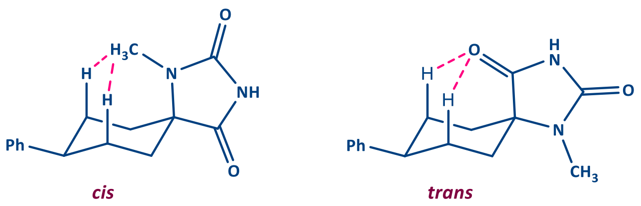

SI FIGURE 6. *Cis* and *trans* arrangement of compound **20**. Schematic representation of the 1,3 diaxial interactions.

NOESY SPECTRA OF BENZYLESTER 7

As mentioned above, during the synthesis of the 2,4-diketoimidazolidine skeleton on the 4-methyl-1,2,3,4-tetrahydronaphthalene ring, an additional asymmetric carbon center was created and two diastereomers (4 stereoisomers) resulted, which were separated in the benzyl bromoacetate substitution step. To determine each pair of enantiomers, a two-dimensional NOESY experiment was performed to study nuclear spin couplings through space. In the 1,2,3,4-tetrahydronaphthalene backbone, the cyclohexane ring adopts the half chair conformation, which is depicted in **SI FIGURE 7**.

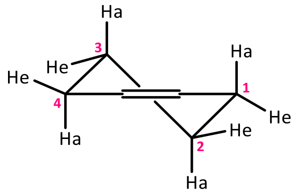

SI FIGURE 7. Half chair conformation of 1,2,3,4-tetrahydronaphthalene backbone.

From the NOESY spectrum of benzyl ester **7** (**SI FIGURE 9**), a signal is observed in the region 2.17-2.28 ppm (horizontal) and 2.93-3.05 ppm (vertical), in which the axial proton H2' and proton H4' are coordinated, while absent resonance peak in the region 1.31-1.40 ppm (vertical), which corresponds to CH_3 protons. The observed coupling between the axial proton H2' and H4' leads to the conclusion that they are in a *cis* arrangement with each other with H2'ax behind the plane and CH_3 above the plane.

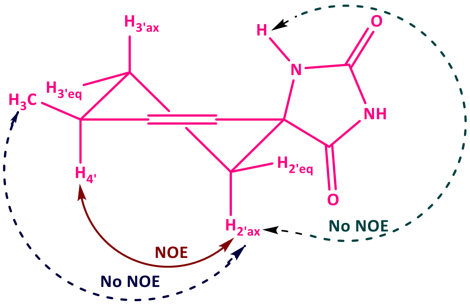

SI FIGURE 8. Schematic representation of NOE couplings.

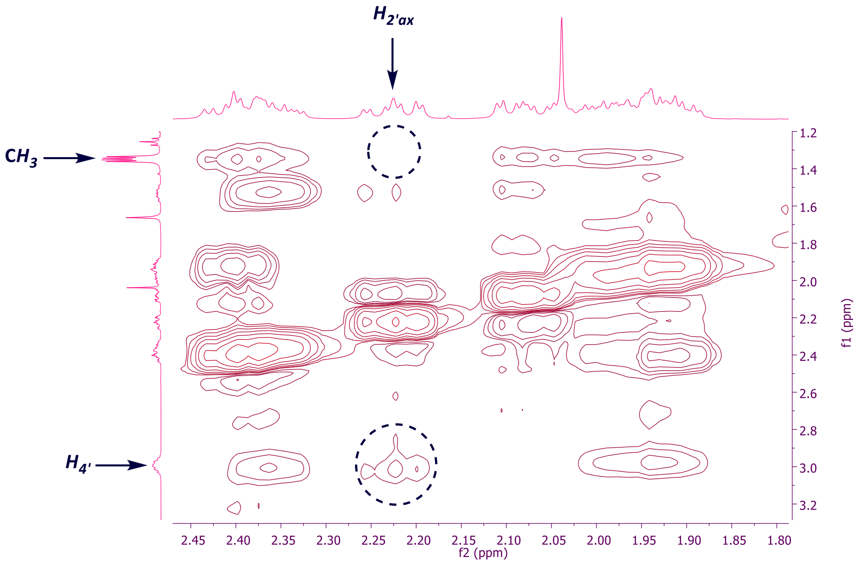

SI FIGURE 9. Region of the 2D NOESY spectrum (400 MHz, $CDCl_3$) of compound **7**.

Moreover, from the NOESY spectrum (**SI FIGURE 10**), the absence of coupling between the axial proton H2' and H3 of the NH group indicates that they are in a *trans* arrangement with each other.

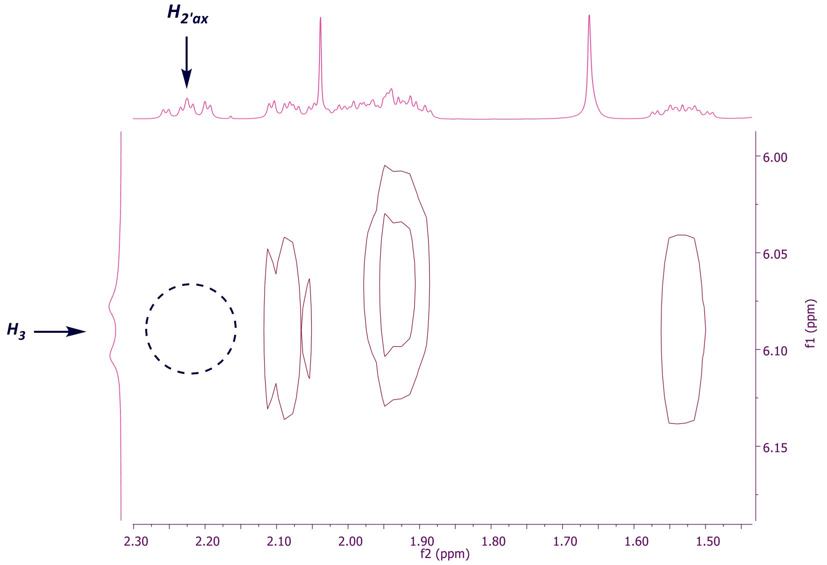

SI FIGURE 10. Region of the 2D NOESY spectrum (400 MHz, $CDCl_3$) of compound **7**.

NOESY data for compound **7** show that it is the *4R,4S′/4S,4R′* enantiomeric pair (**SI FIGURE 11**).

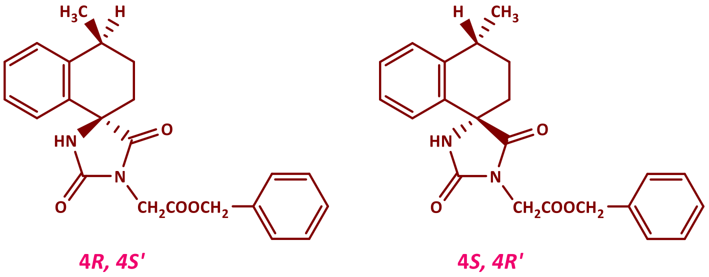

SI FIGURE 11. Structures of the pair of enantiomers *4R,4S′/4S,4R′*.

II. Experimental procedures for the biological evaluation of the compounds

IIa. Anti-HCV Activity

Cell Culture

The stable cell line Huh5-2 harboring the subgenomic reporter replicon of genotype 1b (Con1) has been previously described [1] (kindly provided by R. Bartenschlager, Heidelberg University, Germany). This is a bicistronic replicon expressing a firefly luciferase-ubiquitin-neomycin phosphotransferase fusion protein (luc-ubi-neo) under the translational control of the HCV internal ribosome entry site (IRES) and the HCV non-structural proteins NS3 to NS5B proteins under the control of the encephalomyocarditis virus (EMCV) IRES. The stable cell lines Huh7.5-3a and Huh7.5-4a have been constructed with transfection of the subgenomic reporter replicons of HCV 3a (S52) S52-SG(Feo)(AII) and HCV 4a (ED43) ED43-SG(Feo)(VYG) (kindly provided by C.M. Rice, The Rockefeller University, NY) [3], respectively, of the analogous design, and have been previously described [2]. The subgenomic reporter replicons DENV2 16681 pD2-hRUPac and YFV-17D pYF-hRUPac (kindly provided by C.M. Rice, The Rockefeller University, NY) have been described previously [4, 5] and were used to construct the stable cell lines Huh7-D2 and Huh7-YF [2], respectively. Cells were cultured in high glucose (25 mM) Dulbecco's modified minimal essential medium (Invitrogen), supplemented with 2 mM L-glutamine, 0.1 mM non-essential amino acids, 100 U/mL penicillin, 100 μg/mL streptomycin and 10% (v/v) fetal calf serum (referred to as complete DMEM). Complete DMEM was supplemented with G418, at 500 μg/mL for Huh5-2, 750 μg/ml for Huh7.5-3a and 350 μg/mL for Huh7.5-4a, or puromycin at 0.5 μg/ml for Huh7-D2 and 0.25 μg/ml for Huh7-YF.

Cell-based antiviral and cytotoxicity assays

Serial dilutions of the test compounds, or the solvent DMSO, were used to treat cells for 72 hours. To determine antiviral activity and cytotoxicity, virus-derived luciferase activity or intracellular ATP levels were measured, respectively. The median effective concentration (EC_{50}) of the compounds, decreasing luciferase activity by 50%, and their median cytotoxic concentration (CC_{50}), causing 50% cell death, were determined by nonlinear regression analysis using the Prism 6.0 software (GraphPad Software Inc.). Daclatasvir was kindly provided by Dr. Marc Windisch (Institut Pasteur Korea).

Luciferase and Bradford assays

Firefly luciferase activity was determined using Luciferase Assay System (Promega), according to the manufacturer's instructions in a GloMax 20/20 single tube luminometer (Promega) for 10 s. Values were normalized to total protein amounts that were quantified by Bradford assay (Pierce).

Measurement of intracellular ATP levels

ATP was measured using the ViaLight HS BioAssay kit (Lonza) according to the manufacturer's protocol in a GloMax 20/20 single-tube luminometer (Promega) for 1 s. Values were normalized to total protein amounts.

Gel electrophoresis and Western blot analysis

Denaturing SDS-polyacrylamide gel electrophoresis and Western blotting were performed as previously described [6]. Dilutions of 1:2,000 for HCV NS5A (9E10) monoclonal antibody ([7] kindly provided by Prof. C. Rice), 1:6,000 for β-actin monoclonal antibody (Merck-Millipore) and 1:2,000 for the secondary anti-mouse horseradish peroxidase-conjugated antibody (Cell Signaling) were used.

Total RNA extraction and quantification of viral replicons

Cells were lysed for total RNA extraction using Nucleozol reagent (Macherey-Nagel), according to the manufacturer's instructions. RNA samples were used to perform reverse-transcription (RT) and quantitative real-time polymerase chain reaction (qPCR) for viral RNA. RT was performed using Moloney Murine Leukemia Virus (MMLV) reverse transcriptase (Promega) and reverse primers specific for Con1 IRES (5'-GGATTCGTGCTCATGGTGCA-3') and the housekeeping gene YWHAZ (5'- GGATGTGTTGGTTGCATTTCCT-3'). For qPCR, primers specific for the Con1 IRES (forward: 5'-GGCCTTGTGGTACTGCCTGATA-3'and reverse: 5'-GGATTCGTGCTCATGGTGCA-3') and KAPA SYBR FAST qPCR Master Mix (Kapa Biosystems) were used. YWHAZ mRNA was used as a normalization control (forward: 5'-GCTGGTGATGACAAGAAAGG-3' and reverse: 5'- GGATGTGTTGGTTGCATTTCCT-3').

Statistical analysis

In all diagrams, bars represent mean values of three independent experiments in triplicate. Error bars represent standard deviation. Only results subjected to statistical analysis using Student's t-test with $p \leq 0.05$ were considered as statistically significant and presented. Statistical calculations were performed with Excel Microsoft Office®.

IIb. Trypanocidal Activity

For African trypanosomes, growth inhibition analysis was carried out using bloodstream form *T. b. brucei* (strain 221) cultured at 37°C, as previously described [8]. Briefly, experiments were performed in 96-well microtiter plates (200 µL volumes), with each assay initiated using 2.5×10^4 parasites mL^{-1}, and test compounds then added at 7 concentrations in a range that had been established in preliminary experiments to encompass both EC_{50} and EC_{90} values. Plates were incubated for 48 hours, resazurin (20 µL at 0.125 mg mL^{-1}) was then added, and the plates re-incubated for 16 hours. Fluorescence was determined using a BMG FLUOstar Omega plate reader (excitation 545 nm, emission 590 nm), and the data analysed using GraphPad Prism 9.0 software. The data are expressed as $EC_{50}/EC_{90} \pm$ SD, and are the average of 3 independent replicates.

T. cruzi (CL Brener strain) epimastigotes were grown at 28°C in supplemented RPMI-1640 medium. Inhibitory activity was assessed, as above, by culturing parasites in microtiter plates at different compound concentrations. Parasites were seeded at 2.5×10^5 mL^{-1} in 200 ◎L of growth medium. Plates were incubated for 5 days and 20 ◎L resazurin was then added to each well and the plates incubated for a further 24 hours. Fluorescence was determined by plate reader as outlined above.

To determine cytotoxicity, L6 cells (a rat myoblast line) were seeded at 1×10^4 mL^{-1} into 96-well microtiter plates (200 µL of growth medium), at a range of compound concentrations. After 6 days incubation at 37°C, 20 µL resazurin then added to each well. The plates were incubated for 6 hours, and fluorescence then determined by plate reader.

III. References

1. Vrolijk, J.M.; Kaul, A.; Hansen, B.E.; Lohmann, V.; Haagmans, B.L.; Schalm, S.W.; Bartenschlager, R. A replicon-based bioassay for the measurement of interferons in patients with chronic hepatitis C. *J. Virol. Methods.* **2003**, *110*, 201–209.

2. Giannakopoulou, E.; Pardali, V.; Frakolaki, E.; Siozos, V.; Myrianthopoulos, V.; Mikros, E.; Taylor, M.C.; Kelly, J.M.; Vassilaki, N.; Zoidis, G. Scaffold hybridization strategy towards potent hydroxamate-based inhibitors of Flaviviridae viruses and Trypanosoma species. *Med.Chem.Com.* **2019**, *10*, 991-1006.

3. Saeed, M.; Scheel, T.K.H.; Gottwein, J.M.; Marukian, S.; Dustin, L.B.; Bukh, J.; Rice, C.M. Efficient replication of genotype 3a and 4a hepatitis C virus replicons in human hepatoma cells. *Antimicrob. Agents Chemother.* **2012**, *56*, 5365–5373.

4. J. Dufner-Beattie, A. O'Guin, S. O'Guin, A. Briley, B. Wang, J. Balsarotti, R. Roth, G. Starkey, U. Slomczynska, A. Noueiry, P.D. Olivo, C.M. Rice, Identification of AP80978, a novel small-molecule inhibitor of hepatitis C virus replication that targets NS4B, Antimicrob. Agents Chemother. **2014**, *58*(6), 3399-410.

5. A.O. Noueiry, P.D. Olivo, U. Slomczynska, Y. Zhou, B. Buscher, B. Geiss, M. Engle, R.M. Roth, K.M. Chung, M. Samuel, M.S. Diamond, Identification of novel sma*ll-molecule inhibitors of West Nile virus infection, J. Virol.* **2007**, *81(21) 11992-2004*.

6. Vassilaki, N.; Boleti, H.; Mavromara, P.Expression studies of the core+1 protein of the hepatitis C virus 1a in mammalian cells. The influence of the core protein and proteasomes on the intracellular levels of core+1. *FEBS J.* **2007**, *274*, 4057–4074.

7. Lindenbach, B.D.; Evans, M.J.; Syder, A.J.; Wolk, B.; Tellinghuisen, T.L.; Liu, C.C.; Maruyama, T.; Hynes, R.O.; Burton, D.R.; McKeating, J.A.; et al. Complete replication of hepatitis C virus in cell culture. Science **2005**, *309*, 623–626, doi:10.1126/science.1114016.

8. Taylor, M.C.; McLatchie, A.; Kelly, J.M. Evidence that transport of iron from the lysosome to the cytosol in African trypanosomes is mediated by a mucolipin orthologue. *Molec. Microbiol.* **2013**, *89*, 420-32.

IV Copies of NMR spectra

^{1}H NMR of **4** (400.13 MHz, DMSO-d_6)

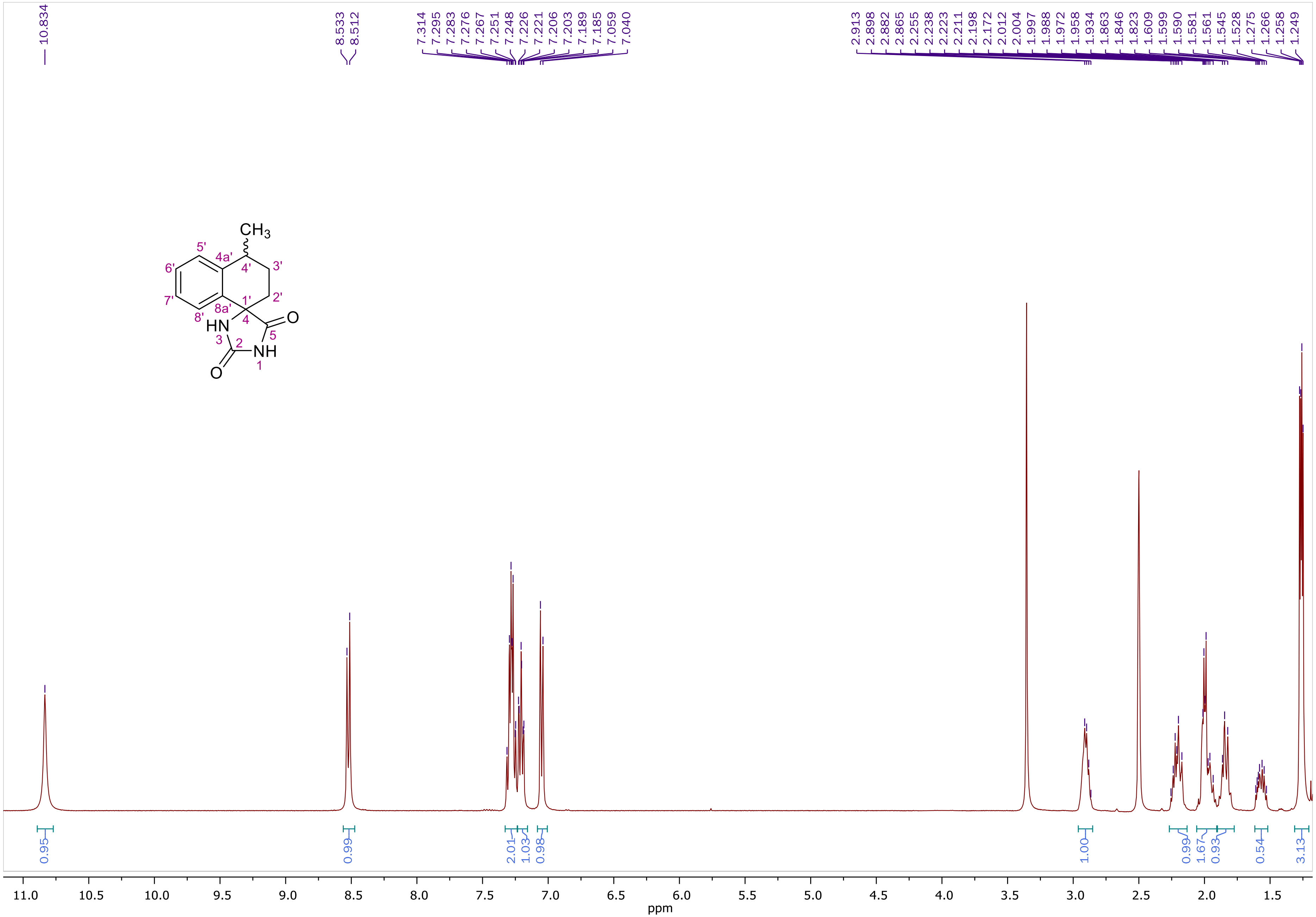

^{13}C NMR of **4** (50.32 MHz, DMSO-d_6)

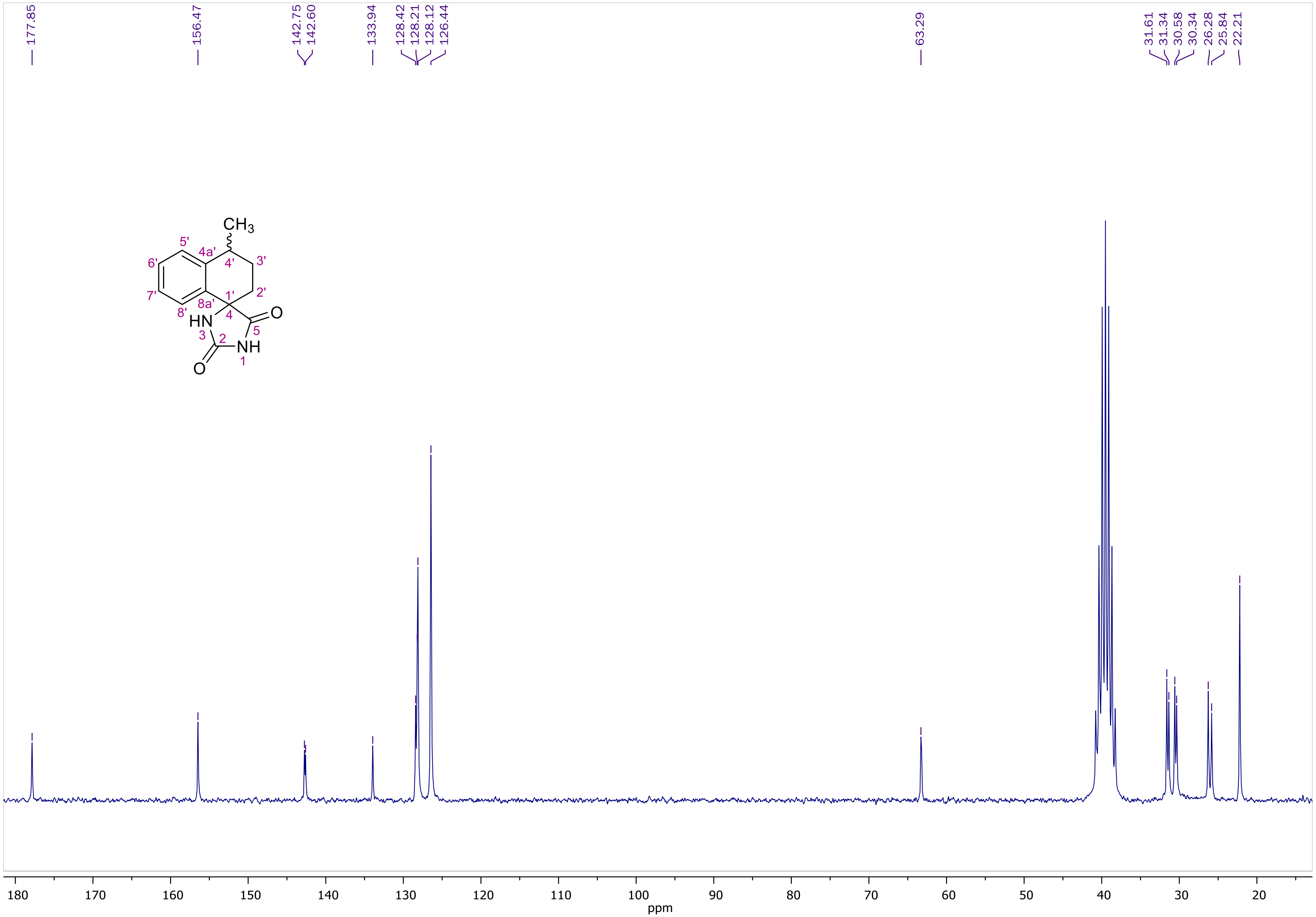

COSY NMR of **4** (400.13 MHz, DMSO-d_6)

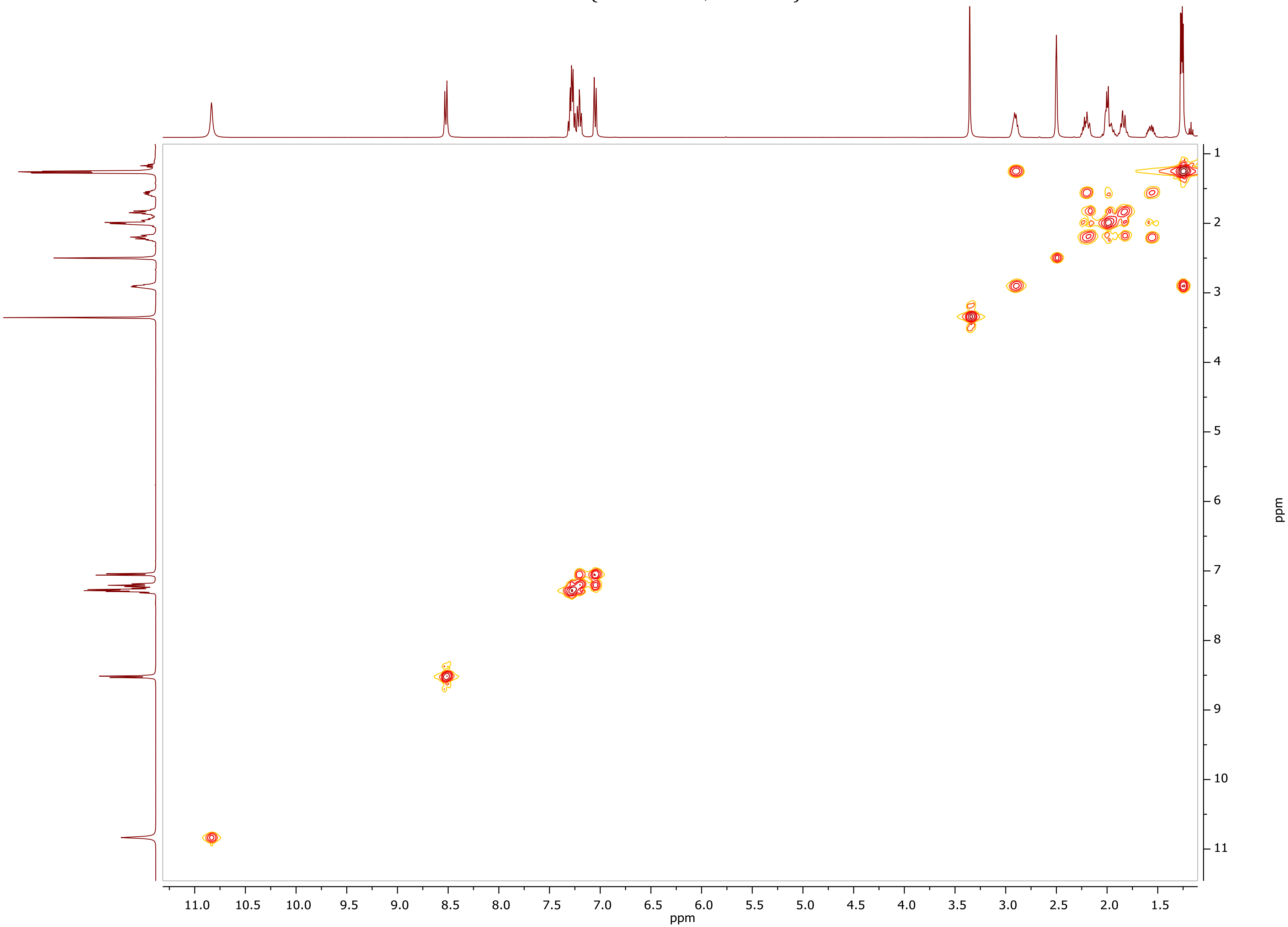

HSQC NMR of **4** (400.13 MHz, DMSO-d_6)

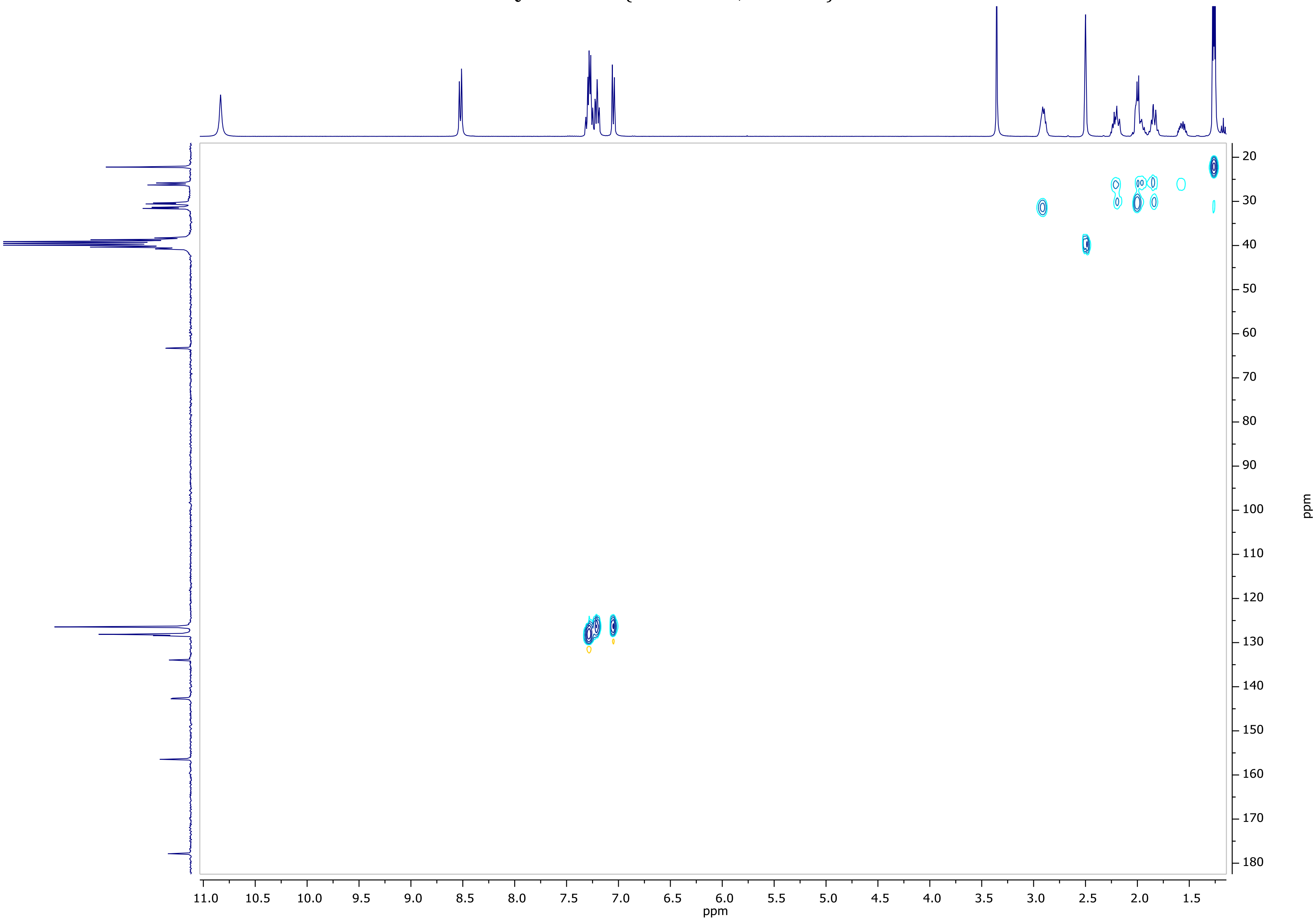

HMBC NMR of **4** (400.13 MHz, DMSO-d_6)

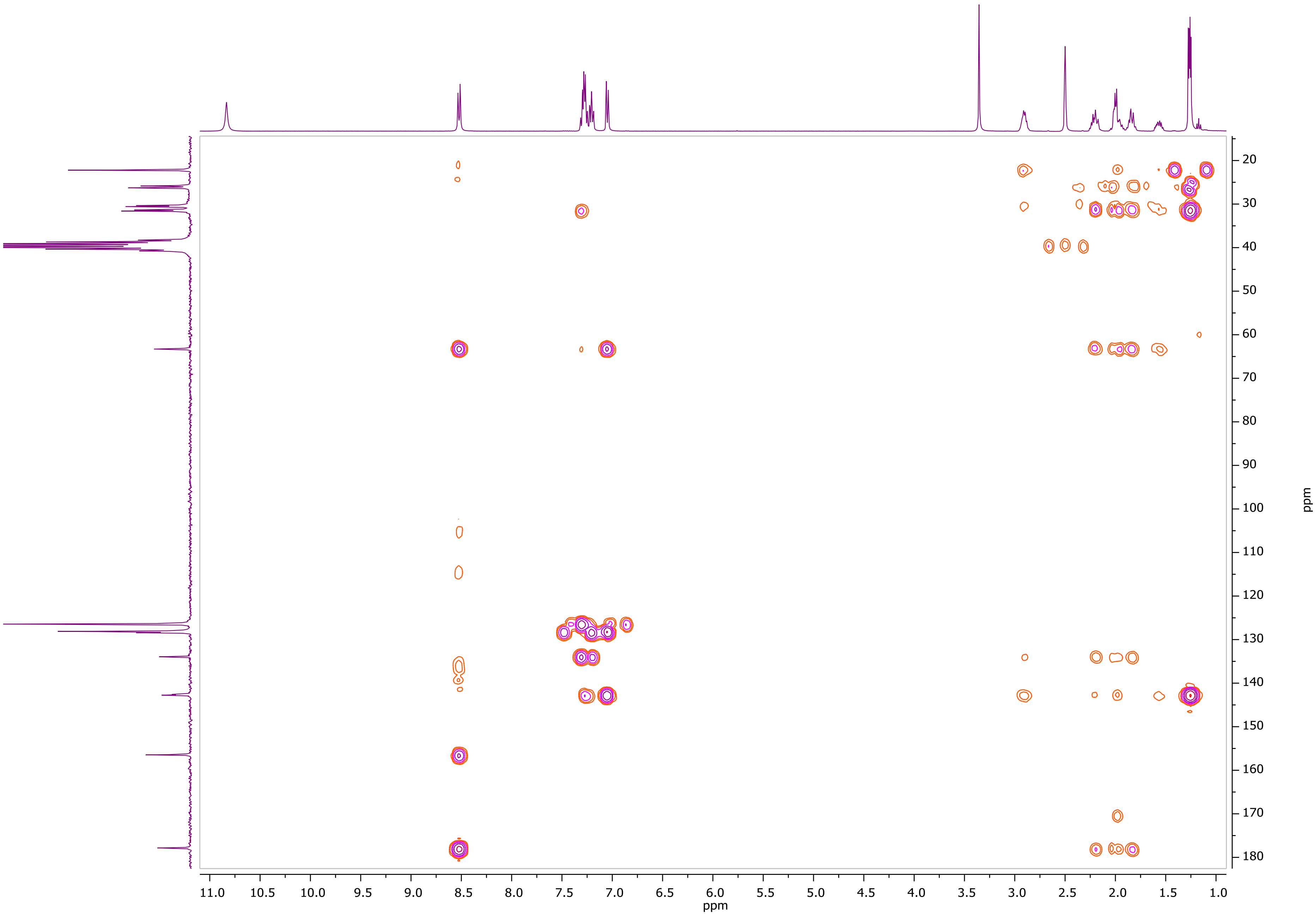

^{1}H NMR of **6** (600.11 MHz, DMSO-d_6)

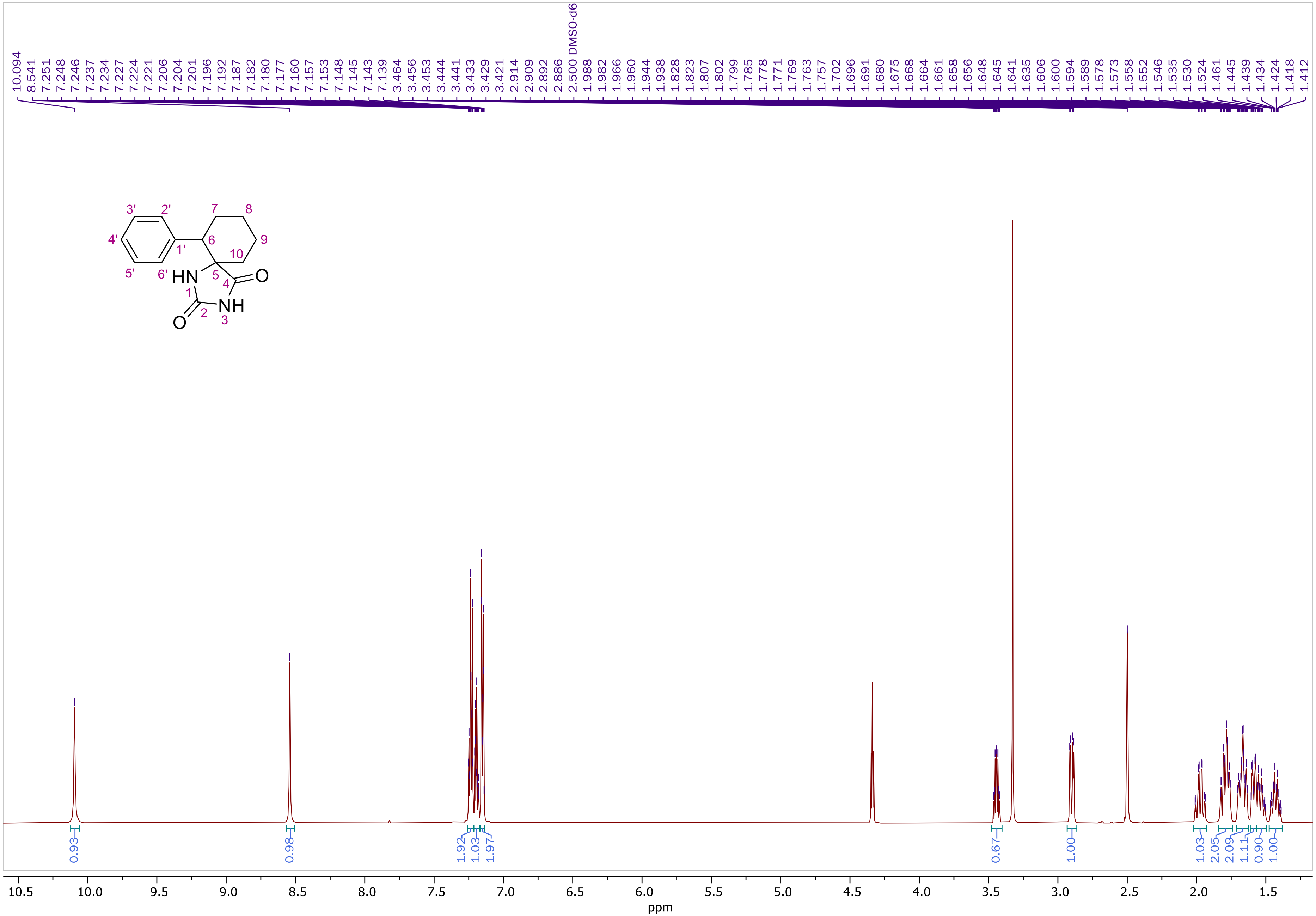

^{13}C NMR of **6** (150.9 MHz, DMSO-d_6)

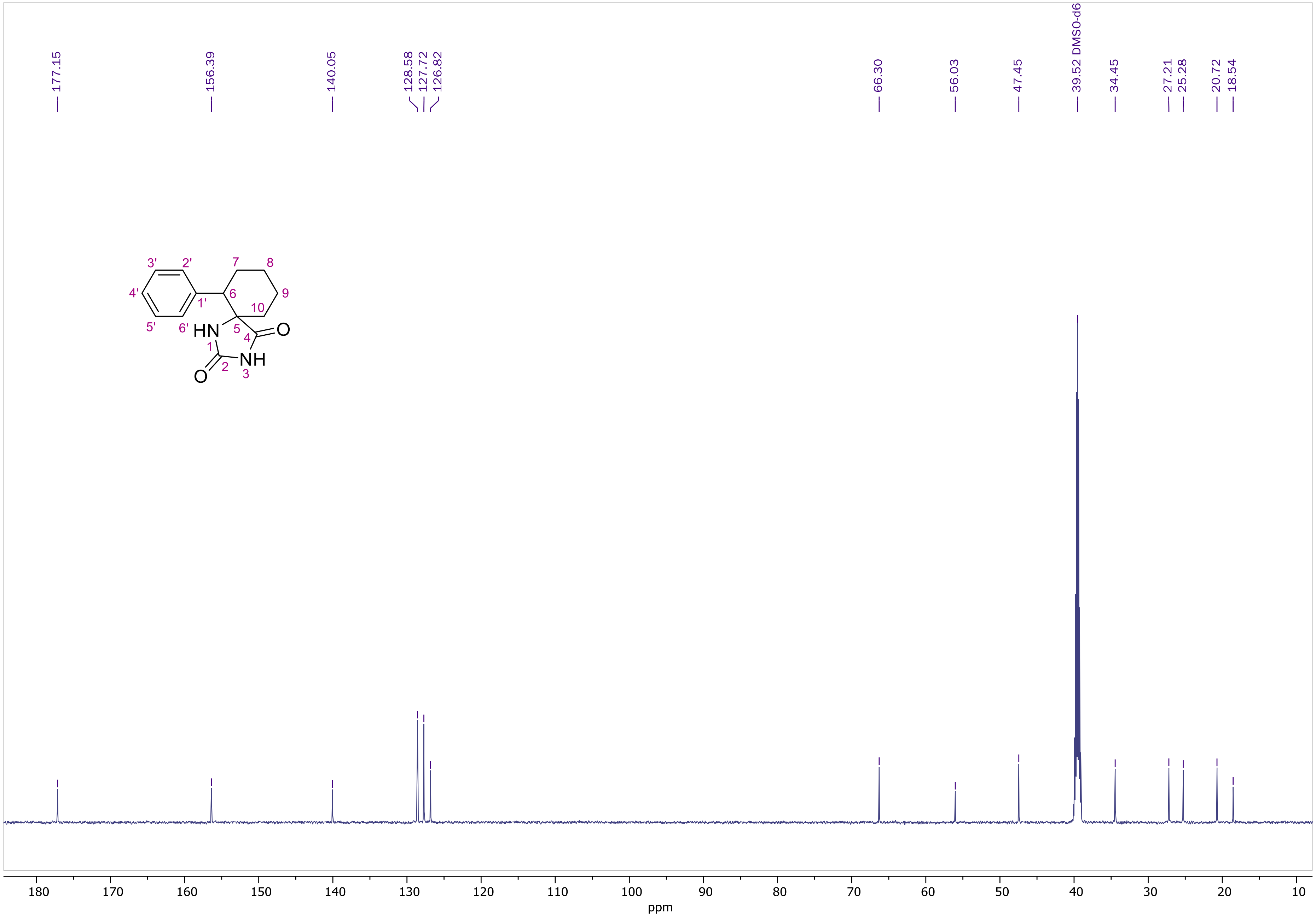

COSY NMR of **6** (400.13 MHz, DMSO-d_6)

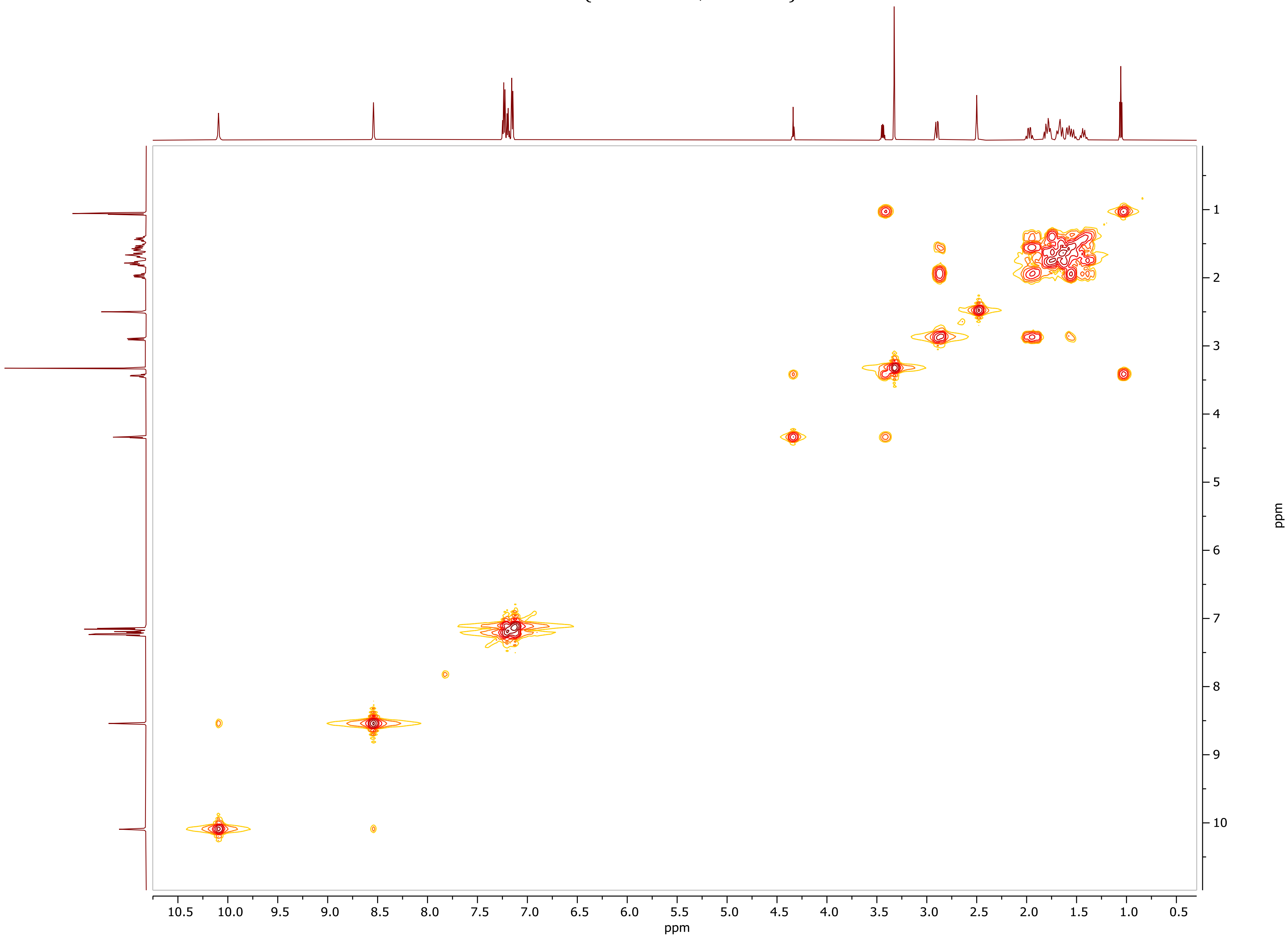

HSQC NMR of **6** (400.13 MHz, DMSO-d_6)

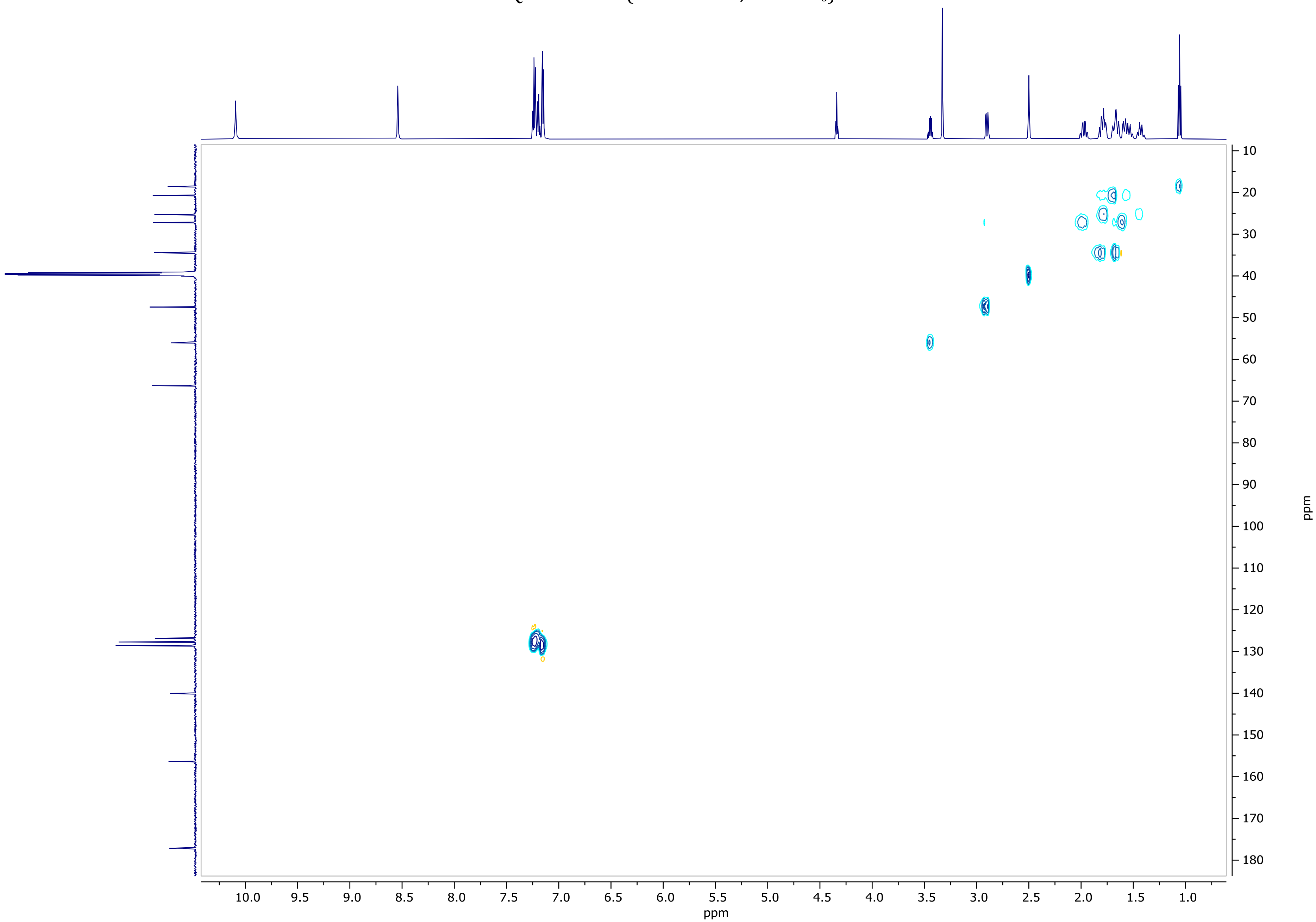

HMBC NMR of **6** (400.13 MHz, DMSO-d_6)

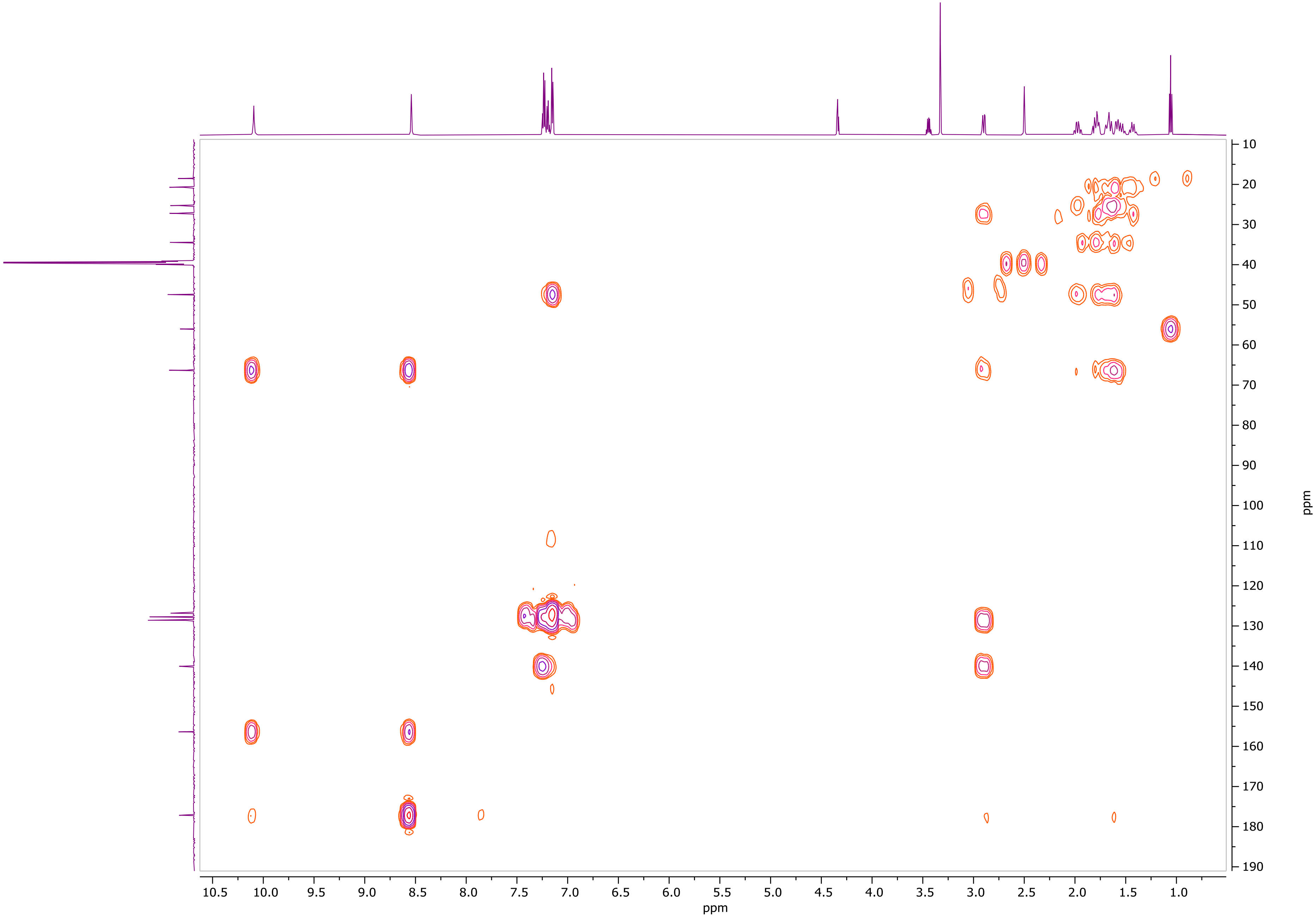

^{1}H NMR of **7** (400.13 MHz, $CDCl_3$)

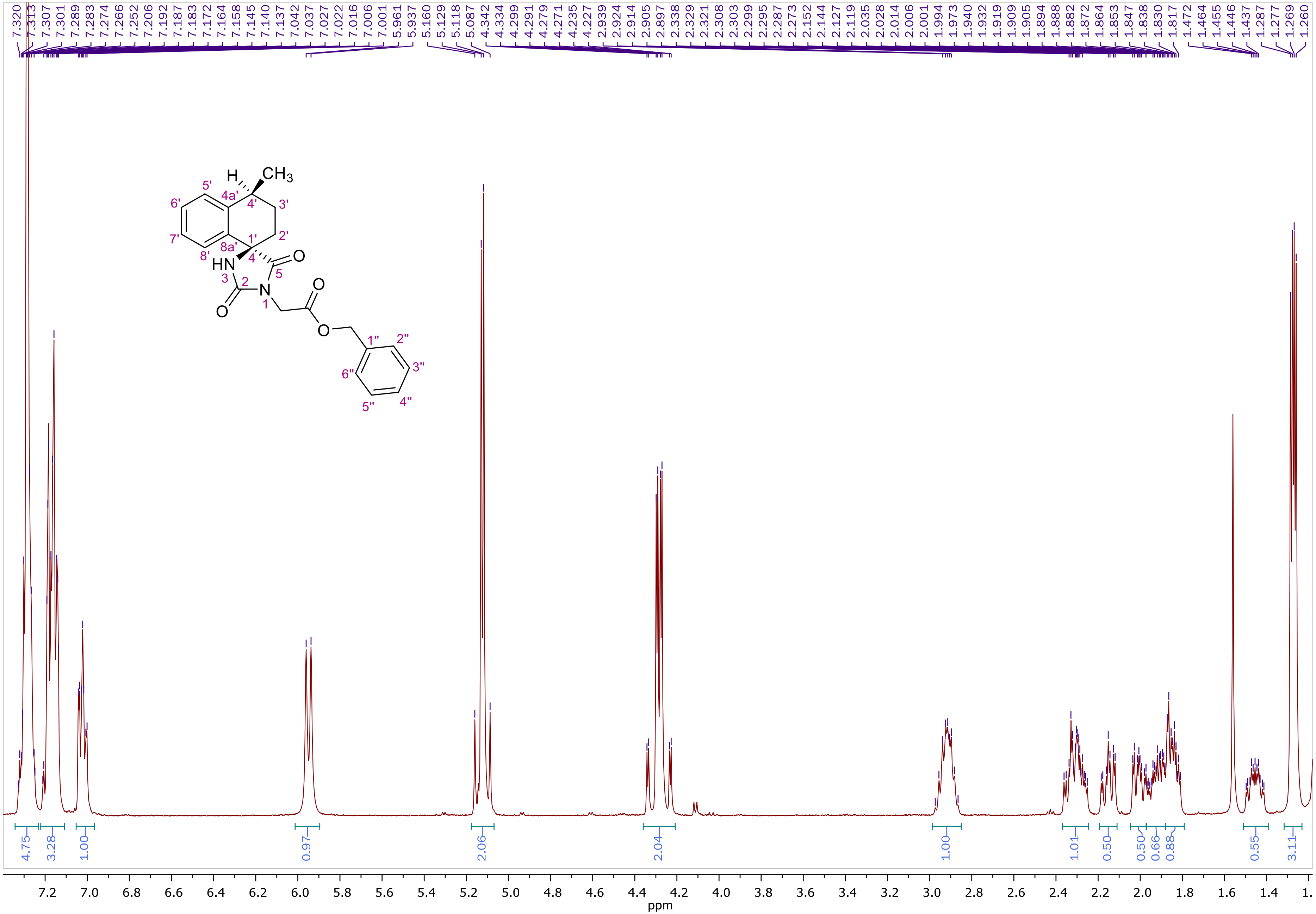

^{13}C NMR of **7** (50.32 MHz, $CDCl_3$)

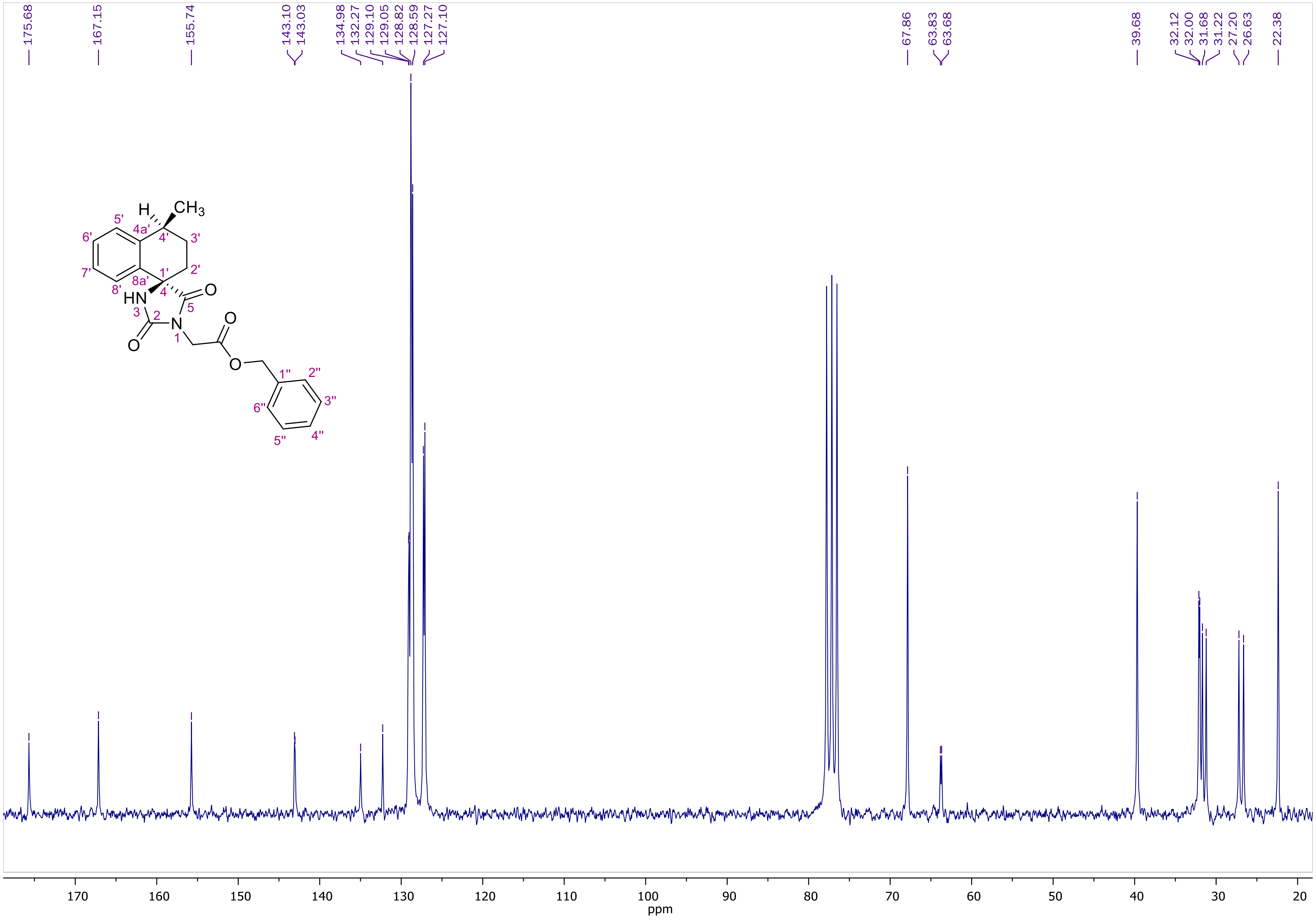

COSY NMR of **7** (400.13 MHz, $CDCl_3$)

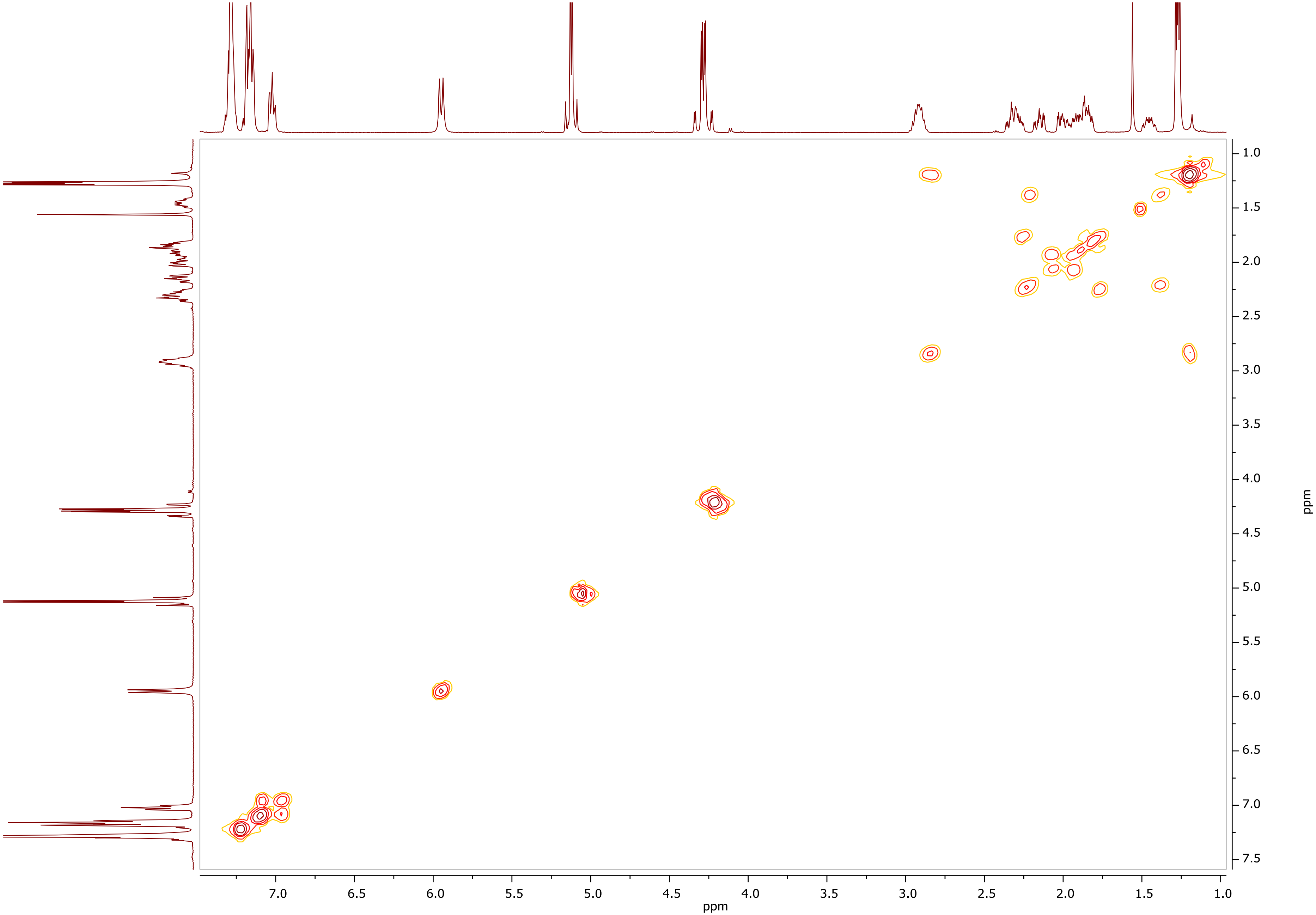

HSQC NMR of **7** (400.13 MHz, $CDCl_3$)

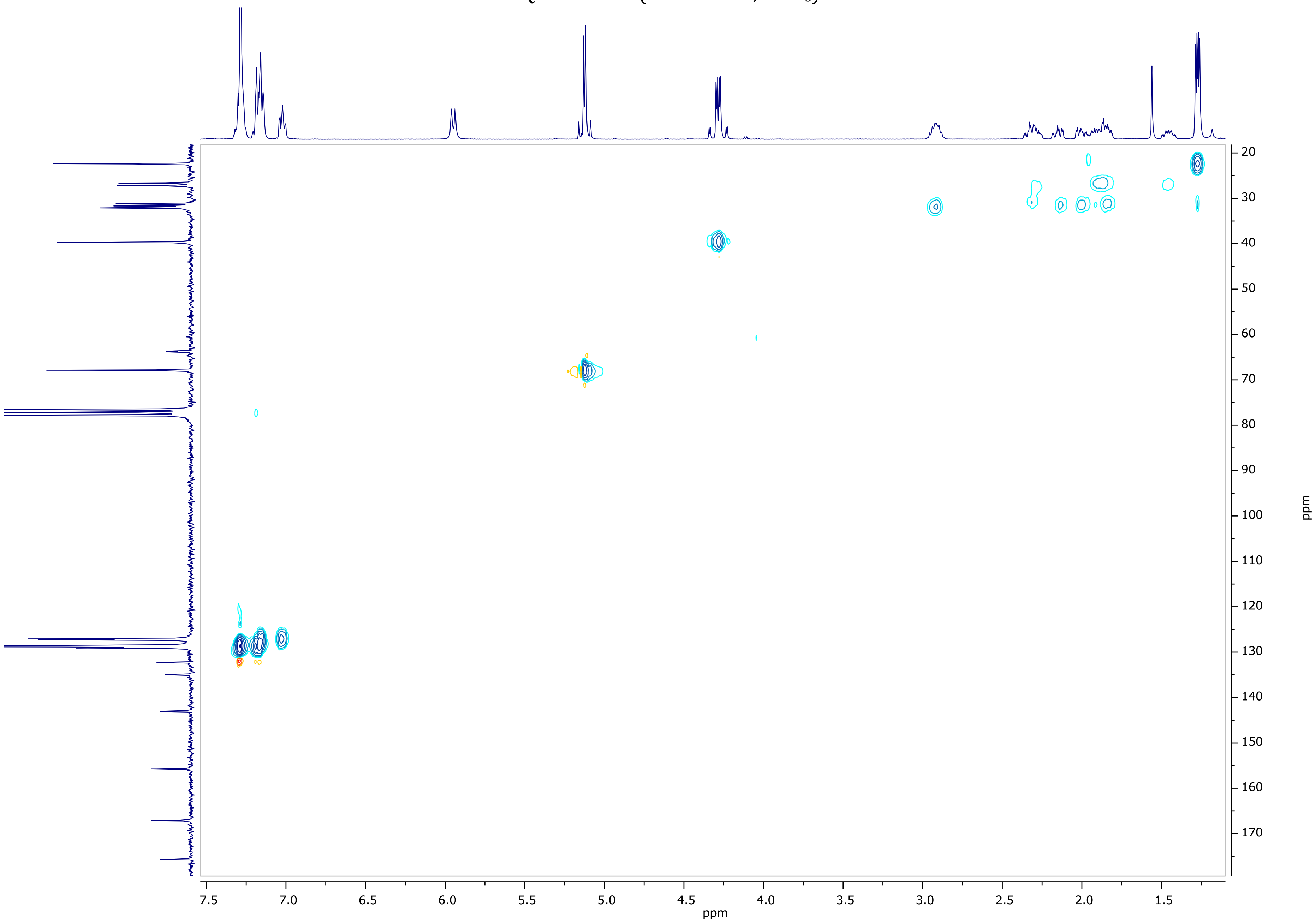

HMBC NMR of **7** (400.13 MHz, $CDCl_3$)

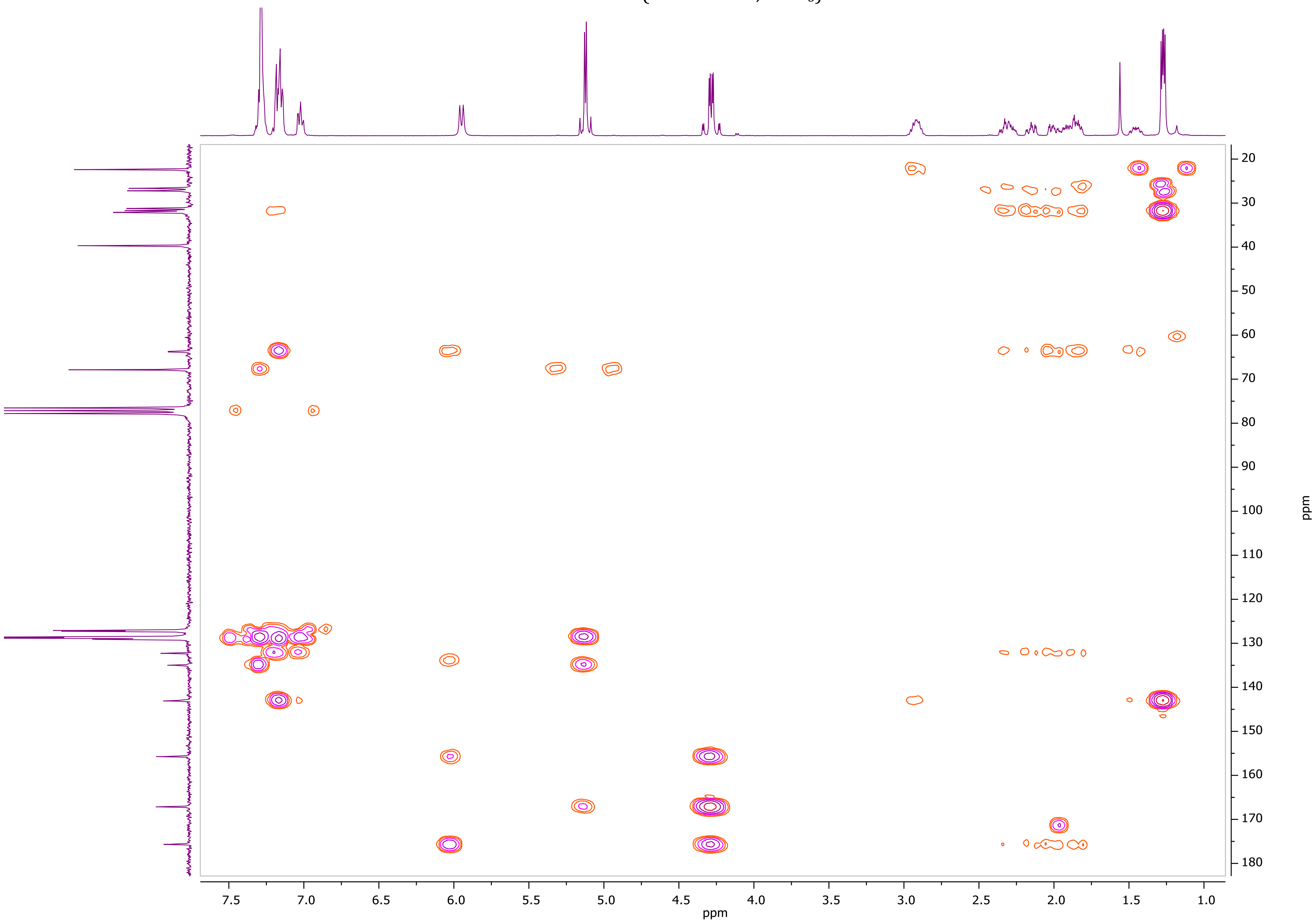

NOESY NMR of **7** (400.13 MHz, $CDCl_3$)

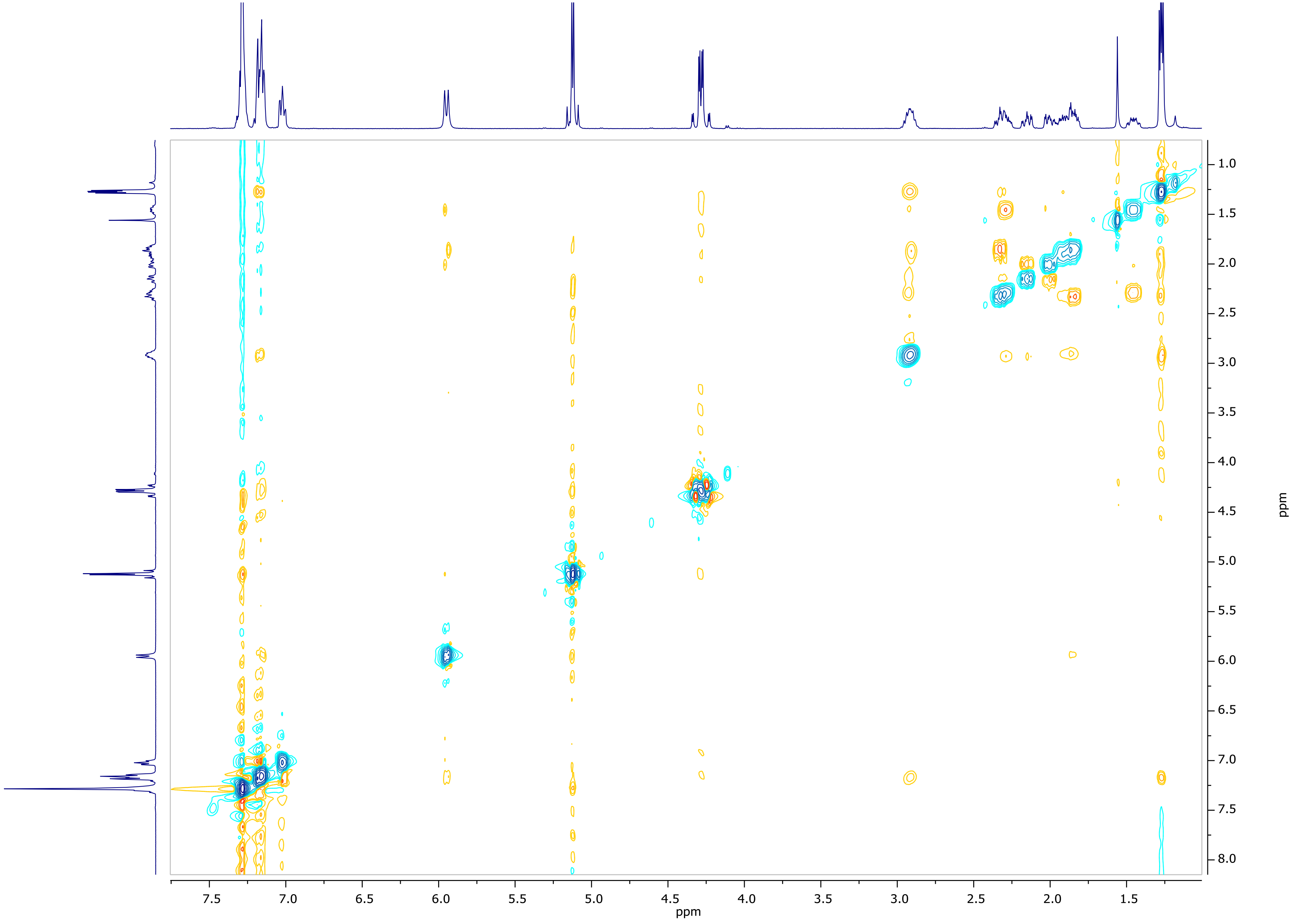

^{1}H NMR of **8** (400.13 MHz, $CDCl_3$)

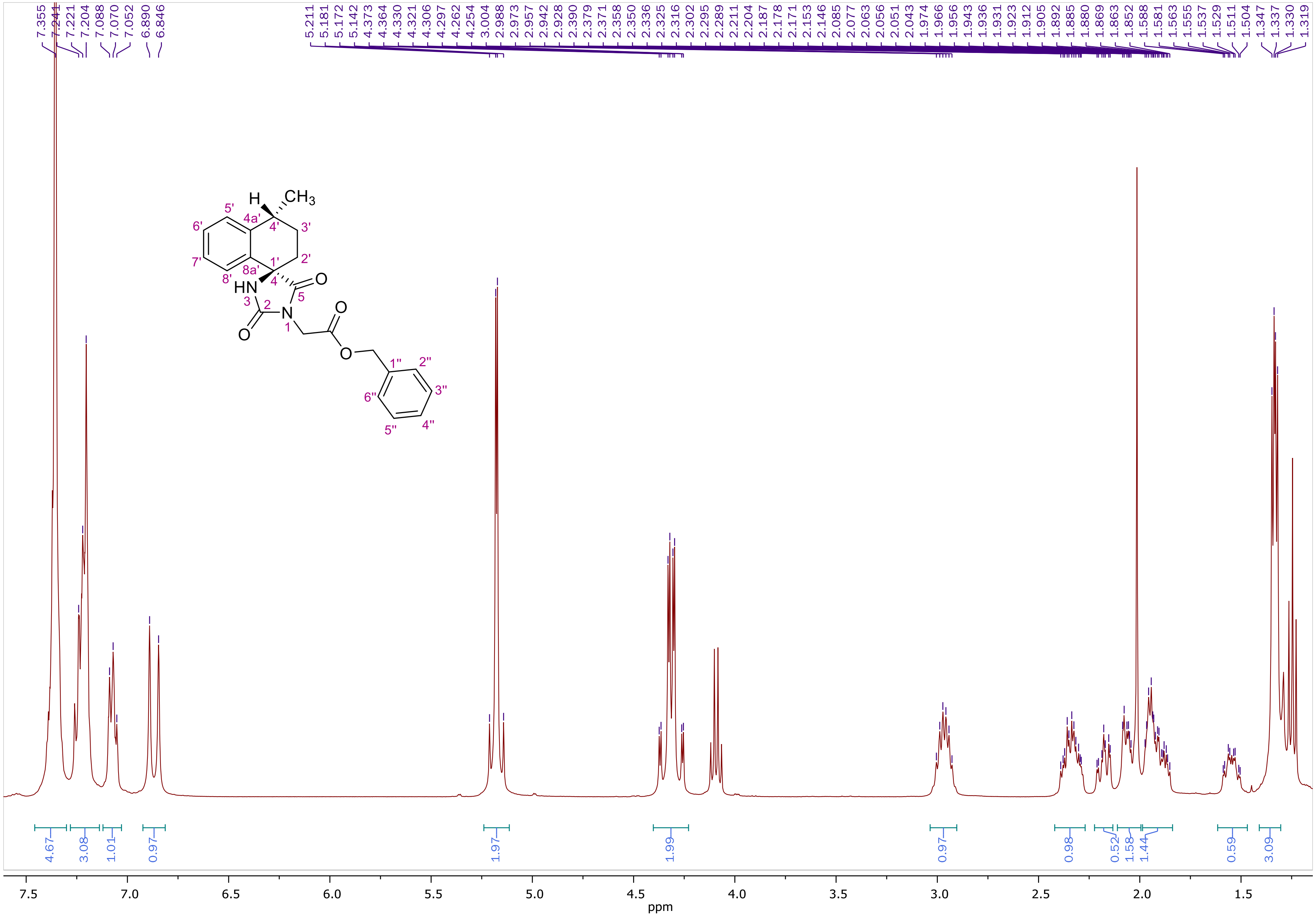

^{13}C NMR of **8** (50.32 MHz, $CDCl_3$)

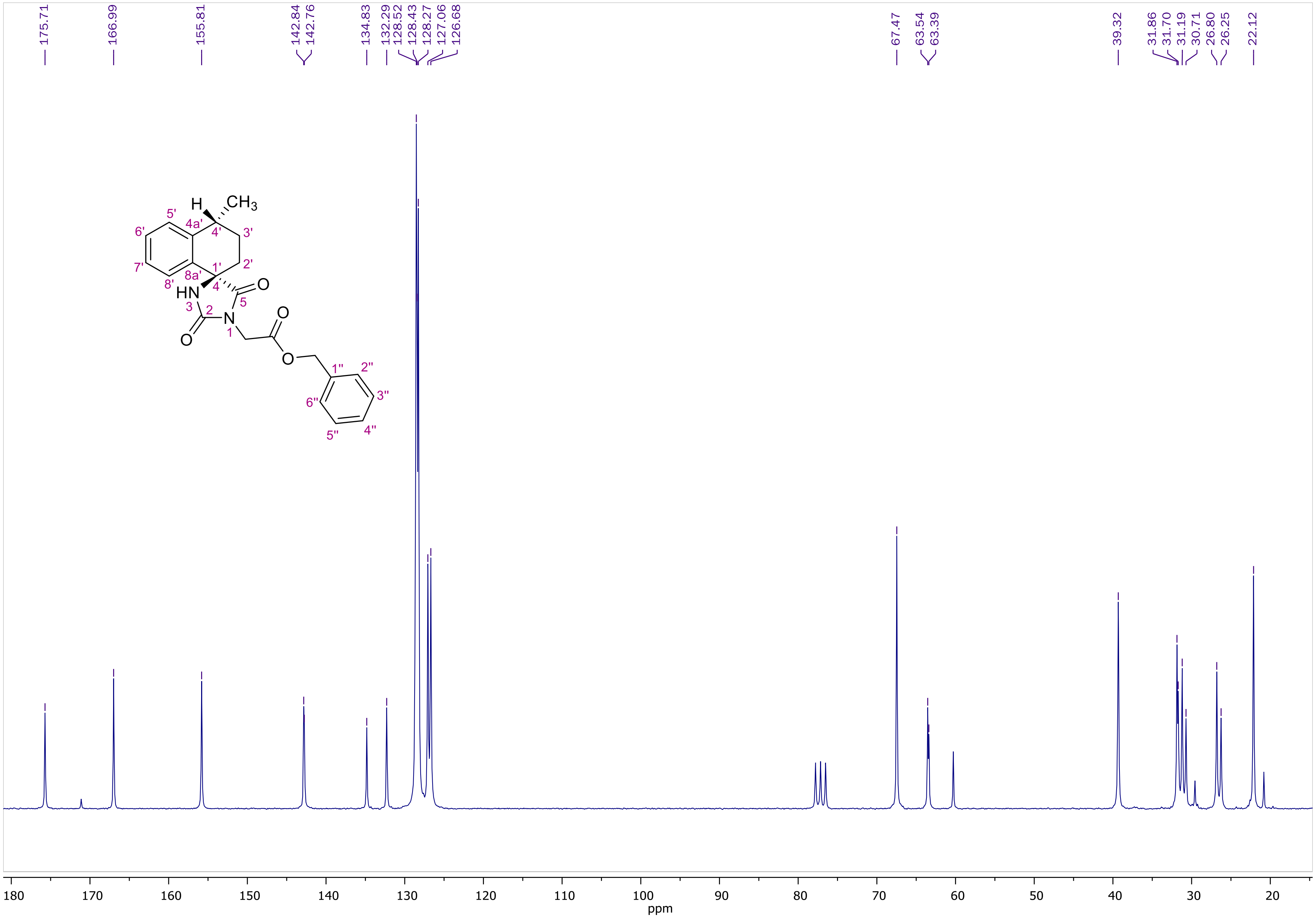

COSY NMR of **8** (400.13 MHz, $CDCl_3$)

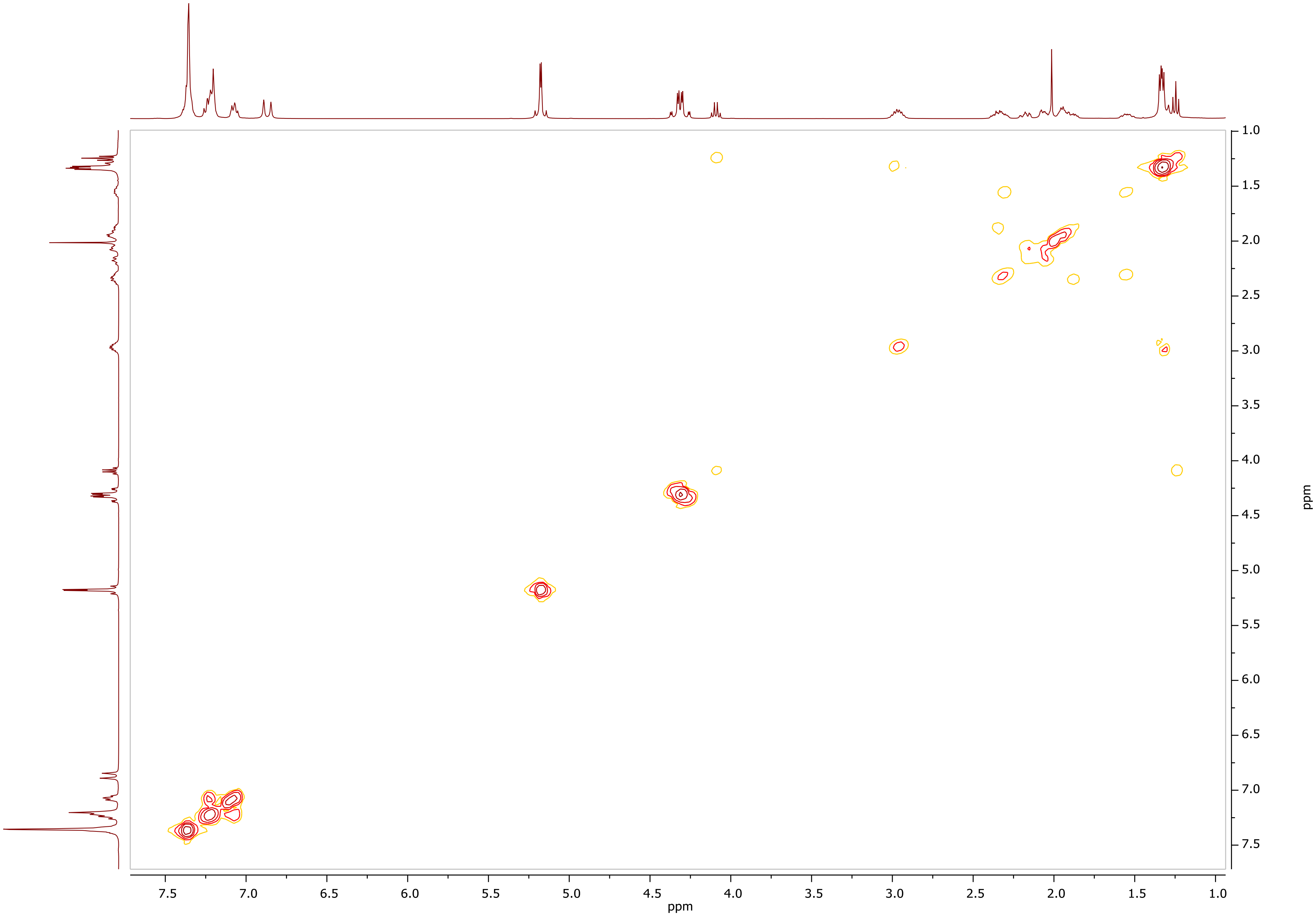

HSQC NMR of **8** (400.13 MHz, $CDCl_3$)

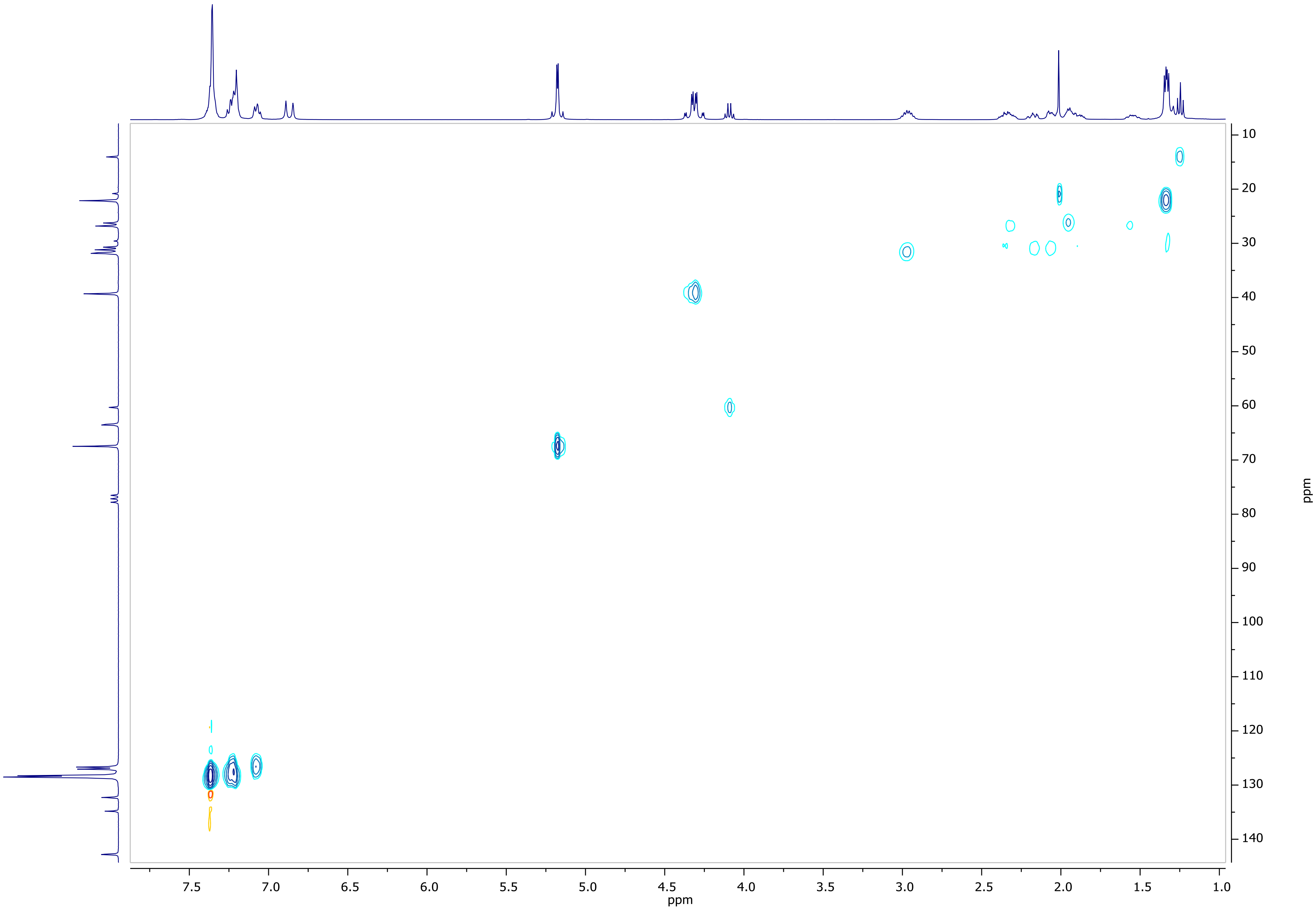

HMBC NMR of **8** (400.13 MHz, $CDCl_3$)

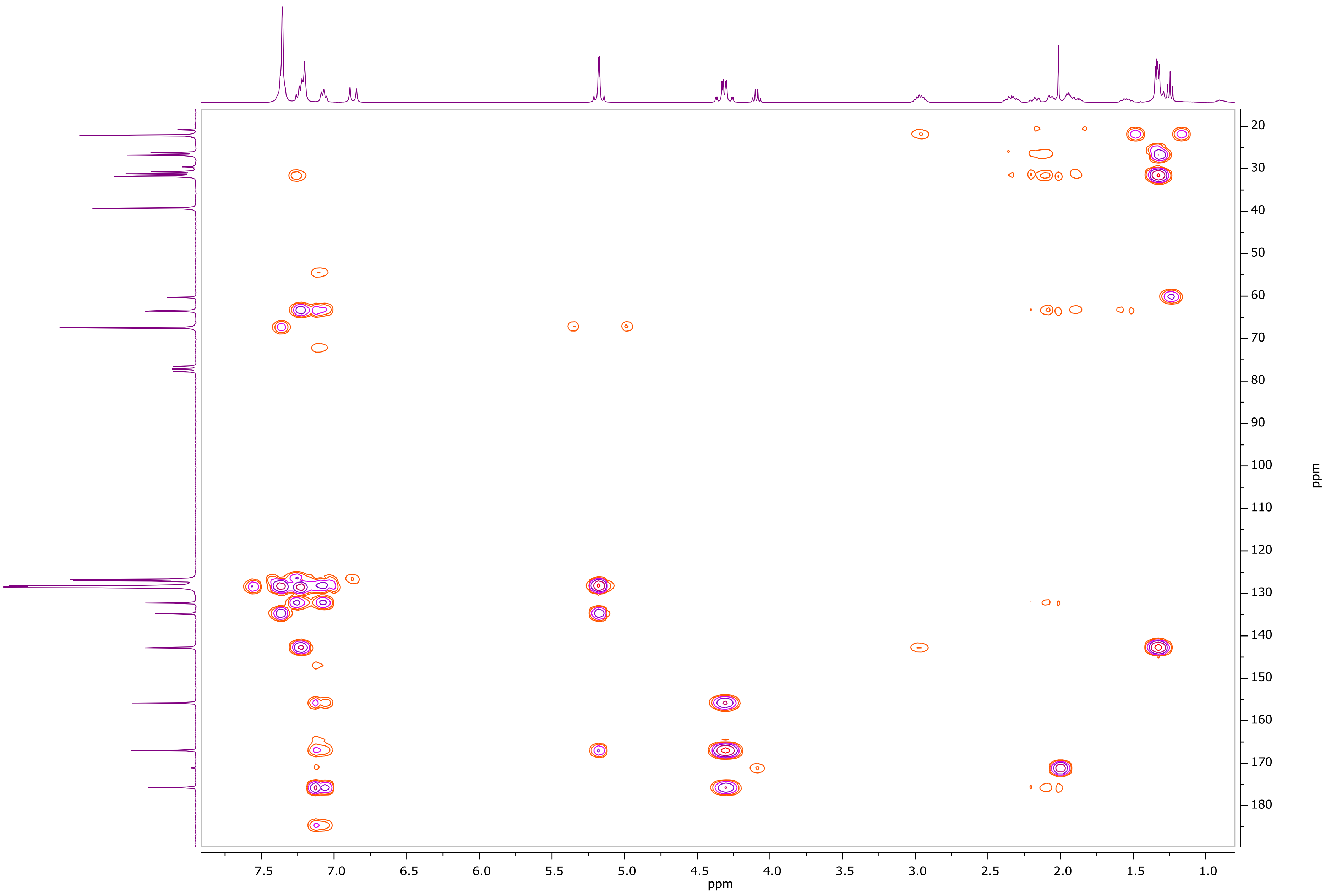

^{1}H NMR of **10** (600.11 MHz, $CDCl_3$)

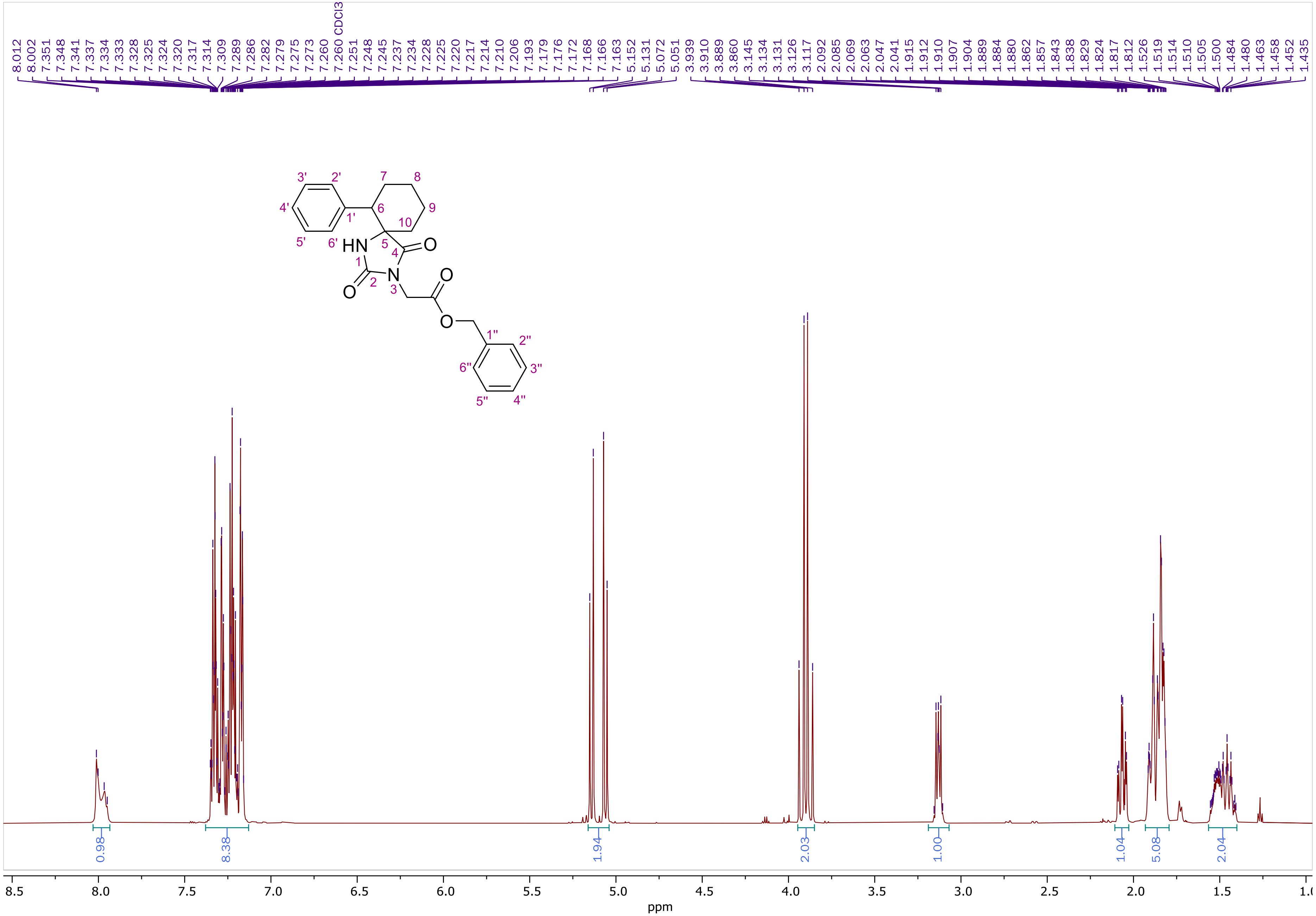

^{13}C NMR of **10** (150.9 MHz, $CDCl_3$)

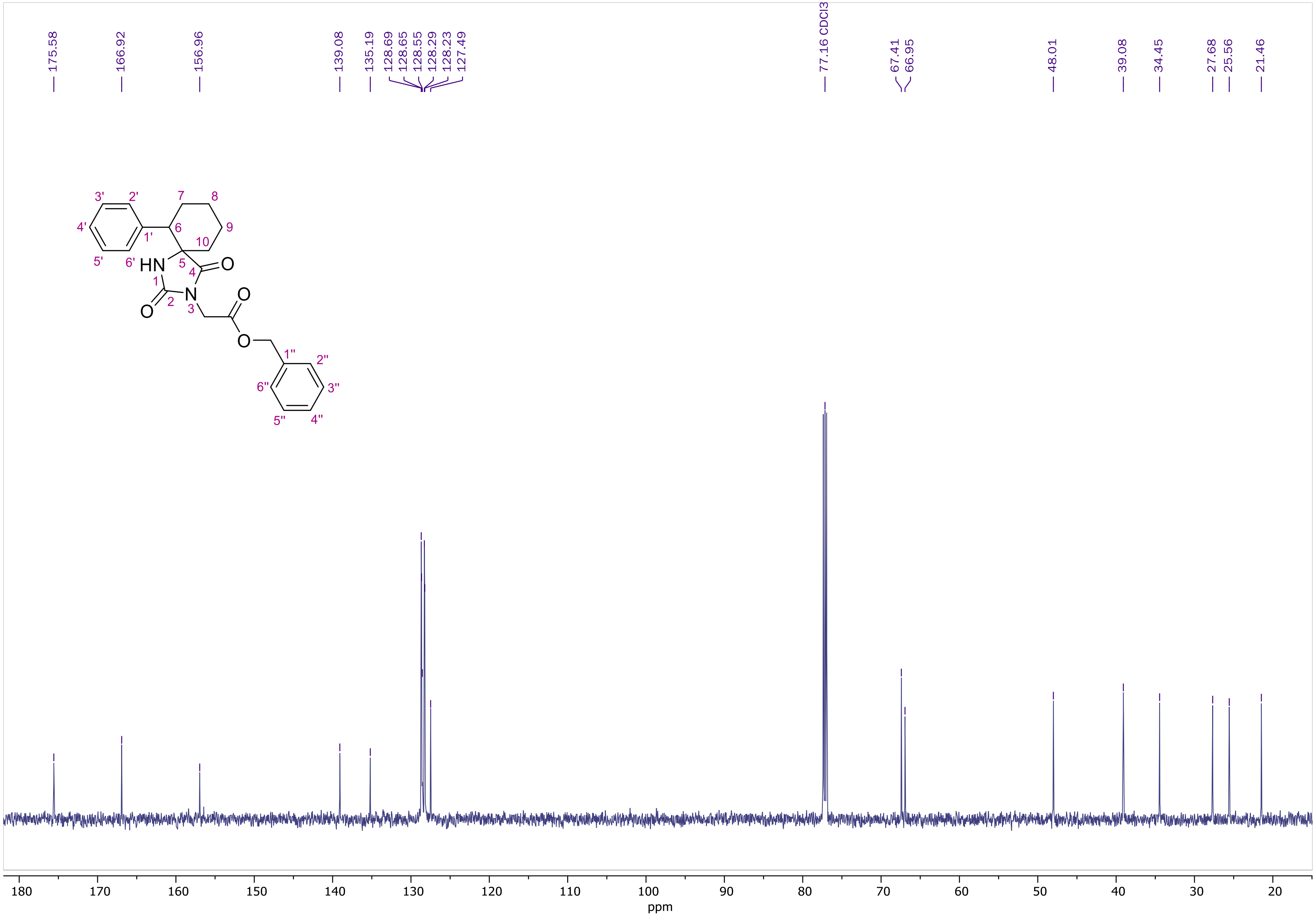

COSY NMR of **10** (400.13 MHz, $CDCl_3$)

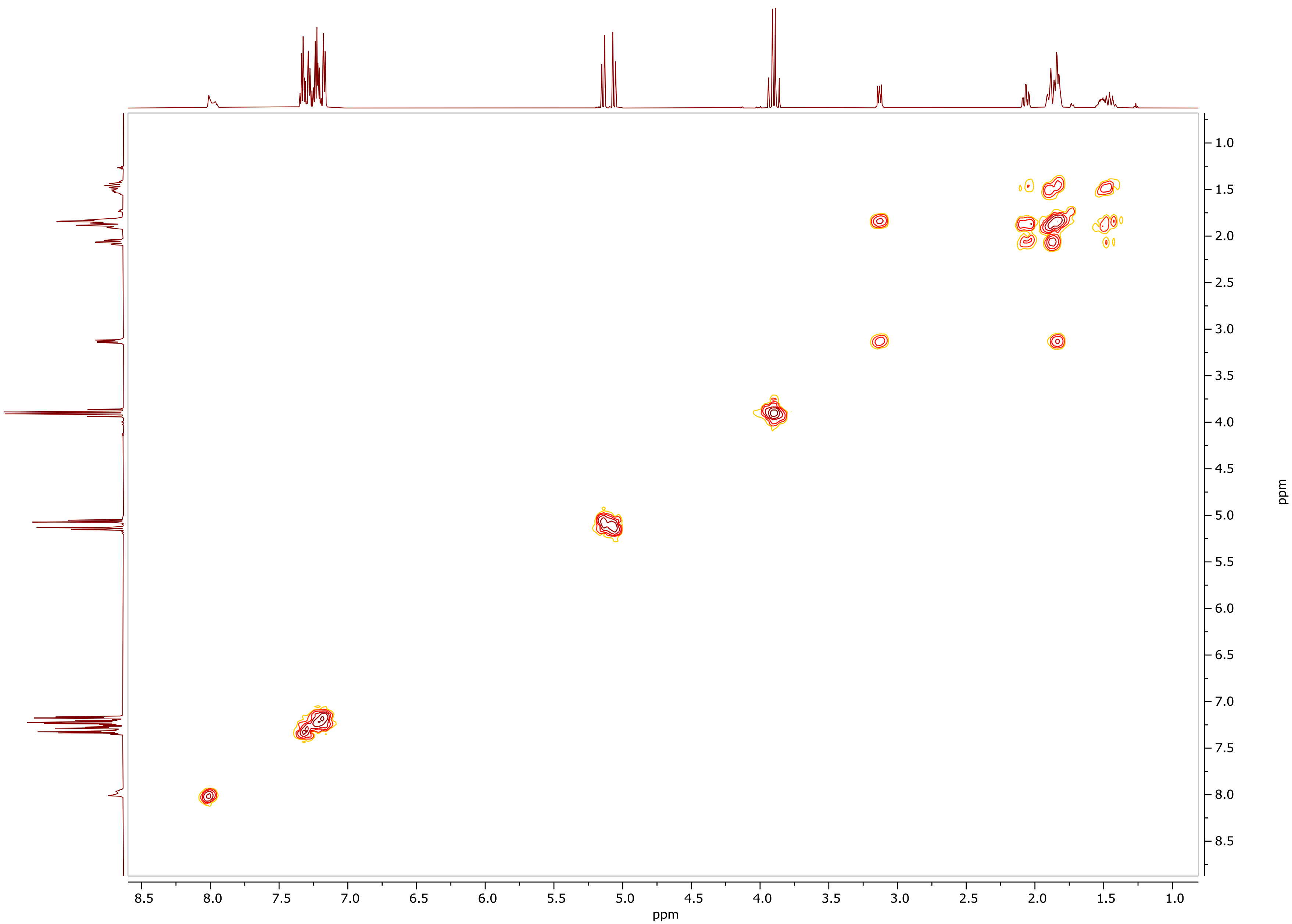

HSQC NMR of **10** (400.13 MHz, $CDCl_3$)

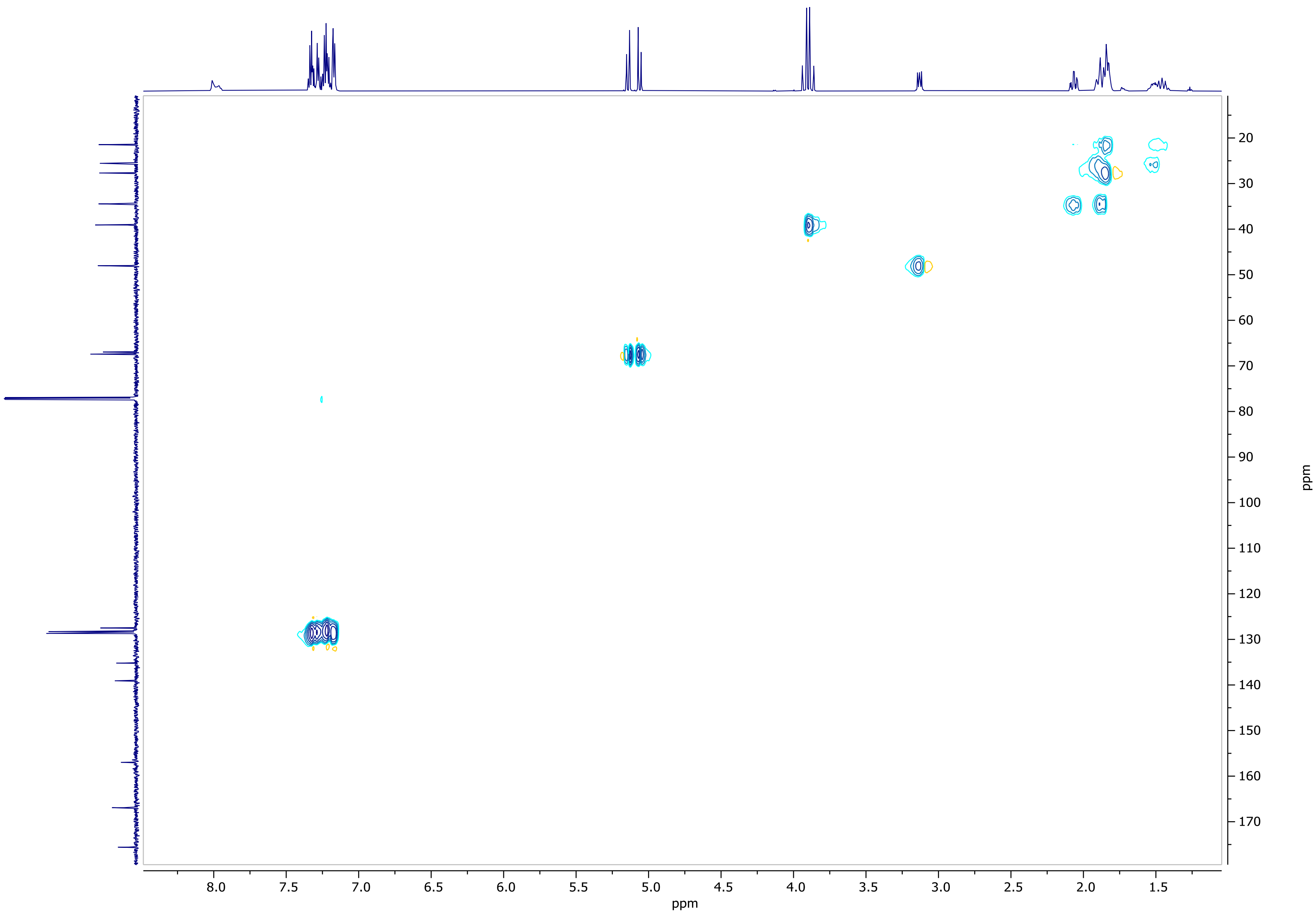

HMBC NMR of **10** (400.13 MHz, $CDCl_3$)

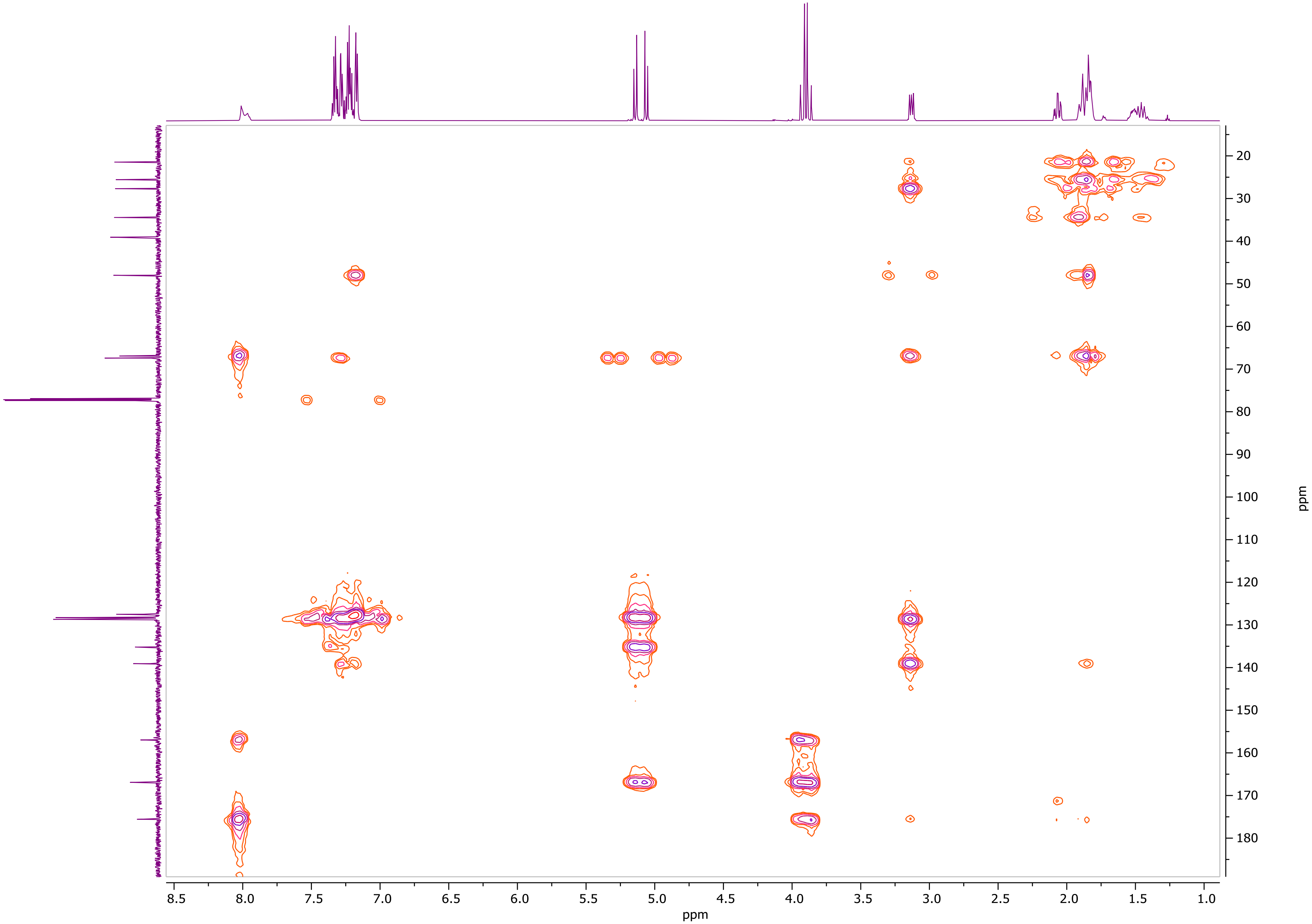

^1H NMR of **11** (600.11 MHz, $CDCl_3$)

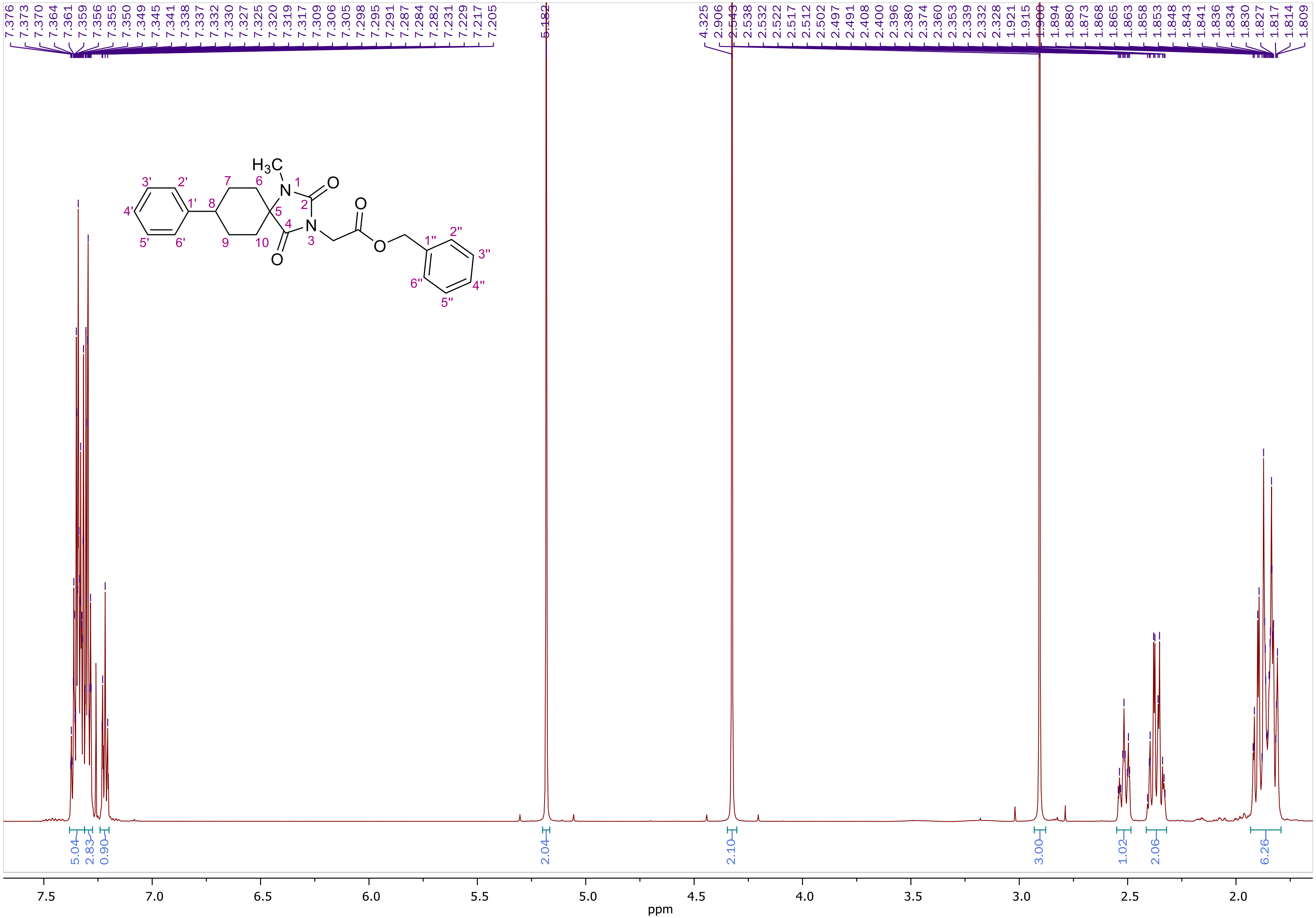

^{13}C NMR of **11** (150.9 MHz, $CDCl_3$)

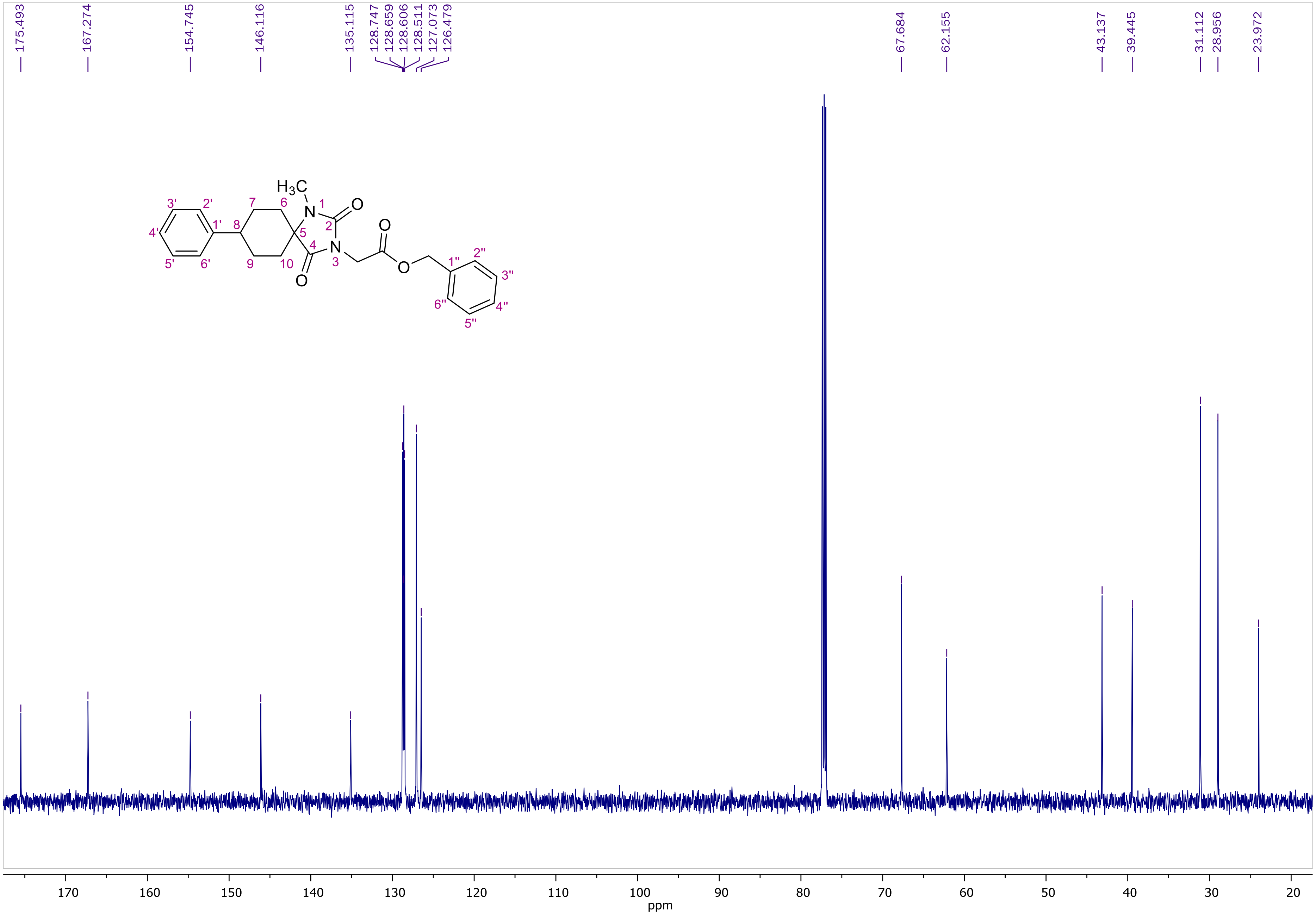

COSY NMR of **11** (600.11 MHz, $CDCl_3$)

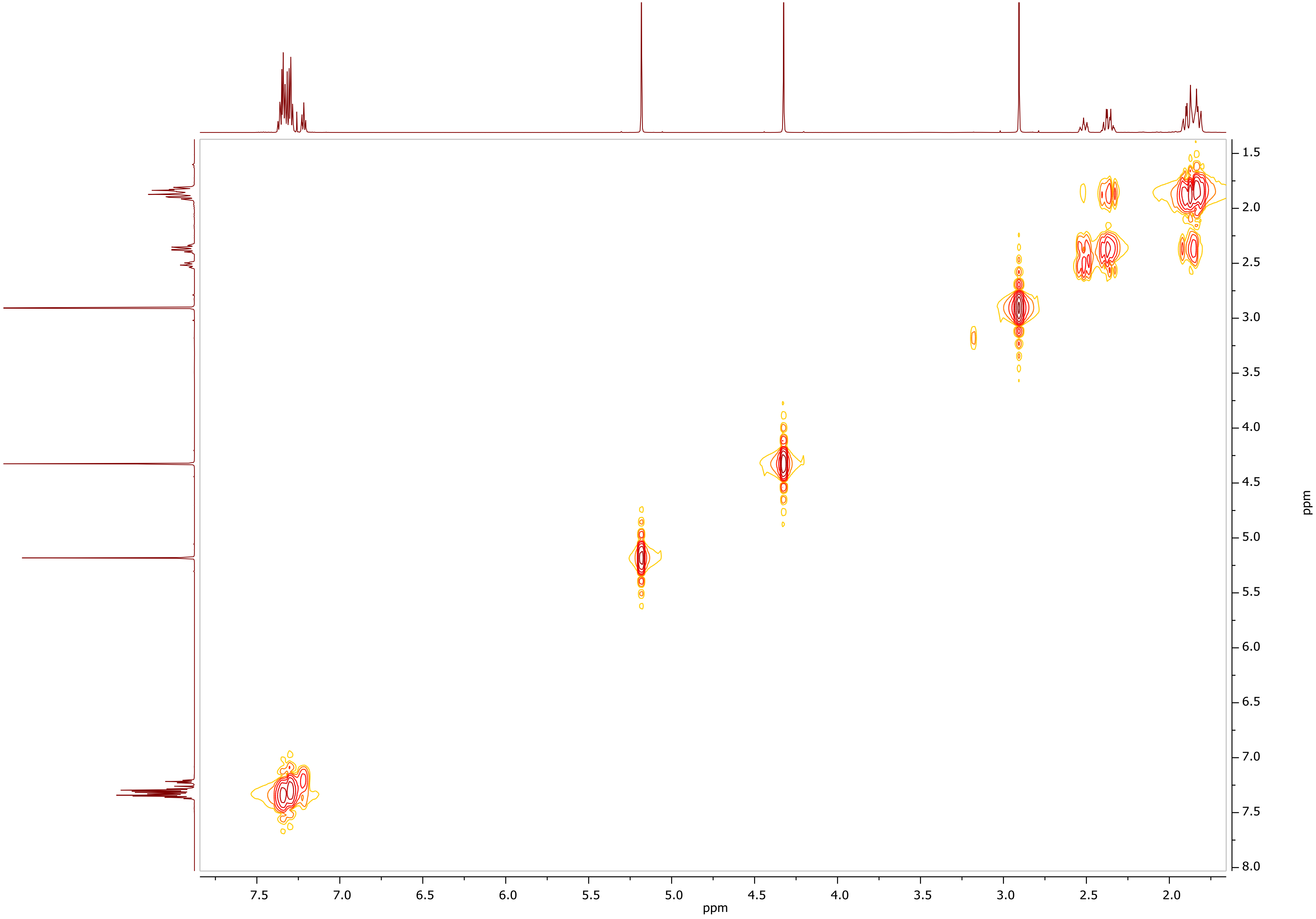

HSQC-DEPT NMR of **11** (600.11 MHz, $CDCl_3$)

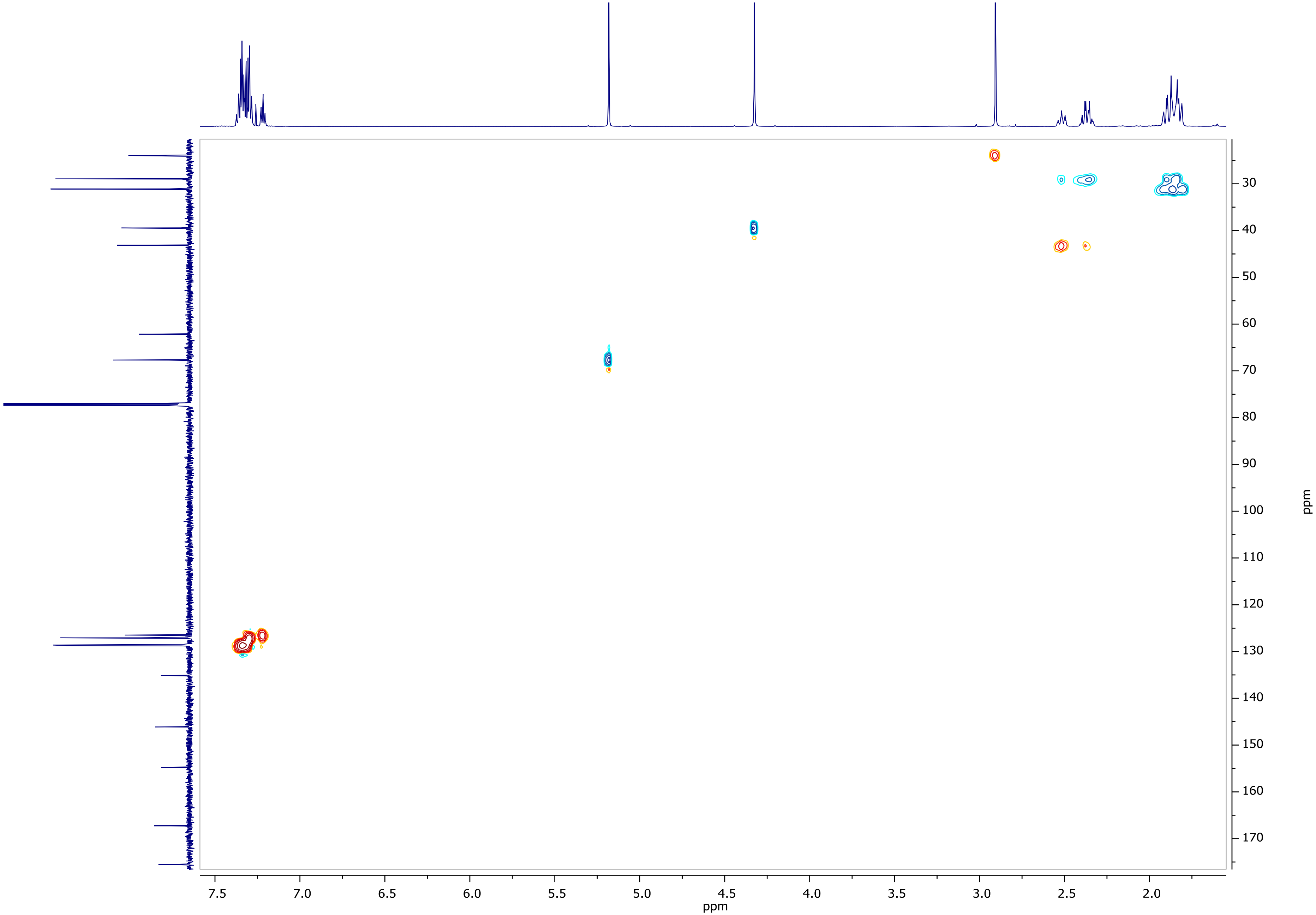

HMBC NMR of **11** (600.11 MHz, $CDCl_3$)

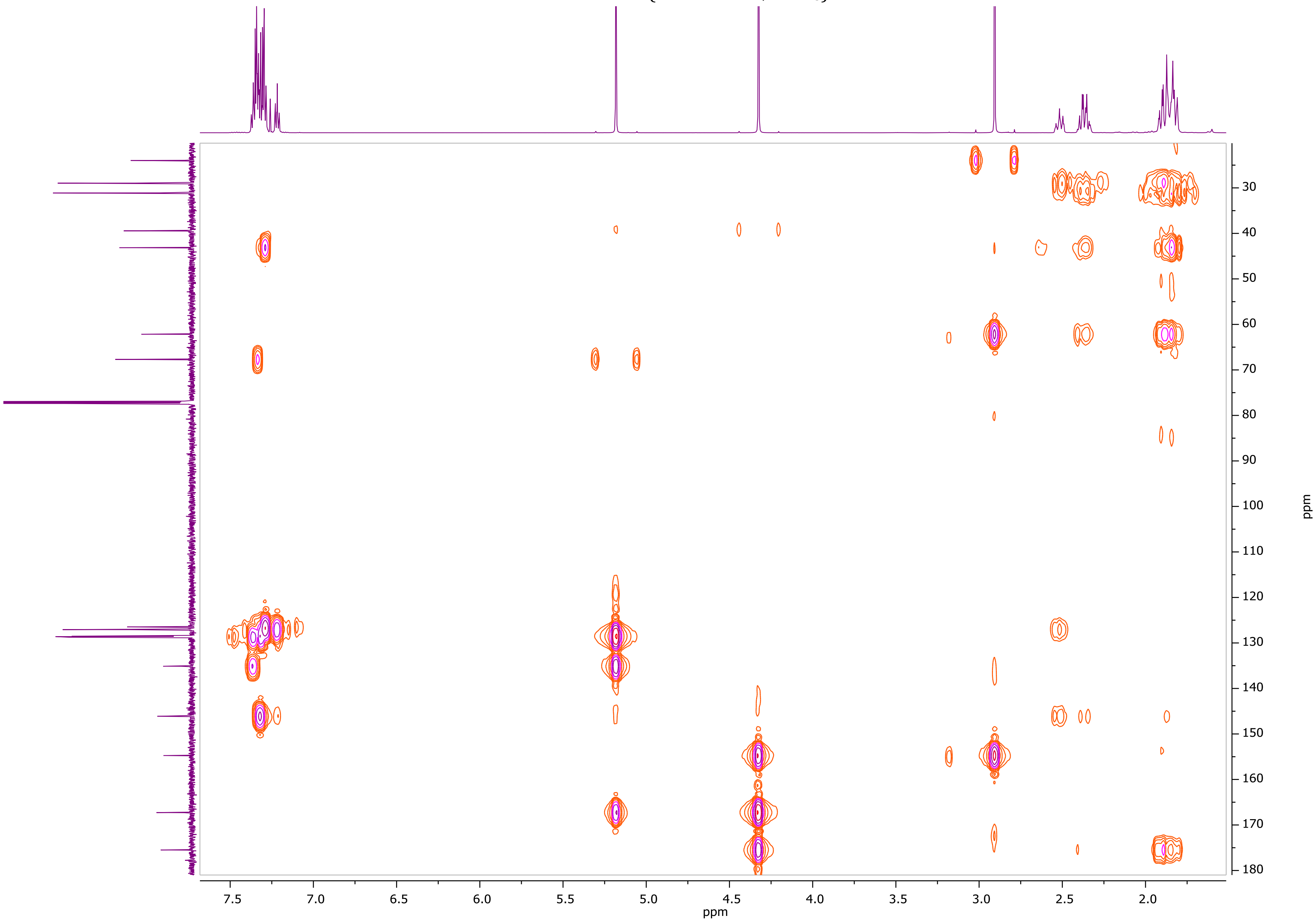

^{1}H NMR of **12** (600.11 MHz, $CDCl_3$)

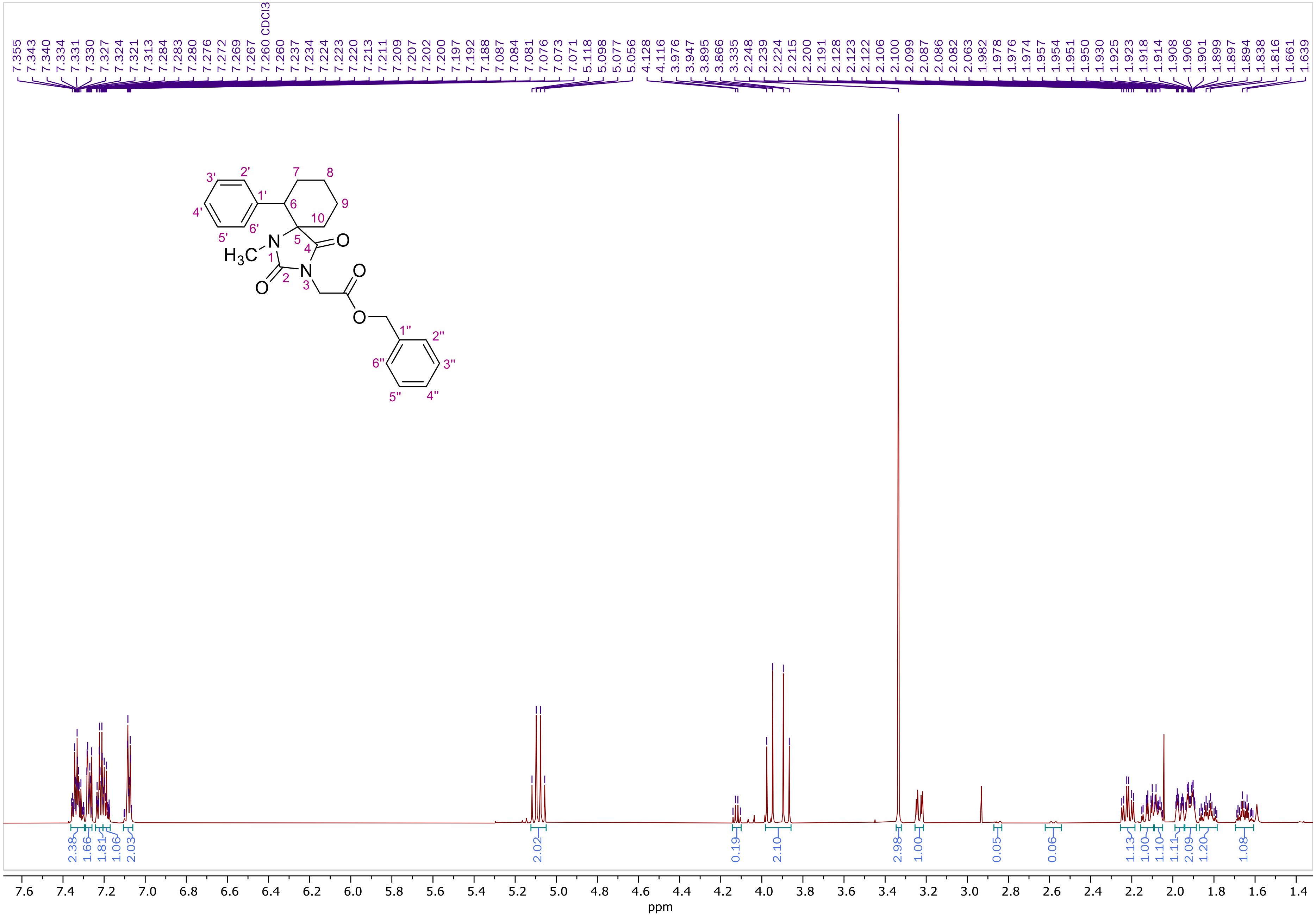

^{13}C NMR of **12** (150.9 MHz, $CDCl_3$)

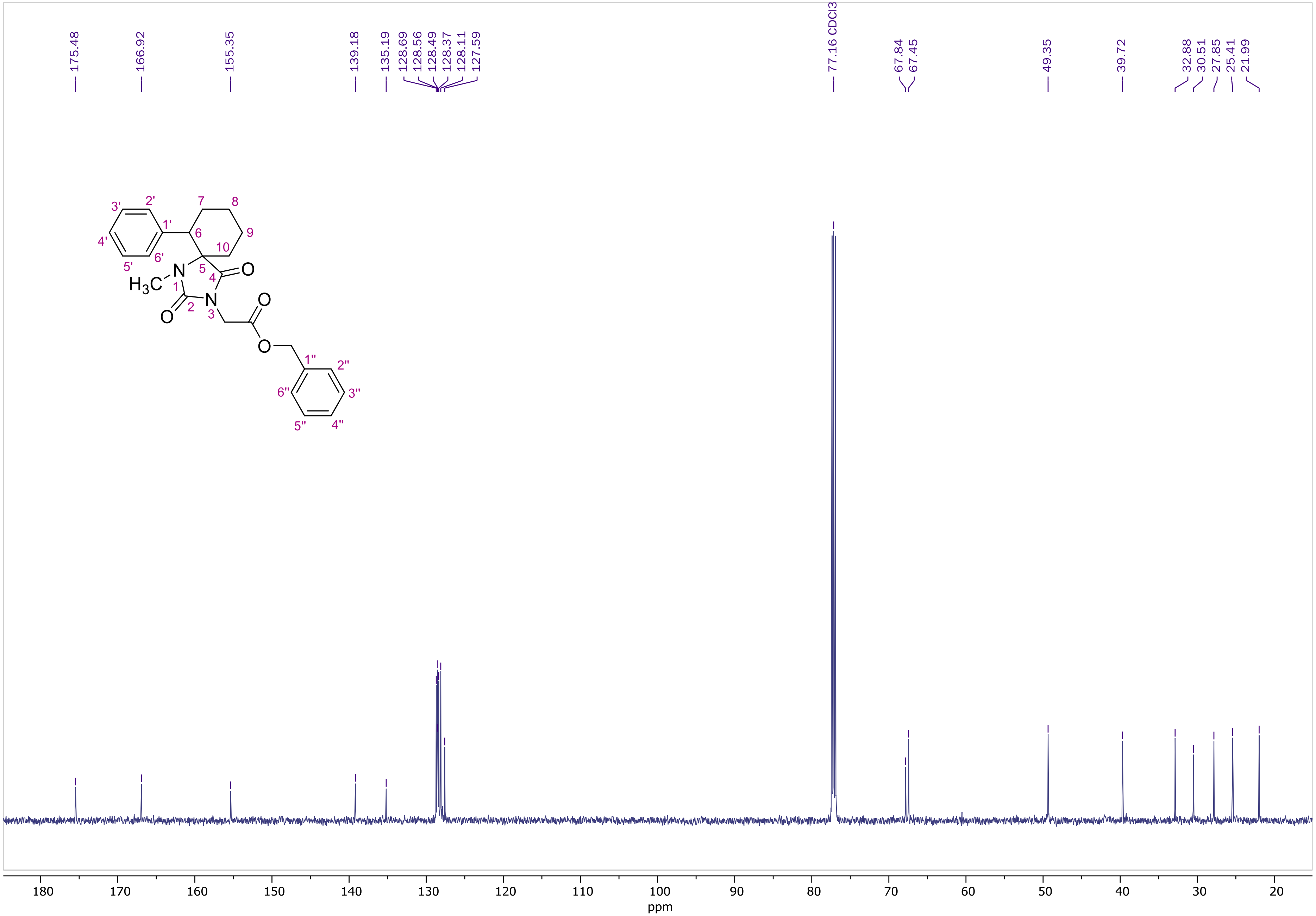

COSY NMR of **12** (400.13 MHz, $CDCl_3$)

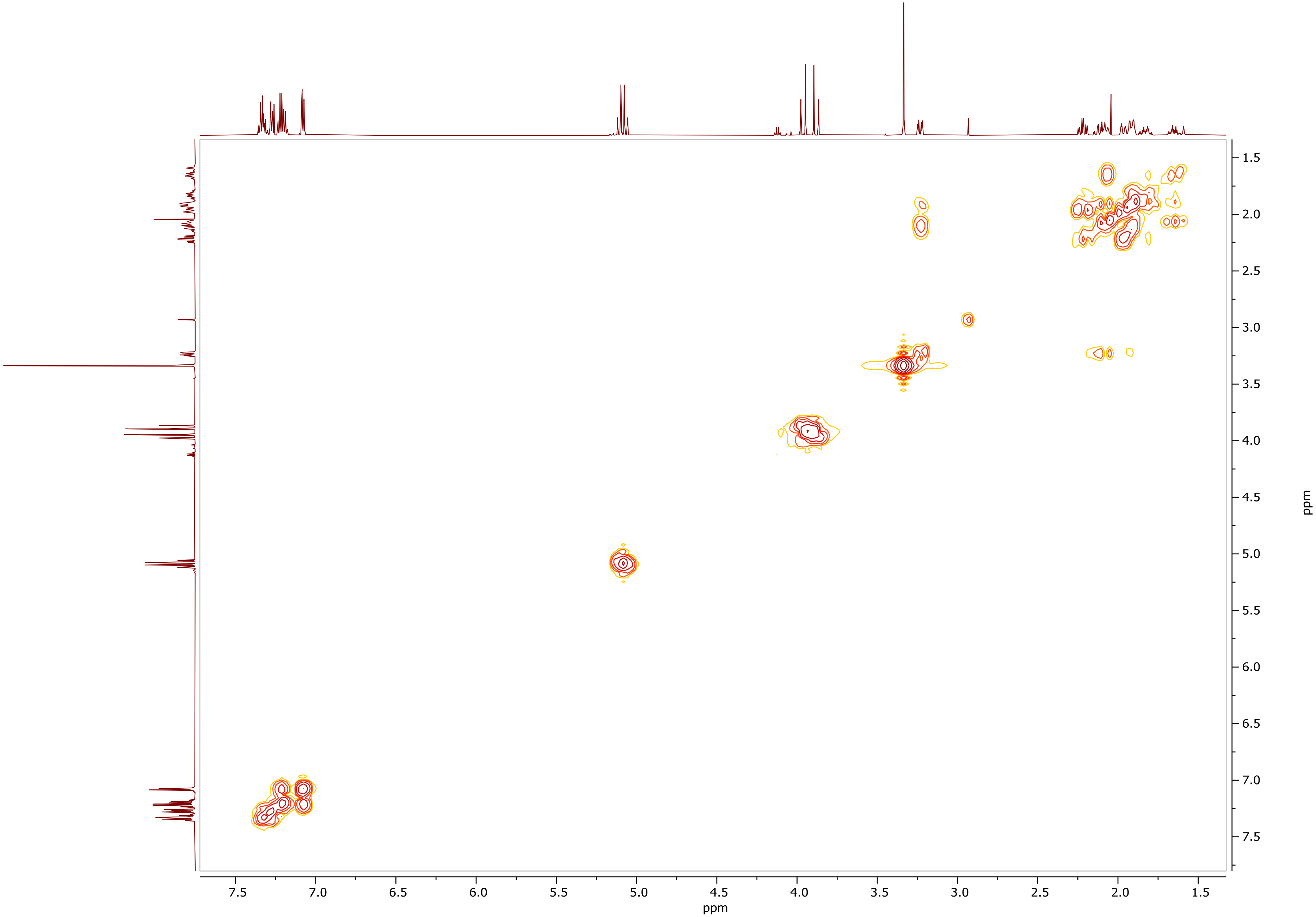

HSQC NMR of **12** (400.13 MHz, $CDCl_3$)

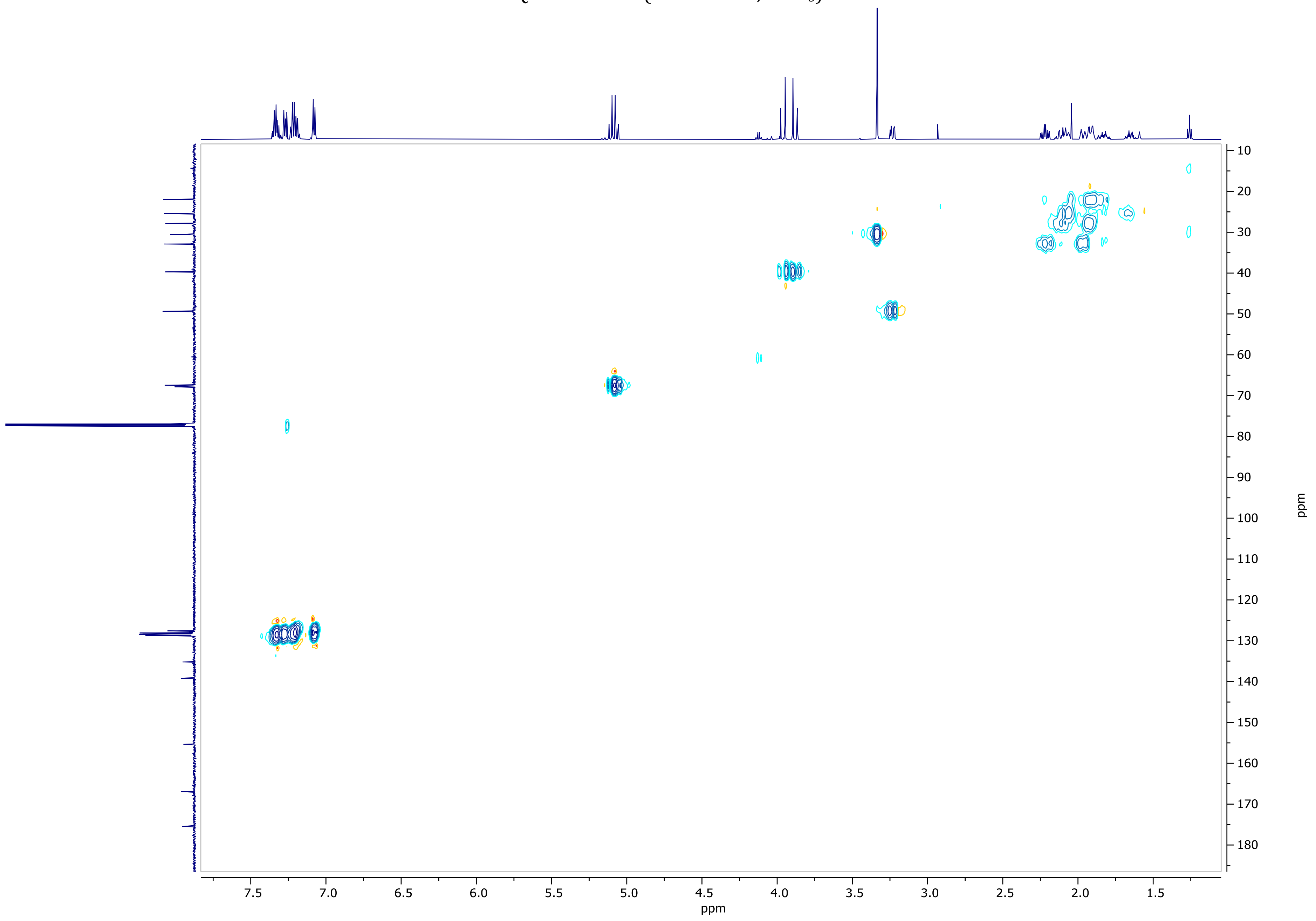

HMBC NMR of **12** (400.13 MHz, $CDCl_3$)

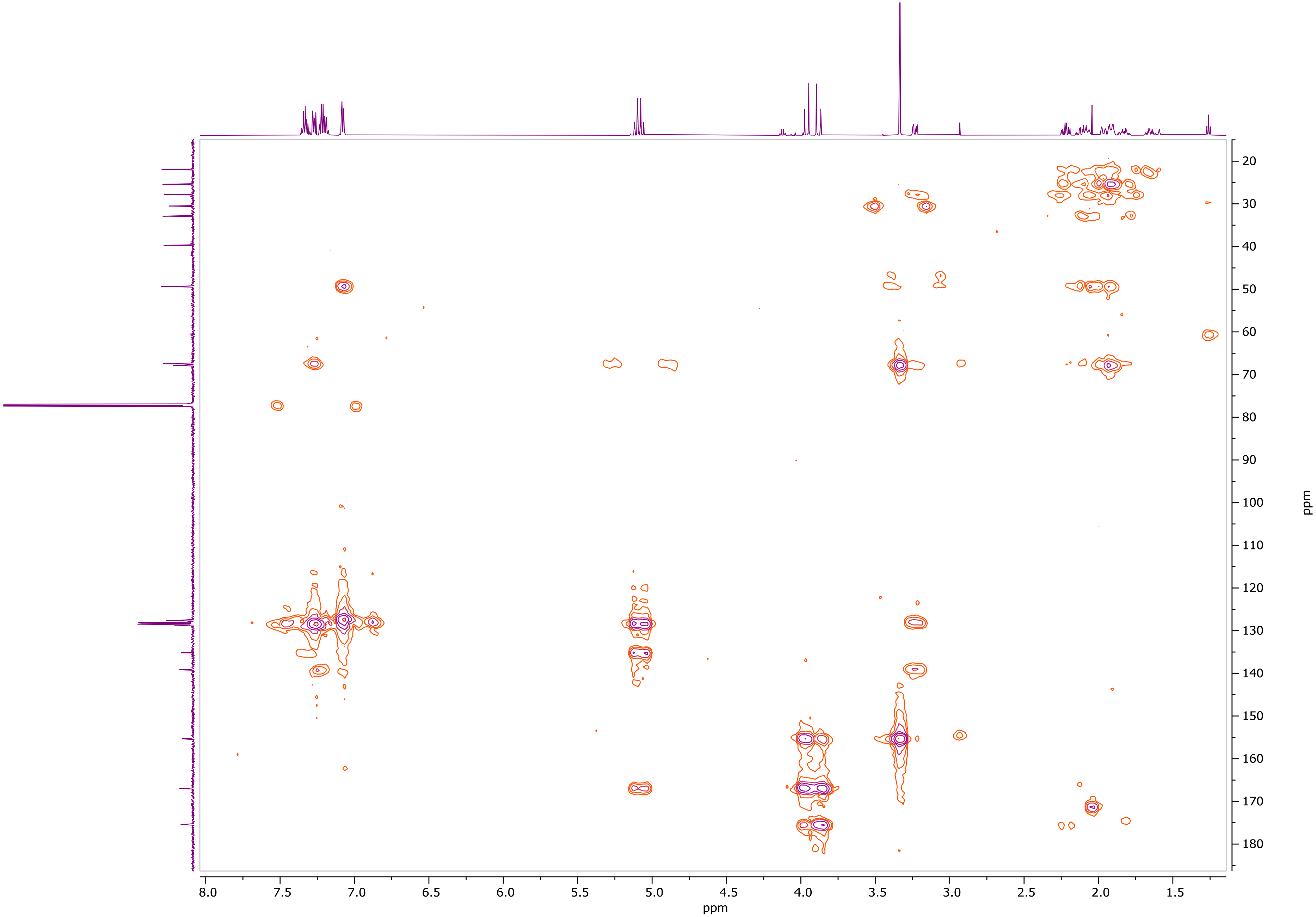

^{1}H NMR of **14** (600.11 MHz, $CDCl_3$)

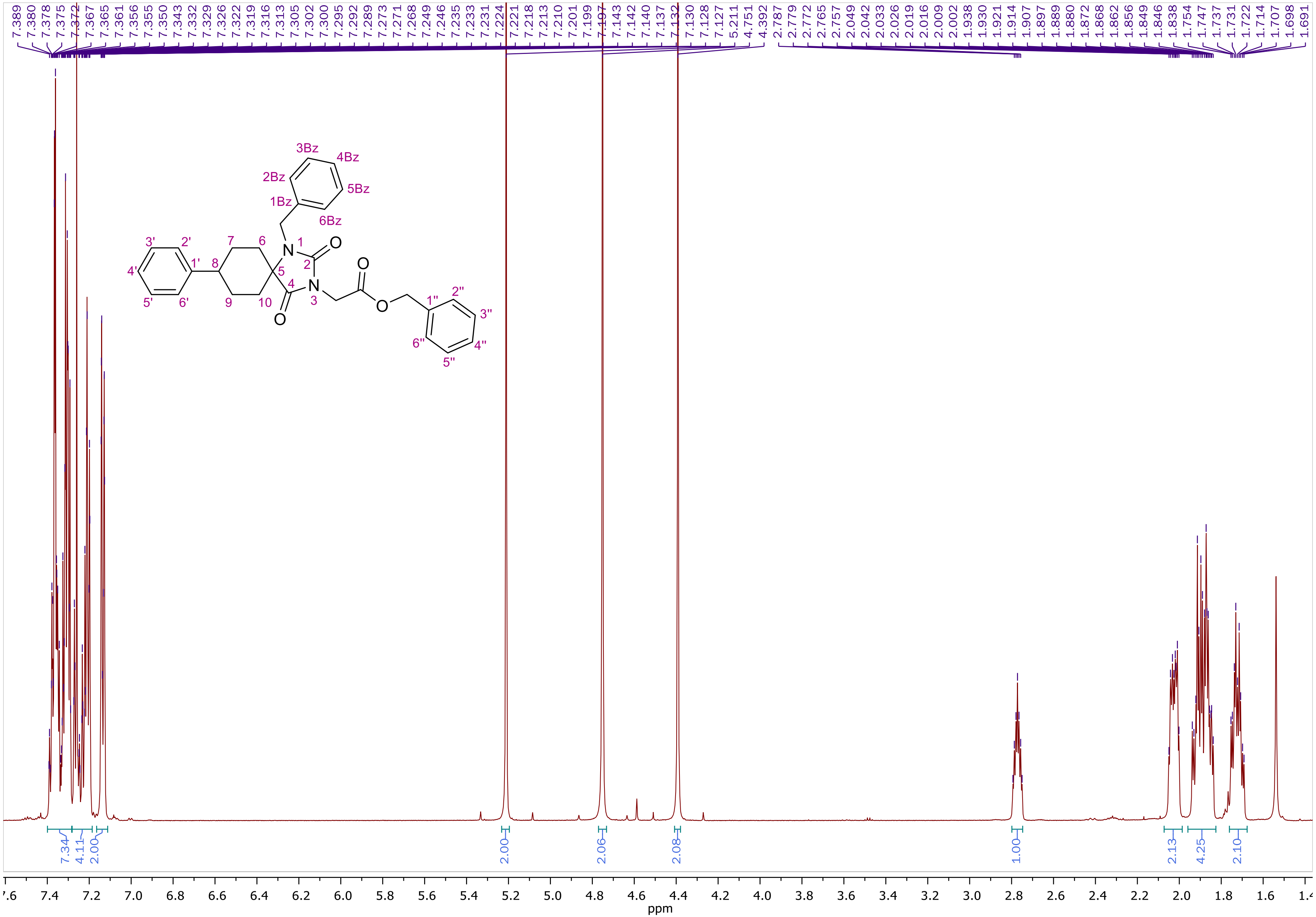

^{13}C NMR of **14** (150.9 MHz, CDCl$_3$)

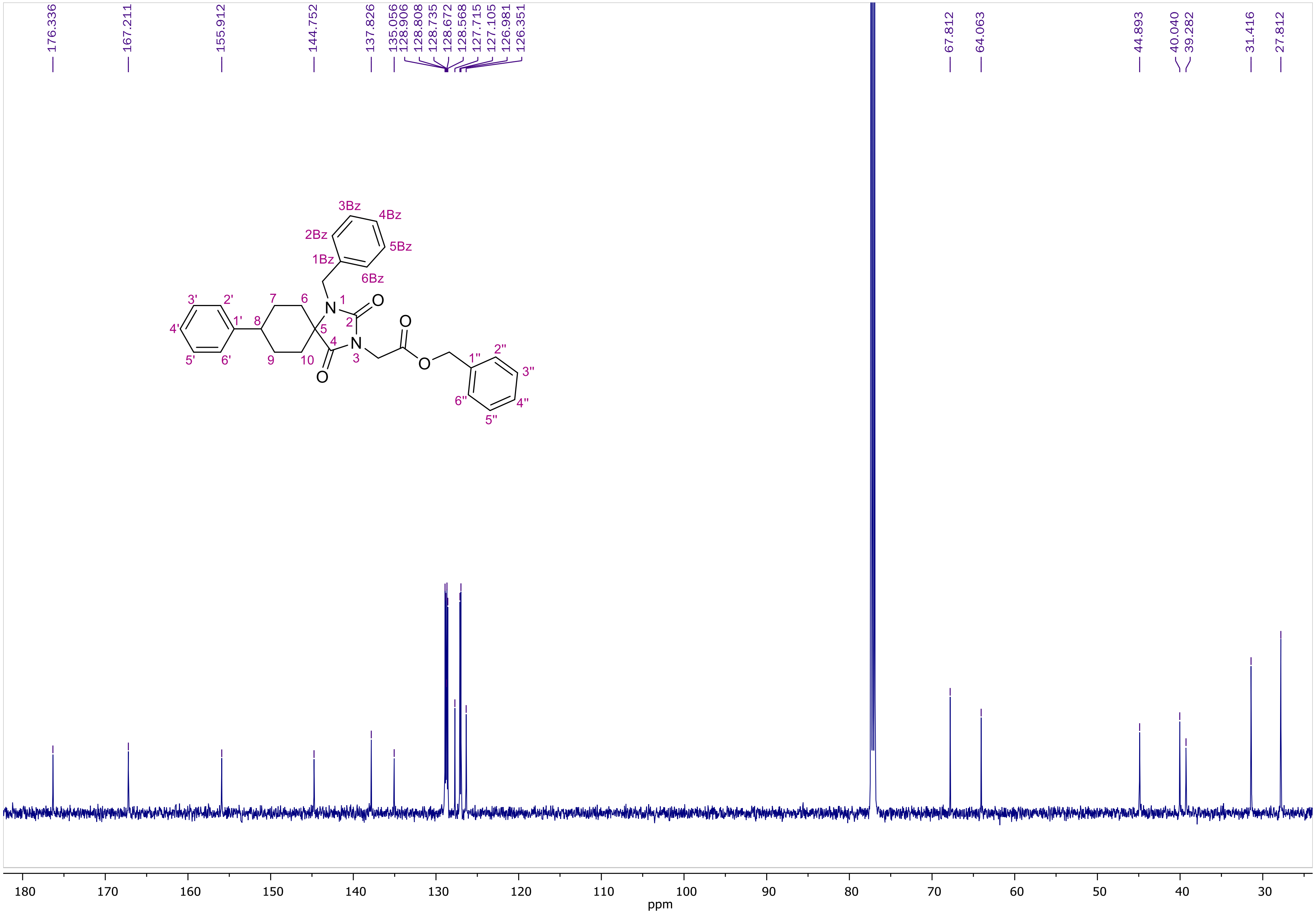

COSY NMR of **14** (600.1 MHz, $CDCl_3$)

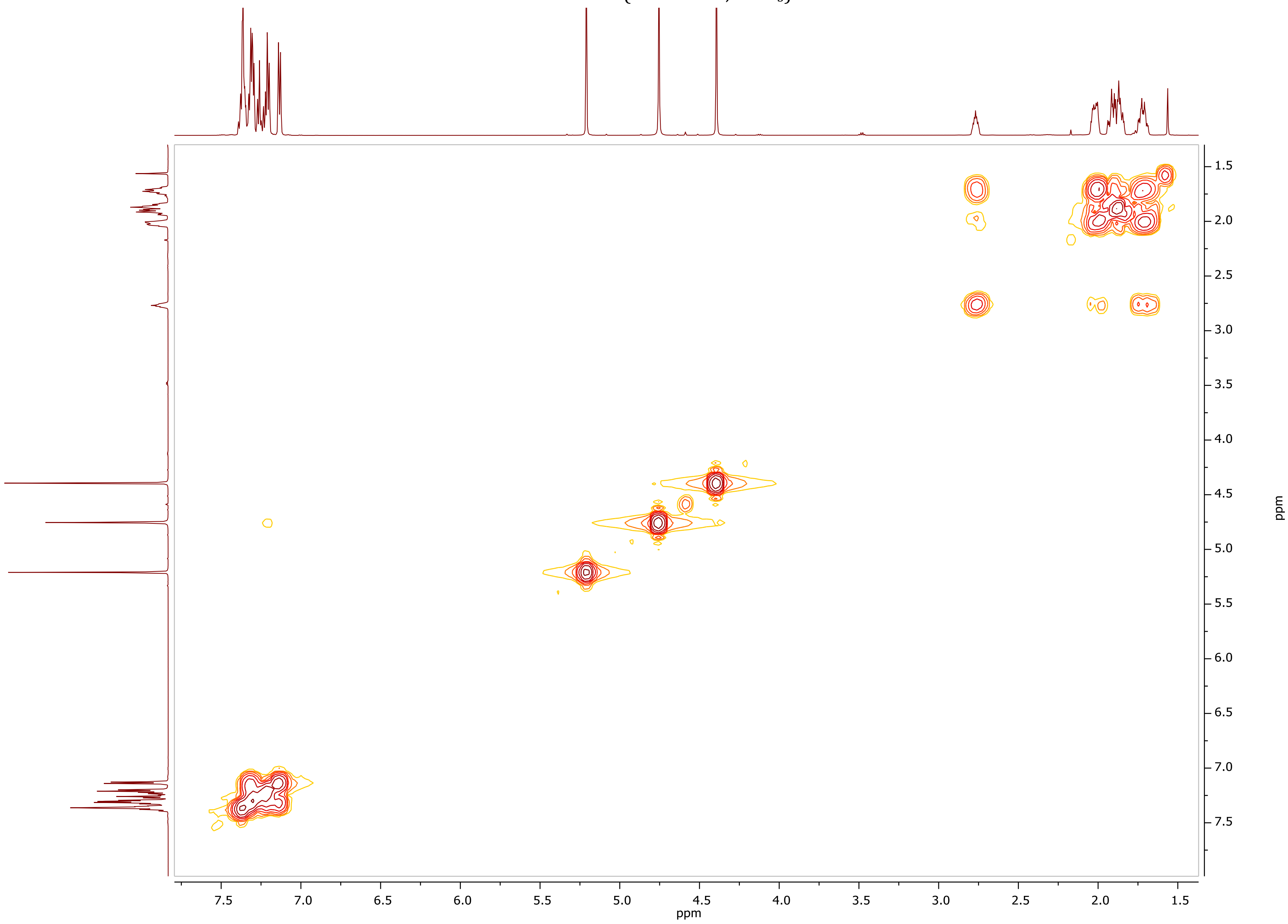

HSQC NMR of **14** (400.13 MHz, $CDCl_3$)

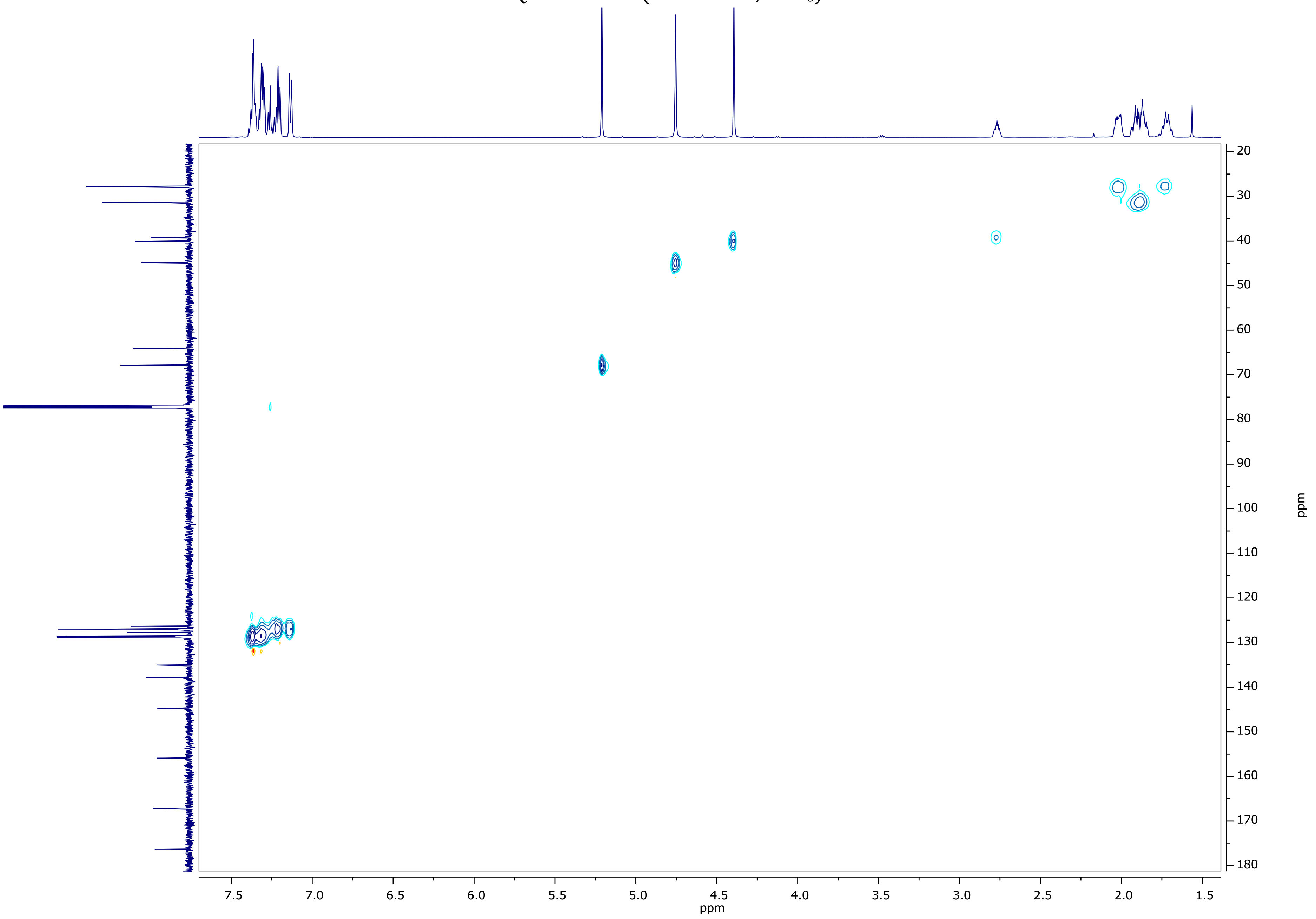

HMBC NMR of **14** (600.11 MHz, $CDCl_3$)

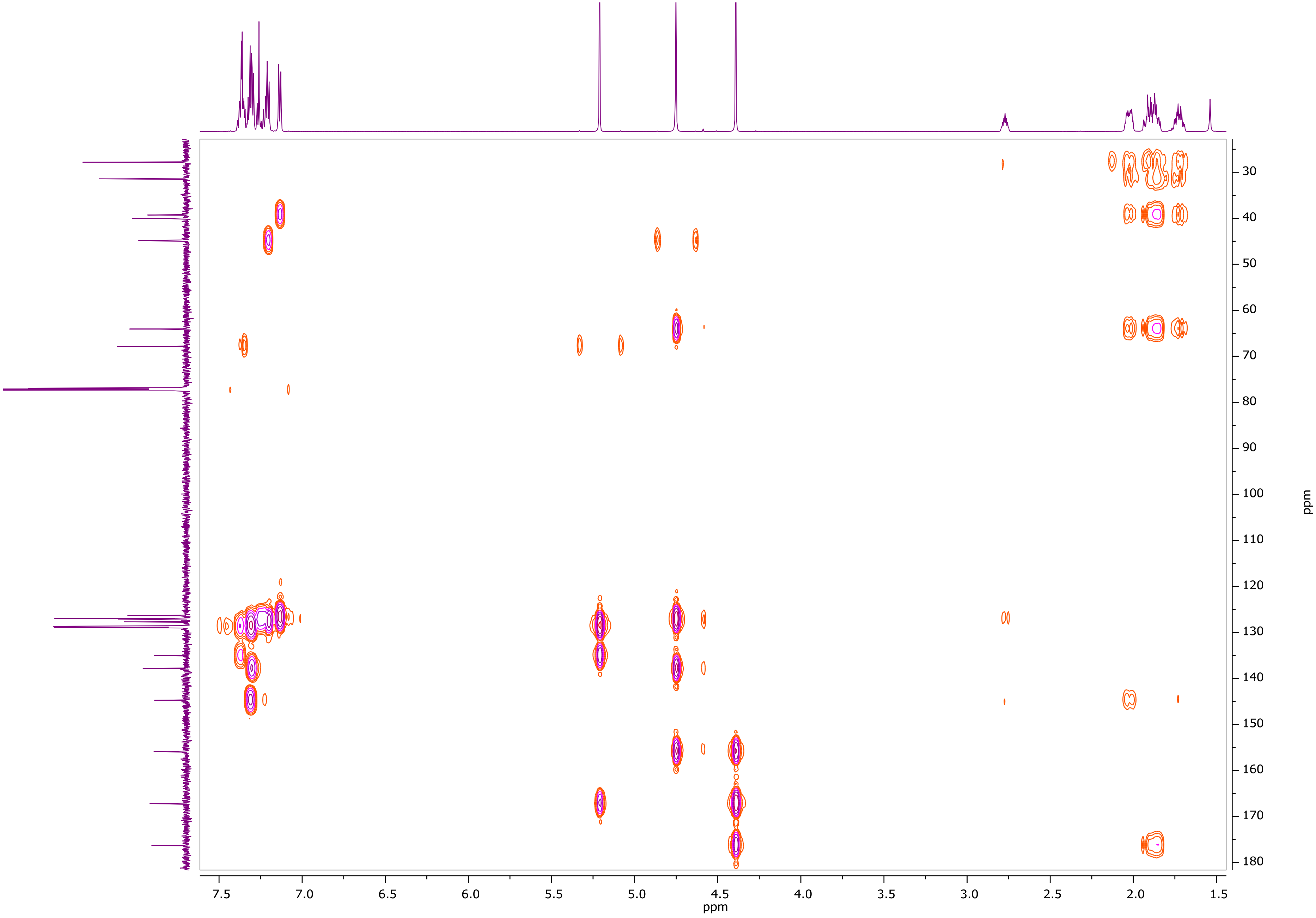

^{1}H NMR of **15** (400.13 MHz, $CDCl_3$)

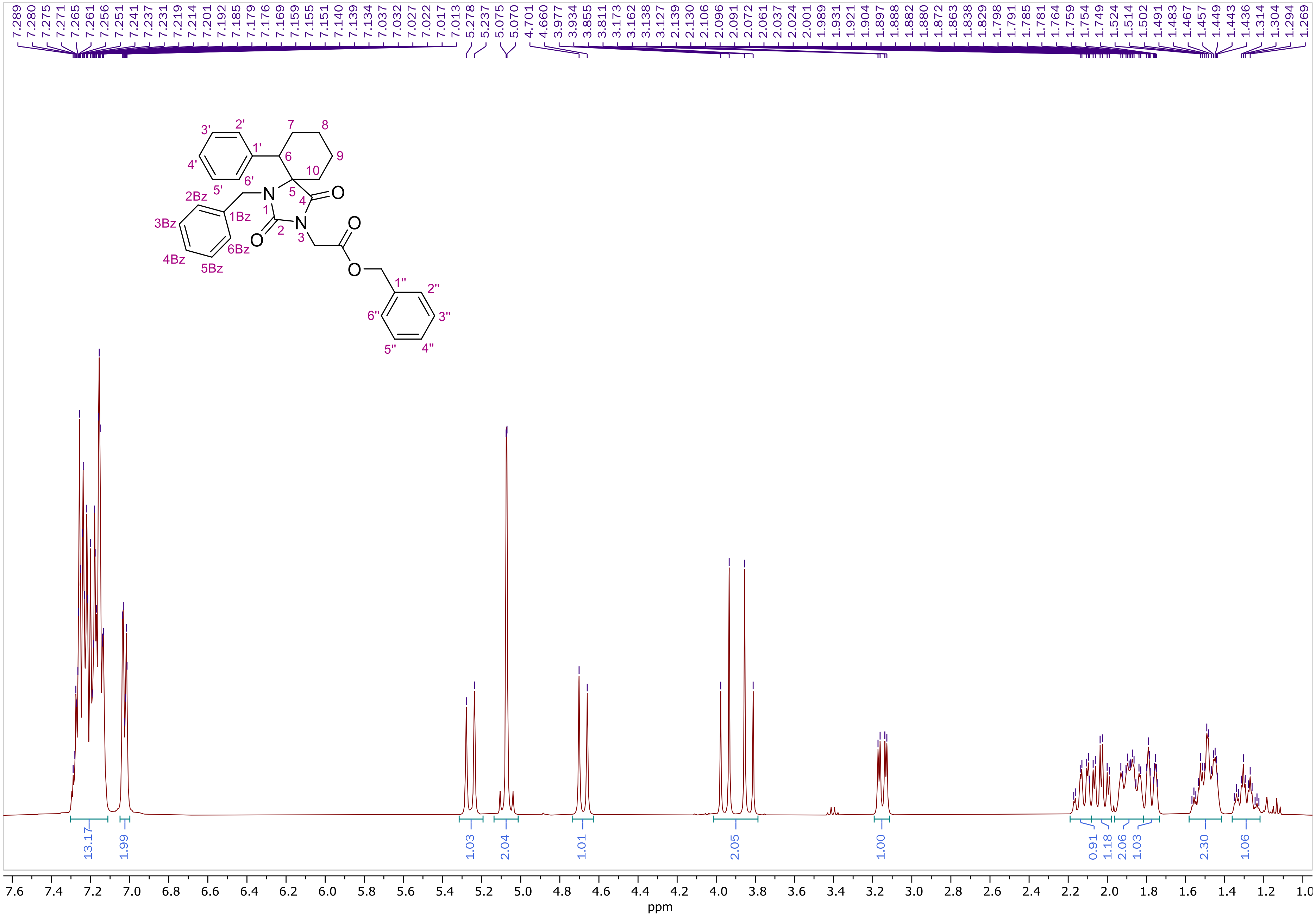

^{13}C NMR of **15** (50.32 MHz, $CDCl_3$)

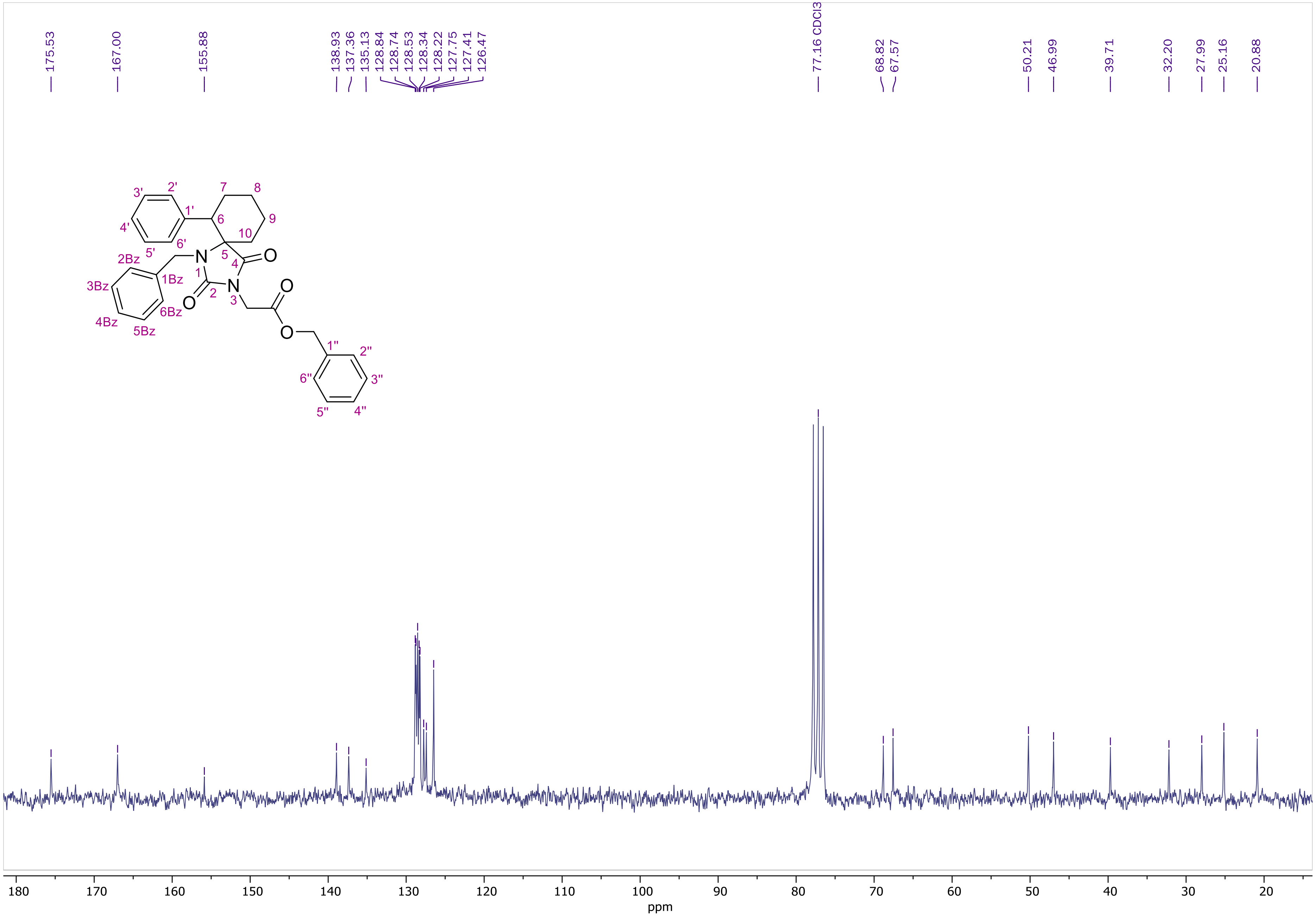

COSY NMR of **15** (400.13 MHz, $CDCl_3$)

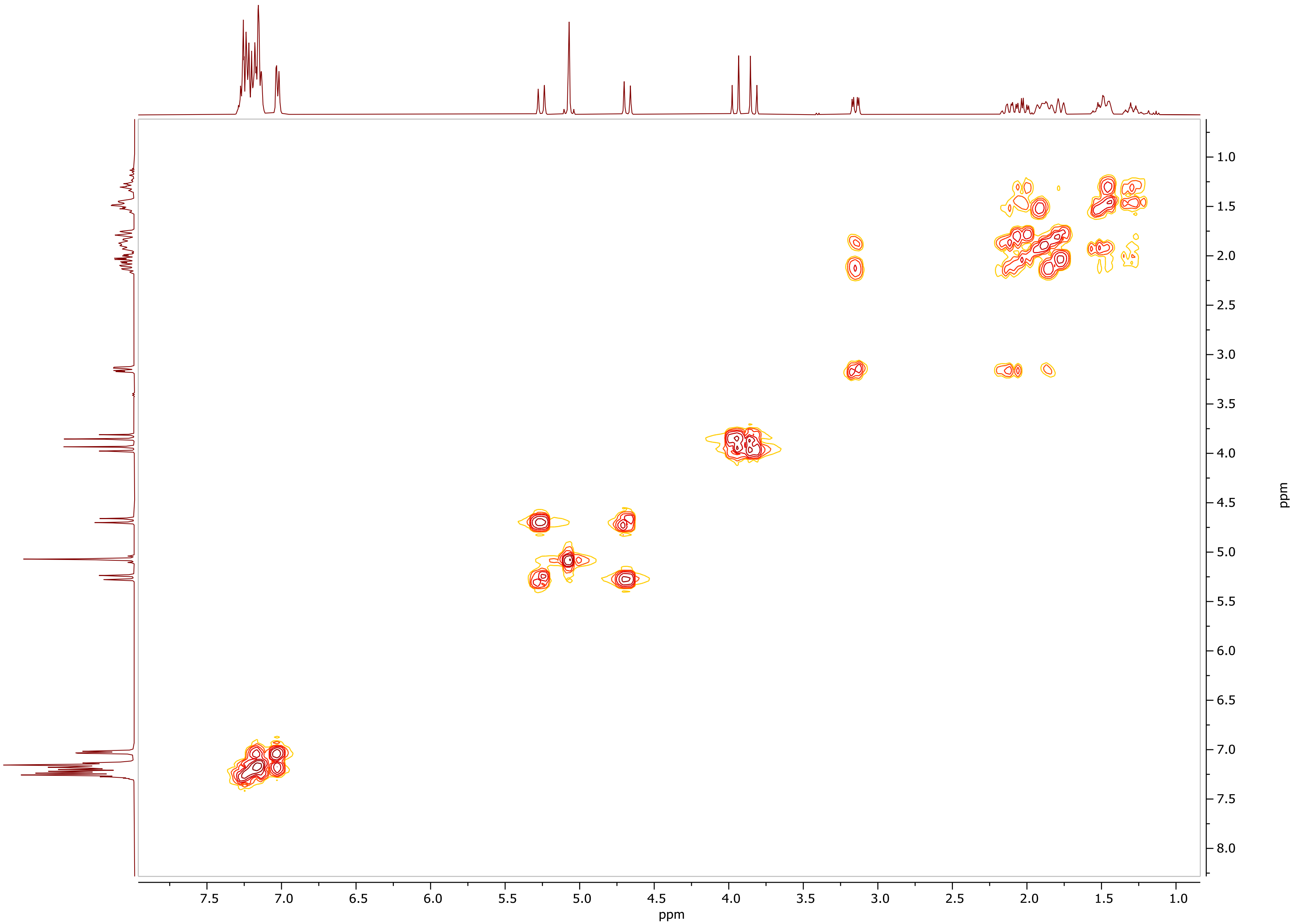

HSQC NMR of **15** (400.13 MHz, $CDCl_3$)

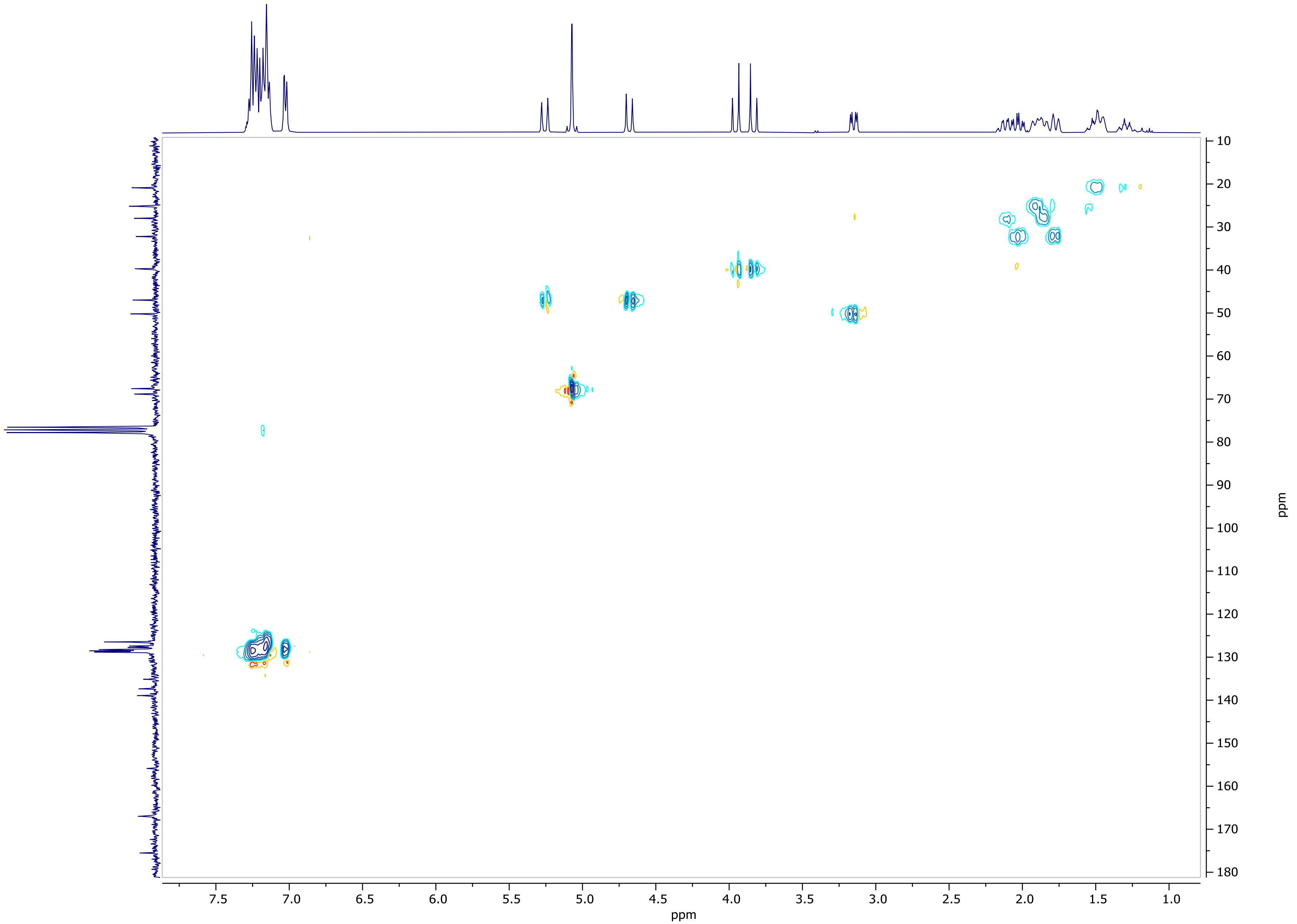

HMBC NMR of **15** (400.13 MHz, $CDCl_3$)

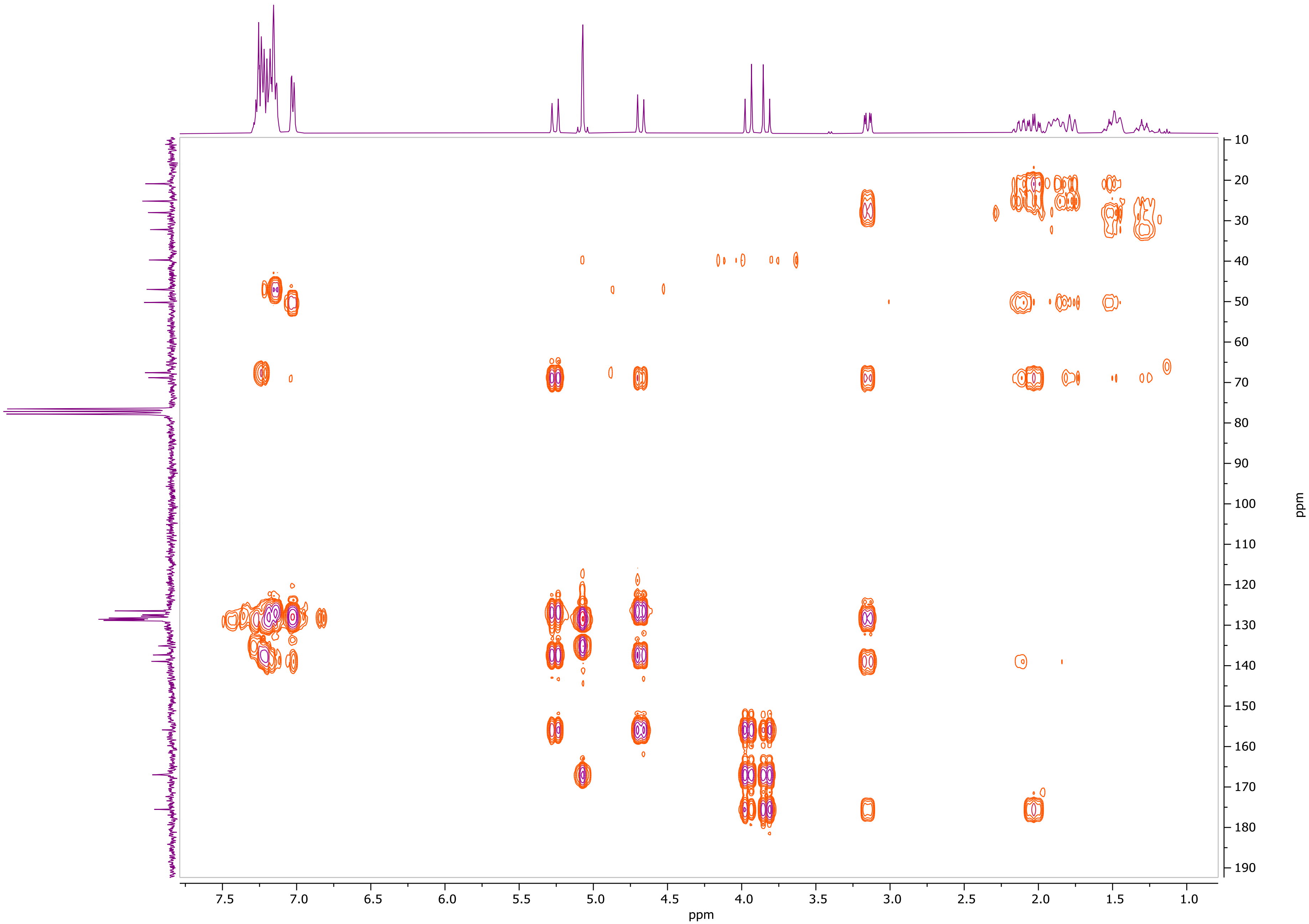

^{1}H NMR of **16** (400.13 MHz, DMSO-d_6)

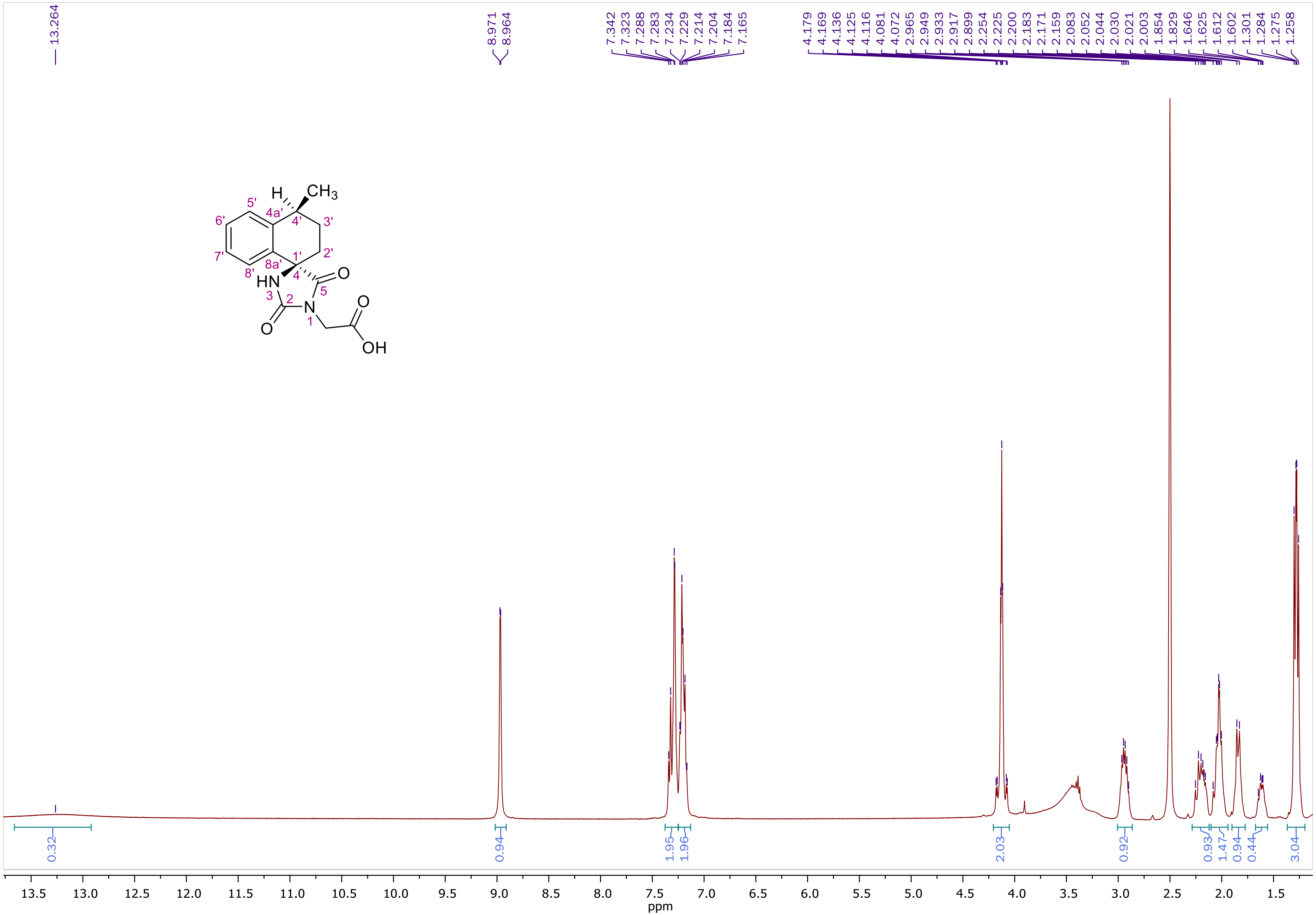

^{13}C NMR of **16** (50.32 MHz, DMSO-d_6)

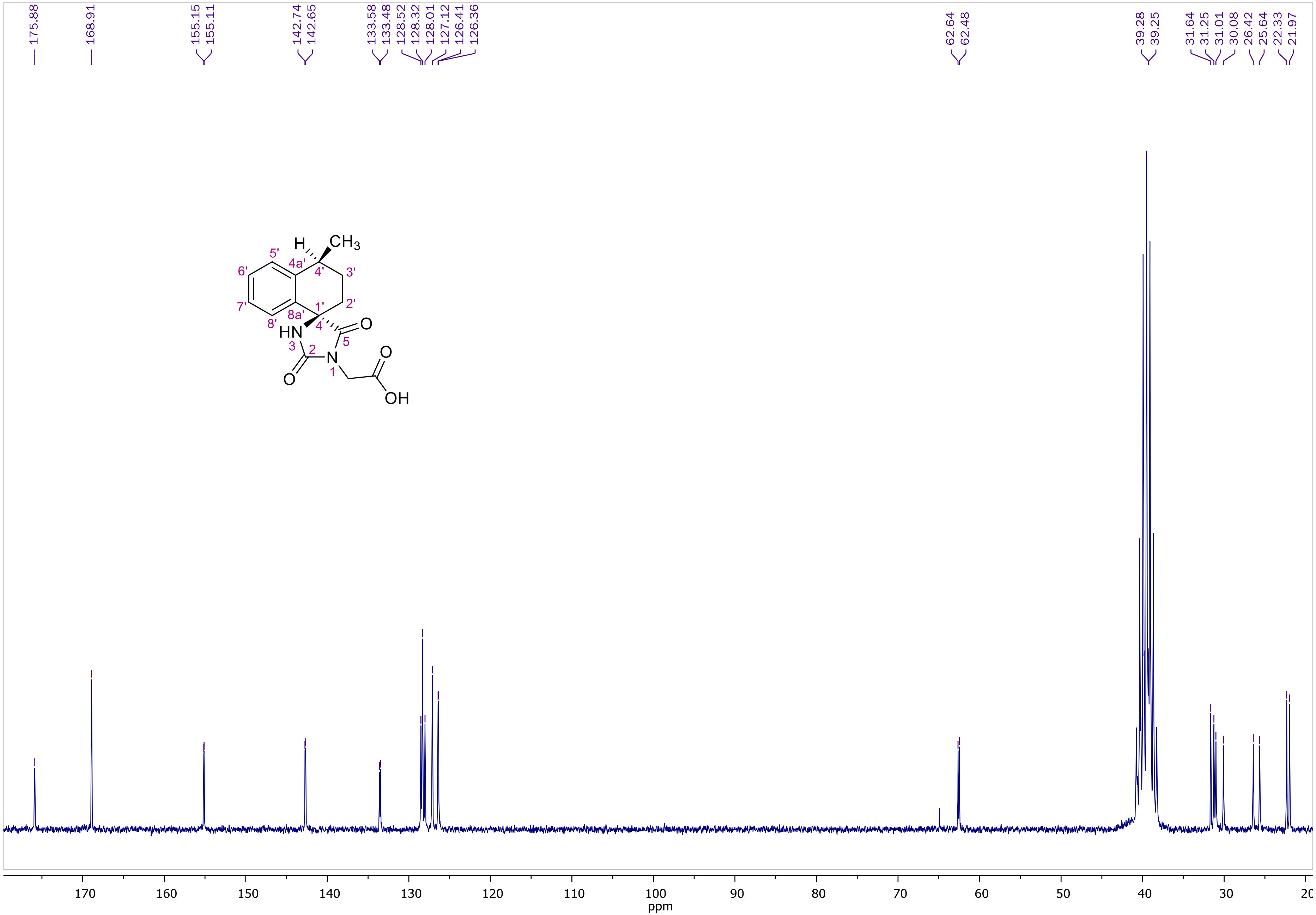

COSY NMR of **16** (400.13 MHz, DMSO-d_6)

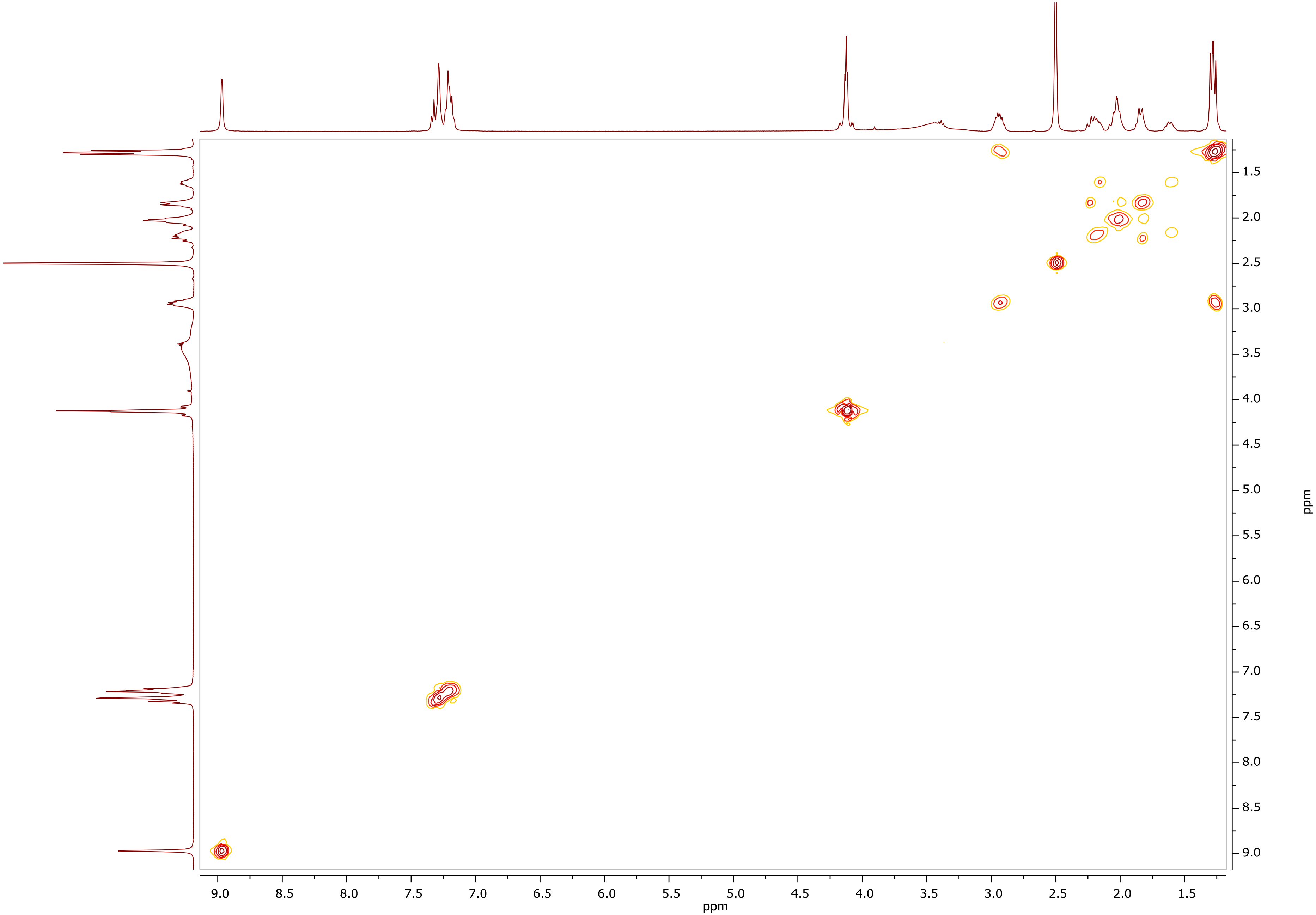

HSQC NMR of **16** (400.13 MHz, DMSO-d_6)

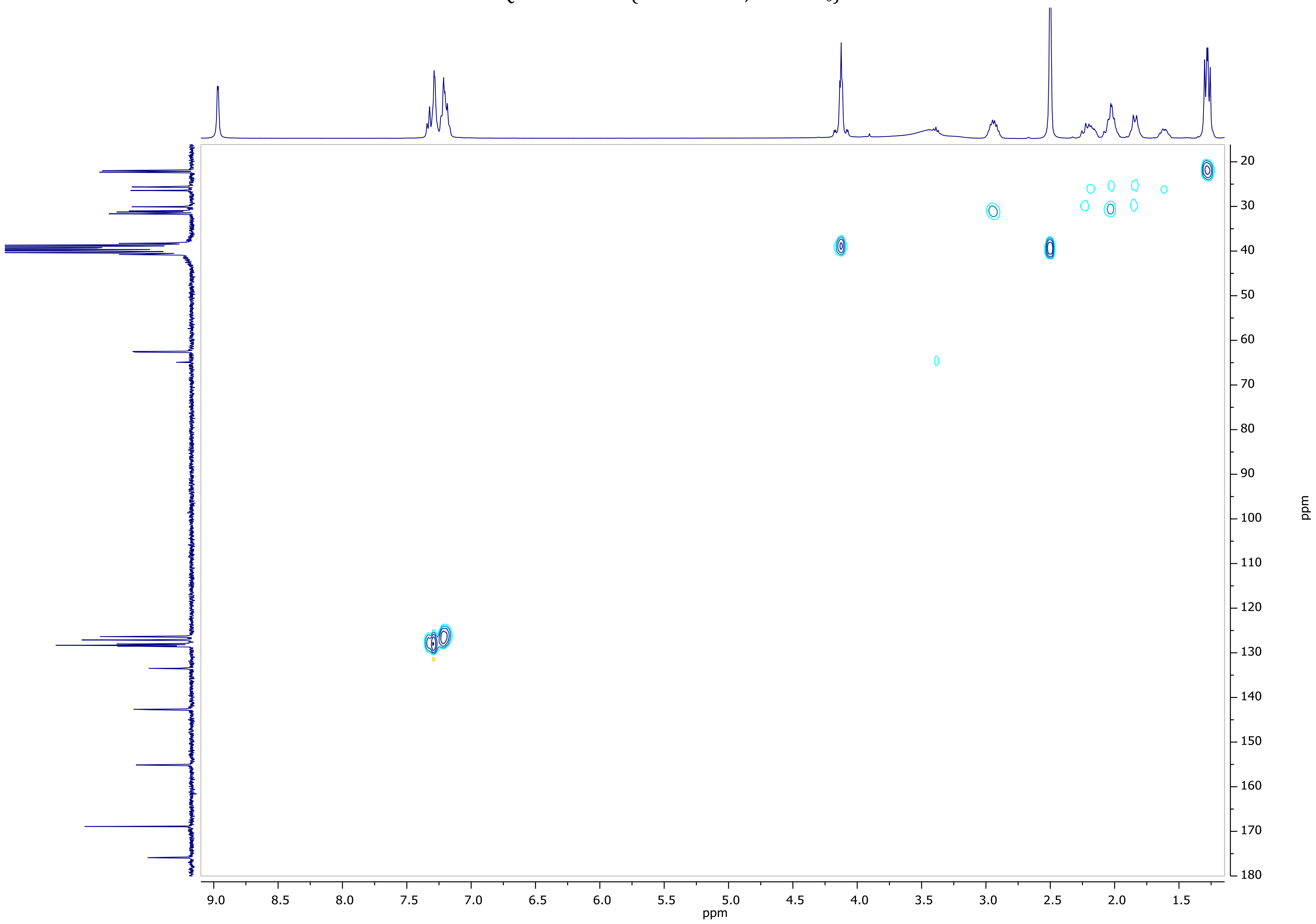

HMBC NMR of **16** (400.13 MHz, DMSO-d_6)

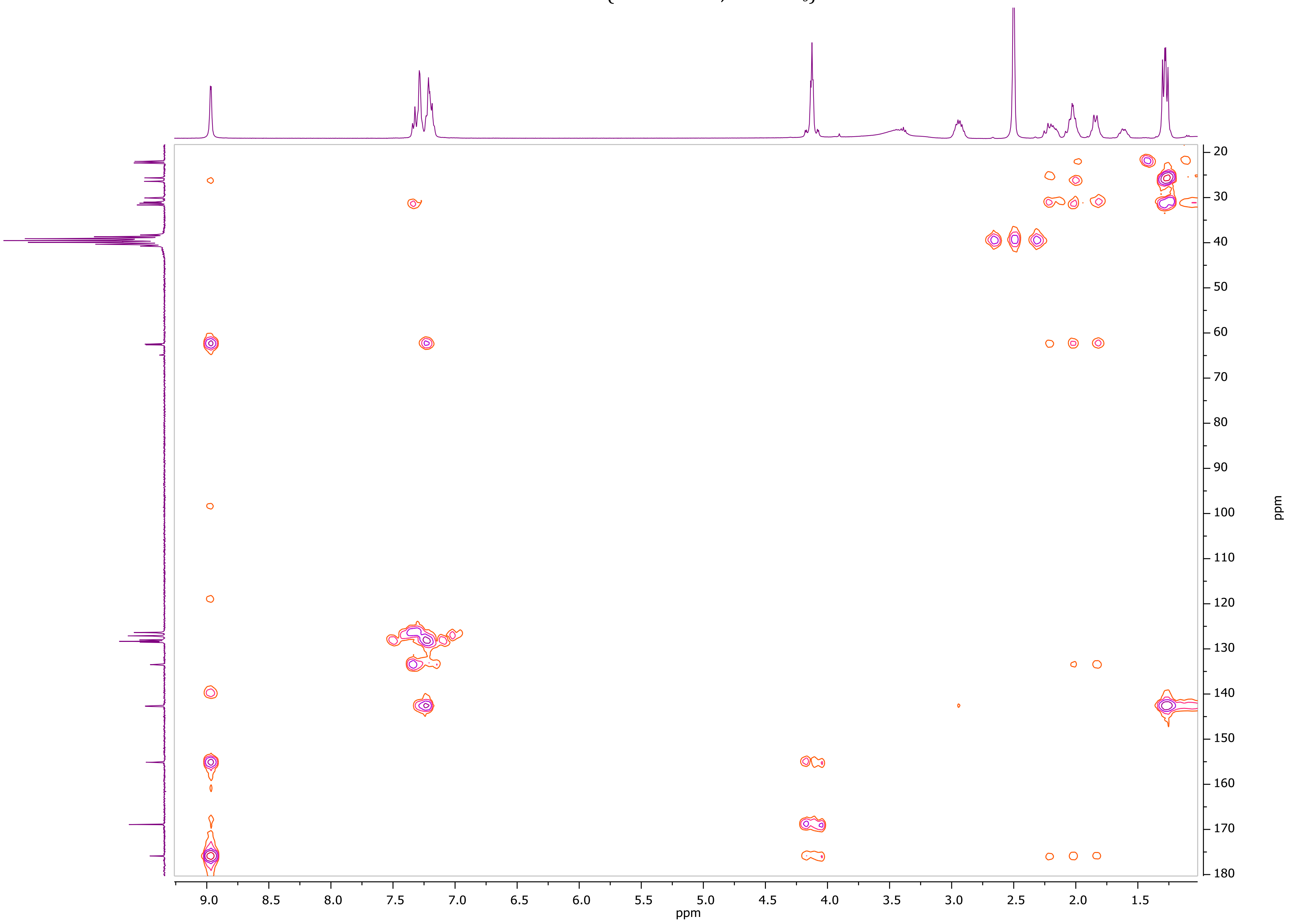

^{1}H NMR of **17** (400.13 MHz, $CDCl_3$)

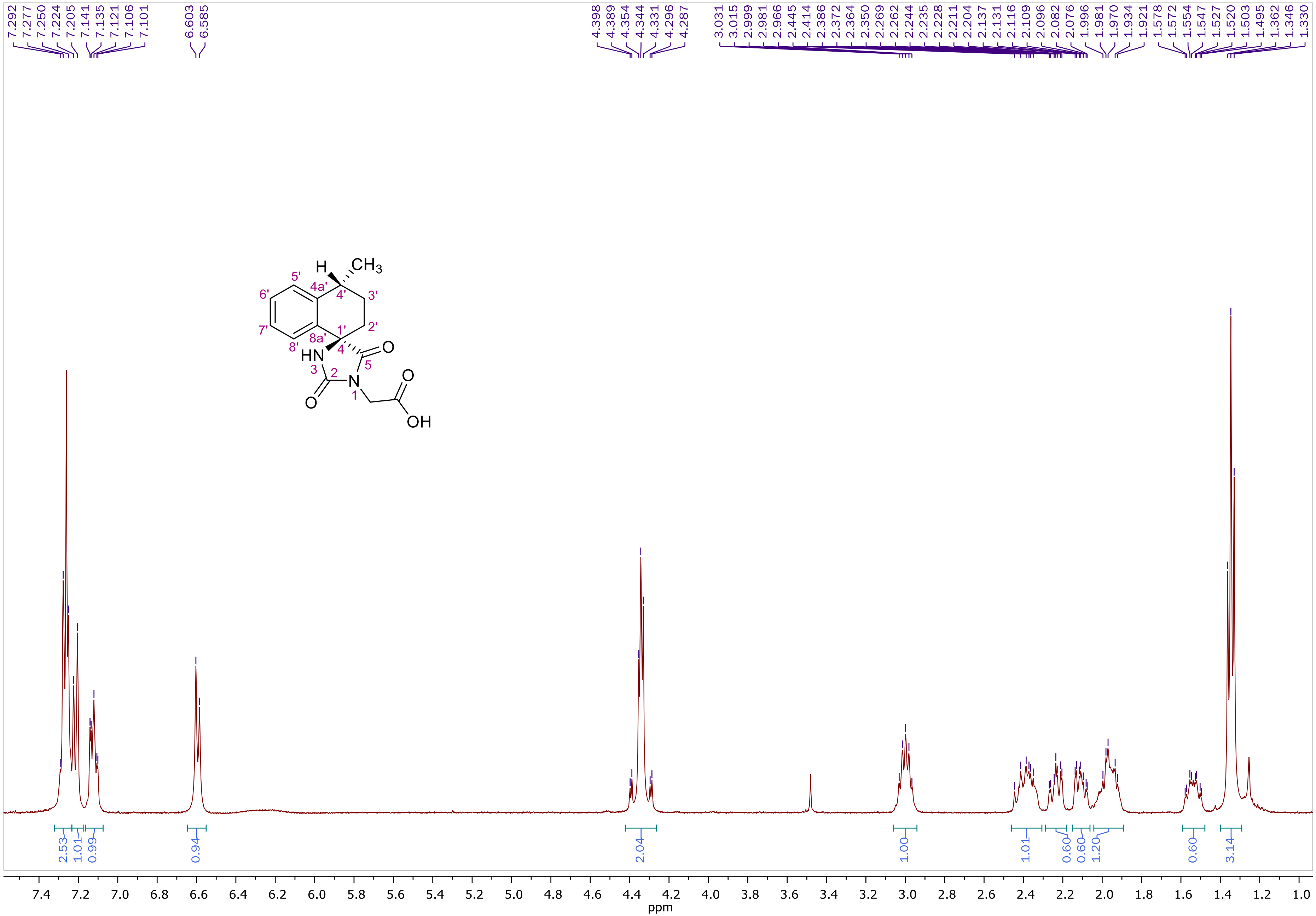

^{13}C NMR of **17** (50.32 MHz, $CDCl_3$)

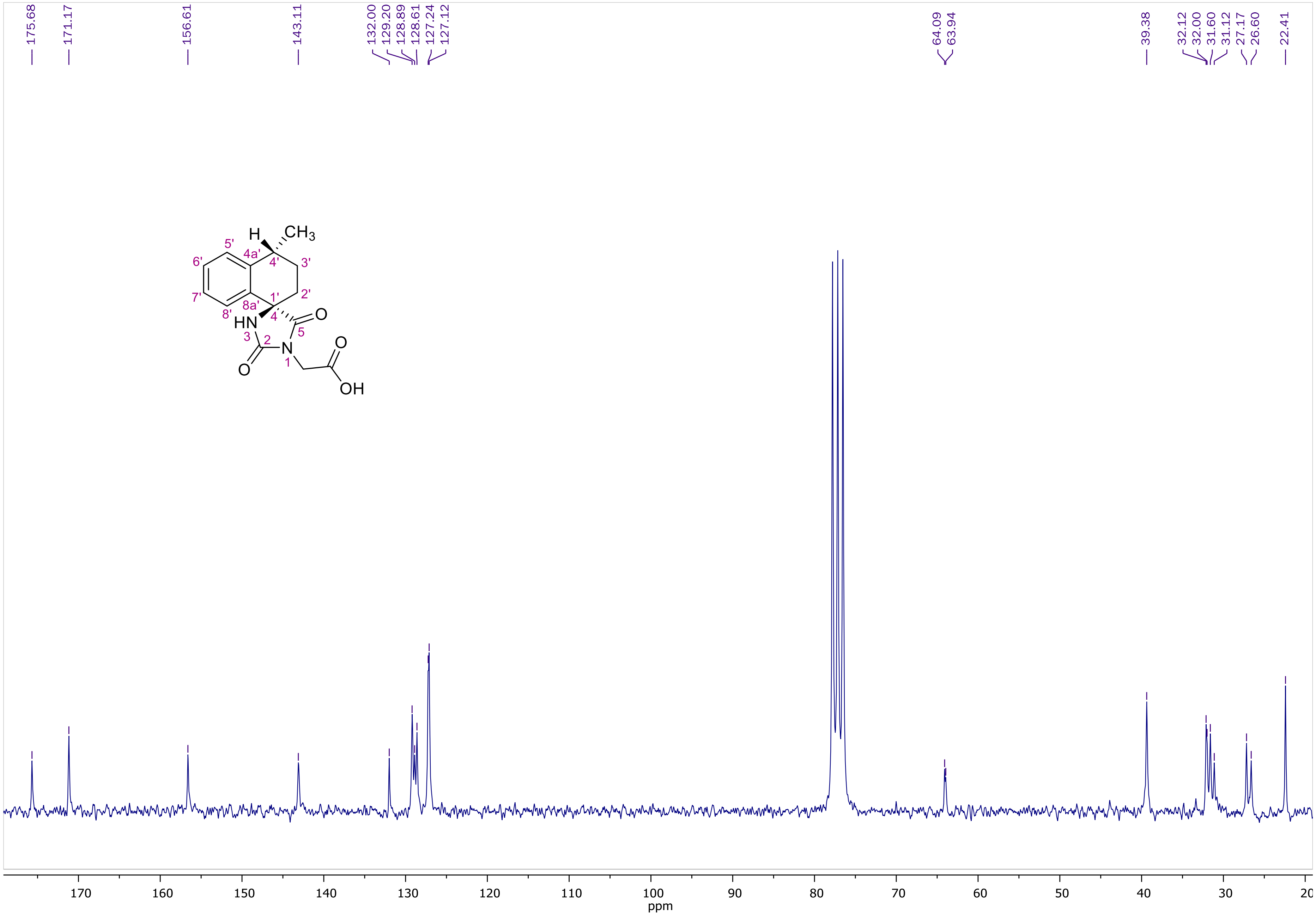

COSY NMR of **17** (400.13 MHz, $CDCl_3$)

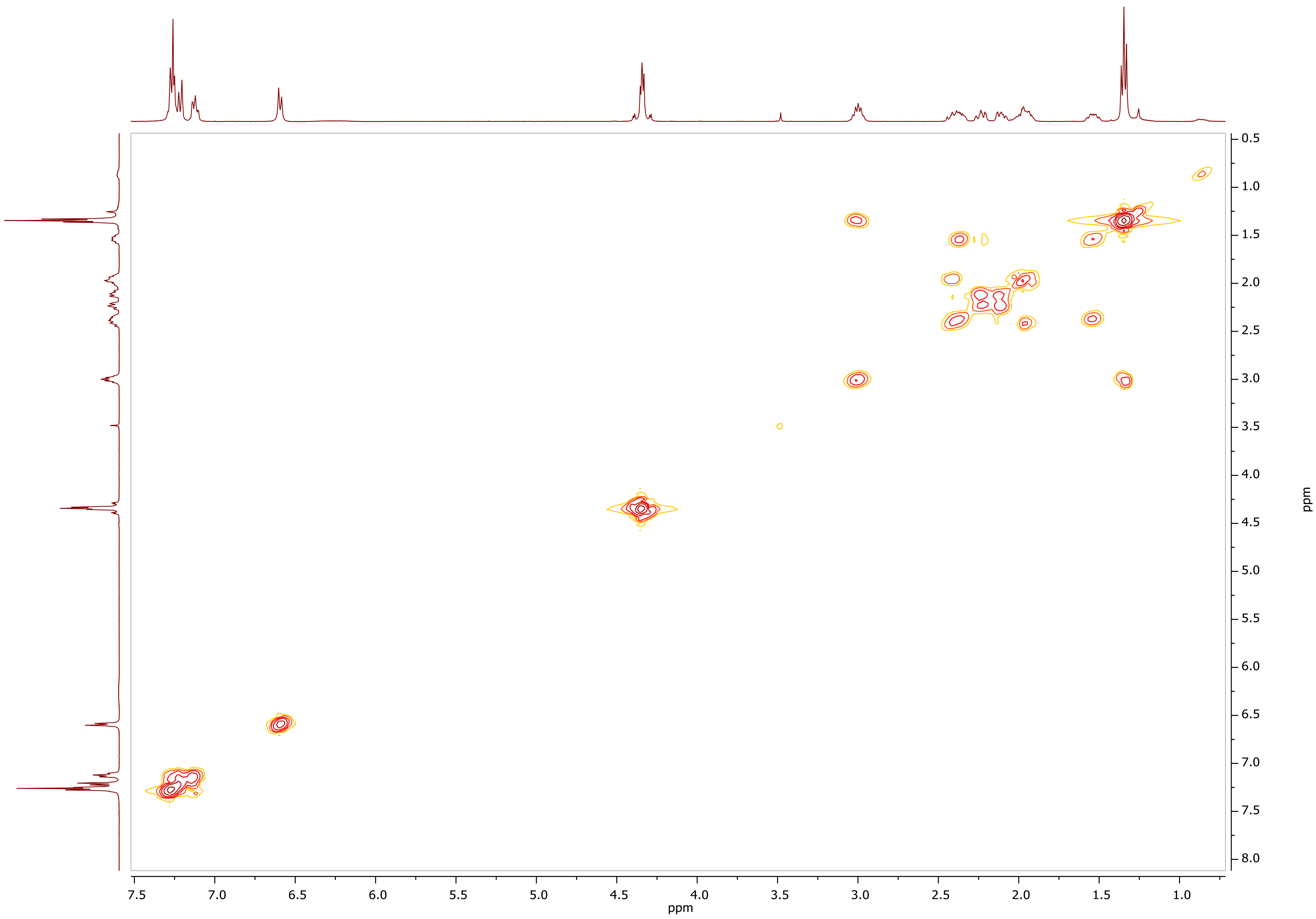

HSQC-DEPT NMR of **17** (400.13 MHz, $CDCl_3$)

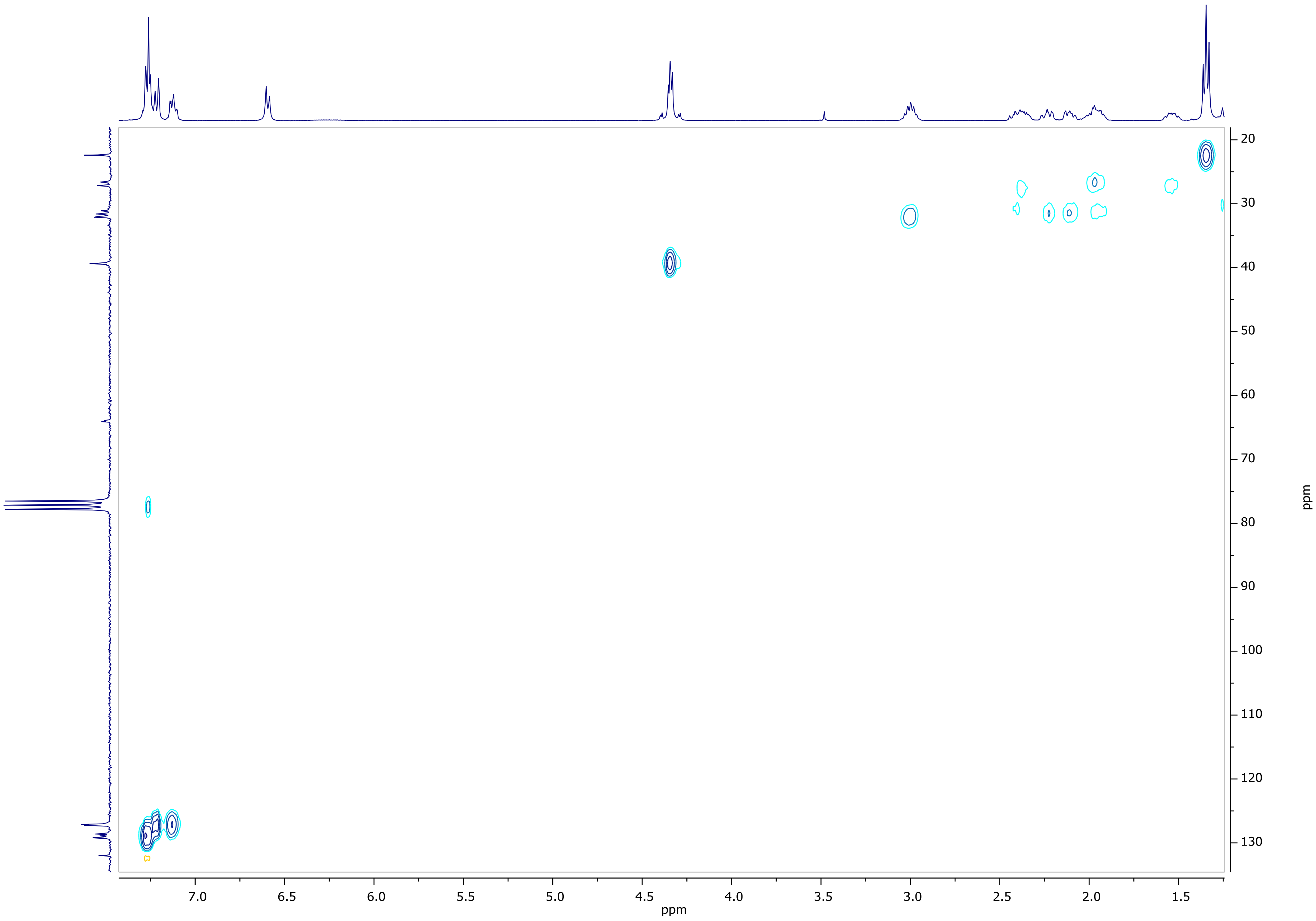

HMBC NMR of **17** (400.13 MHz, $CDCl_3$)

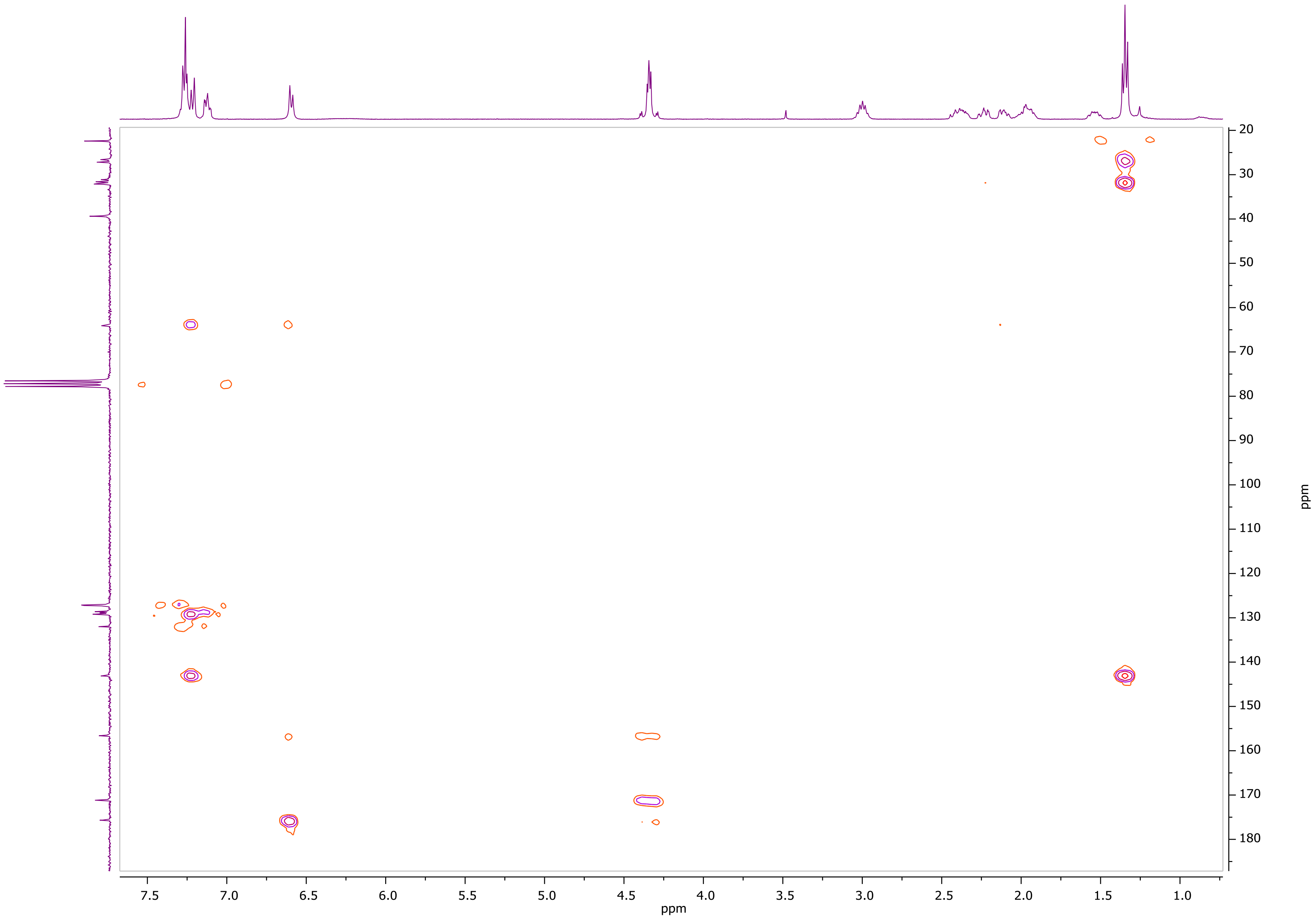

^{1}H NMR of **18** (600.11 MHz, DMSO-d_6)

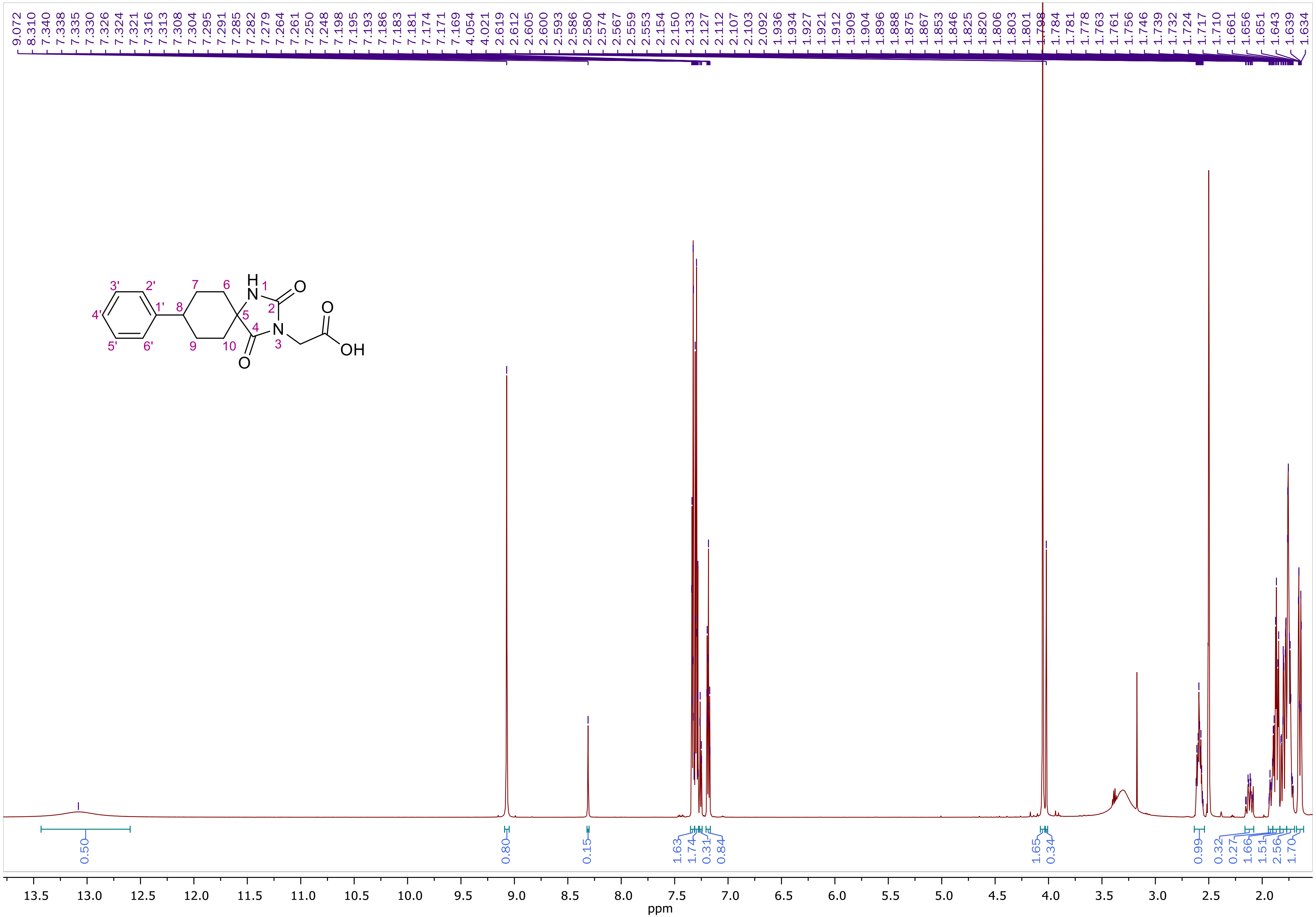

^{13}C NMR of **18** (100.61 MHz, DMSO-d_6)

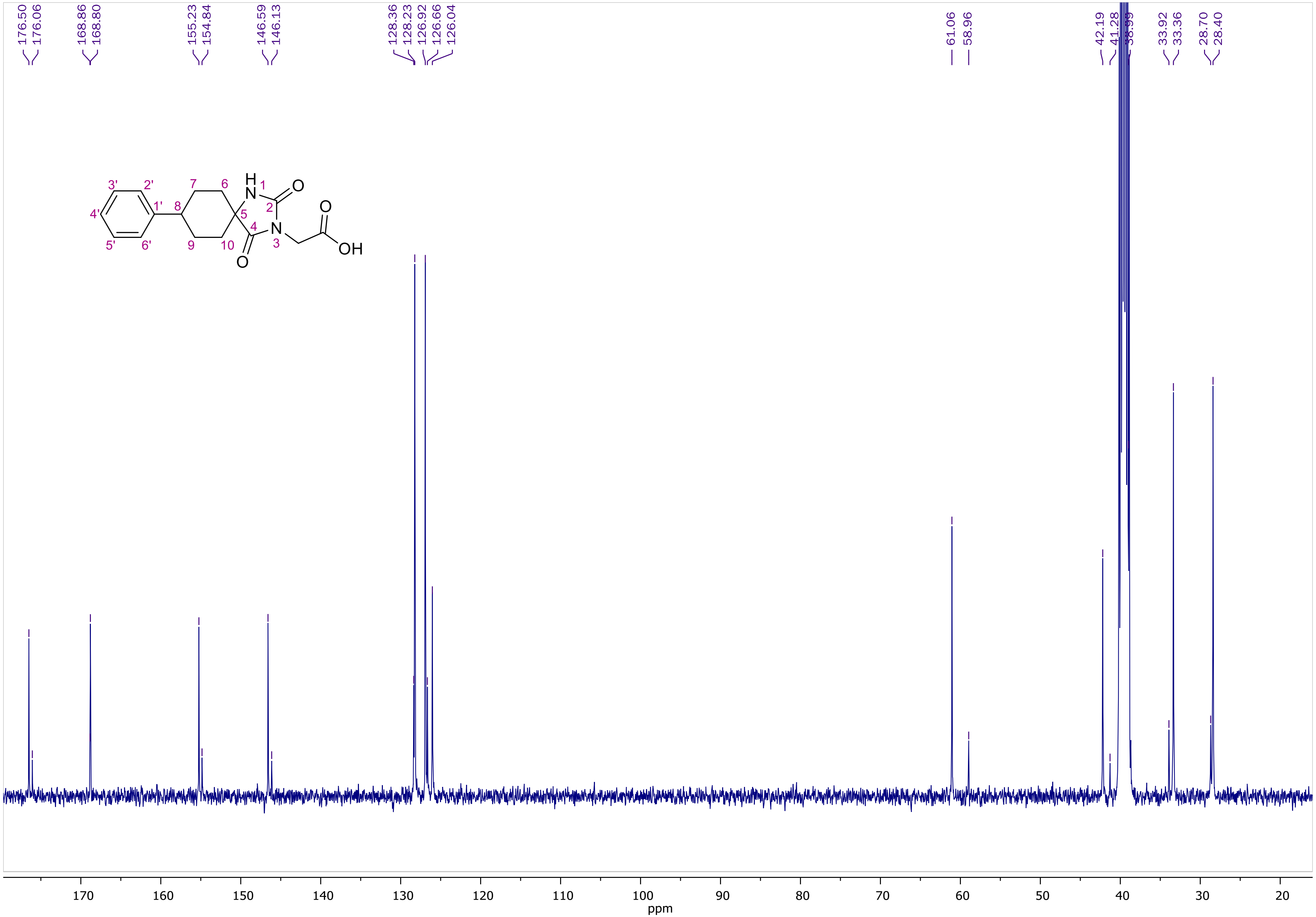

COSY NMR of **18** (400.13 MHz, DMSO-d_6)

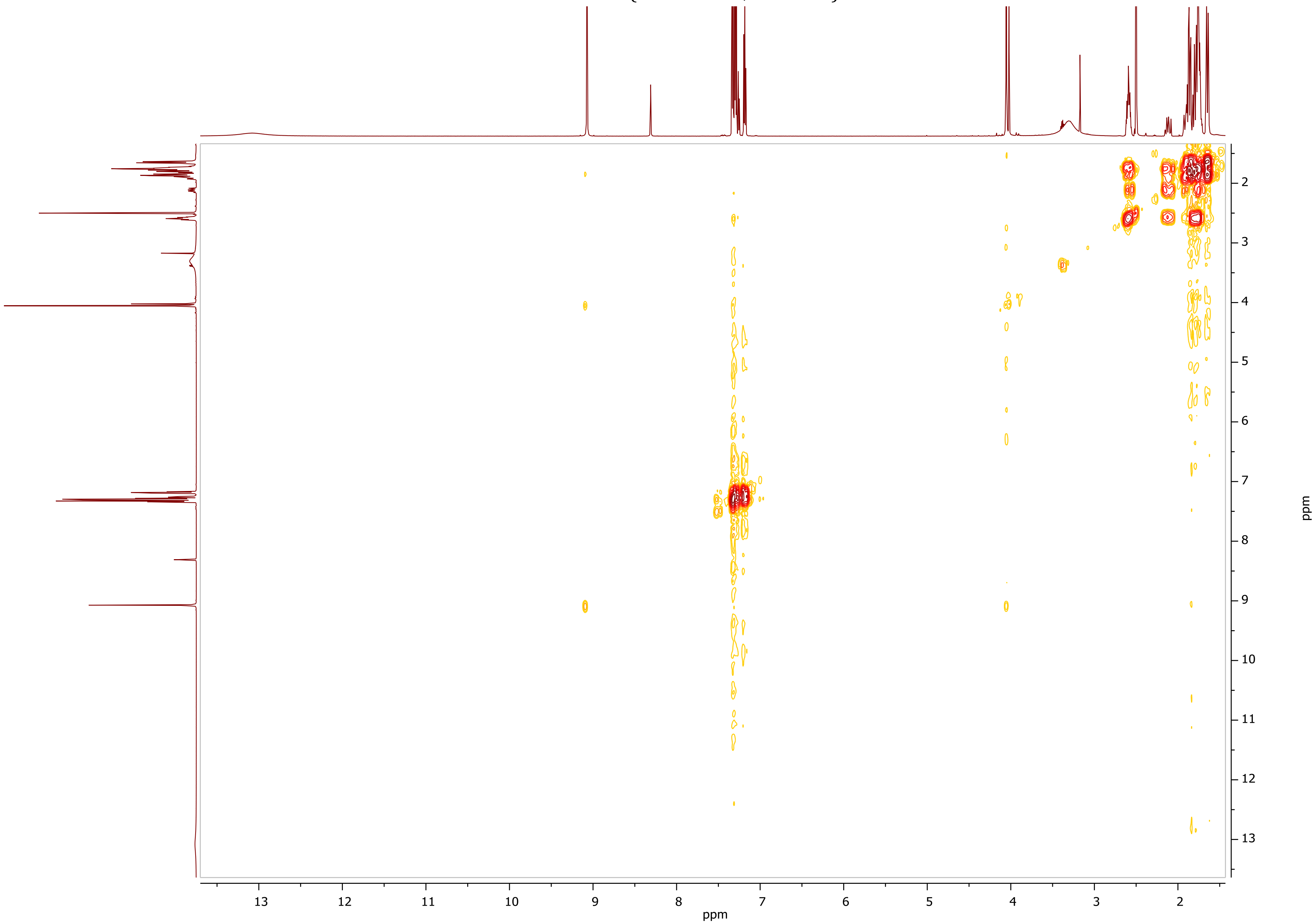

HSQC-DEPT NMR of **18** (400.13 MHz, DMSO-d_6)

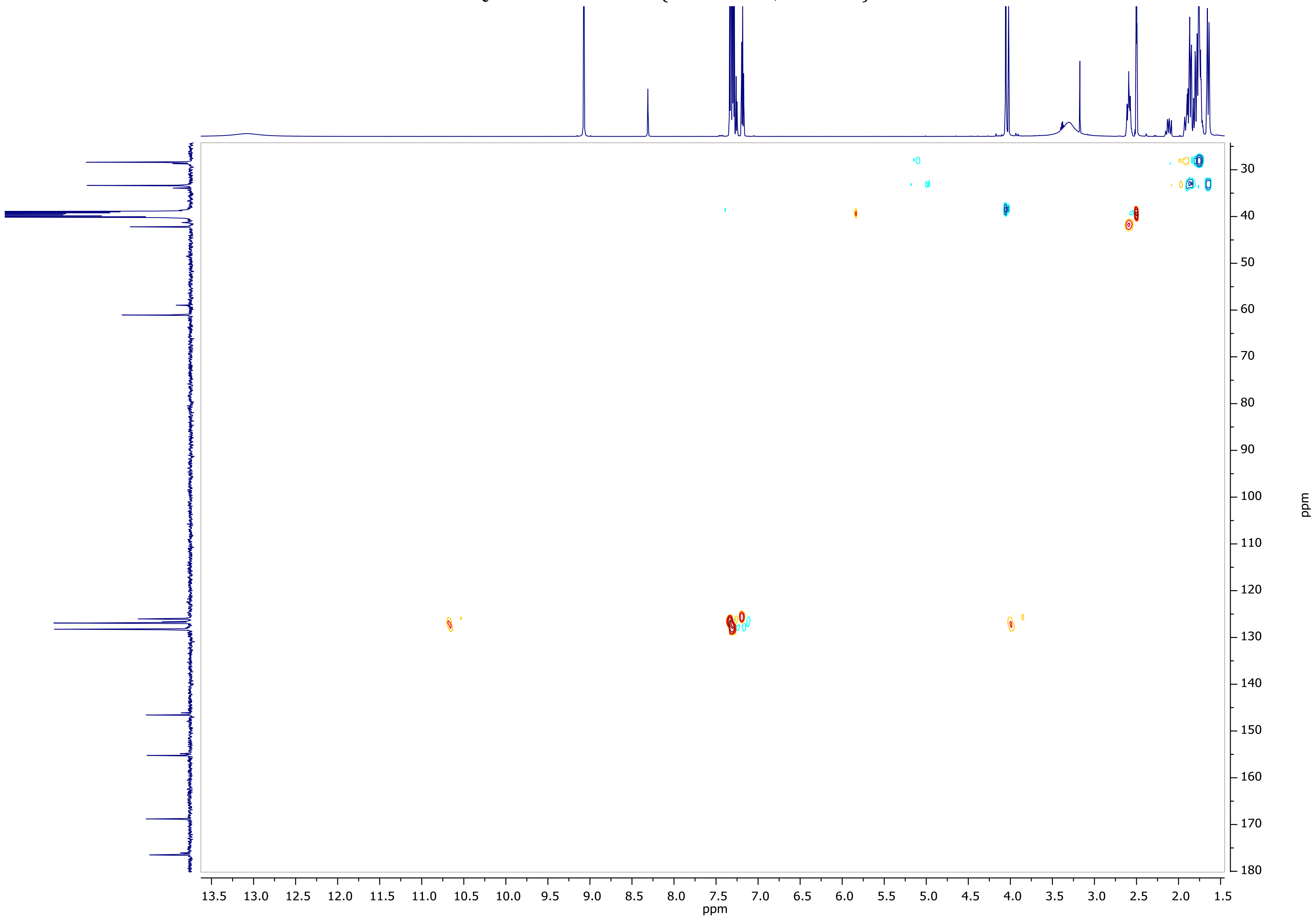

HMBC NMR of **18** (400.13 MHz, DMSO-d_6)

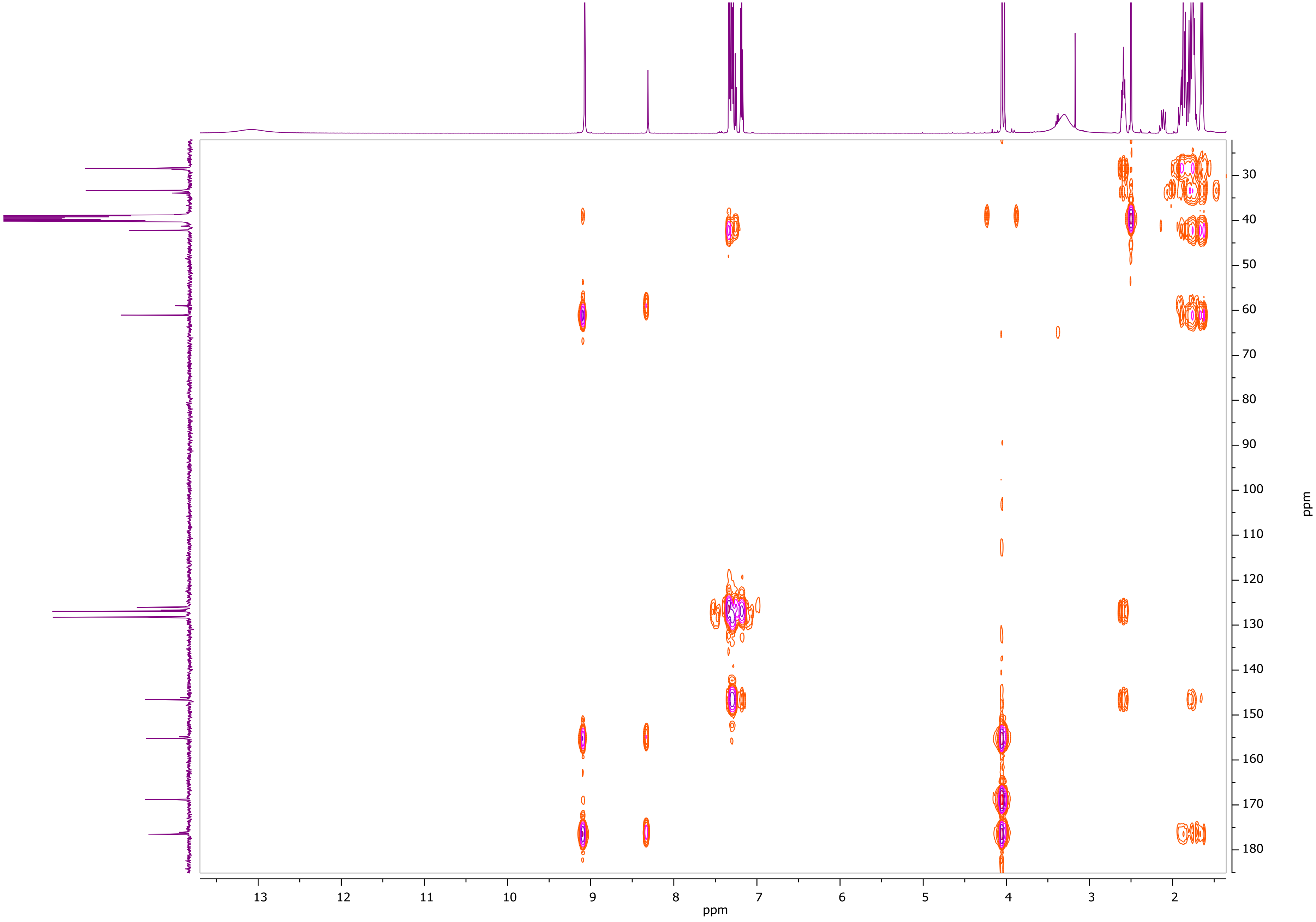

NOESY NMR of **18** (400.13 MHz, DMSO-d_6)

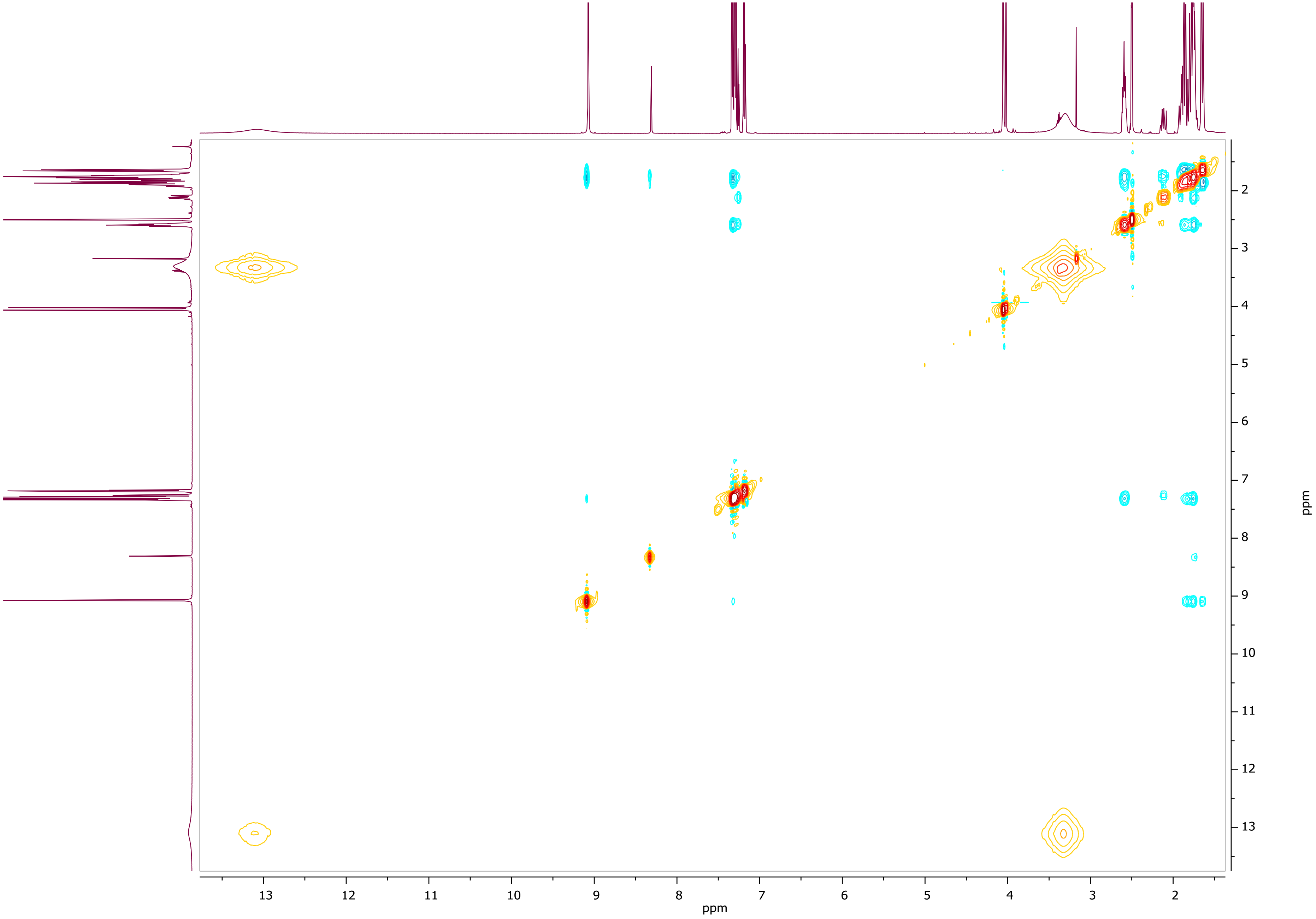

^{1}H NMR of **19** (600.11 MHz, DMSO-d_6)

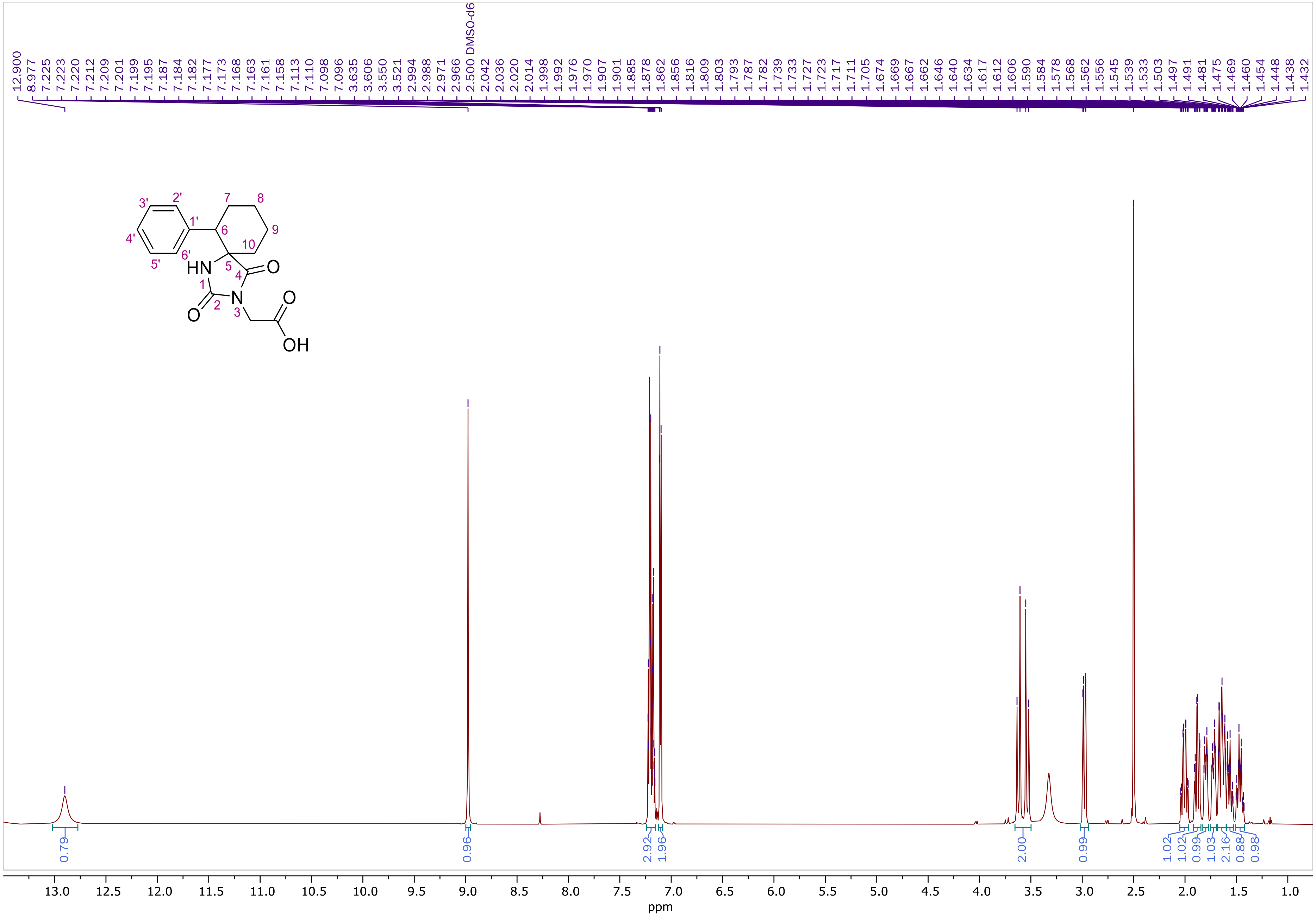

^{13}C NMR of **19** (150.9 MHz, DMSO-d_6)

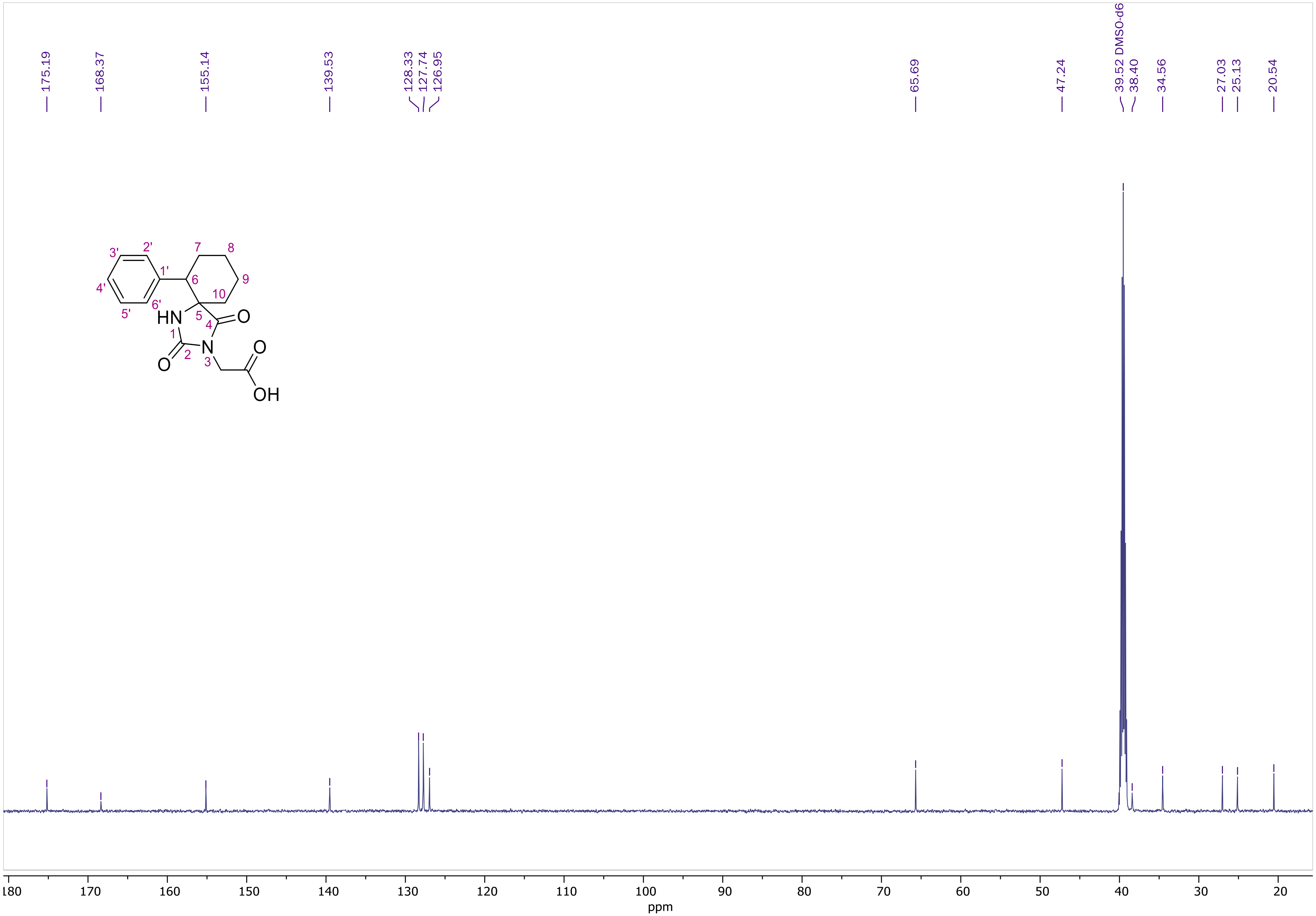

COSY NMR of **19** (400.13 MHz, DMSO-d_6)

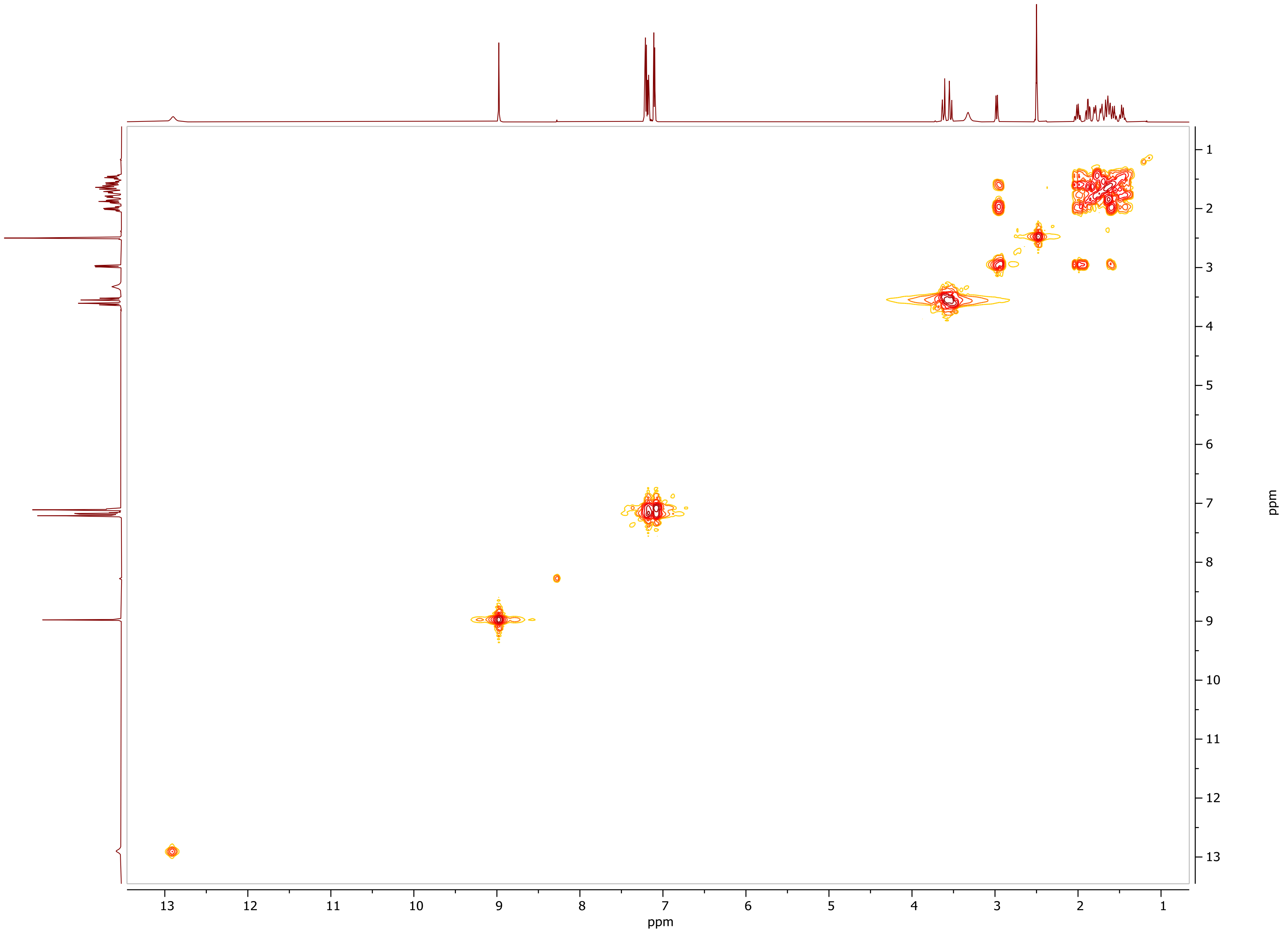

HSQC NMR of **19** (400.13 MHz, DMSO-d_6)

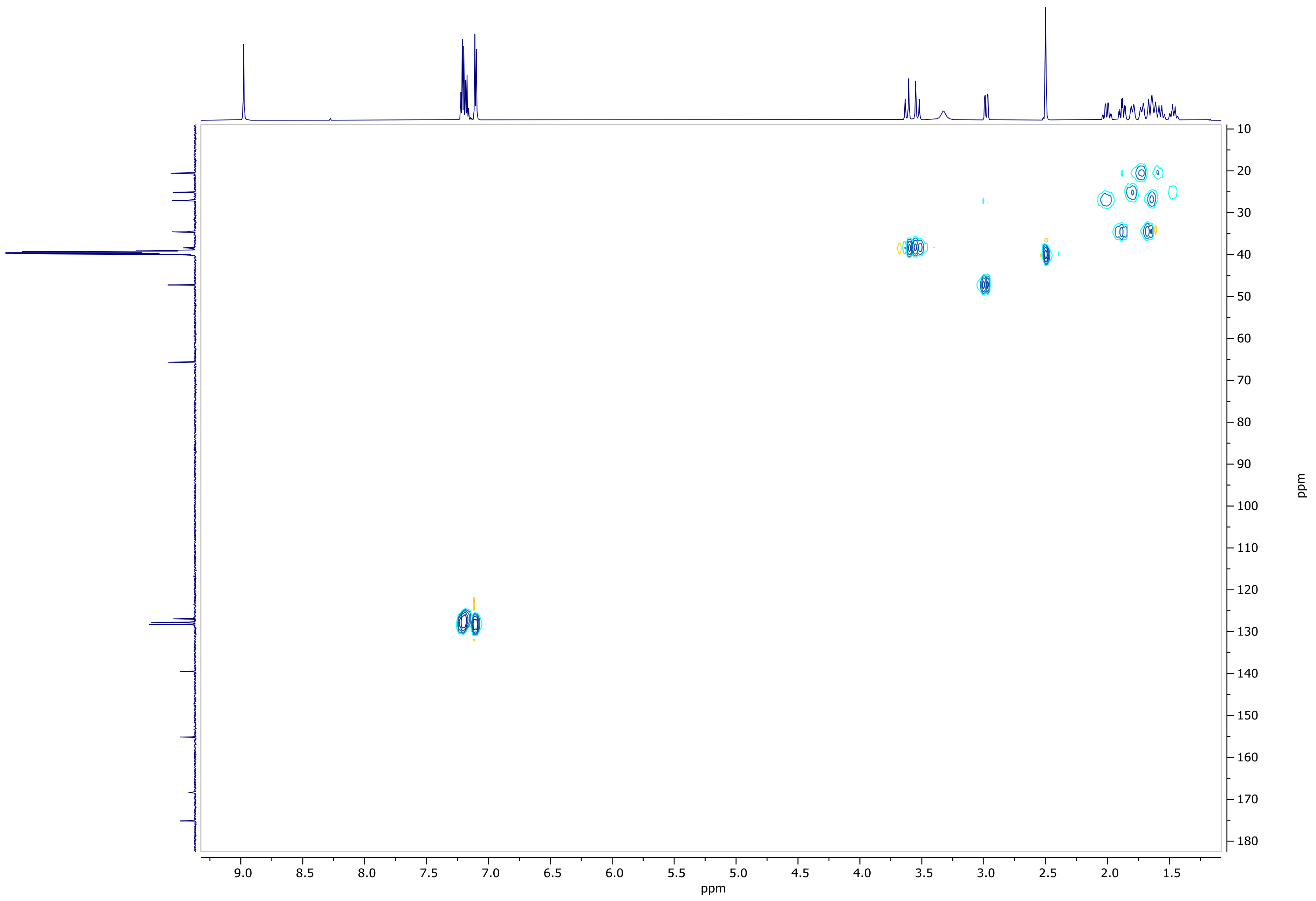

HMBC NMR of **19** (400.13 MHz, DMSO-d_6)

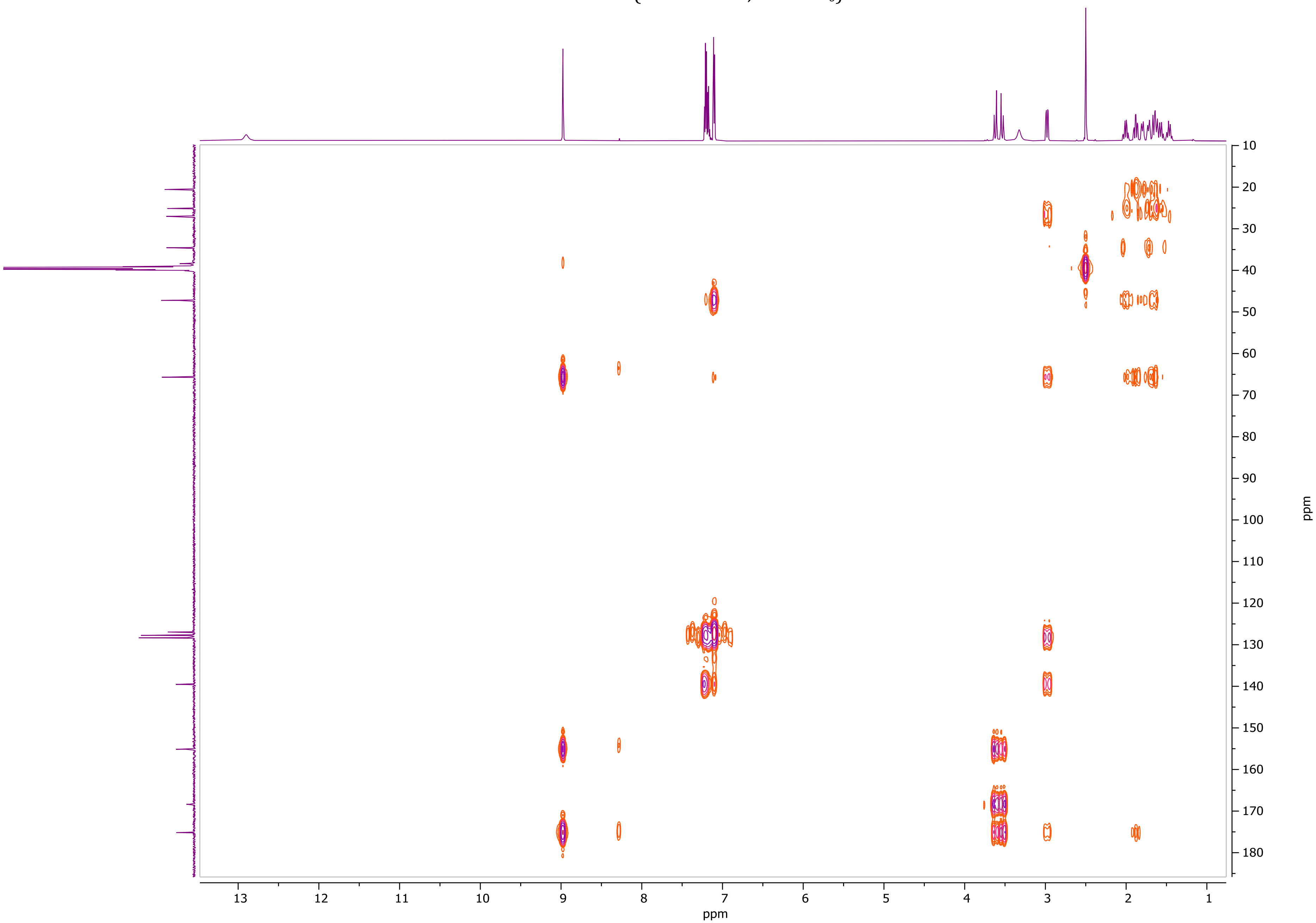

^{1}H NMR of **20** (400.13 MHz, DMSO-d_6)

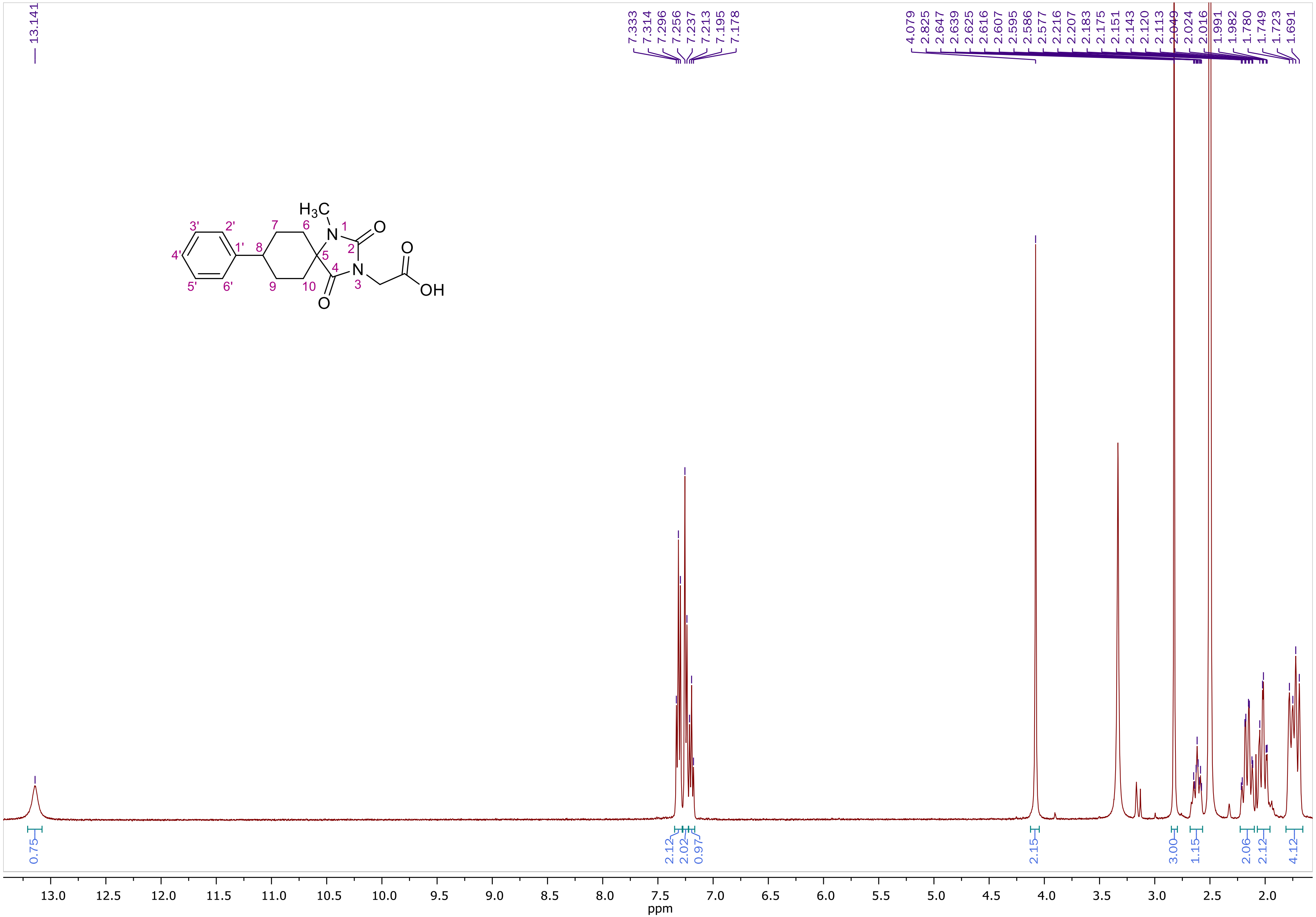

^{13}C NMR of **20** (50.32 MHz, DMSO-d_6)

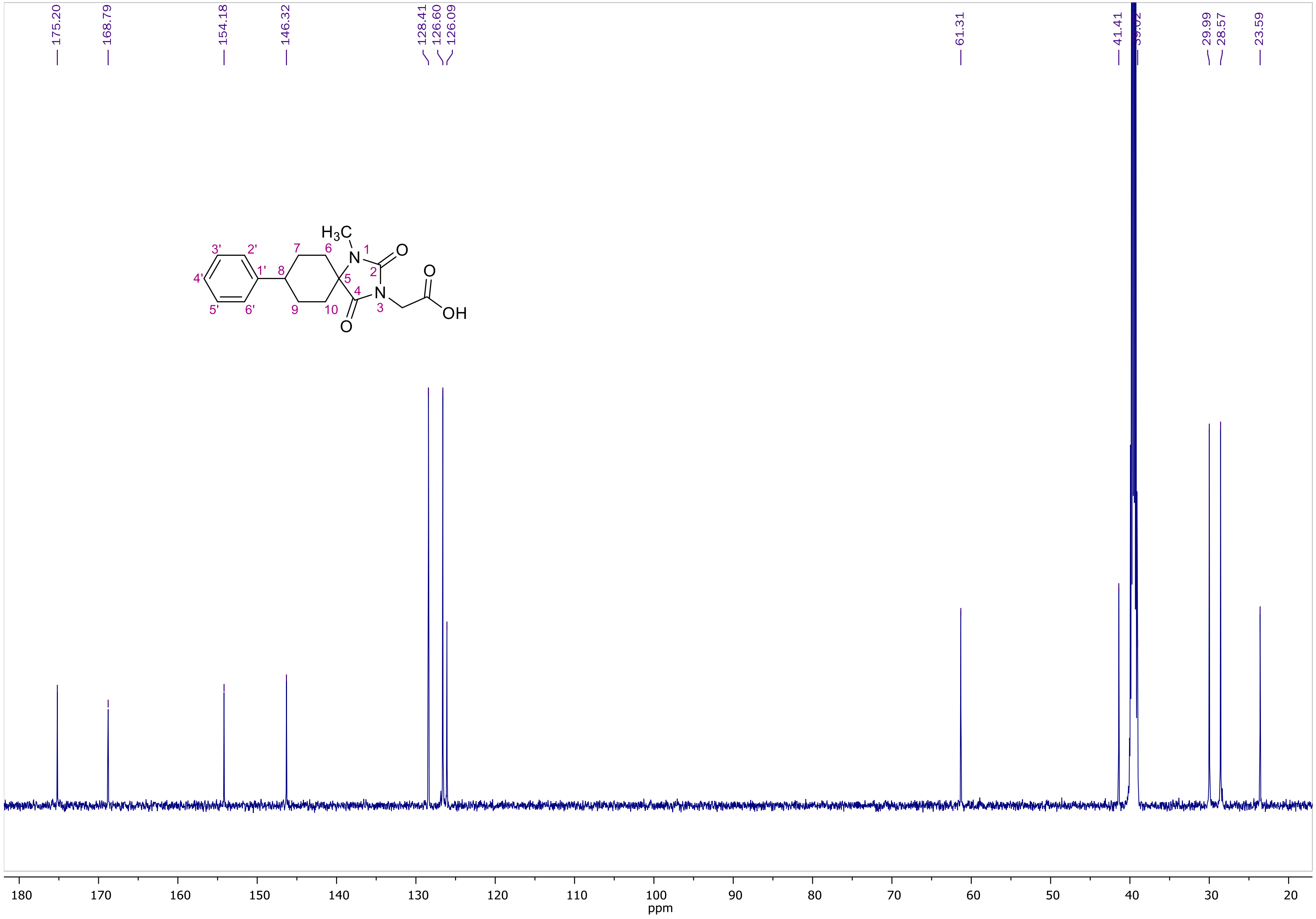

COSY NMR of **20** (400.13 MHz, DMSO-d_6)

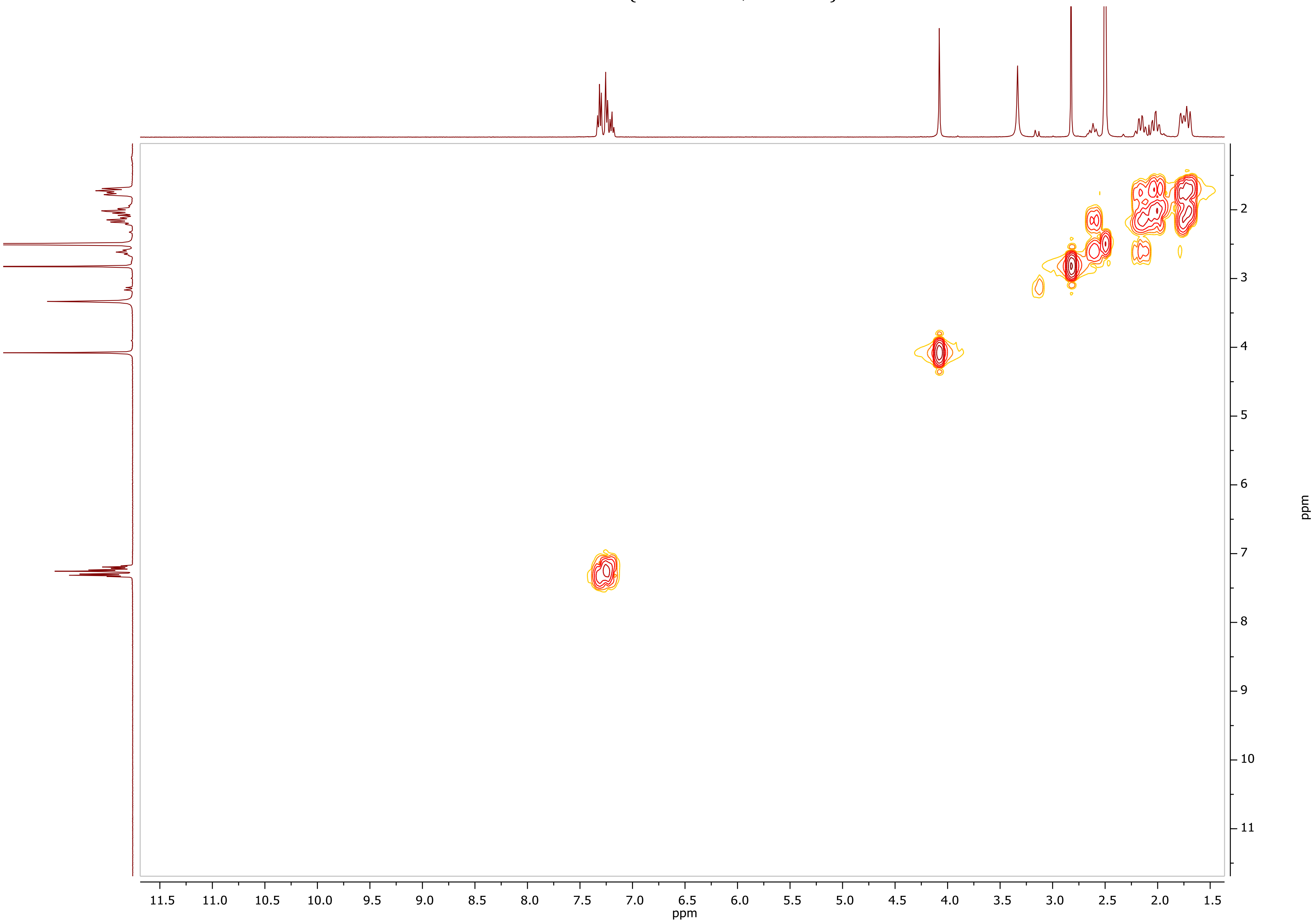

HSQC NMR of **20** (400.13 MHz, DMSO-d_6)

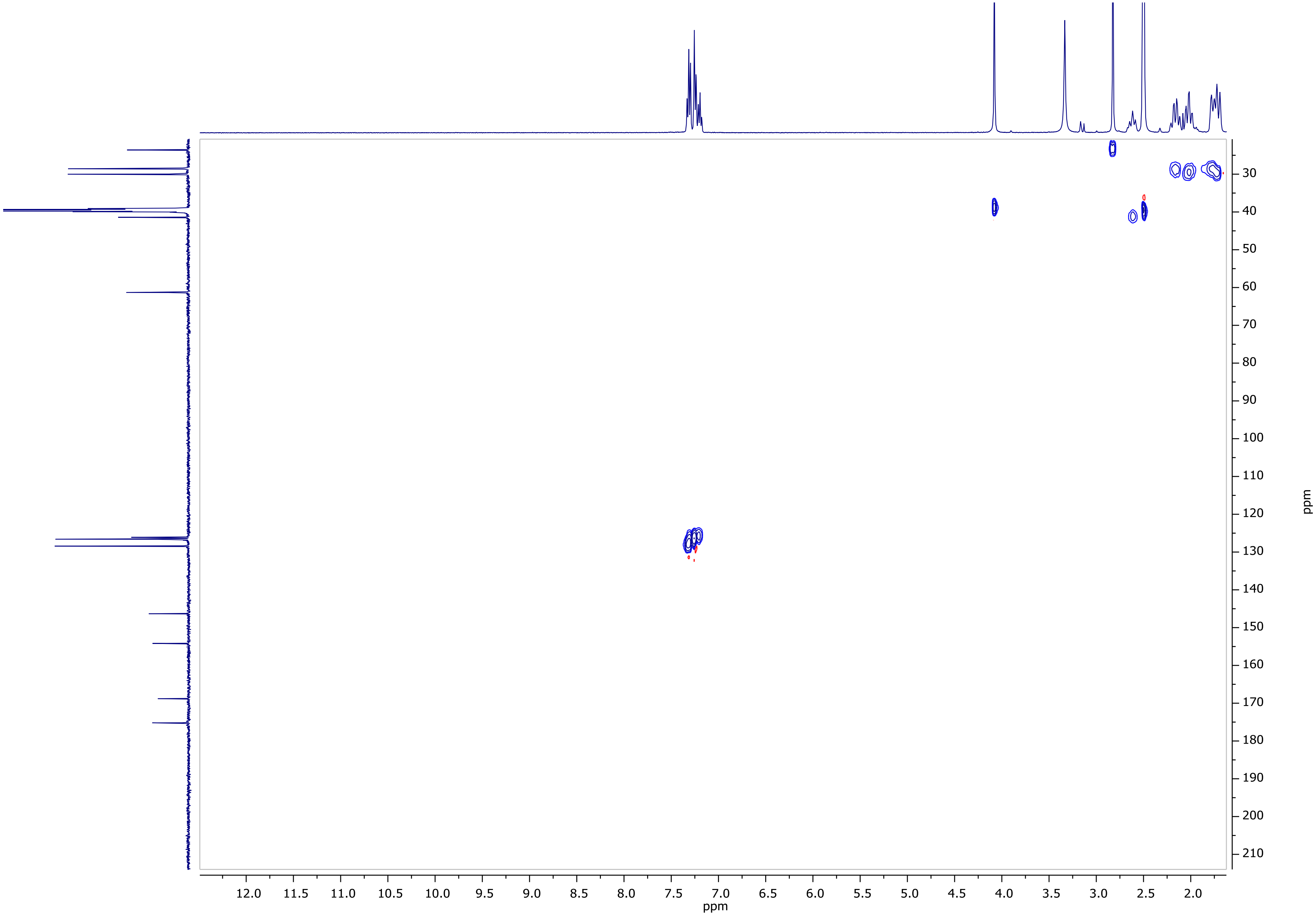

HMBC NMR of **20** (400.13 MHz, DMSO-d_6)

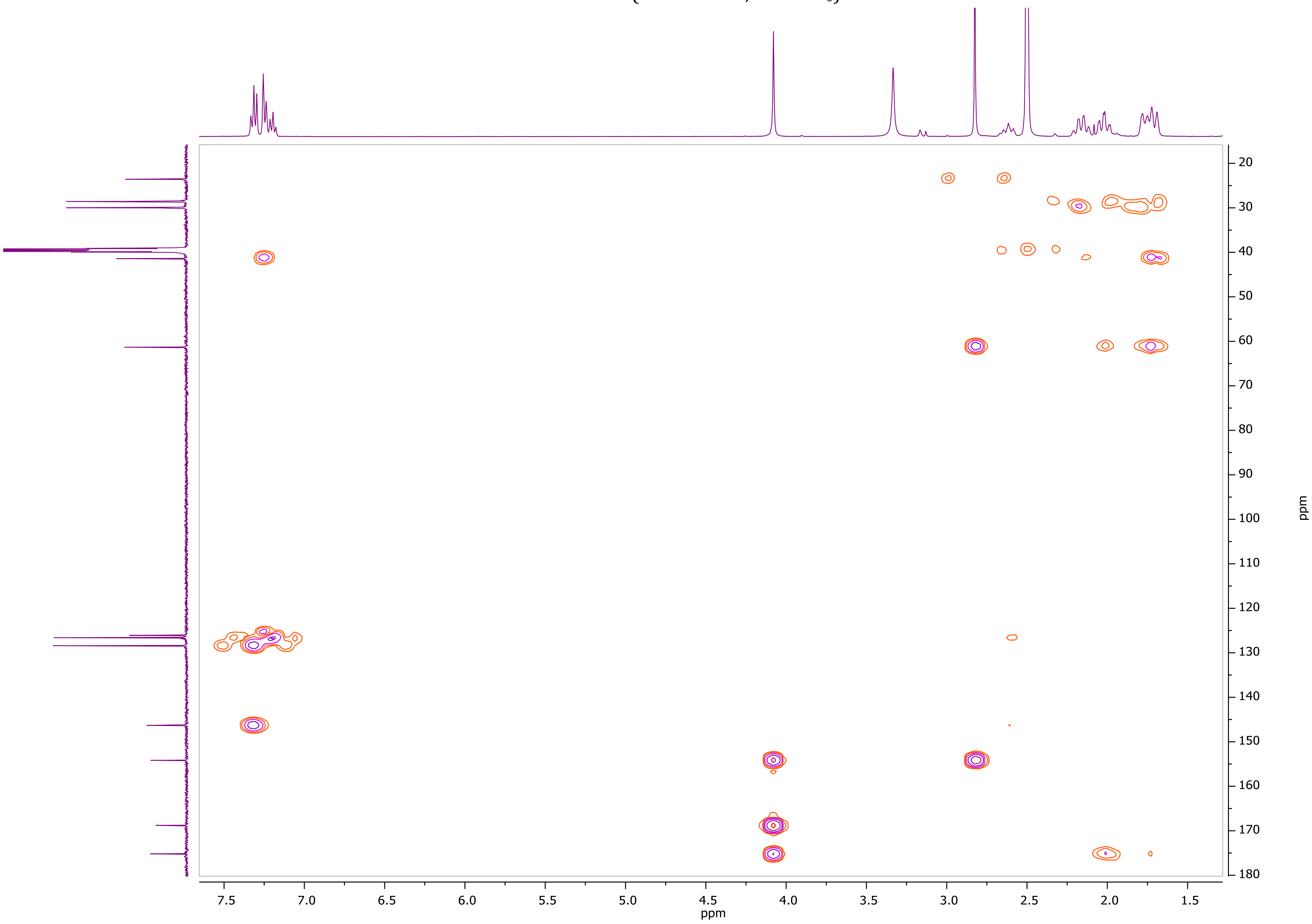

^{1}H NMR of **21** (600.11 MHz, DMSO-d_6)

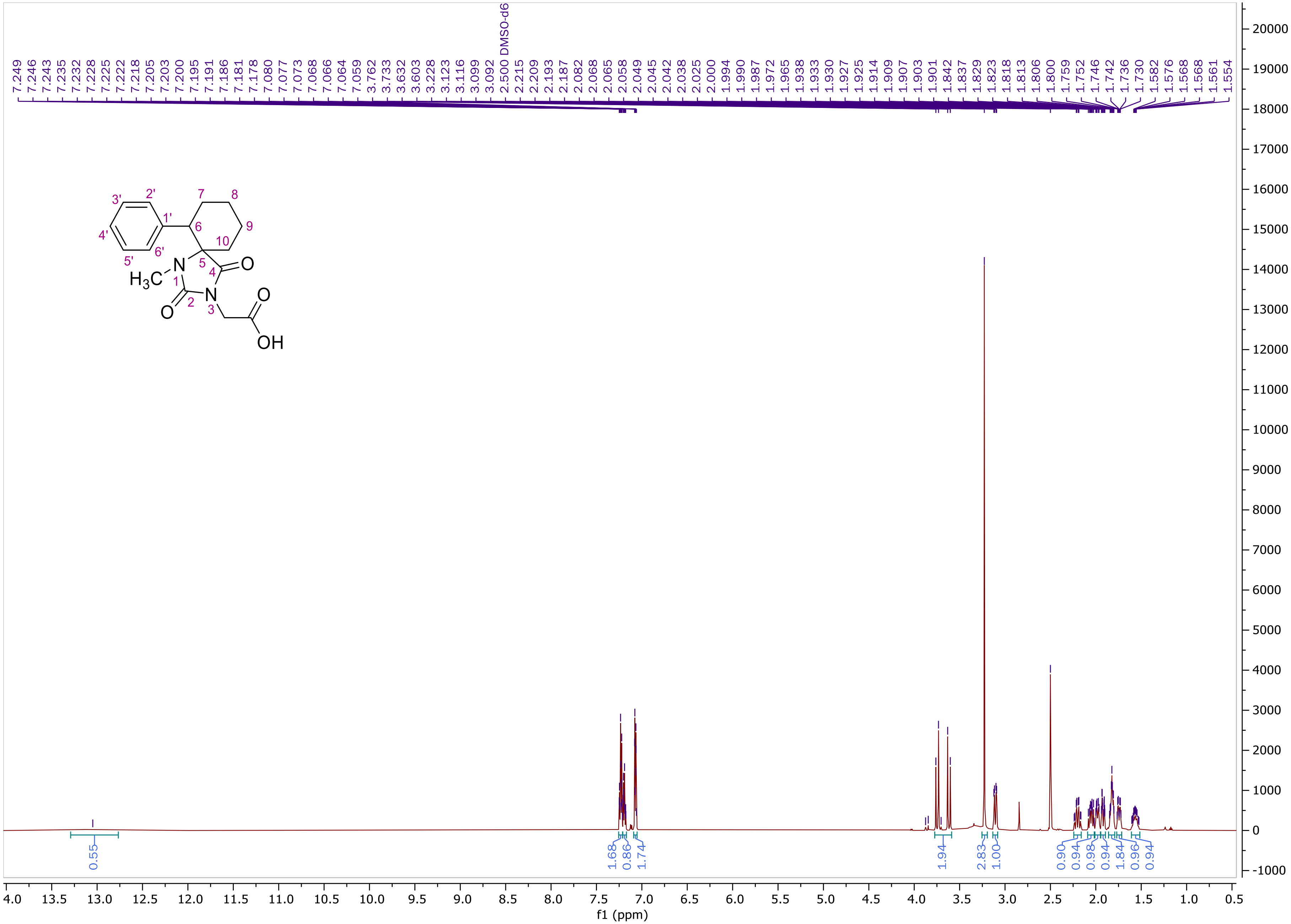

^{13}C NMR of **21** (150.9 MHz, DMSO-d_6)

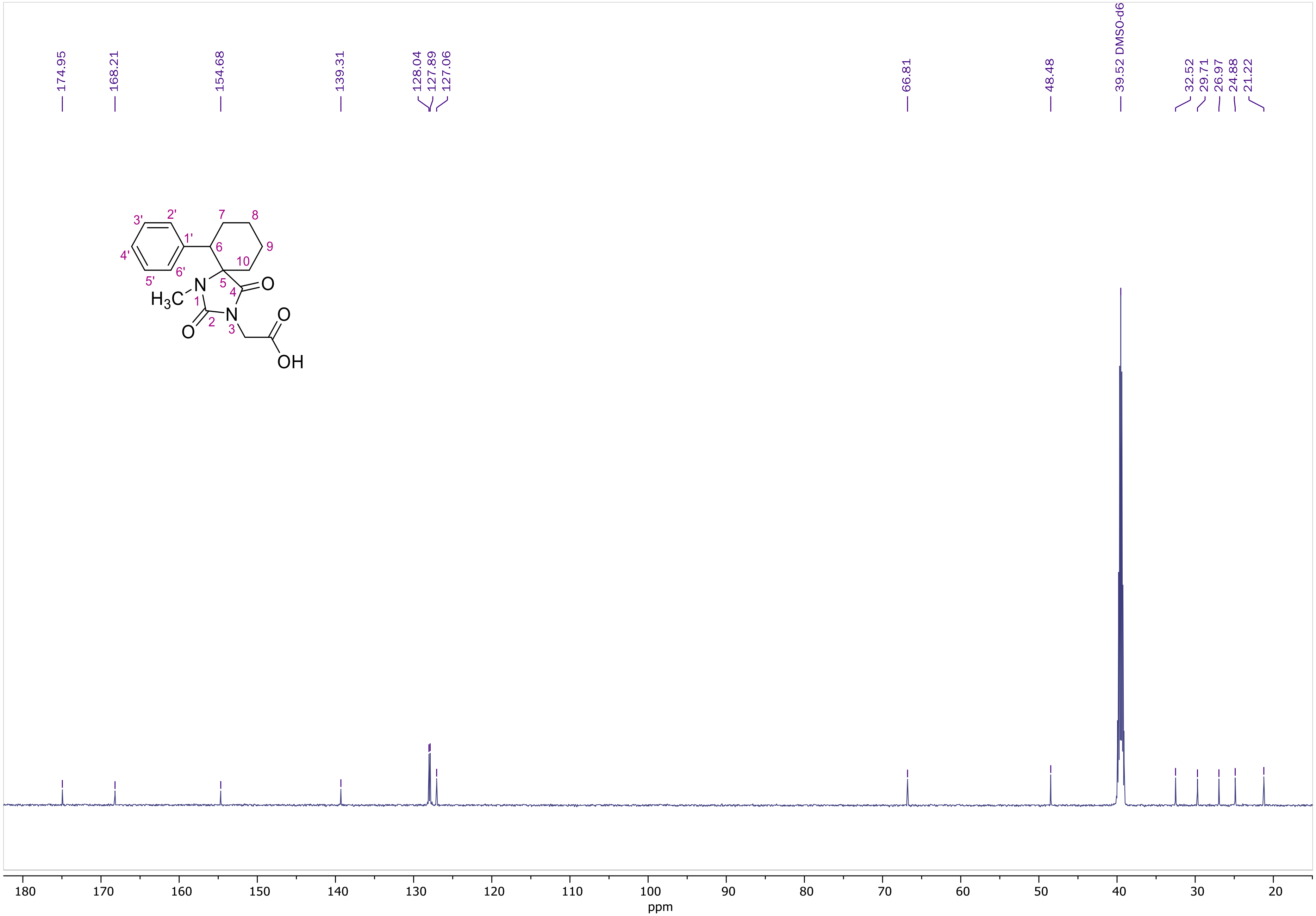

COSY NMR of **21** (400.13 MHz, DMSO-d_6)

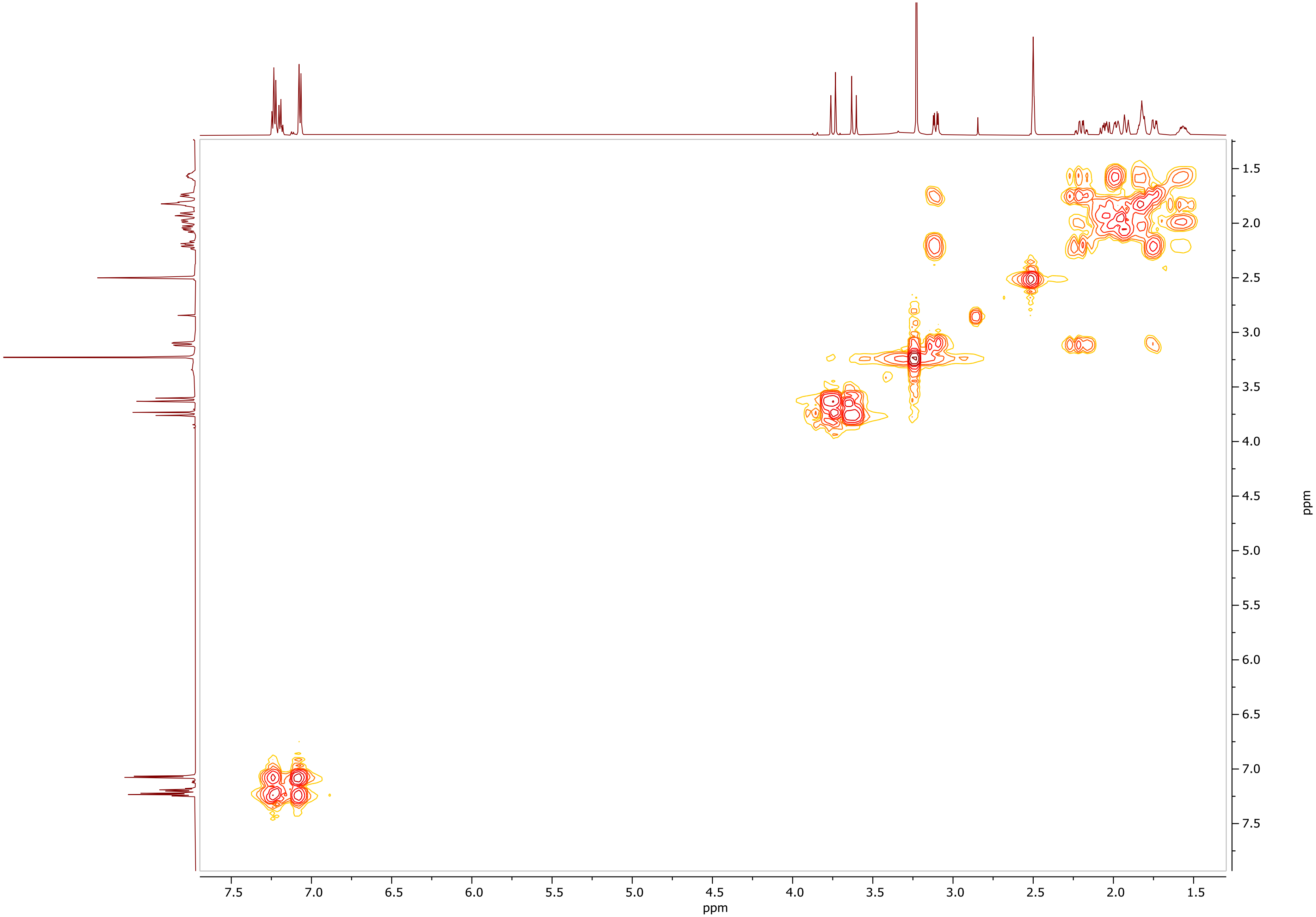

HSQC NMR of **21** (400.13 MHz, DMSO-d_6)

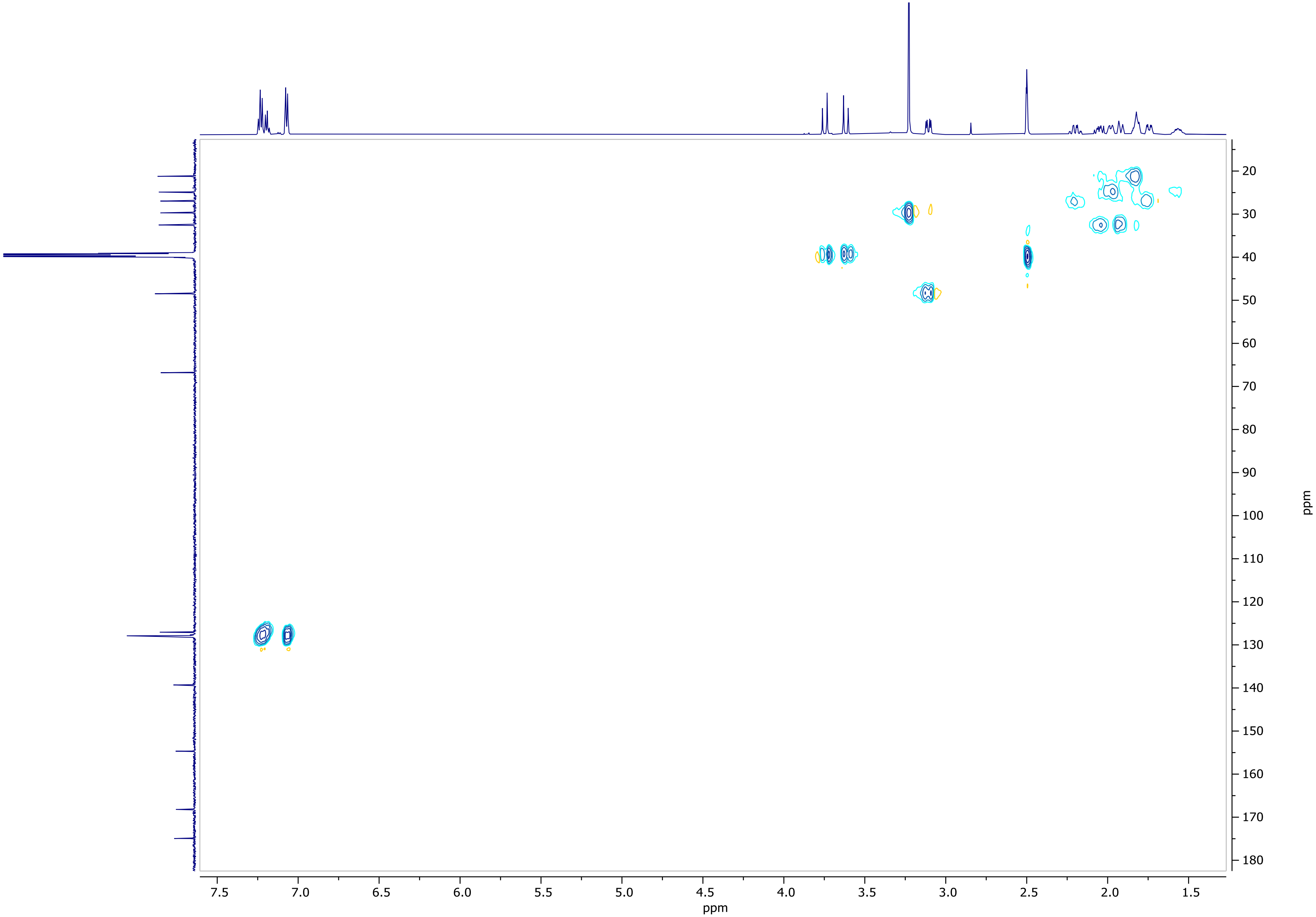

HMBC NMR of **21** (400.13 MHz, DMSO-d_6)

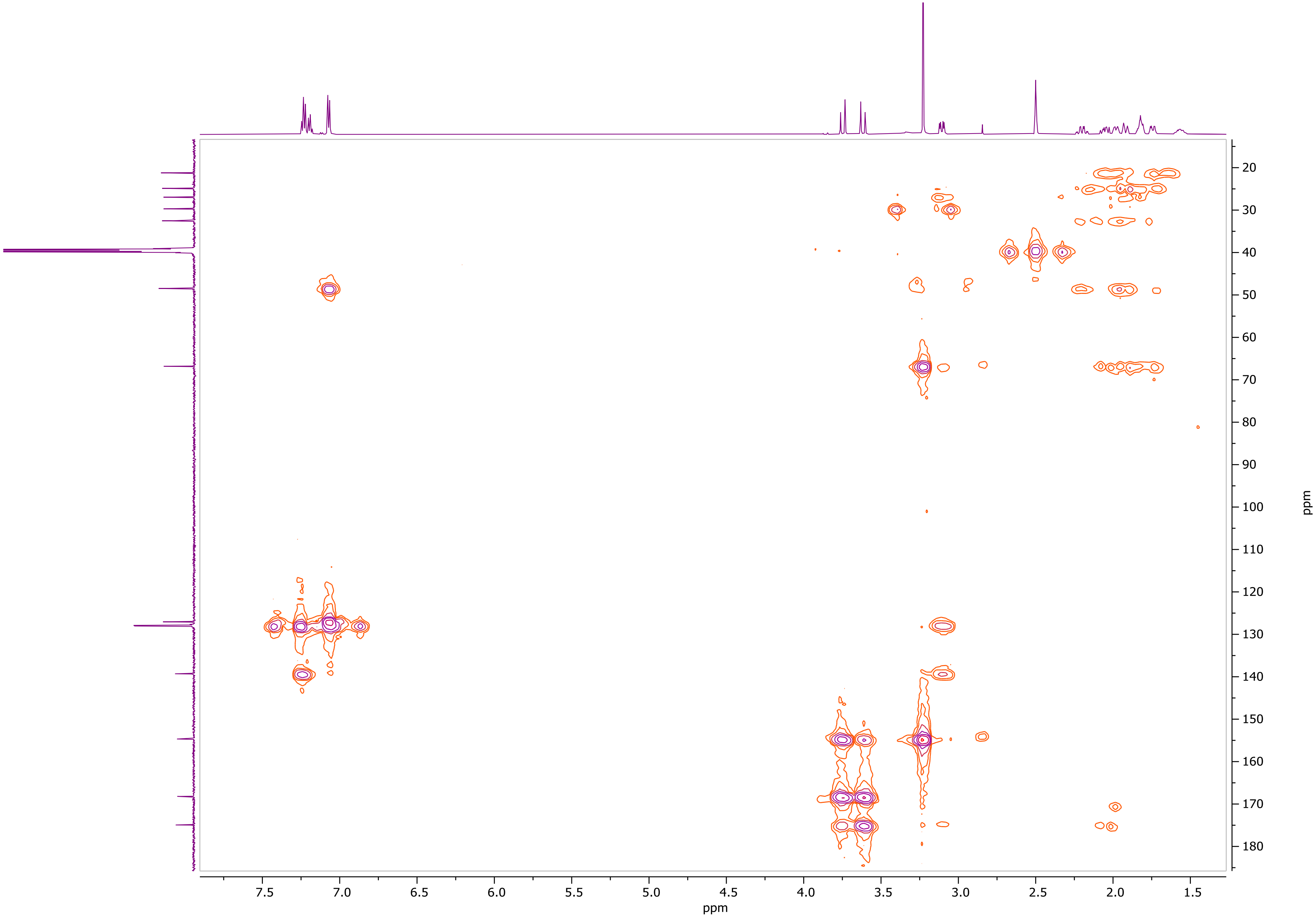

^{1}H NMR of **22** (600.11 MHz, DMSO-d_6)

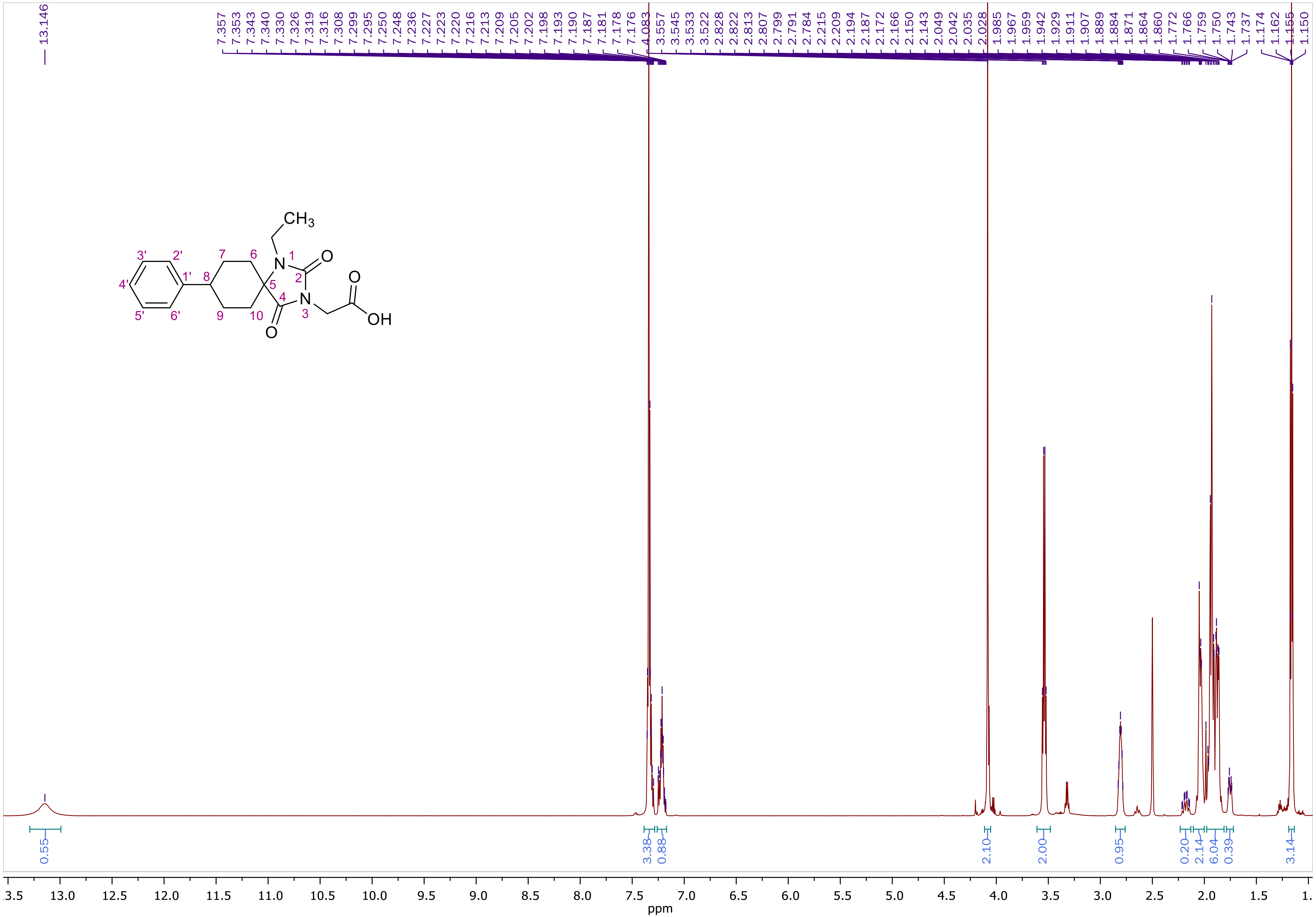

^{13}C NMR of **22** (150.9 MHz, DMSO-d_6)

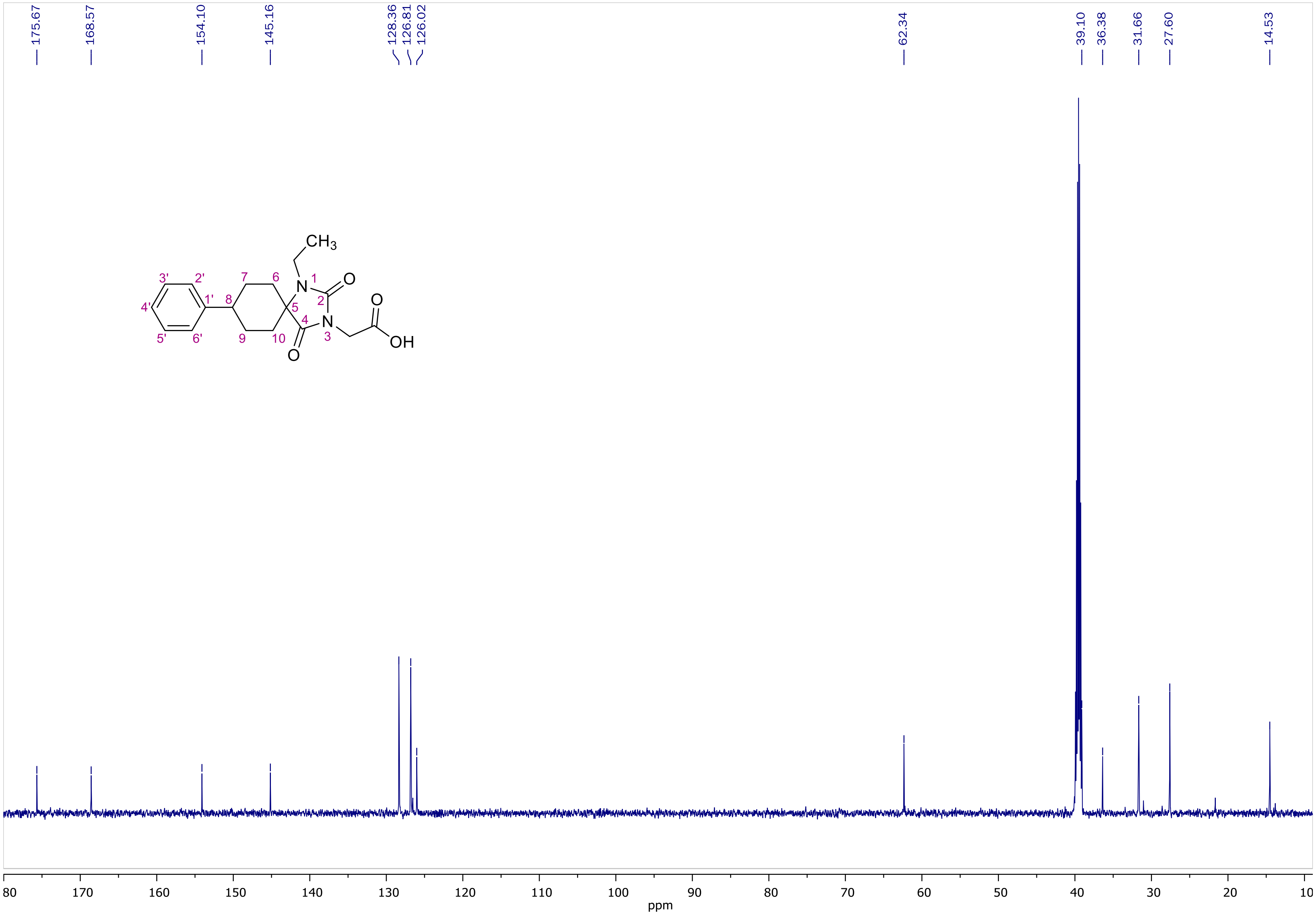

COSY NMR of **22** (600.11 MHz, DMSO-d_6)

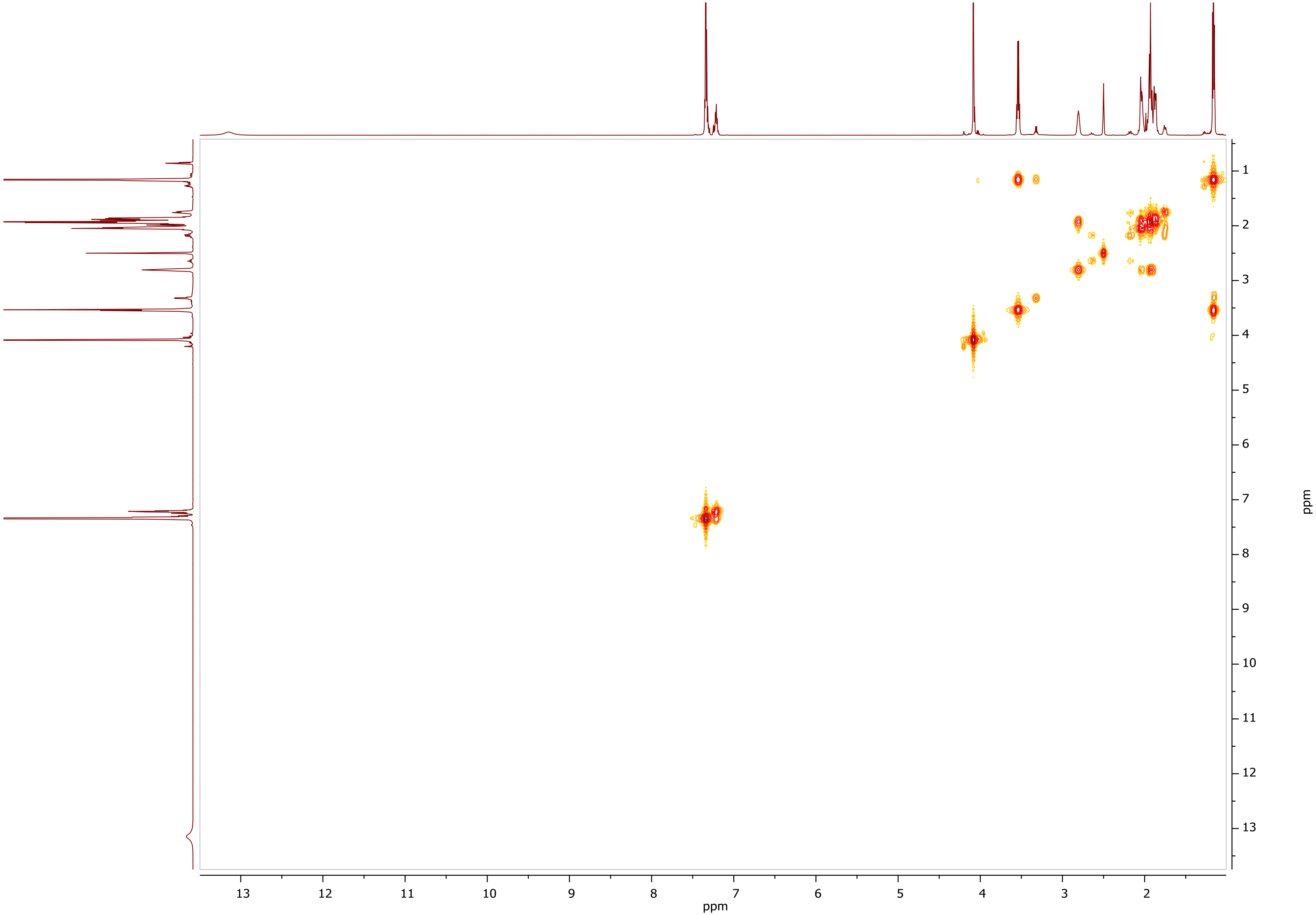

HSQC-DEPT NMR of **22** (600.11 MHz, DMSO-d_6)

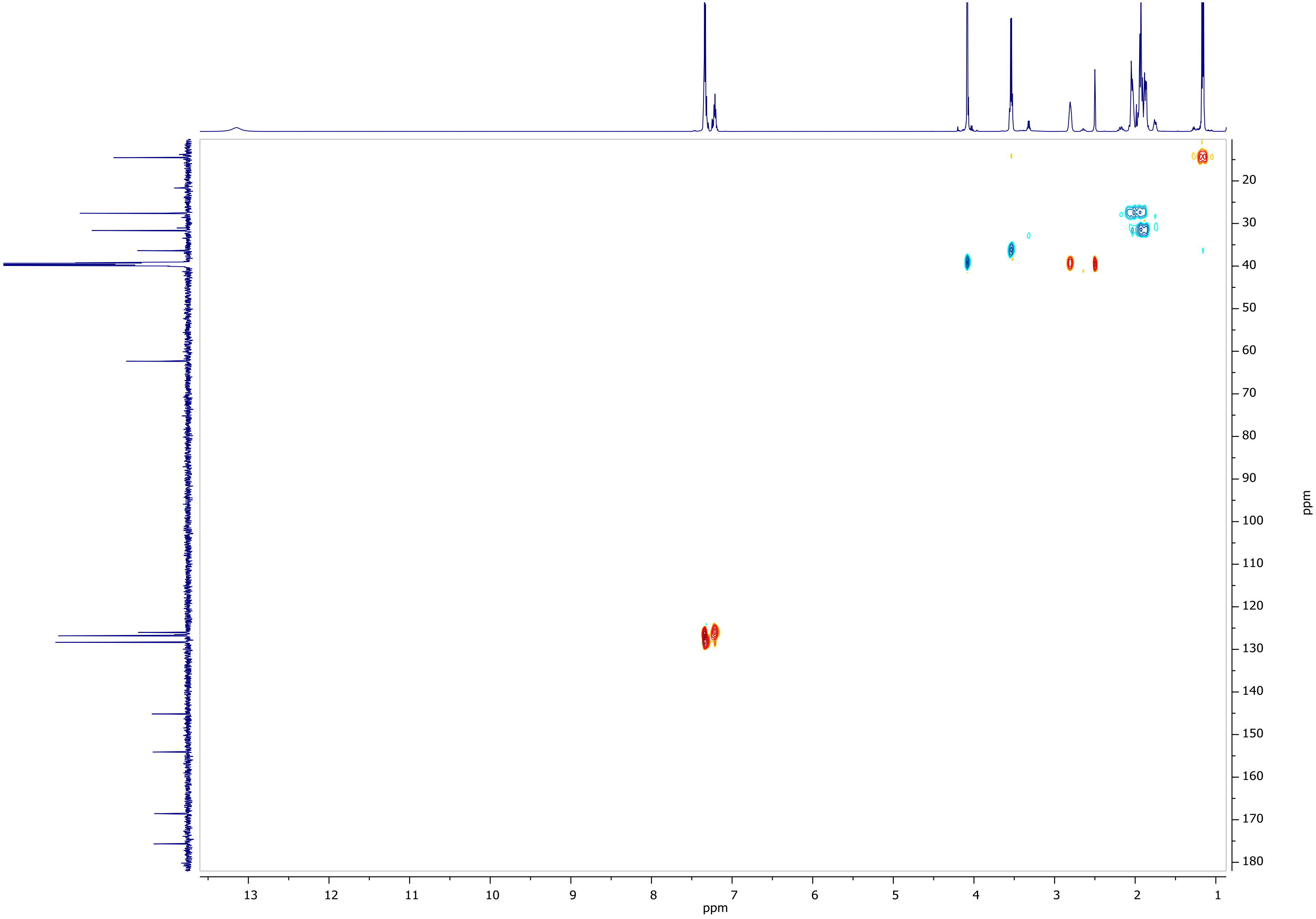

HMBC NMR of **22** (600.11 MHz, DMSO-d_6)

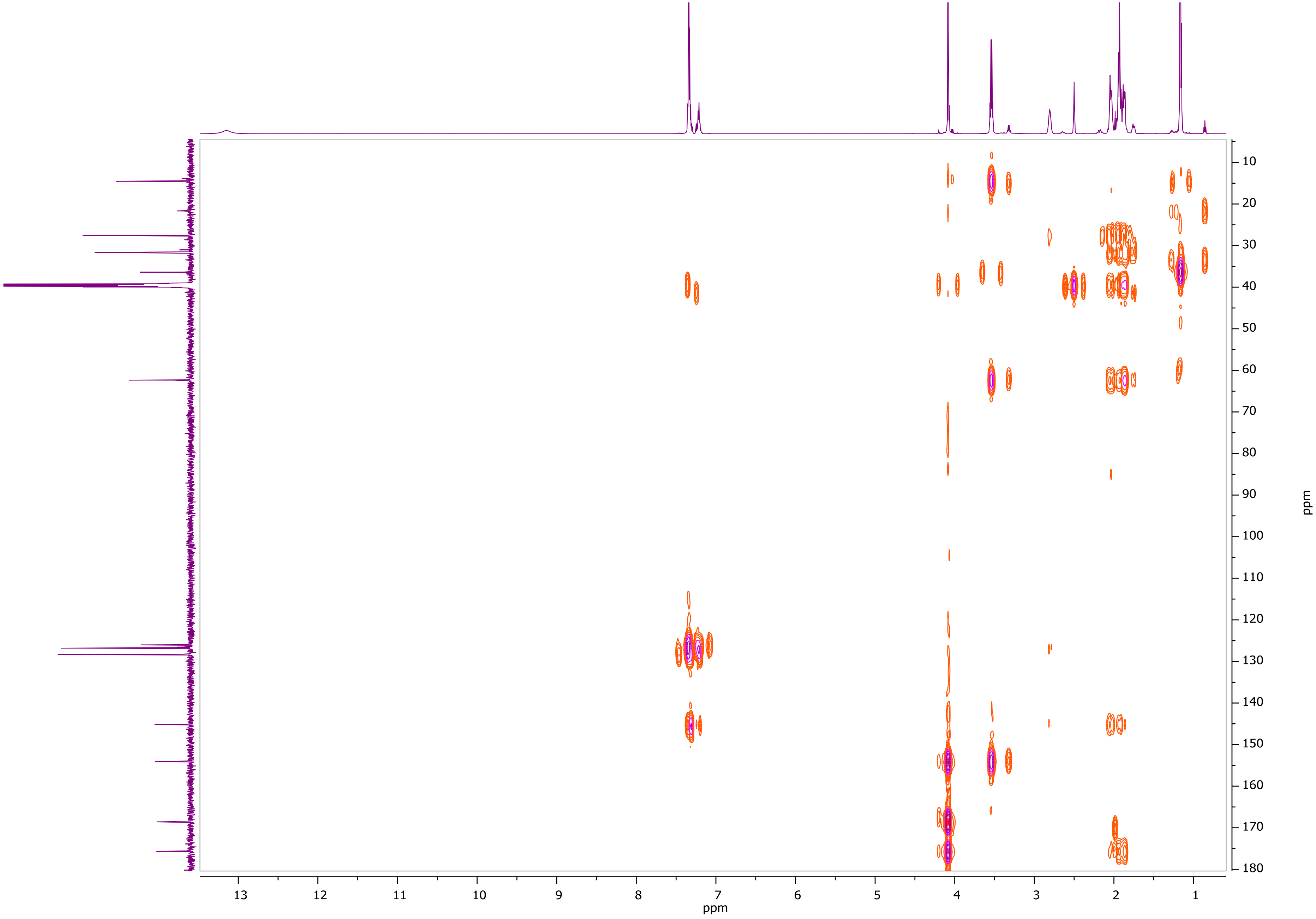

^{1}H NMR of **23** (600.11 MHz, DMSO-d_6)

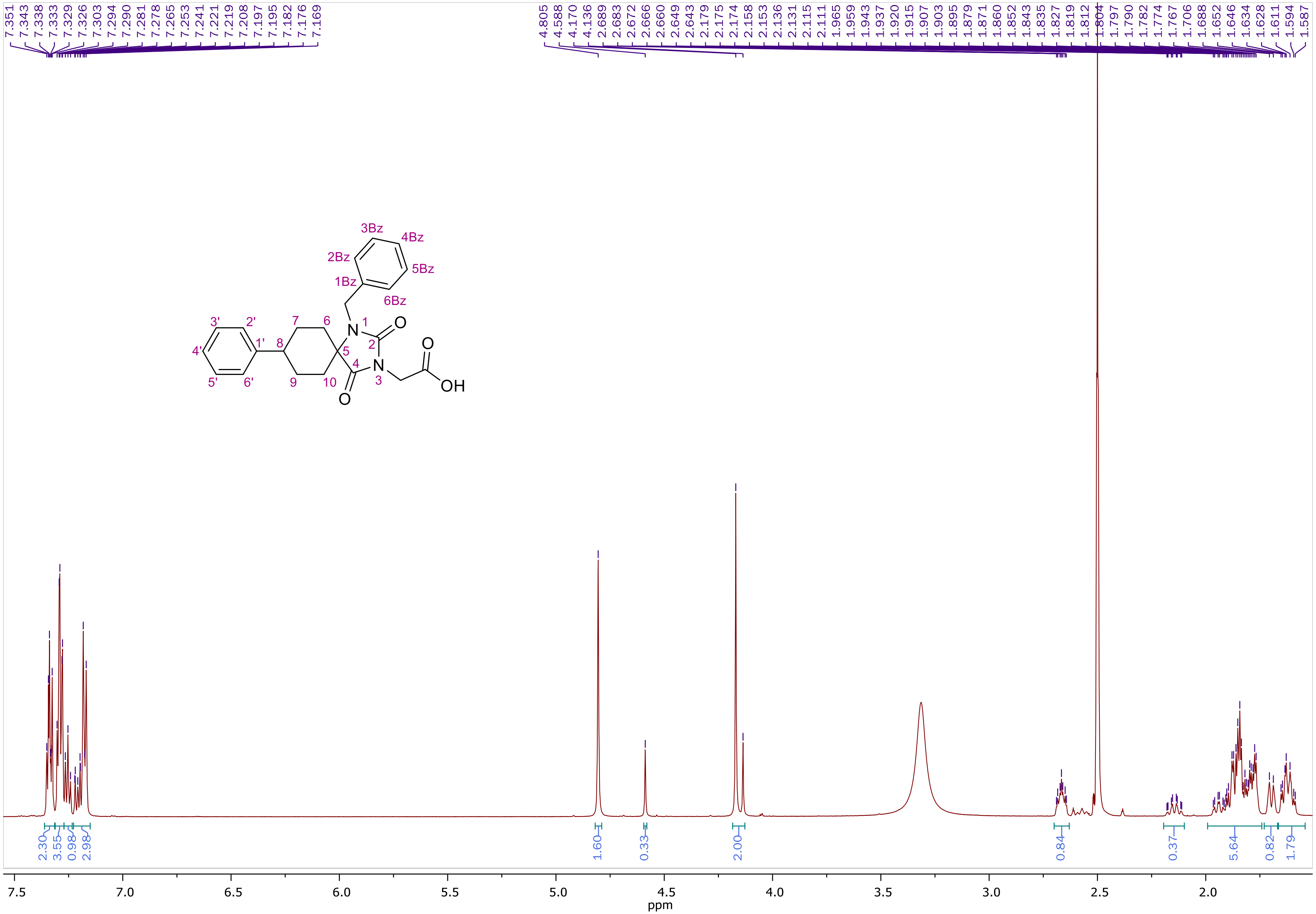

^{13}C NMR of **23** (150.9 MHz, DMSO-d_6)

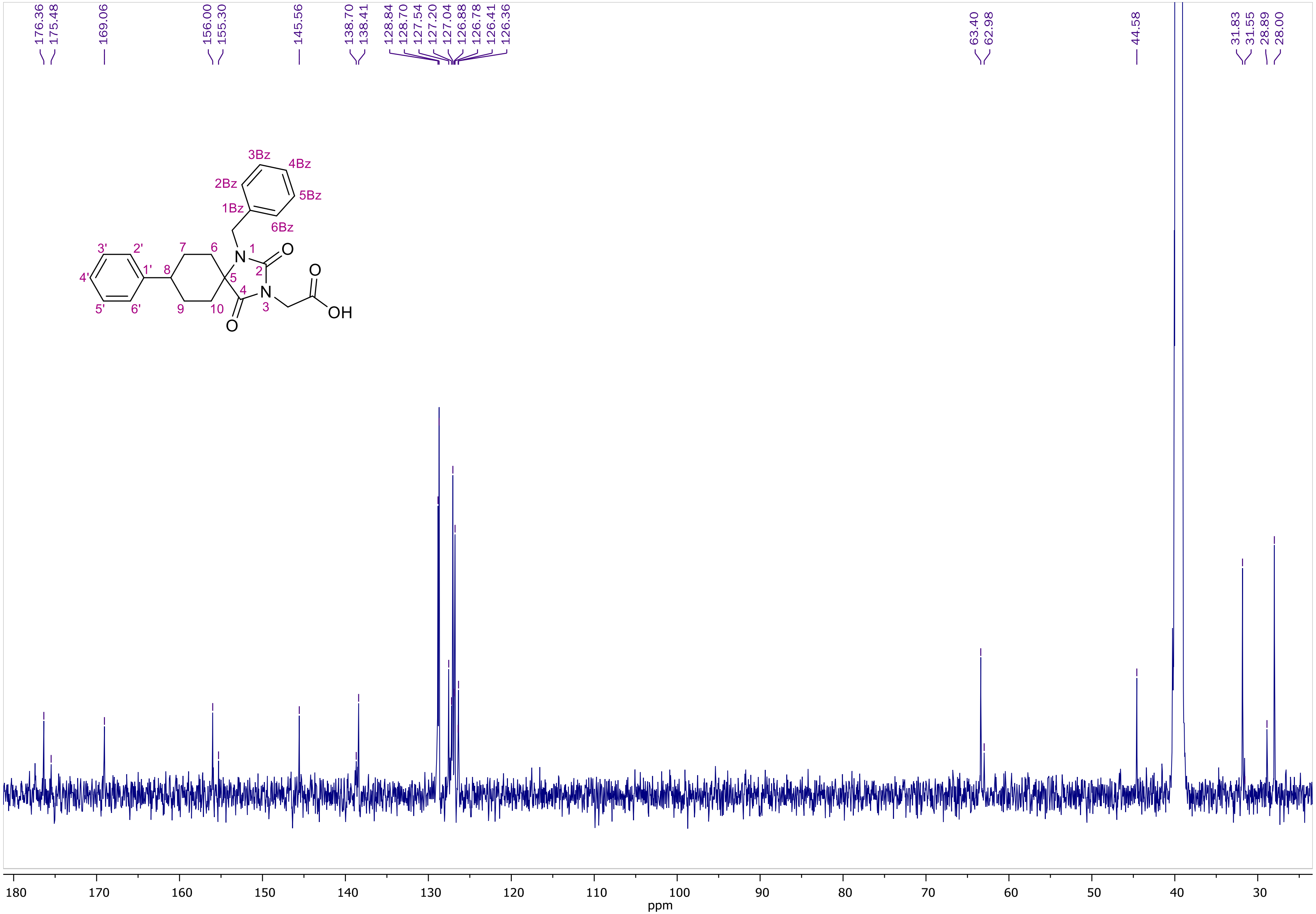

COSY NMR of **23** (600.11 MHz, DMSO-d_6)

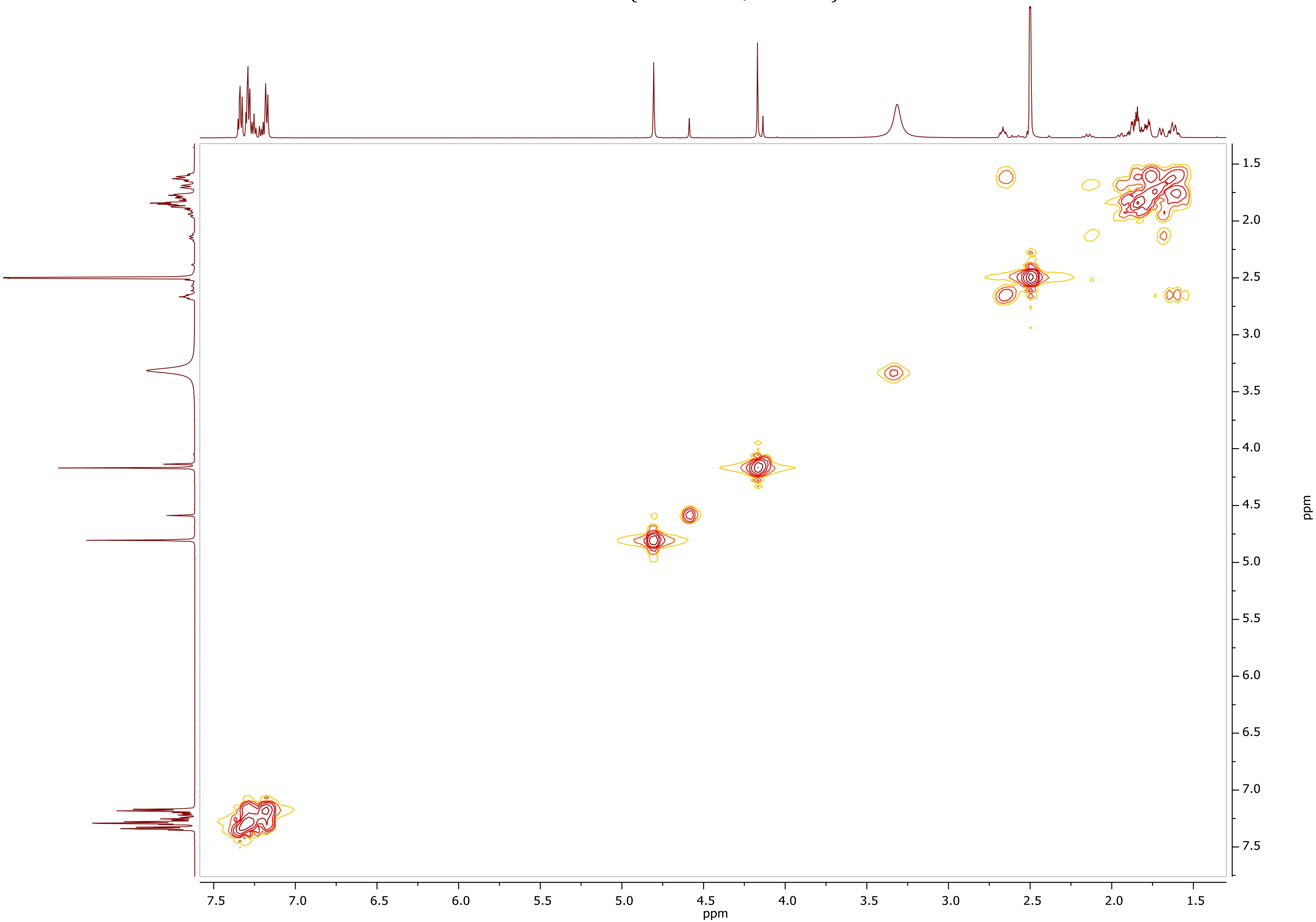

HSQC NMR of **23** (400.13 MHz, DMSO-d_6)

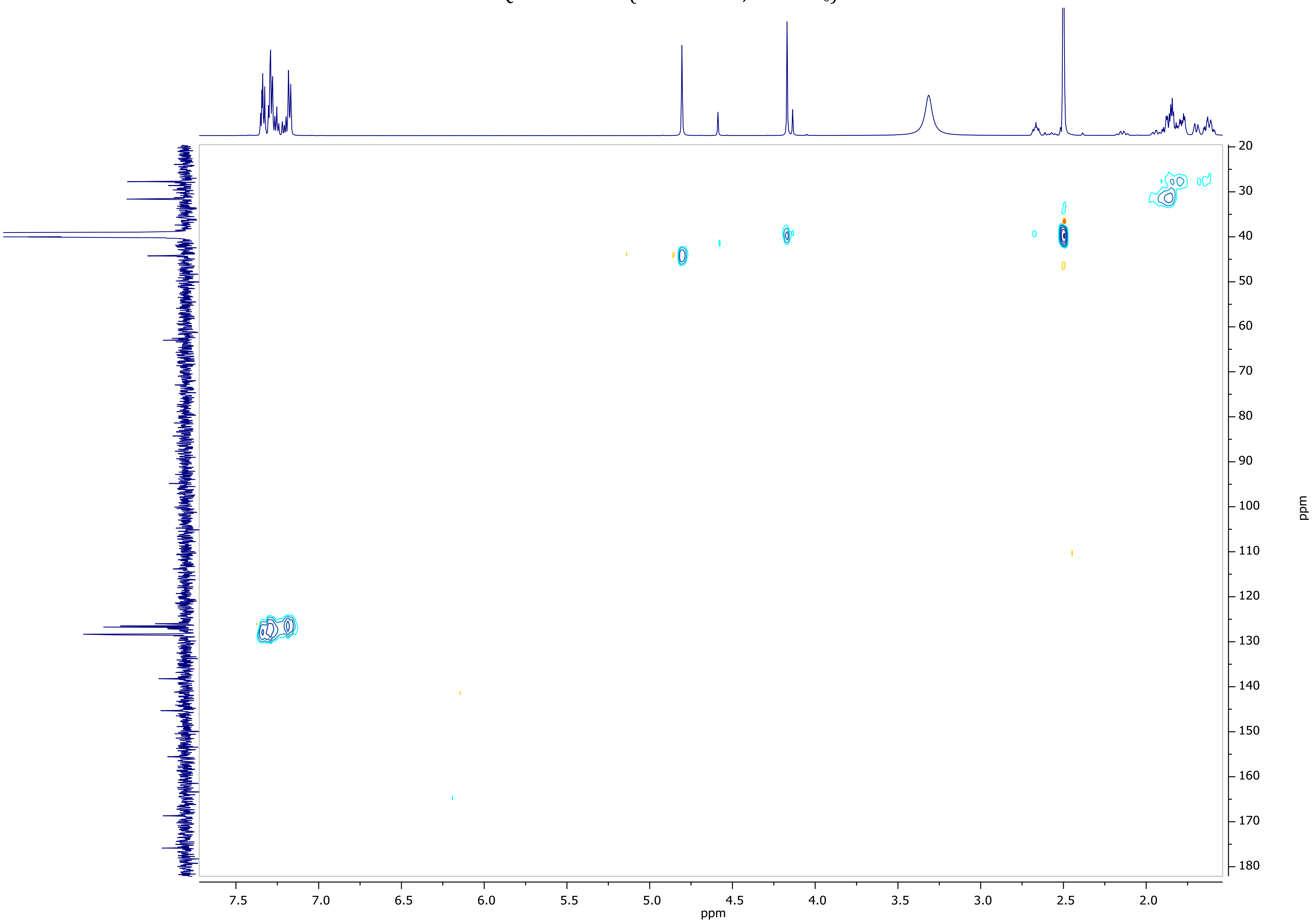

HMBC NMR of **23** (600.11 MHz, DMSO-d_6)

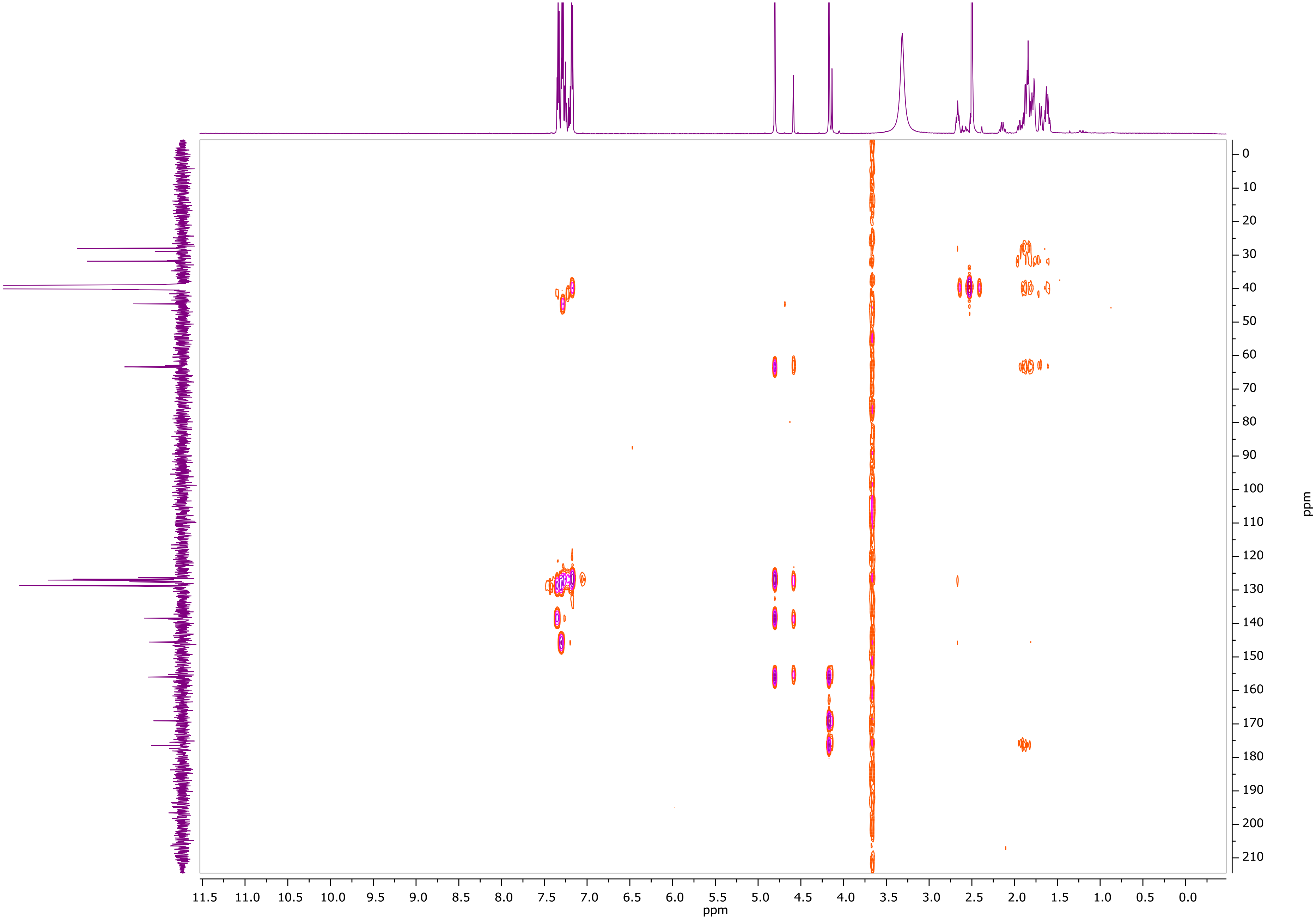

^{1}H NMR of **24** (600.11 MHz, DMSO-d_6)

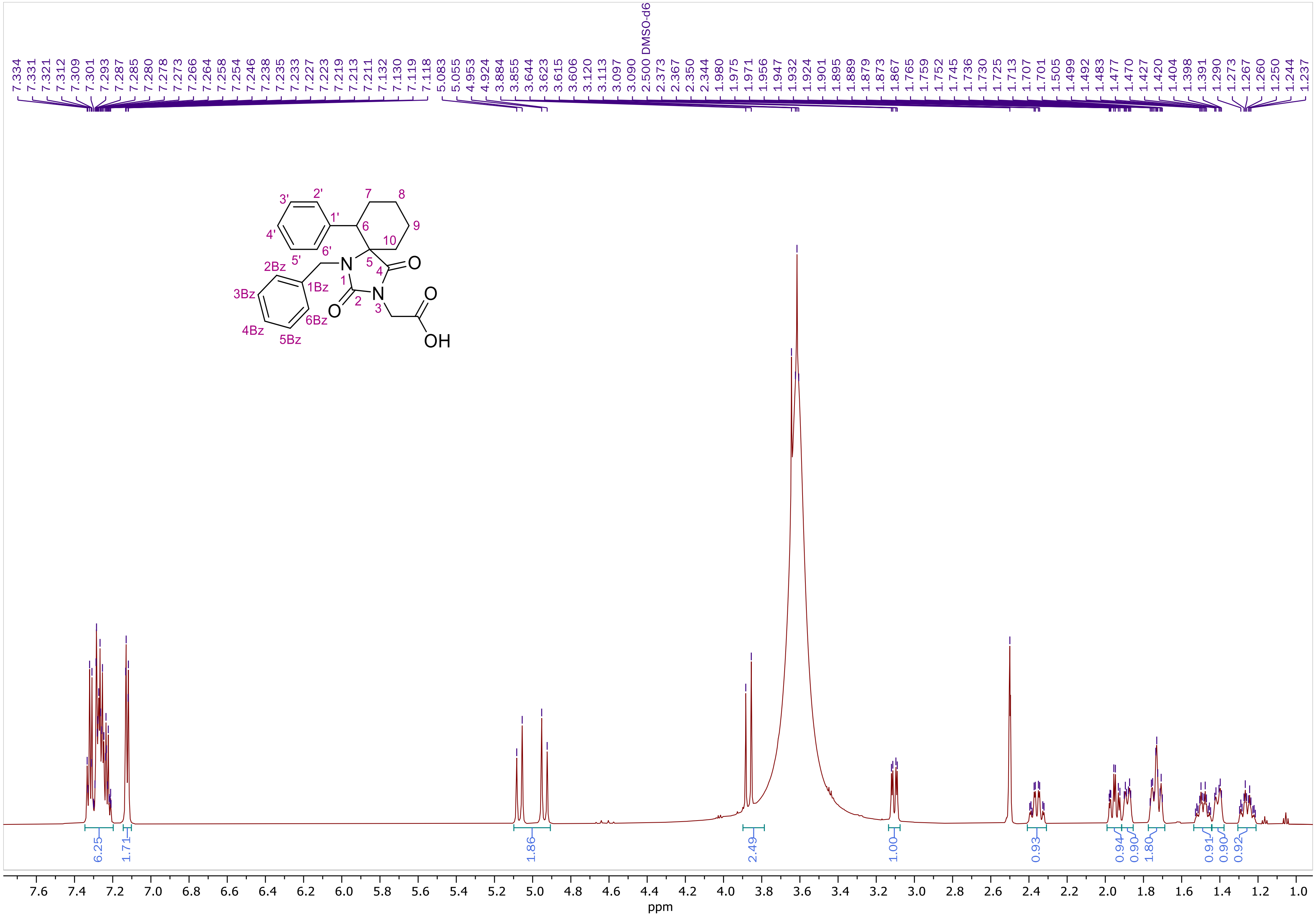

^{13}C NMR of **24** (50.32 MHz, DMSO-d_6)

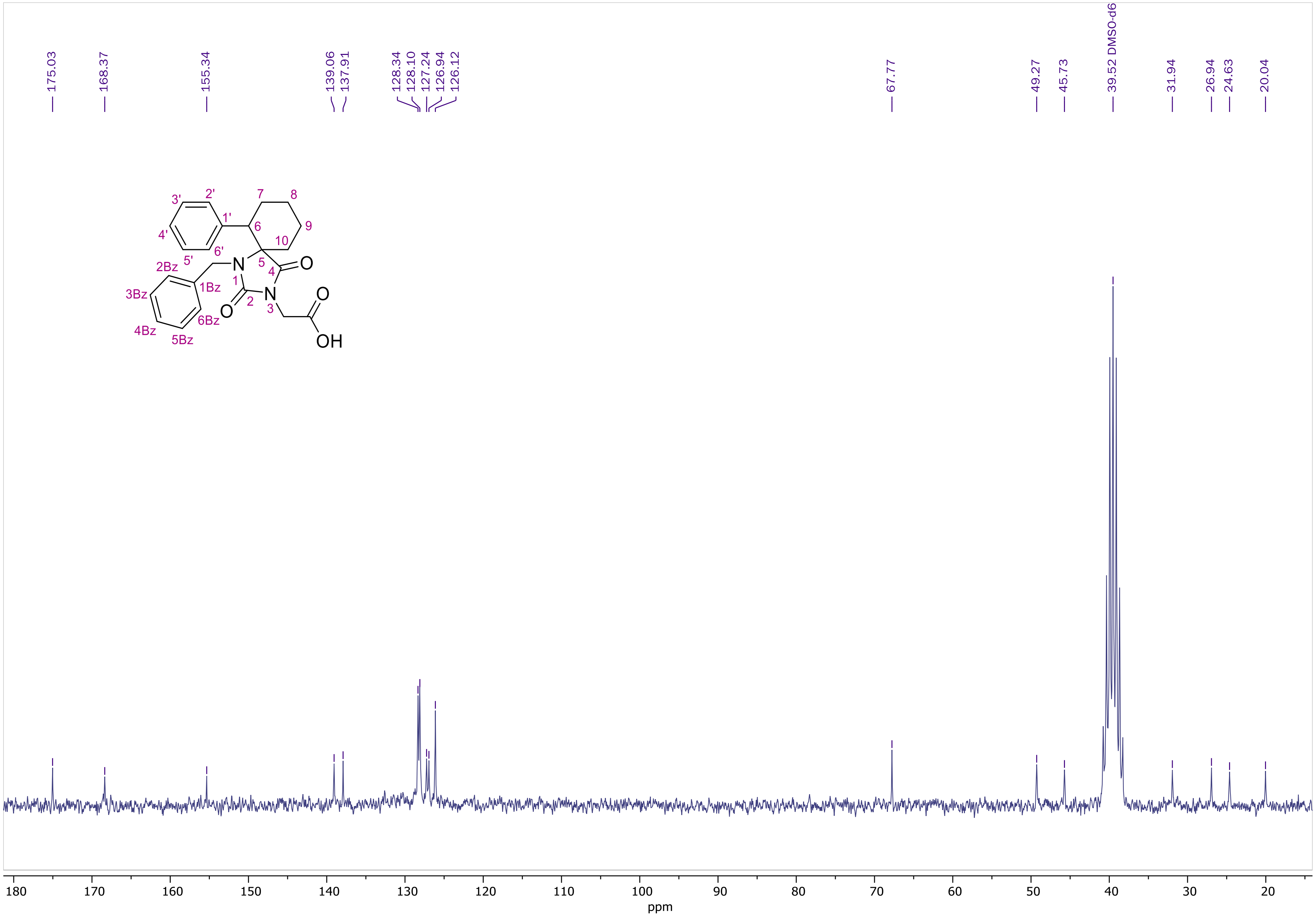

COSY NMR of **24** (400.13 MHz, DMSO-d_6)

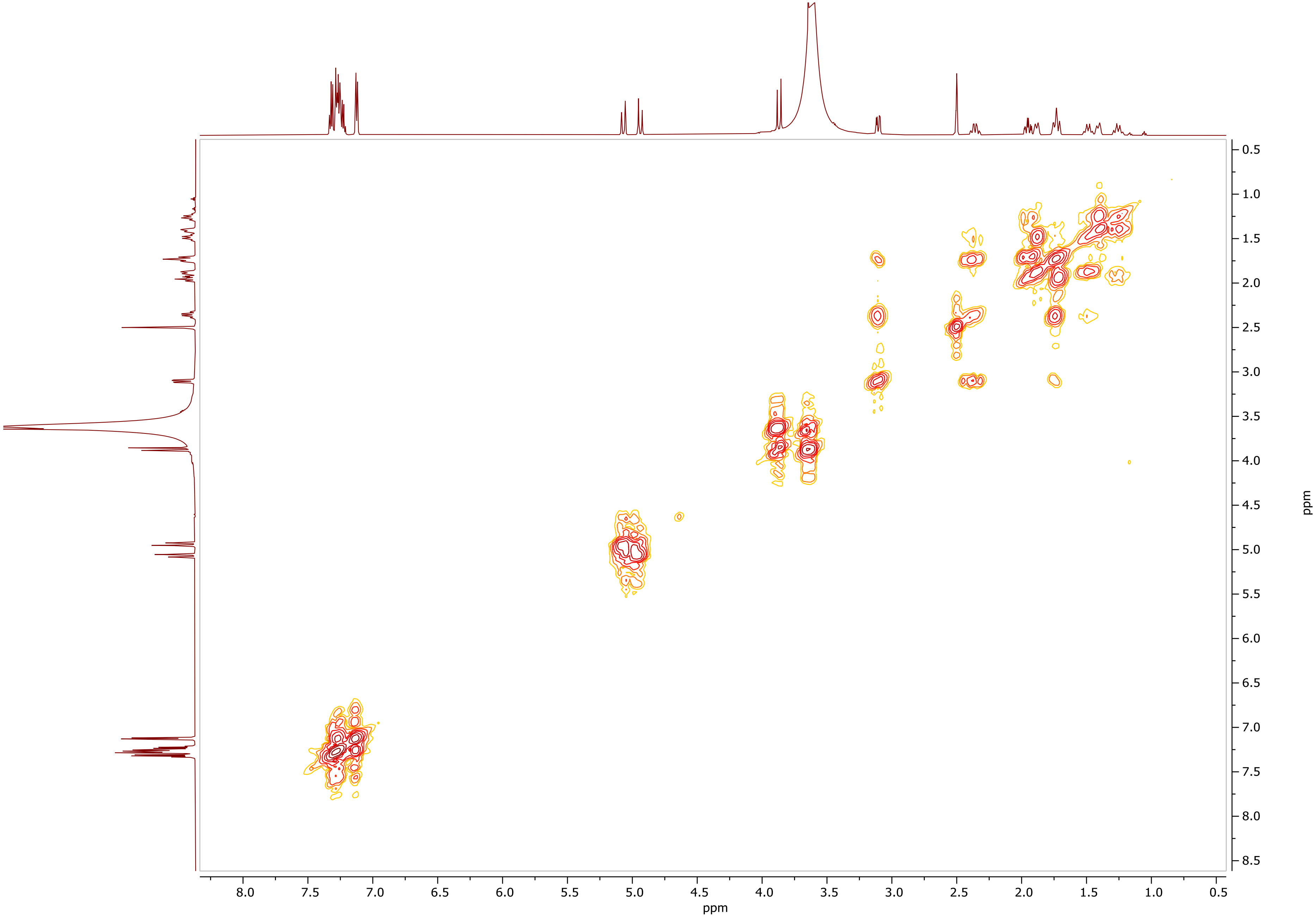

HSQC NMR of **24** (400.13 MHz, DMSO-d_6)

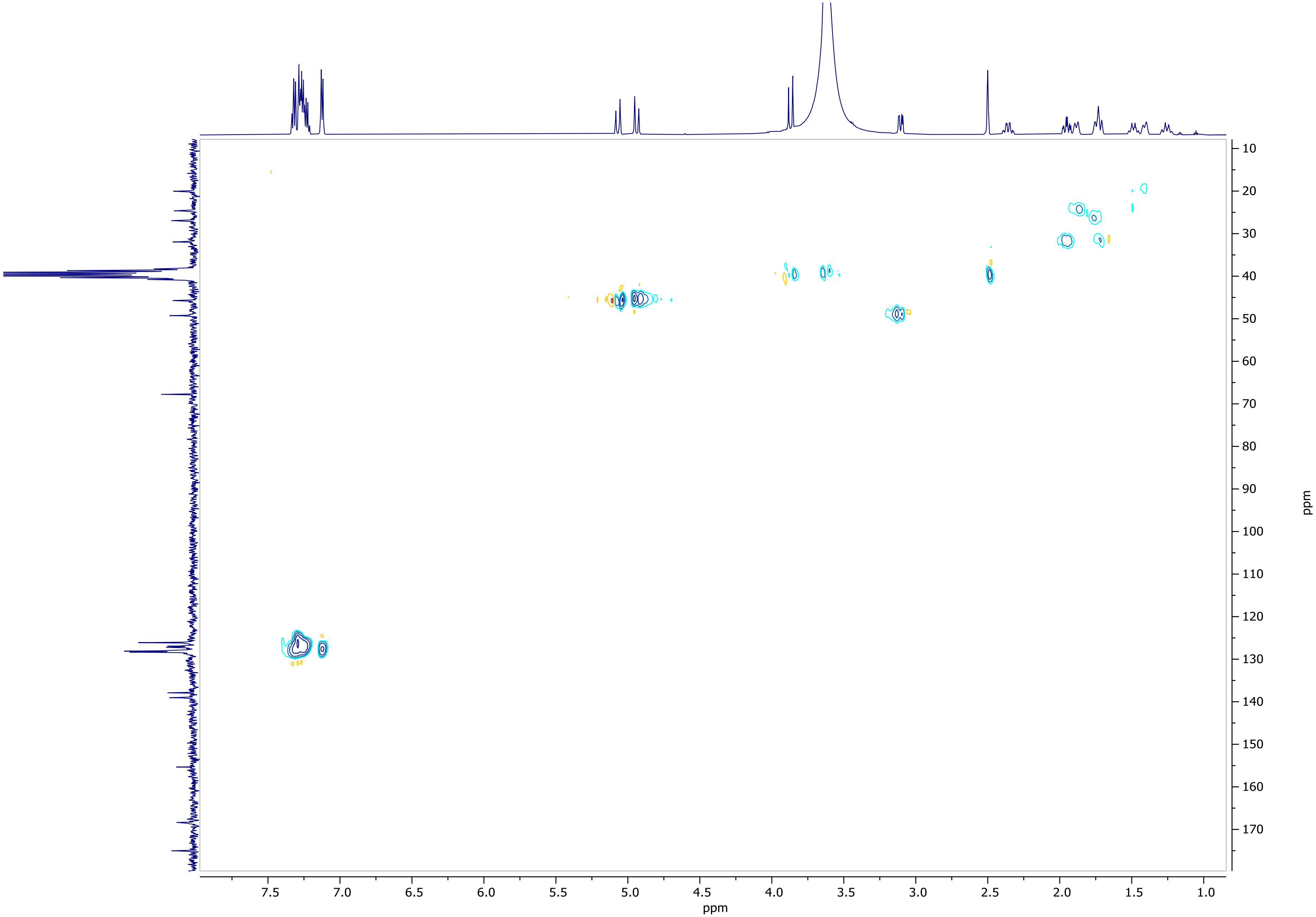

HMBC NMR of **24** (400.13 MHz, DMSO-d_6)

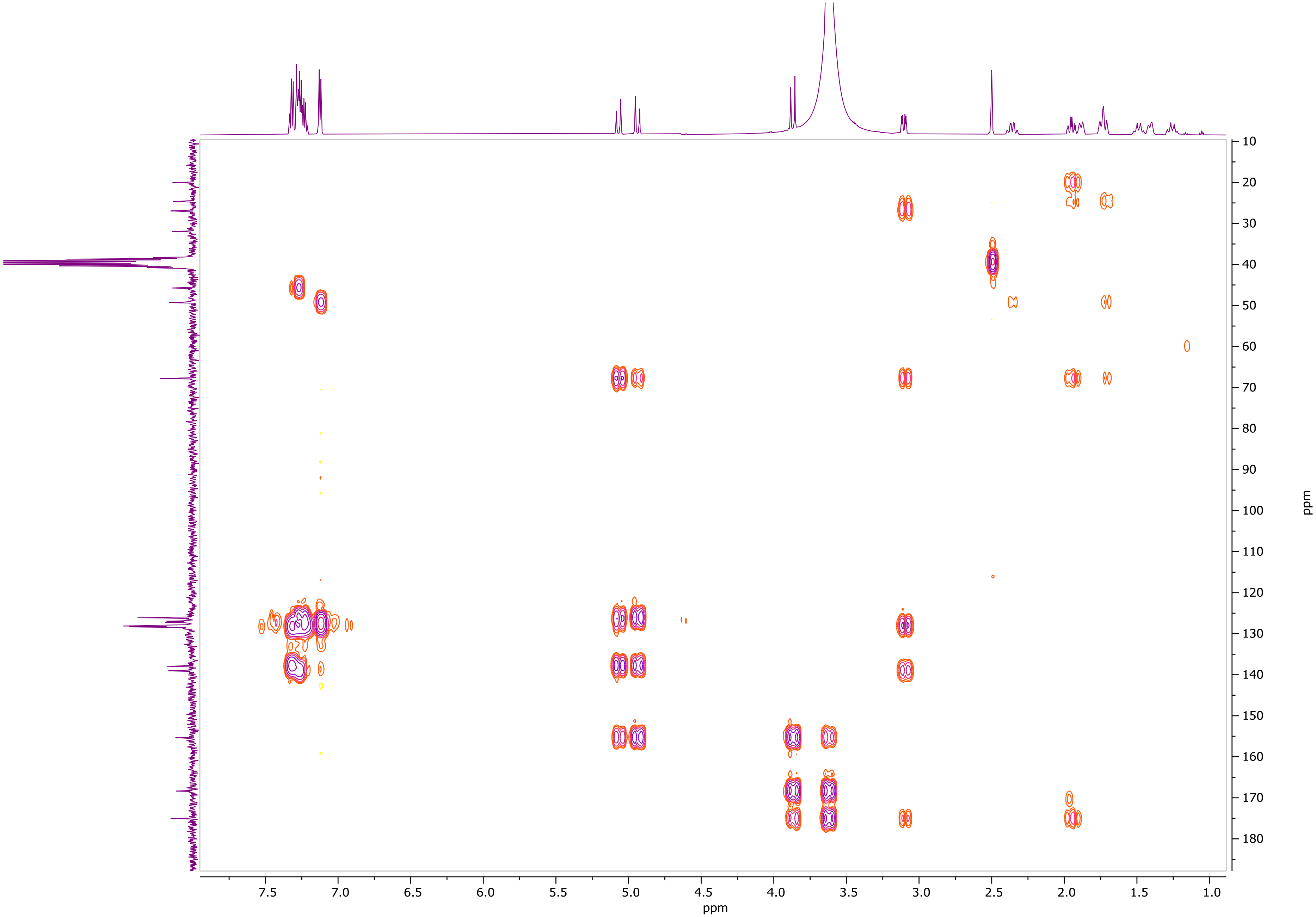

^1H NMR of **25** (400.13 MHz, DMSO-d_6)

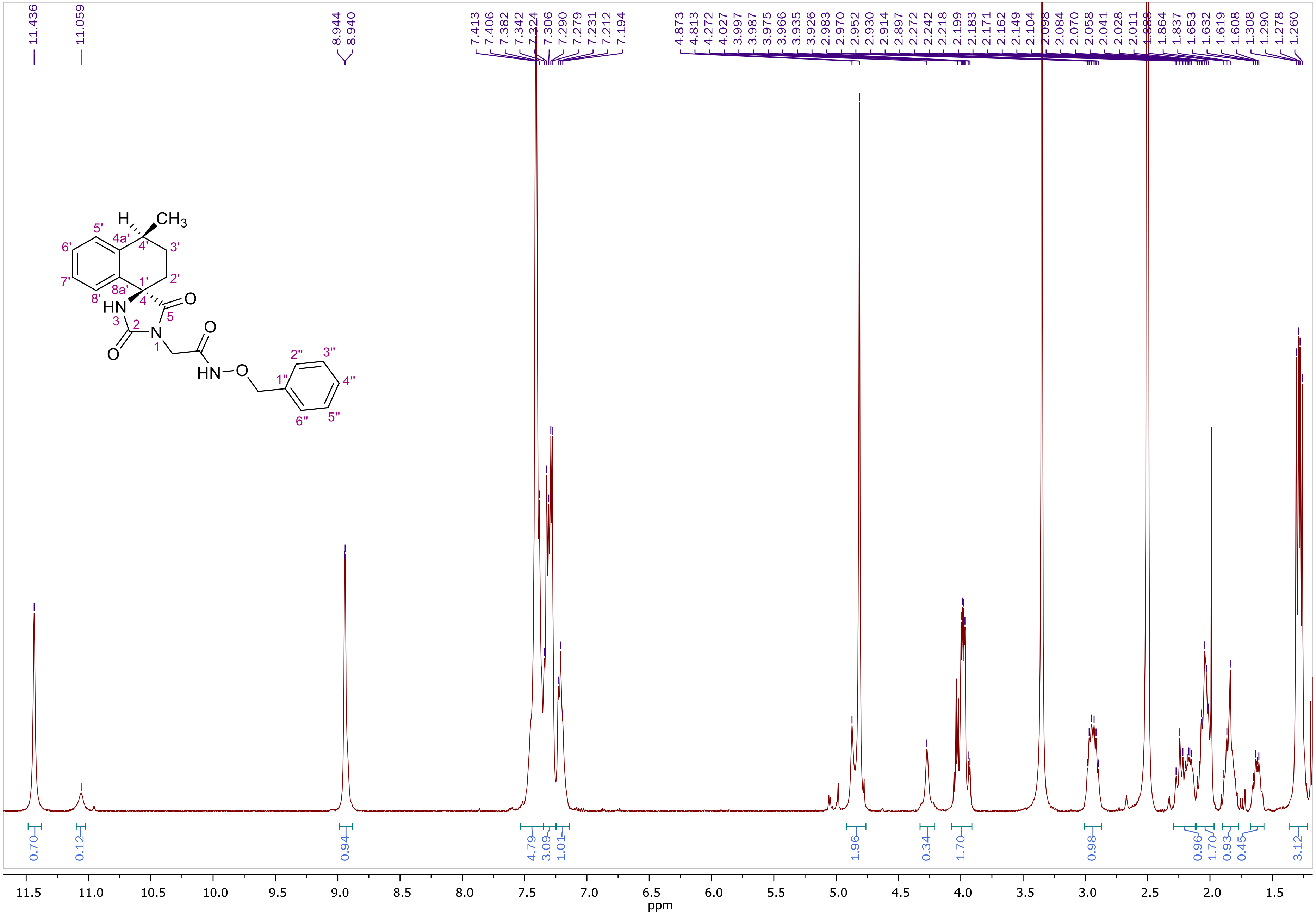

^{13}C NMR of **25** (50.32 MHz, DMSO-d_6)

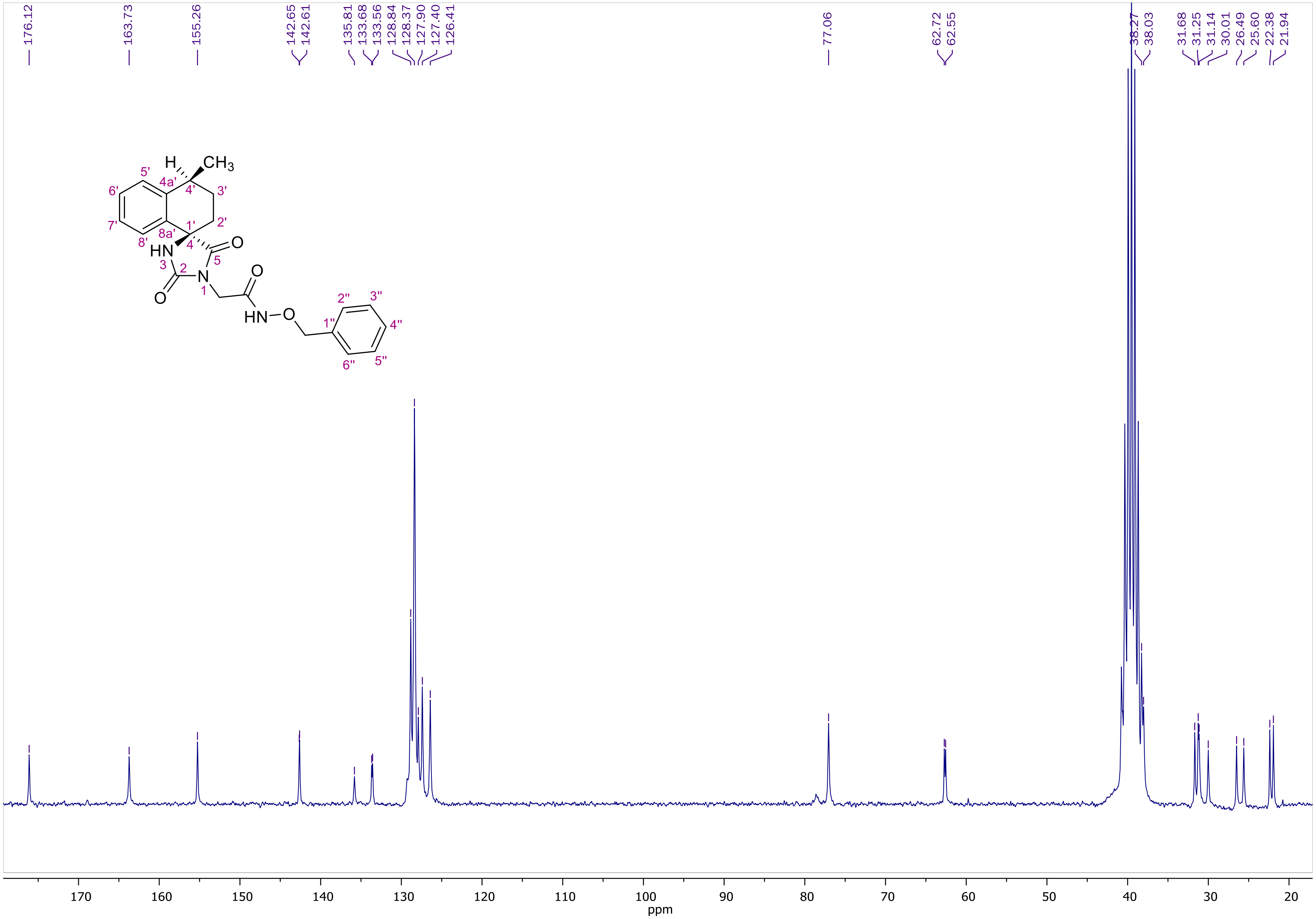

COSY NMR of **25** (400.13 MHz, DMSO-d_6)

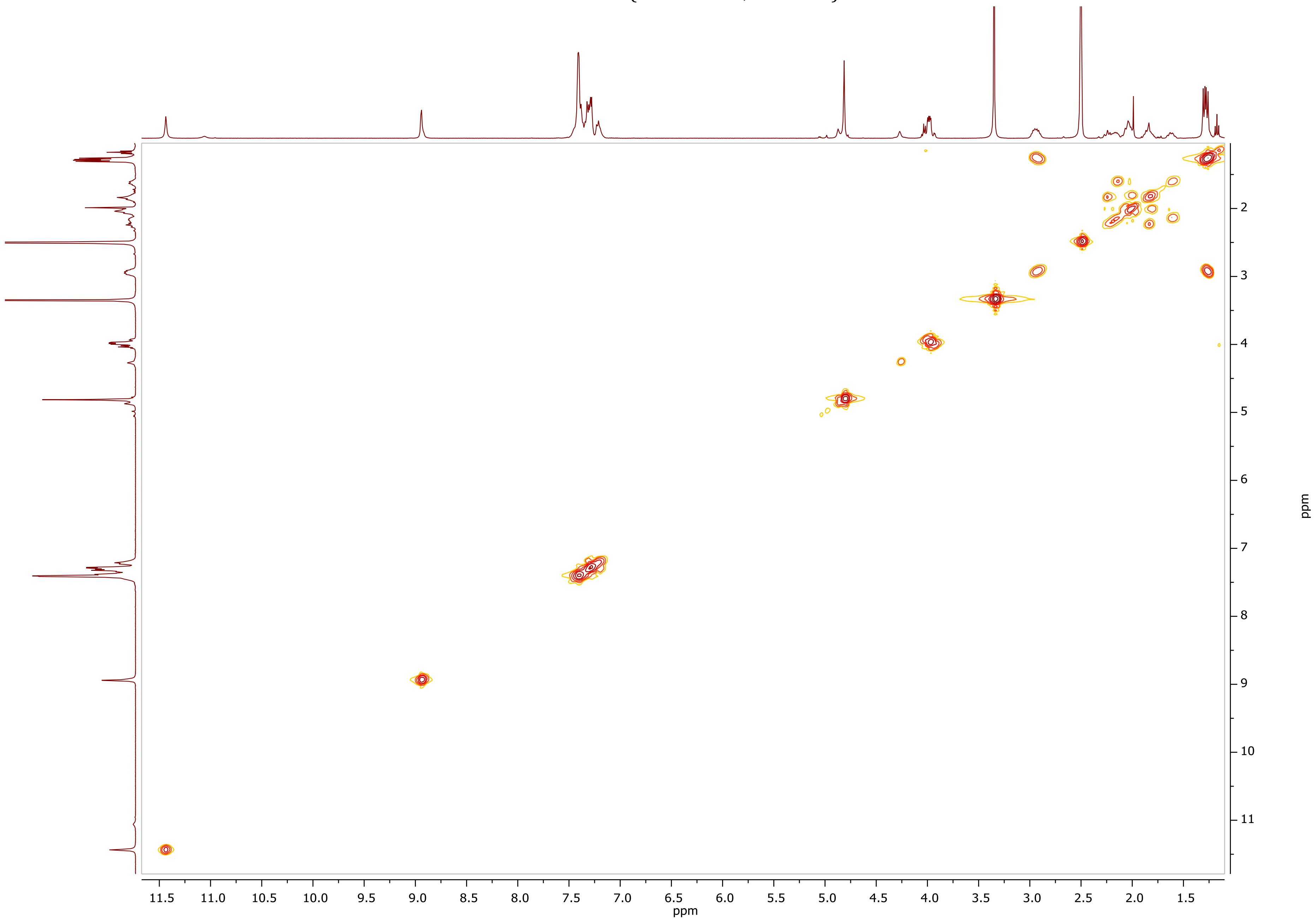

HSQC NMR of **25** (400.13 MHz, DMSO-d_6)

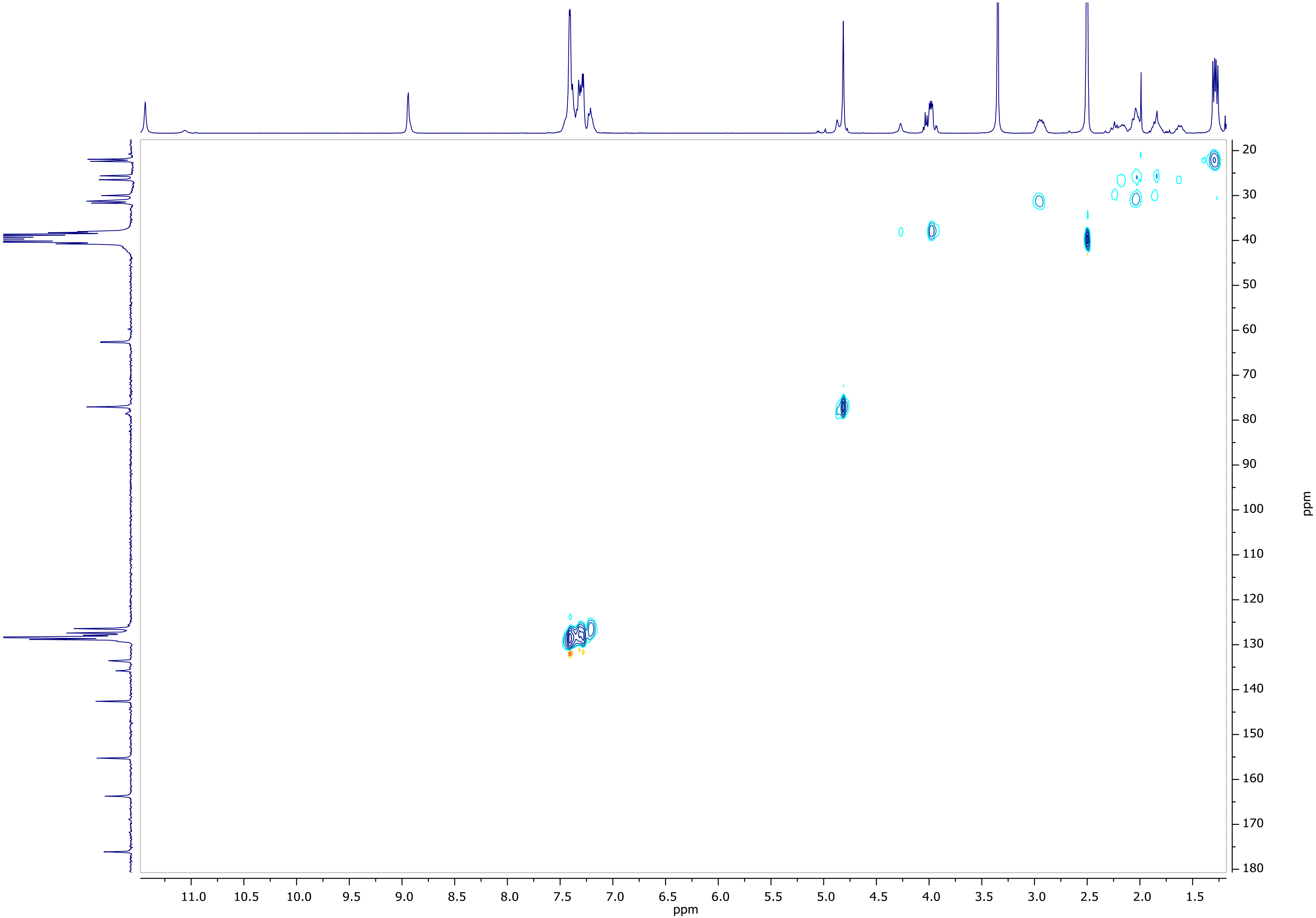

HMBC NMR of **25** (400.13 MHz, DMSO-d_6)

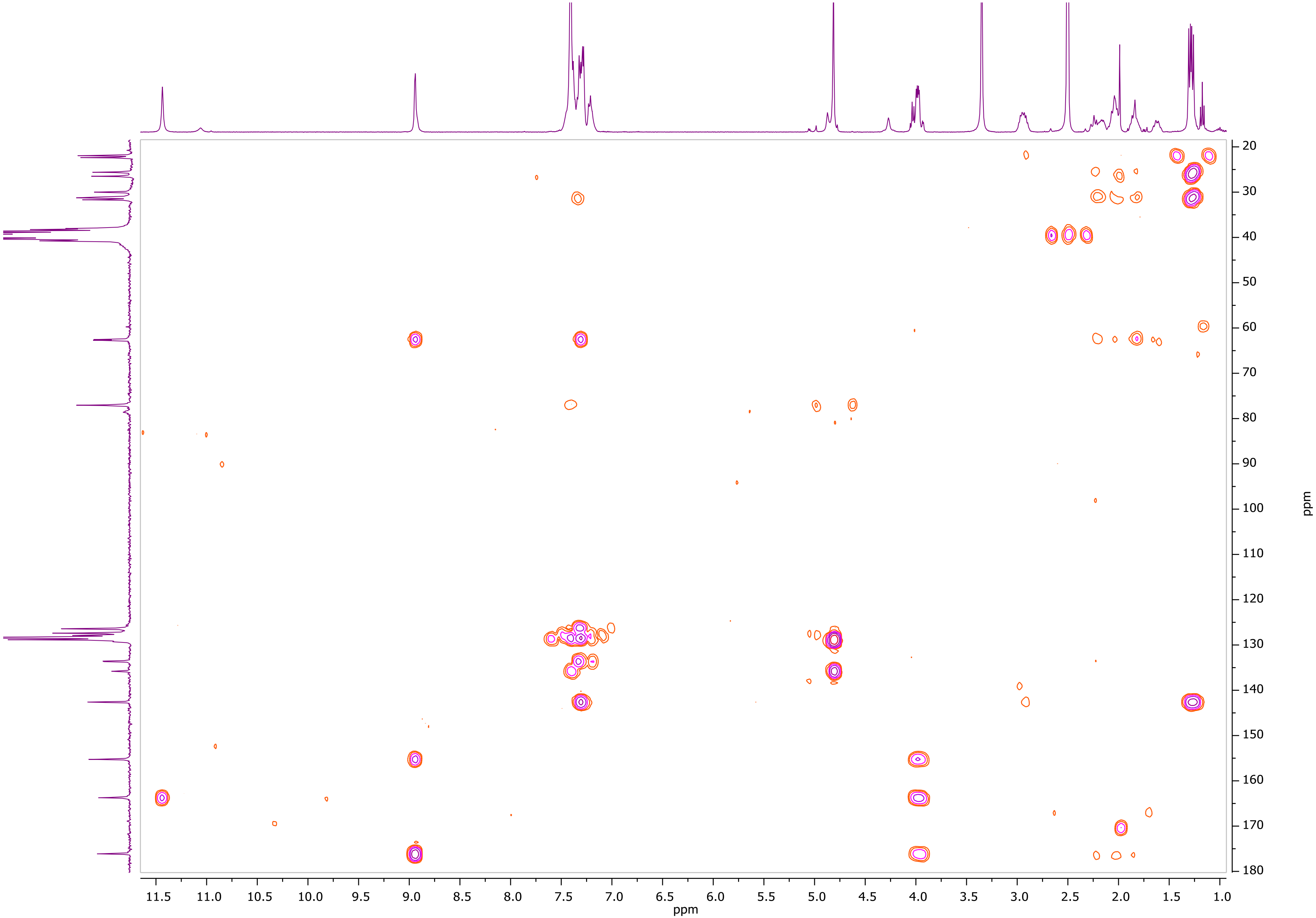

^{1}H NMR of **26** (400.13 MHz, DMSO-d_6)

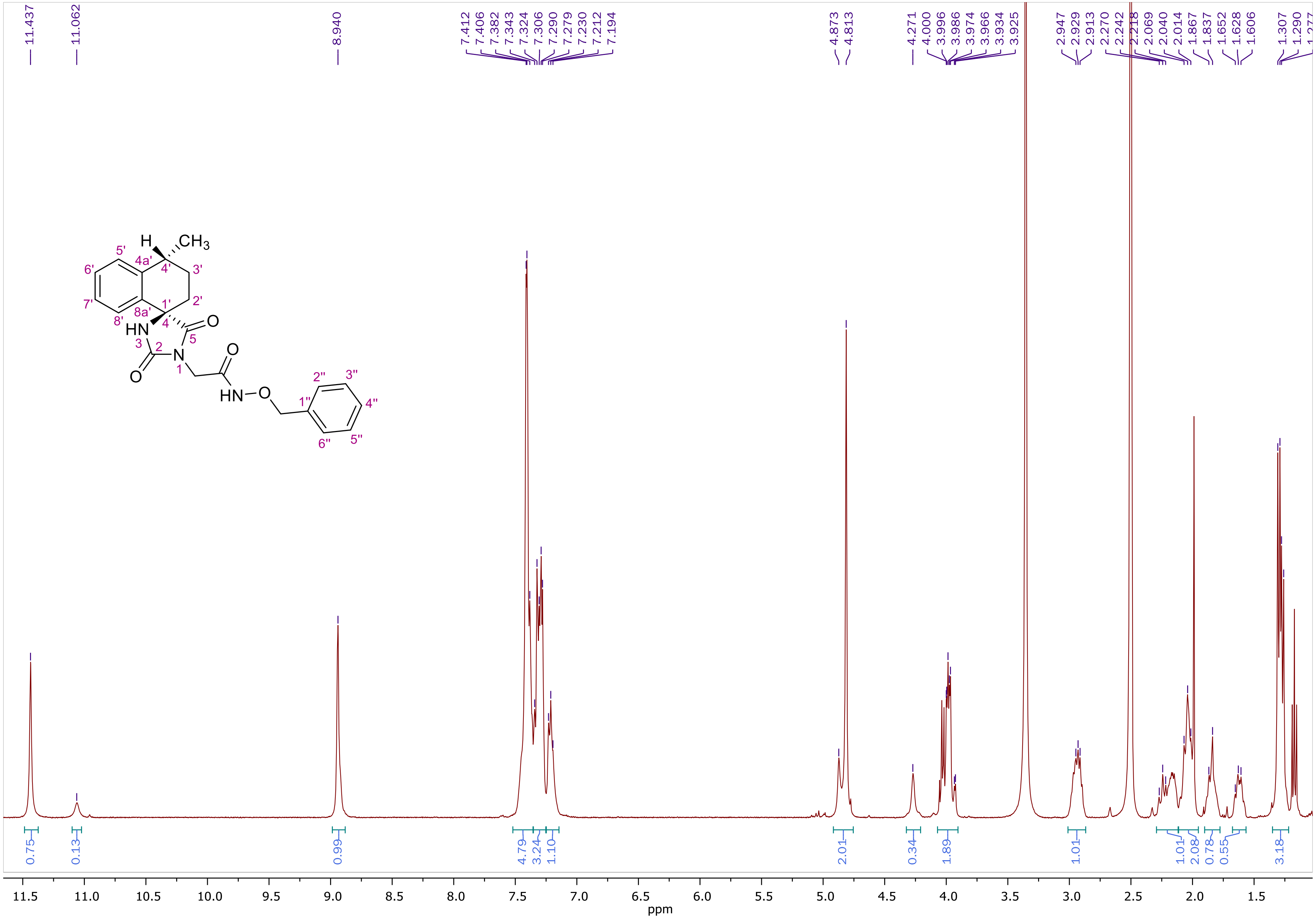

^{13}C NMR of **26** (50.32 MHz, DMSO-d_6)

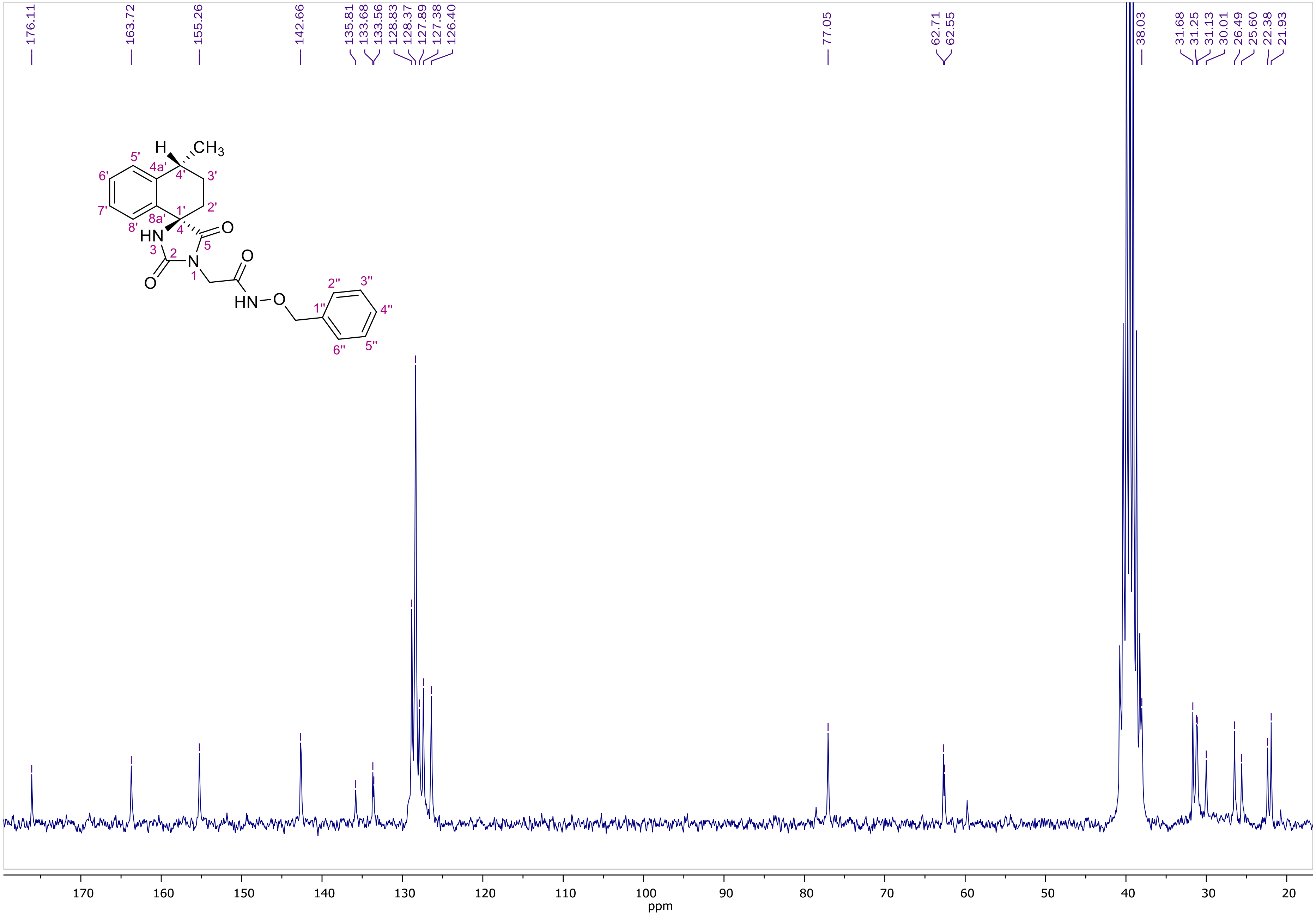

COSY NMR of **26** (400.13 MHz, DMSO-d_6)

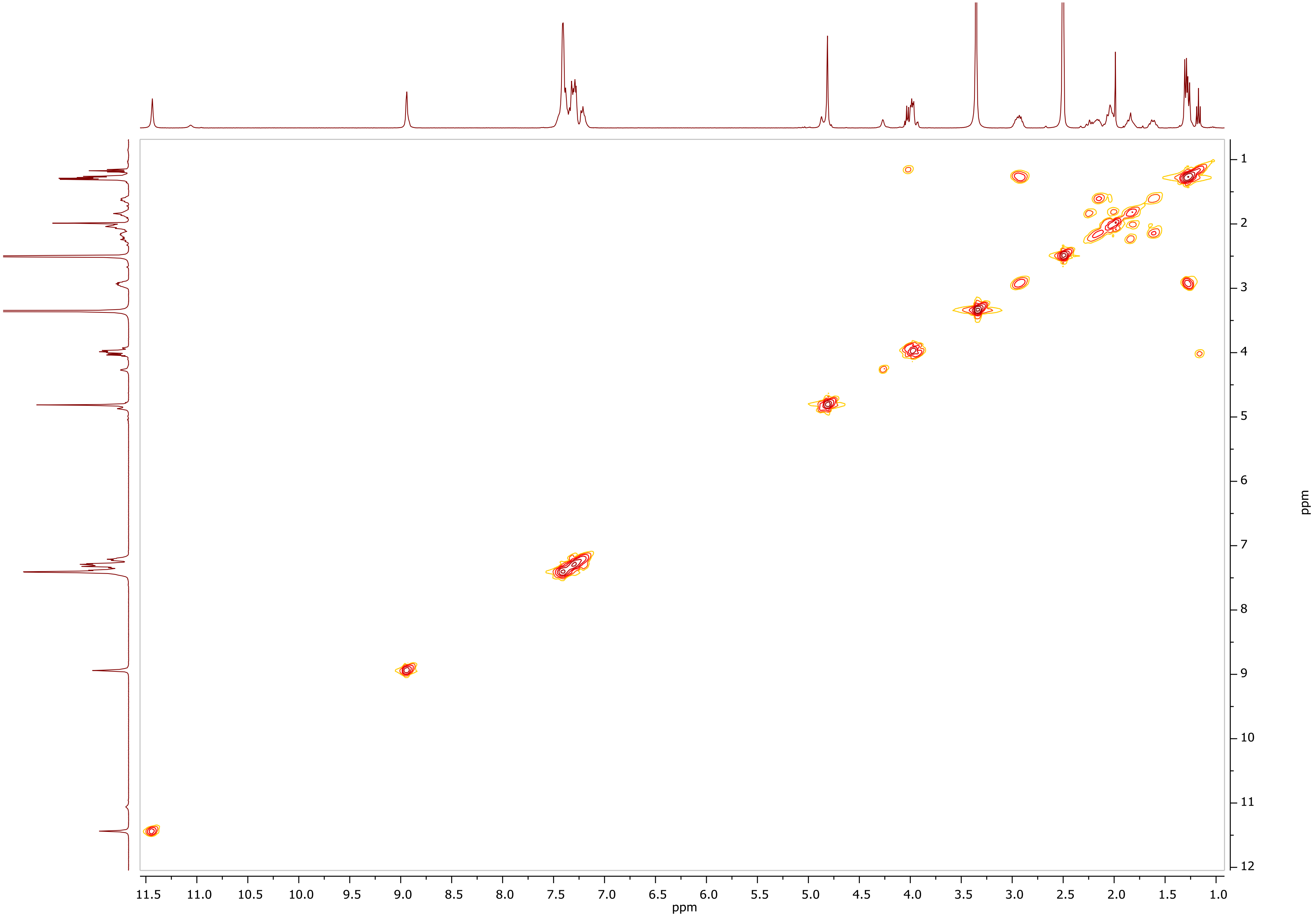

HSQC NMR of **26** (400.13 MHz, DMSO-d_6)

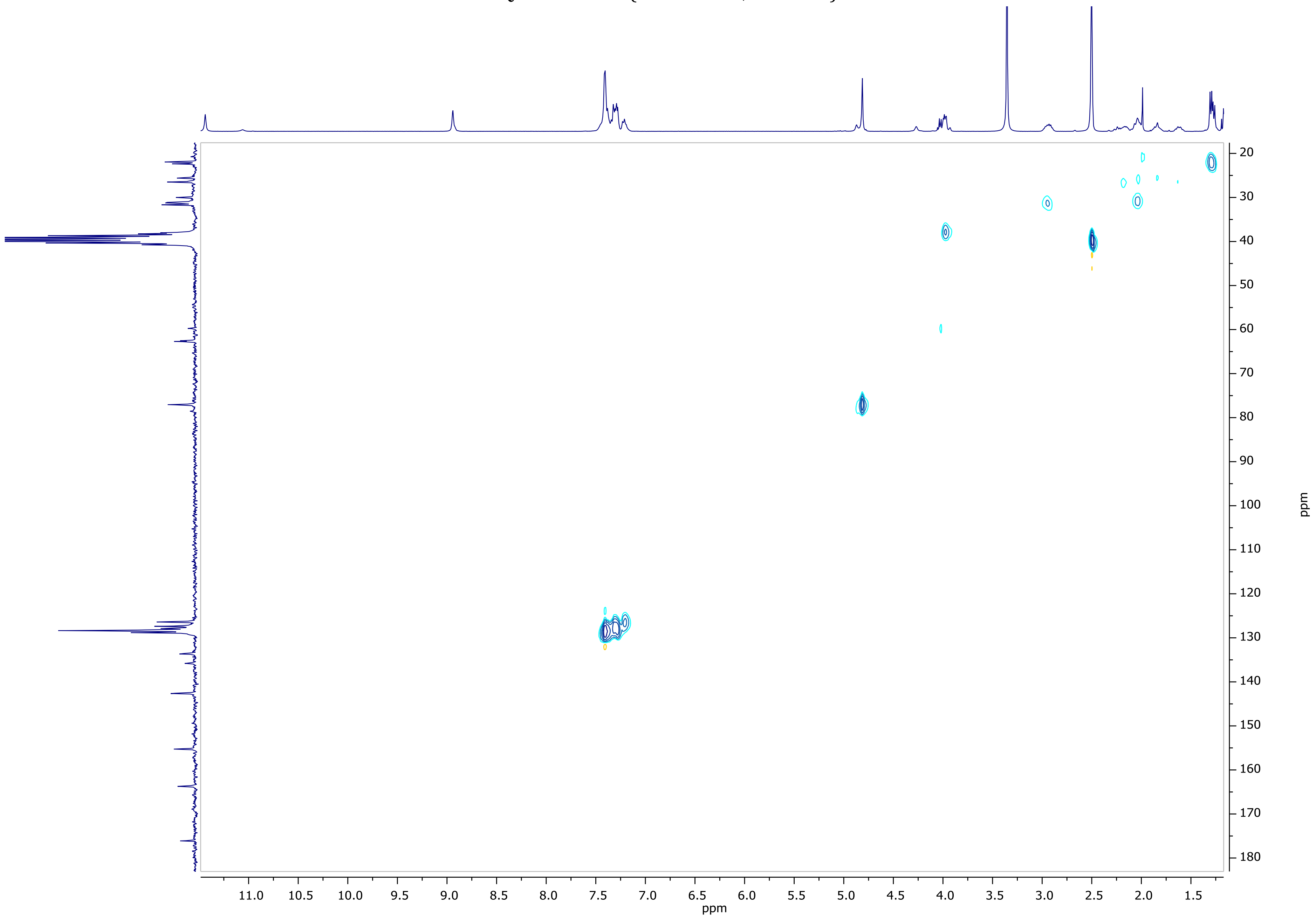

HMBC NMR of **26** (400.13 MHz, DMSO-d_6)

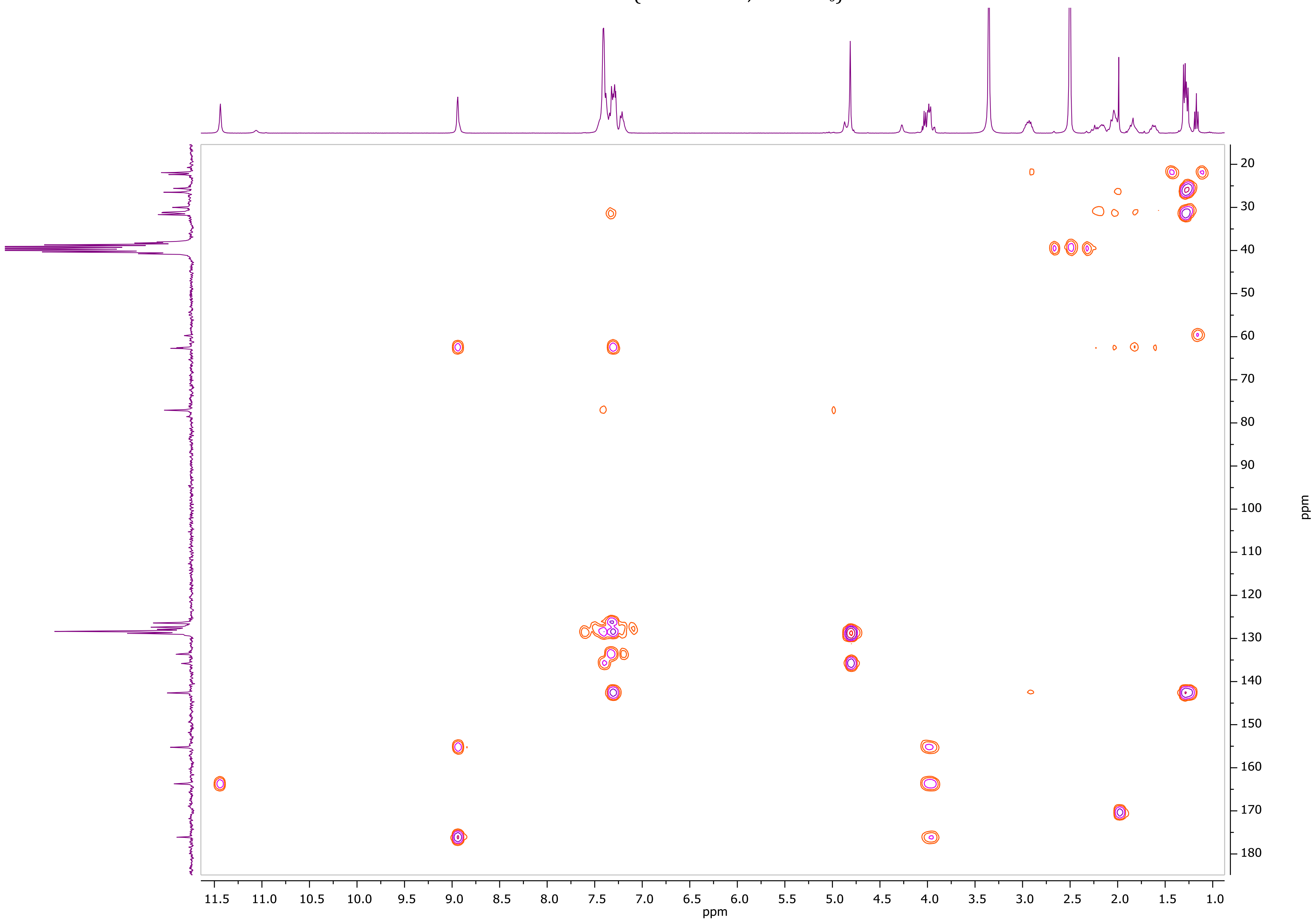

^{1}H NMR of **27** (600.11 MHz, DMSO-d_6)

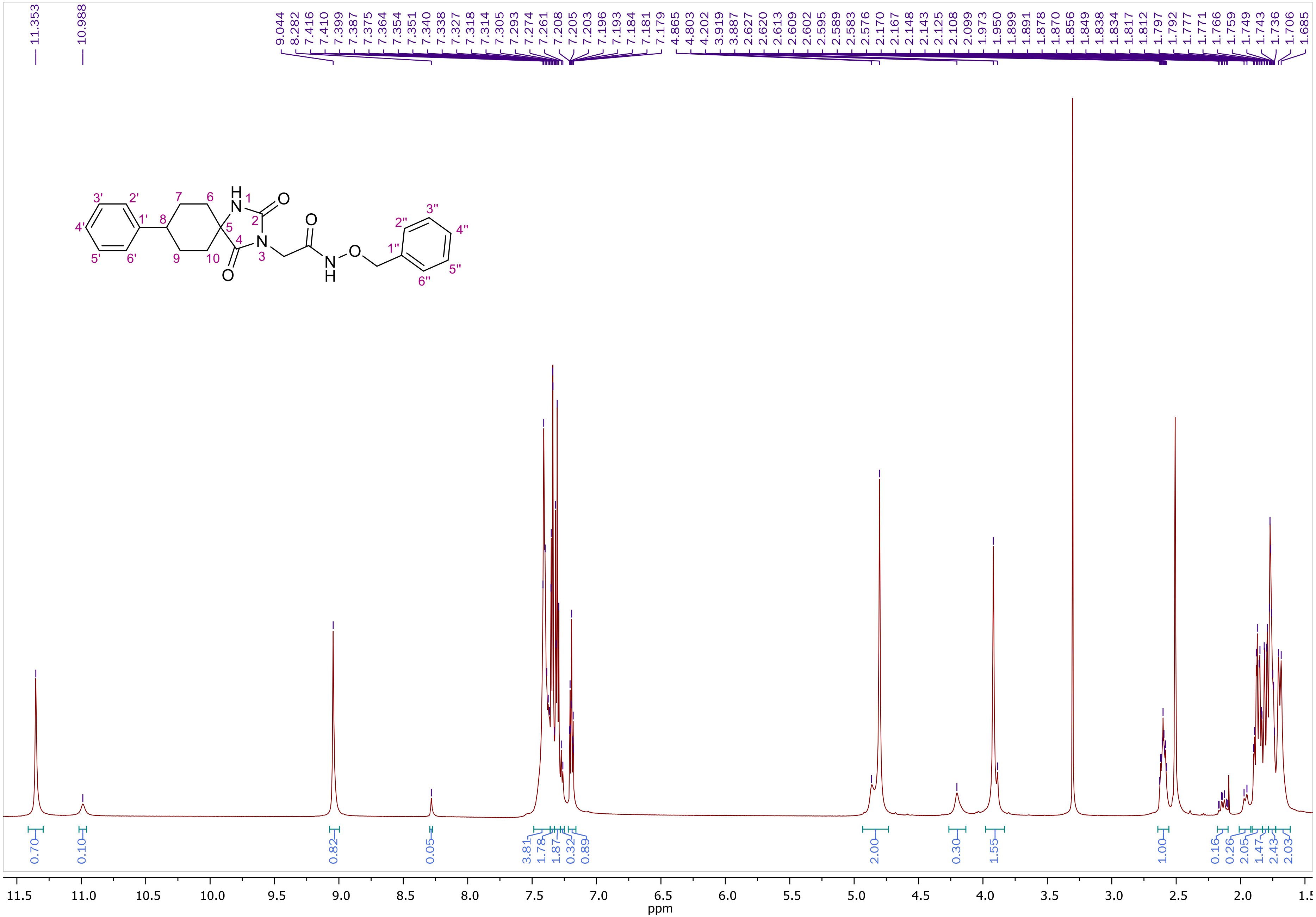

^{13}C NMR of **27** (50.32 MHz, DMSO-d_6)

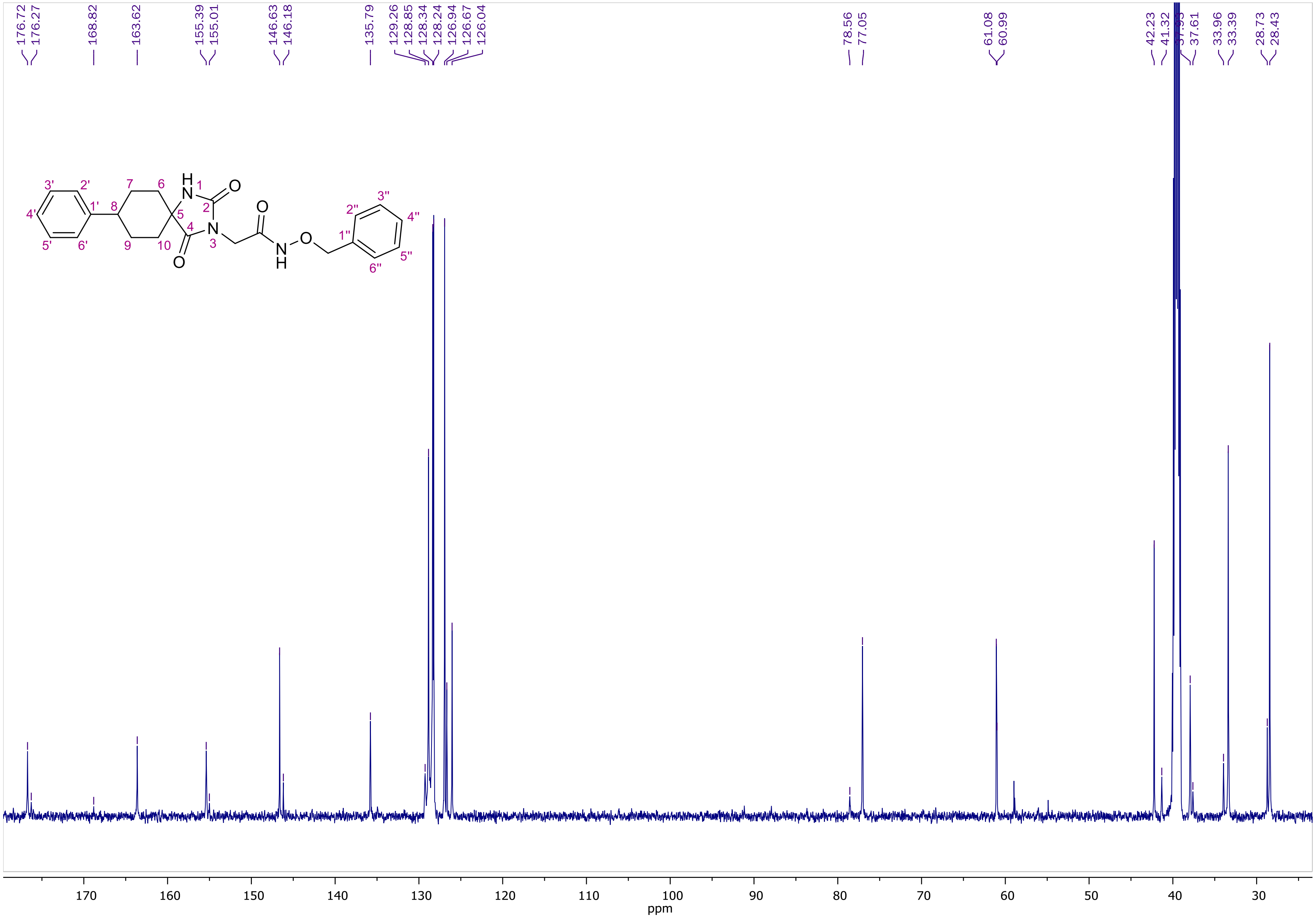

COSY NMR of **27** (400.13 MHz, DMSO-d_6)

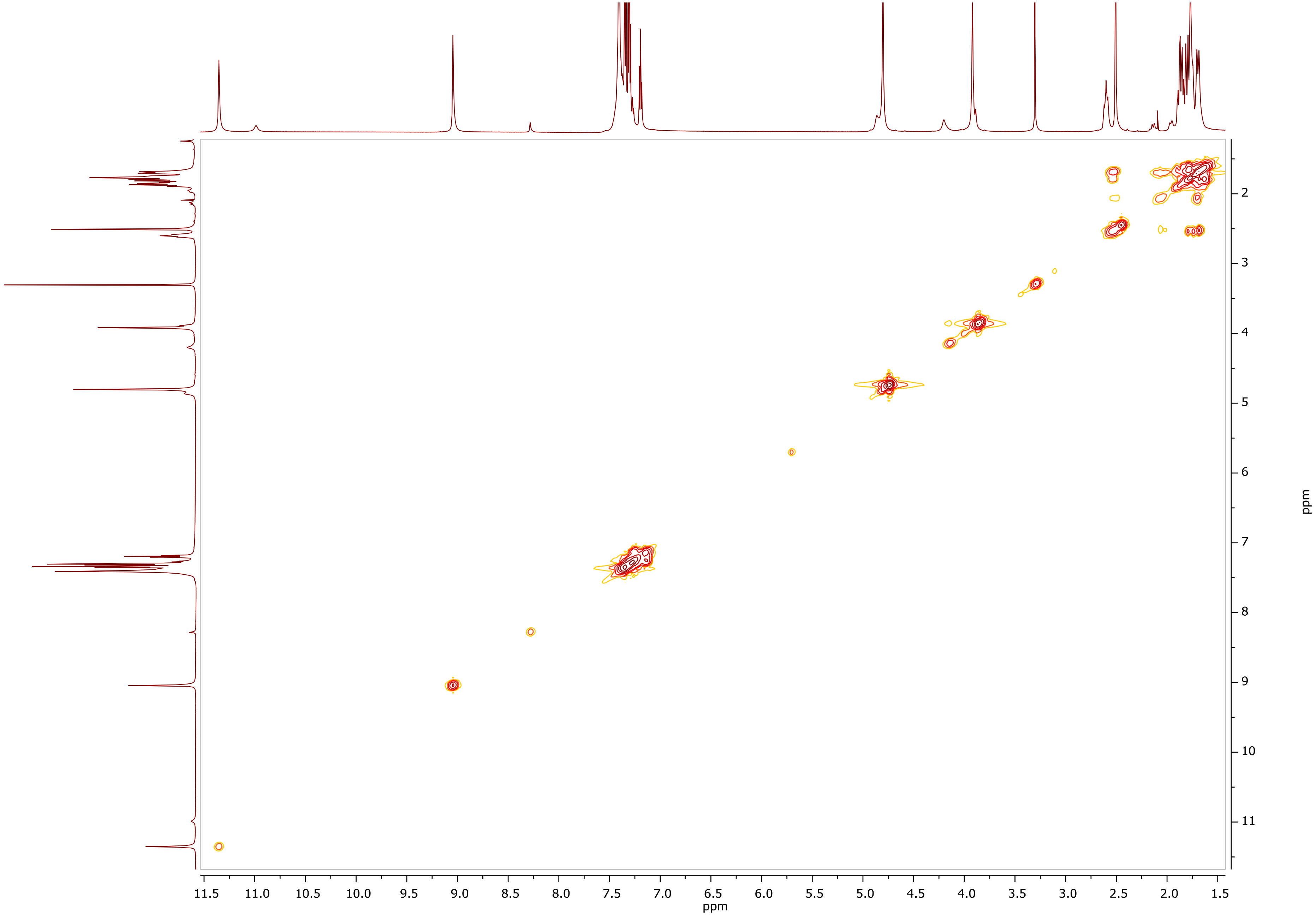

HSQC NMR of **27** (400.13 MHz, DMSO-d_6)

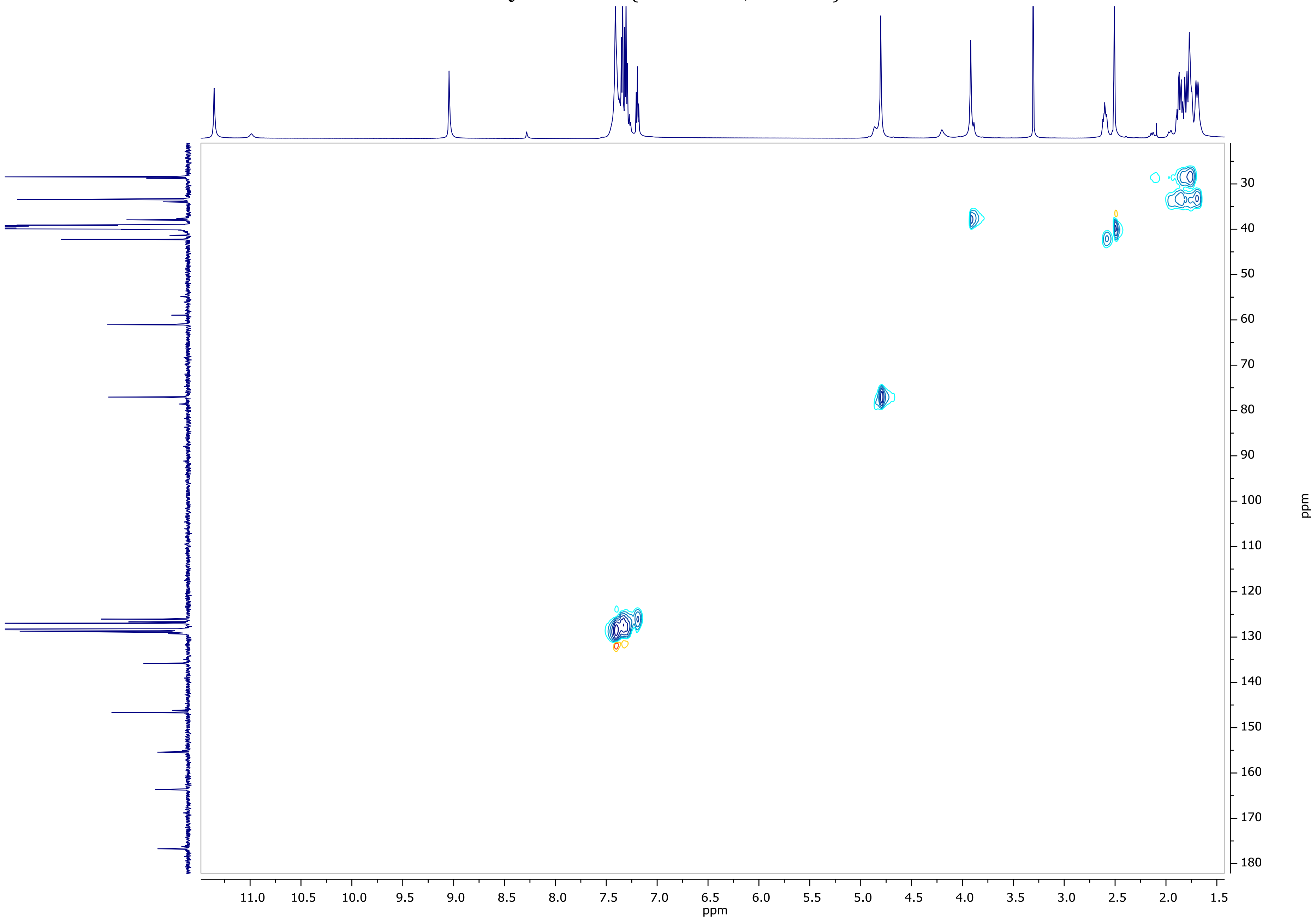

HMBC NMR of **27** (400.13 MHz, DMSO-d_6)

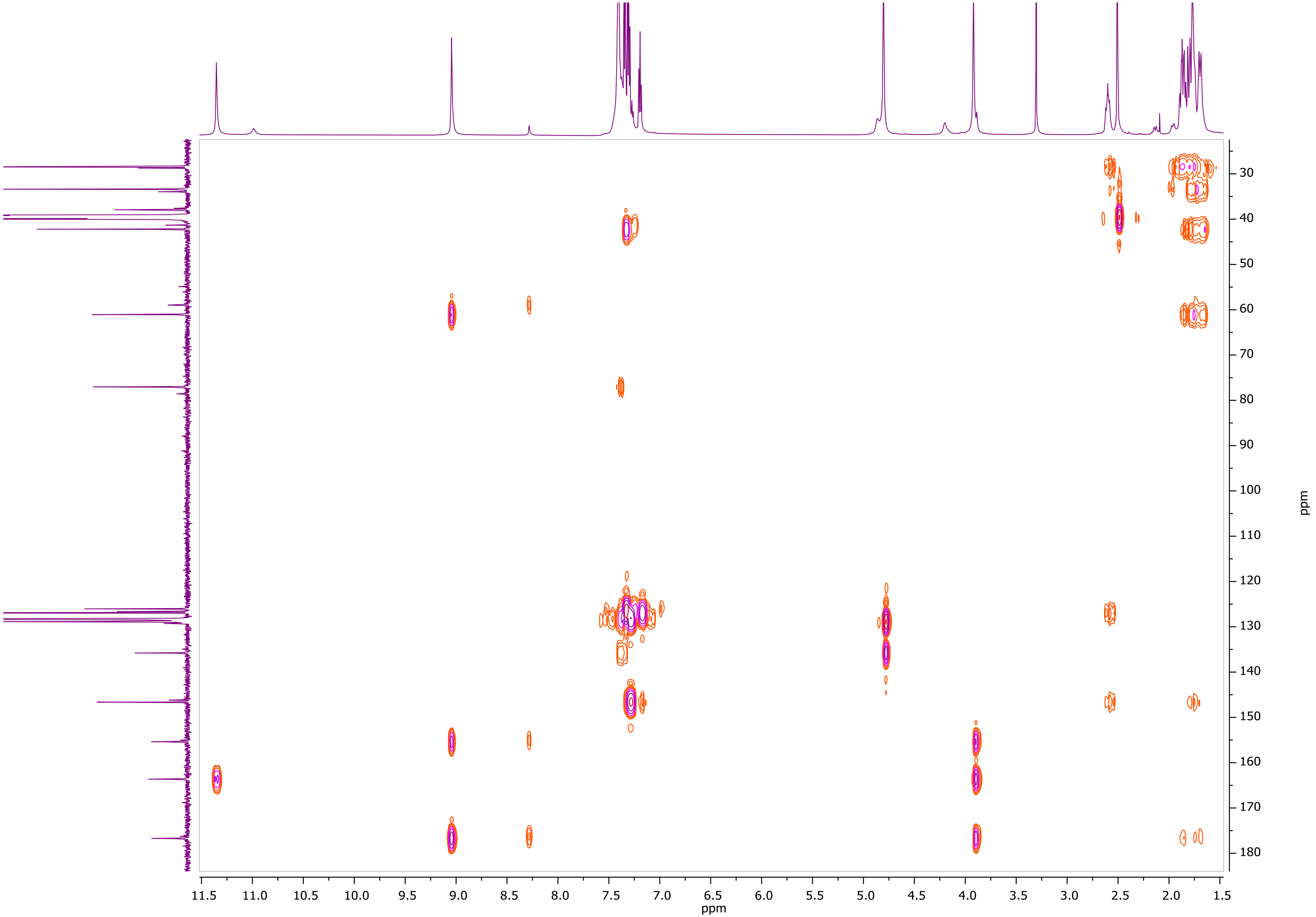

^{1}H NMR of **28** (600.11 MHz, DMSO-d_6)

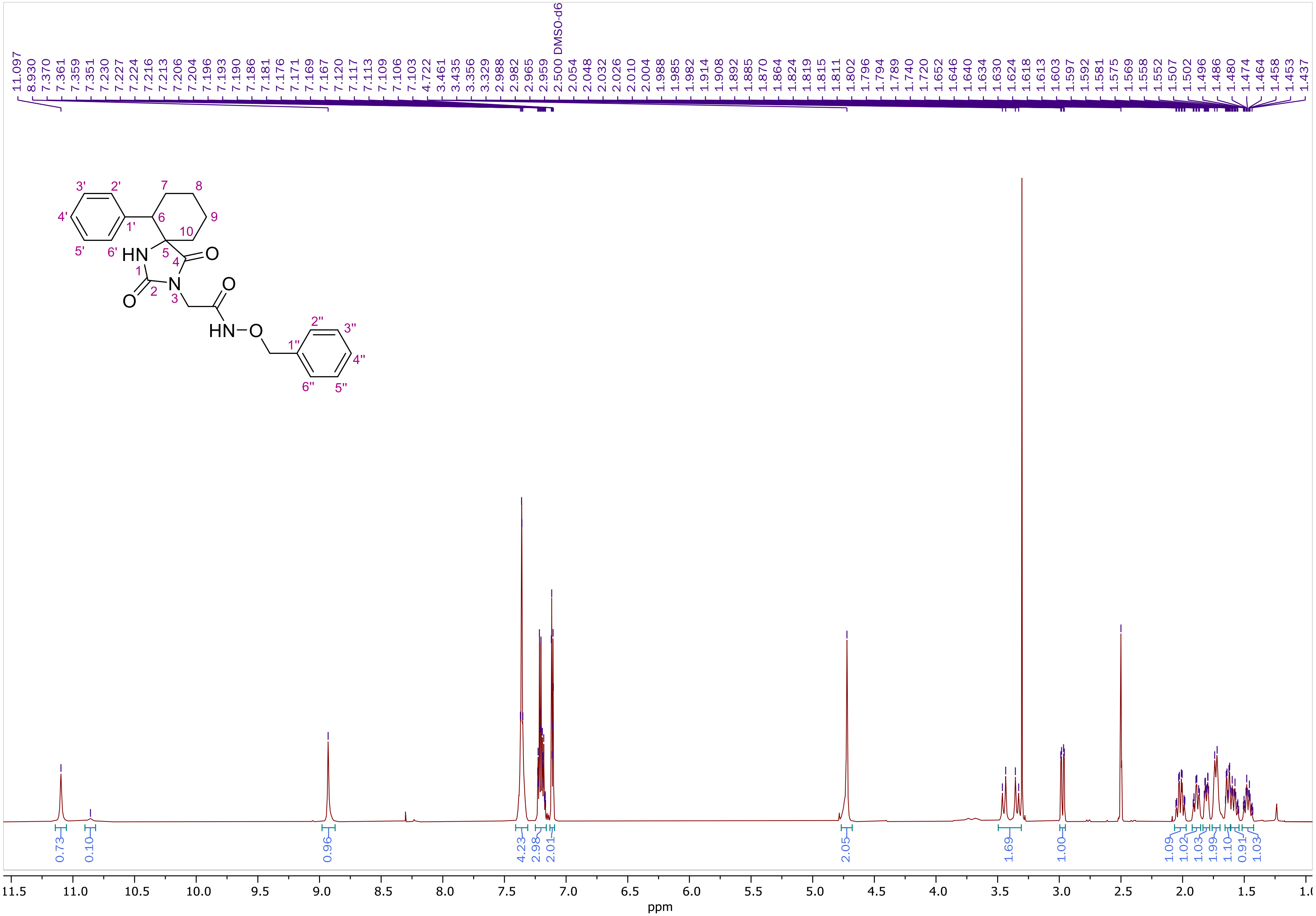

^{13}C NMR of **28** (100.61 MHz, DMSO-d_6)

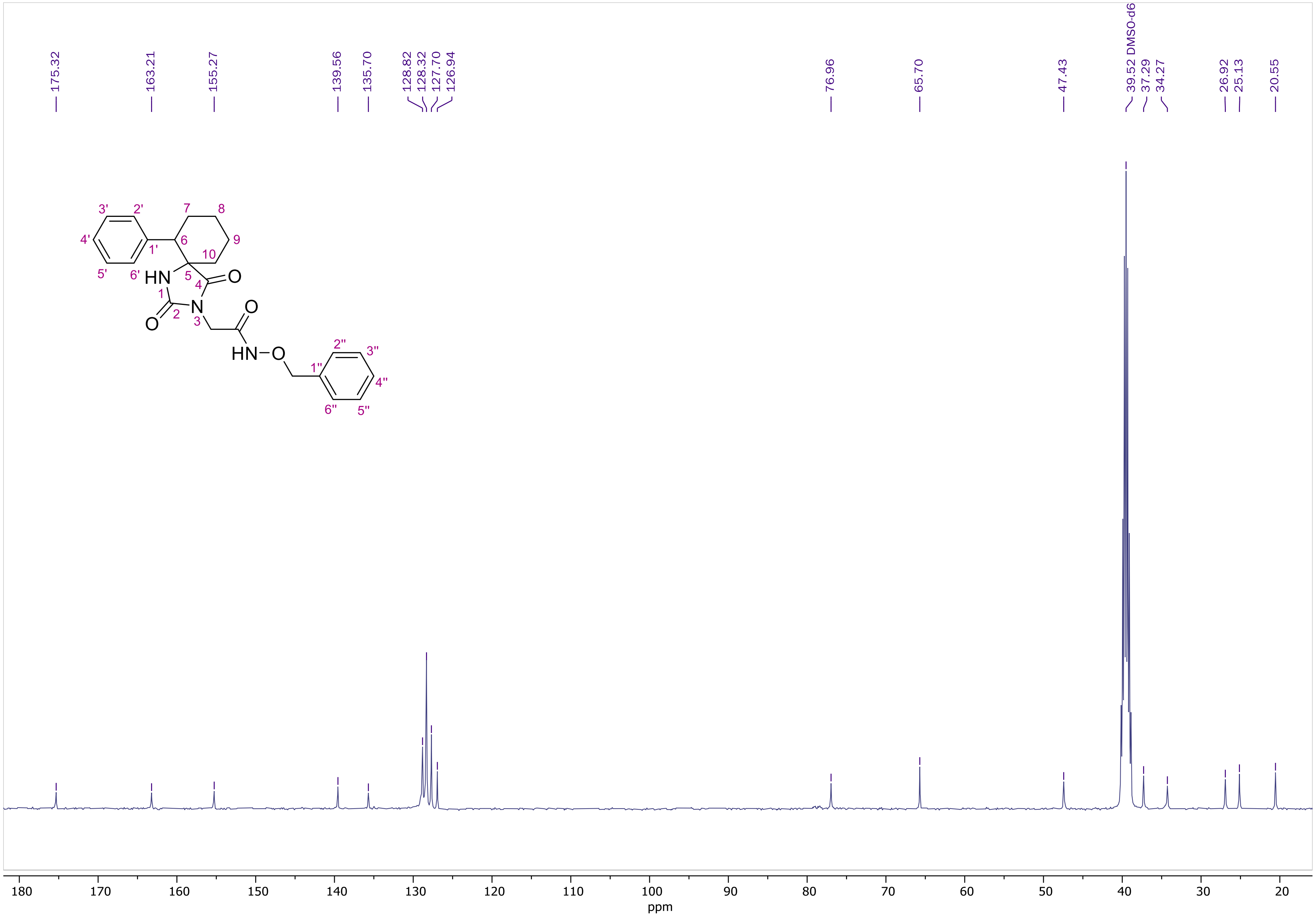

COSY NMR of **28** (400.13 MHz, DMSO-d_6)

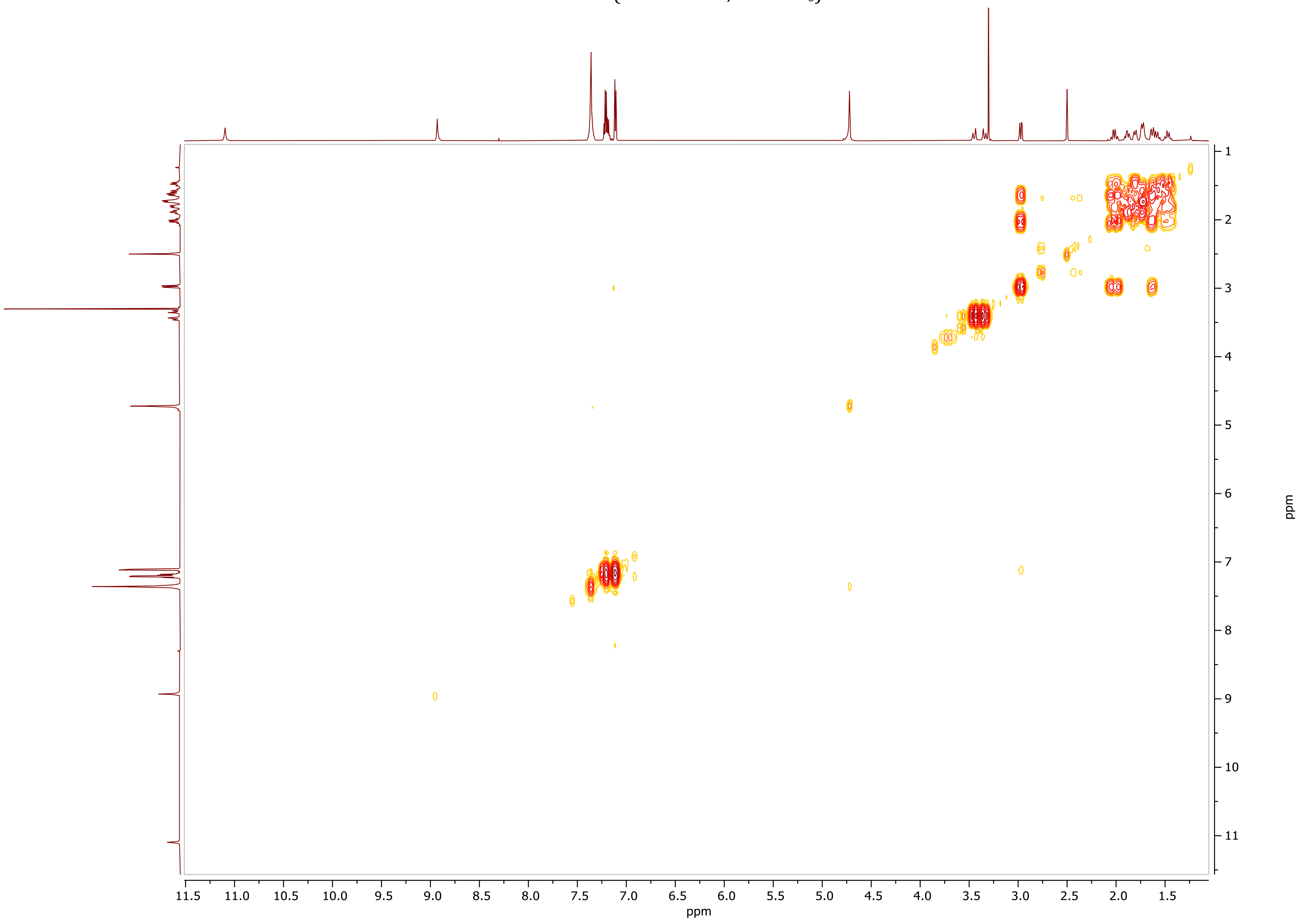

HSQC-DEPT NMR of **28** (600.11 MHz, DMSO-d_6)

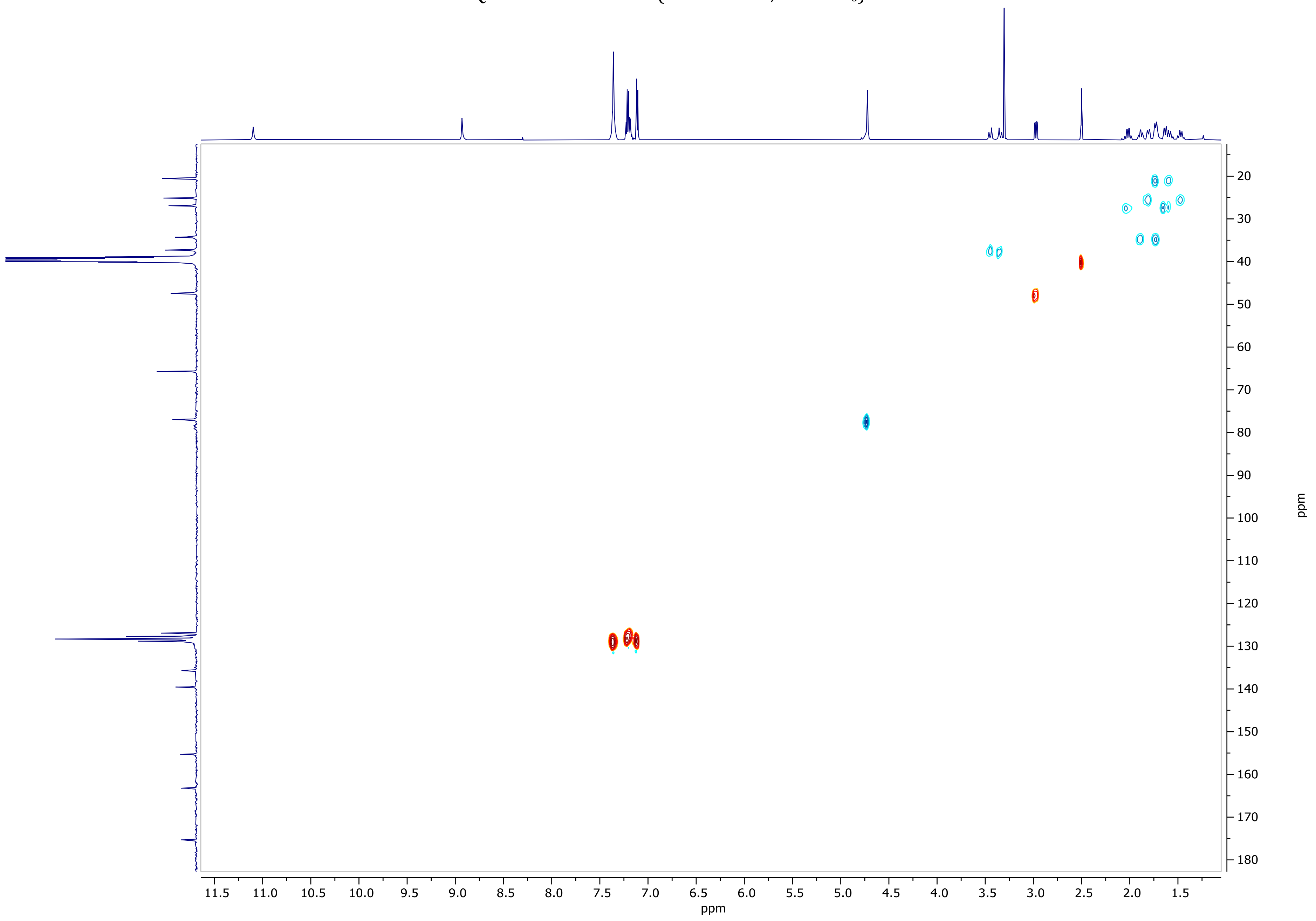

HMBC NMR of **28** (400.13 MHz, DMSO-d_6)

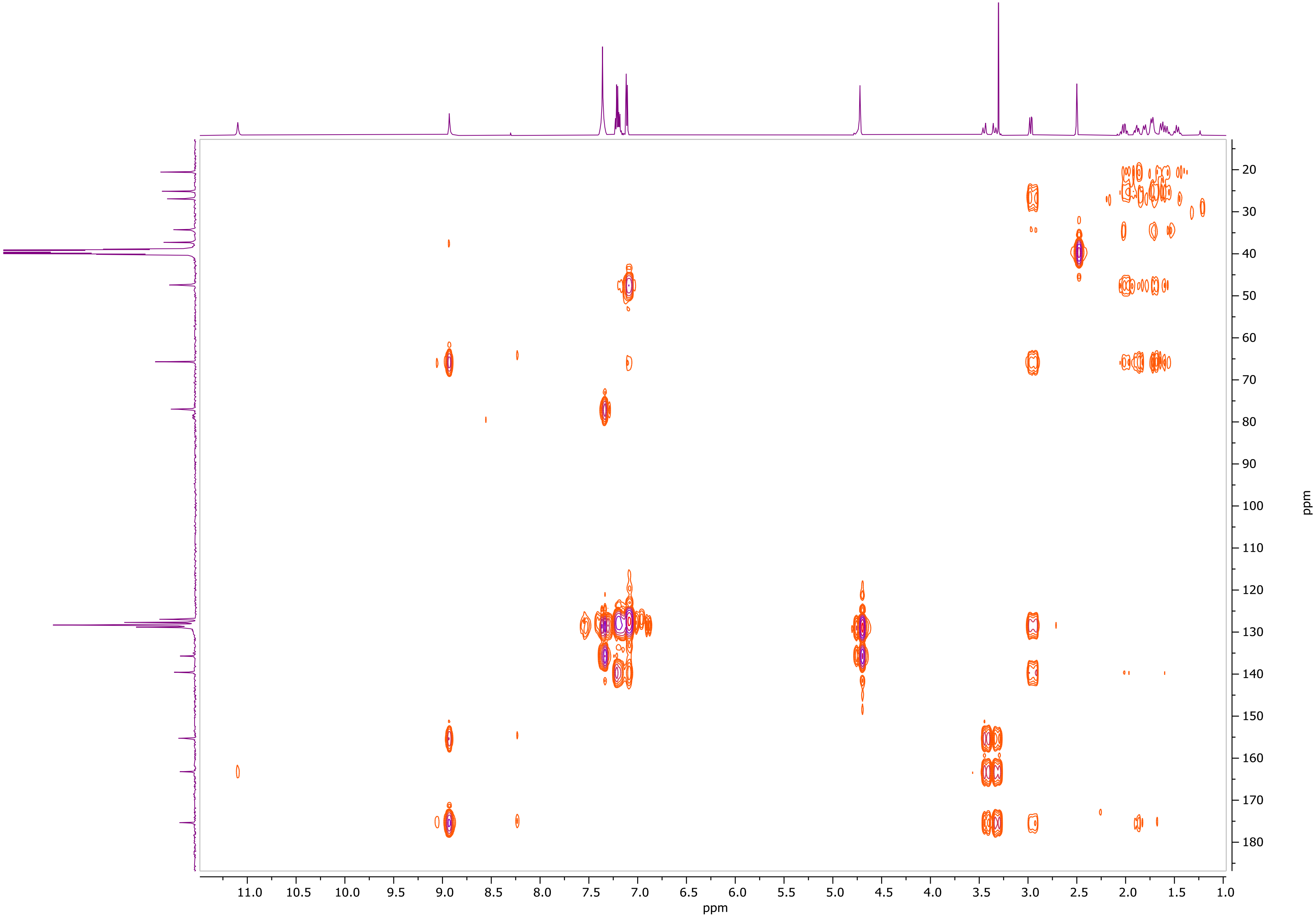

¹H NMR of **29** (400.13 MHz, $CDCl_3$)

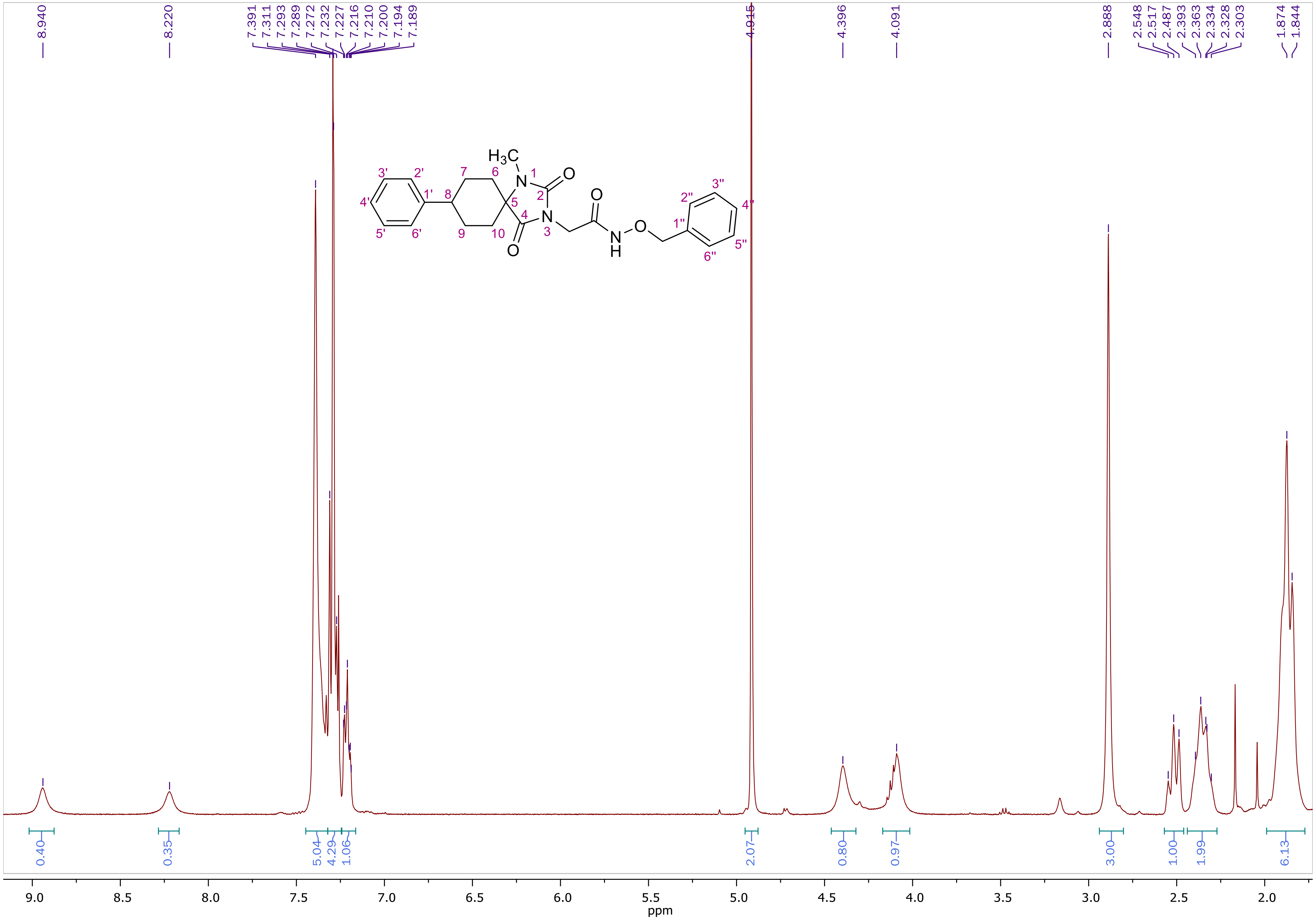

^{13}C NMR of **29** (50.32 MHz, $CDCl_3$)

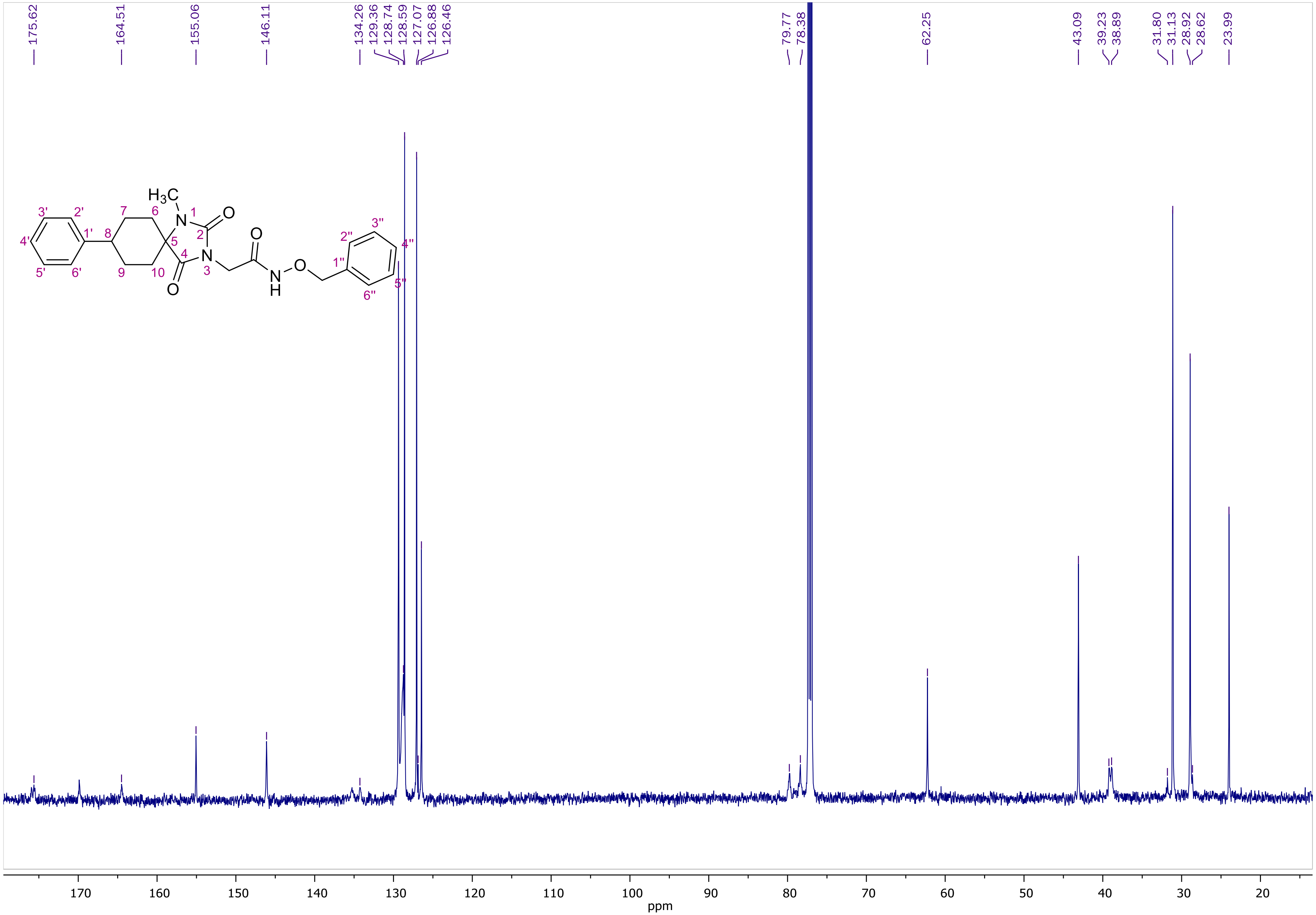

COSY NMR of **29** (400.13 MHz, $CDCl_3$)

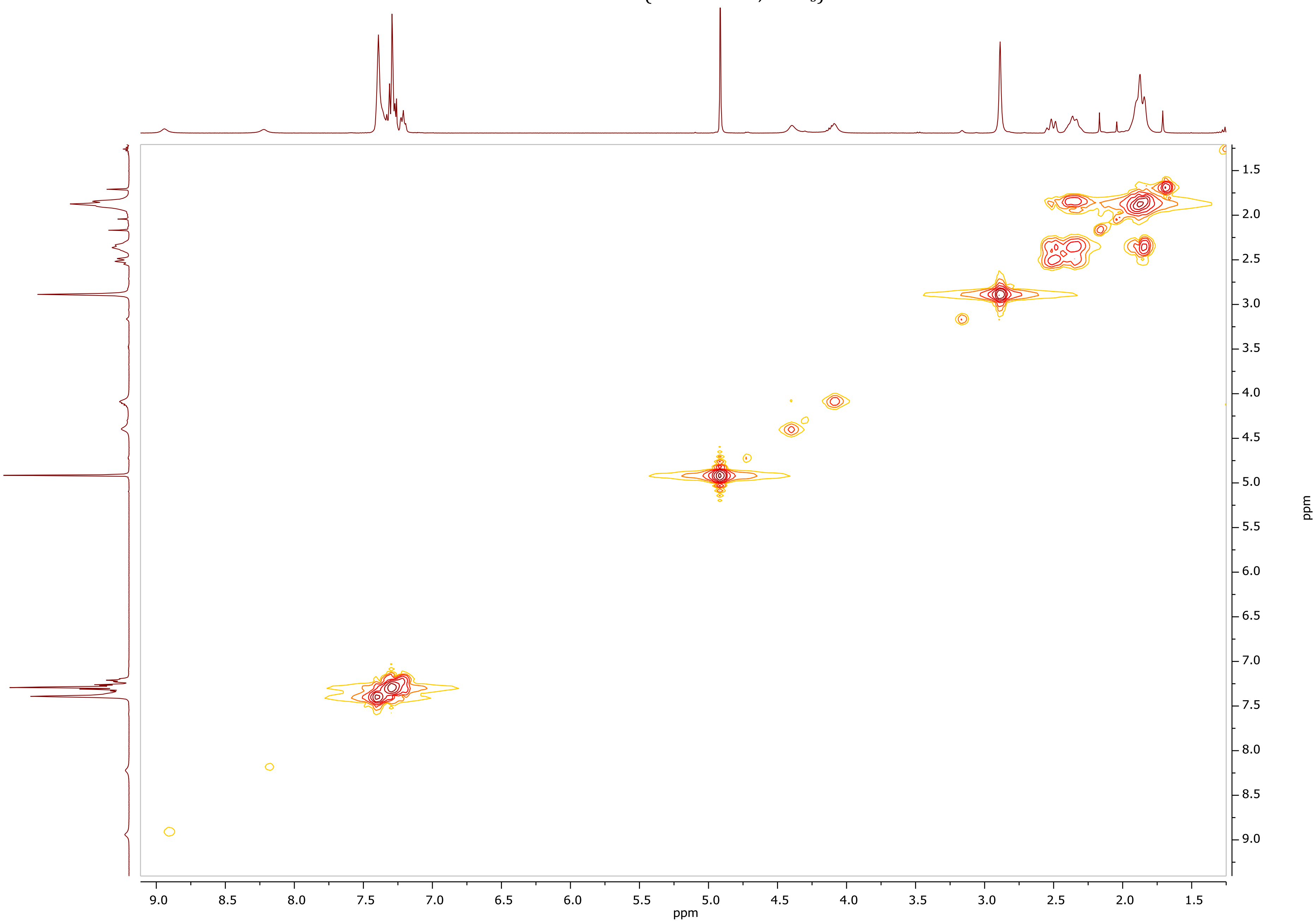

HSQC NMR of **29** (400.13 MHz, $CDCl_3$)

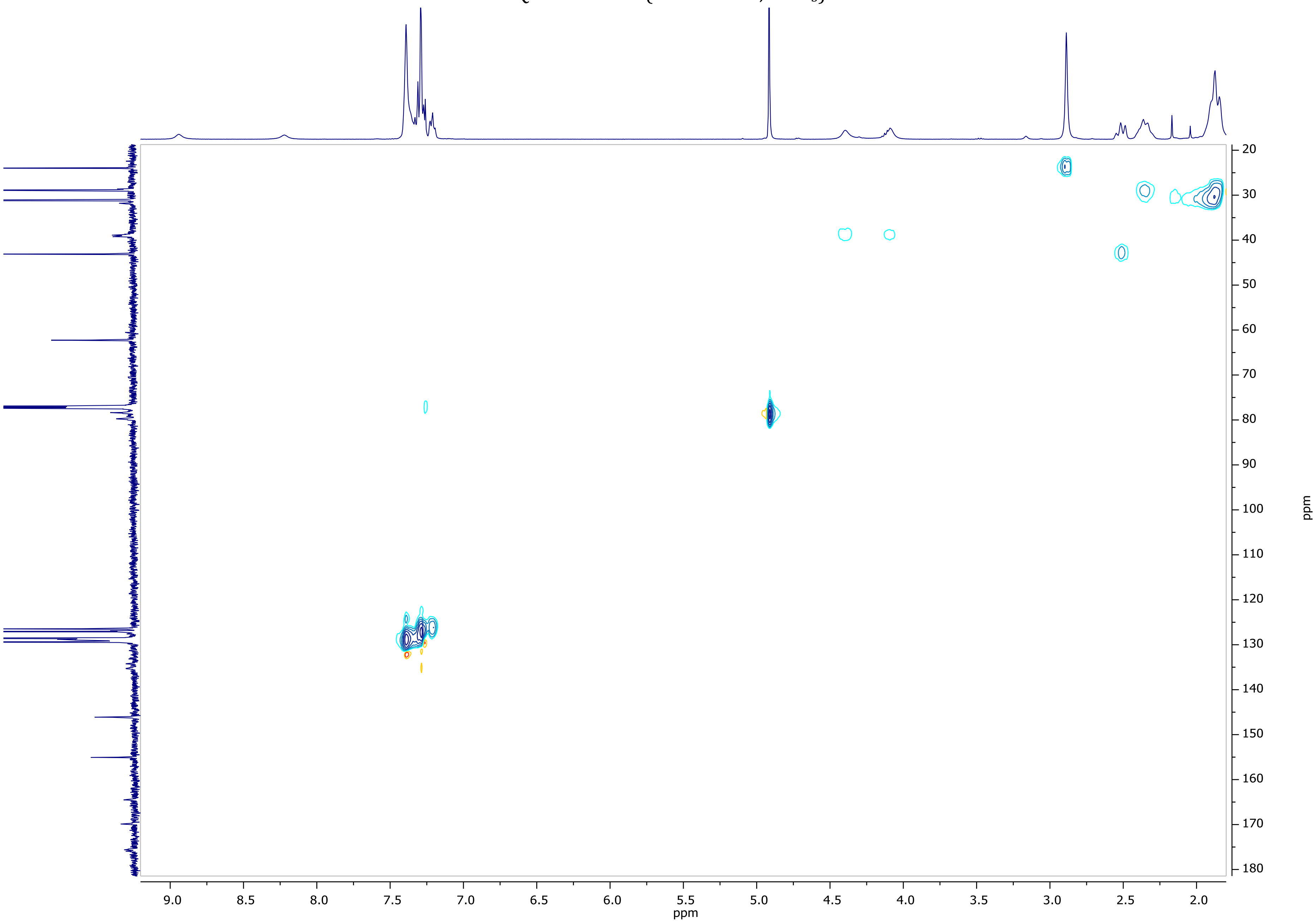

HMBC NMR of **29** (400.13 MHz, $CDCl_3$)

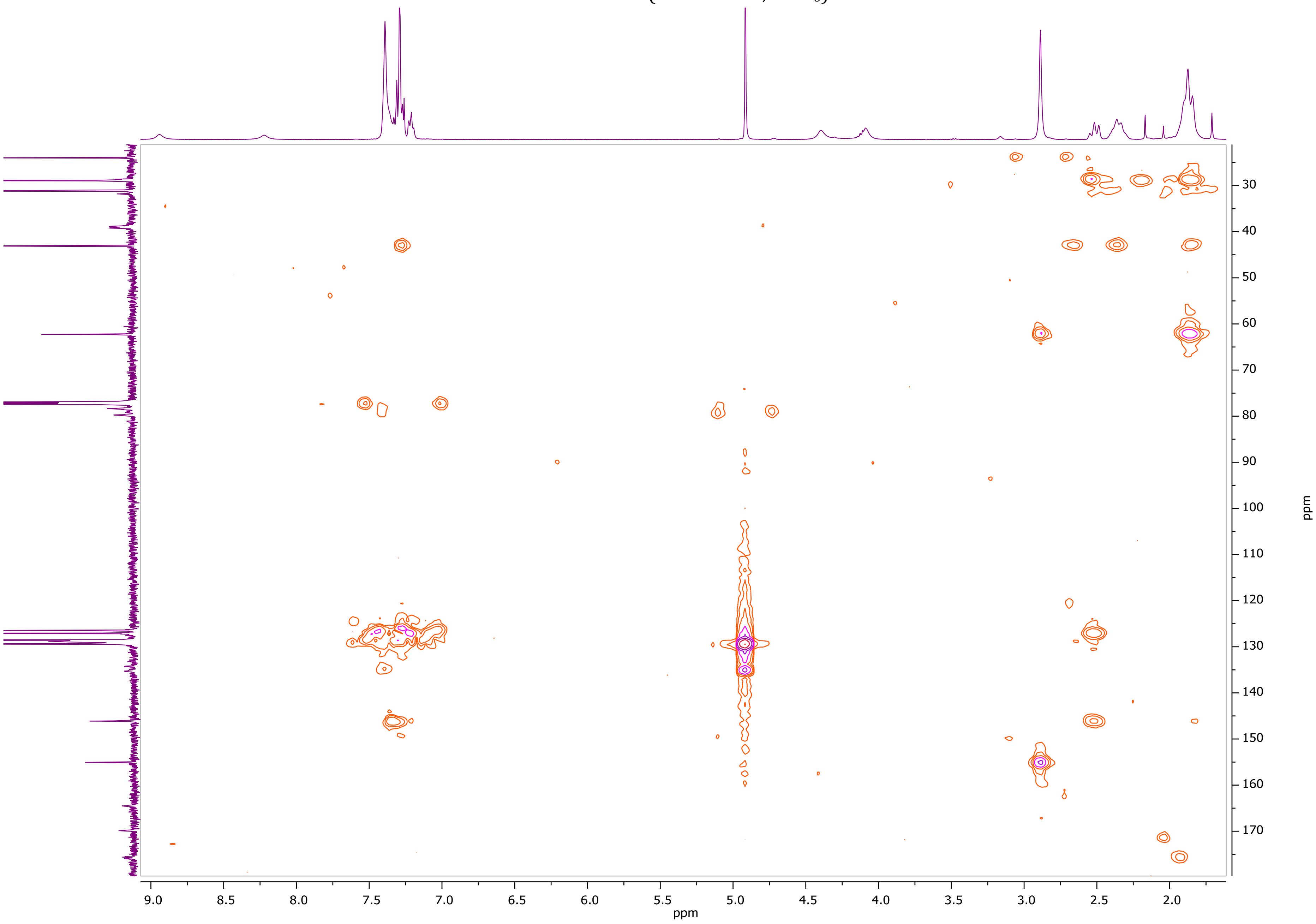

^{1}H NMR of **30** (600.11 MHz, DMSO-d_6)

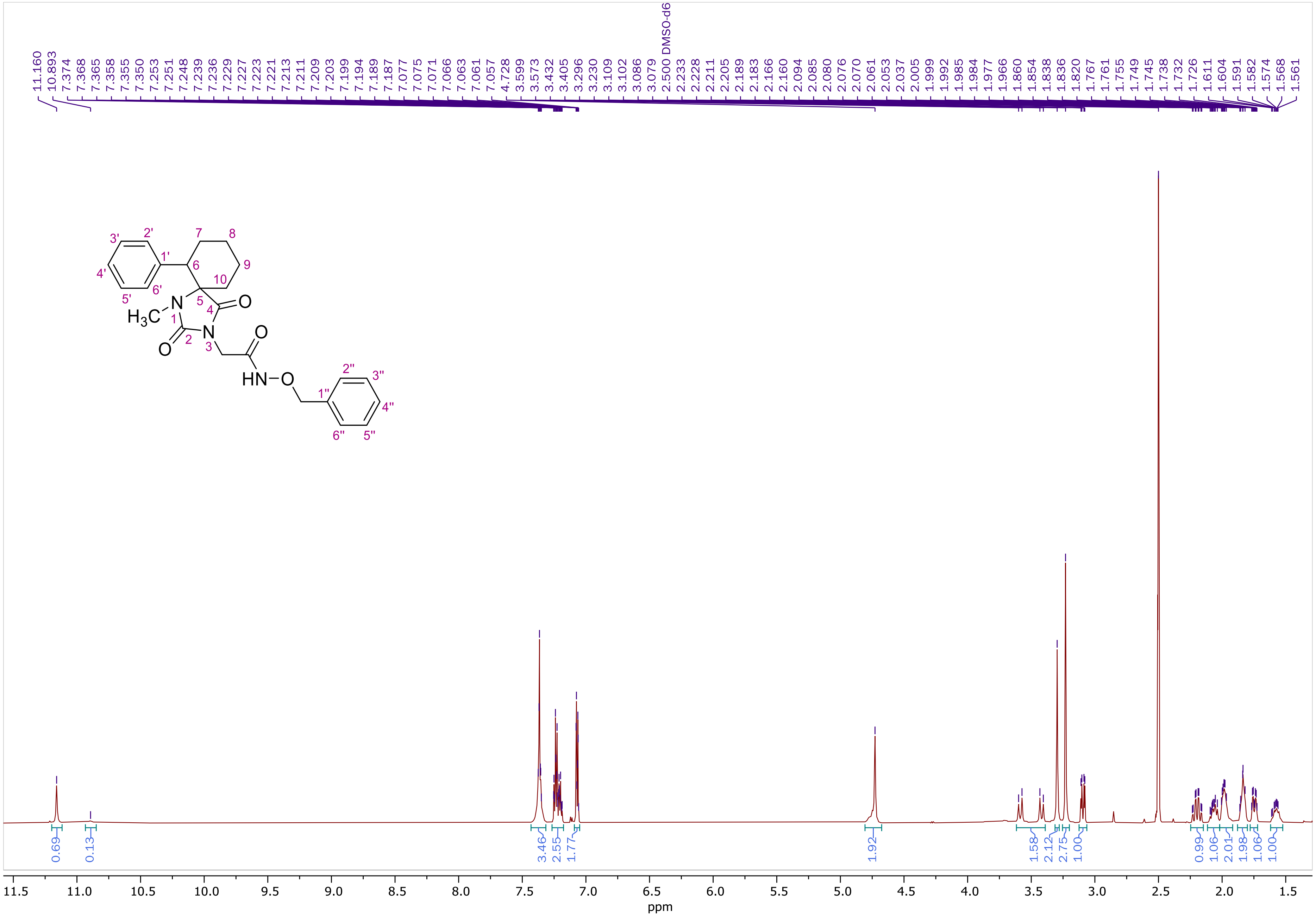

^{13}C NMR of **30** (100.61 MHz, DMSO-d_6)

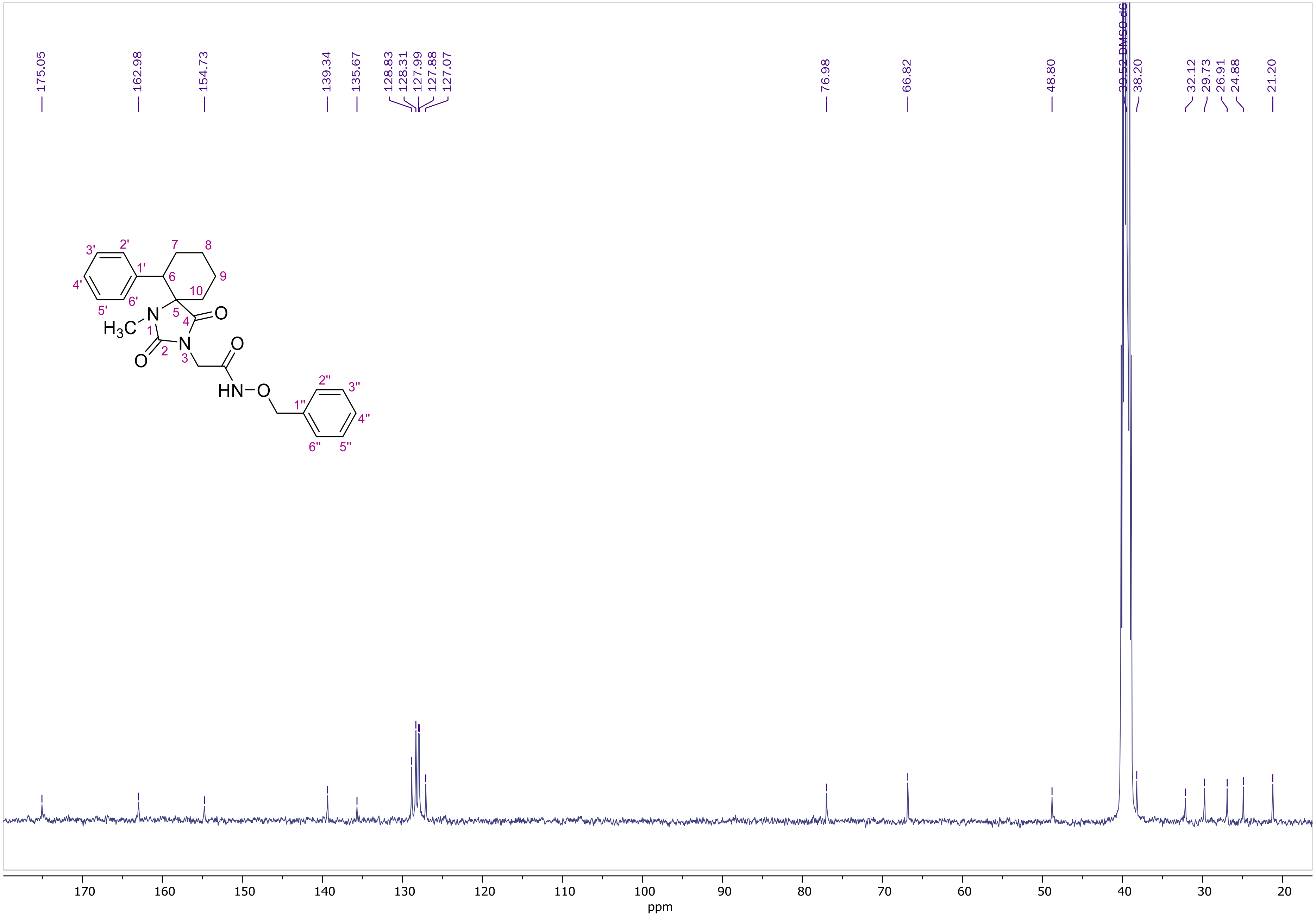

COSY NMR of **30** (400.13 MHz, DMSO-d_6)

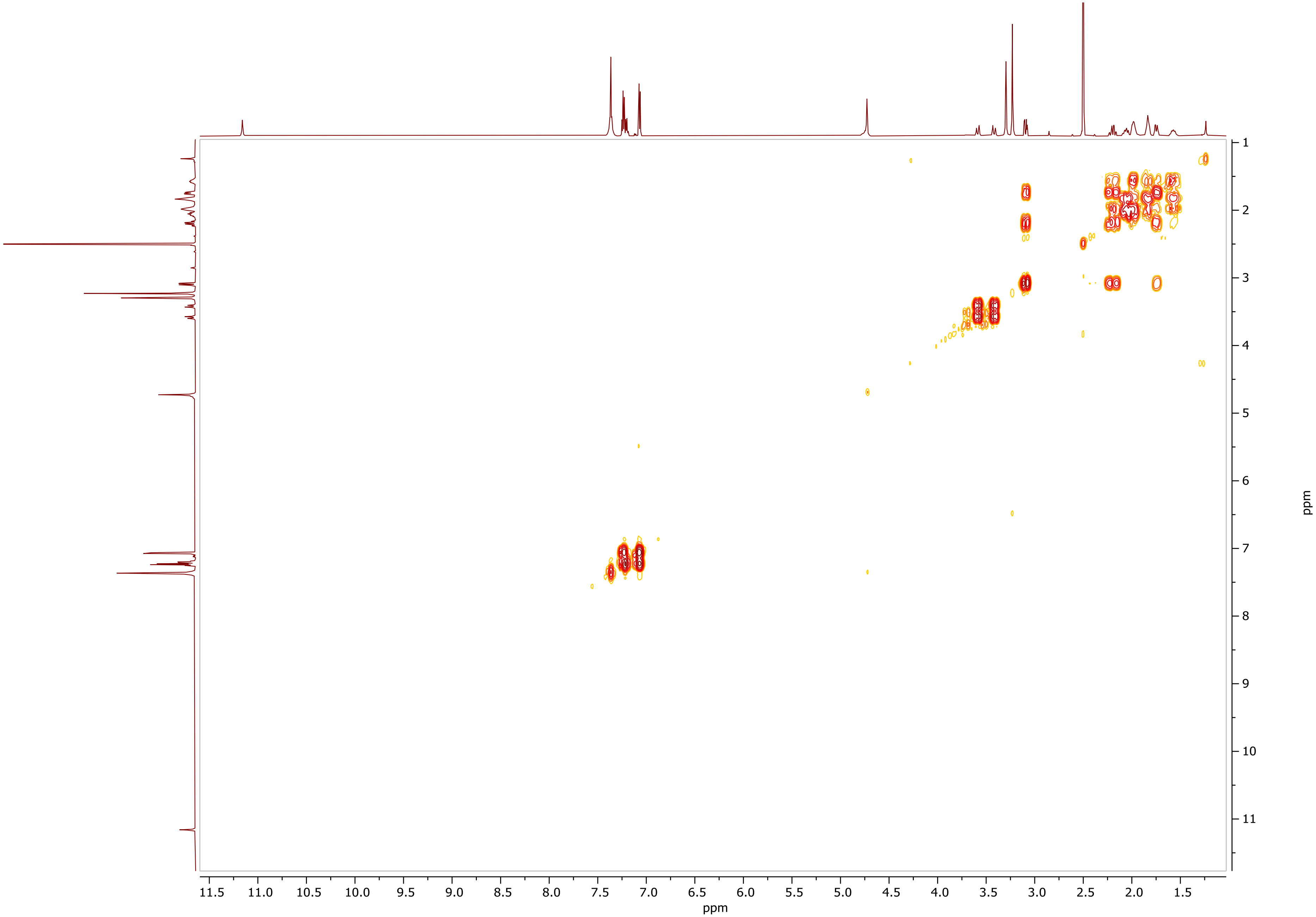

HSQC-DEPT NMR of **30** (600.11 MHz, DMSO-d_6)

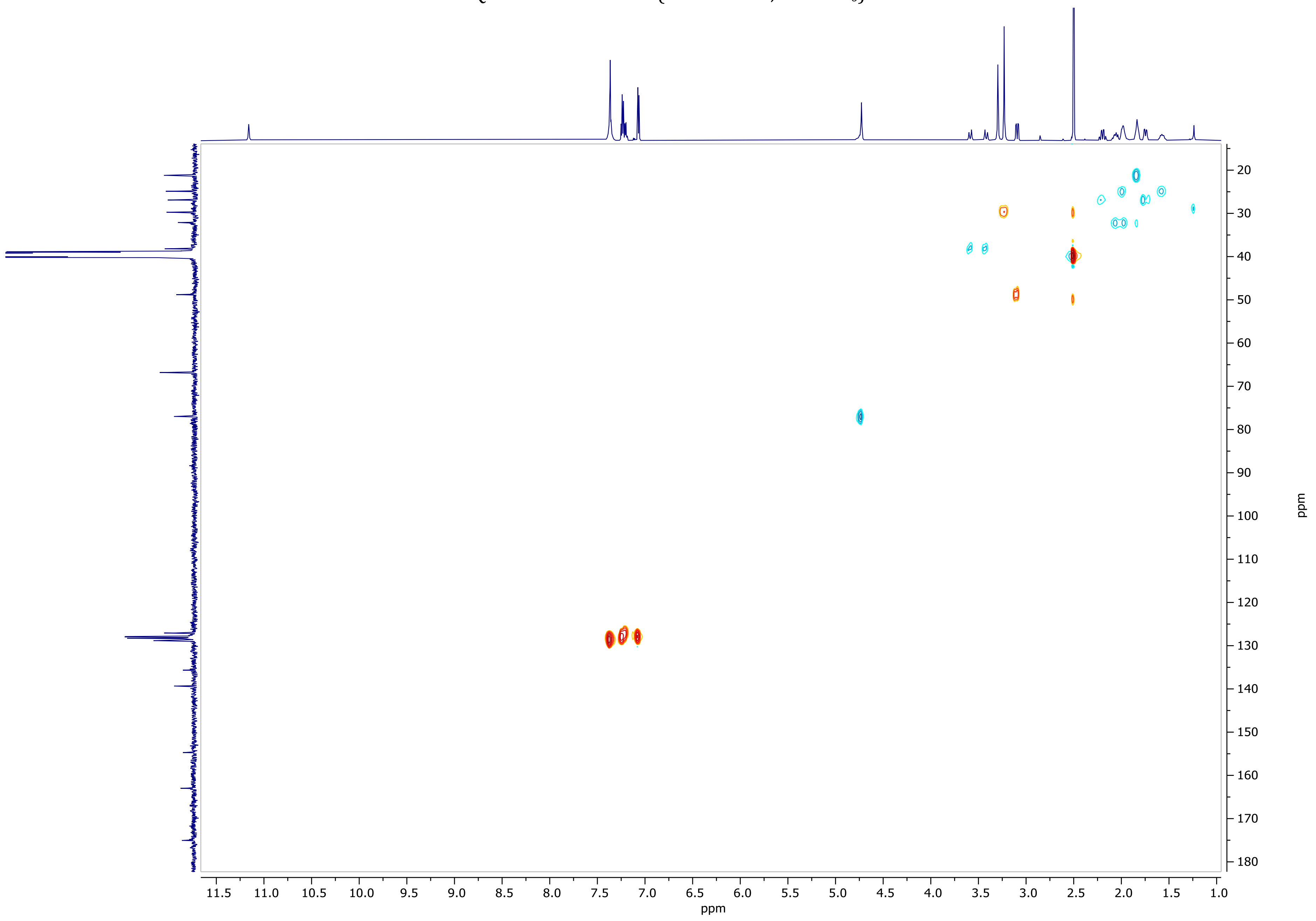

HMBC NMR of **30** (400.13 MHz, DMSO-d_6)

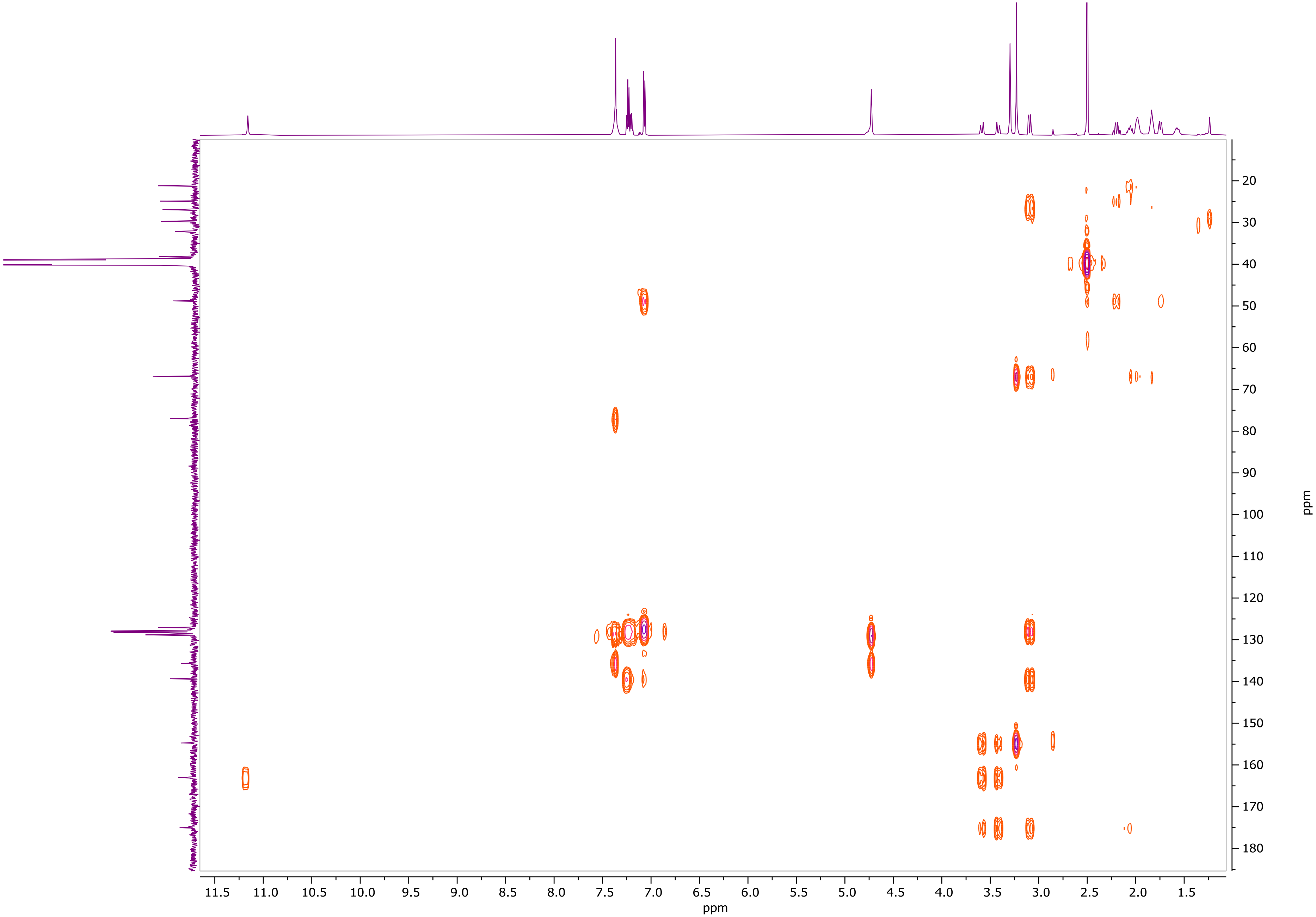

^{1}H NMR of **31** (400.13 MHz, DMSO-d_6)

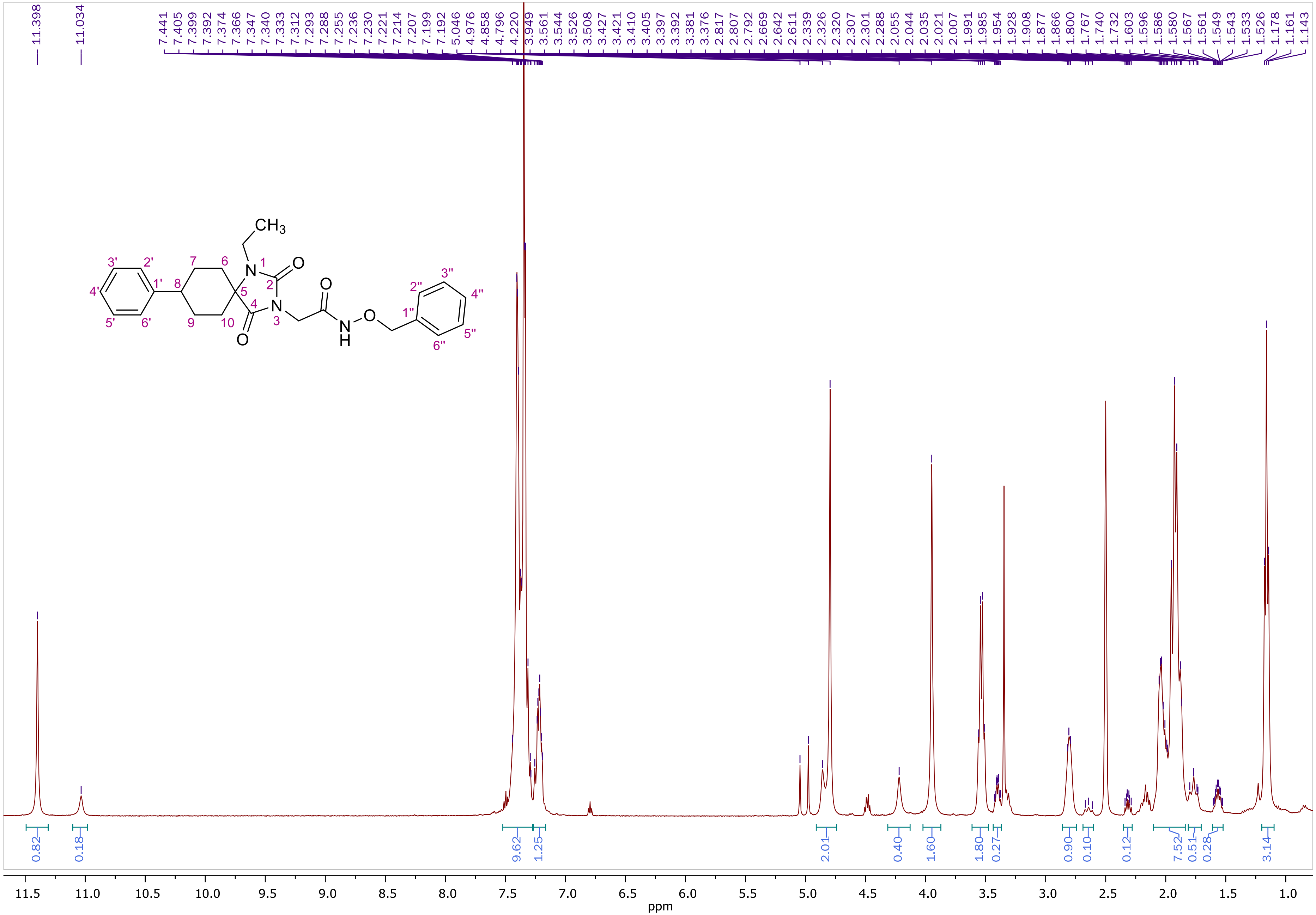

^{13}C NMR of **31** (100.61 MHz, DMSO-d_6)

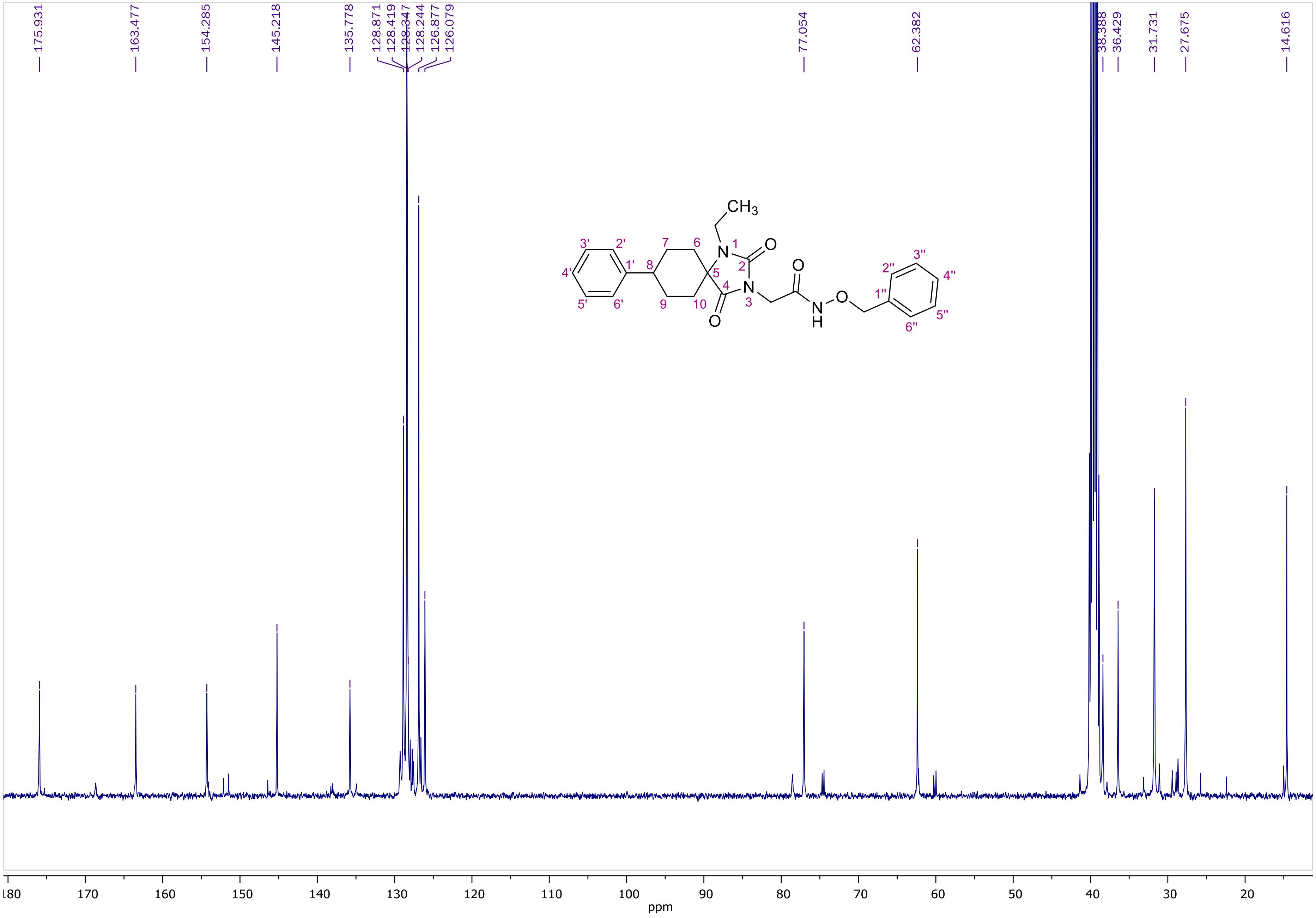

COSY NMR of **31** (400.13 MHz, DMSO-d_6)

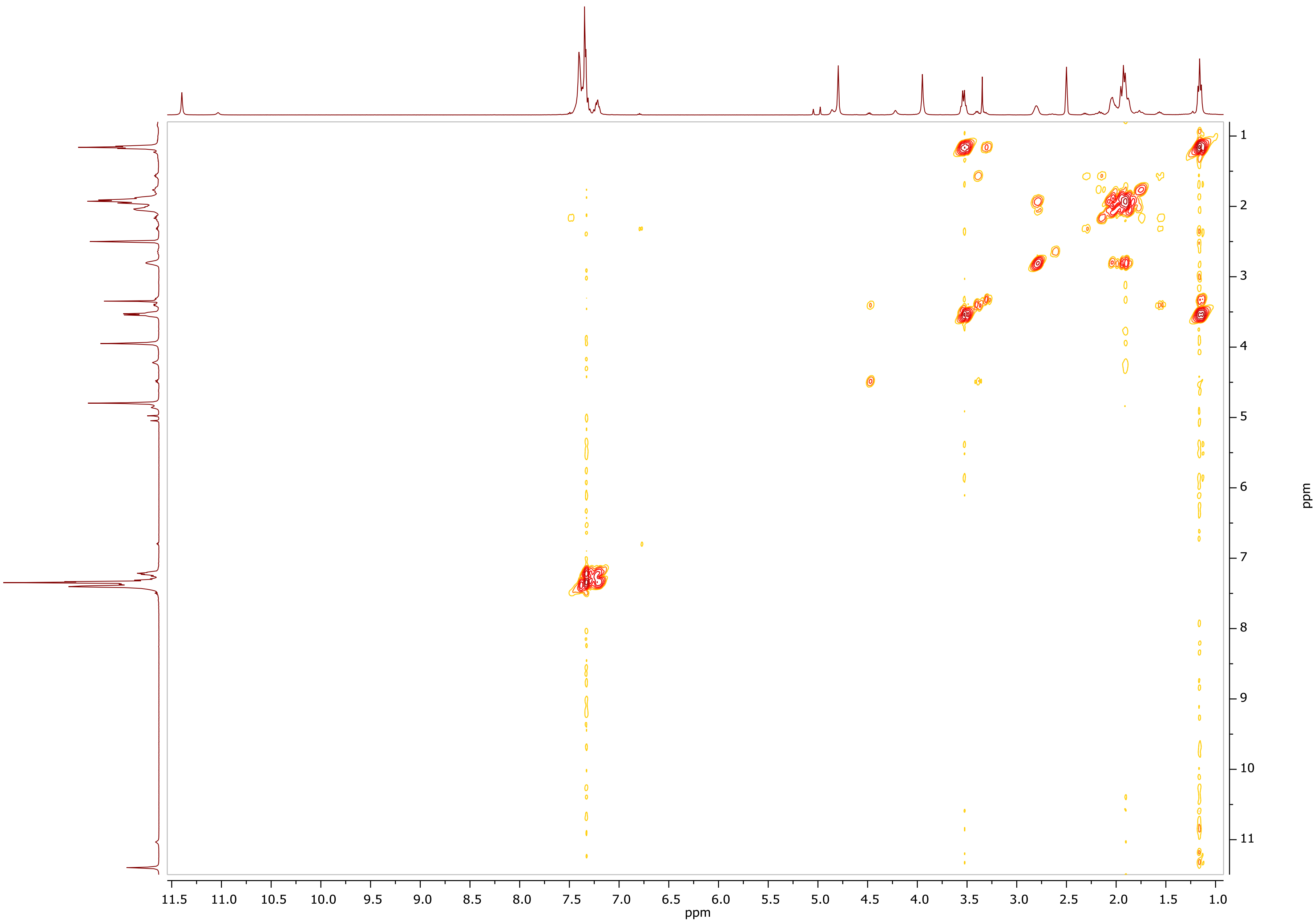

HSQC-DEPT NMR of **31** (400.13 MHz, DMSO-d_6)

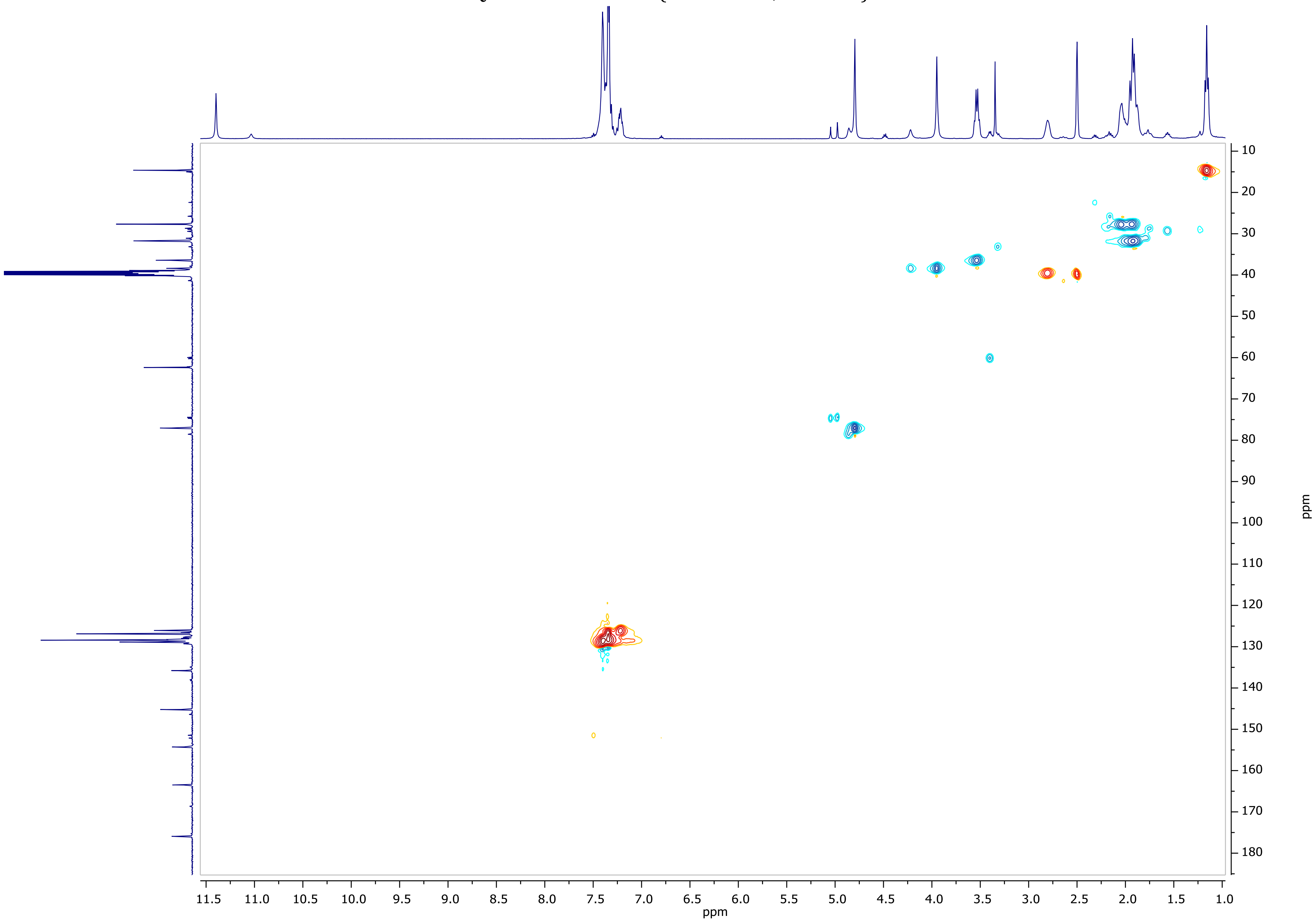

HMBC NMR of **31** (400.13 MHz, DMSO-d_6)

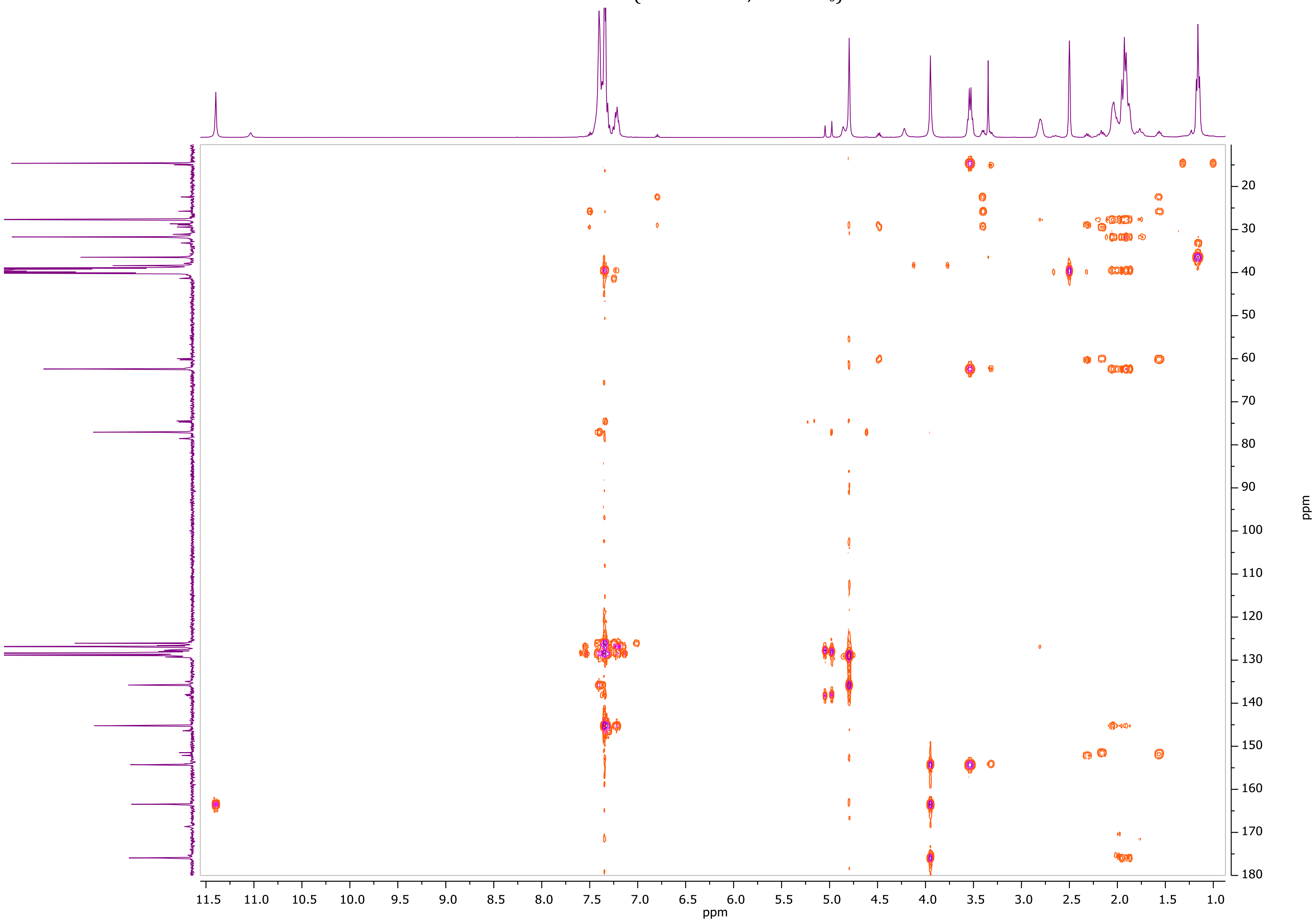

^{1}H NMR of **32** (600.11 MHz, DMSO-d_6)

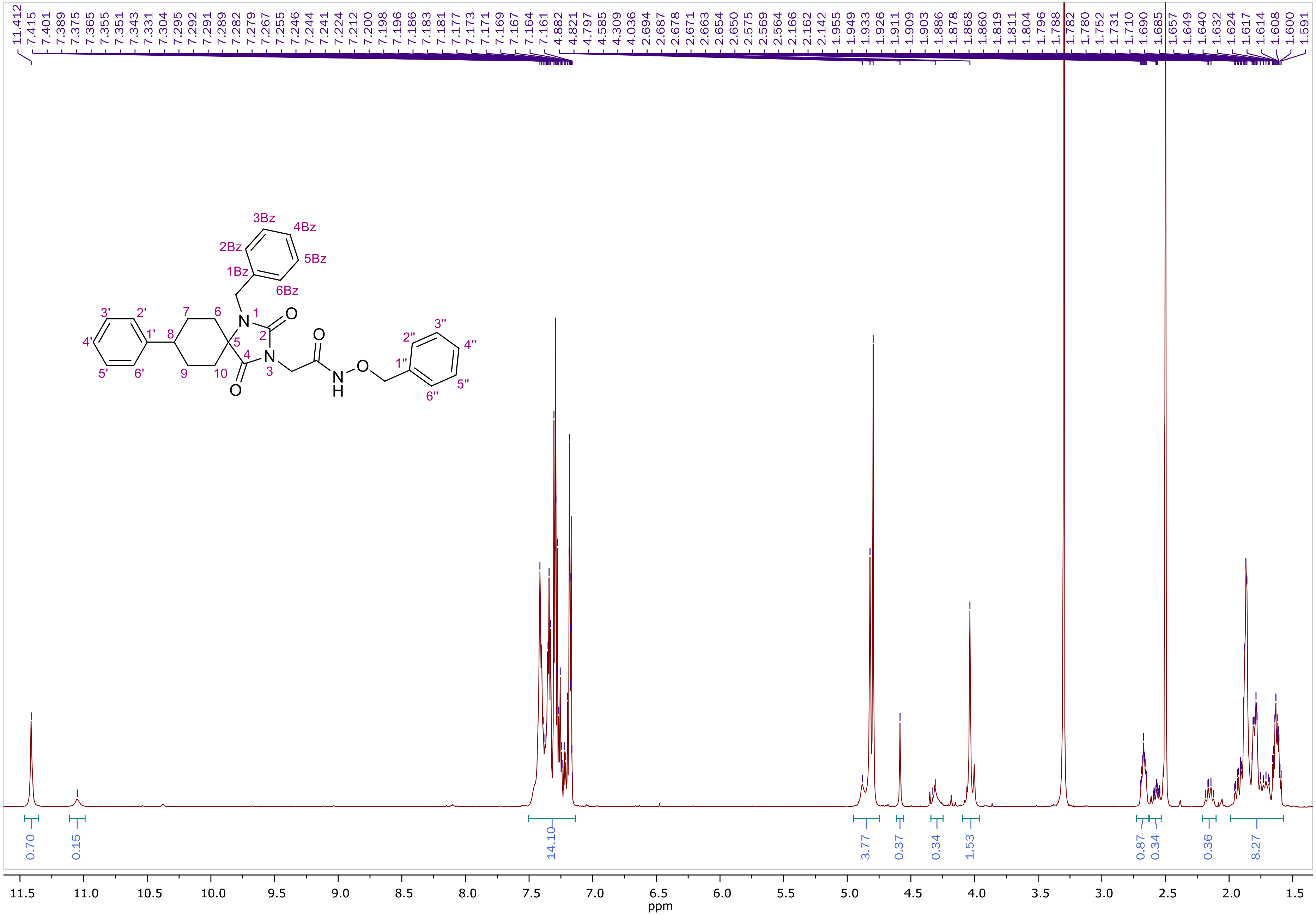

^{13}C NMR of **32** (100.61 MHz, DMSO-d_6)

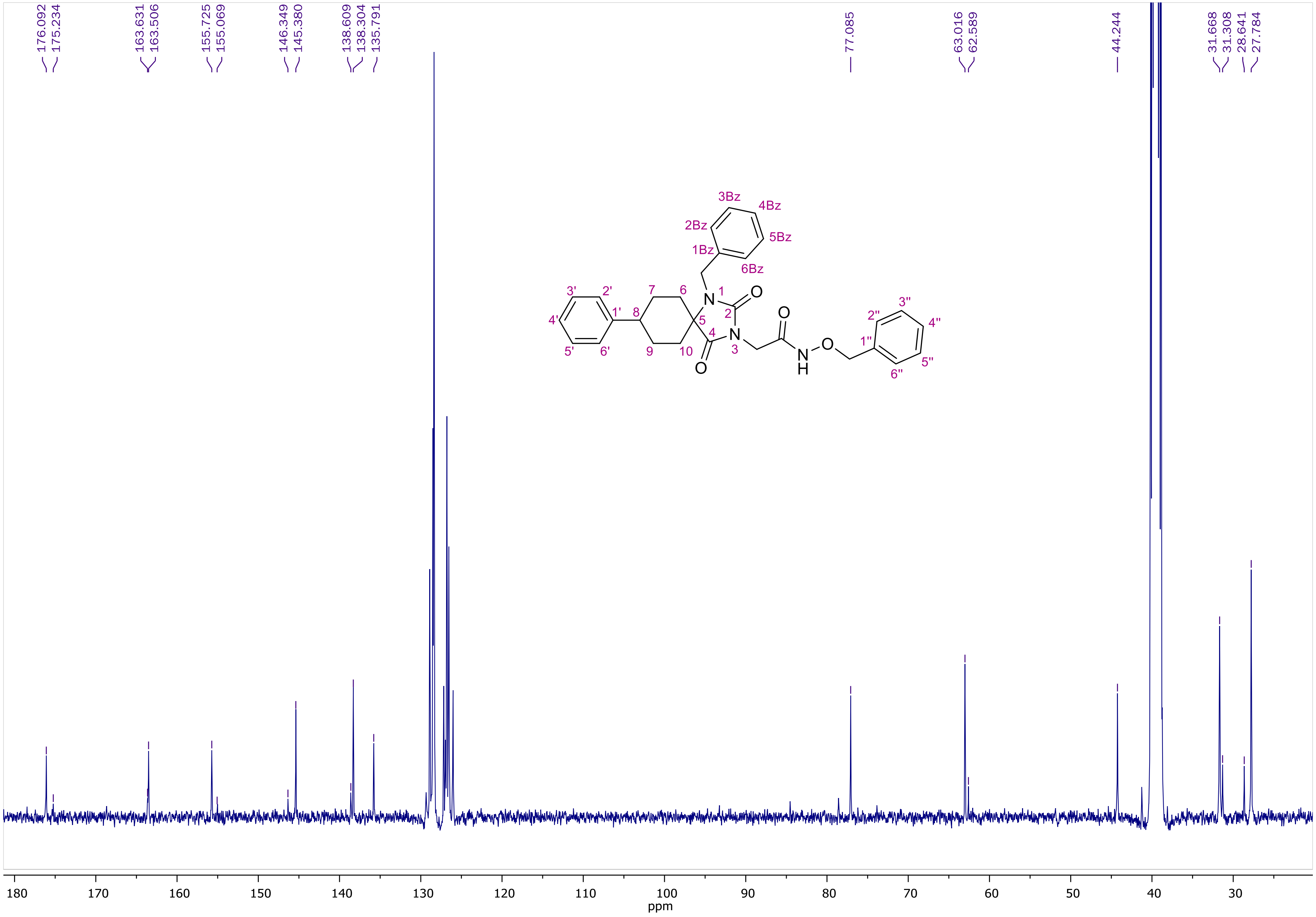

COSY NMR of **32** (400.13 MHz, DMSO-d_6)

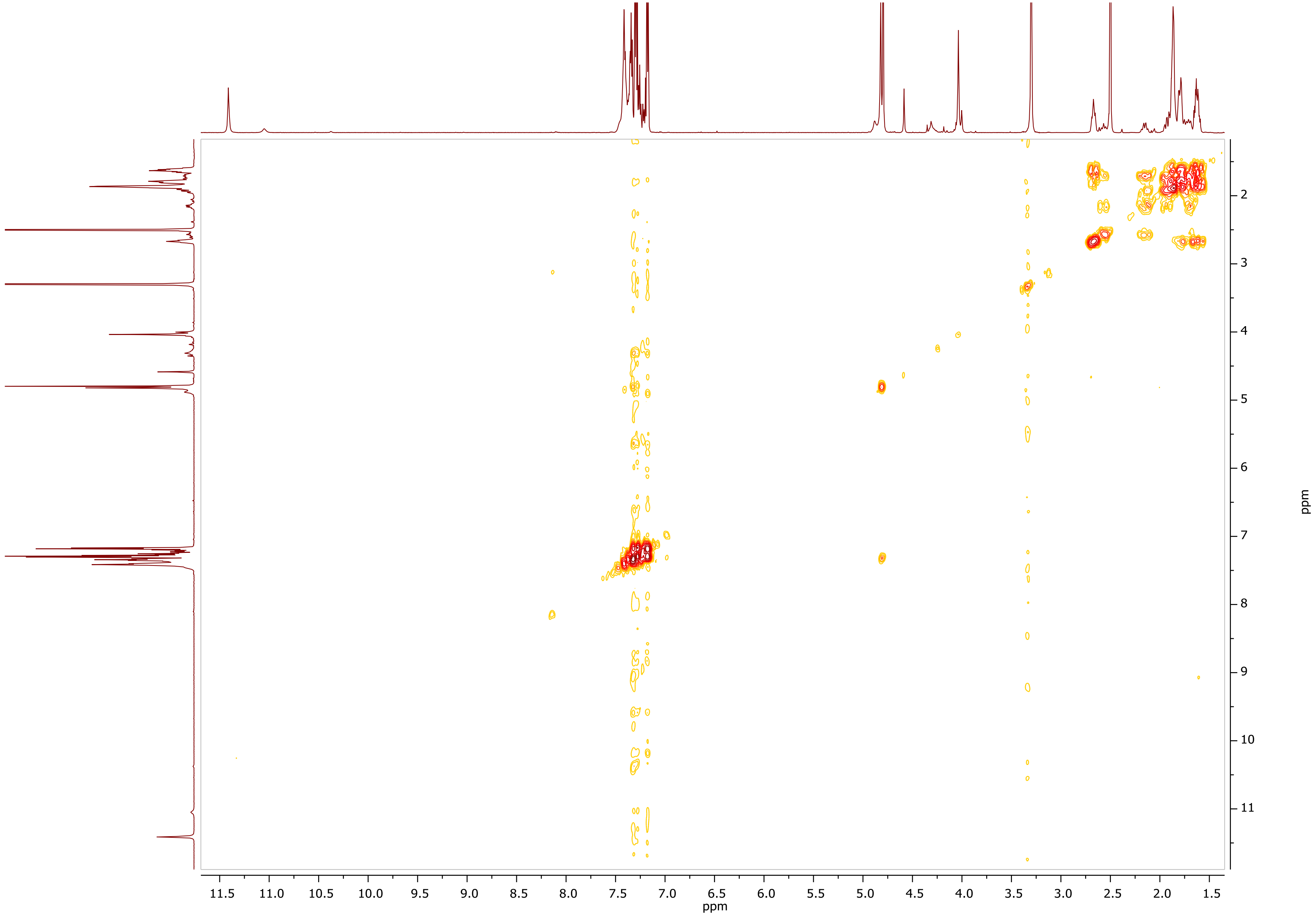

HSQC-DEPT NMR of **32** (400.13 MHz, DMSO-d_6)

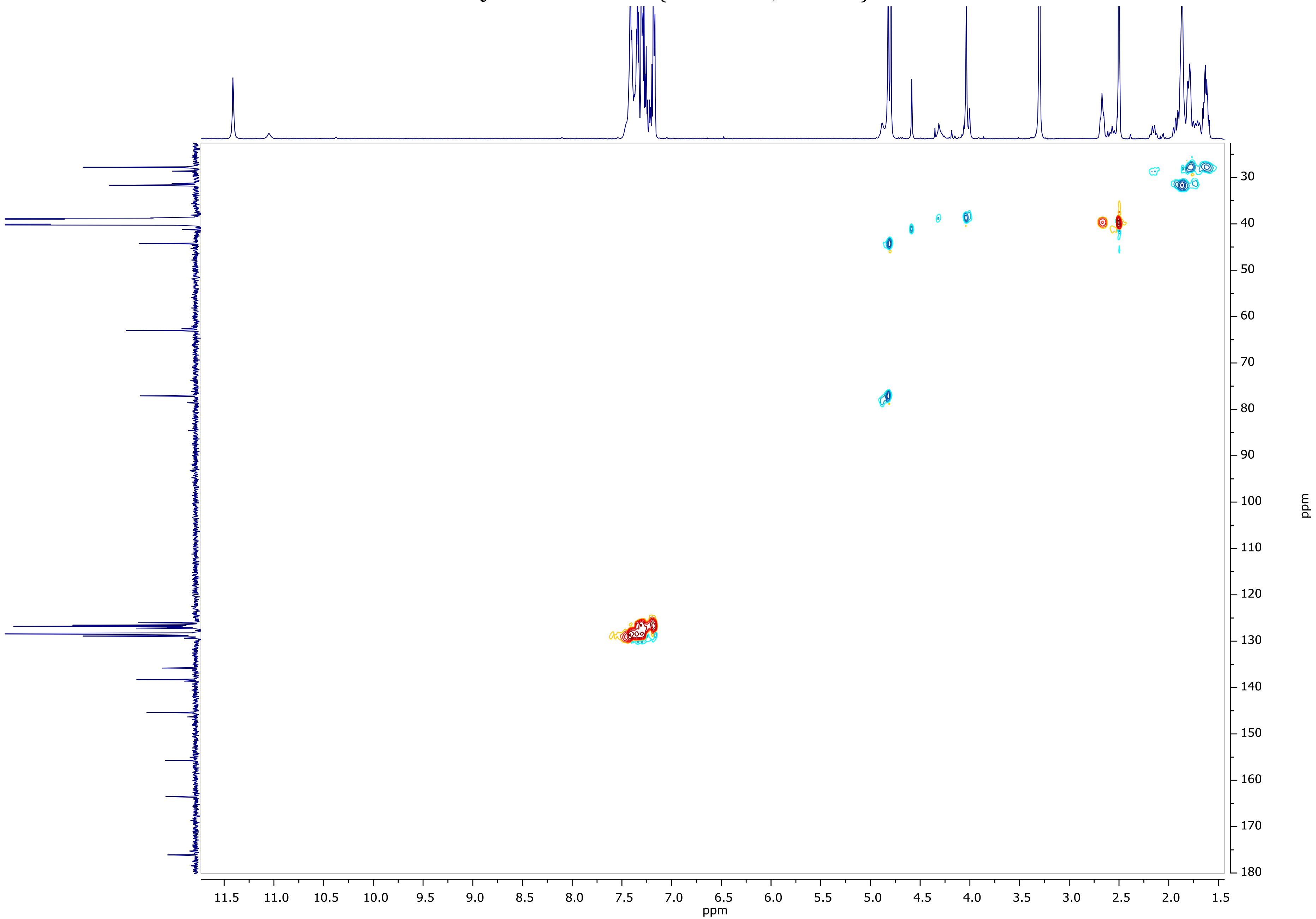

HMBC NMR of **32** (400.13 MHz, DMSO-d_6)

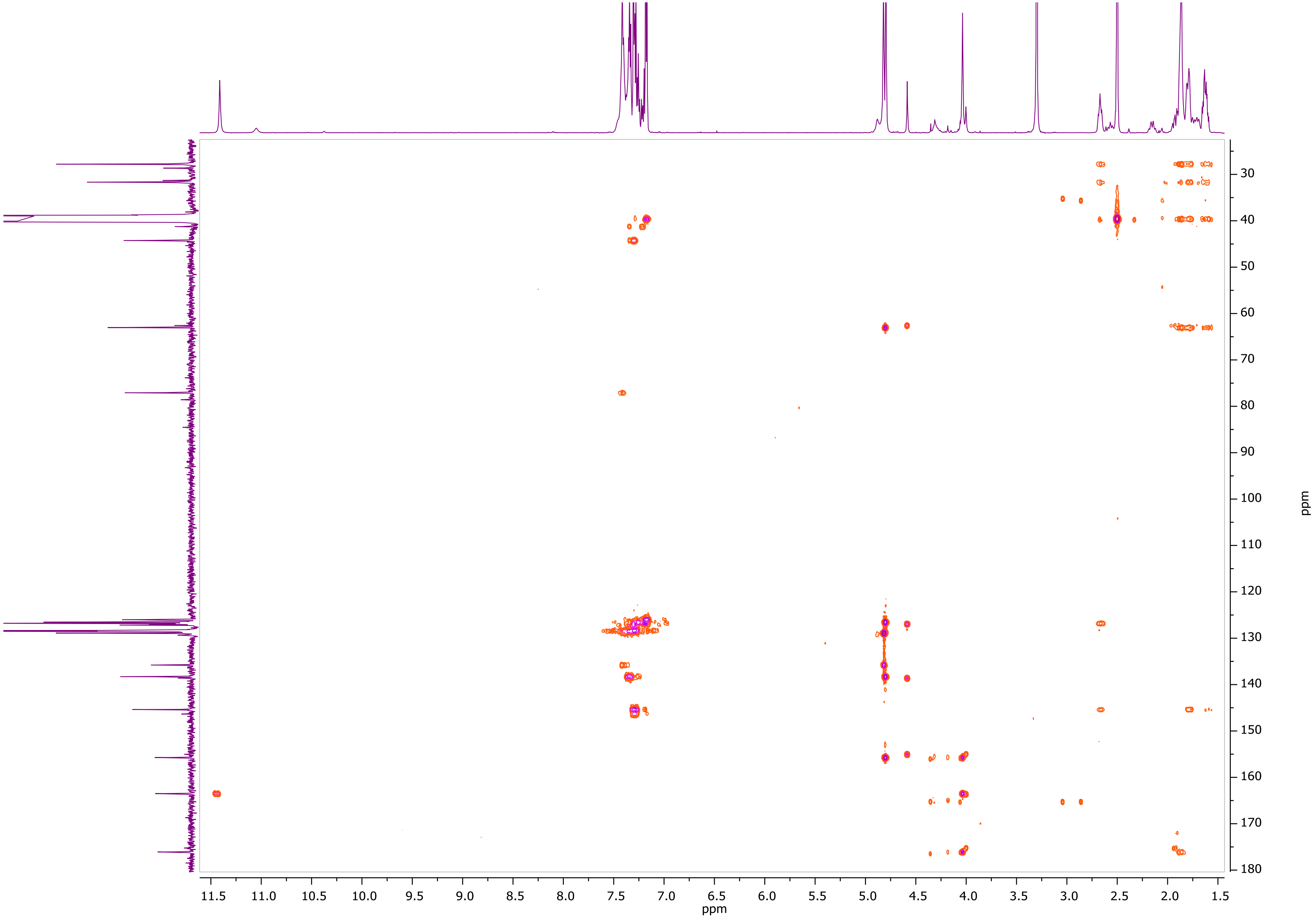

¹H NMR of **33** (600.11 MHz, DMSO-d_6)

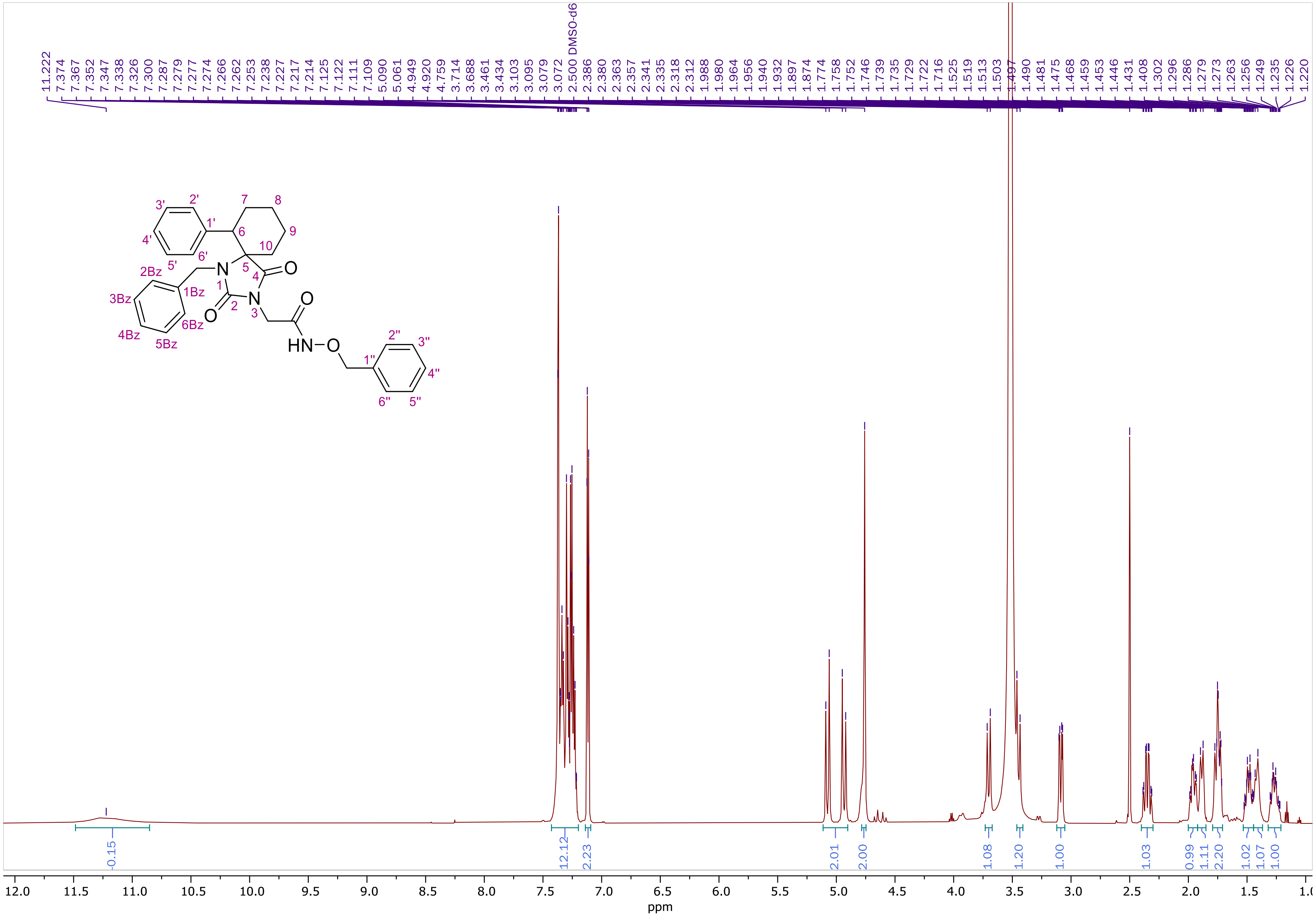

^{13}C NMR of **33** (50.32 MHz, DMSO-d_6)

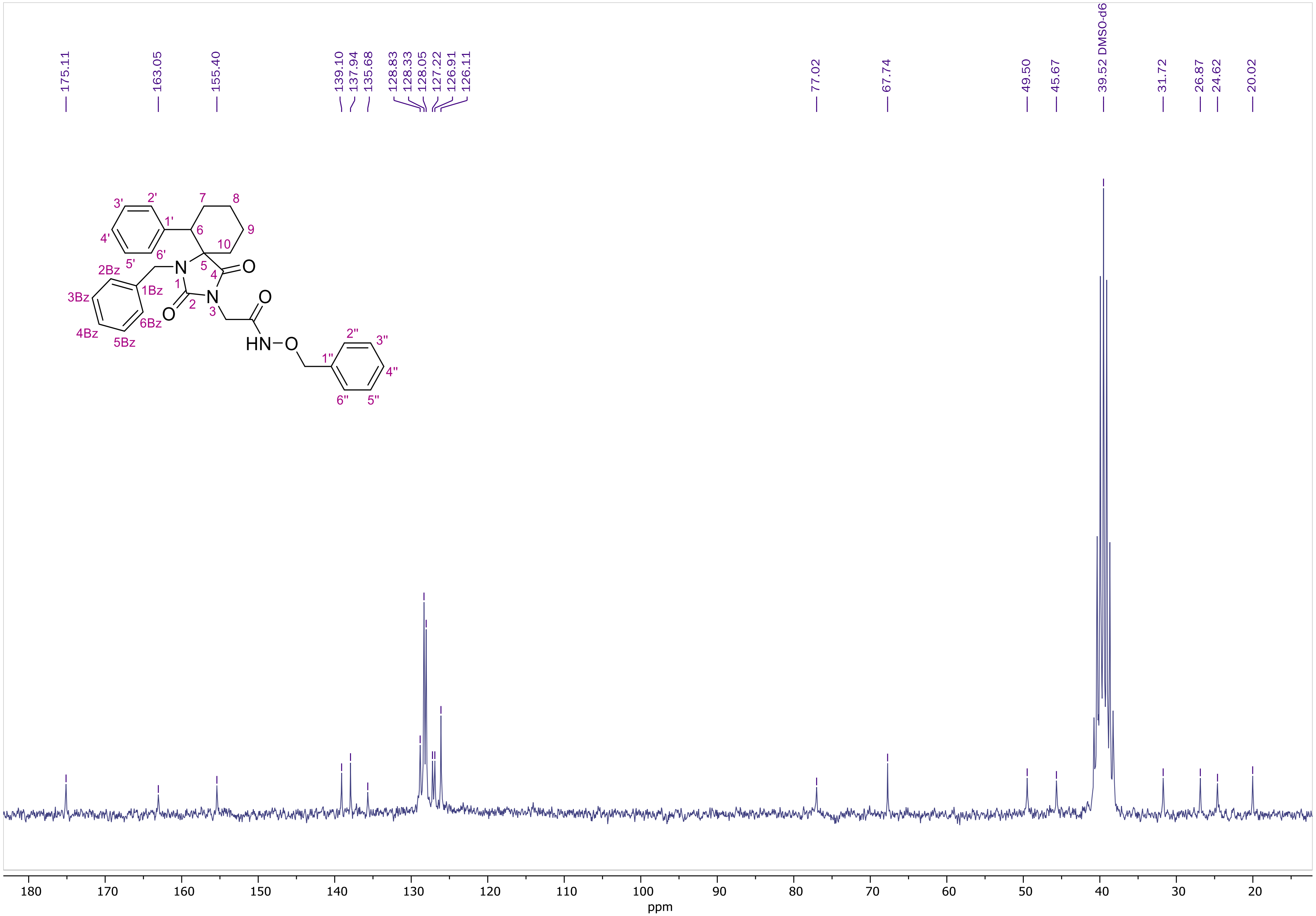

COSY NMR of **33** (400.13 MHz, DMSO-d_6)

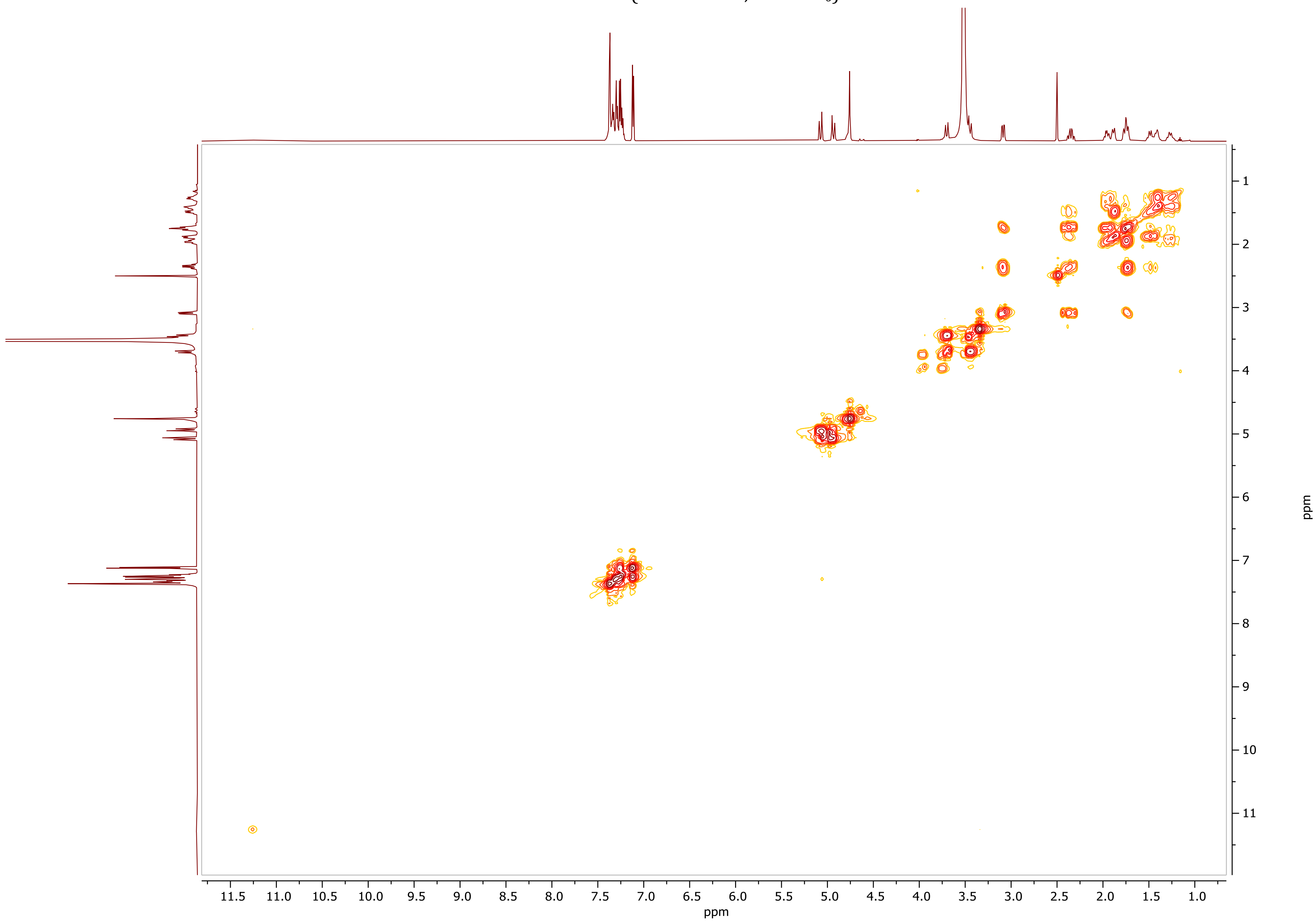

HSQC-DEPT NMR of **33** (400.13 MHz, DMSO-d_6)

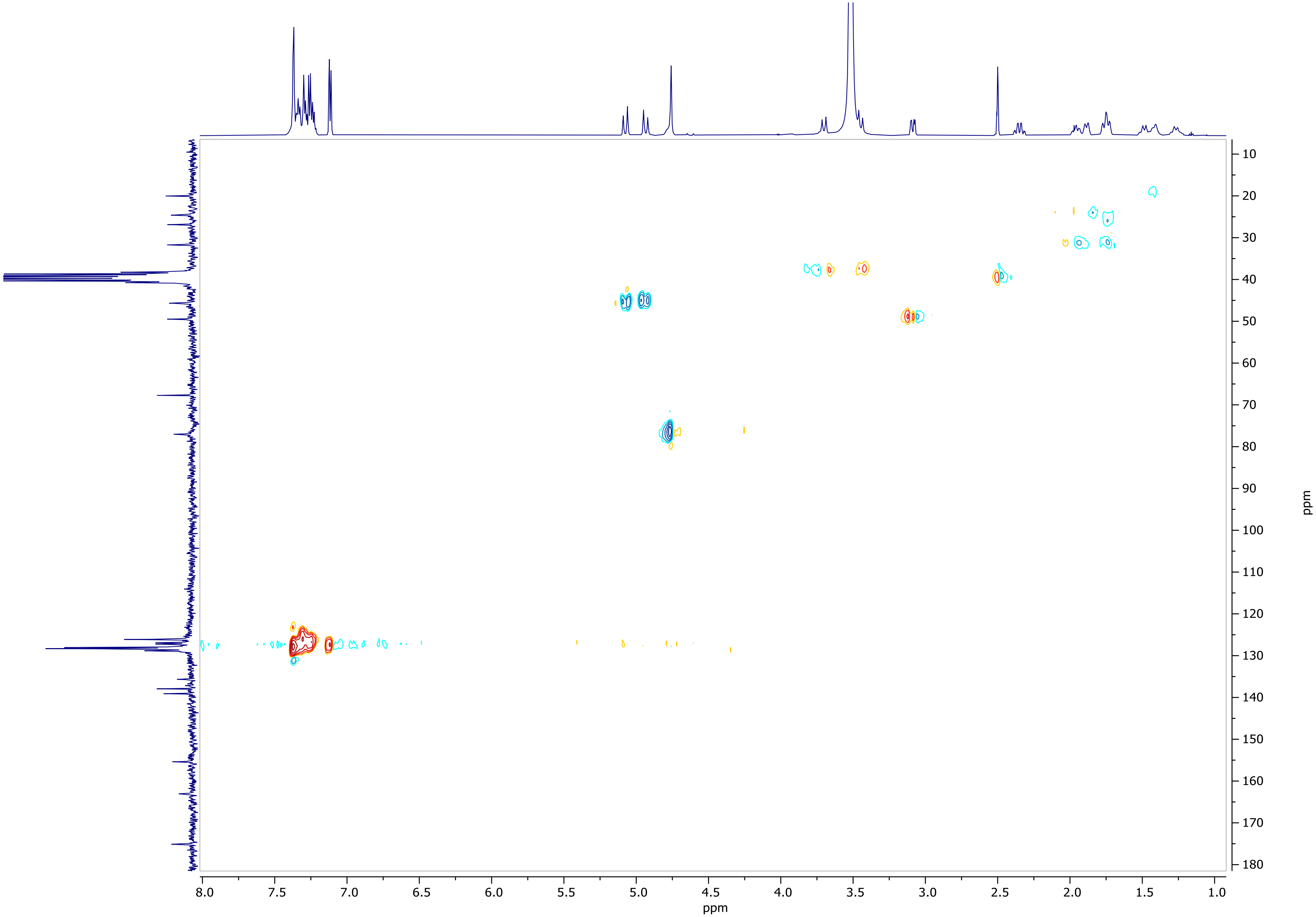

HMBC NMR of **33** (400.13 MHz, DMSO-d_6)

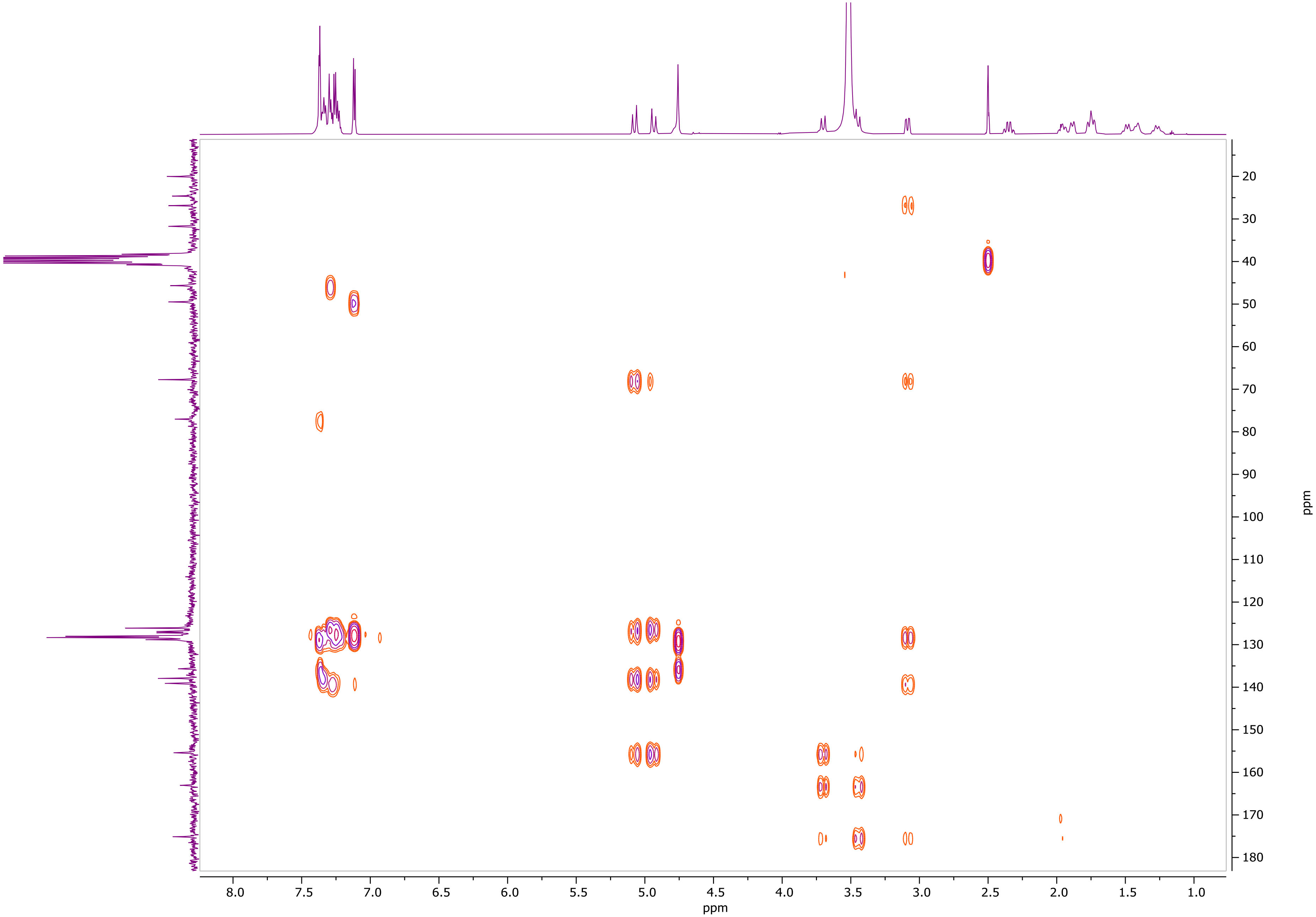

^{1}H NMR of **34** (600.11 MHz, DMSO-d_6)

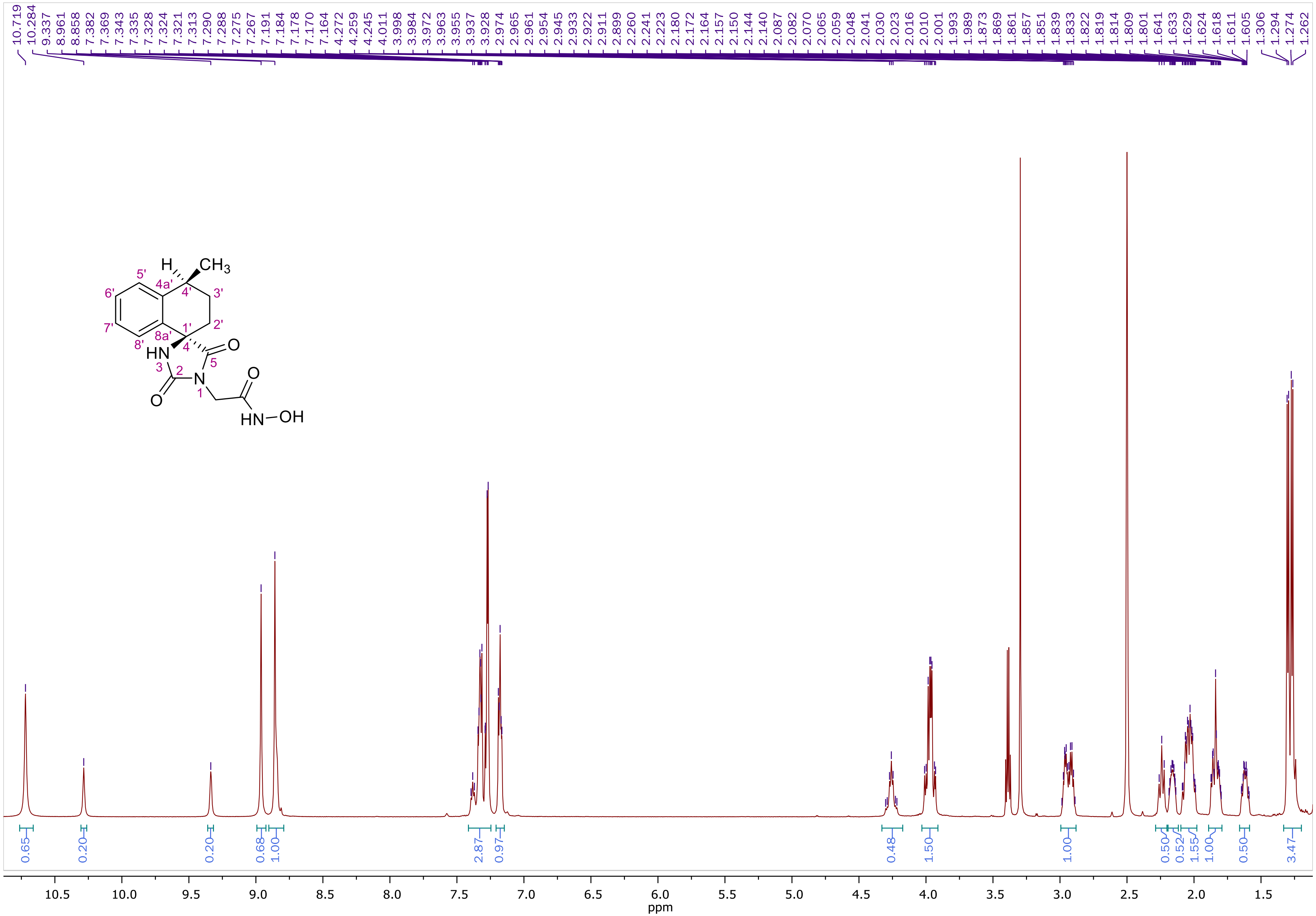

^{13}C NMR of **34** (150.9 MHz, DMSO-d_6)

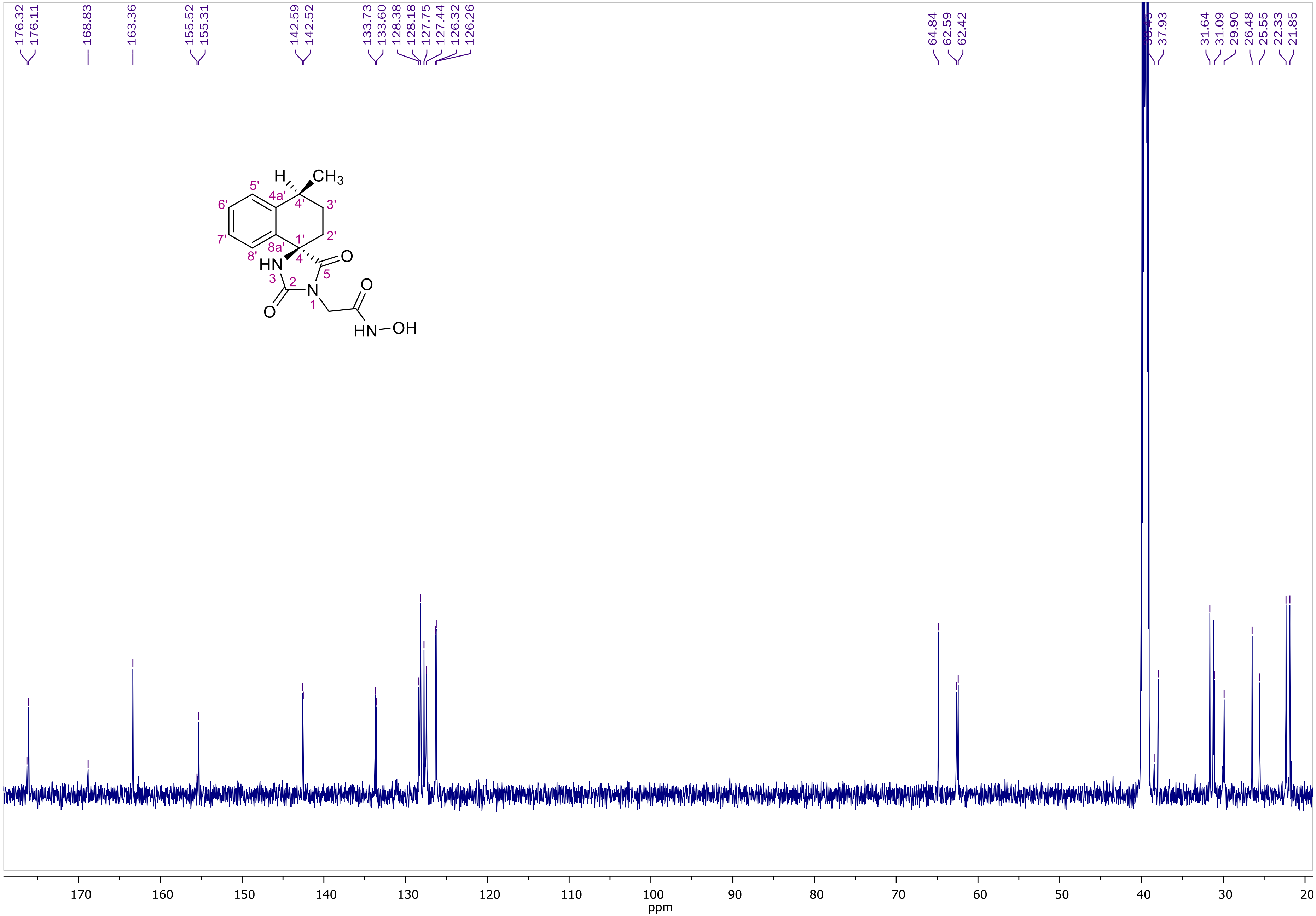

COSY NMR of **34** (600.11 MHz, DMSO-d_6)

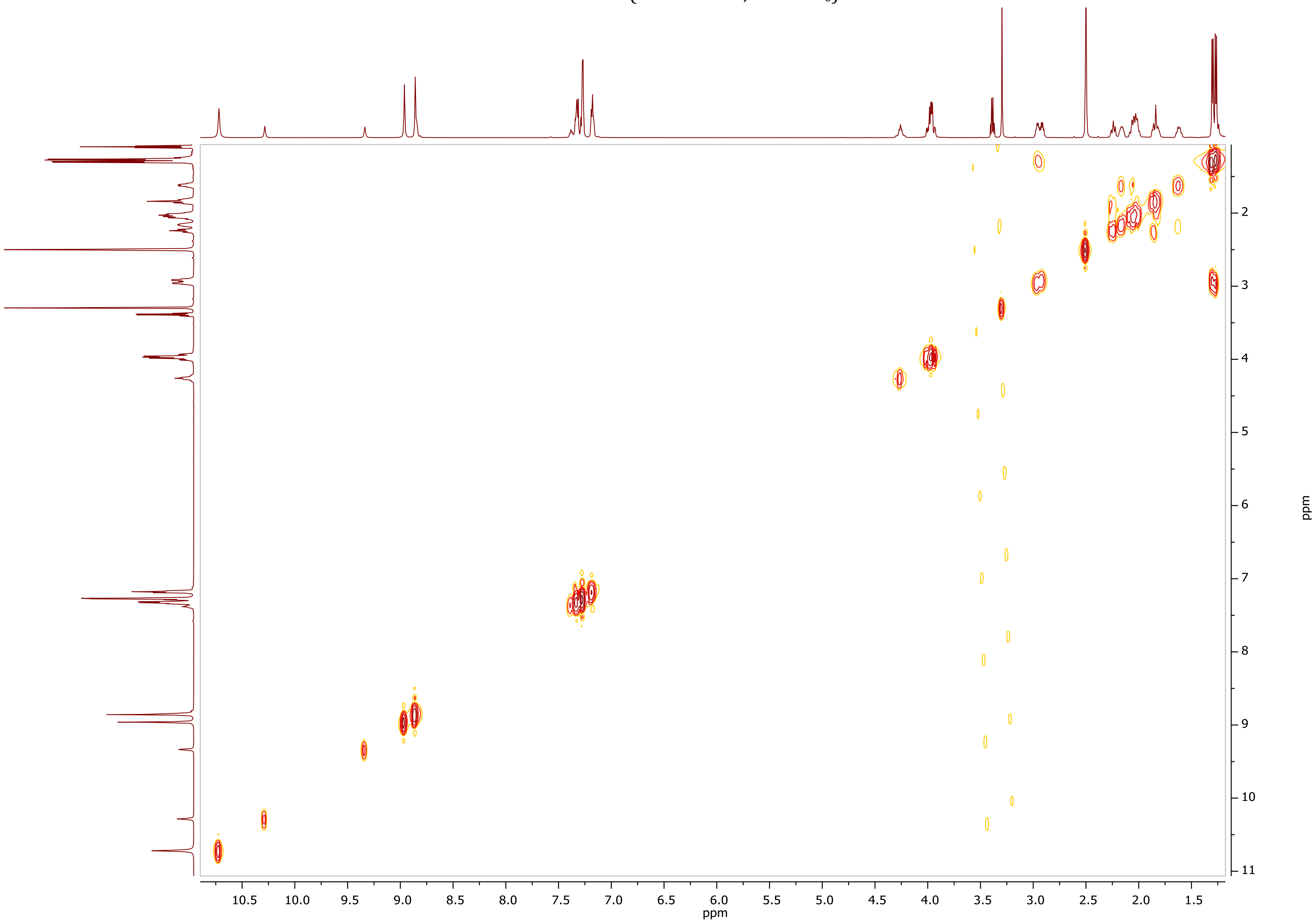

HSQC-DEPT NMR of **34** (600.11 MHz, DMSO-d_6)

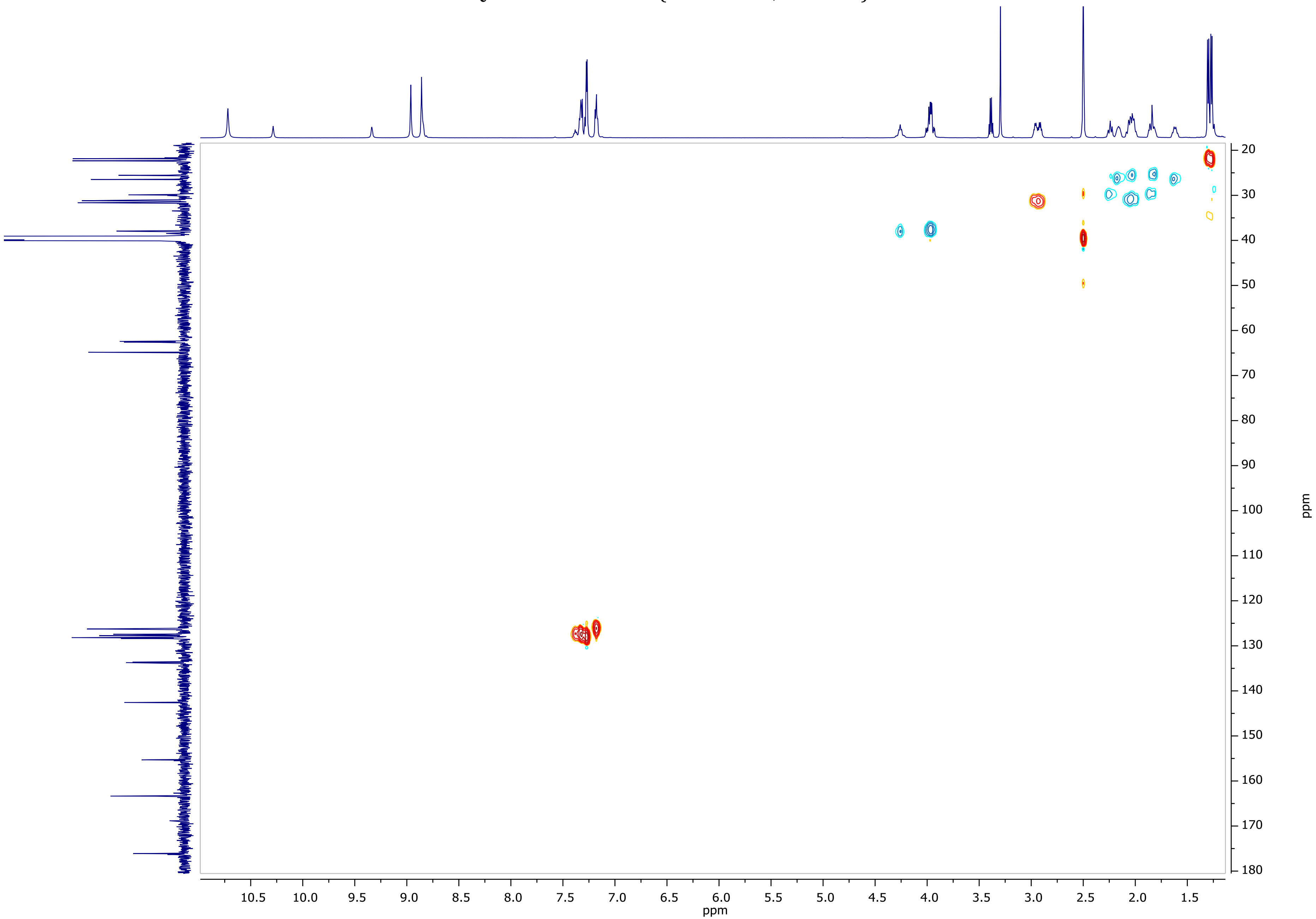

HMBC NMR of **34** (600.11 MHz, DMSO-d_6)

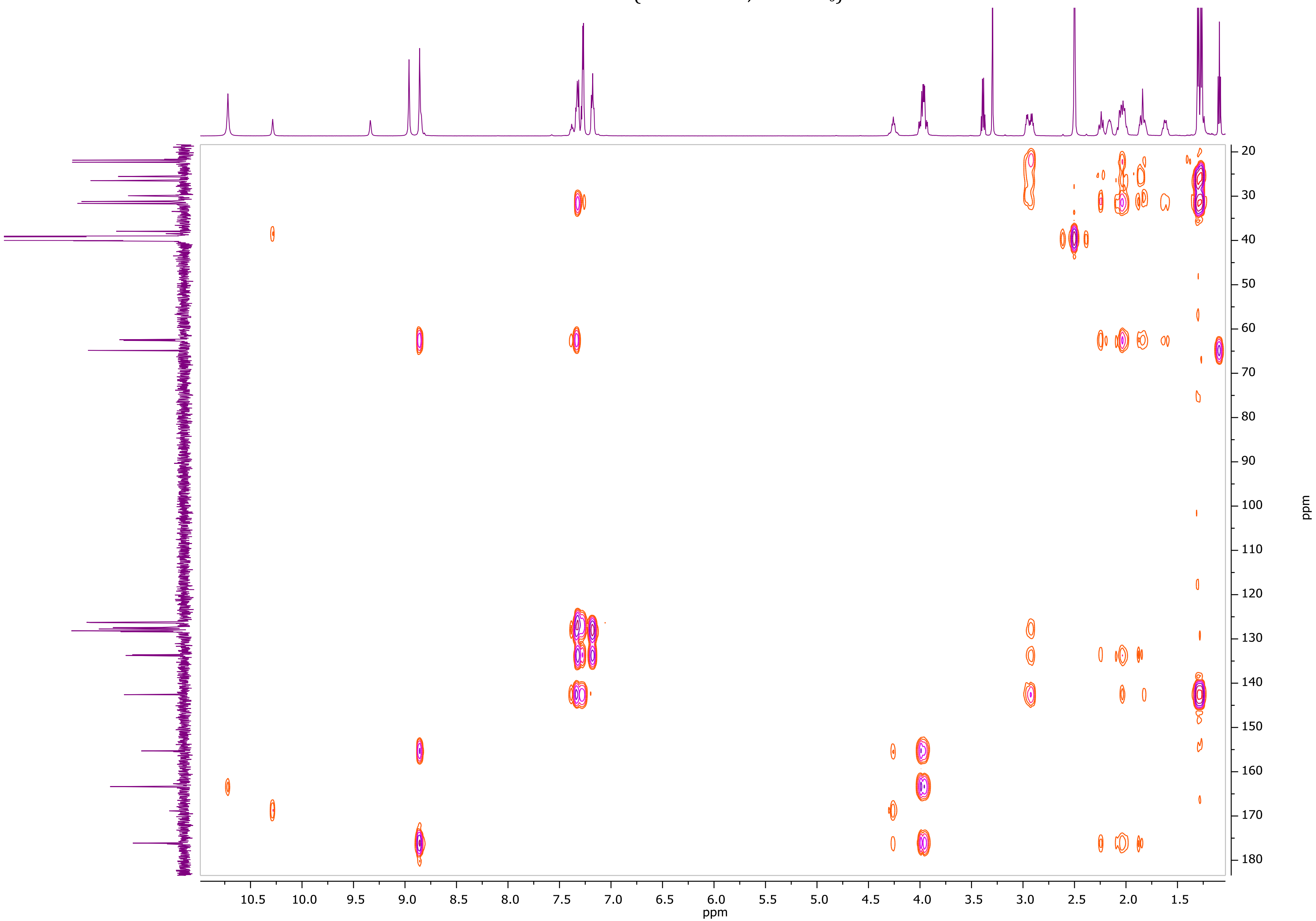

NOESY NMR of **34** (600.11 MHz, DMSO-d_6)

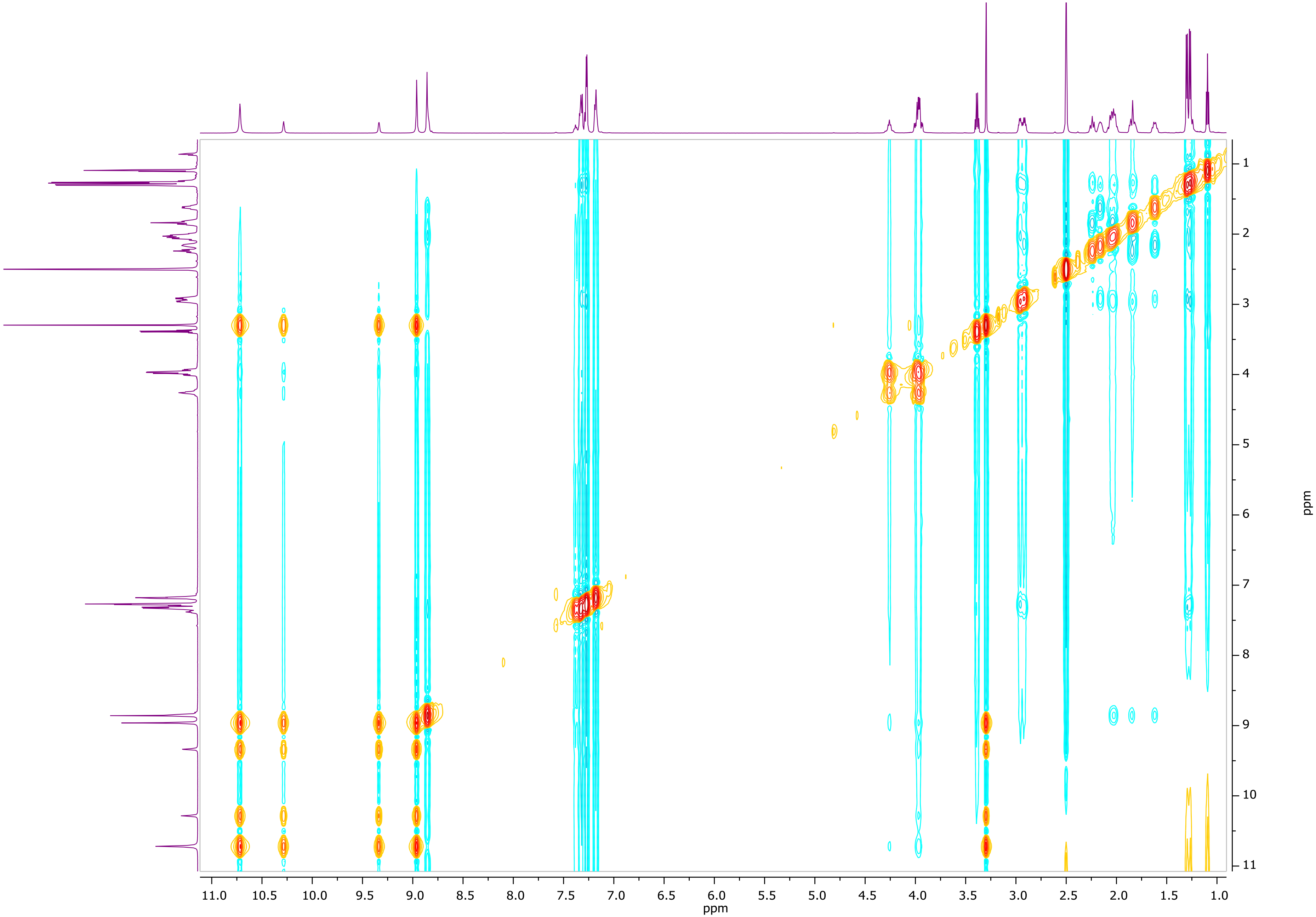

^{1}H NMR of **35** (400.13 MHz, DMSO-d_6)

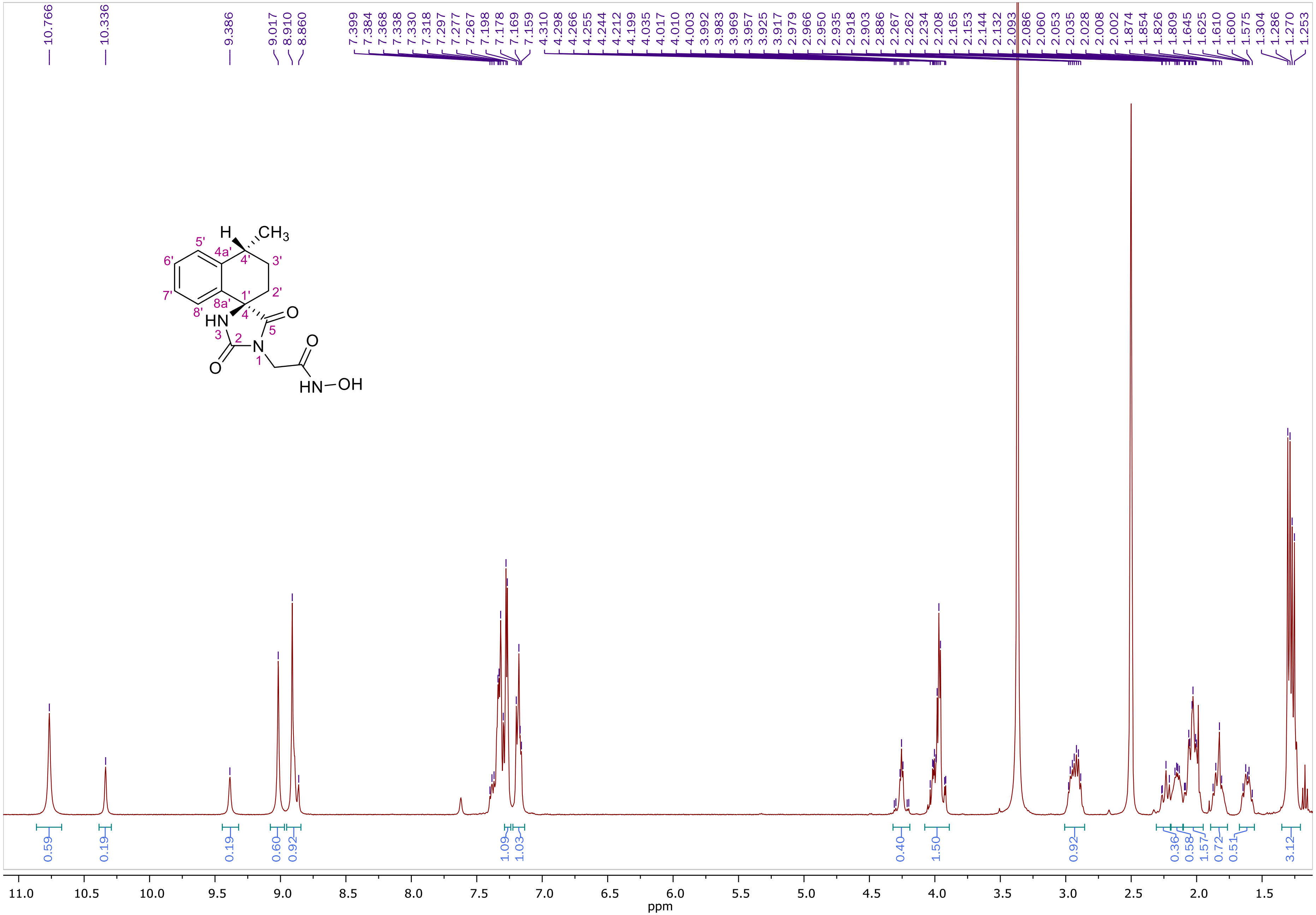

^{13}C NMR of **35** (150.9 MHz, DMSO-d_6)

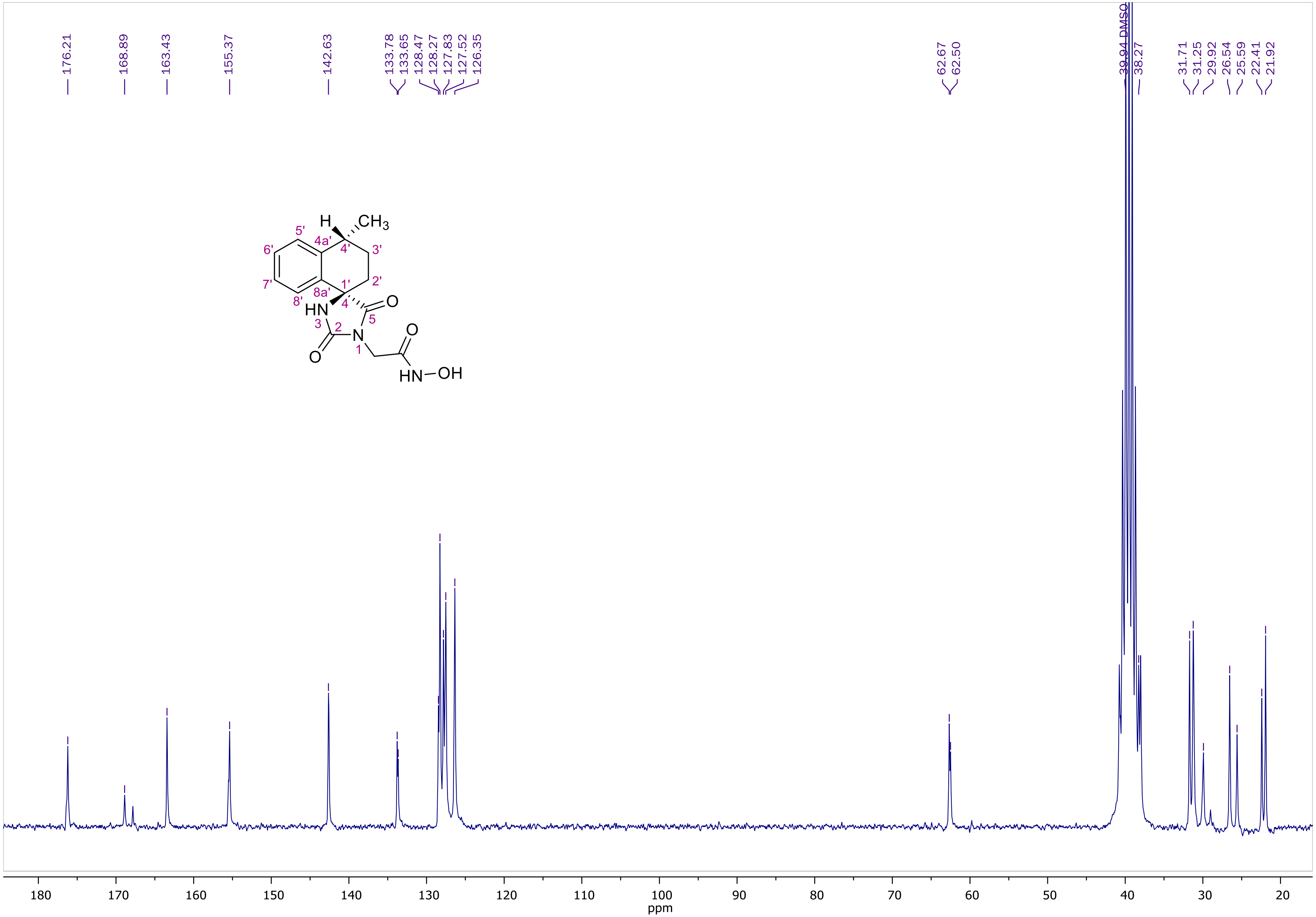

COSY NMR of **35** (400.13 MHz, DMSO-d_6)

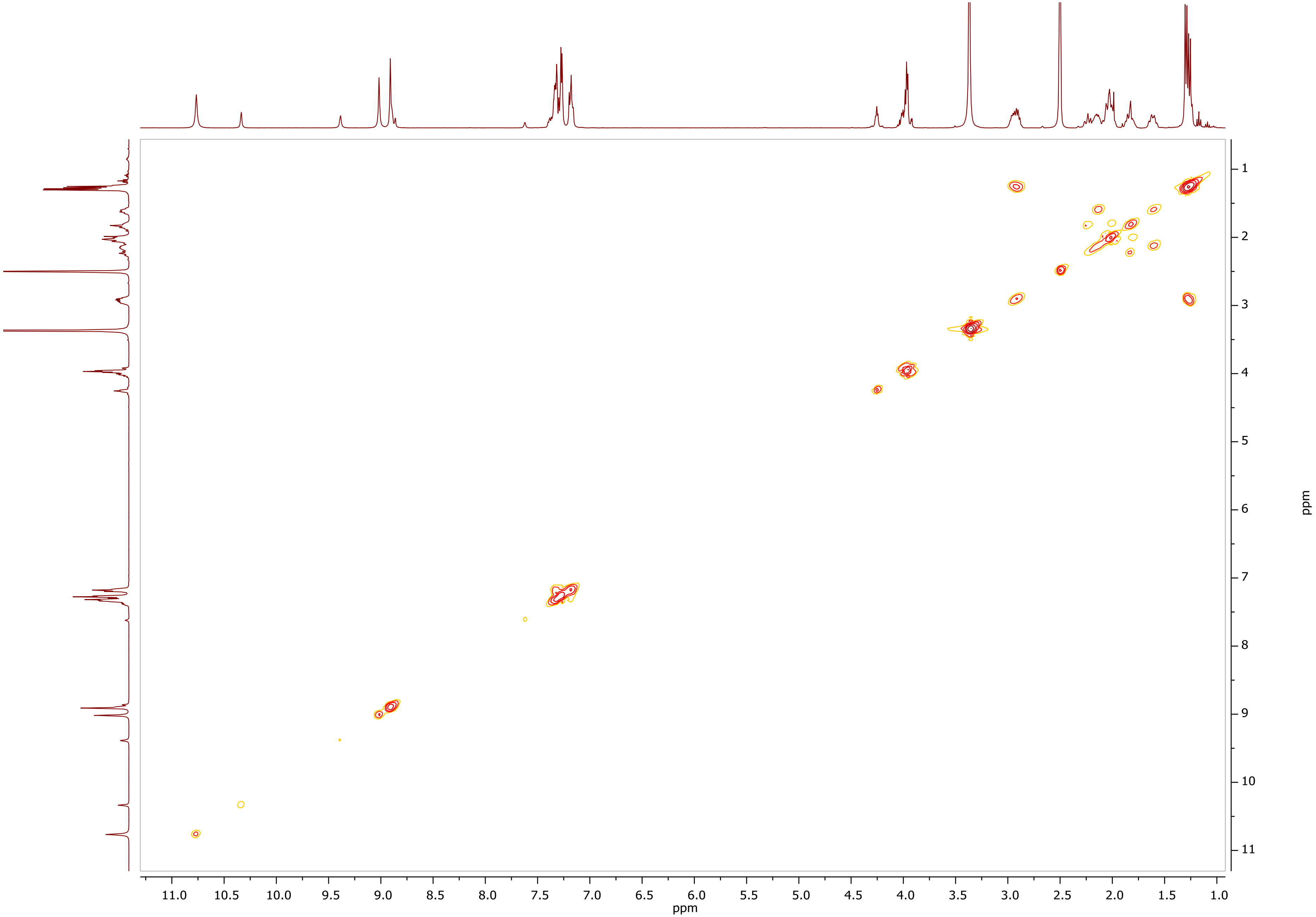

HSQC NMR of **35** (400.13 MHz, DMSO-d_6)

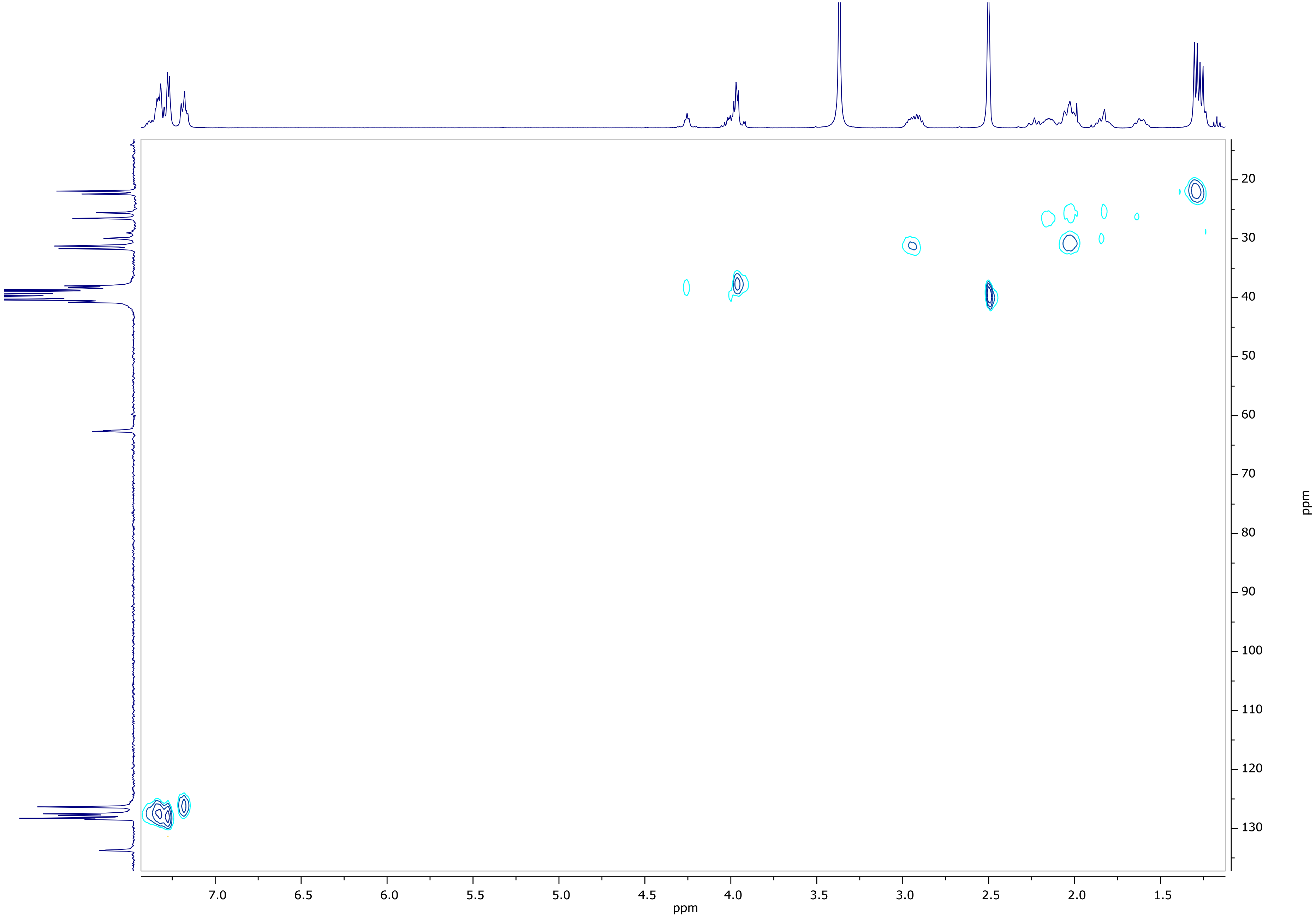

HMBC NMR of **35** (400.13 MHz, DMSO-d_6)

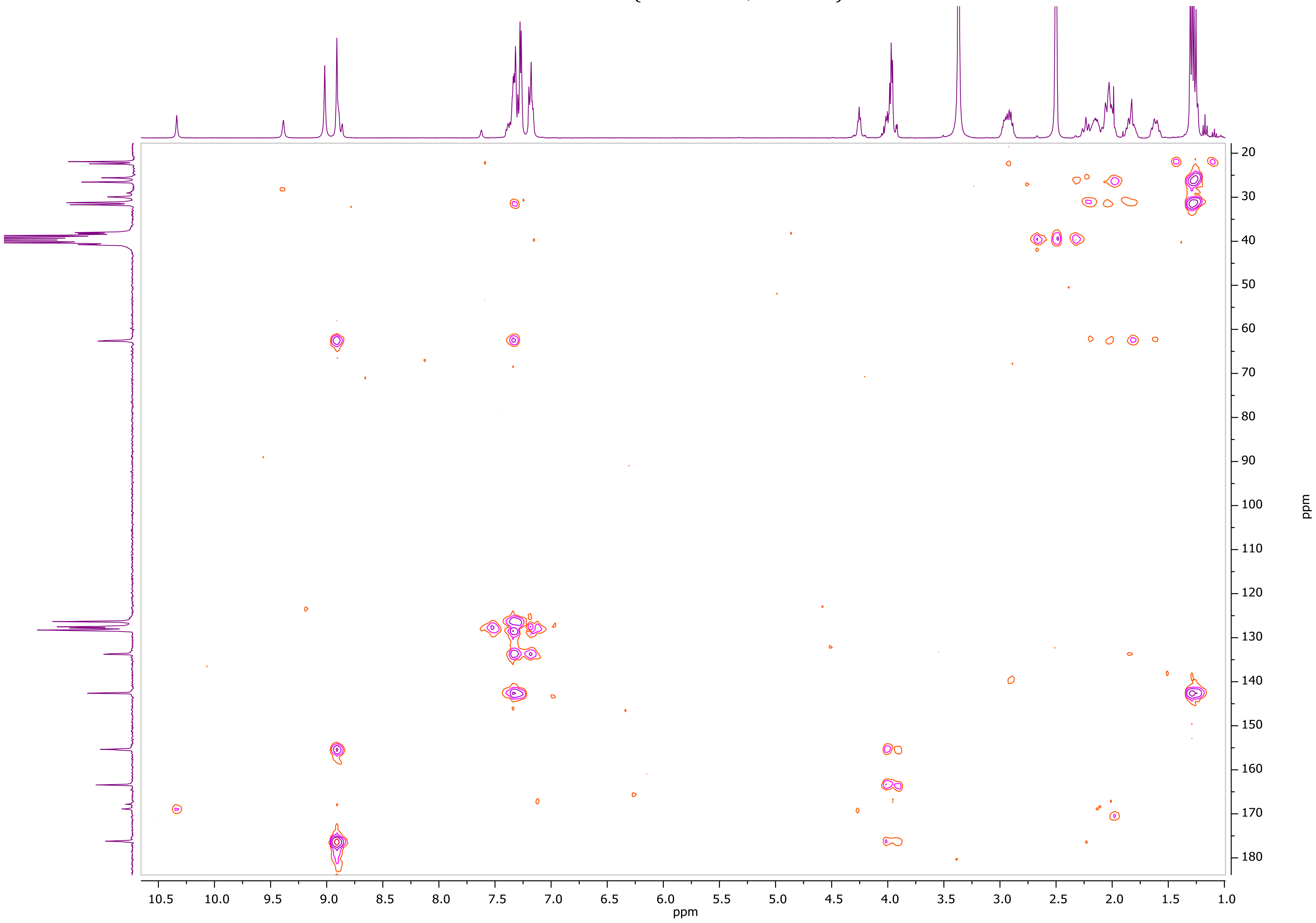

NOESY NMR of **35** (400.13 MHz, DMSO-d_6)

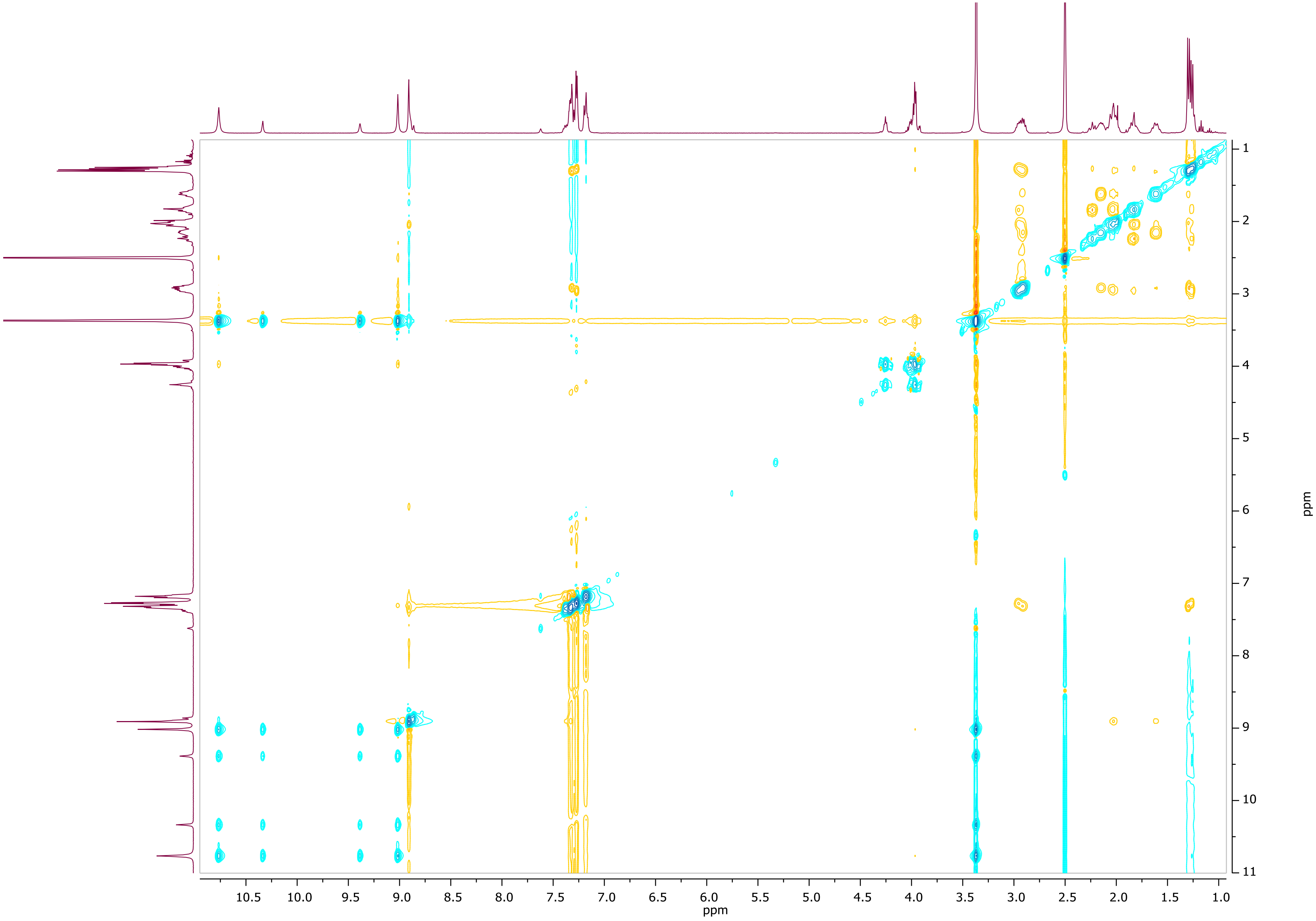

^{1}H NMR of **36** (600.11 MHz, DMSO-d_6)

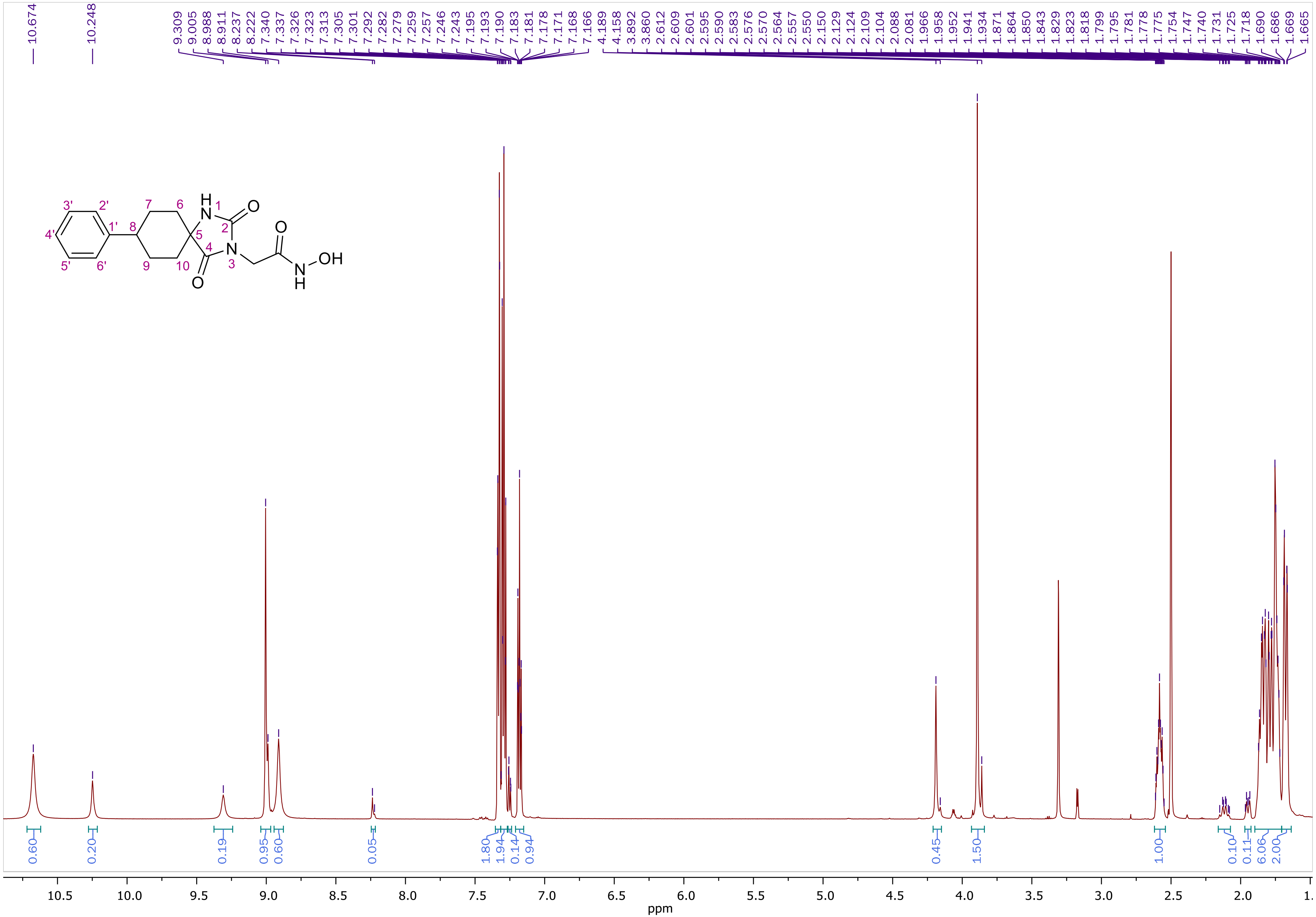

^{13}C NMR of **36** (150.9 MHz, DMSO-d_6)

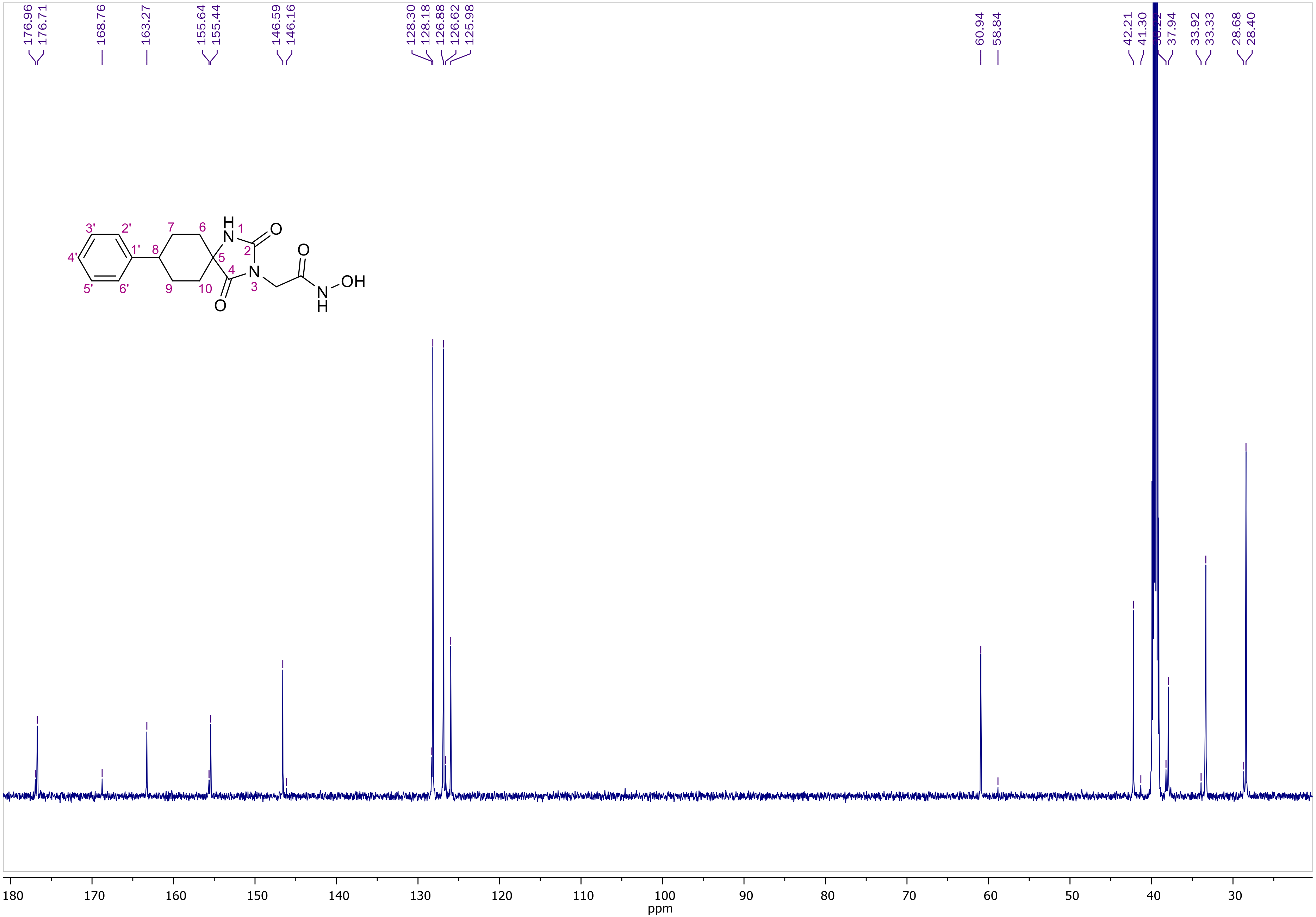

COSY NMR of **36** (600.11 MHz, DMSO-d_6)

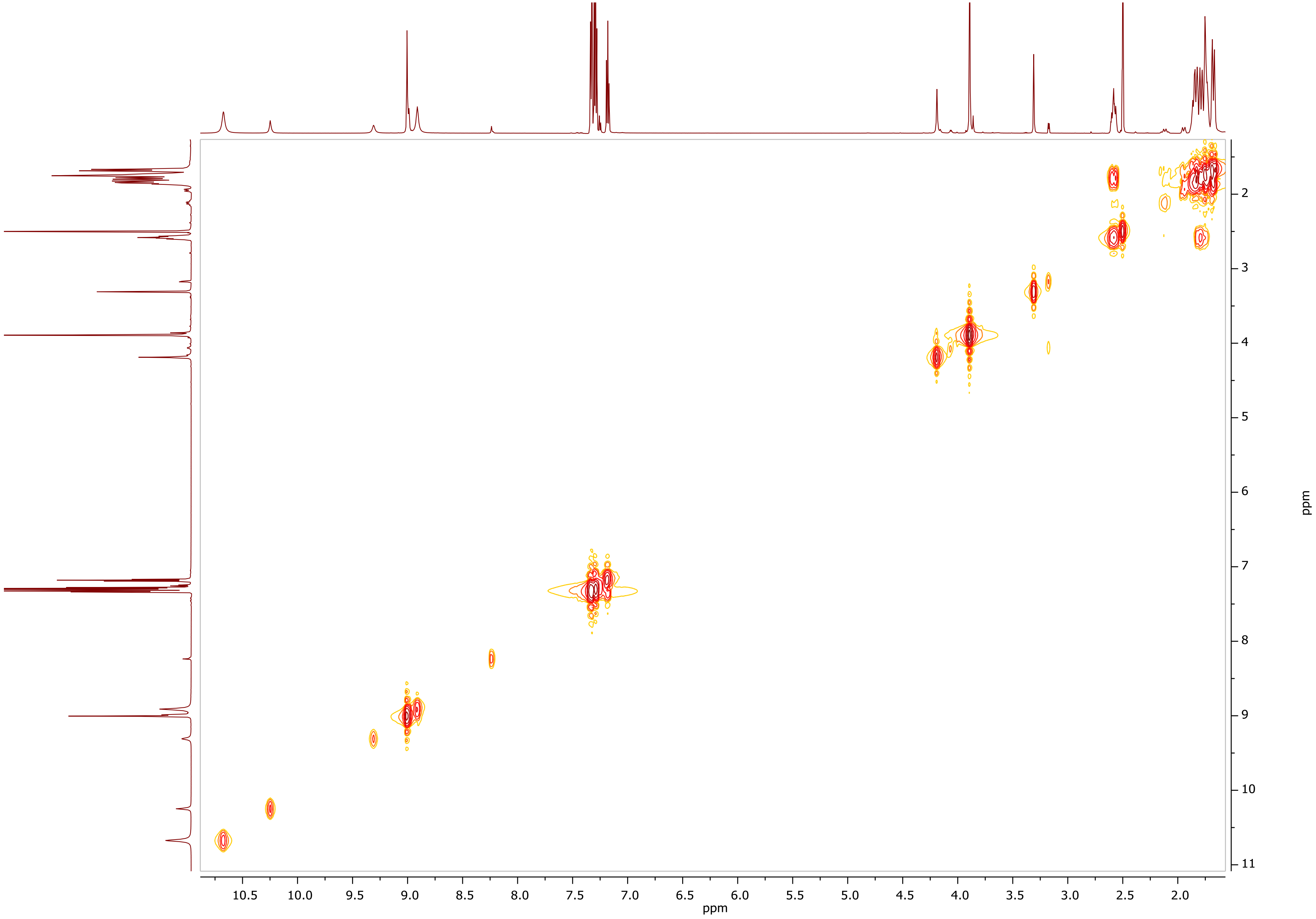

HSQC-DEPT NMR of **36** (600.11 MHz, DMSO-d_6)

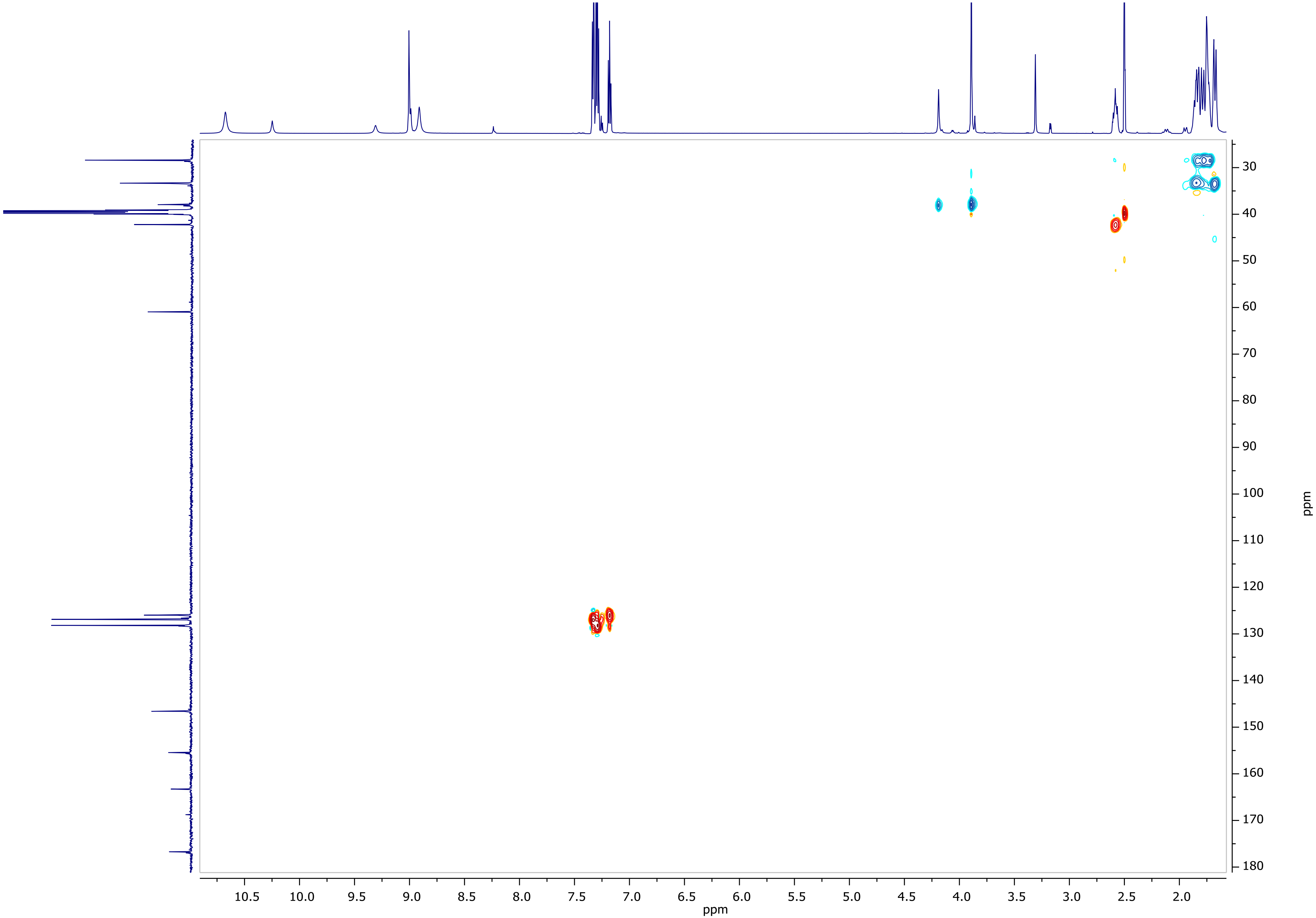

HMBC NMR of **36** (600.11 MHz, DMSO-d_6)

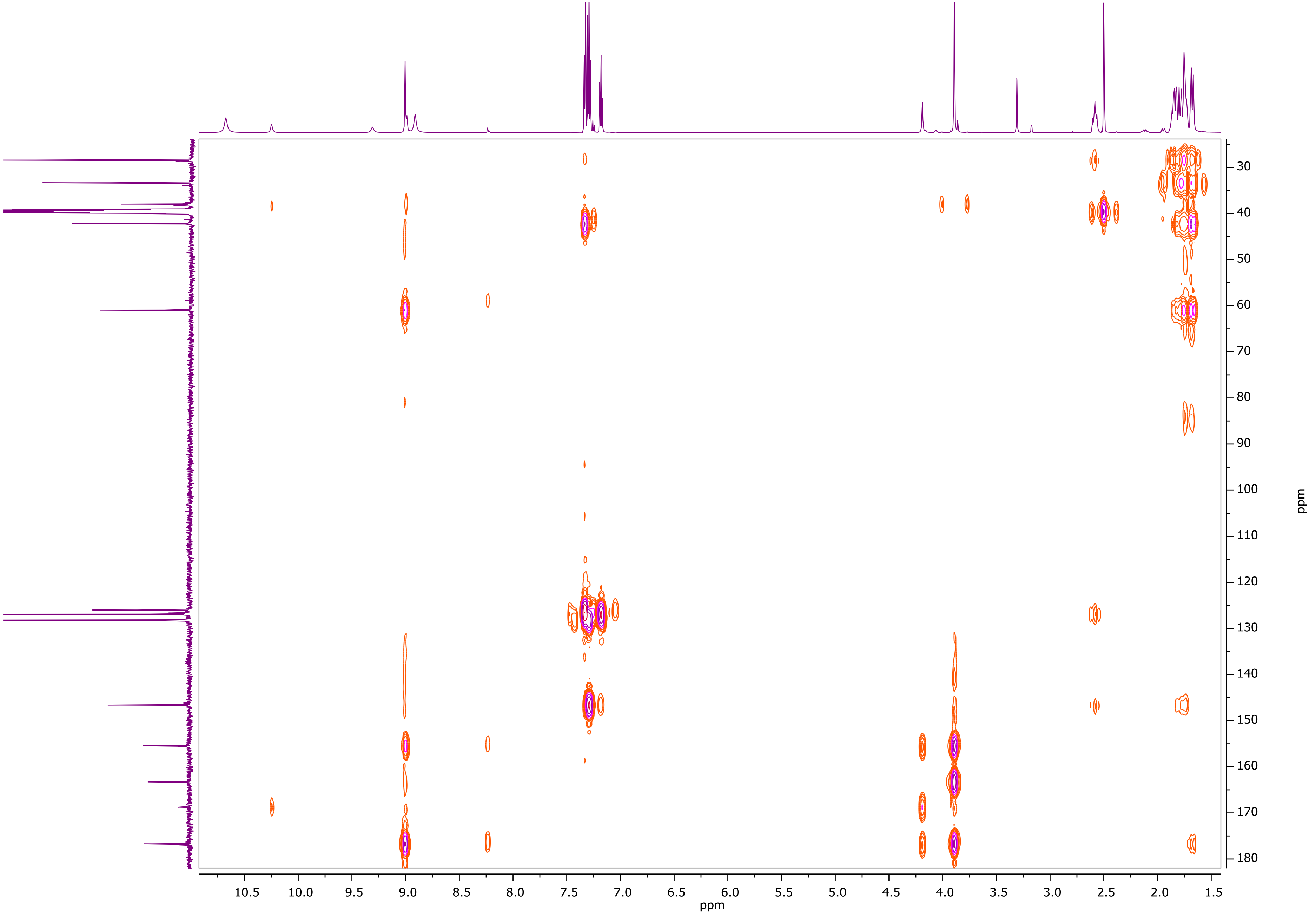

^1H NMR of **37** (600.11 MHz, DMSO-d_6)

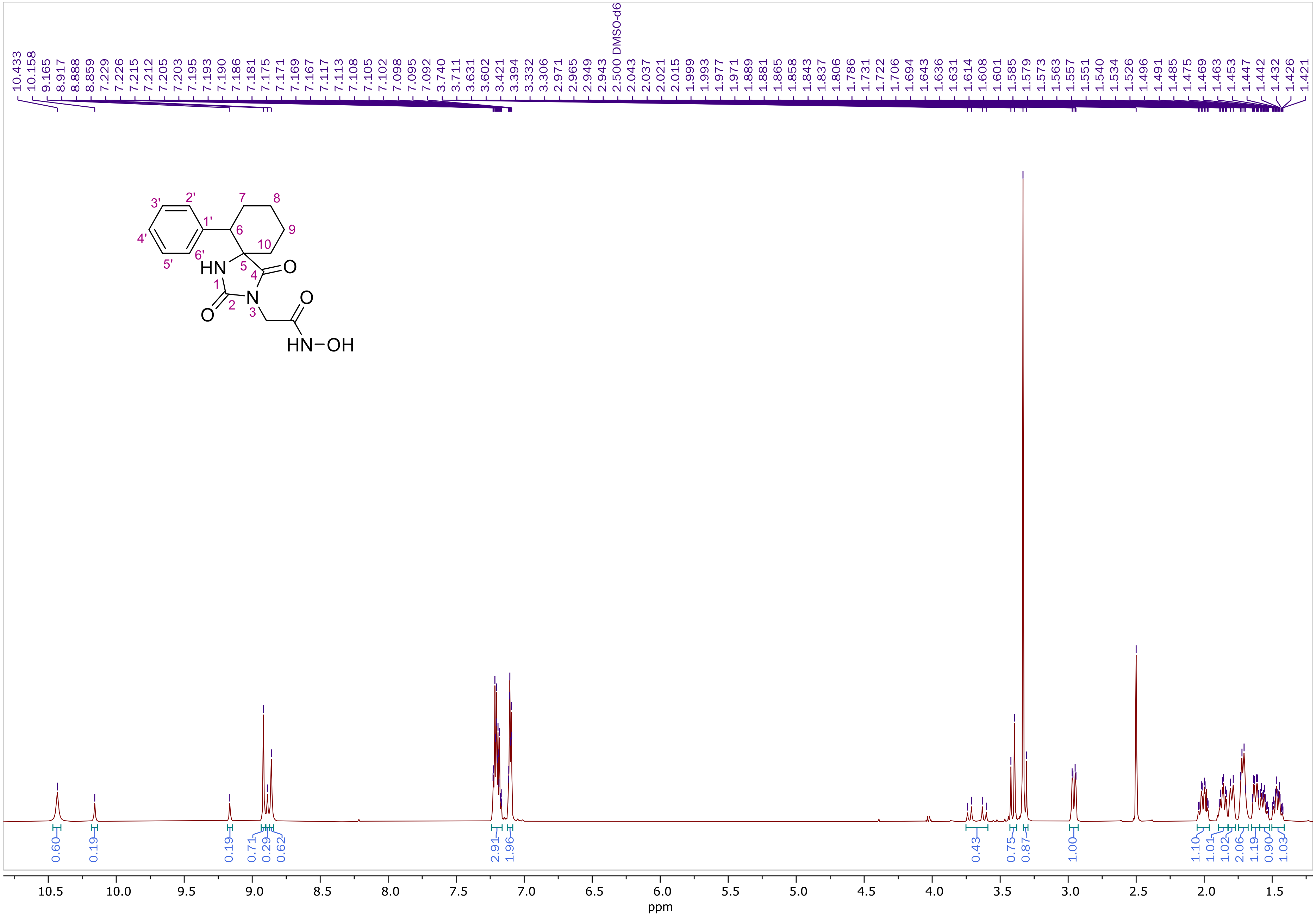

^{13}C NMR of **37** (150.9 MHz, DMSO-d_6)

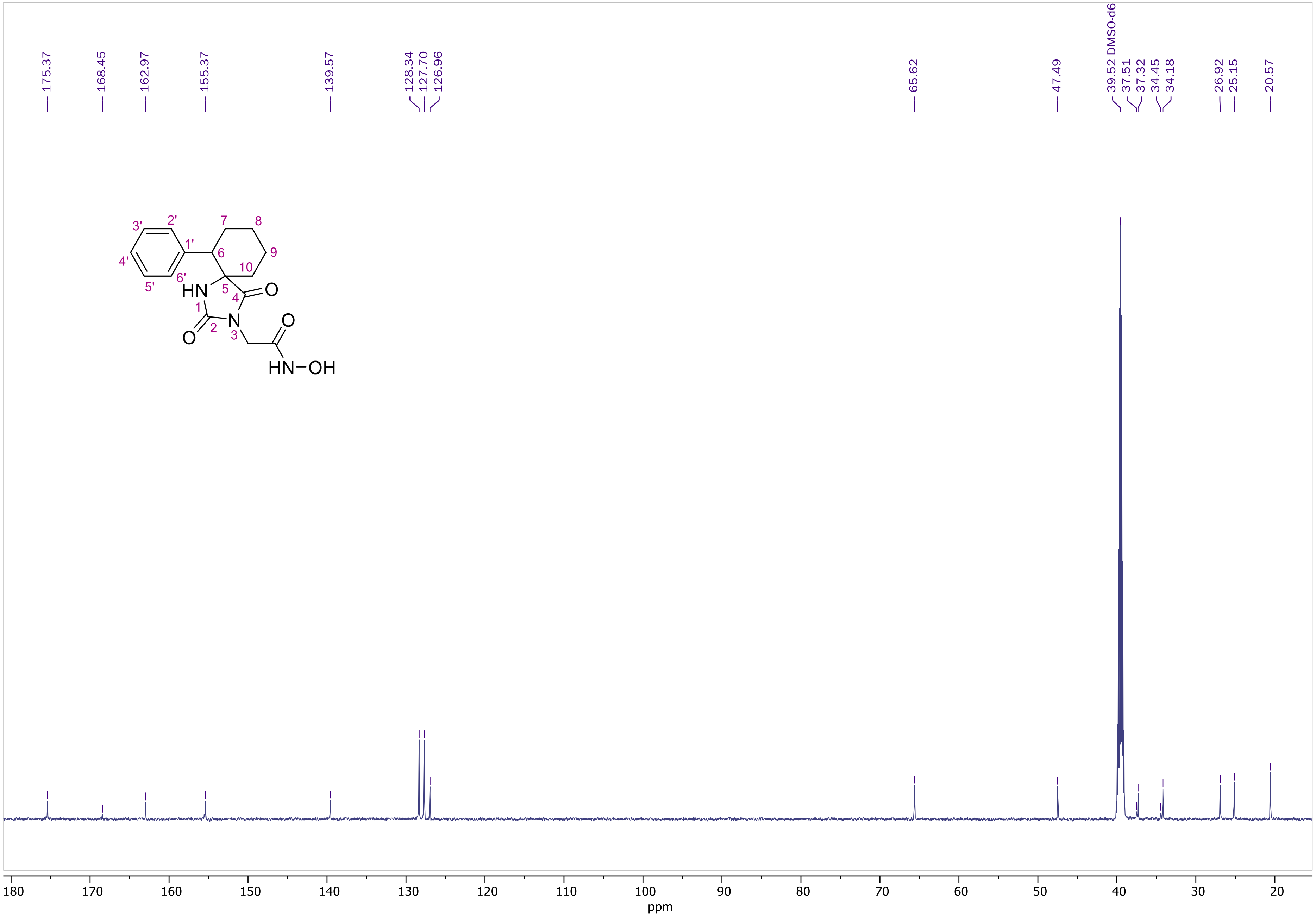

COSY NMR of **37** (400.13 MHz, DMSO-d_6)

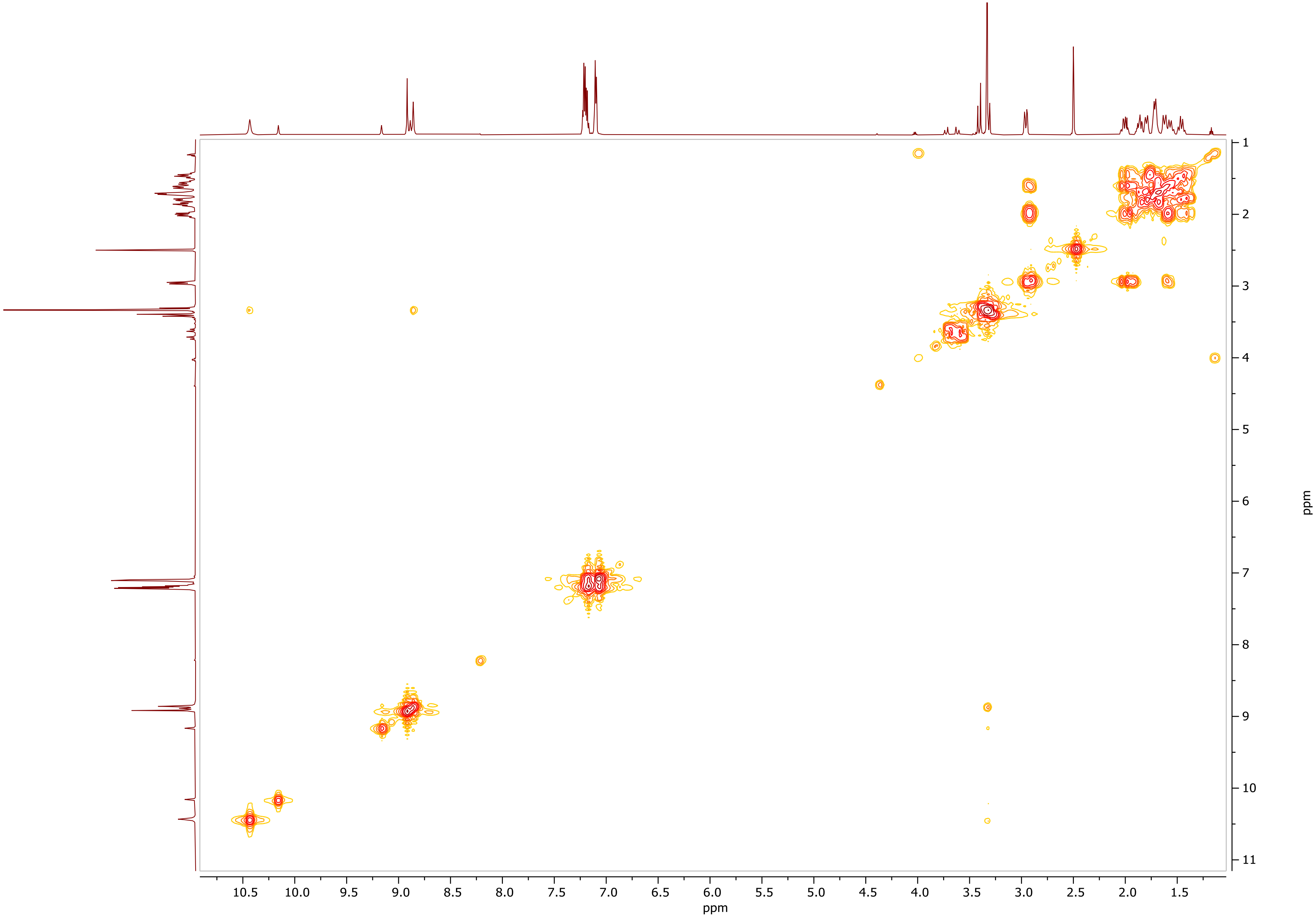

HSQC NMR of **37** (400.13 MHz, DMSO-d_6)

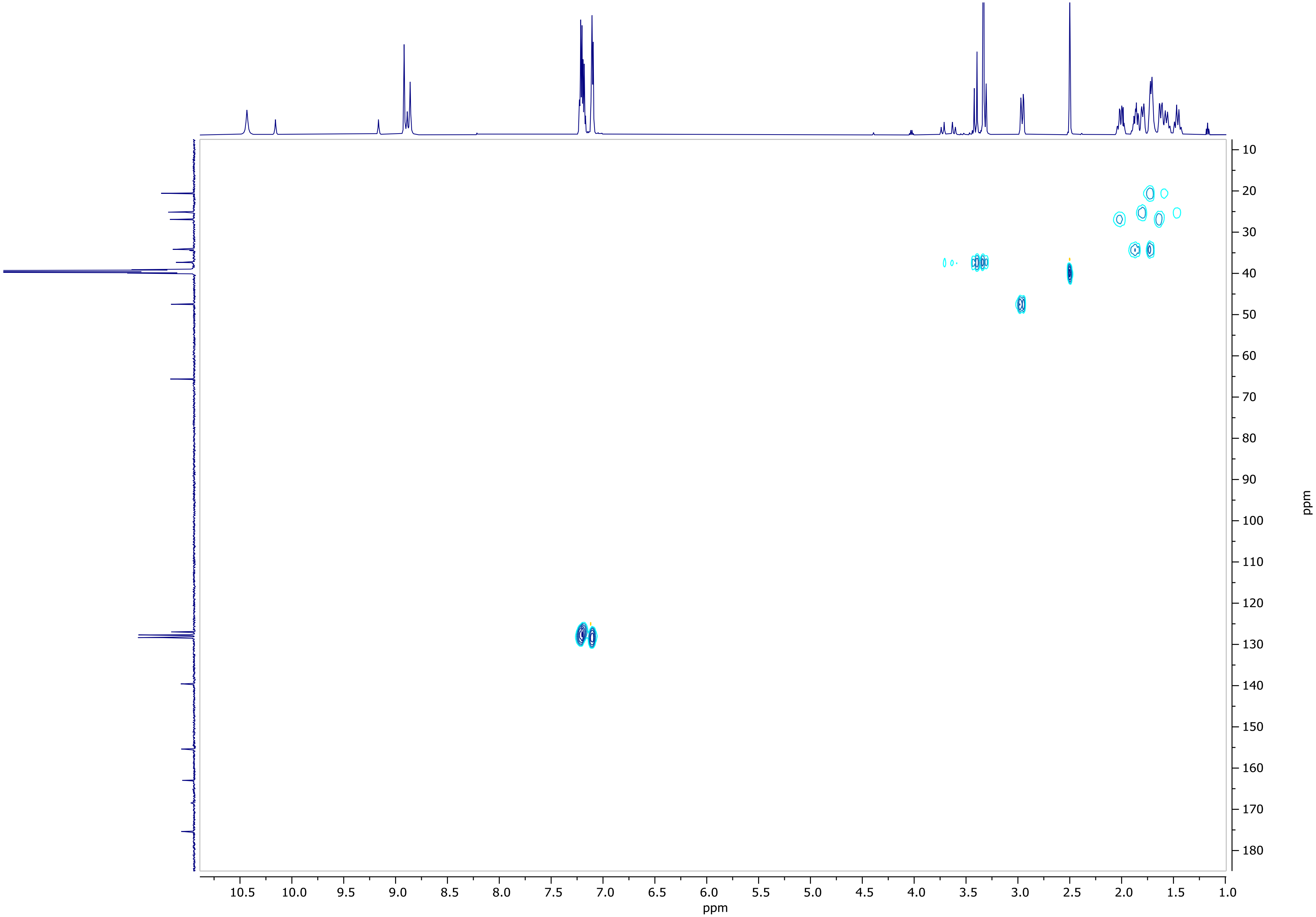

HMBC NMR of **37** (400.13 MHz, DMSO-d_6)

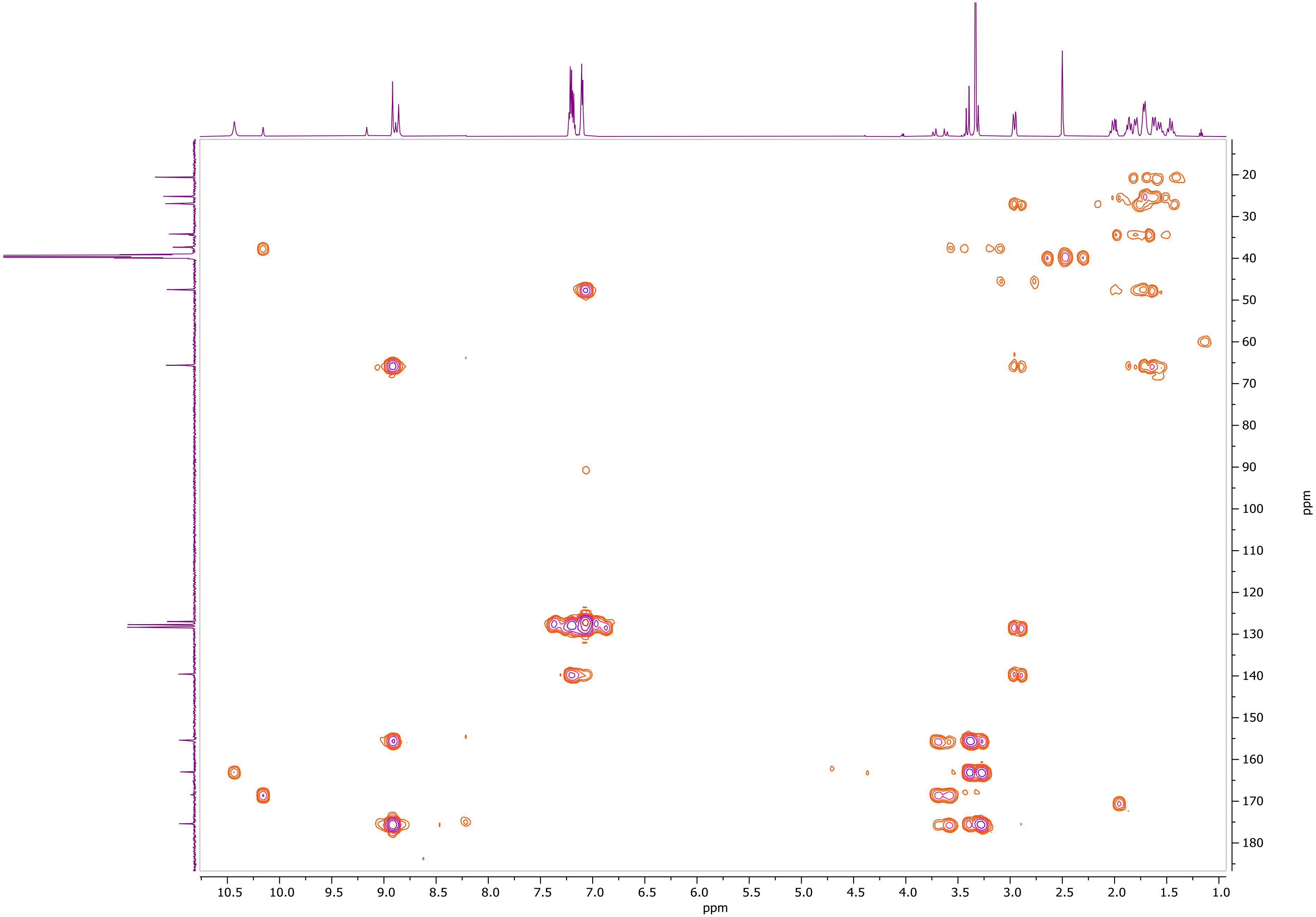

^{1}H NMR of **38** (400.13 MHz, DMSO-d_6)

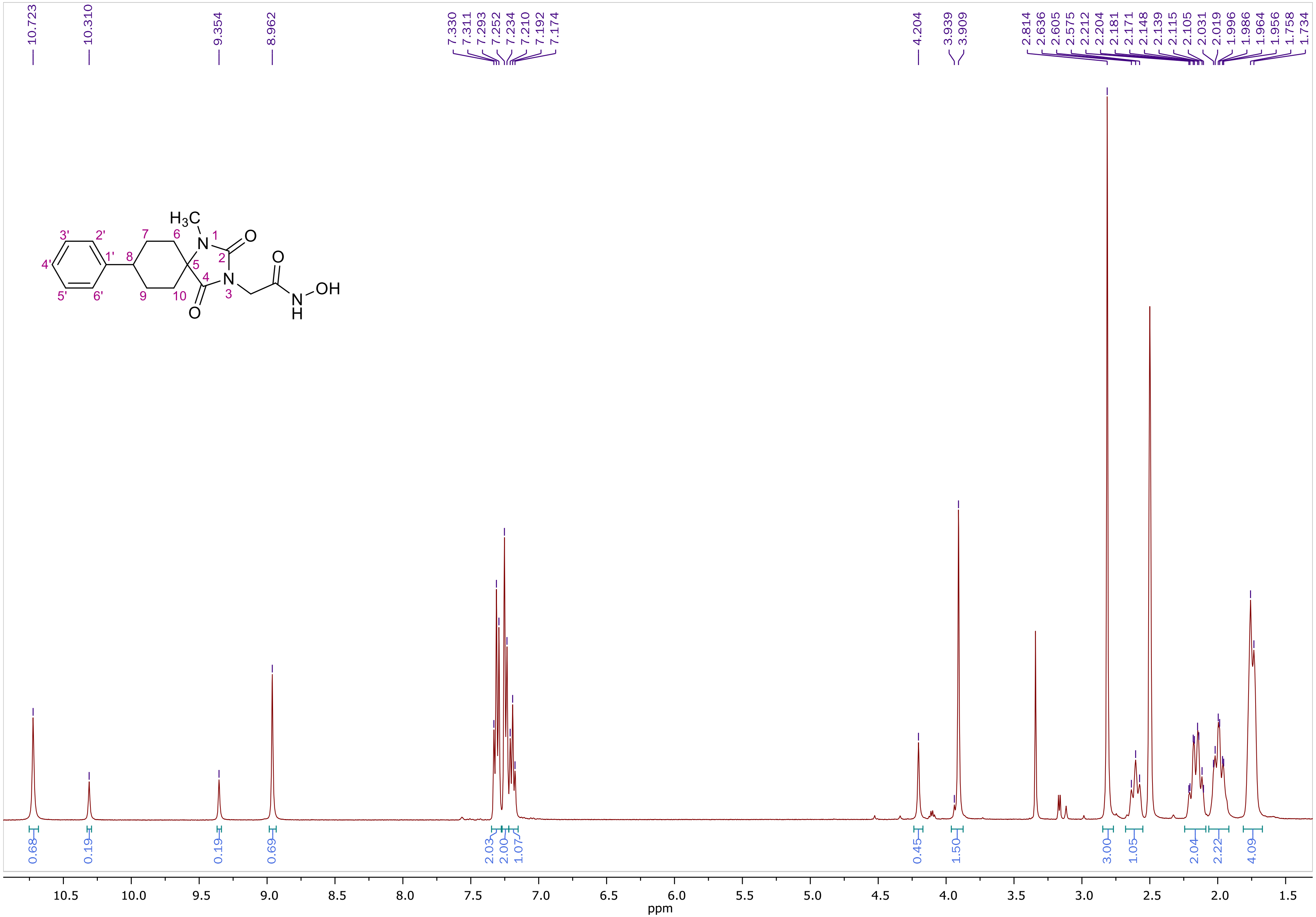

^{13}C NMR of **38** (50.32 MHz, DMSO-d_6)

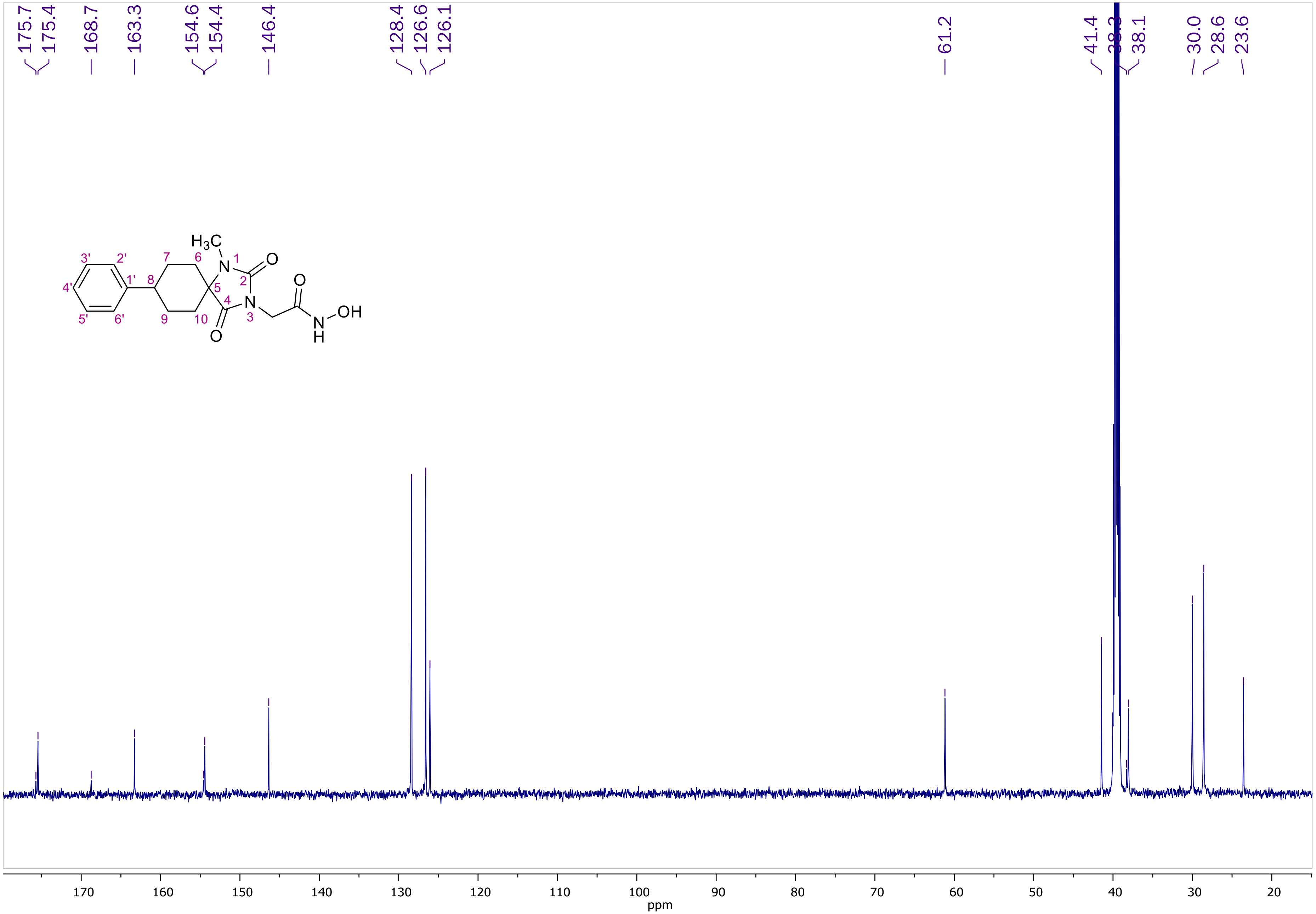

COSY NMR of **38** (400.13 MHz, DMSO-d_6)

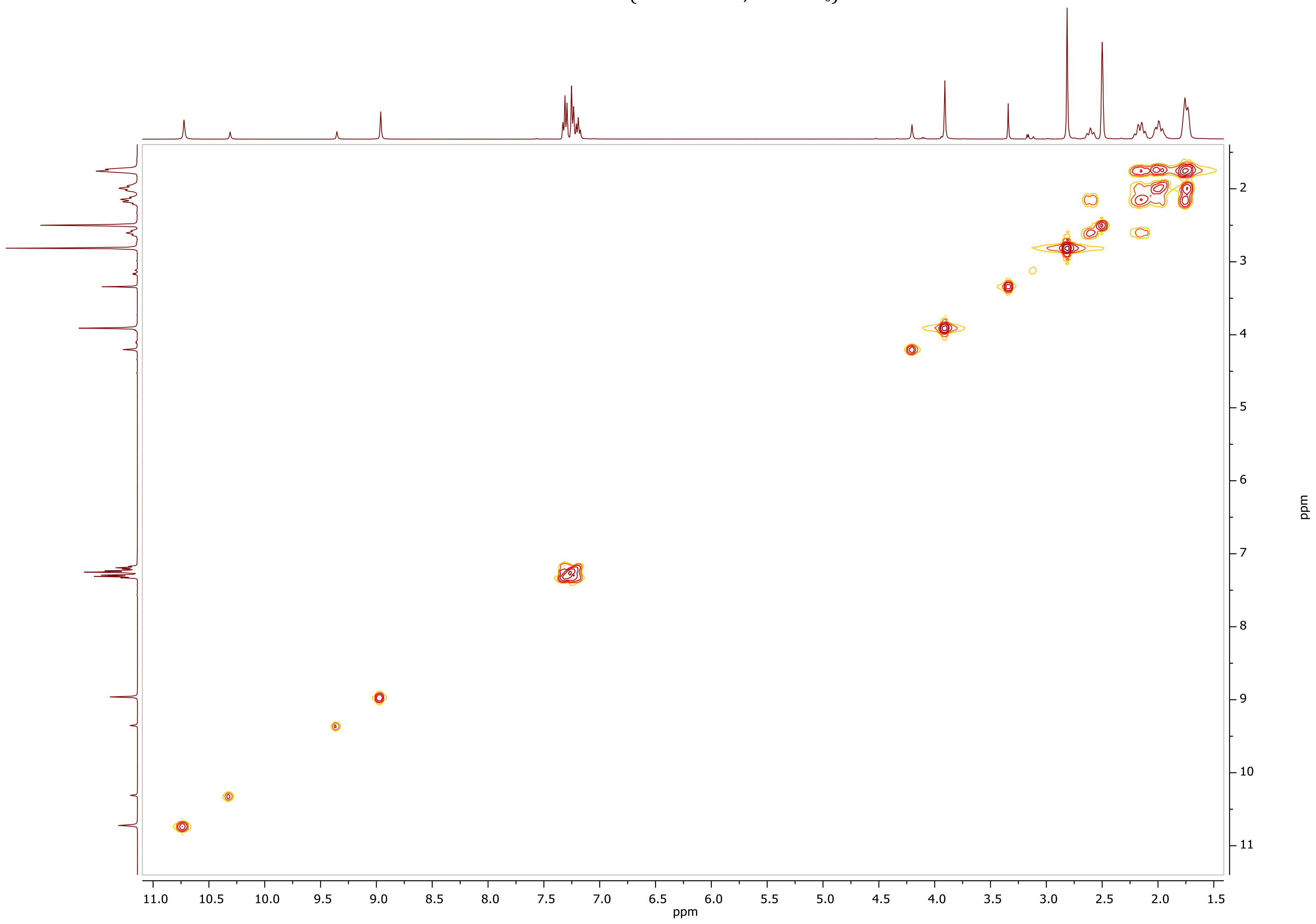

HSQC NMR of **38** (400.13 MHz, DMSO-d_6)

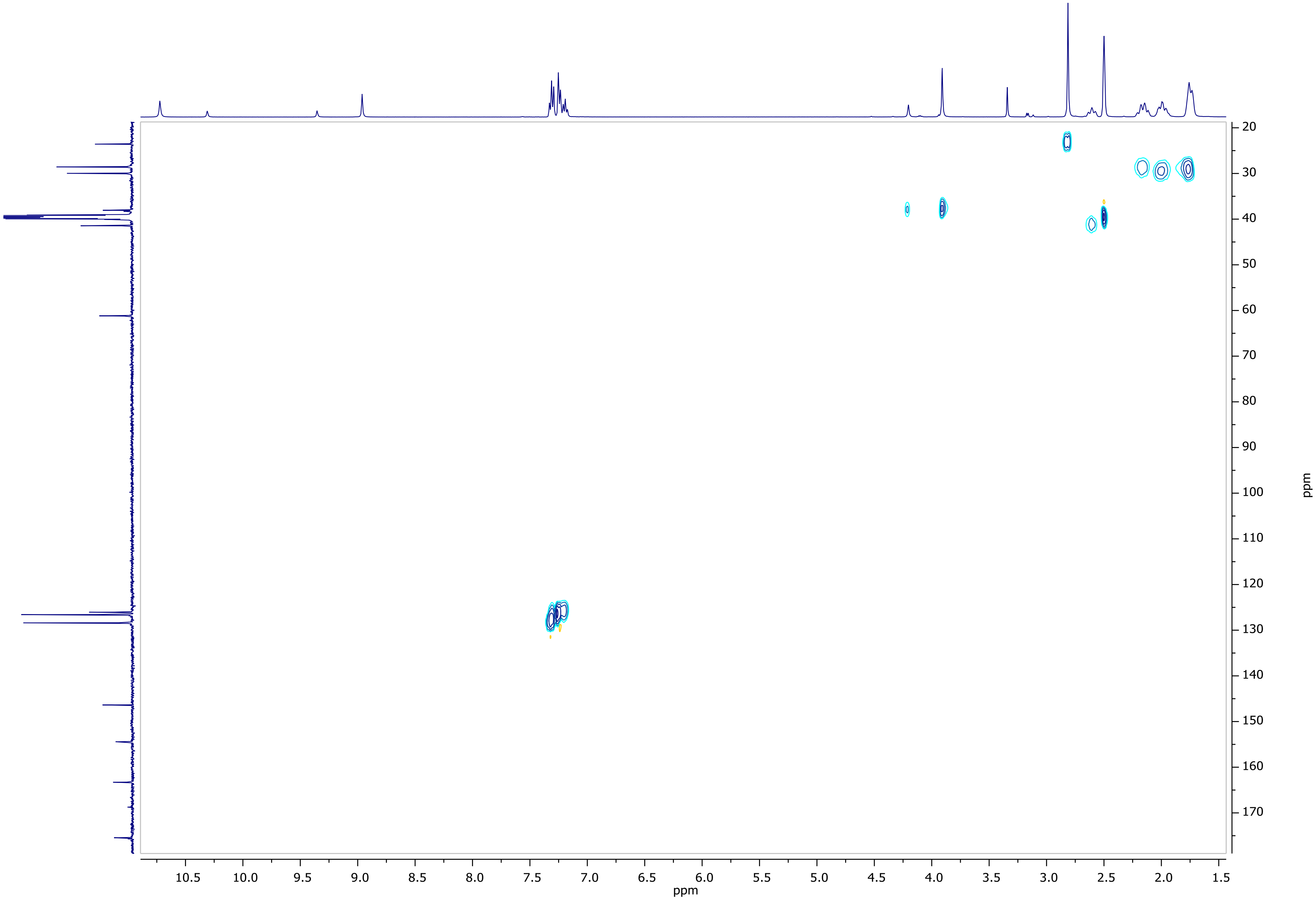

HMBC NMR of **38** (400.13 MHz, DMSO-d_6)

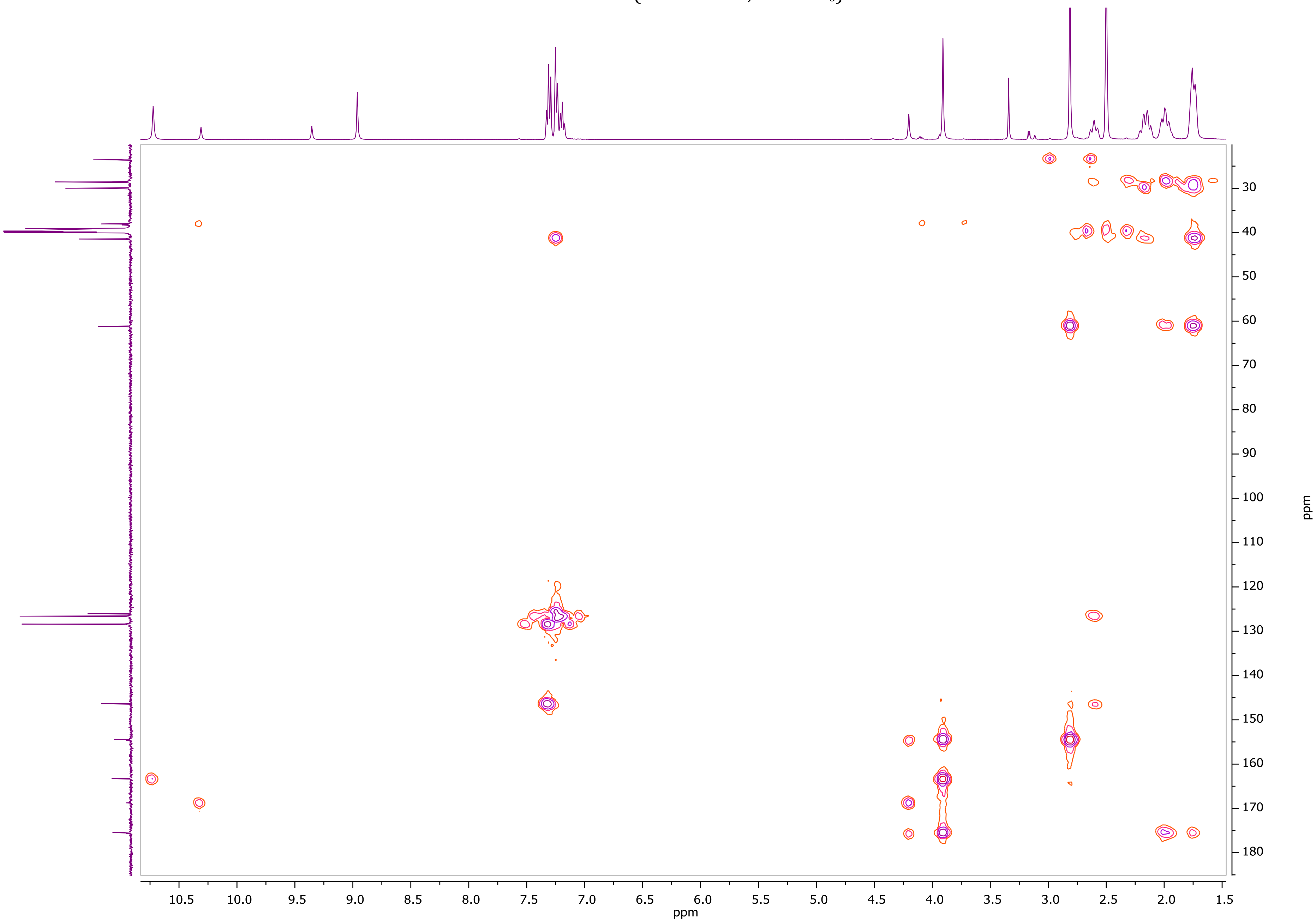

^{1}H NMR of **39** (600.11 MHz, DMSO-d_6)

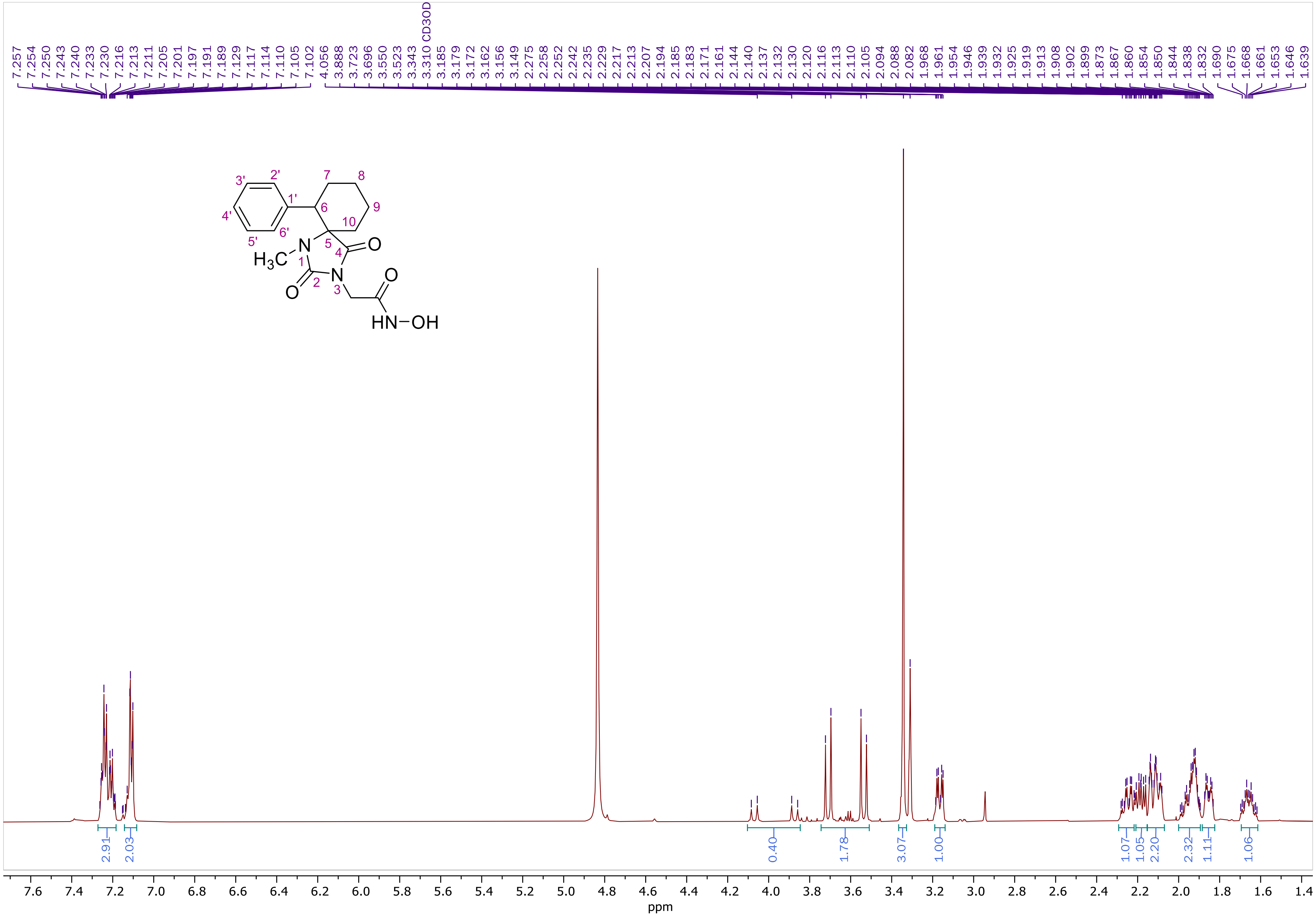

^{13}C NMR of **39** (150.9 MHz, DMSO-d_6)

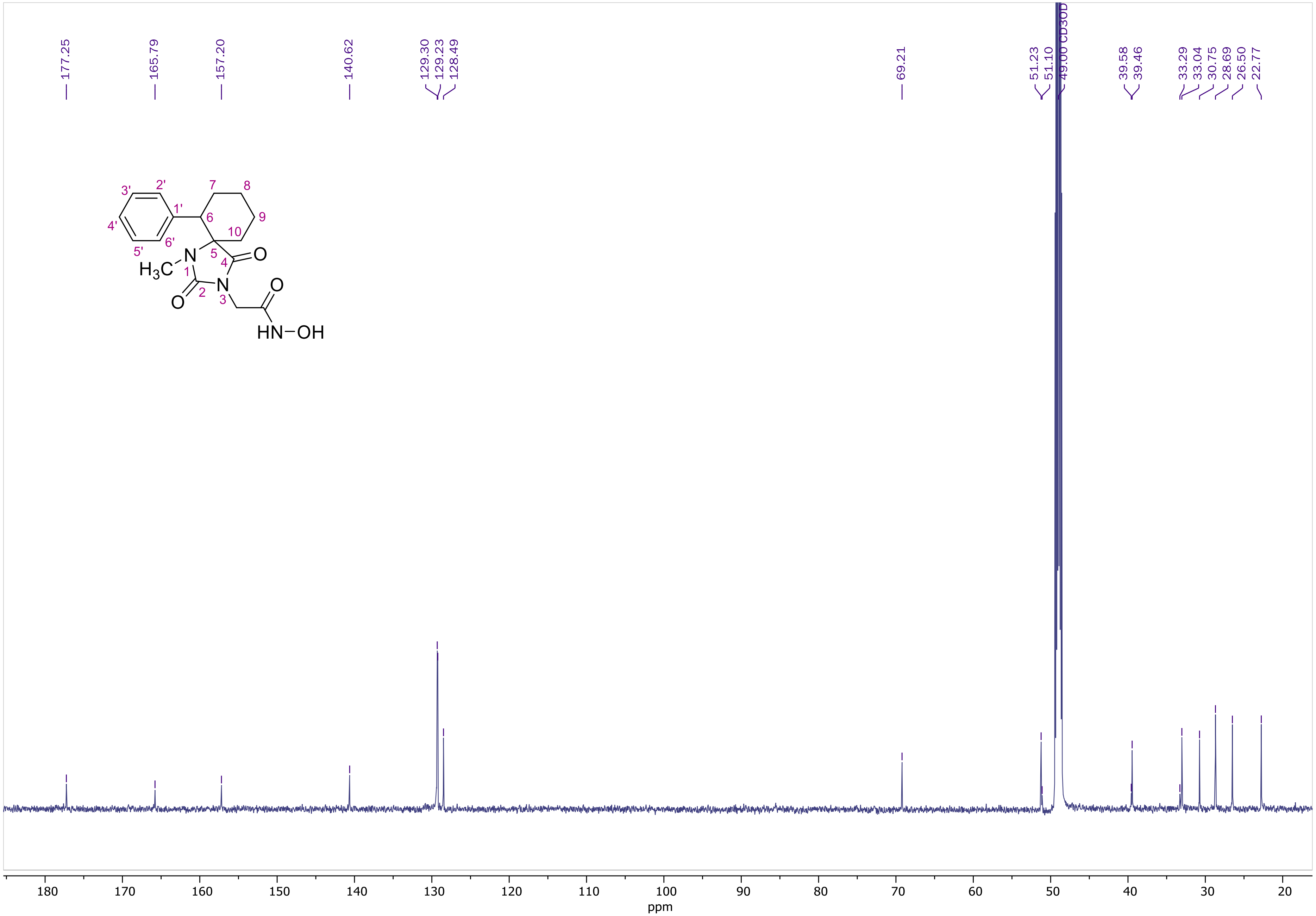

COSY NMR of **39** (400.13 MHz, DMSO-d_6)

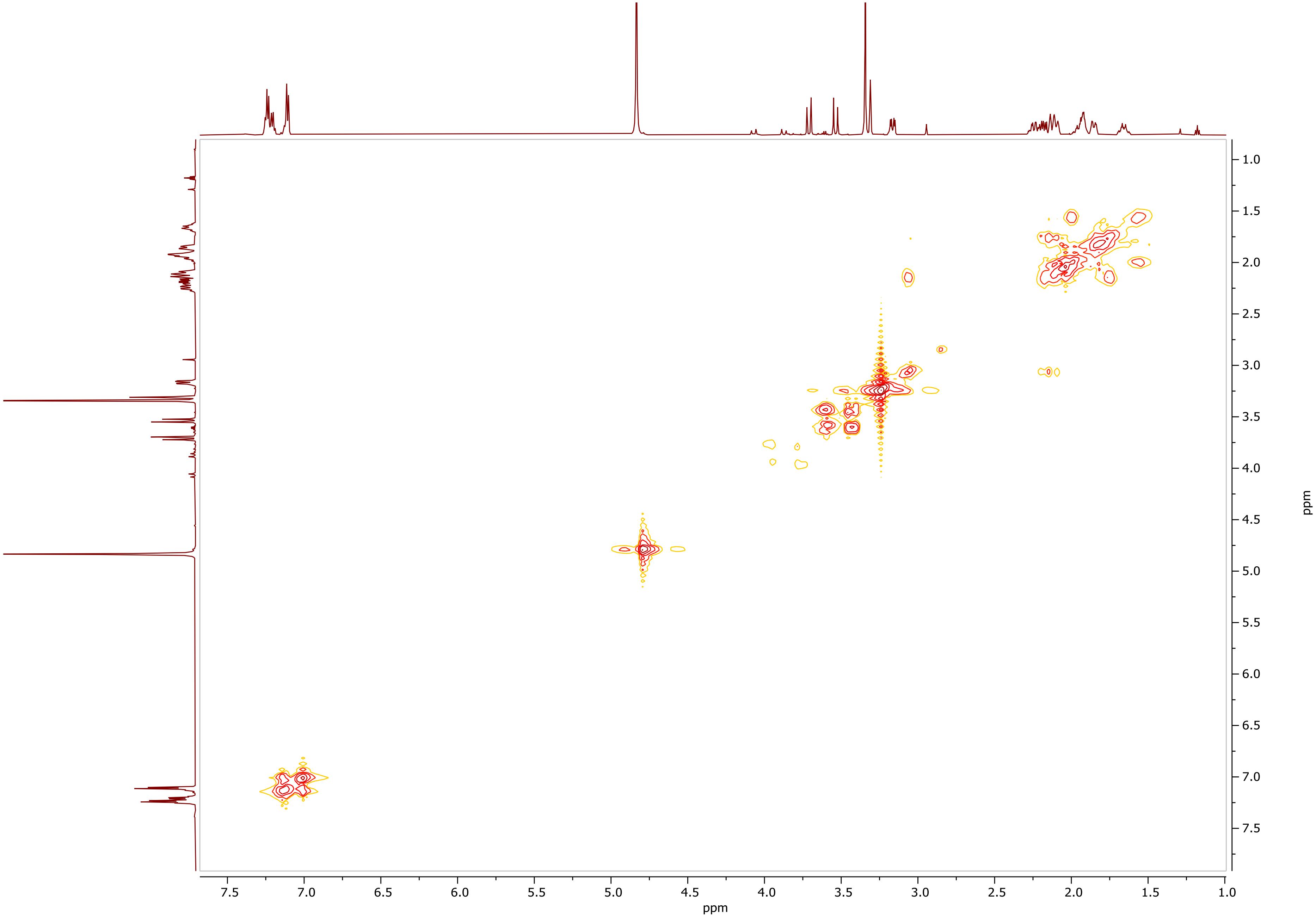

HSQC-DEPT NMR of **39** (600.11 MHz, DMSO-d_6)

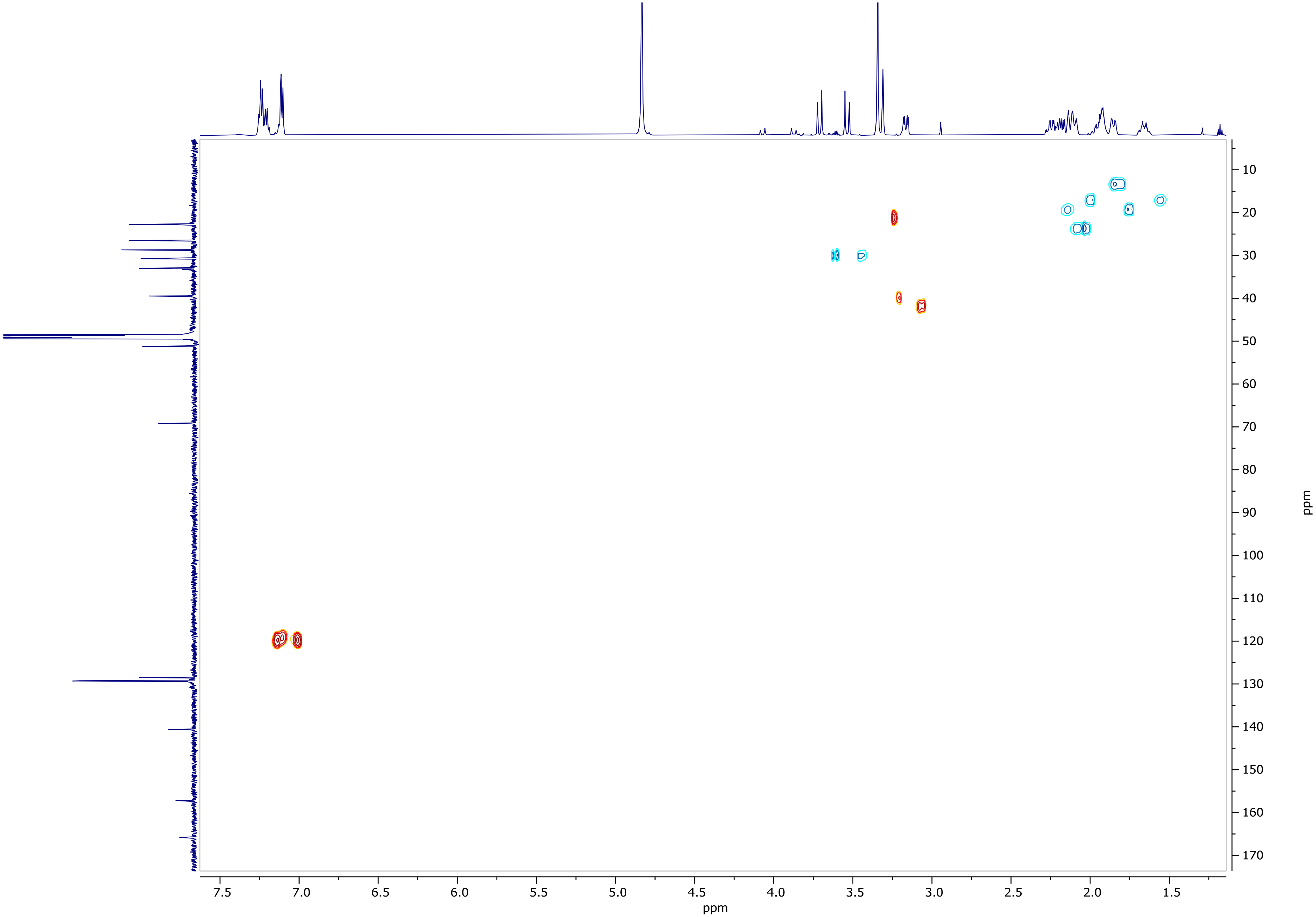

HMBC NMR of **39** (600.11 MHz, DMSO-d_6)

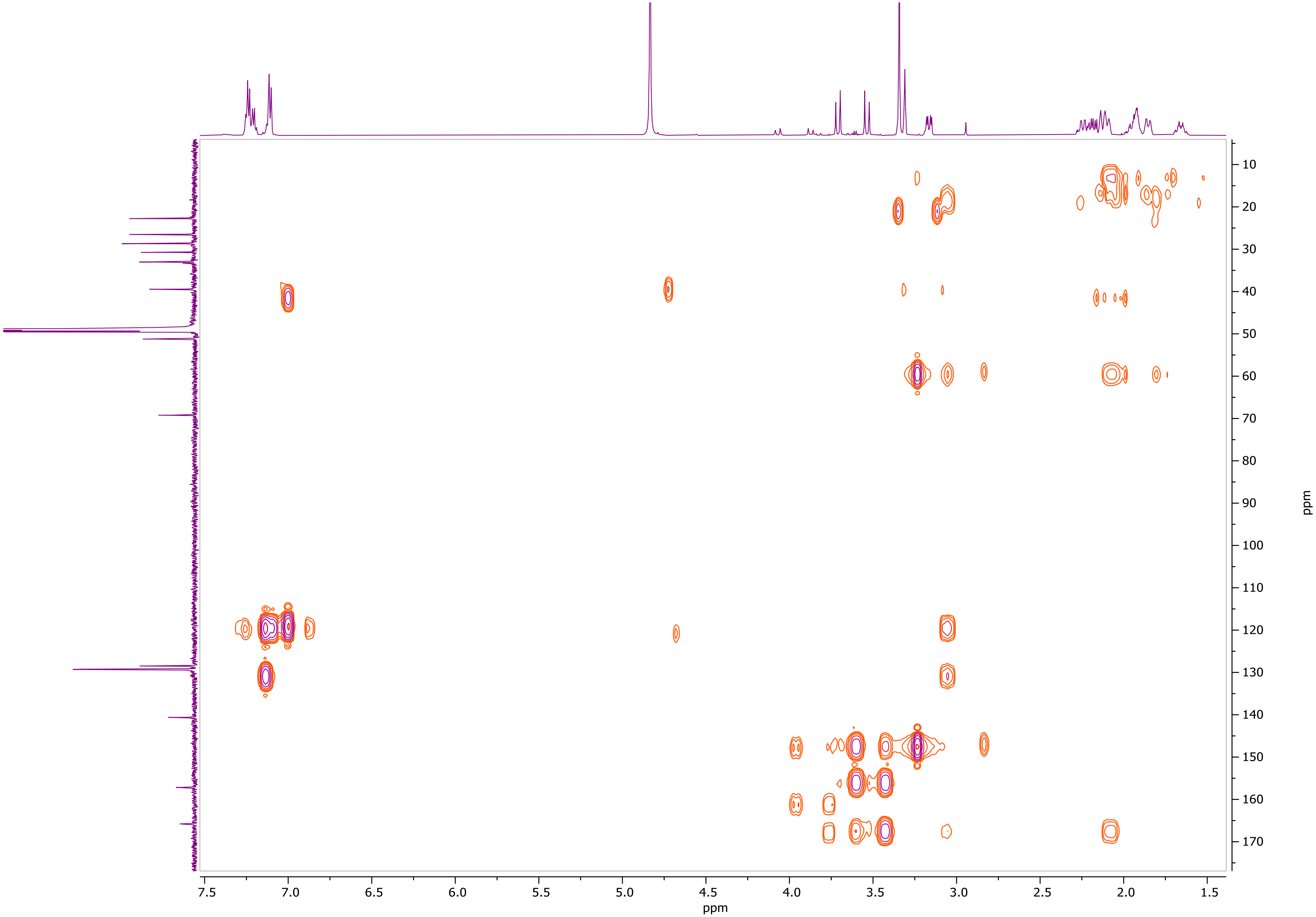

^{1}H NMR of **40** (600.11 MHz, DMSO-d_6)

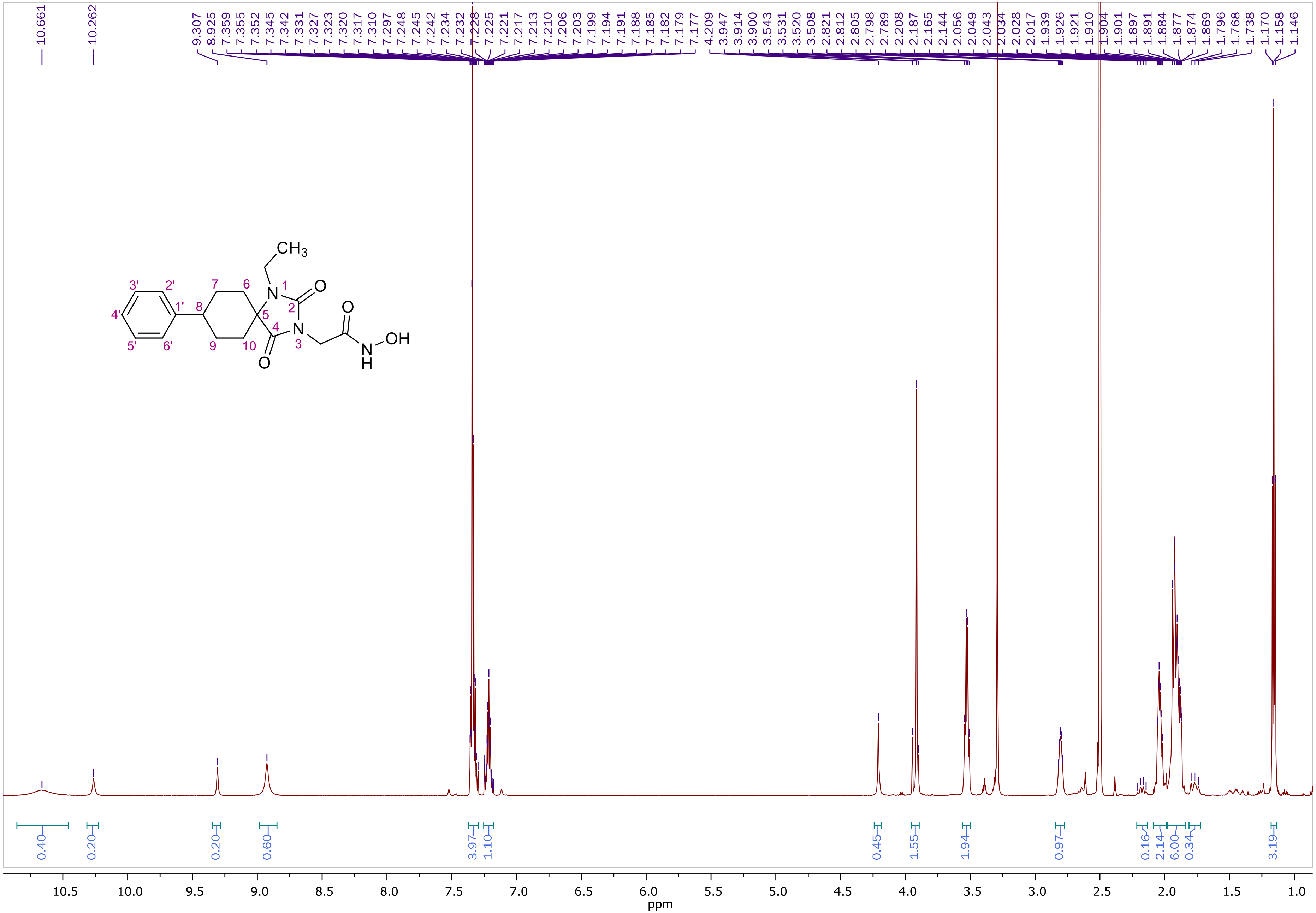

^{13}C NMR of **40** (150.9 MHz, DMSO-d_6)

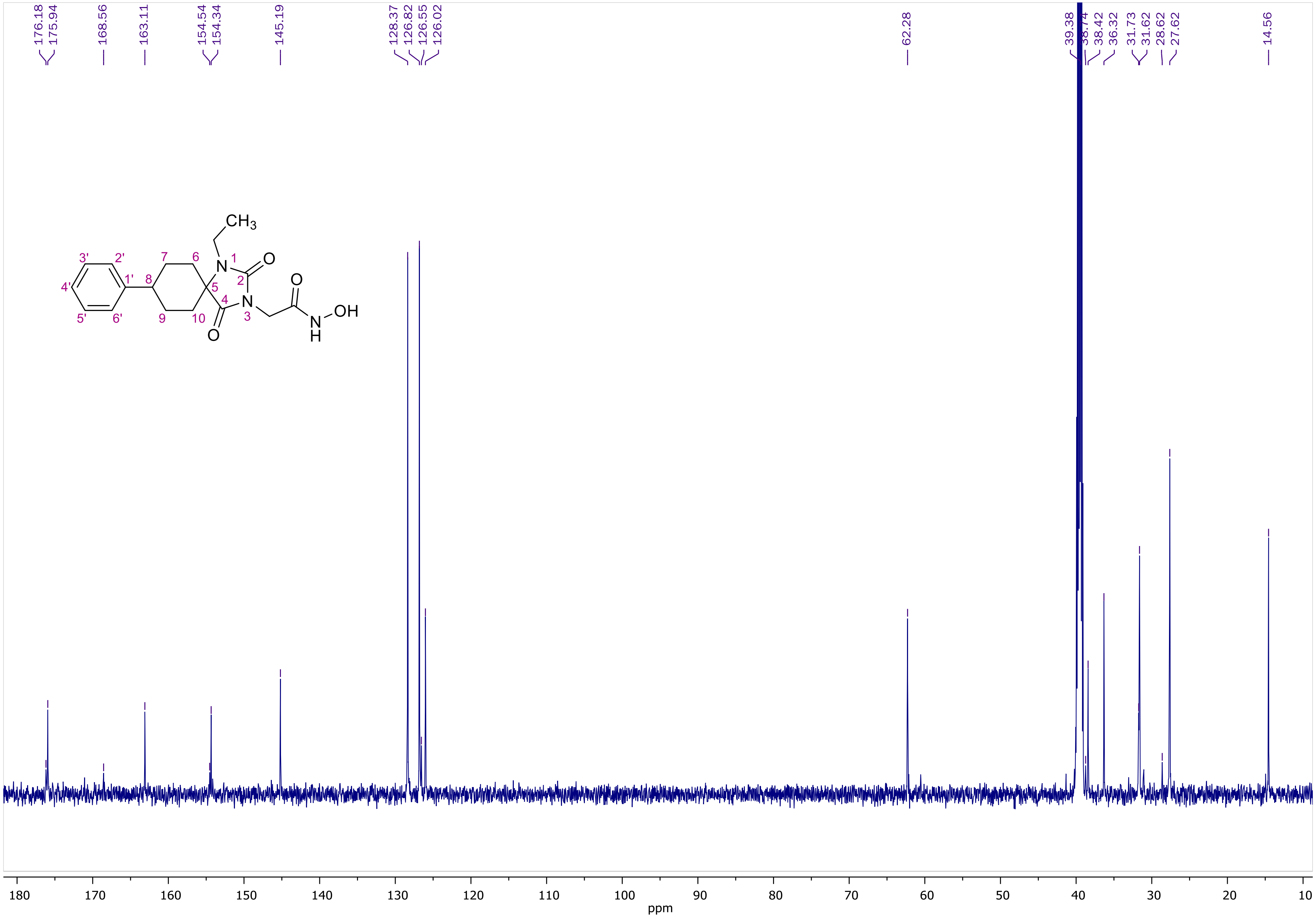

COSY NMR of **40** (600.11 MHz, DMSO-d_6)

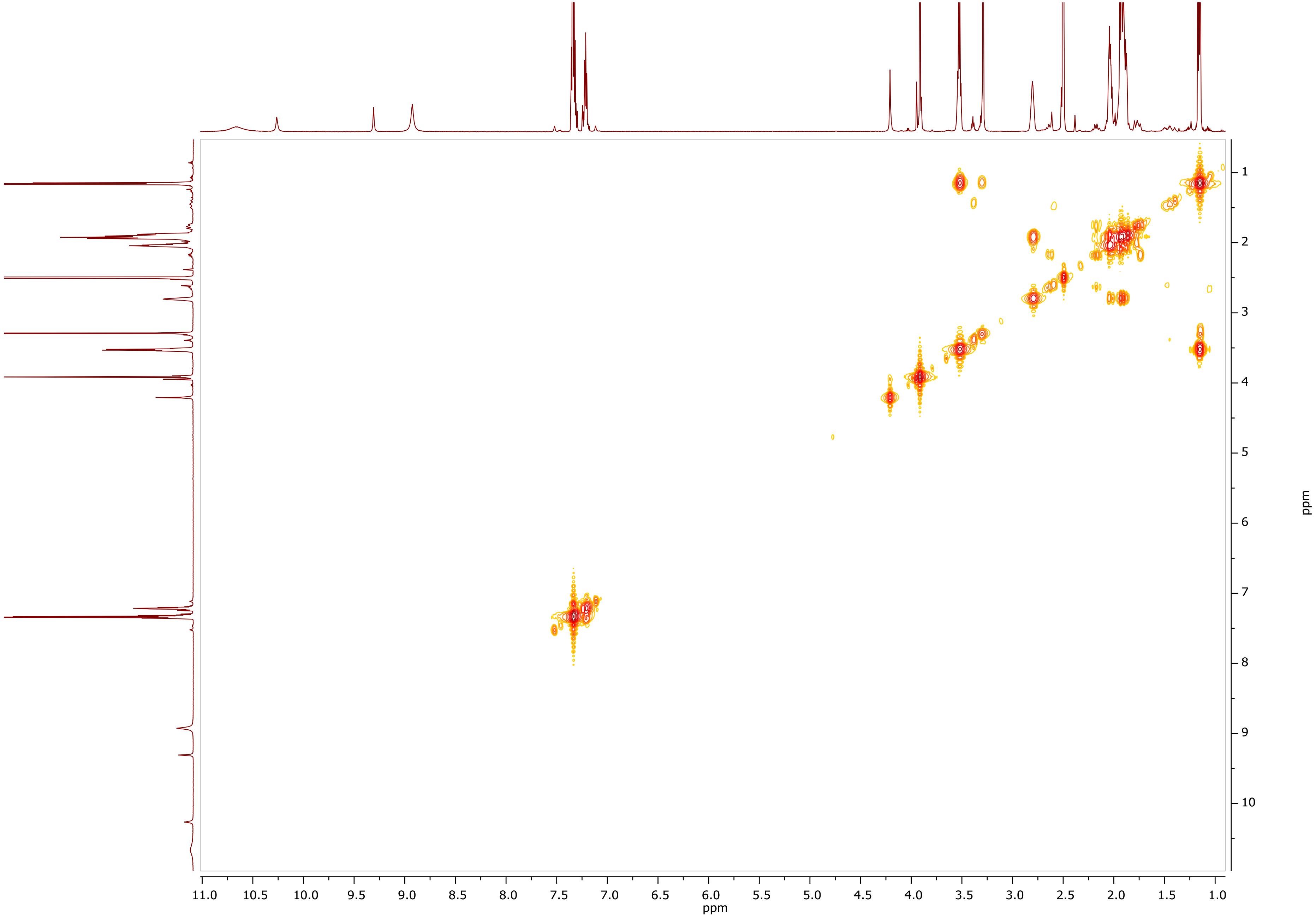

HSQC-DEPT NMR of **40** (600.11 MHz, DMSO-d_6)

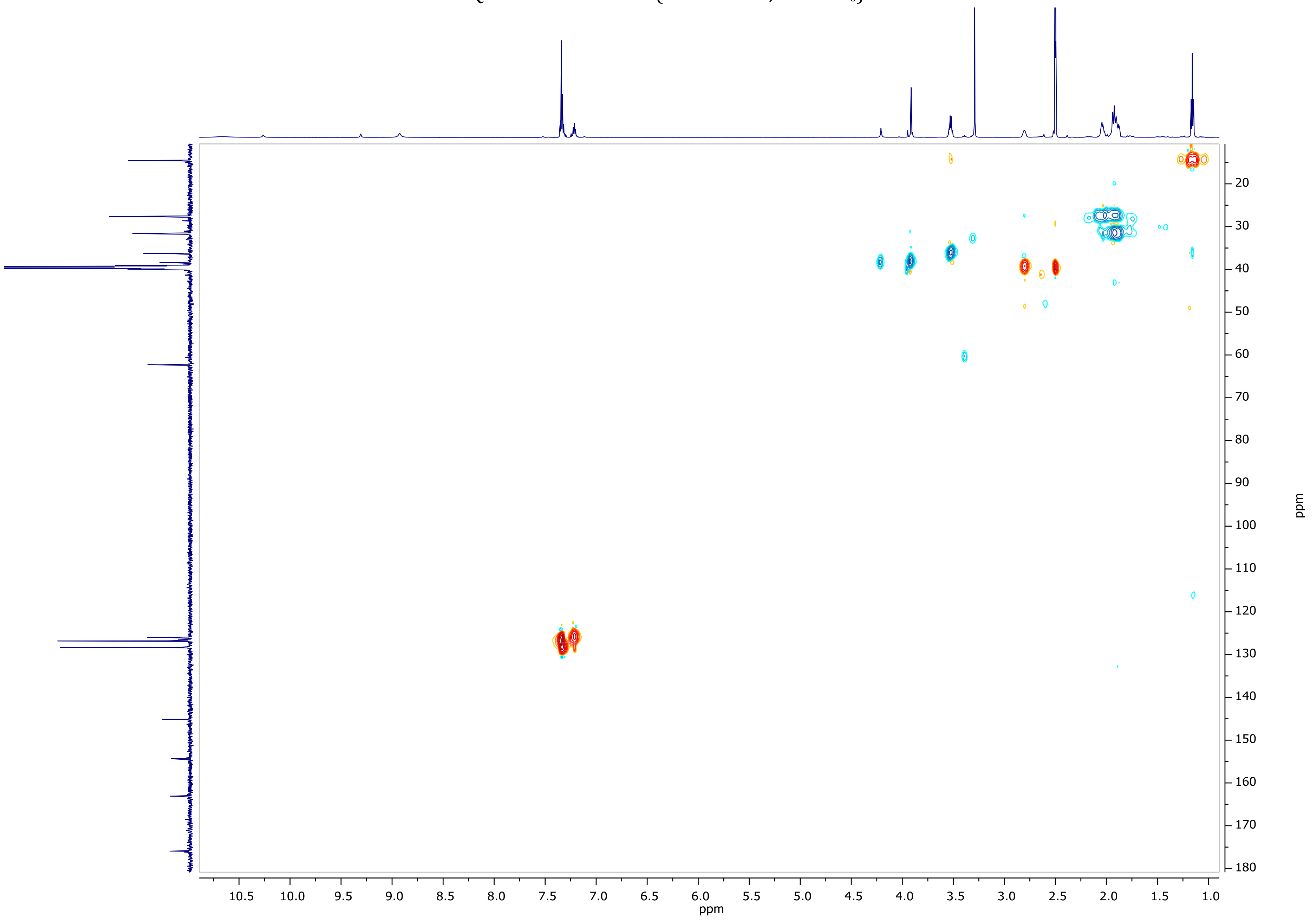

HMBC NMR of **40** (600.11 MHz, DMSO-d_6)

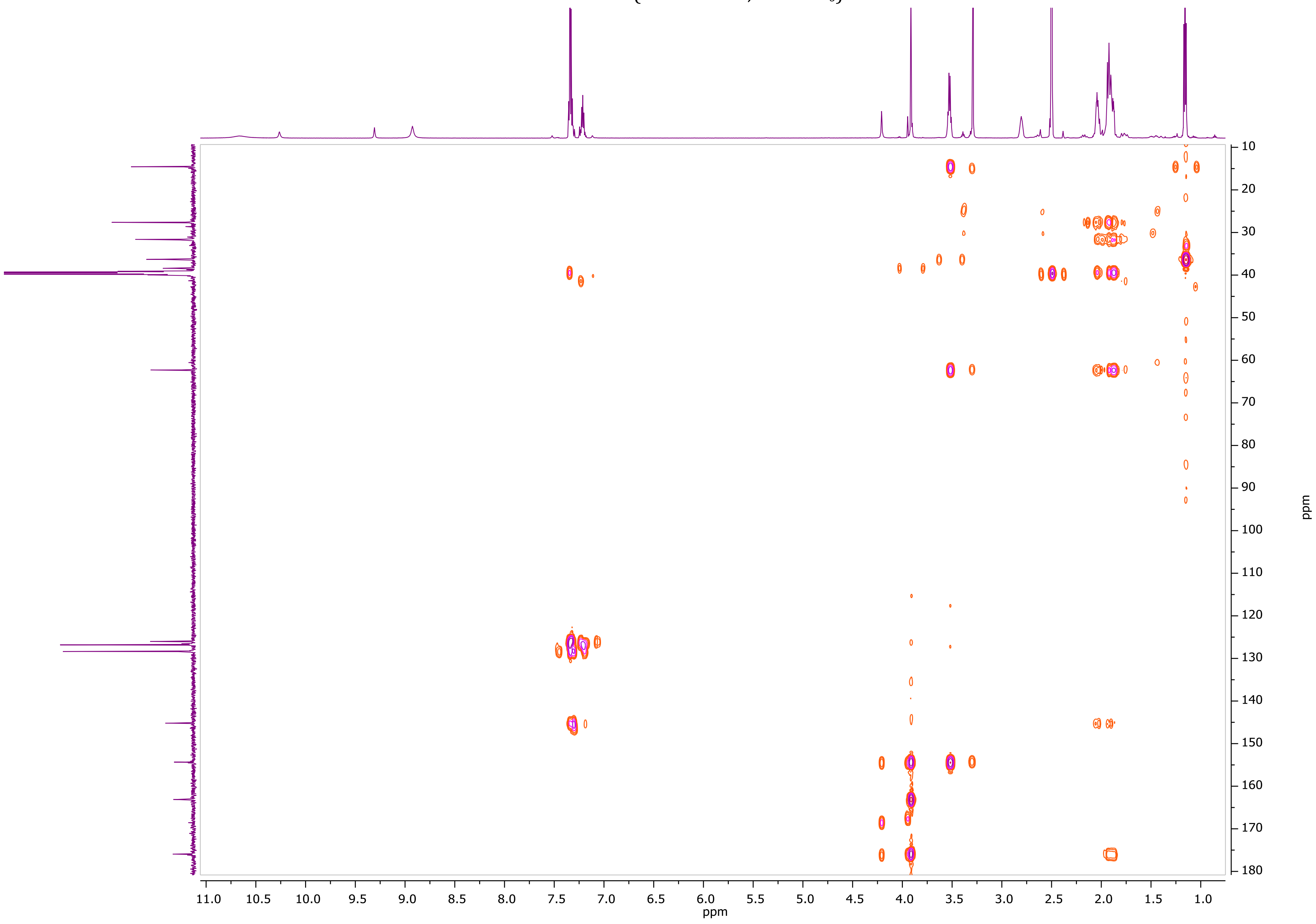

^{1}H NMR of **41** (400.13 MHz, Acetone-d_6)

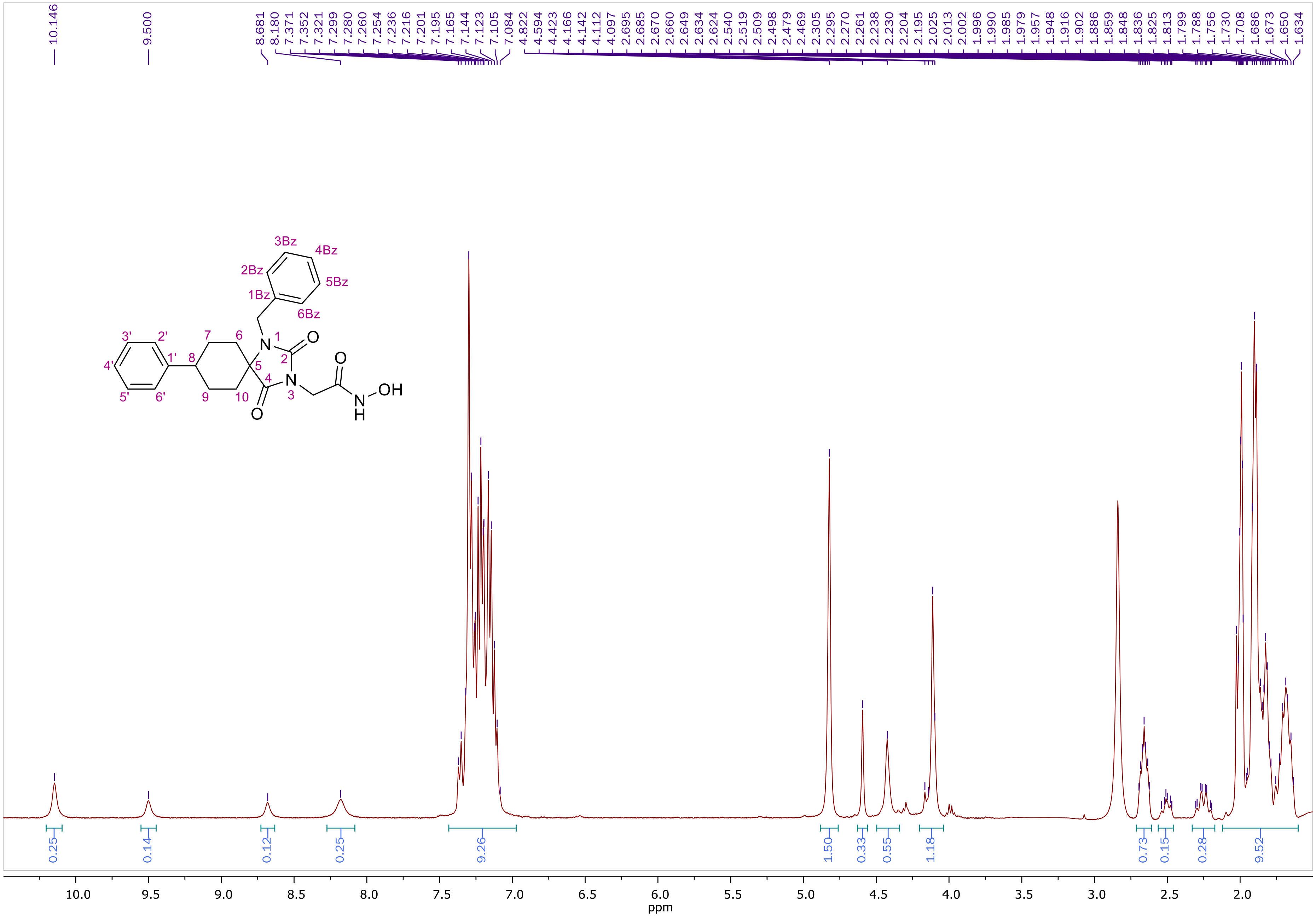

^{13}C NMR of **41** (50.32 MHz, Acetone-d_6)

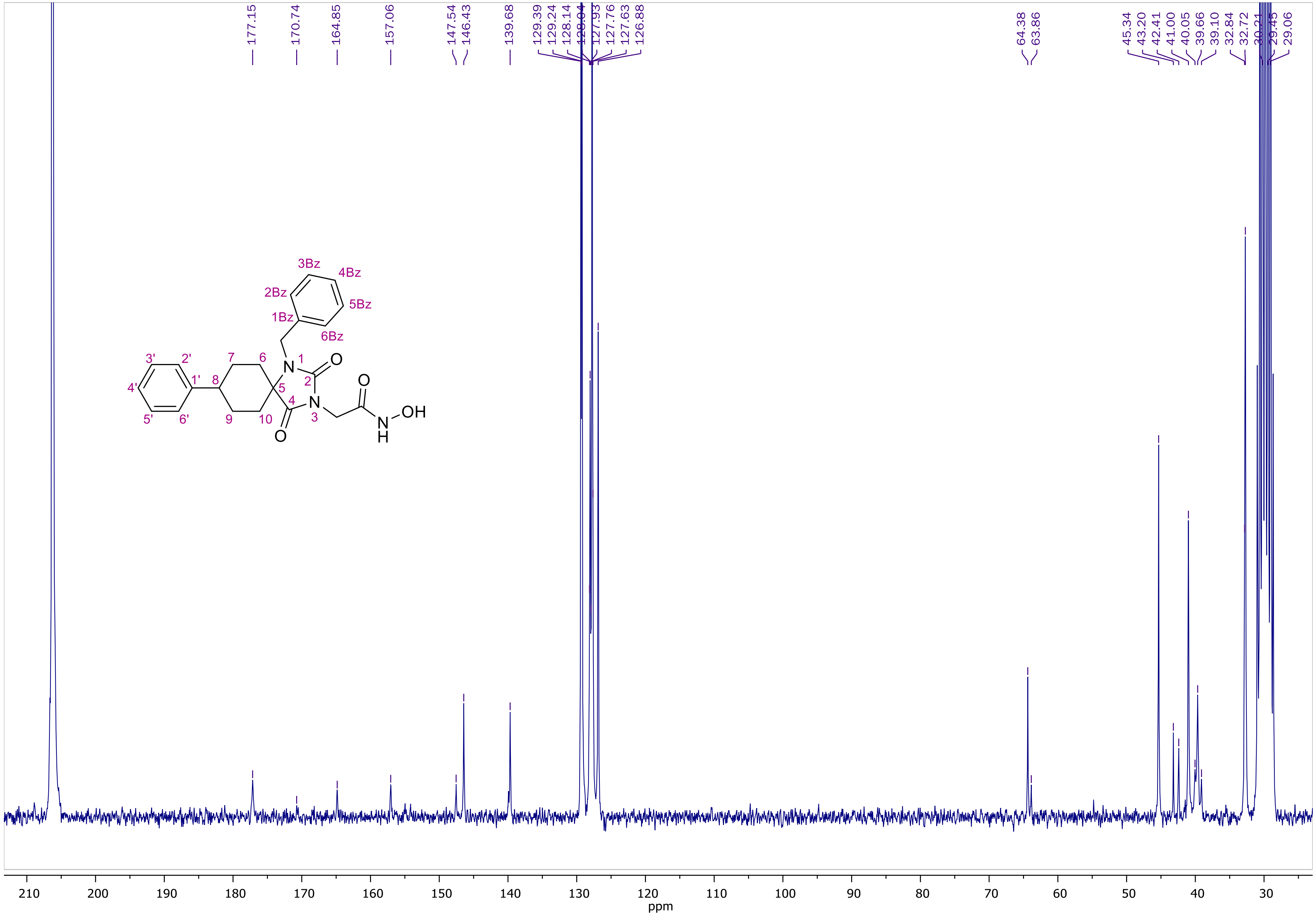

COSY NMR of **41** (400.13 MHz, Acetone-d_6)

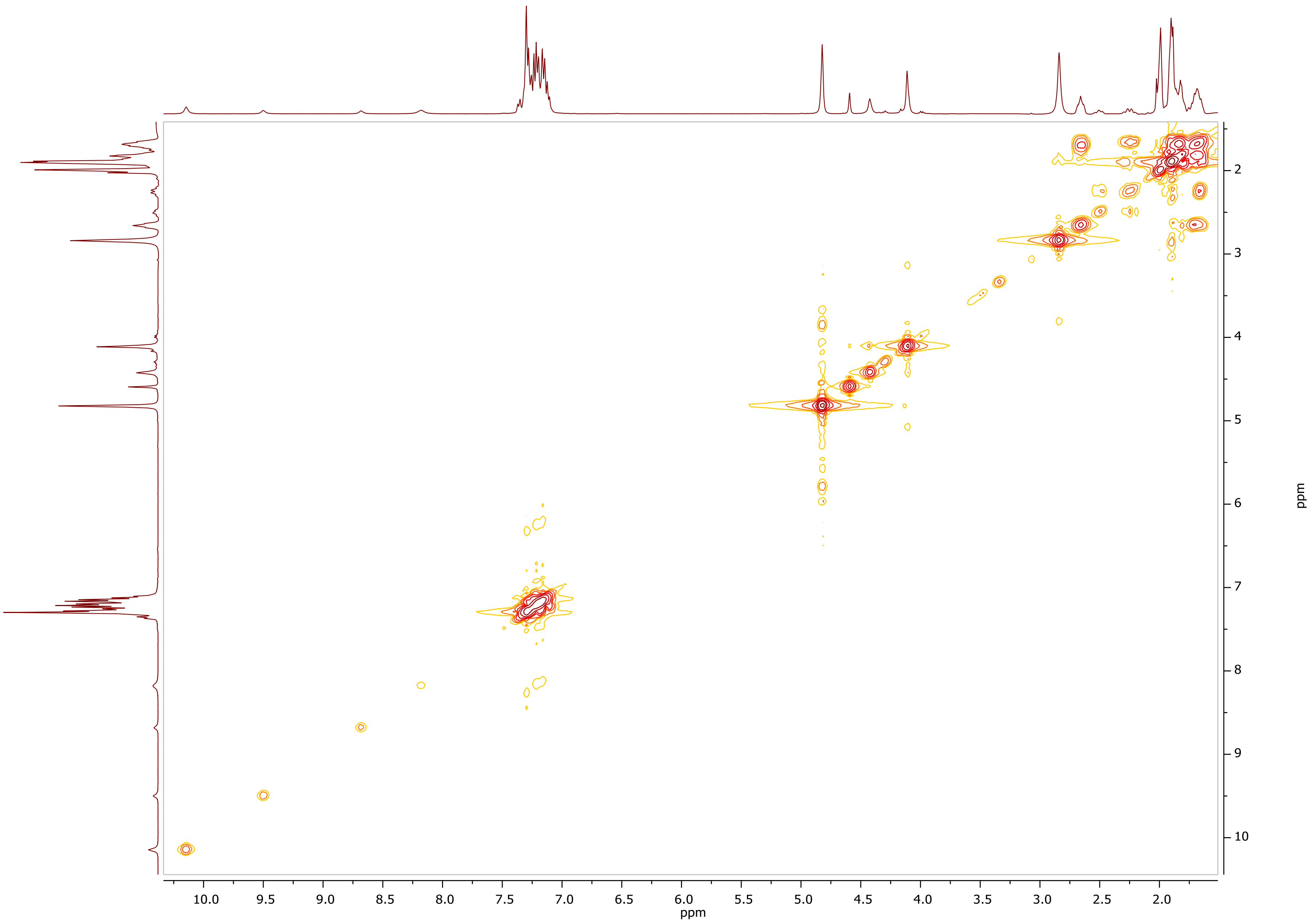

HSQC NMR of **41** (400.13 MHz, Acetone-d_6)

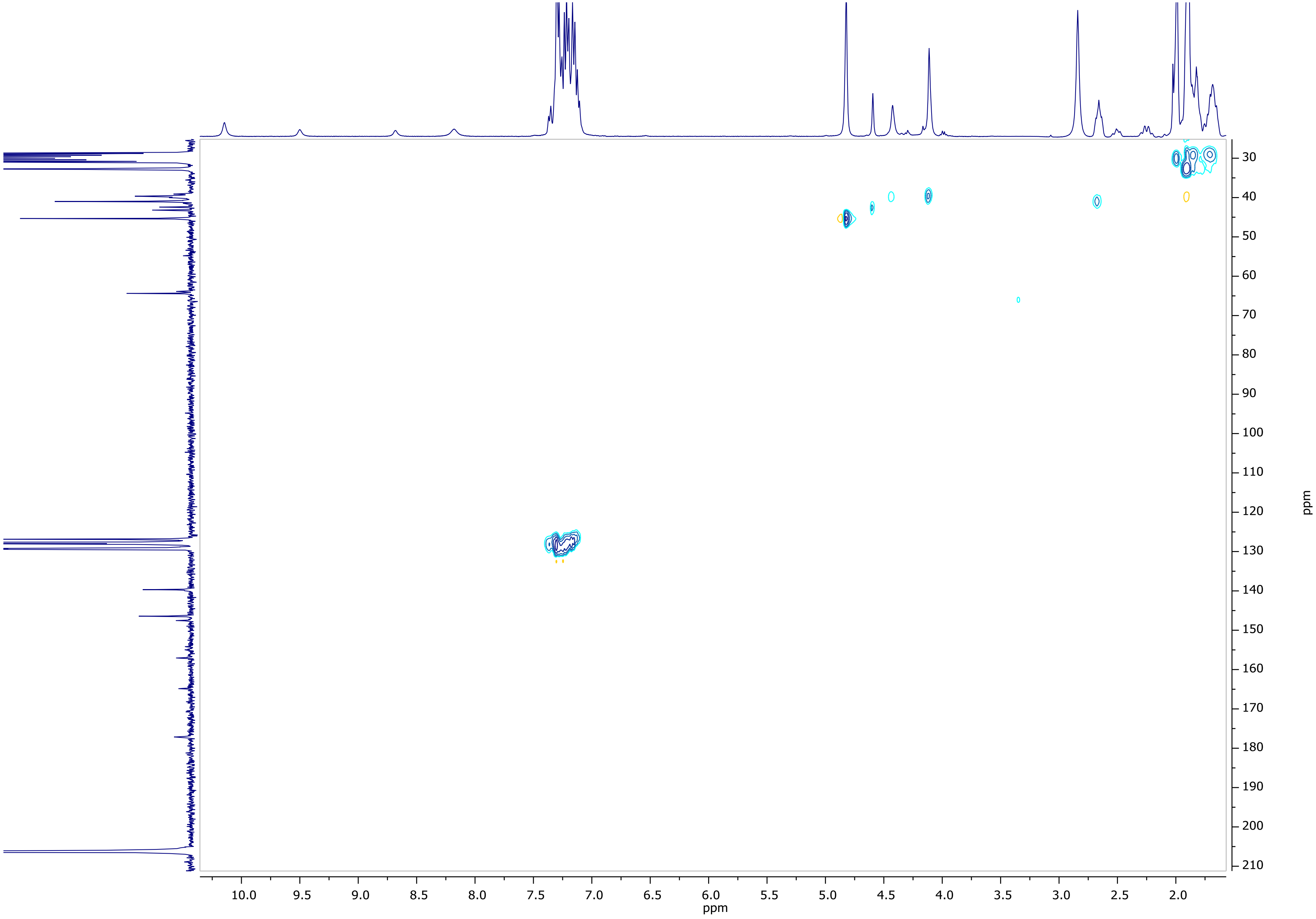

HMBC NMR of **41** (400.13 MHz, Acetone-d_6)

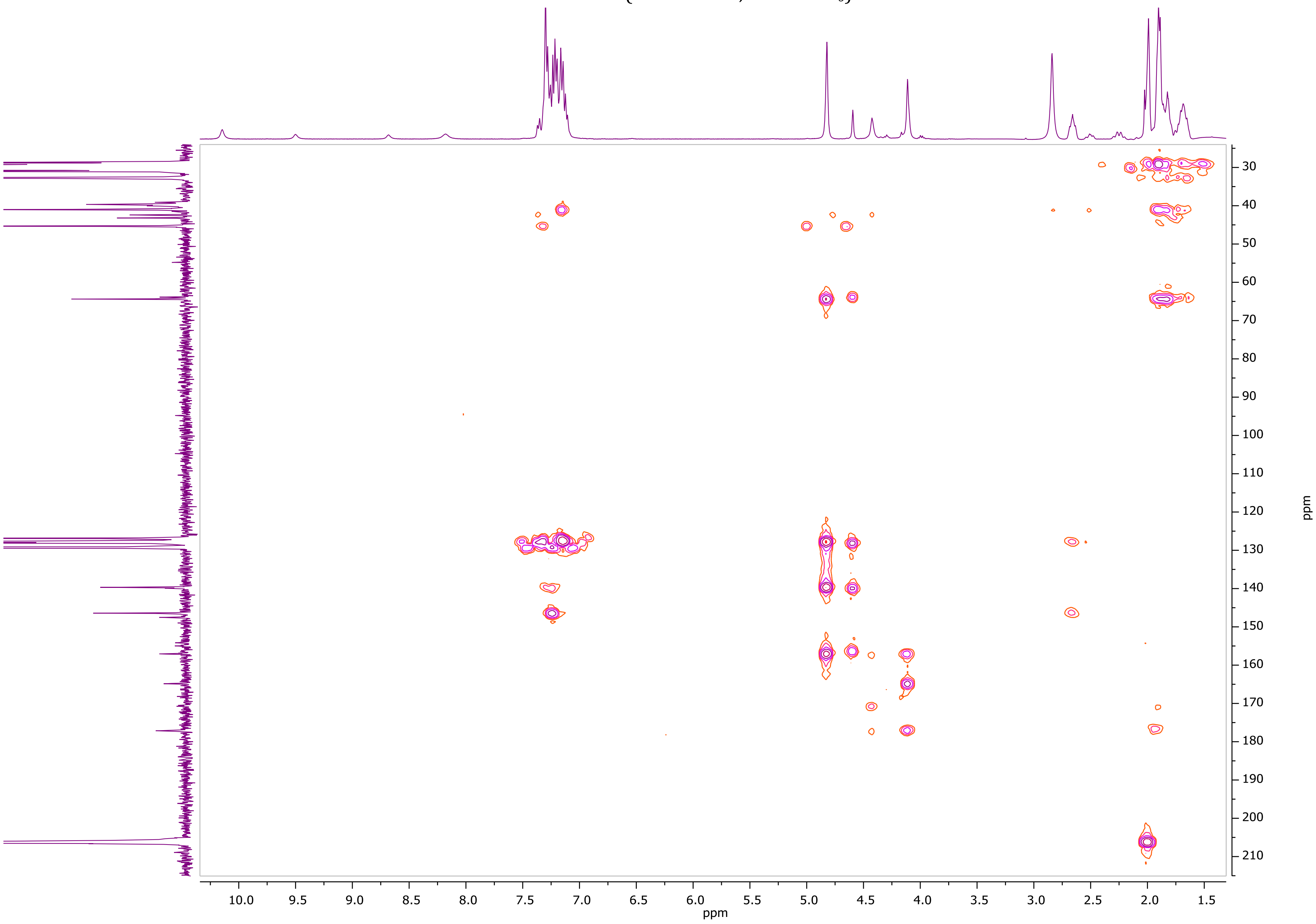

^{1}H NMR of **42** (600.11 MHz, DMSO-d_6)

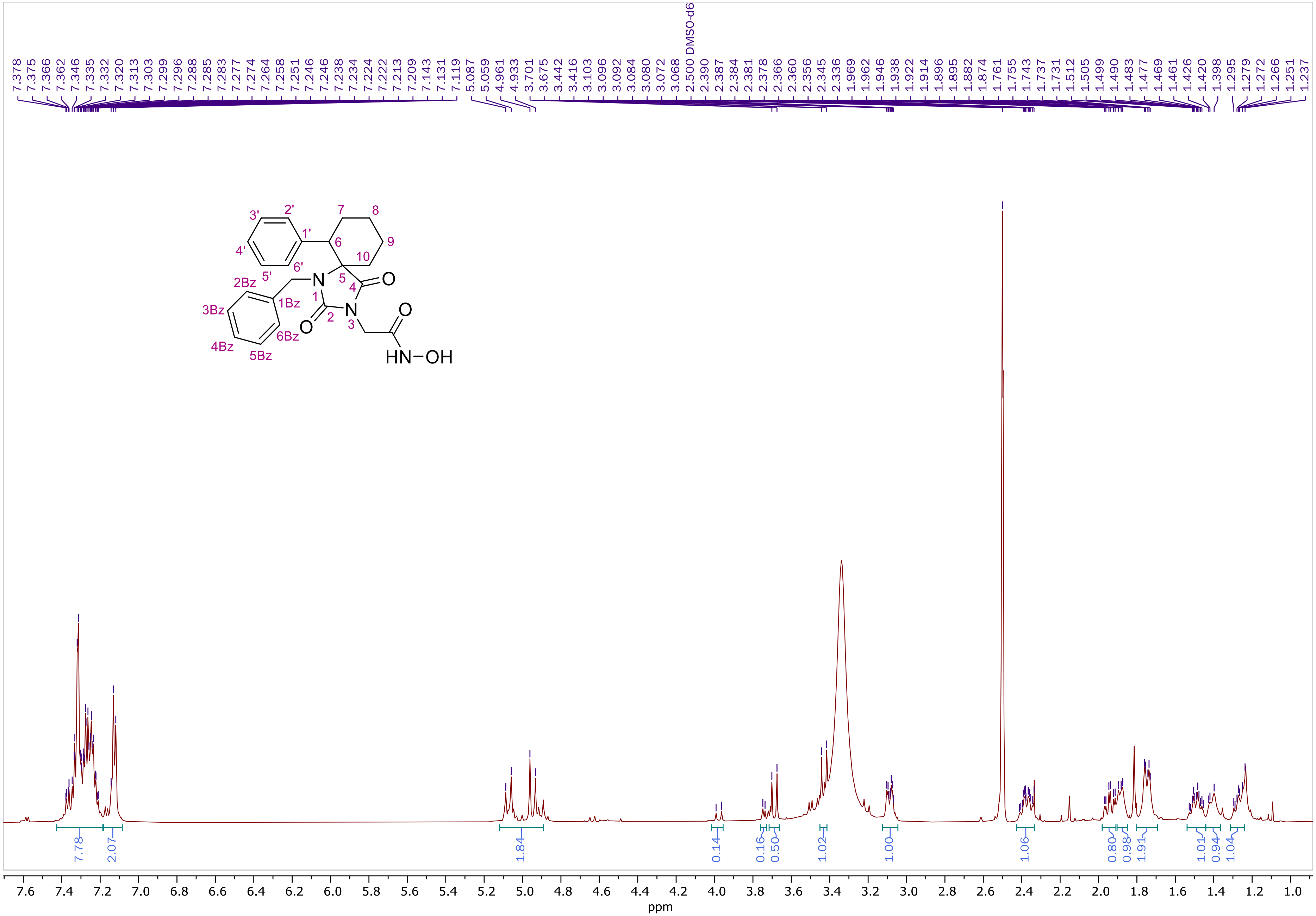

^{13}C NMR of **42** (100.61 MHz, DMSO-d_6)

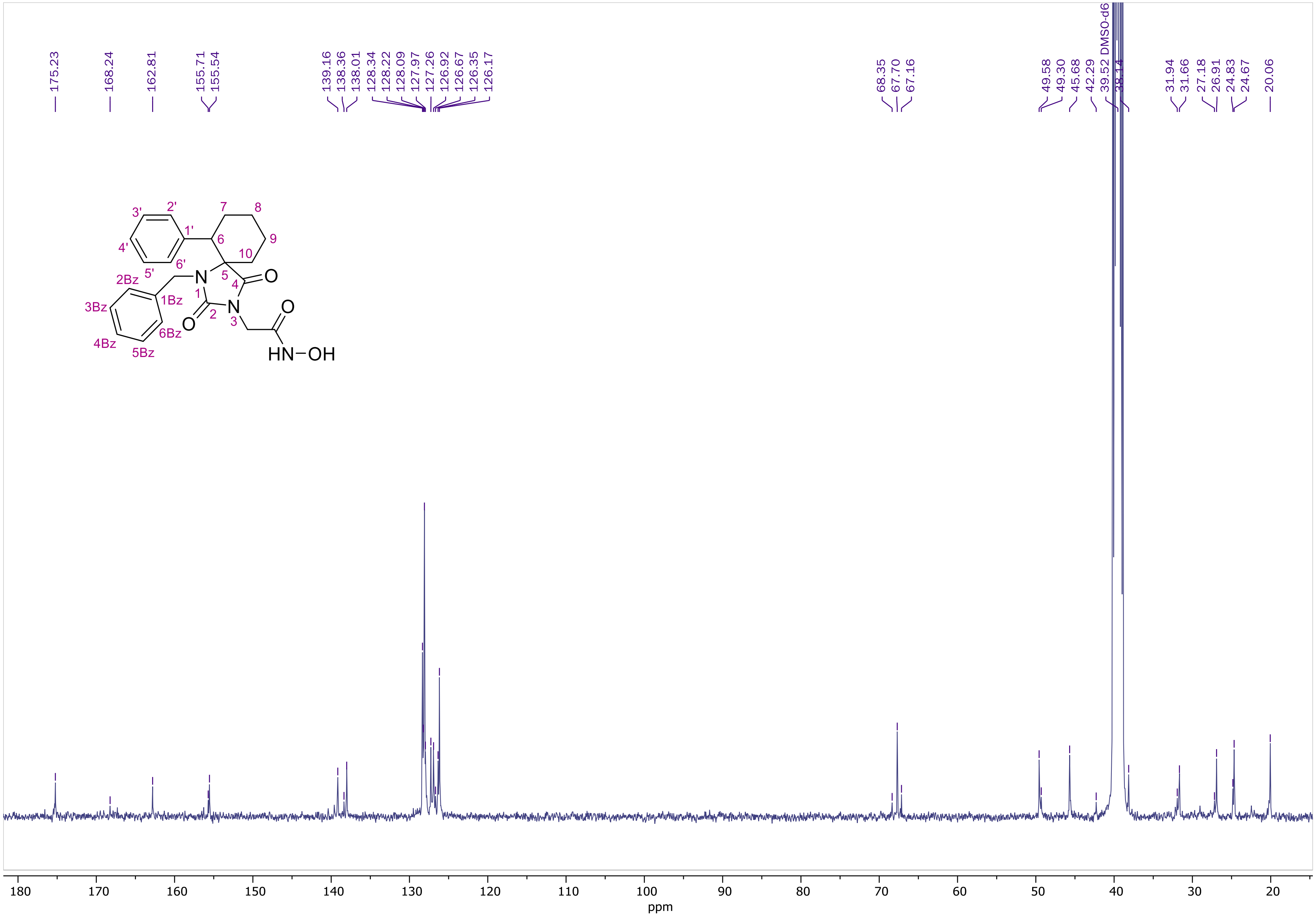

COSY NMR of **42** (400.13 MHz, DMSO-d_6)

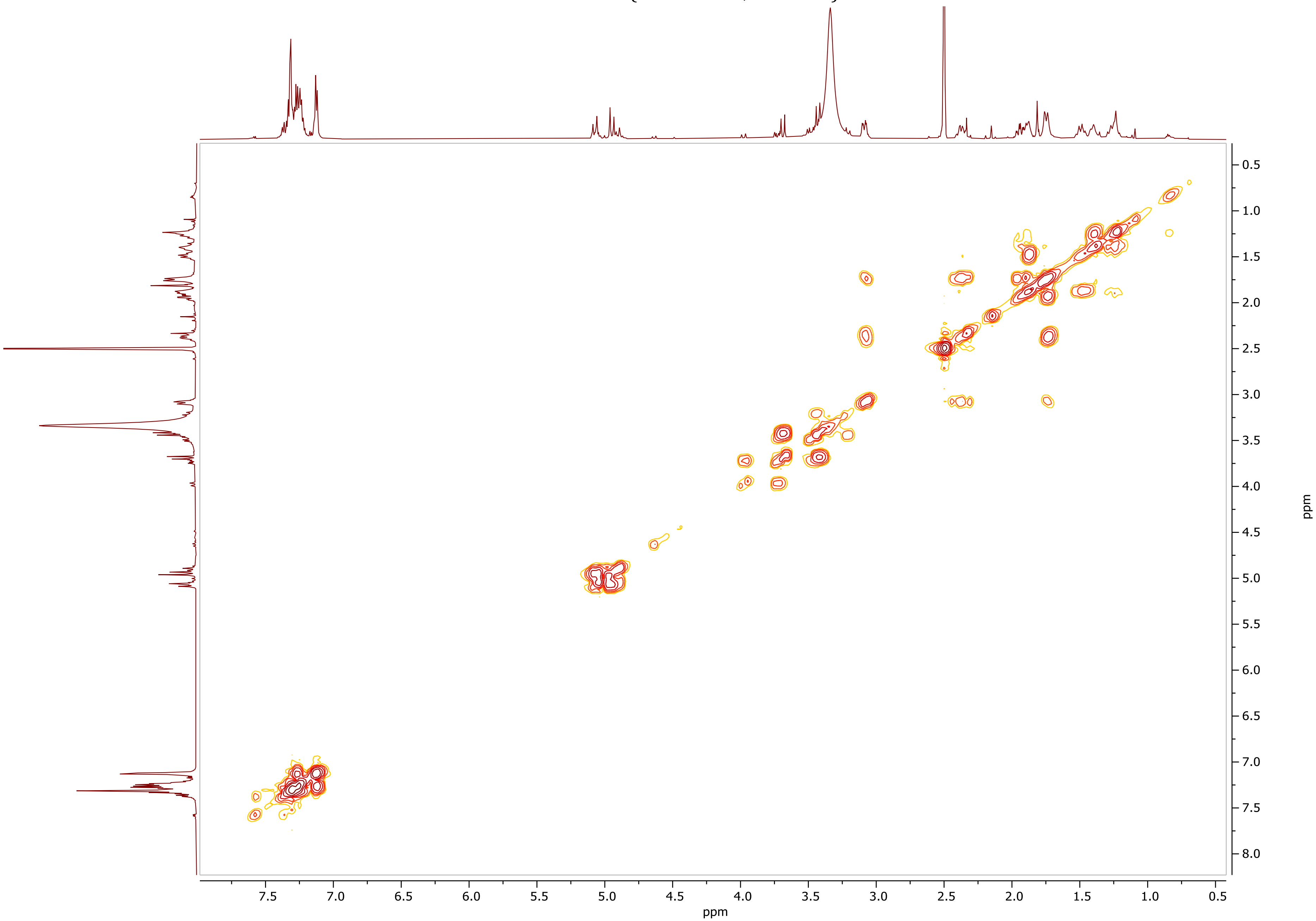

HSQC-DEPT NMR of **42** (200 MHz, DMSO-d_6)

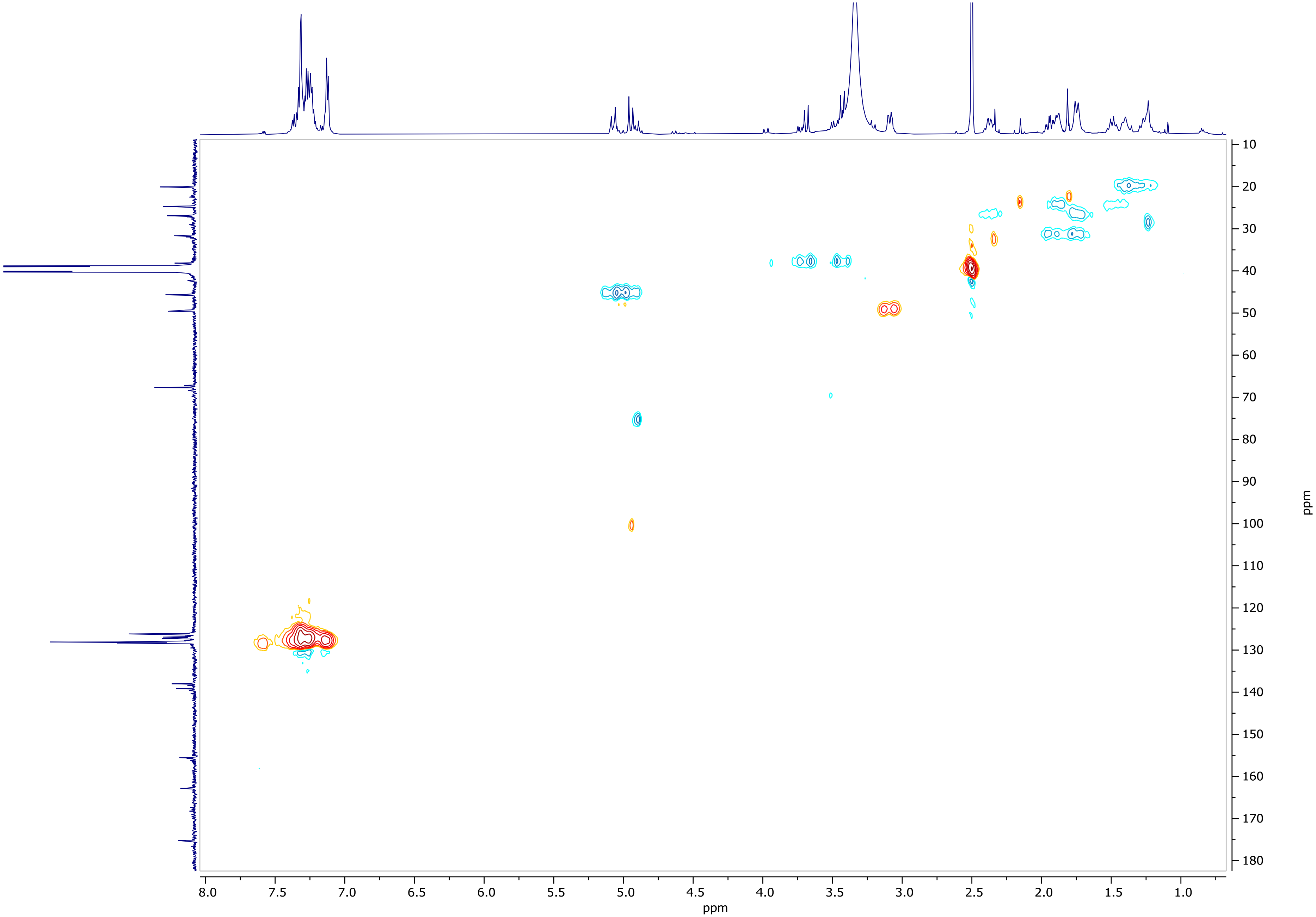

HMBC NMR of **42** (600.11 MHz, DMSO-d_6)

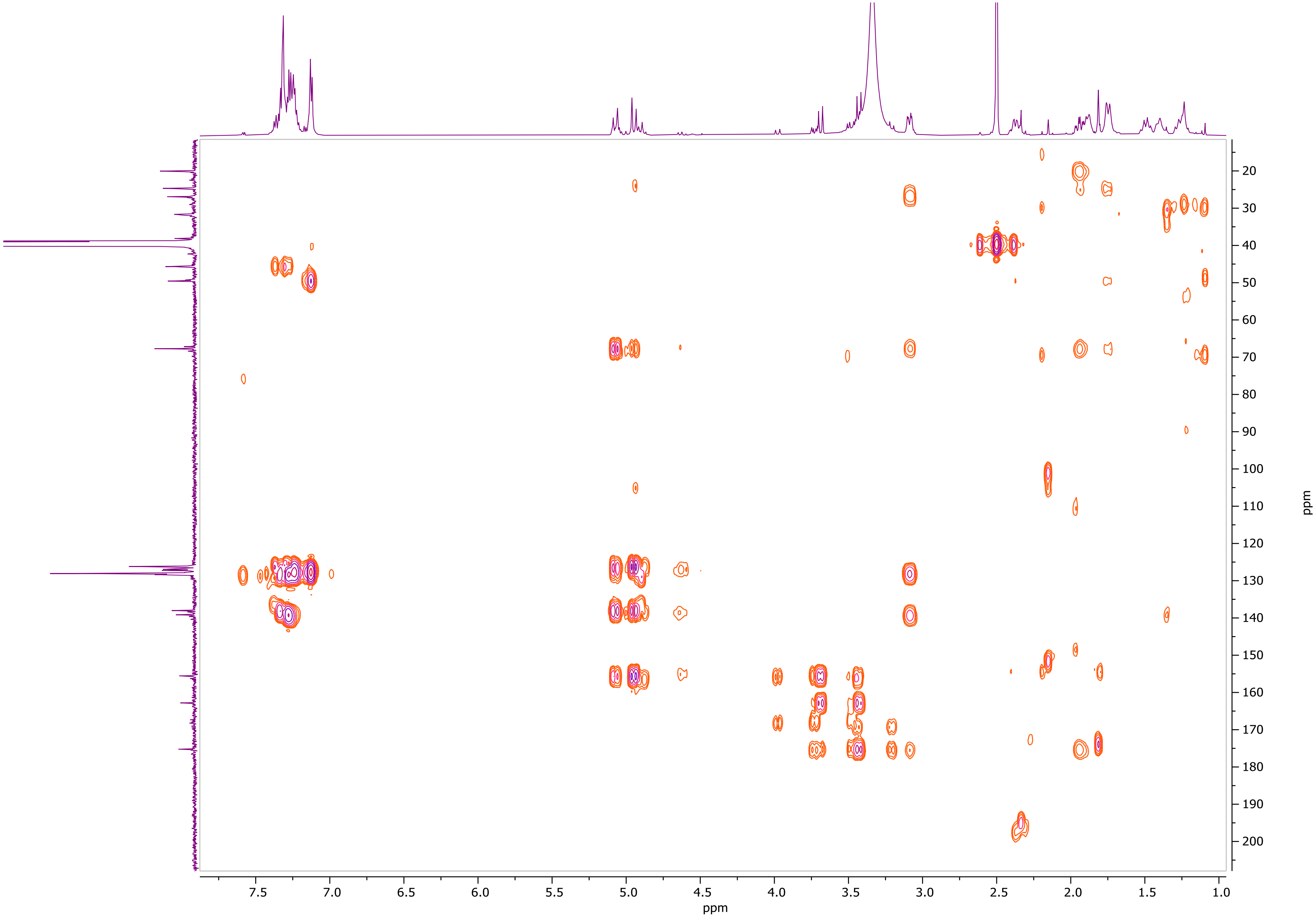

^{1}H NMR of **46** (600.16 MHz, $CDCl_3$)

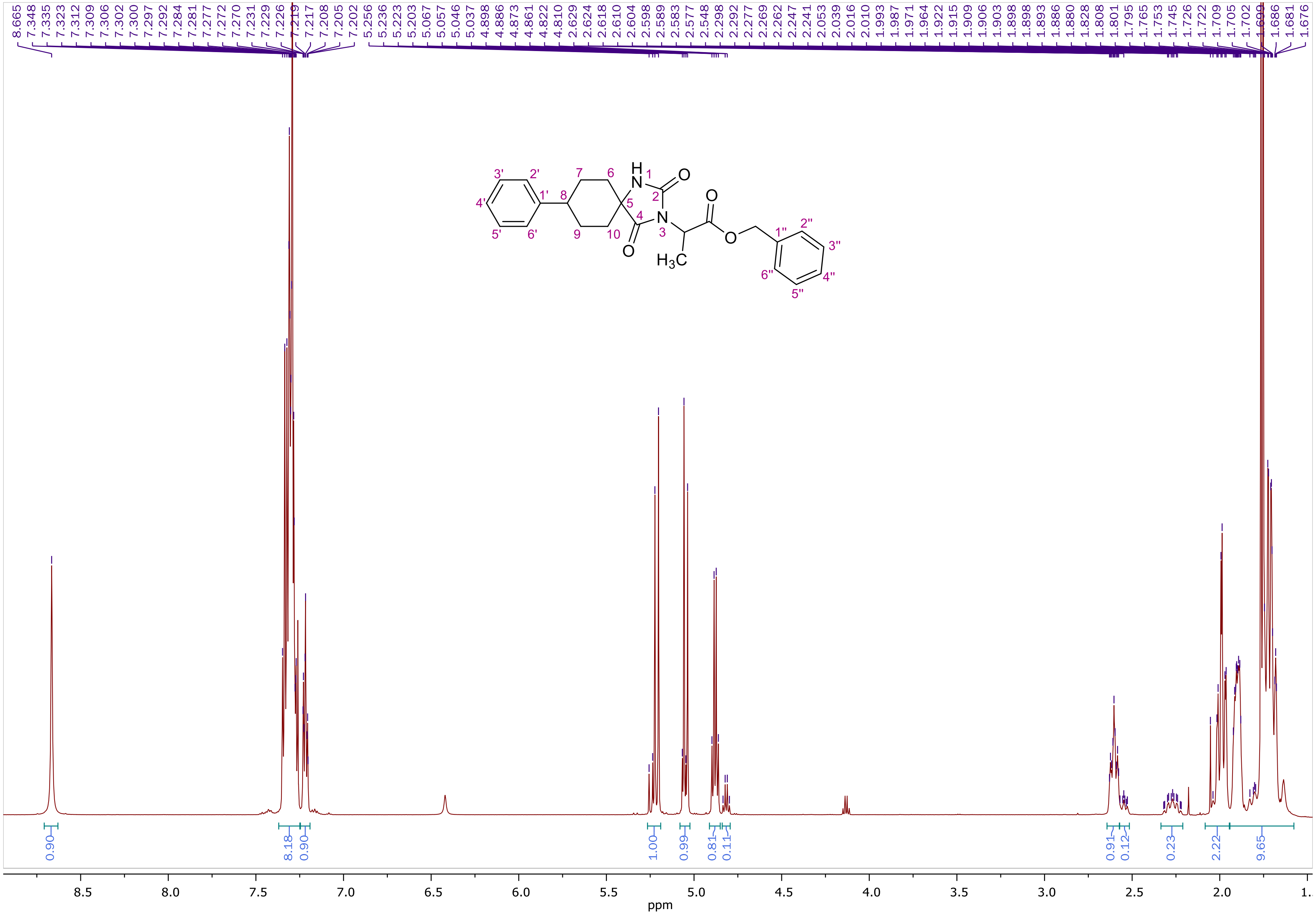

^{13}C NMR of **46** (50.32 MHz, $CDCl_3$)

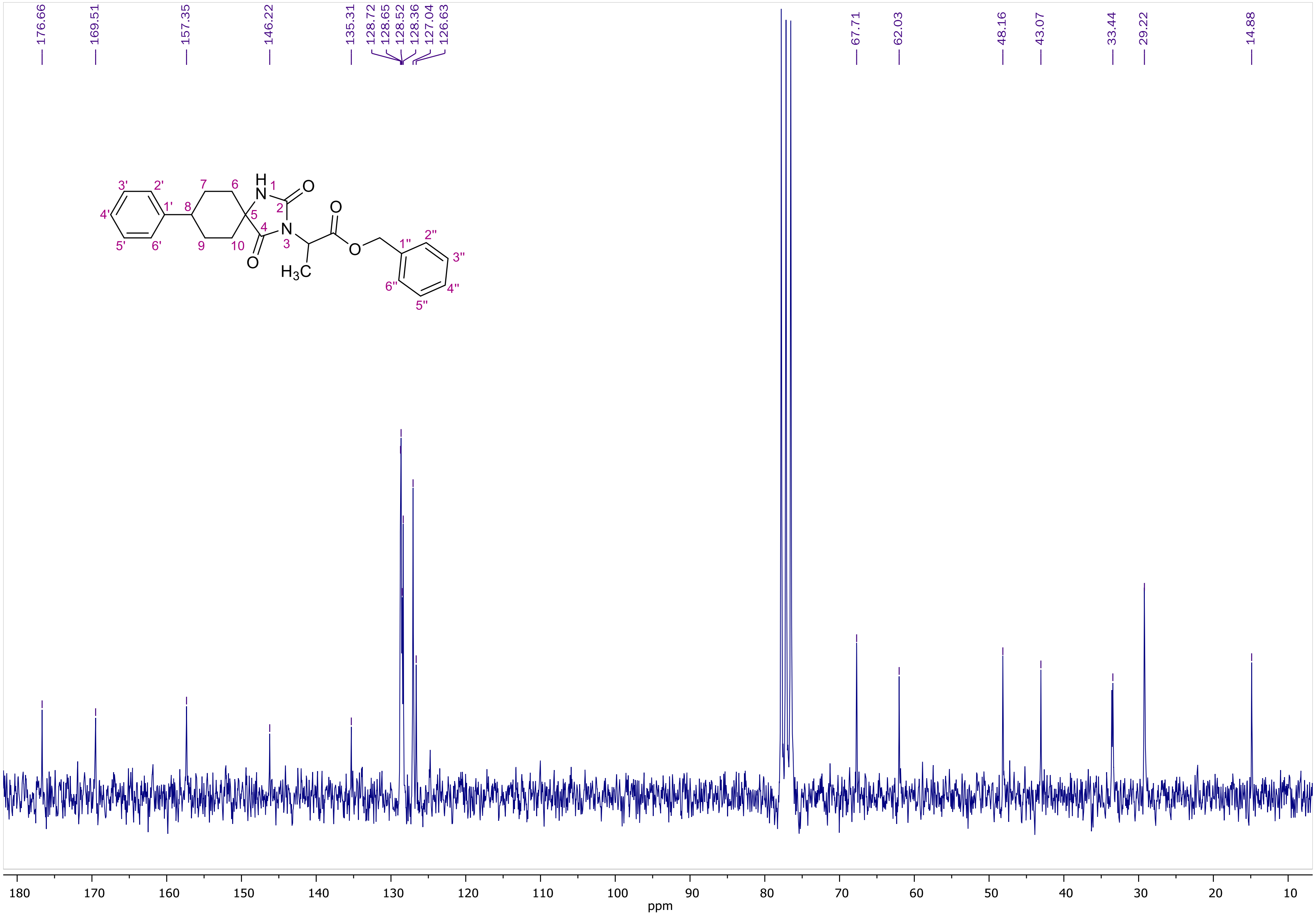

COSY NMR of **46** (400.13 MHz, $CDCl_3$)

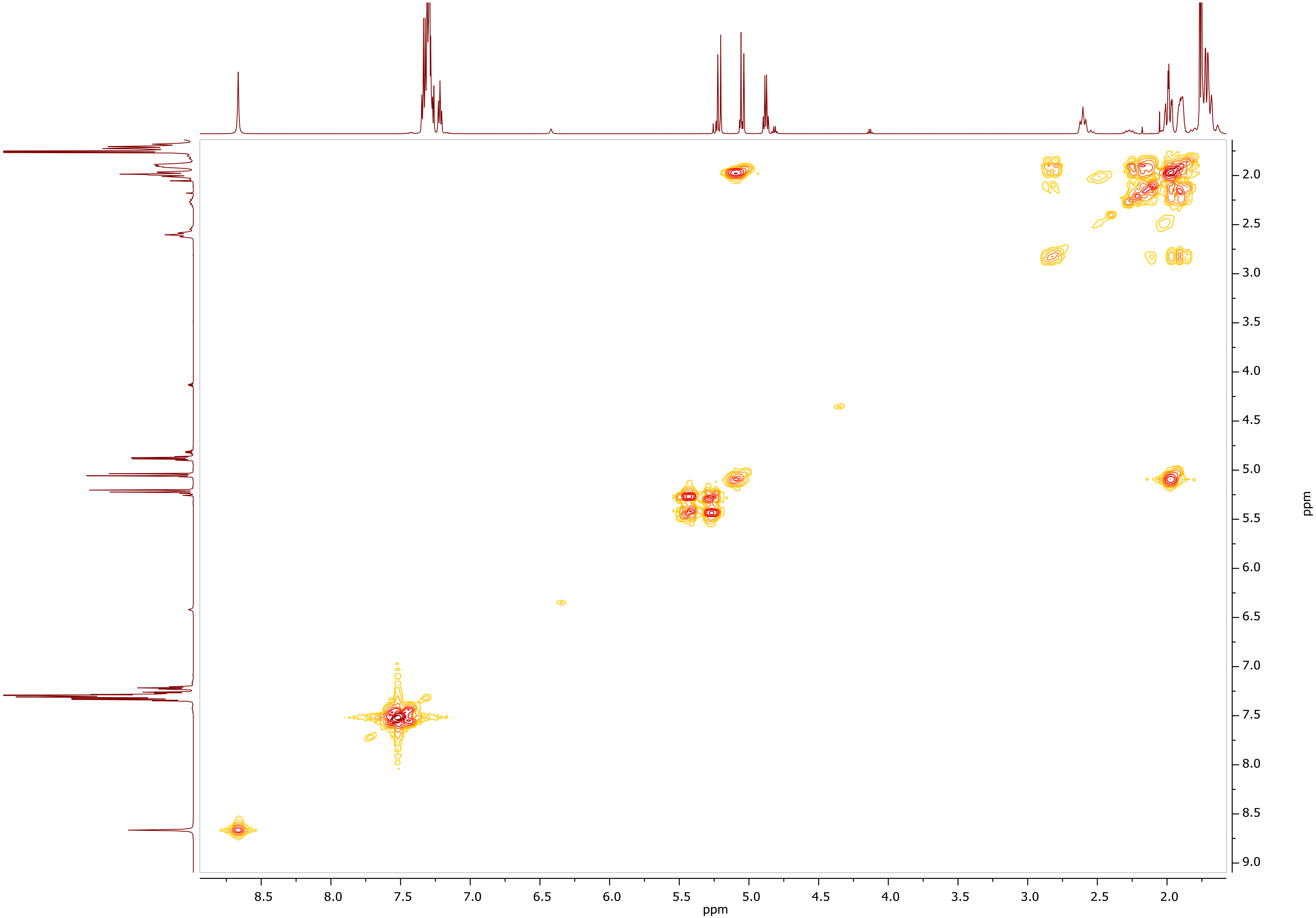

HSQC NMR of **46** (400.13 MHz, $CDCl_3$)

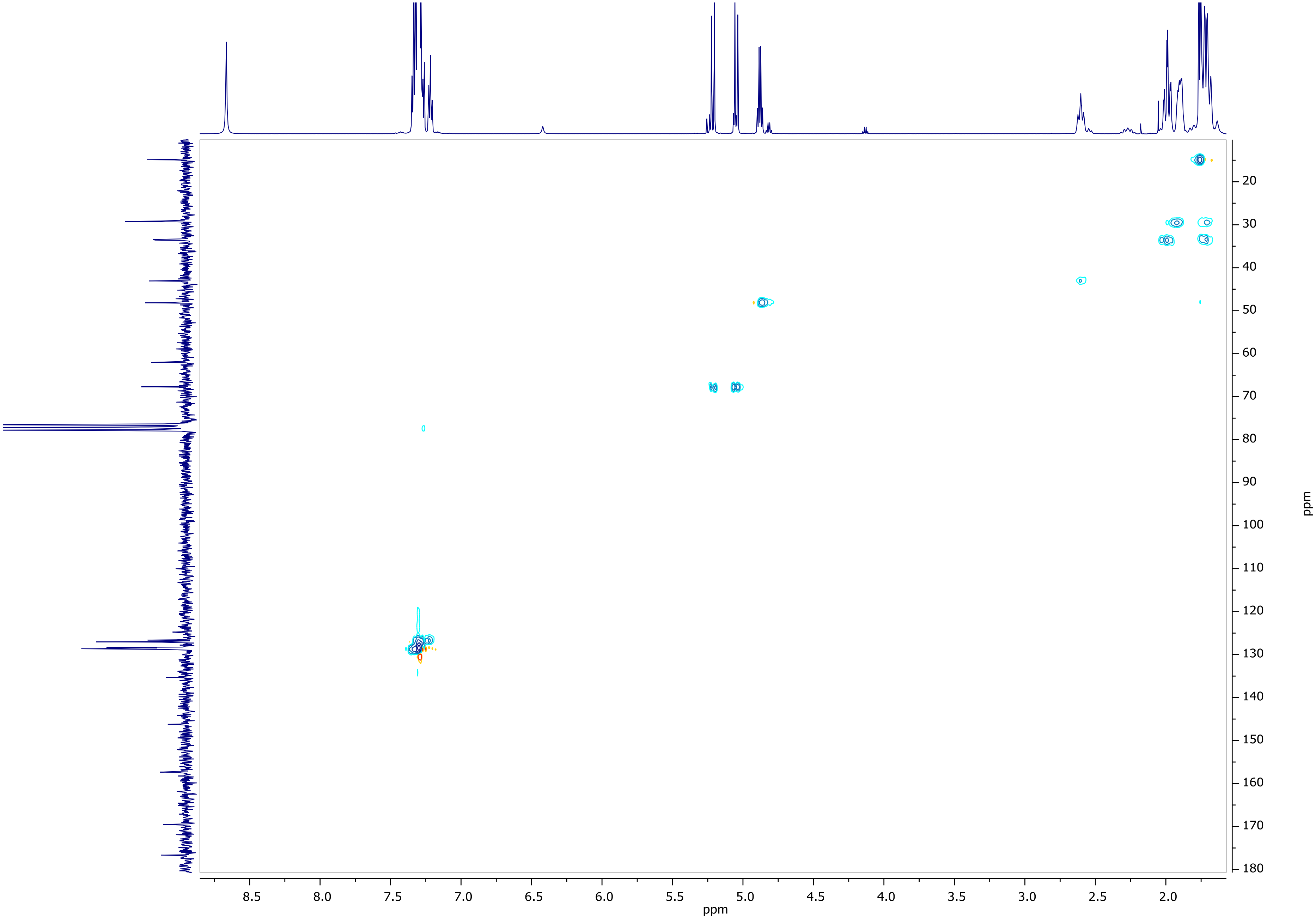

HMBC NMR of **46** (400.13 MHz, $CDCl_3$)

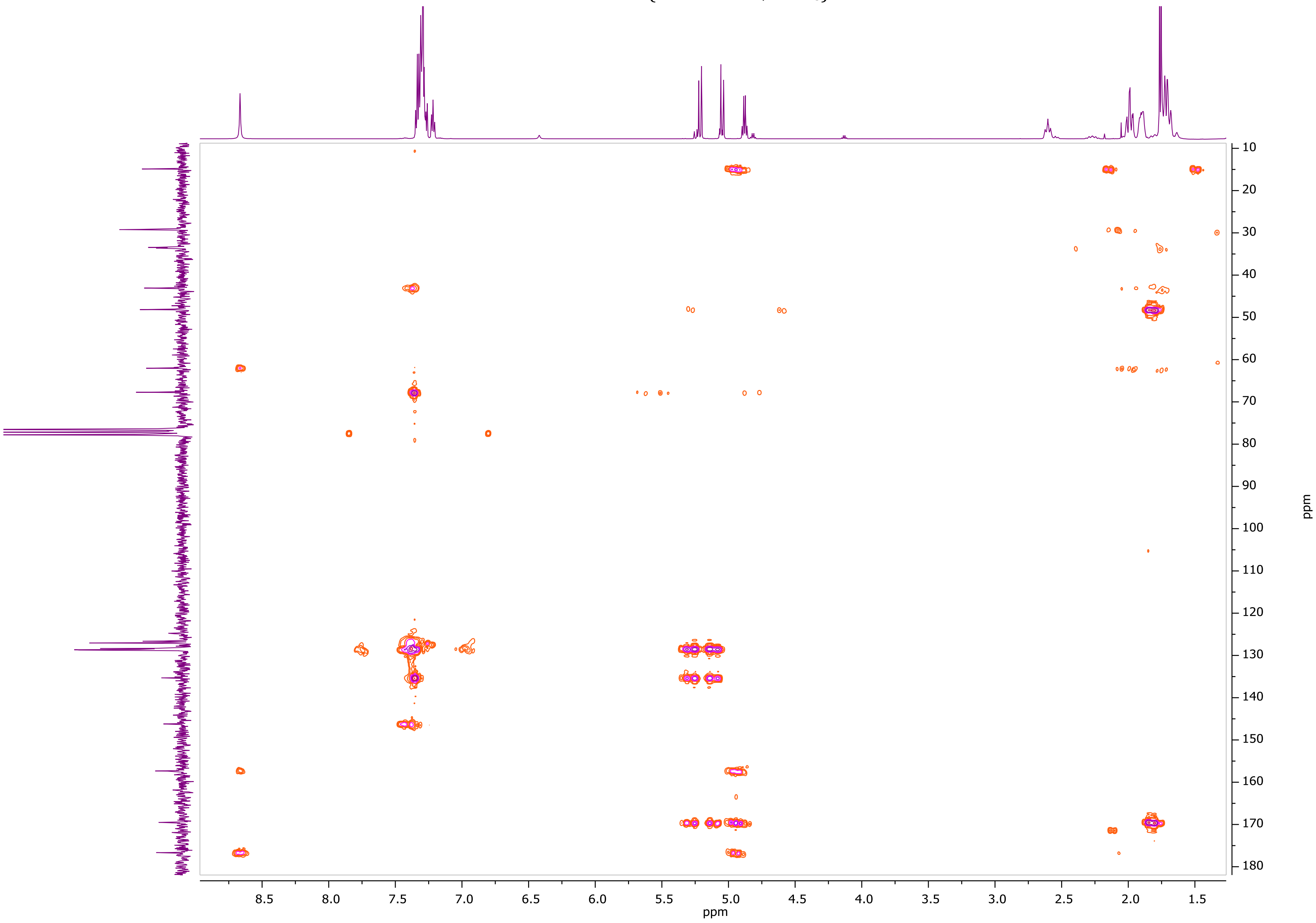

^{1}H NMR of **47** (600.11 MHz, DMSO-d_6)

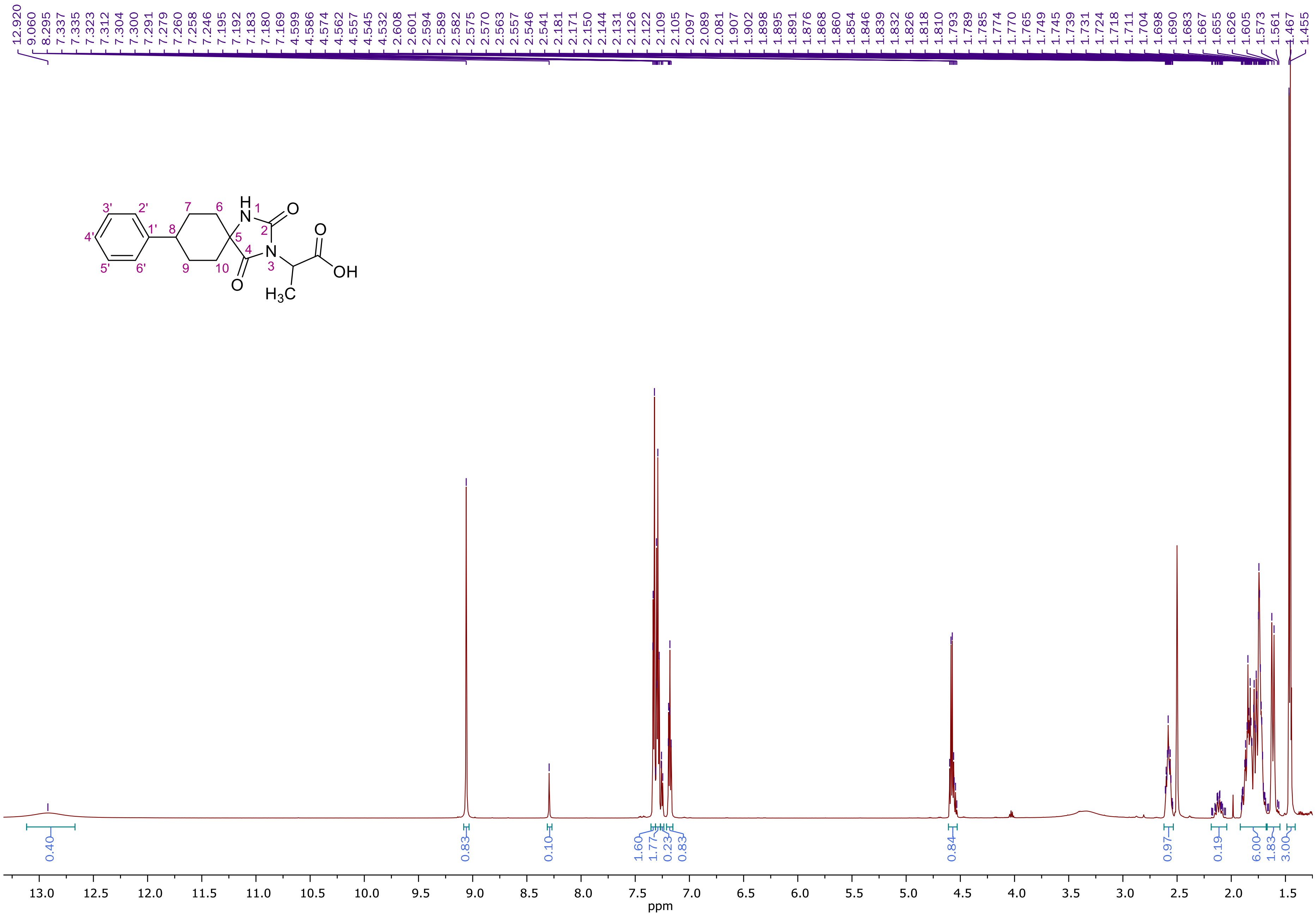

^{13}C NMR of **47** (150.9 MHz, DMSO-d_6)

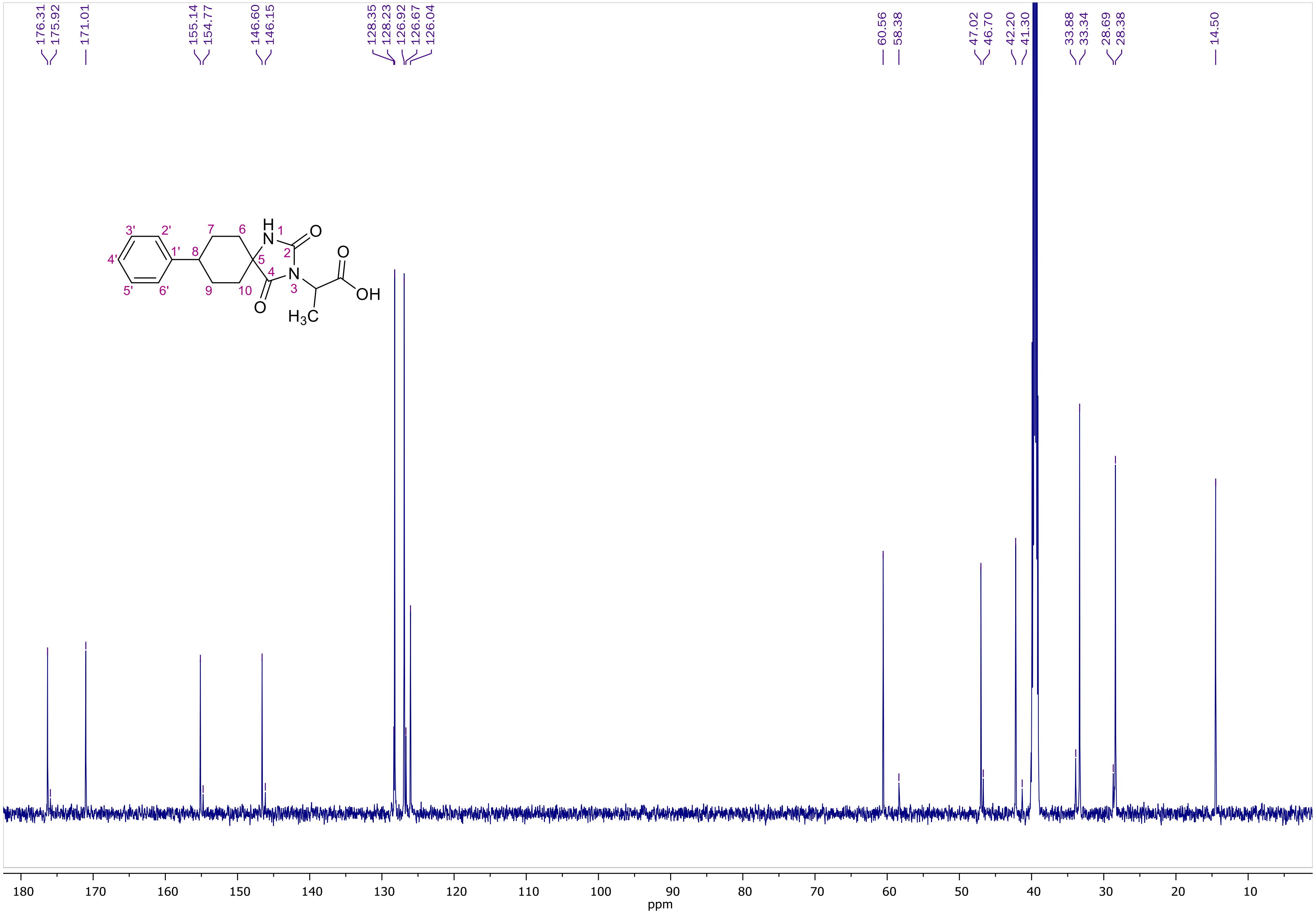

COSY NMR of **47** (400.13 MHz, DMSO-d_6)

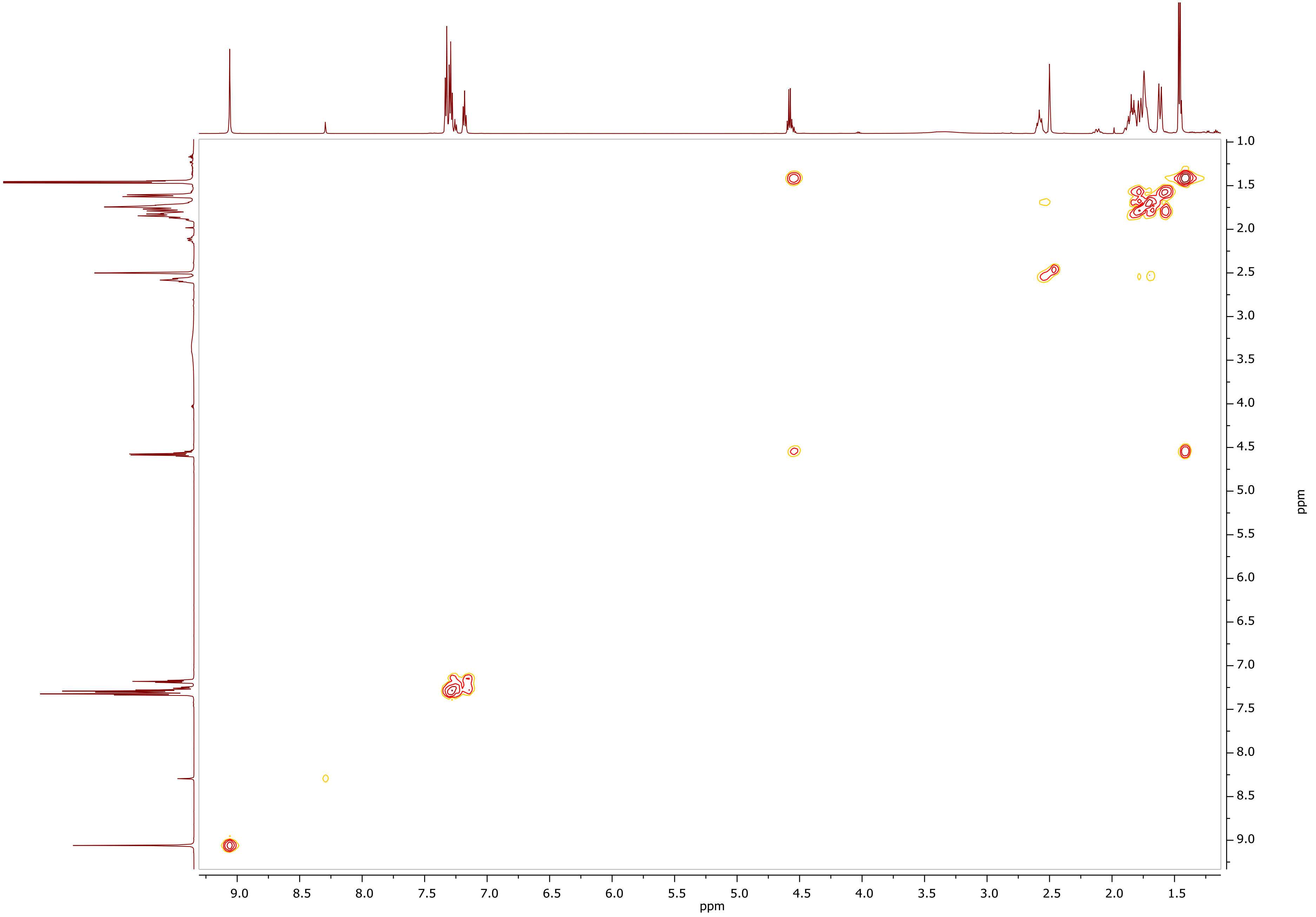

HSQC NMR of **47** (400.13 MHz, DMSO-d_6)

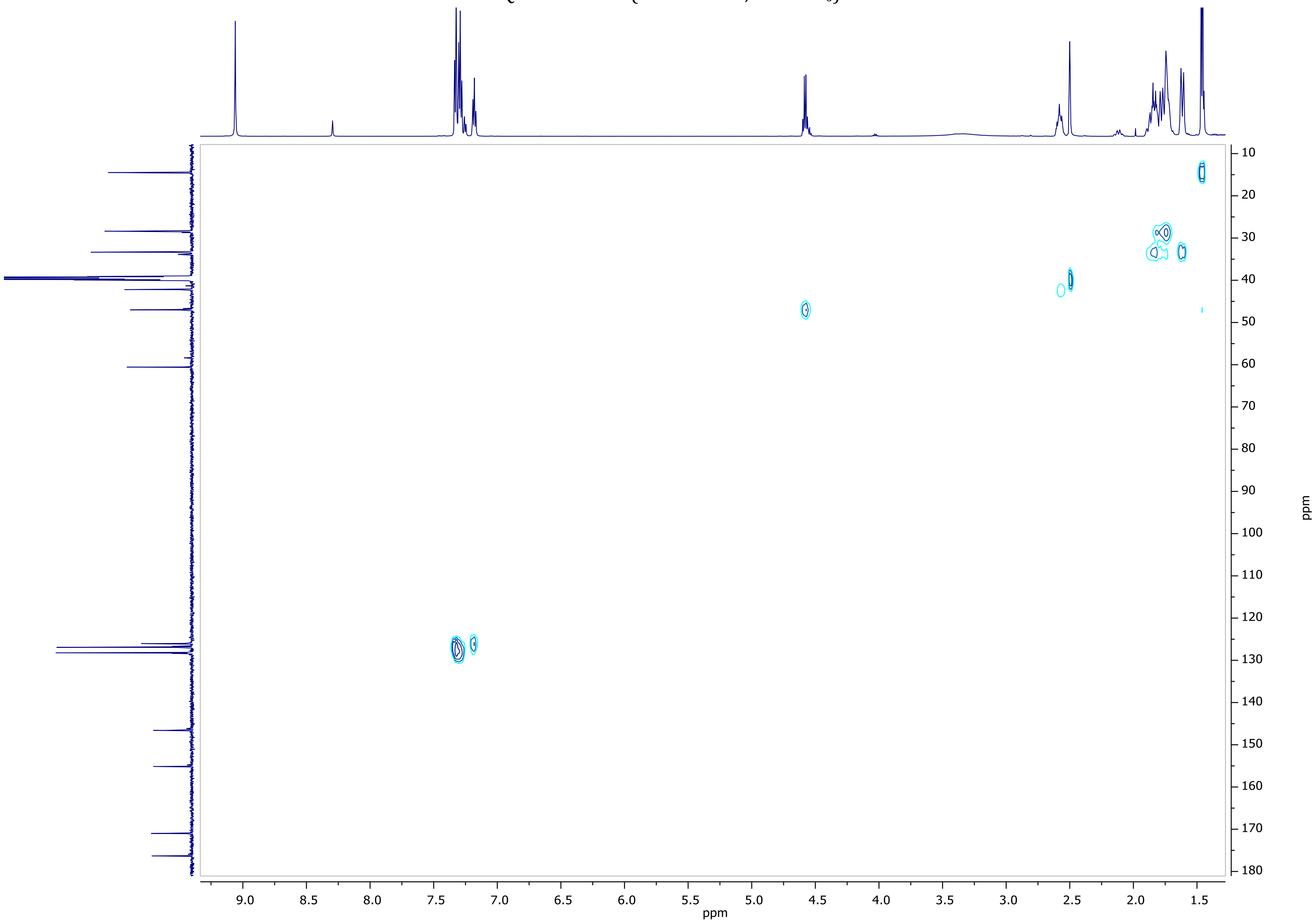

HMBC NMR of **47** (400.13 MHz, DMSO-d_6)

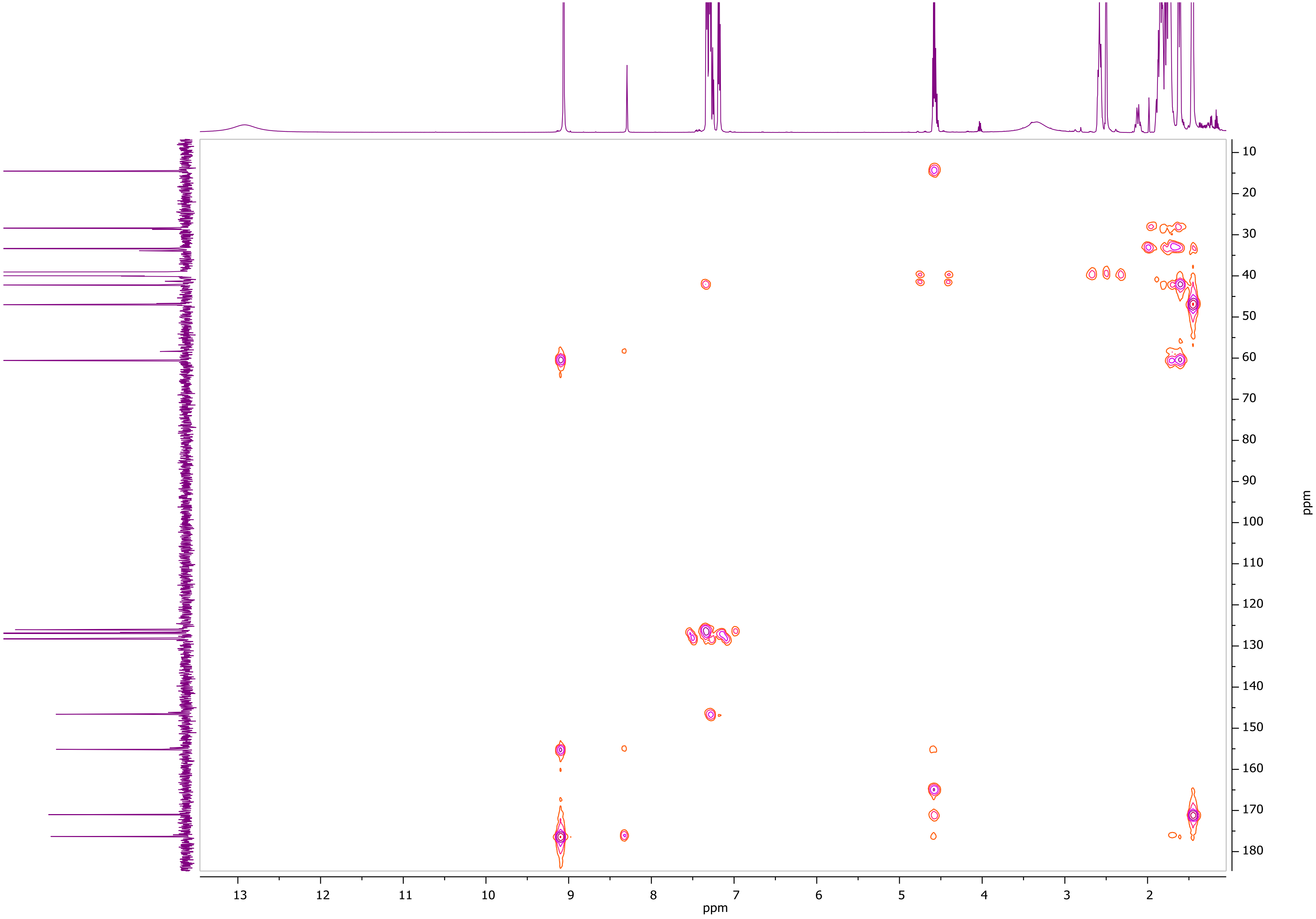

^{1}H NMR of **48** (600.11 MHz, DMSO-d_6)

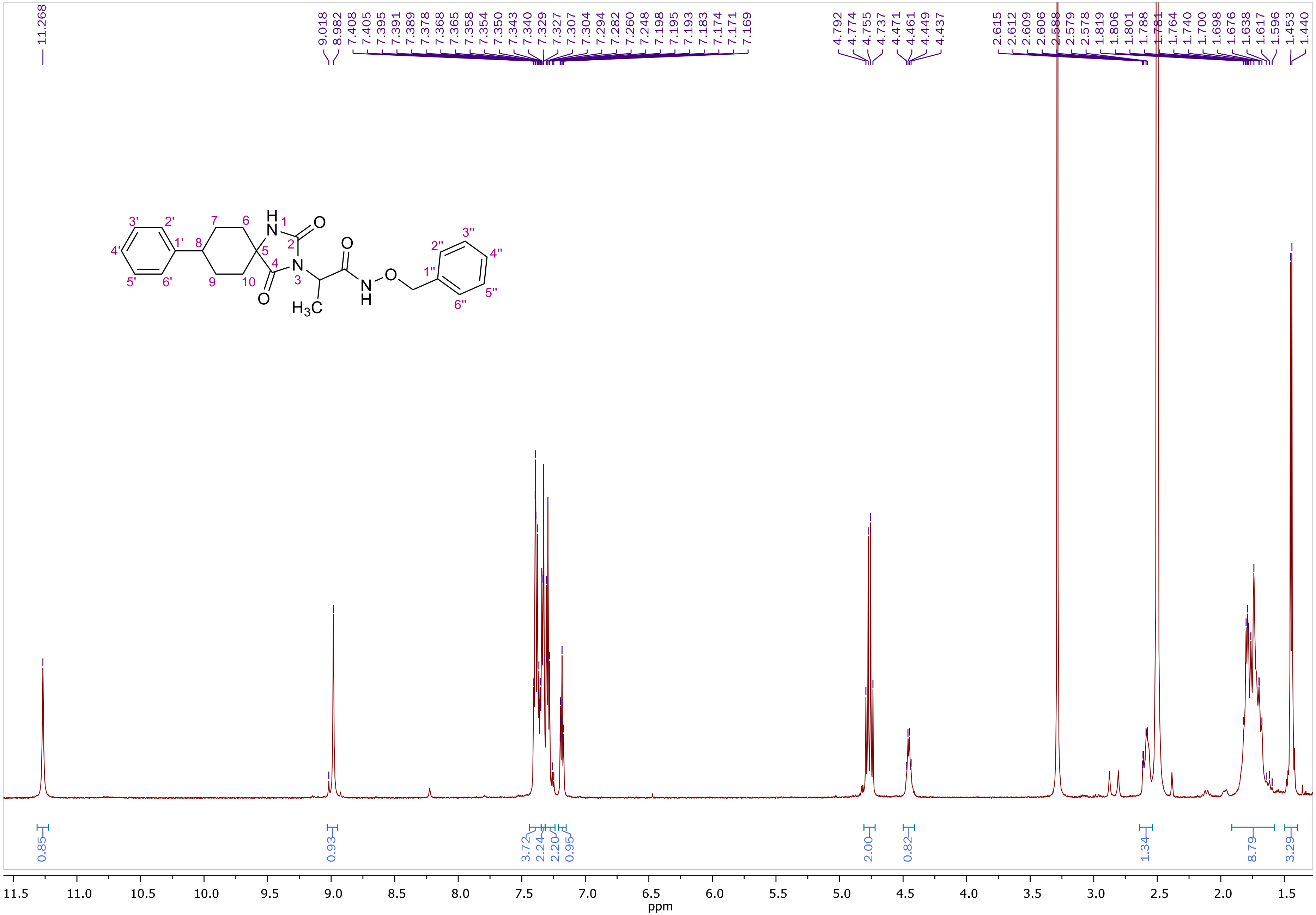

^{13}C NMR of **48** (150.9 MHz, DMSO-d_6)

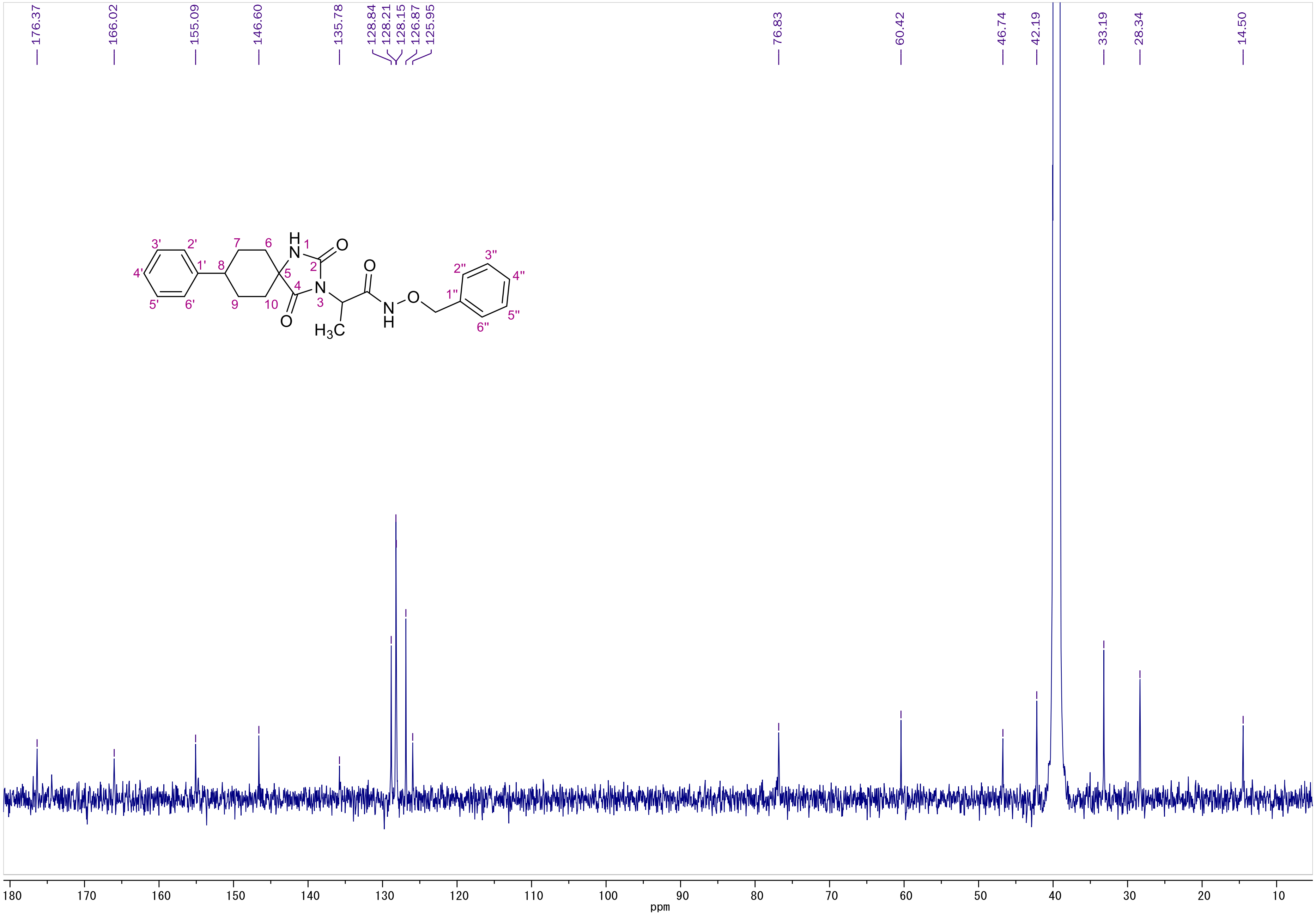

COSY NMR of **48** (600.11 MHz, DMSO-d_6)

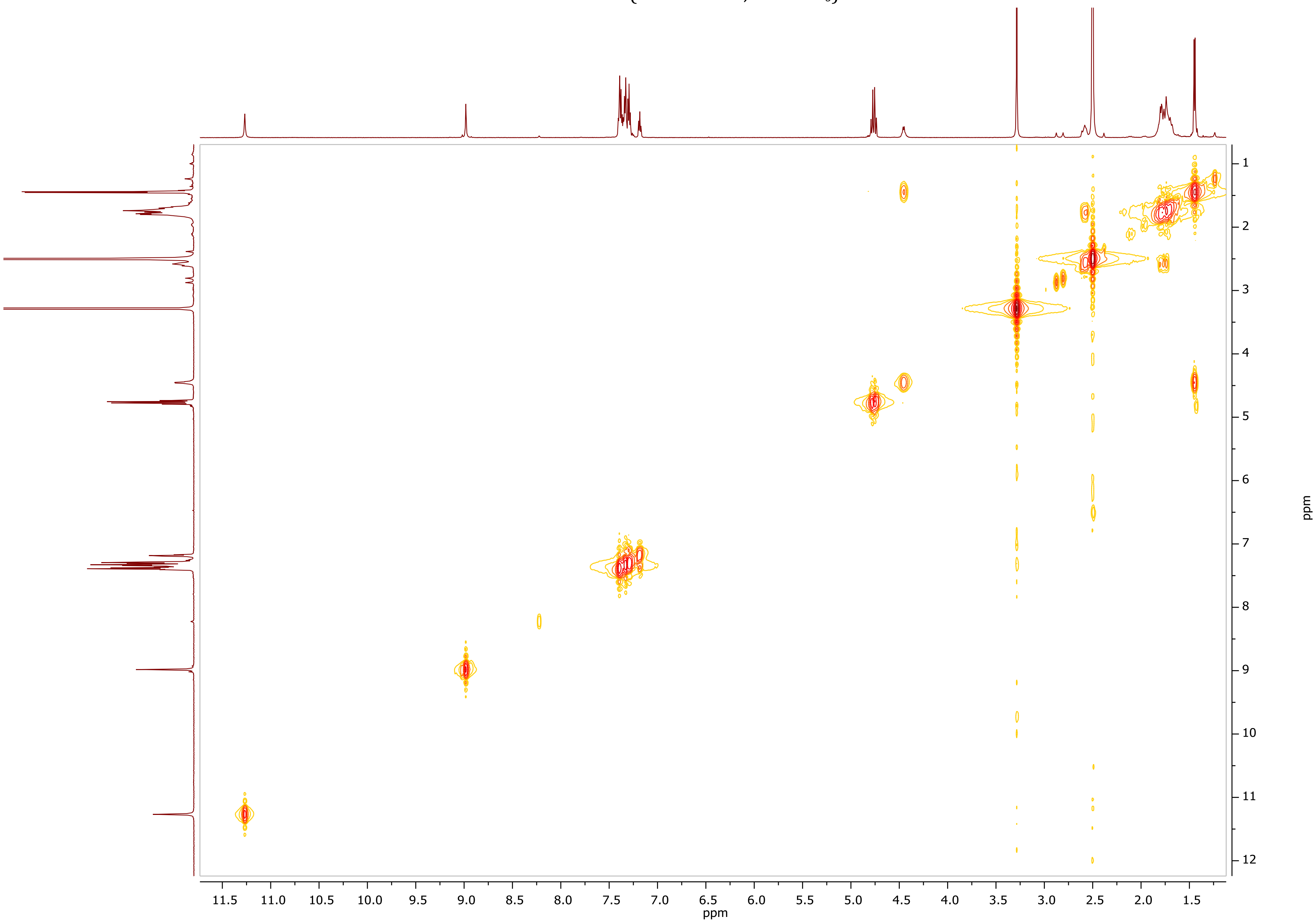

HSQC-DEPT NMR of **48** (600.11 MHz, DMSO-d_6)

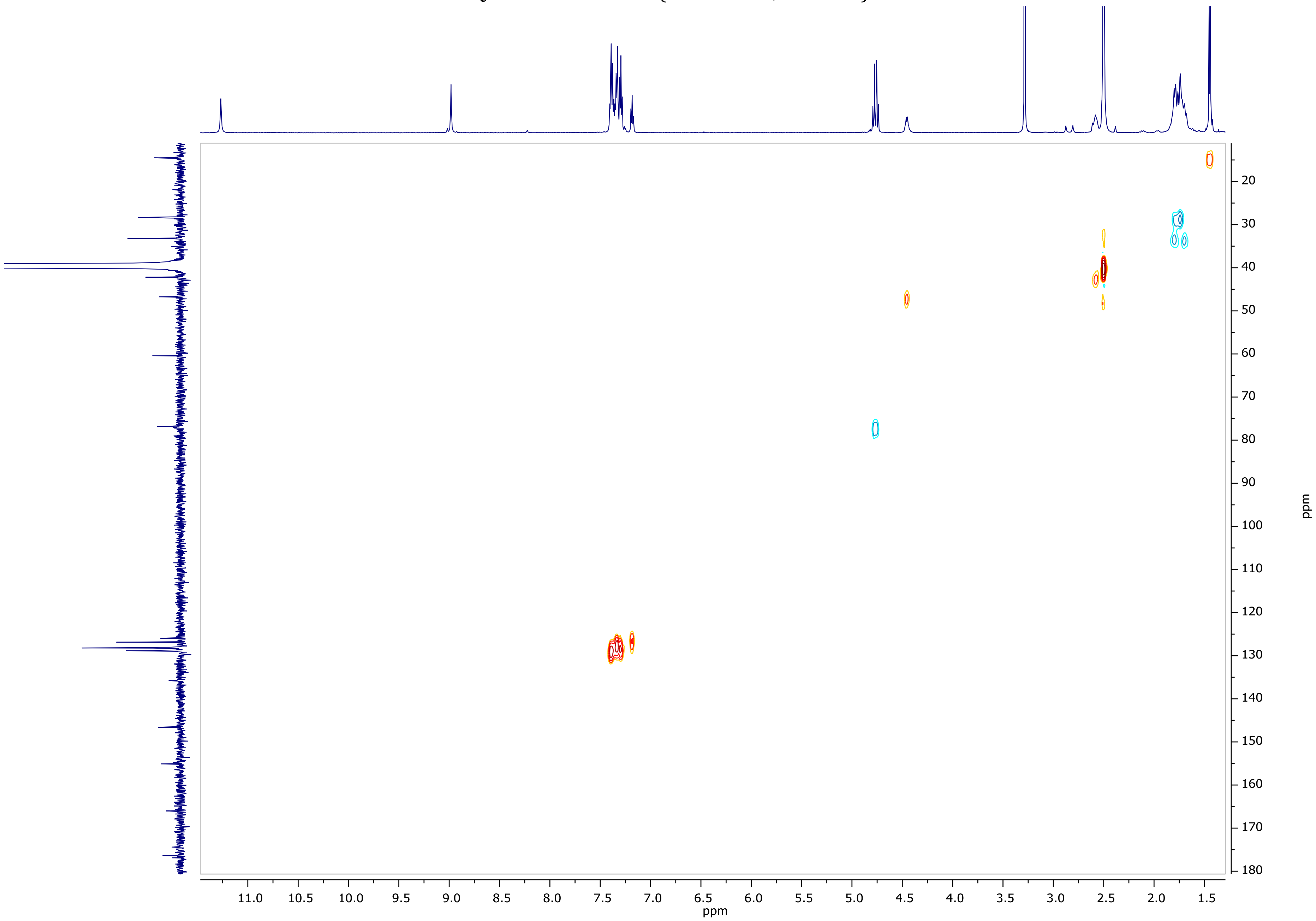

HMBC NMR of **48** (600.11 MHz, DMSO-d_6)

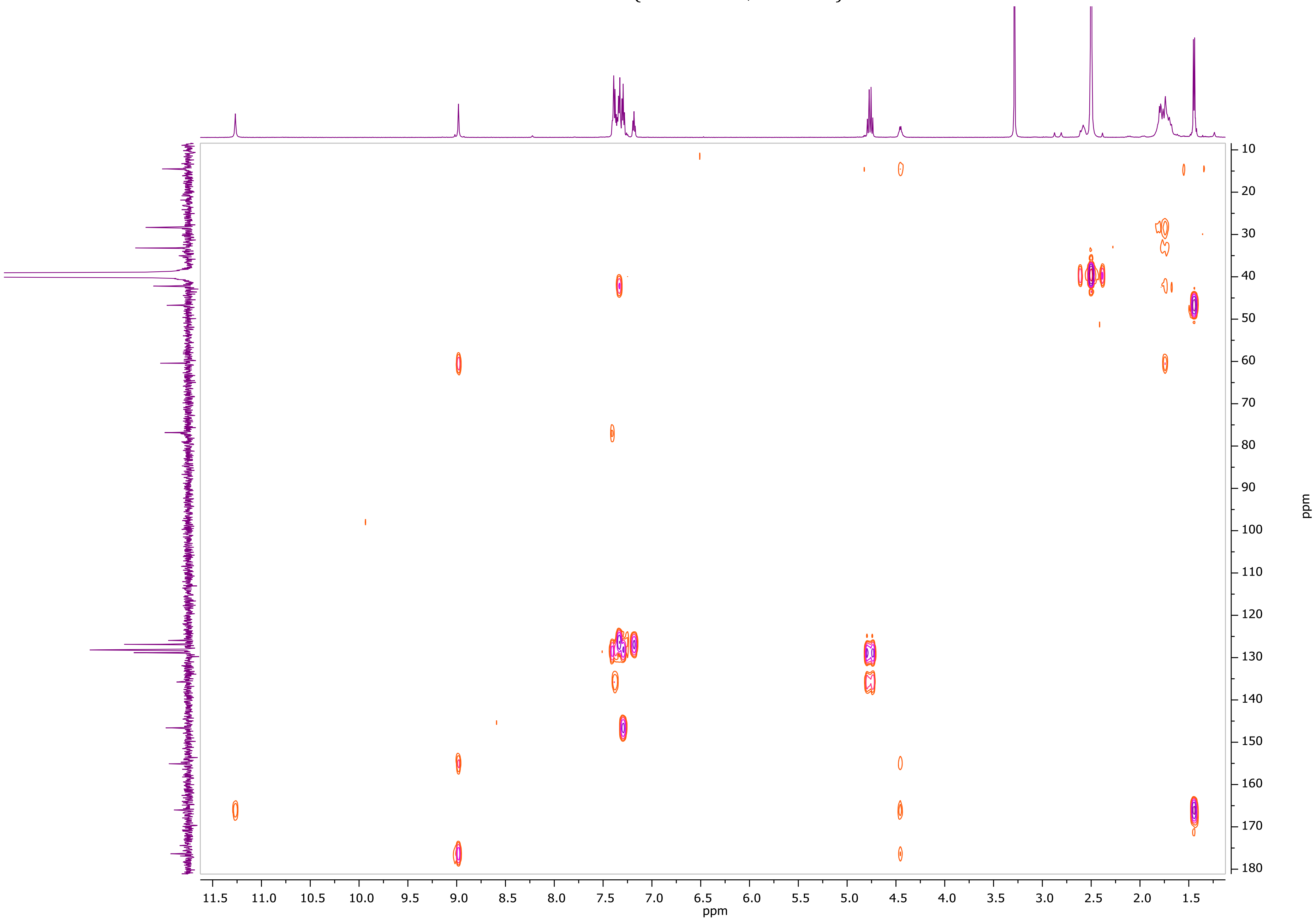

^{1}H NMR of **49** (600.11 MHz, DMSO-d_6)

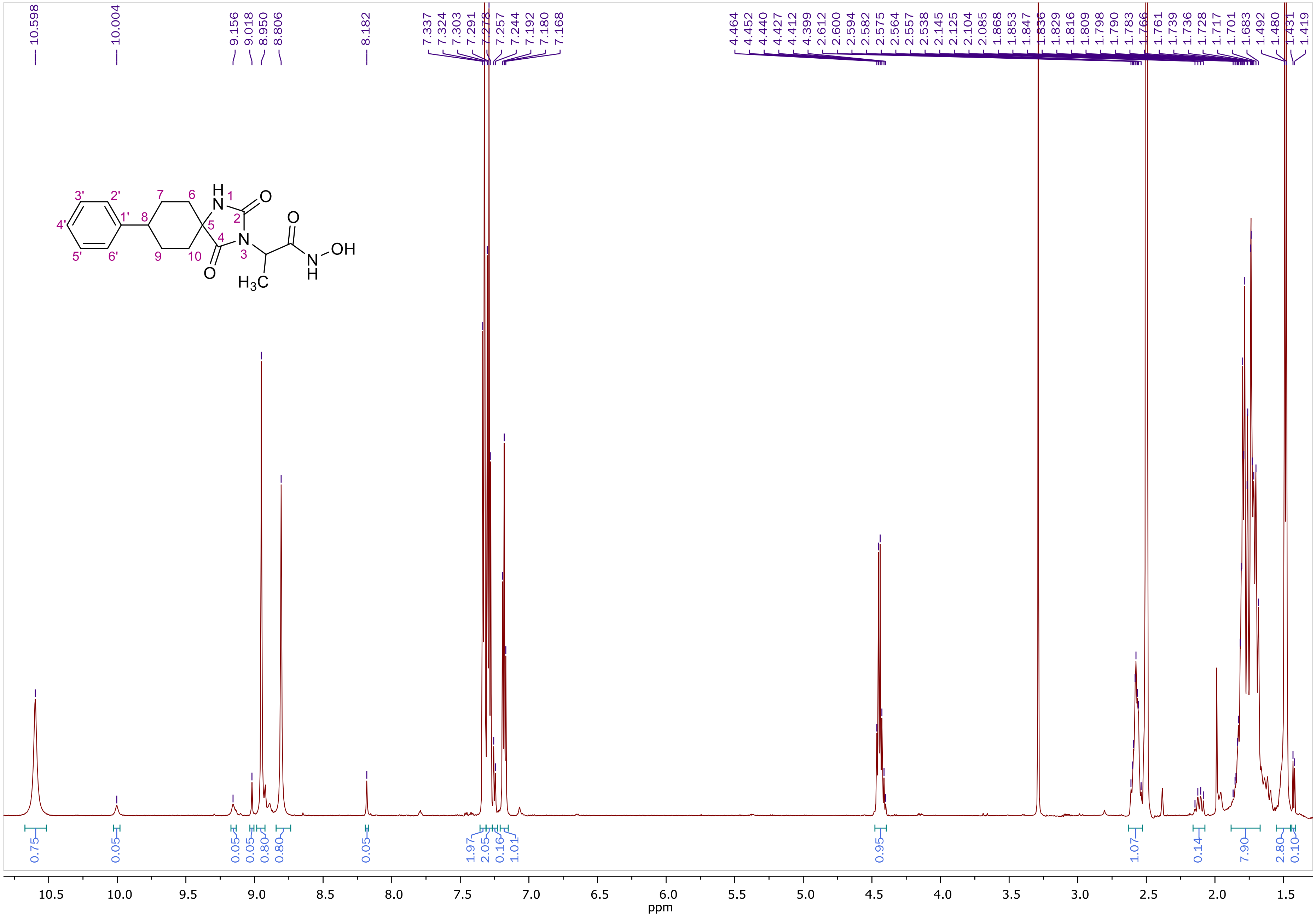

^{13}C NMR of **49** (150.9 MHz, DMSO-d_6)

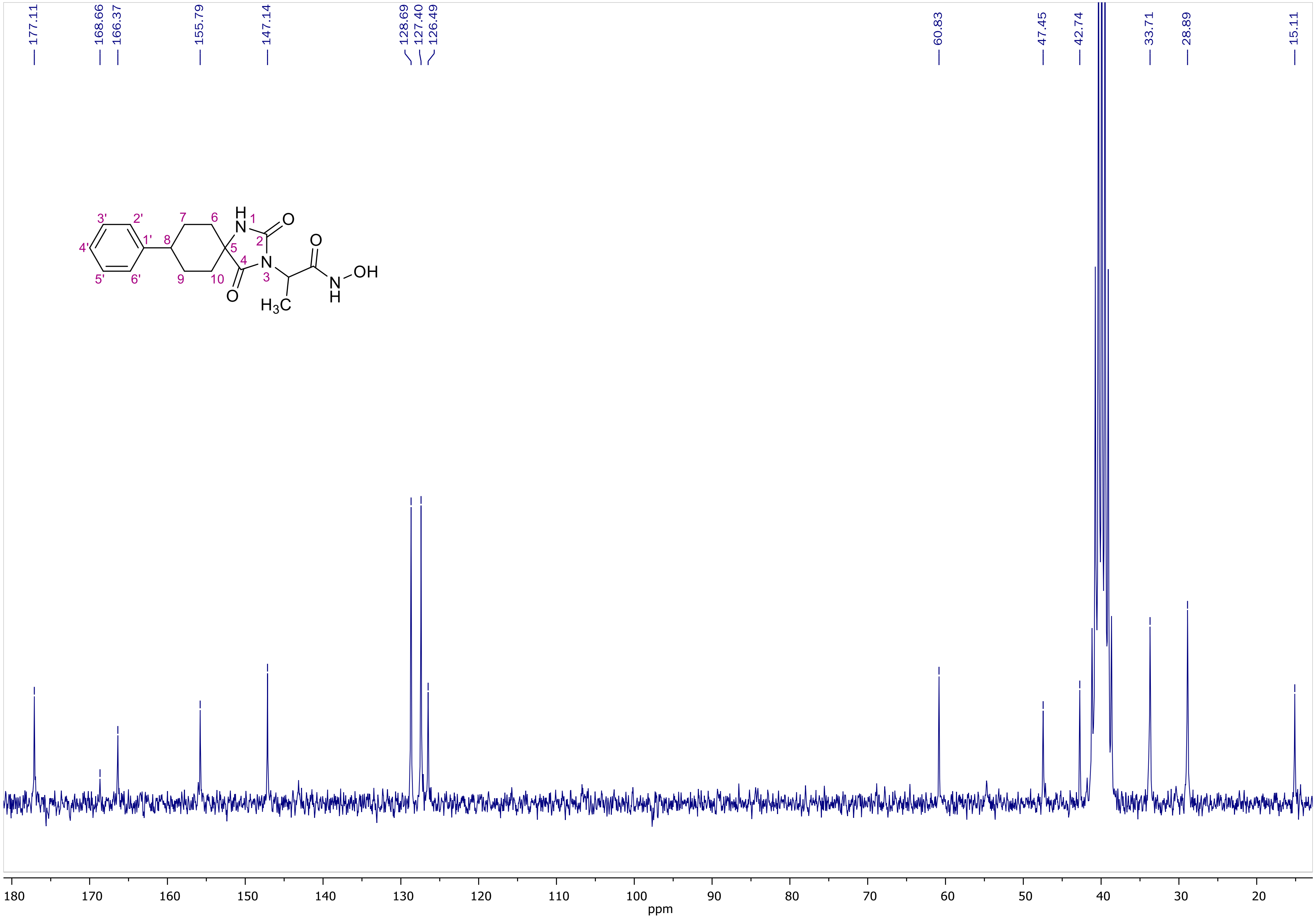

COSY NMR of **49** (600.11 MHz, DMSO-d_6)

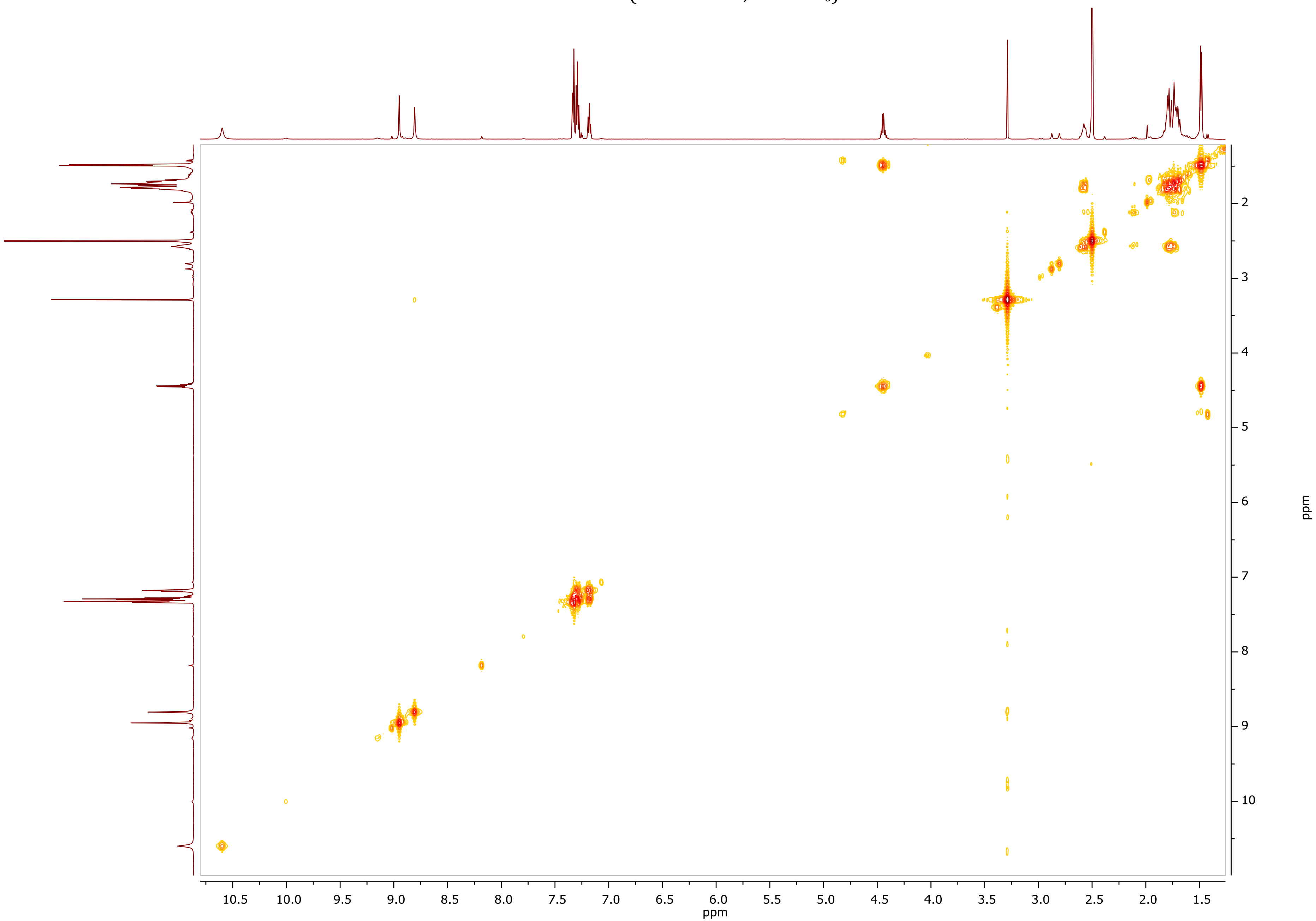

HSQC-DEPT NMR of **49** (600.11 MHz, DMSO-d_6)

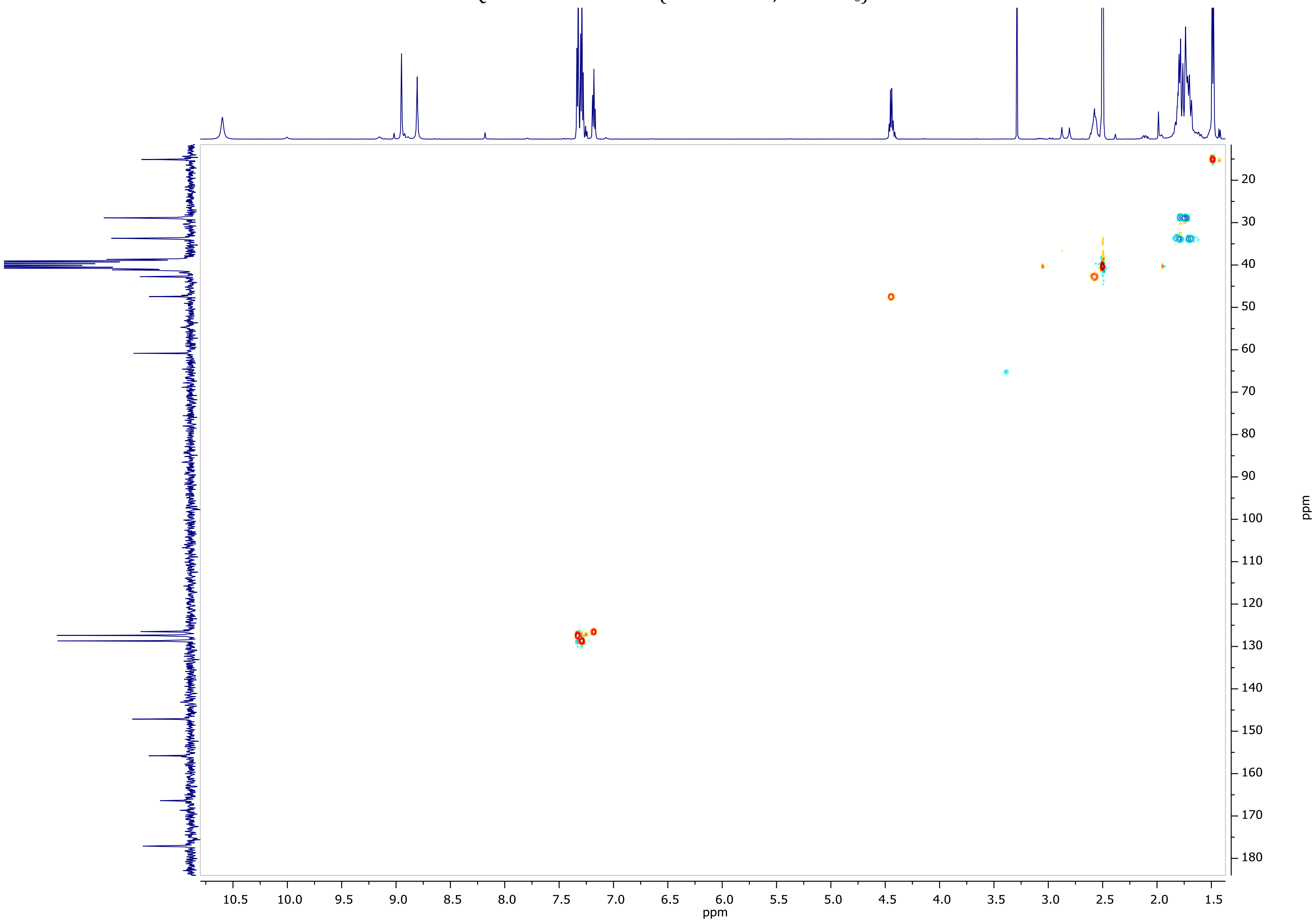

HMBC NMR of **49** (600.11 MHz, DMSO-d_6)

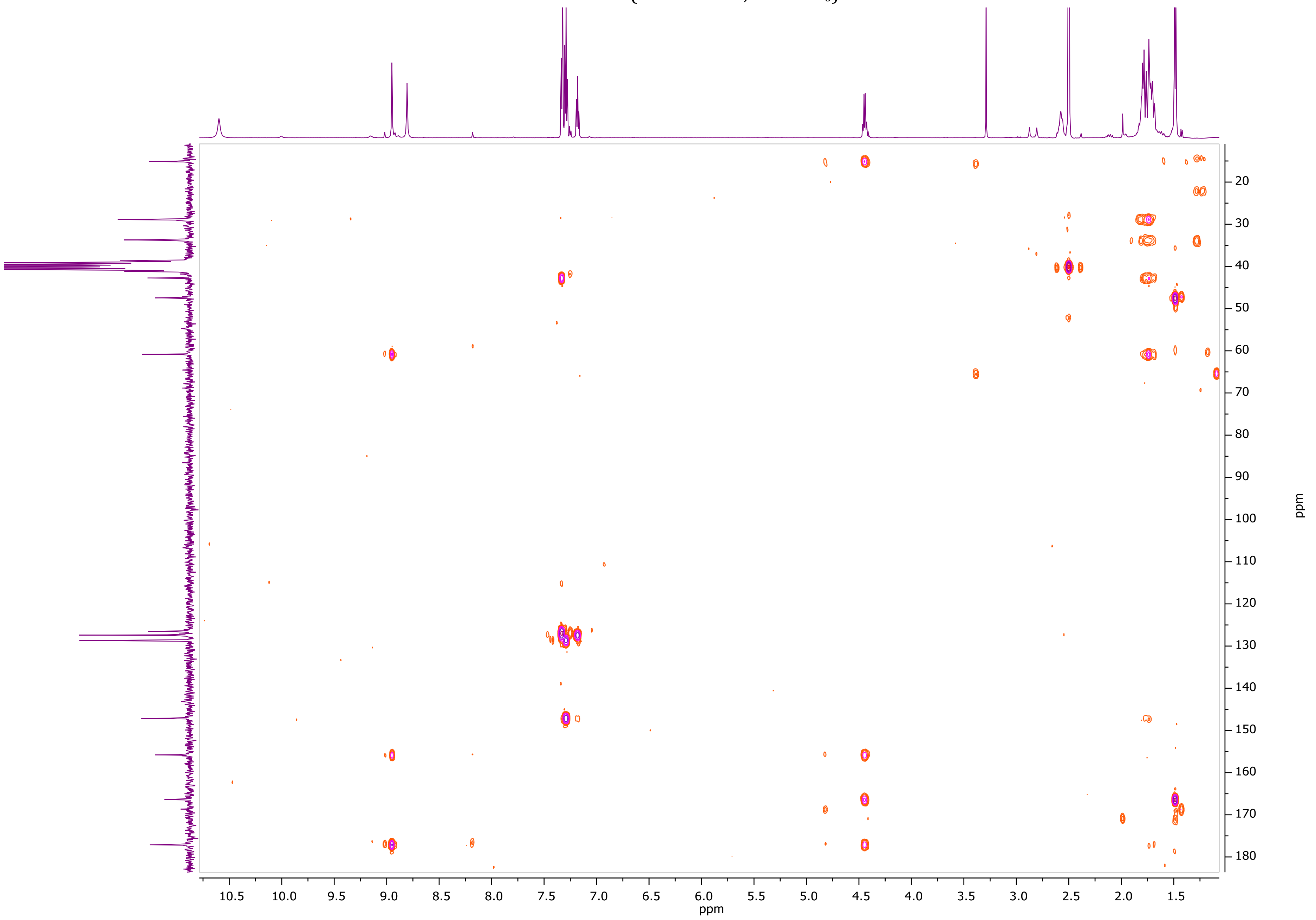

¹H NMR of **50** (400.13 MHz, $CDCl_3$)

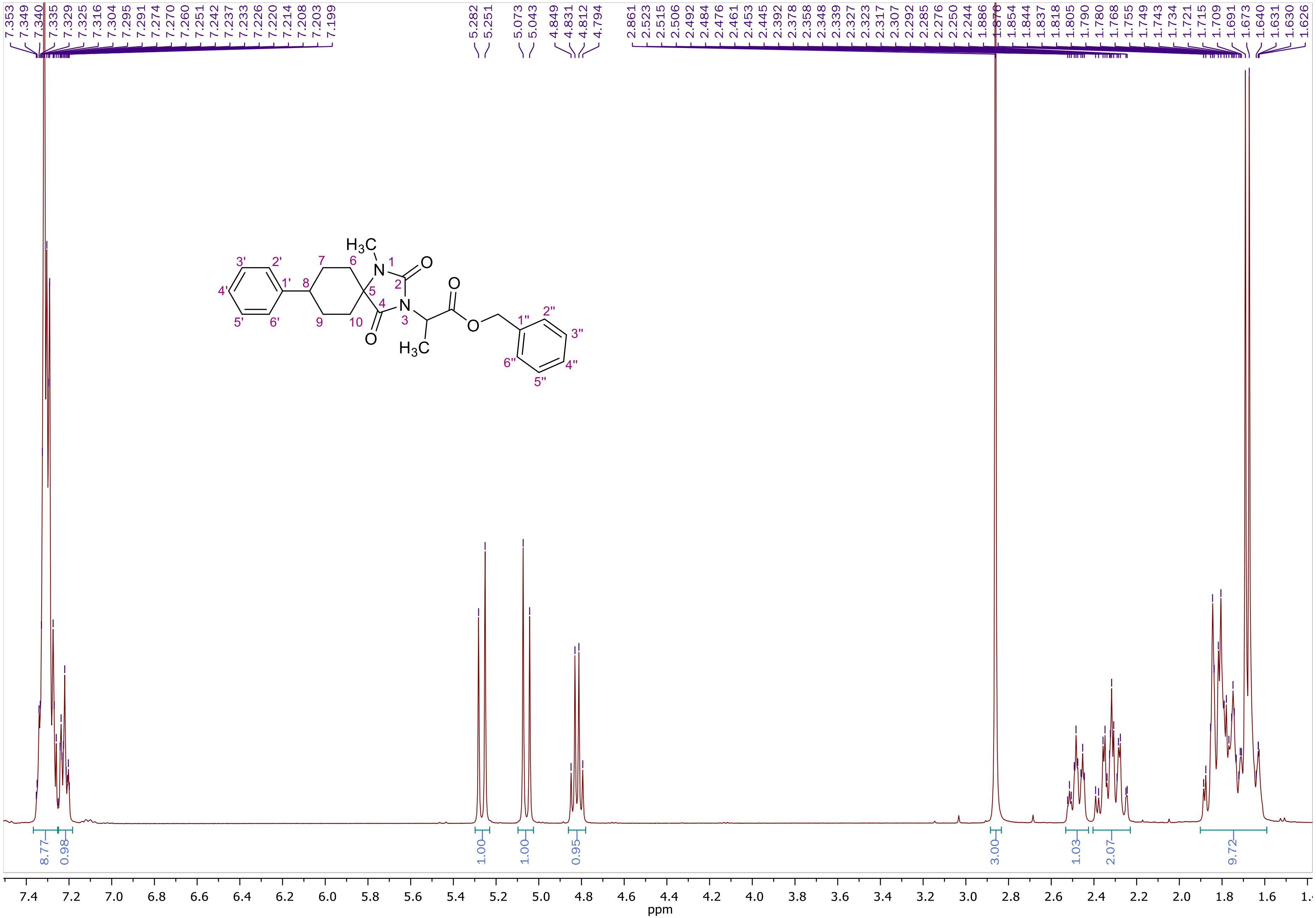

^{13}C NMR of **50** (150.9 MHz, $CDCl_3$)

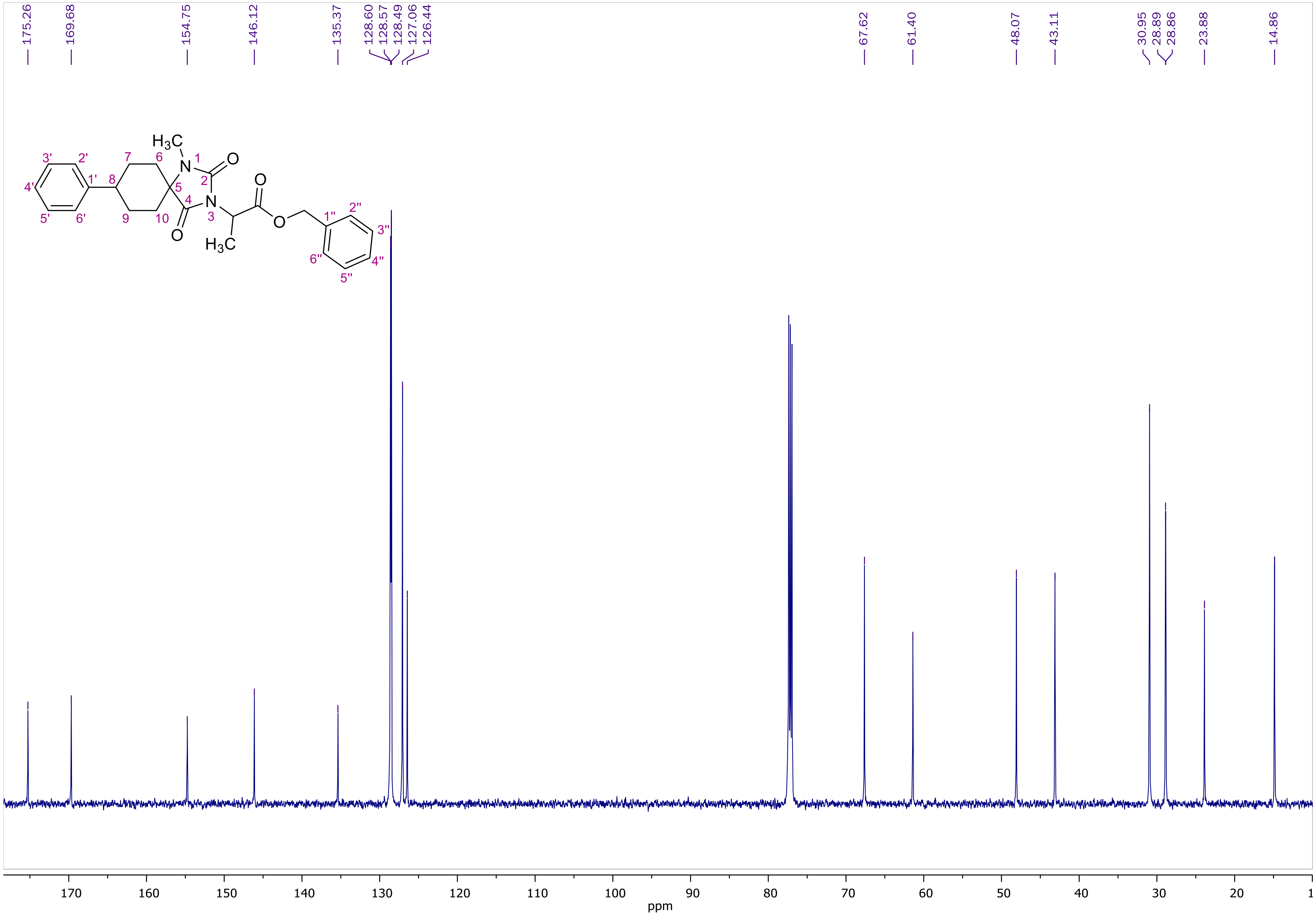

COSY NMR of **50** (400.13 MHz, $CDCl_3$)

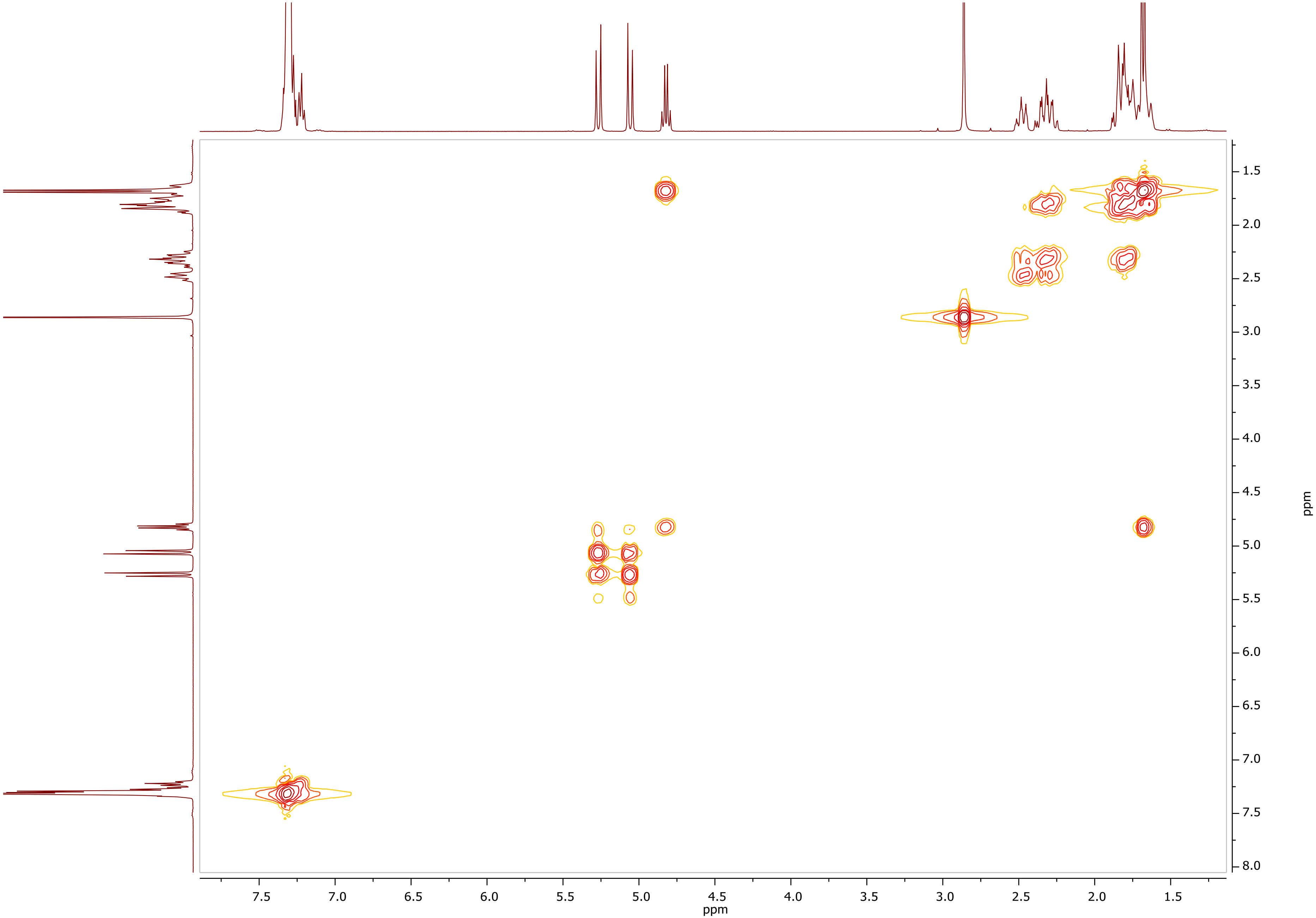

HSQC NMR of **50** (400.13 MHz, $CDCl_3$)

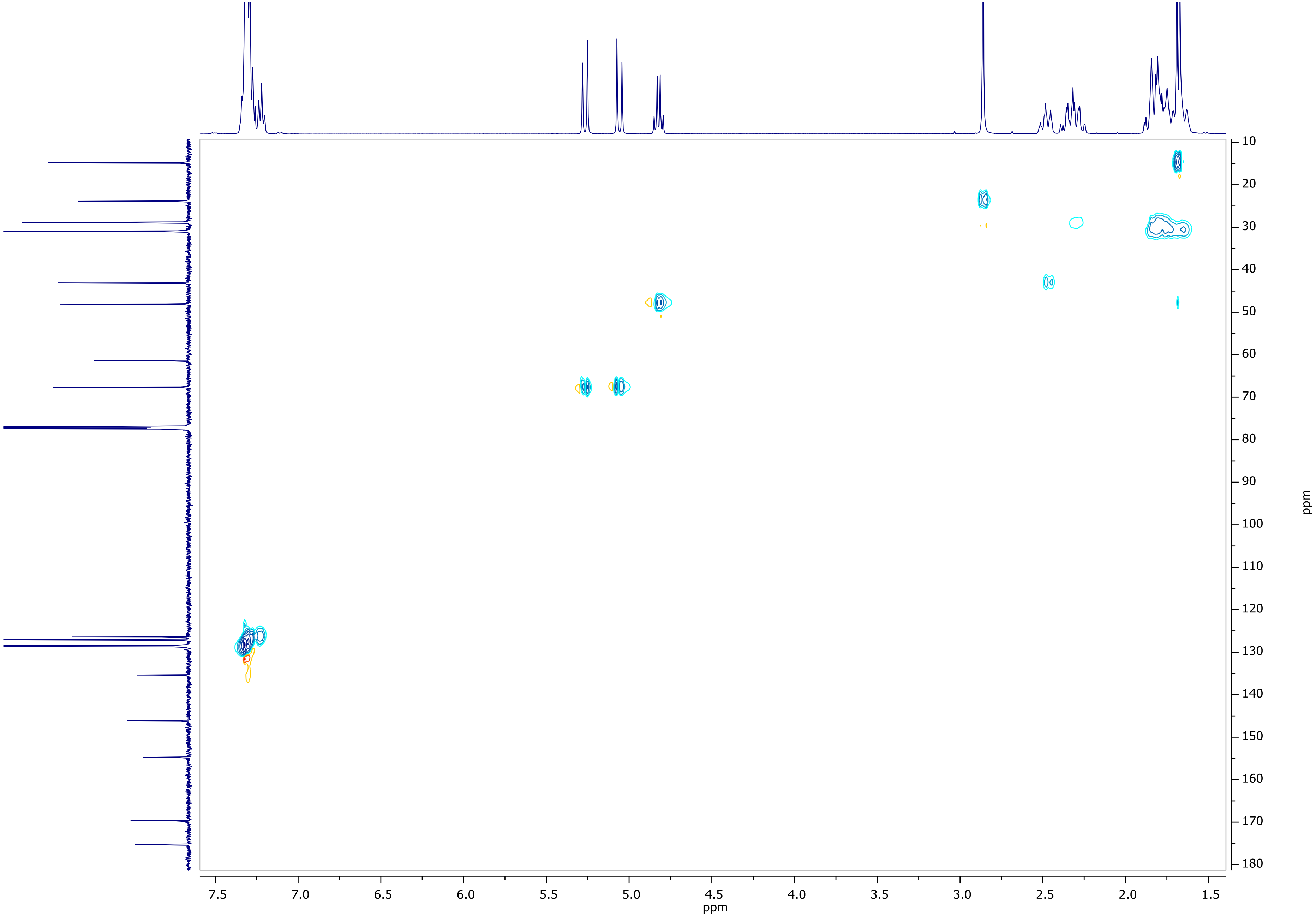

HMBC NMR of **50** (400.13 MHz, $CDCl_3$)

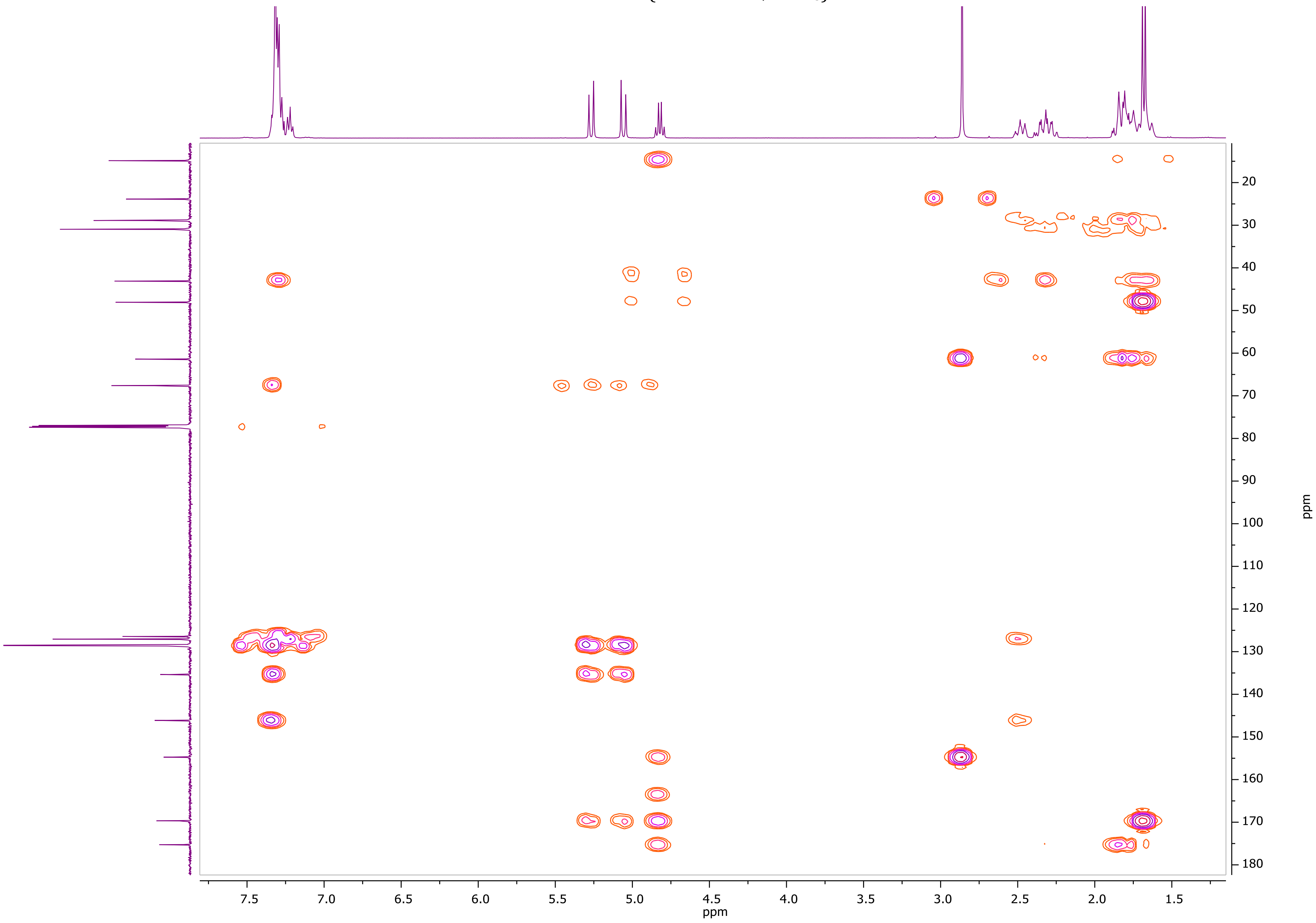

^{1}H NMR of **51** (400.13 MHz, DMSO-d_6)

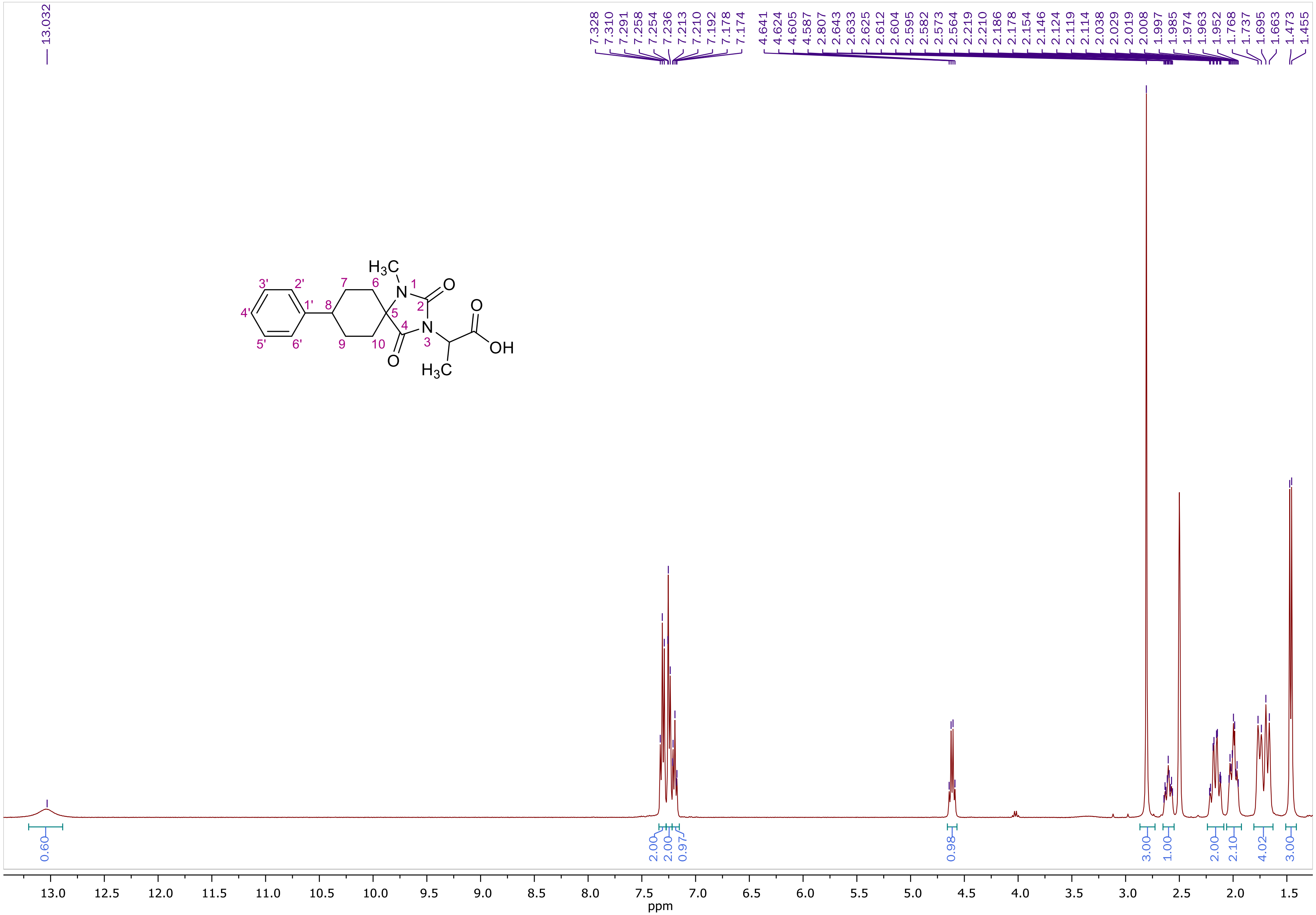

^{13}C NMR of **51** (150.9 MHz, DMSO-d_6)

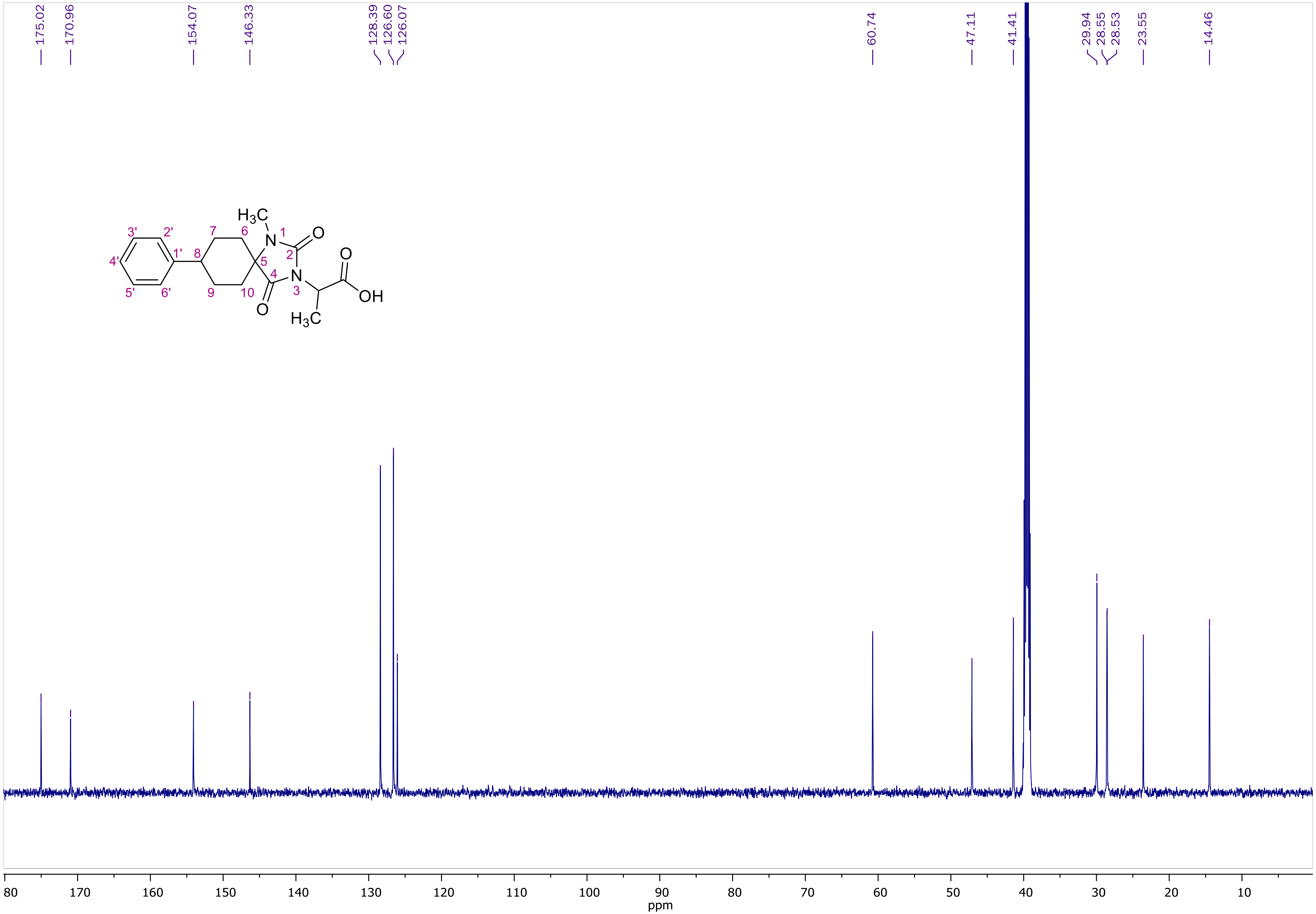

COSY NMR of **51** (400.13 MHz, DMSO-*d_6*)

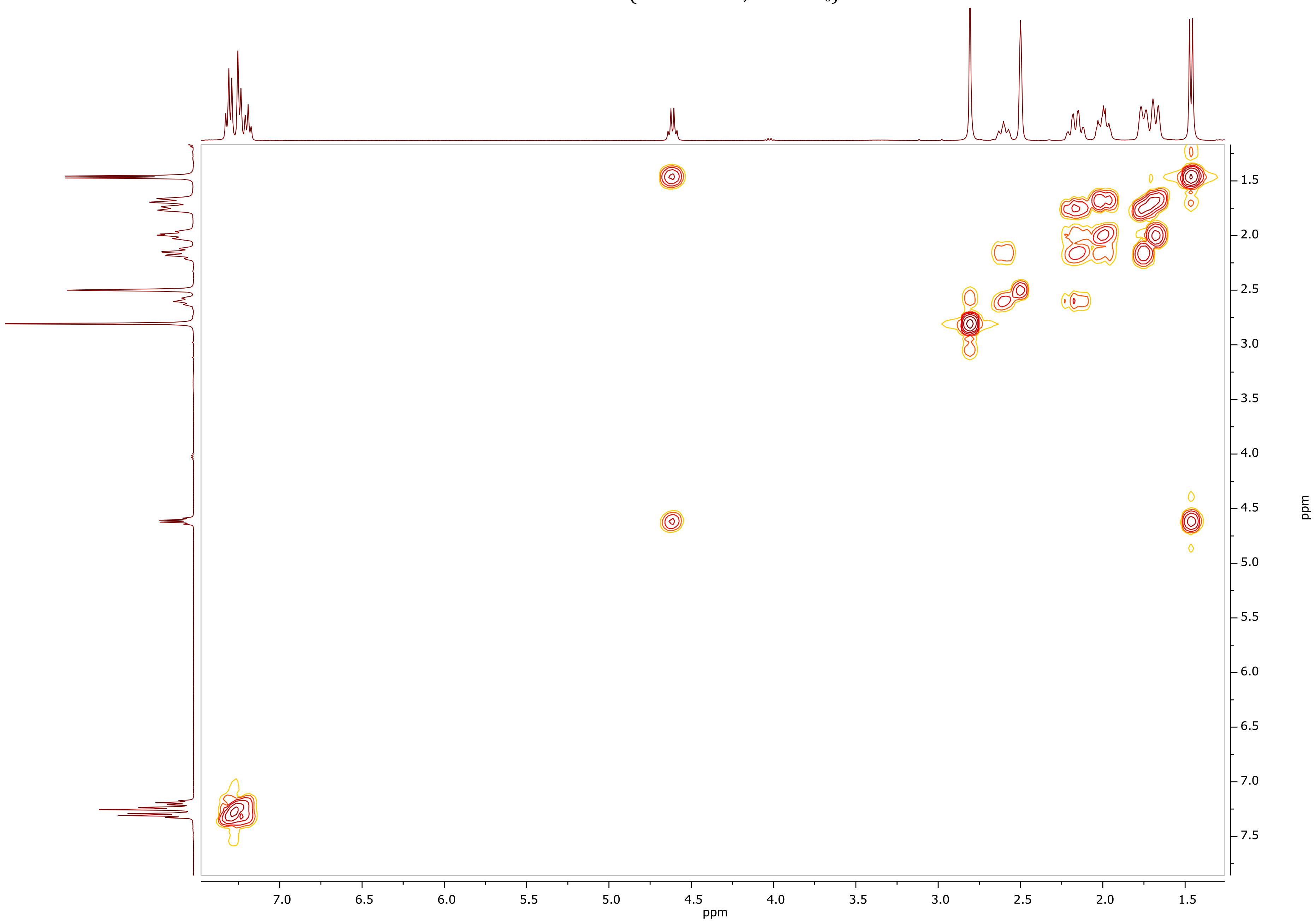

HSQC NMR of **51** (400.13MHz, DMSO-d_6)

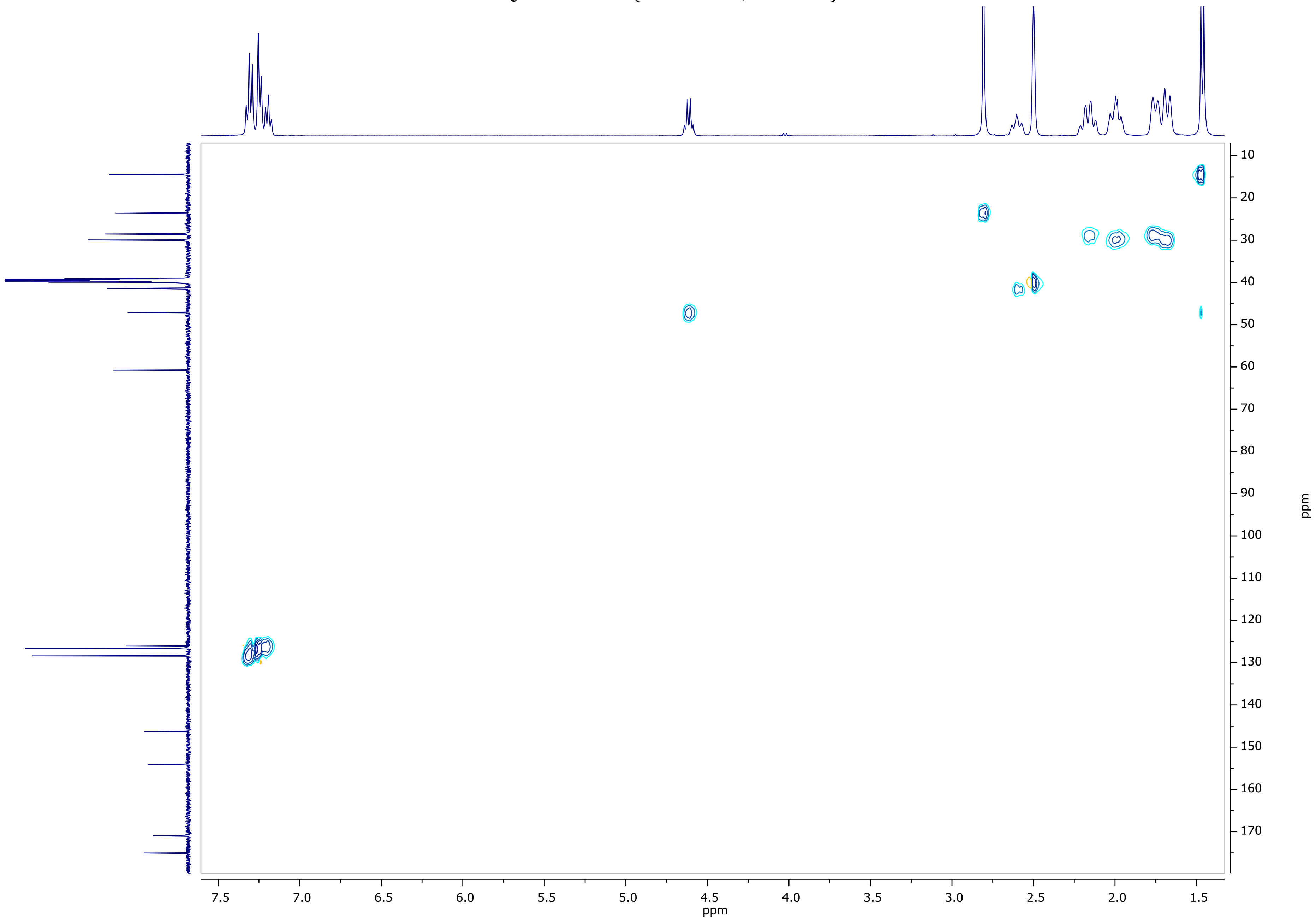

HMBC NMR of **51** (400.13 MHz, DMSO-d_6)

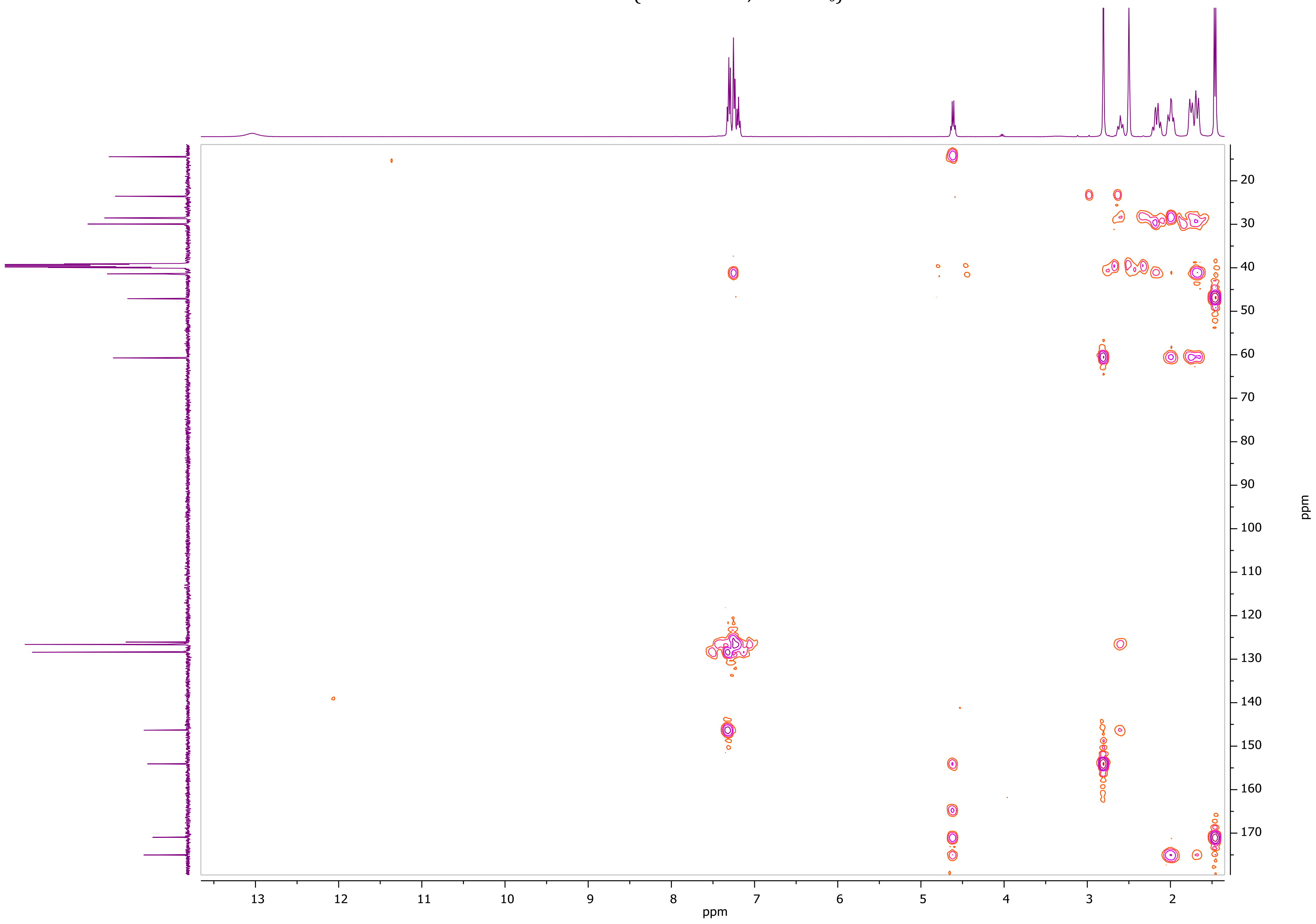

^{1}H NMR of **52** (600.11 MHz, DMSO-d_6)

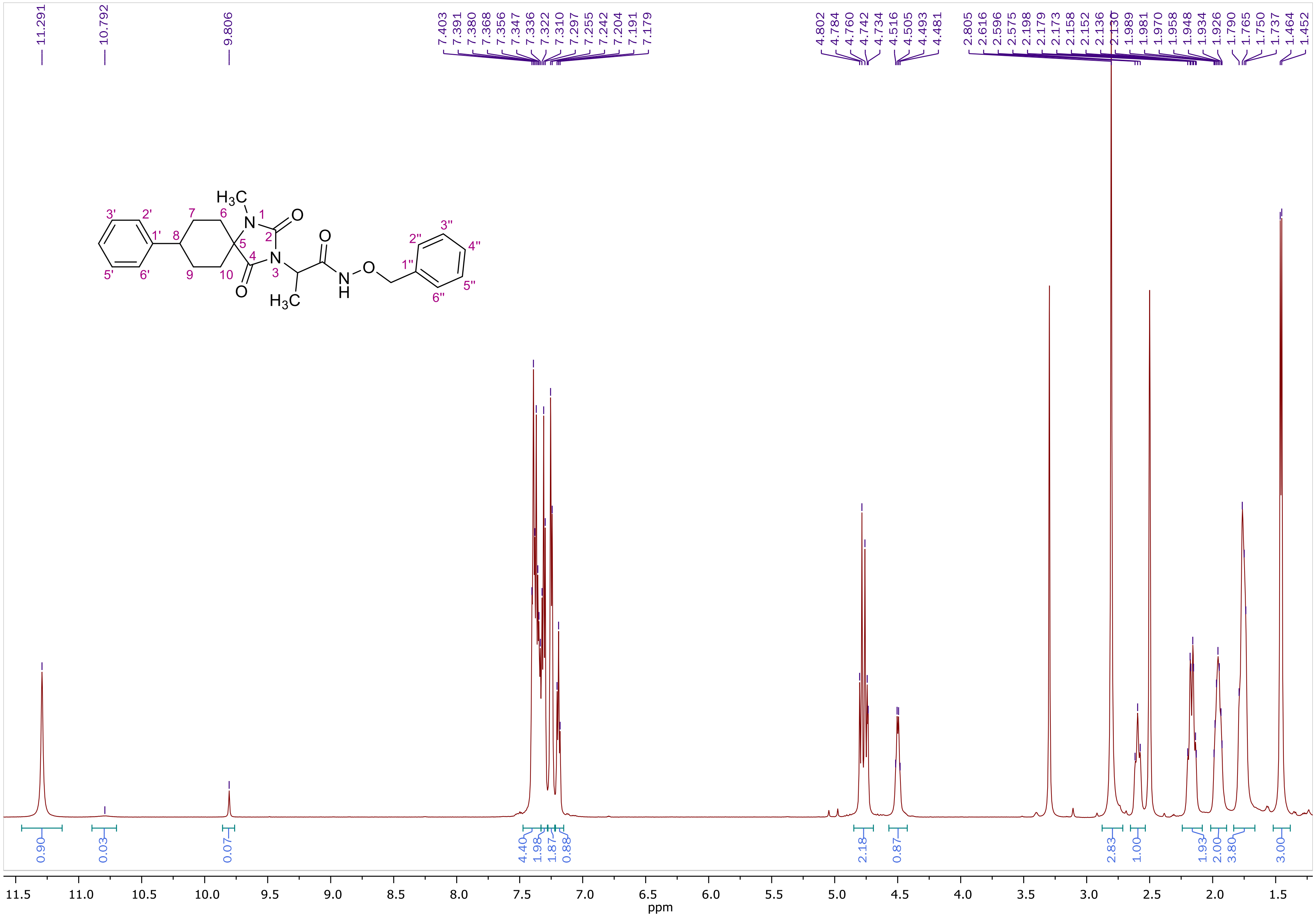

^{13}C NMR of **52** (150.9 MHz, DMSO-d_6)

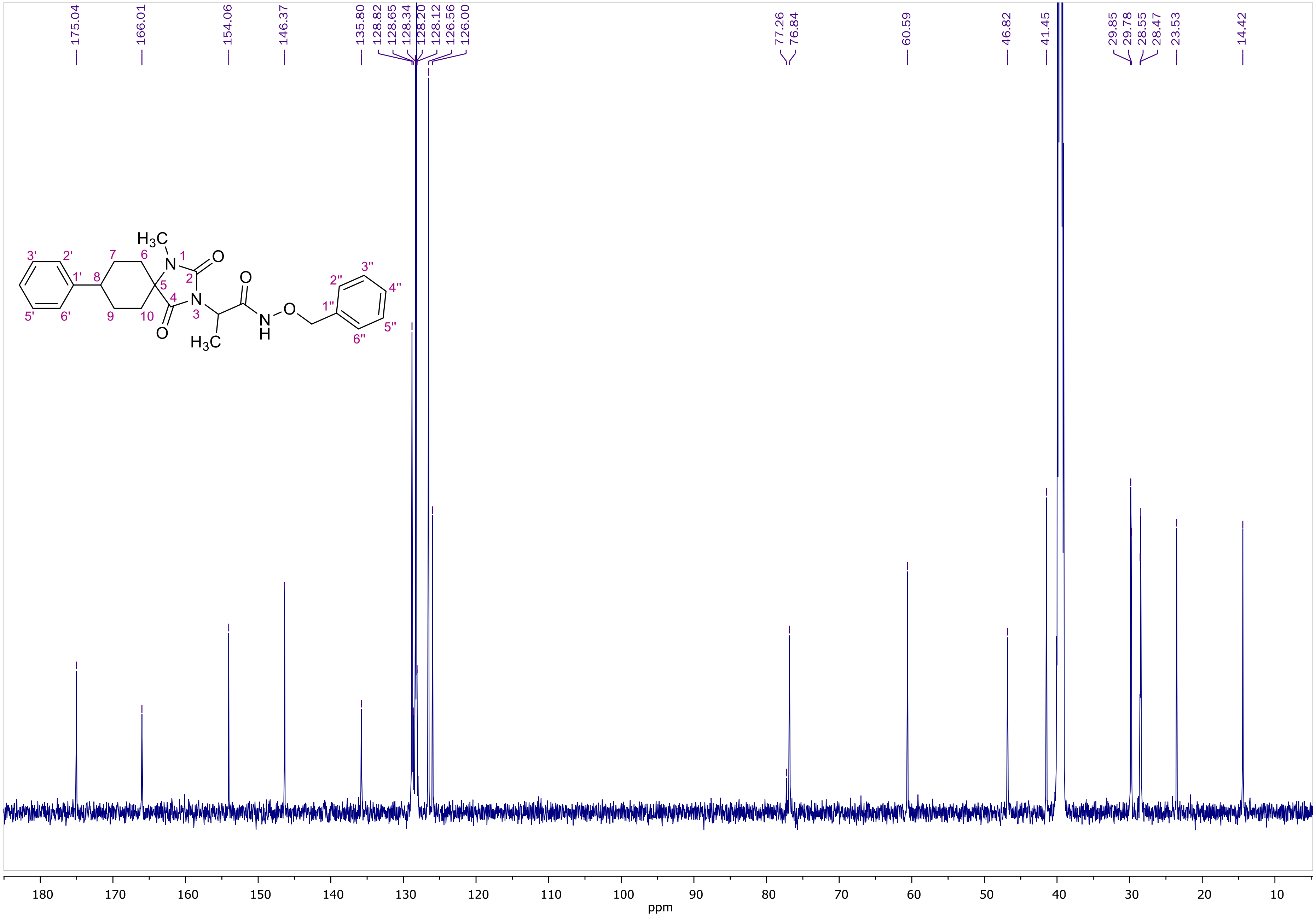

COSY NMR of **52** (600.11 MHz, DMSO-d_6)

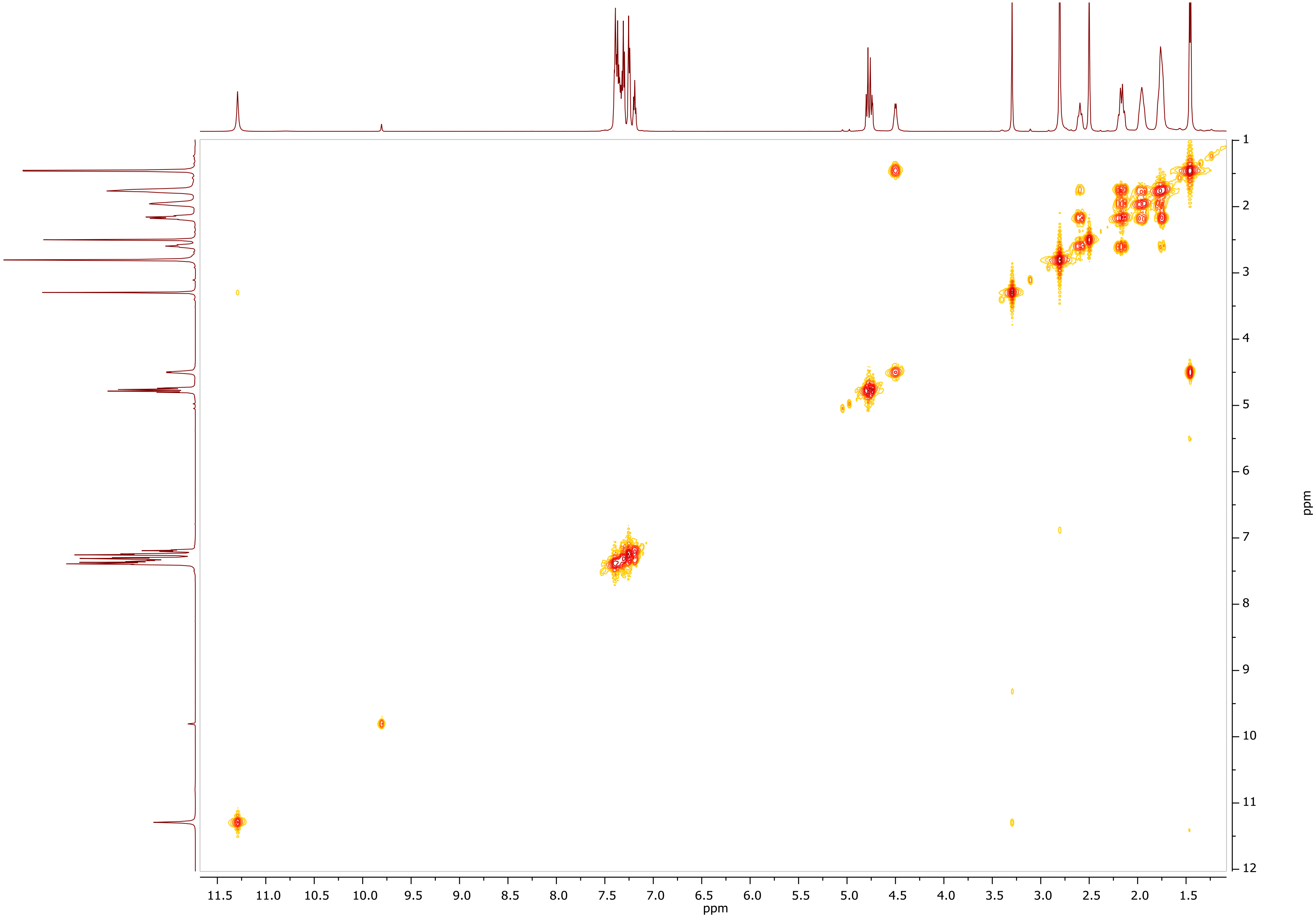

HSQC-DEPT NMR of **52** (600.11 MHz, DMSO-d_6)

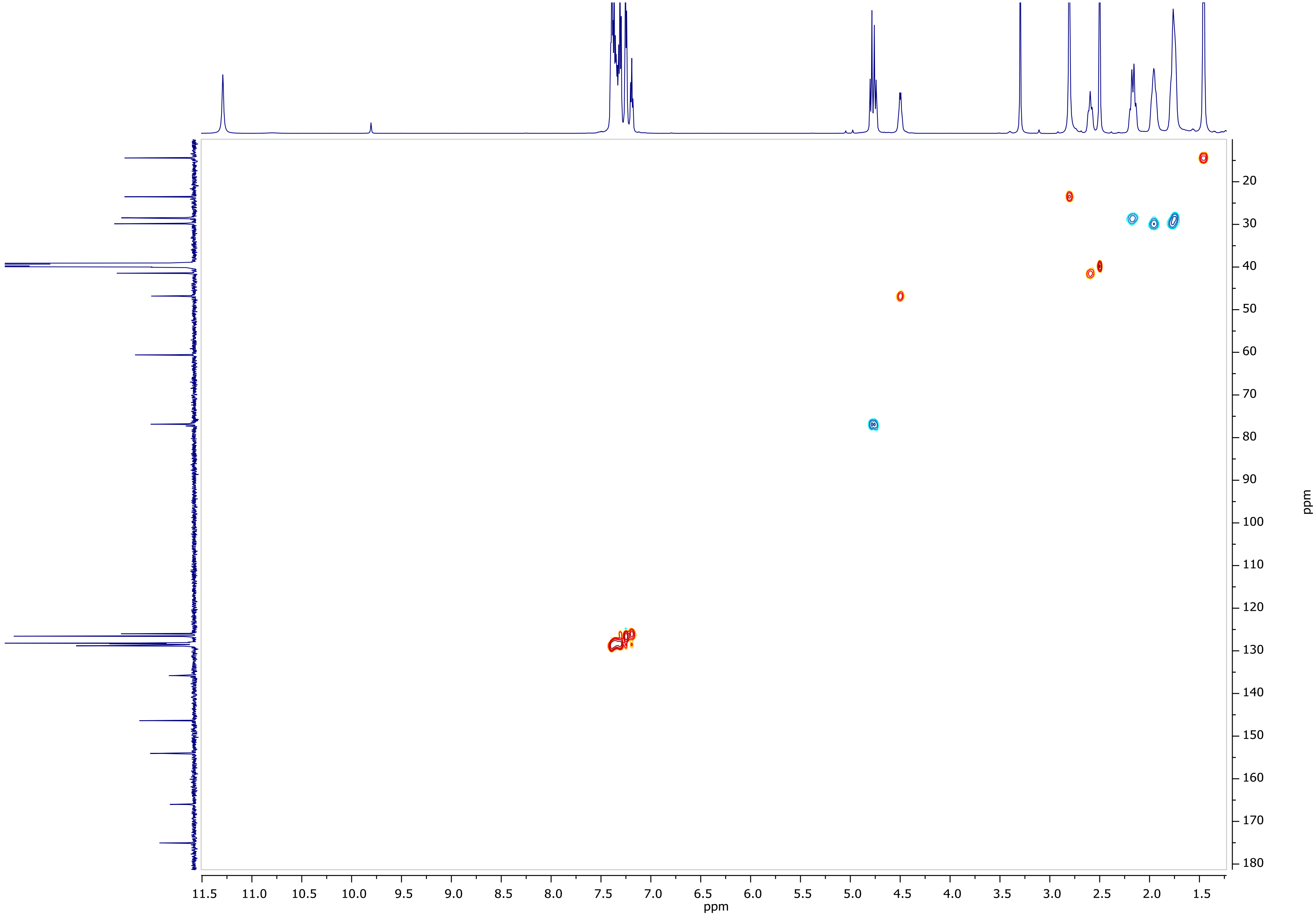

HMBC NMR of **52** (600.11 MHz, DMSO-d_6)

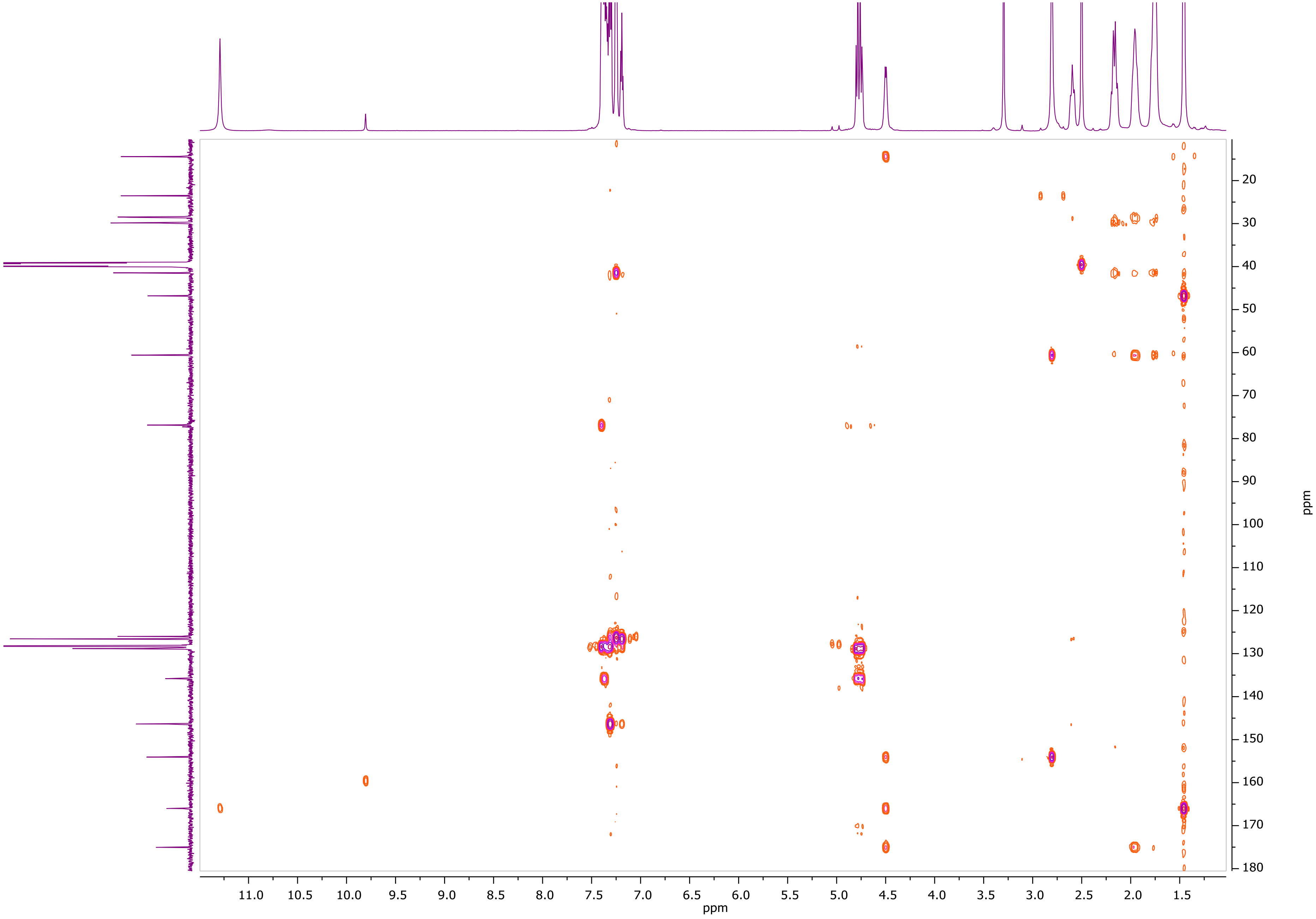

^1H NMR of **53** (600.11 MHz, DMSO-d_6)

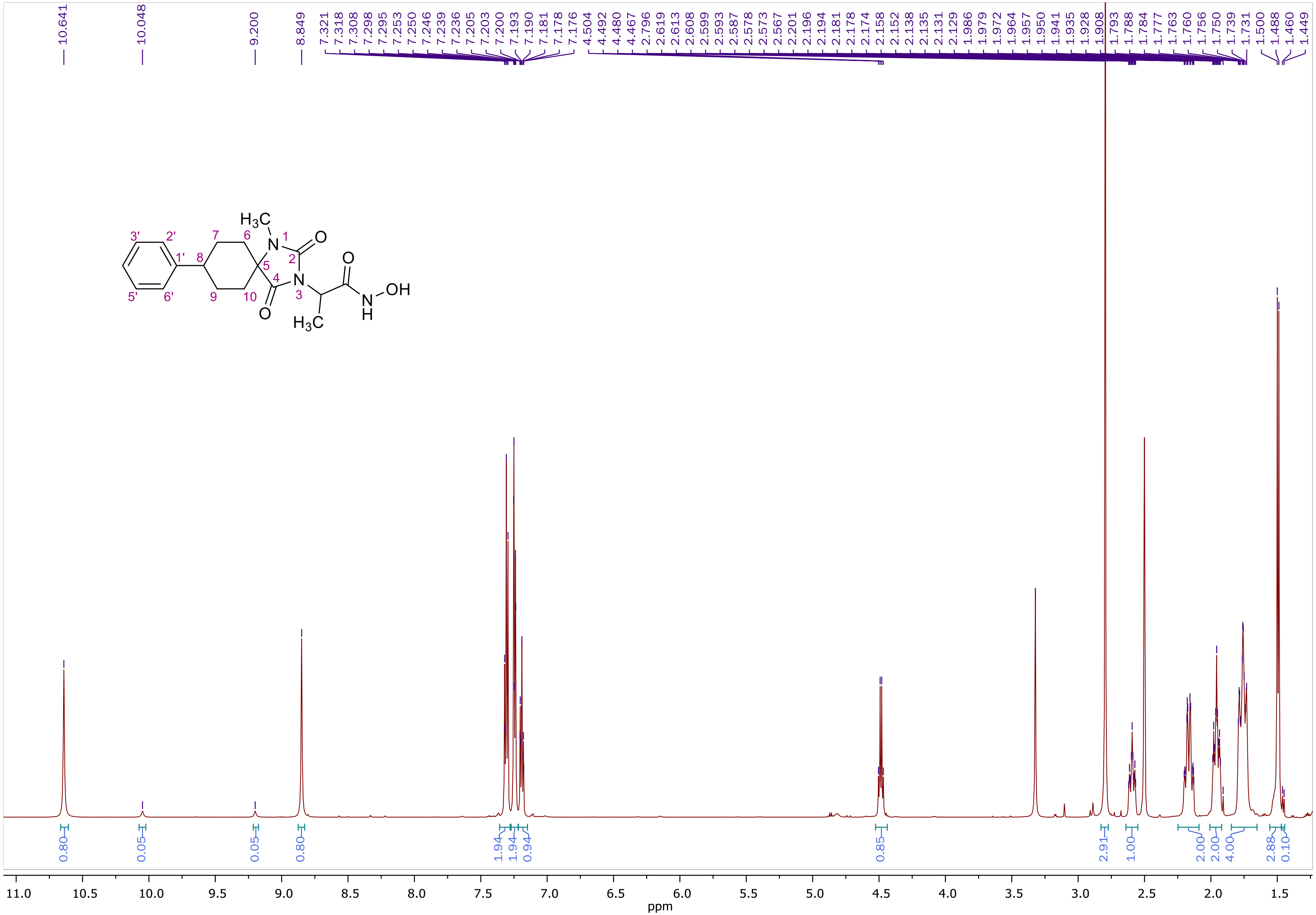

^{13}C NMR of **53** (50.32 MHz, DMSO-d_6)

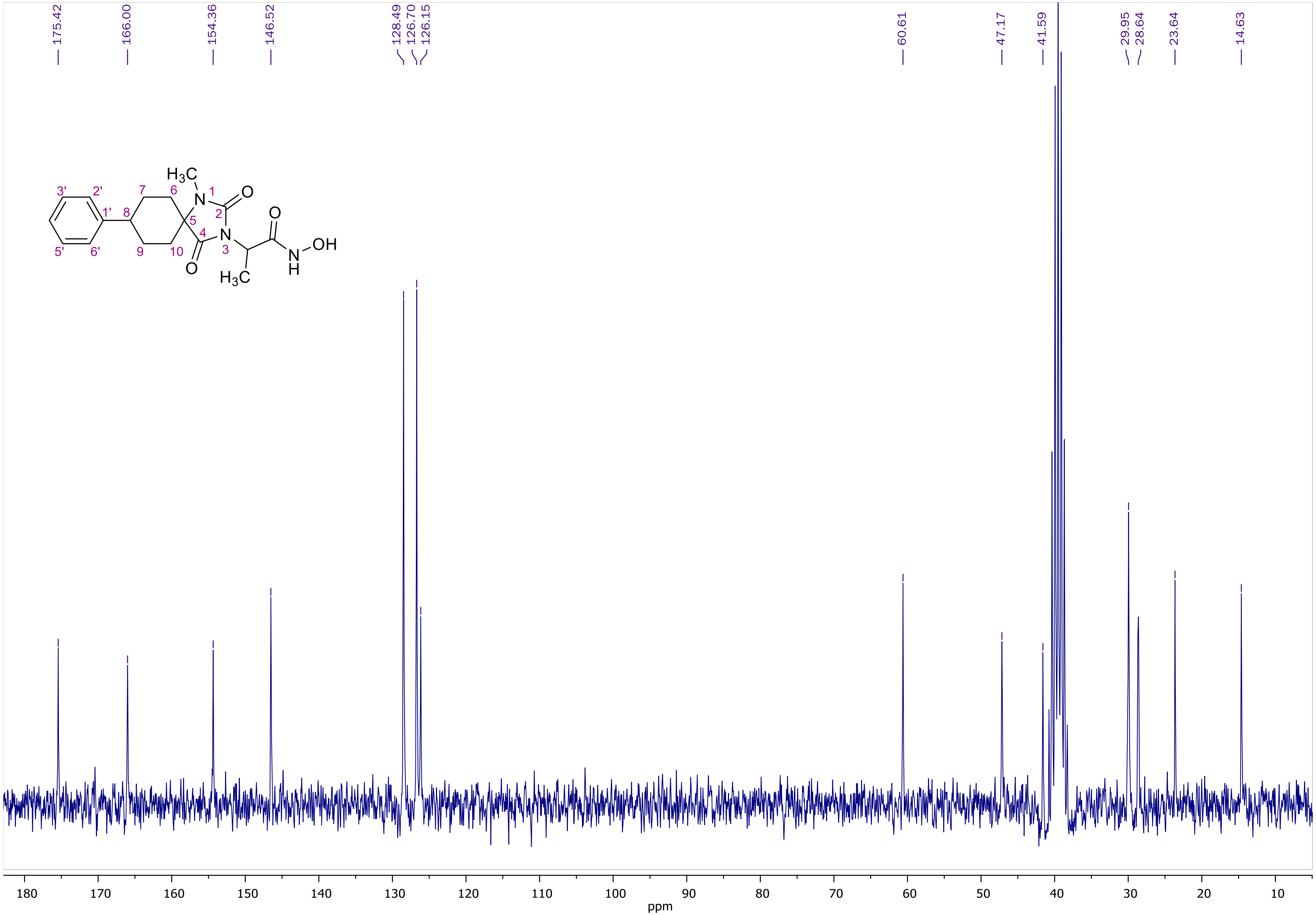

COSY NMR of **53** (400.13 MHz, DMSO-d_6)

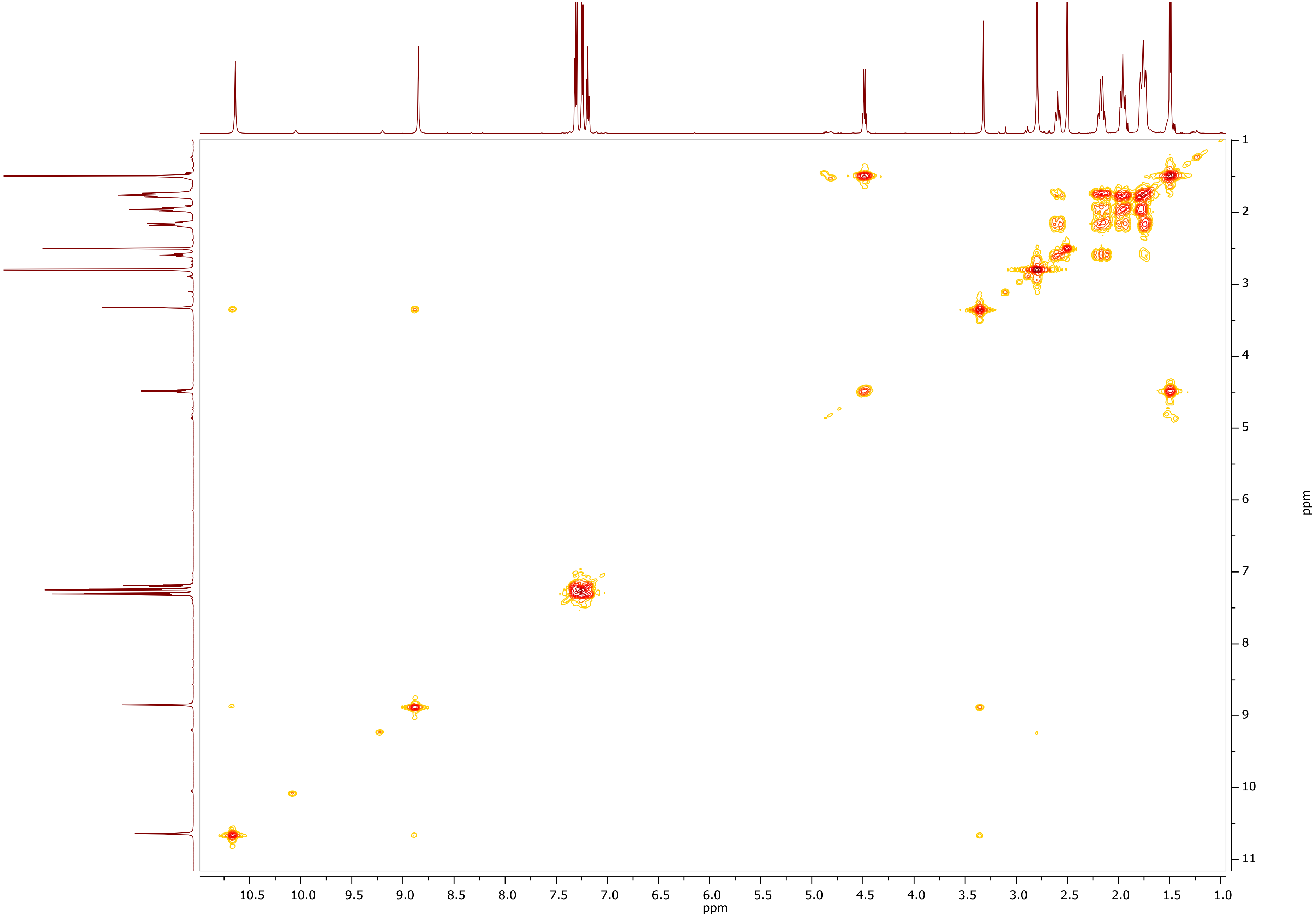

HSQC NMR of **53** (400.13 MHz, DMSO-d_6)

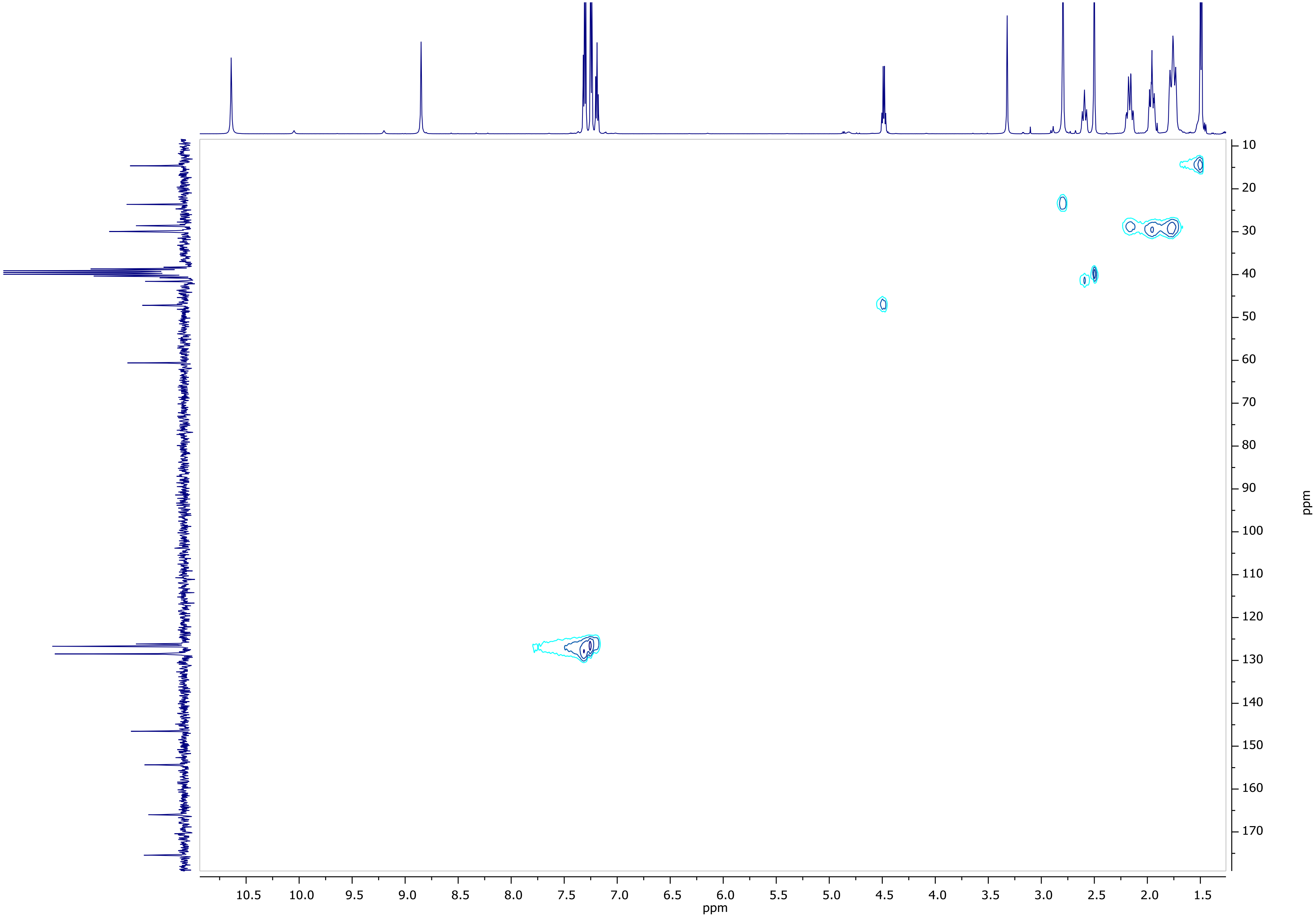

HMBC NMR of **53** (400.13 MHz, DMSO-d_6)

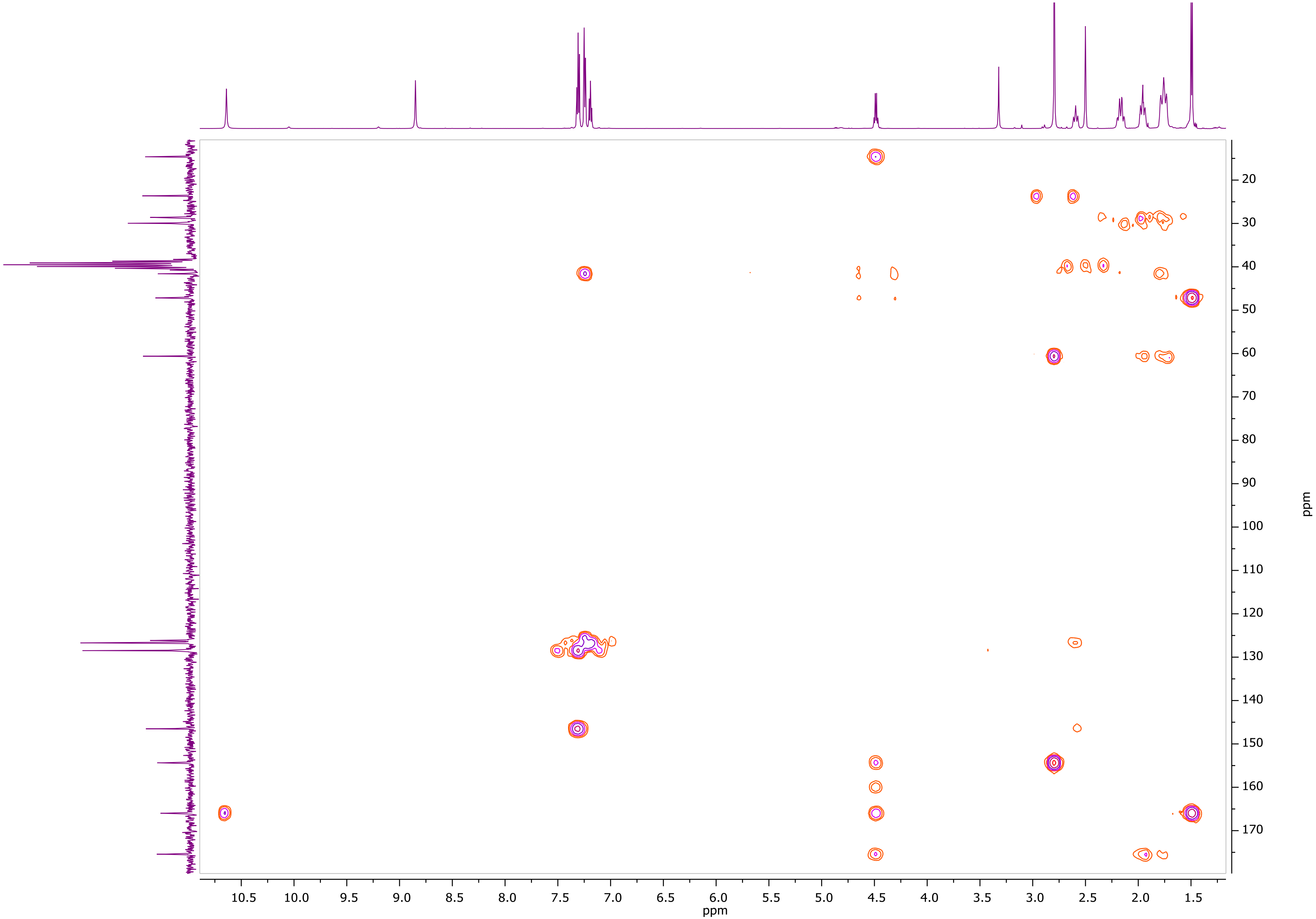

Supplement: Supplementary file 1 [file pharmaceuticals-16-01046-s001.zip › pharmaceuticals-2476481-supplementary.pdf]
